# Qualitative lysine crotonylome analysis in the ovarian tissue of *Harmonia axyridis* (Pallas)

**DOI:** 10.1371/journal.pone.0258371

**Published:** 2021-10-18

**Authors:** Changying Zheng, Lijuan Sun

**Affiliations:** Key Laboratory of Integrated Crop Pest Management of Shandong Province, College of Plant Health and Medicine, Qingdao Agricultural University, Qingdao City, Shandong Province, P. R. China; Aarhus University, DENMARK

## Abstract

Lysine crotonylation (Kcr) is a newly discovered posttranslational modification (PTM), which has been studied at the proteomics level in a few species, with the study of Kcr in female fertility and in insect species is still lacking. *Harmonia axyridis* (Pallas) is a well-known beneficial insect used as a natural biological control agent against aphids in agriculture. Here, global Kcr identification in ovarian tissue of *H*. *axyridis* at diapause stage was performed to reveal potential roles for Kcr in *H*. *axyridis* ovarian cellular processes, female fertility and diapause regulation. In total, 3084 Kcr sites in 920 proteins were identified. Bioinformatic analyses revealed the distribution of these proteins in multiple subcellular localization categories and their involvement in diverse biological processes and metabolism pathways. Carbohydrate and energy metabolism related cellular processes including citric acid cycle, glycolysis and oxidative phosphorylation appeared be affected by Kcr modification. In addition, regulation of translation and protein biosynthesis may reflect Kcr involvement in diapause in *H*. *axyridis*, with Kcr affecting ribosome activities and amino acid metabolism. Moreover, Kcr modulation *H*. *axyridis* ovary development regulation may share some common mechanism with Kcr participation in some disease progression. These processes and pathways were uncovered under diapause stage, but possibly not enriched/specific for diapause stage due to limitations of qualitative proteomics experimental design. Our results informs on the potential for Kcr modifications to regulate female fertility and insect physiology.

## Introduction

It has been well known that protein posttranslational modifications (PTMs) are important regulation pattern of various biological processes and metabolism pathways in life science field [1–3]. To date, more than 400 PTMs have been reported and their roles in diverse cellular processes and life activities have been studied, such as phosphorylation, ubiquitination and glycosylation [1, 4]. The study of PTMs is not only a long history, but also an ongoing career of scientific researchers in both life science and chemical science. With the fast development of analytical instruments (ultra-performance liquid chromatography, high resolution mass spectrometry etc.) and bioinformatics, increasing novel PTMs have been discovered in the latest decade, especially various acylation modifications on lysine including acetylation (Kac), succinylation (Ksucc), butyrylation (Kbu), malonylation (Kma), 2-hydroxyisobutyrylation (Khib) and crotonylation (Kcr) [5, 6].

Among these acylation modifications, Kcr was firstly discovered in mammal histone, which has been proved a conserved histone PTM among various species [7]. In addition, Zhao et al.’s study has indicated histone Kcr participated in gene expression regulation in meiotic and postmeiotic male germ cells, and further influenced spermatogenesis related events [7, 8]. Furthermore, Liu et al. further reported chromo domain Y-like transcription core presser CDYL negatively regulated histone Kcr level, and then postmeiotic gene reactivation and histone replacement events were induced by changed Kcr level; consequently, spermatogenesis and male fertility were influenced [9]. Bao et al. have reported Kcr might interplay with phosphorylation in regulating *E*. *sinensis* spermiogenesis [10].

Non-histone Kcr modification has also been reported in various species and tissues with antibody based affinity enrichment and advanced proteomics tools [11, 12]. Qualitative Lysine crotonylome analysis in zebrafsh larvae showed Kcr may regulate muscle contraction and protein synthesis [13]. Several cell lines based crotonylome studies revealed Kcr involved in multiple cellular functions and biological processes such as DNA repair, RNA processing, chromosome organization, gene expression. hepatocellular carcinoma development, in response to p300 [9, 14–17]. Moreover, in some plant species, plenty plant physiological processes and metabolic pathways including photosynthesis, carbon metabolism and energy production, biosynthesis of antibiotics, amino acids and protein metabolism could be affected by Kcr modification [18–21]. Even some studies of Kcr in diverse species and tissues, especially in male germ cell and tissues, the study of Kcr in ovarian cell and tissues is still lacking and its effects in ovary development and female fertility processes is unknown.

Asian lady beetle *Harmonia axyridis* (Pallas) is a well-known natural enemy of aphids and other pests and has been used as a natural biological control agent in agriculture throughout the world [22, 23]. In insect species, reproductive diapause, a long-term development stagnation stage showing high resistant to various adverse environmental conditions, is a common and important environment adaptation strategy [24]. *H*. *axyridis* also applies this strategy to dispose environmental challenges, such as cold winter [24]. Previous studies indicate *H*. *axyridis* enter adult diapause upon short-day photoperiod condition while go for directly development with long-day photoperiod treatment [25]. However, the molecular mechanism of reproductive diapause in *H*. *axyridis* is still insufficient. Understand the molecular mechanism of diapause regulation could help people manipulate the diapause of *H*. *axyridis* for better insect storage and shelf-life prolonging purpose in agricultural biology control.

Ovary is an import reproductive organ in *H*.*axyridis* and its development degree is closely associated with reproductive diapause assessment [22]. Here, the global Kcr identification at proteomics level in the ovarian tissue of *H*. *axyridis* at diapause stage was performed, with the purpose of demonstrating the potentially roles of Kcr in *H*. *axyridis* ovarian cellular processes, female fertility and diapause regulation. To our knowledge, this is the first Kcr study in ovarian tissues and female fertility system, as well as the first Kcr study in beetle species. Our study not only expanded the species and scope of Kcr study, but also revealed the function of Kcr in female ovarian development, female fertility regulation and insect reproductive diapause.

## Materials and methods

### Experimental insects

The animal protocol was approved by the Institution Animal Care and Use Committee of Qingdao Agricultural University. The *H*. *axyridis* used in this study were captured from an organic vegetable planting farm in Laixi City, Shangdong Province, China. Lady beetles were reared at a laboratory condition (25 ± 1°C, 60–70% relative humidity, long day photoperiod of 16 h light—8 h dark, fed on *Myzus persicae* (Sulzer)) for two generations to obtain stable *H*. *axyridis* population. Then the following third generation was treated with a short-day photoperiod (10 h light—14 h dark) at 20°C to induce reproductive diapause [22, 25, 26]. At the 20th day after eclosion, the female *H*. *axyridis* were collected and the ovarian tissues of these beetles were carefully peeled follow previous report [22]. To verify the success of reproductive diapause induction, a morphologic observation for *H*. *axyridis* internal reproductive organs after diapause induction were performed and compared with that in non-diapause group treated with long day photoperiod [22]. About 100 mg ovarian tissues collected from 40 *H*. *axyridis* at diapause stage were mixed and used a biological replication. Three biological replications were performed. After washing with freezing 0.01 M PBS buffer, the tissues were thrown into liquid nitrogen and kept at -80°C for future use.

### Sample preparation for Kcr analysis

Protein extraction and digestion were performed with previous report with some modifications [13]. Briefly, 100 mg ovarian tissues were ground into power with liquid nitrogen and transferred to a tube. Then lysis buffer containing 8 M urea, 2 mM EDTA, 3 μM TSA, 50 mM NAM, 10 mM DTT and 1% protease inhibitor mixture was added into the power and sonicated on ice for 5 times. After 15 min centrifugation at 12 000 g at 4°C, the supernatant was transferred to a new tube and the cell debris was discarded. The protein concentration was assayed with 2-D Quant kit (GE Healthcare) referring the manufacturer’s instructions.

The protein solution (3 mg protein) was firstly reduced with 10 mM DTT (1 h, 37°C) and then alkylated with 20 mM iodoacetamide (45 min, room temperature in darkness). The 100 mM TEAB was used to make the urea concentration of protein solution less than 2 M and then trypsin (Promega, Madison, WI, USA) was added with 1:50 trypsin-to-protein mass ratio to digest the protein. After 4 h digestion at 37°C, a second digestion procedure with 1:100 trypsin-to-protein mass ratio and overnight incubation at 37°C was performed to fully digest the protein mixture. The resulted peptide mixtures were cleaned with C18 SPE column and vacuum dried.

To enrich Kcr peptides, peptides were dissolved in the immunoprecipitation (IP) buffer (1 mM EDTA, 50 mM Tris-HCl, 100 mM NaCl, 0.5% NP-40, pH 8.0). The re-suspended peptide mixture was incubated with pre-washed pan-crotonylation antibody-conjugated resin beads (WM402, Micron biotech, Hangzhou, China) at 4°C overnight with gentle rotation. Following the incubation, the resin beads were washed with IP buffer (4 times) and clean water (2 times) to deplete the nonspecific binding peptides. Then the enriched Kcr peptides were eluted with 0.1% trifluoroacetic acid (TFA) and vacuum dried [27]. The Kcr peptides were desalted and cleaned with C18 ZipTips (Millipore) following the manufacturer’s instructions.

### Western blot analysis

Each lane in 10% polyacrylamide SDS gel was loaded with 20 μg protein. Following electrophoresis, proteins were transferred to a polyvinylidene difuoride membrane and blocked with %5 skim milk. The embrane was incubated with pan anti-Kcr antibodies for 6h at room temperature (1:2500 dilution). After washed for five times with 20 mM Tris, the membrane was incubated with a secondary horseradish peroxidase-conjugated antibody at for 2 hours (1:20000 dilution) [14].

### LC-MS/MS analysis

All the peptides mixture enriched form aforementioned IP was dissolved in mobile buffer A (0.1% FA in water) and the supernatant was transferred into the auto-sampler vial. The data acquisition was performed with an Ultimate RSLCnano 3000 UPLC system combining Q Exactive HFX (ThermoFisher Scientific, America) and a reversed-phase analytical column (Thermo, Acclaim PepMap RSLC C18 column, 2 μm, 75 μm×50 mm). The peptides were eluted with a linear gradient of 5–20% buffer B (0.1% FA in 98% ACN) in 30 min, 20–35% in 10 min, and increased to 85% in 5 min, then maintained at 85% for 3 min.

The parameters for mass spectrometer were given as follows. The electrospray voltage was 2.0 kV and the capillary temperature was 250°C. Data dependent acquisition mode was used which alternated between one MS scan followed by 20 MS/MS scans. The resolution for MS1 was set as 70, 000 and the scan range was set as m/z 350–1800, combined with 50 ms max injection time (MIT) and automatic gain control (AGC) threshold 1E6. The HCD energy for the MS2 ion production was set as 26% normalization collision energy. Other MS2 parameters: dynamic exclusion 15.0 s, first fixed mass 110 m/z, resolution 17500, MIT 200 ms and AGC 1E5.

### Database searching

The database searching was performed with MaxQuant (v.1.5.2.8) software against the *H*. *axyridis* transcriptome data (NCBI, BioProject accession number PRJNA707403) concatenated with a reverse decoy database. The specified digestion enzyme was Trypsin/P with up to 4 missing cleavages. Mass error for precursor ions and fragment ions was 10 ppm and 0.02 Da, respectively. Fix modification was set as carbamidomethylation on cysteine and variable modifications were set as crotonylation on lysine, oxidation on methionine, and acetylation on protein N-terminal. False discovery rate thresholds for proteins/peptides/modification sites were set at 1%. The applied minimum peptide length was 7. All other parameters were used as software default.

### Bioinformatics

The analysis of sequences around the Kcr site was performed with Motif-X software [28]. Protein function annotation classification was performed based on Gene Ontology (GO) database [29]. Kyoto Encyclopedia of Genes and Genomes (KEGG) database was used to annotate protein pathway with BlastKOALA tool provided by KEGG (https://www.kegg.jp/blastkoala) [30, 31]. Subcellular localization of the Kcr proteins was performed with WoLF PSORT software [32]. Enrichment analyses were conducted by DAVID (the Database for Annotation, Visualization and Integrated Discovery) software and corrected p-value < 0.05 is regarded as significant for all the enrichment analysis [33].

## Results and discussion

### Identification of Kcr in *H*. *axyridis* ovarian tissues

**Fig 1:**
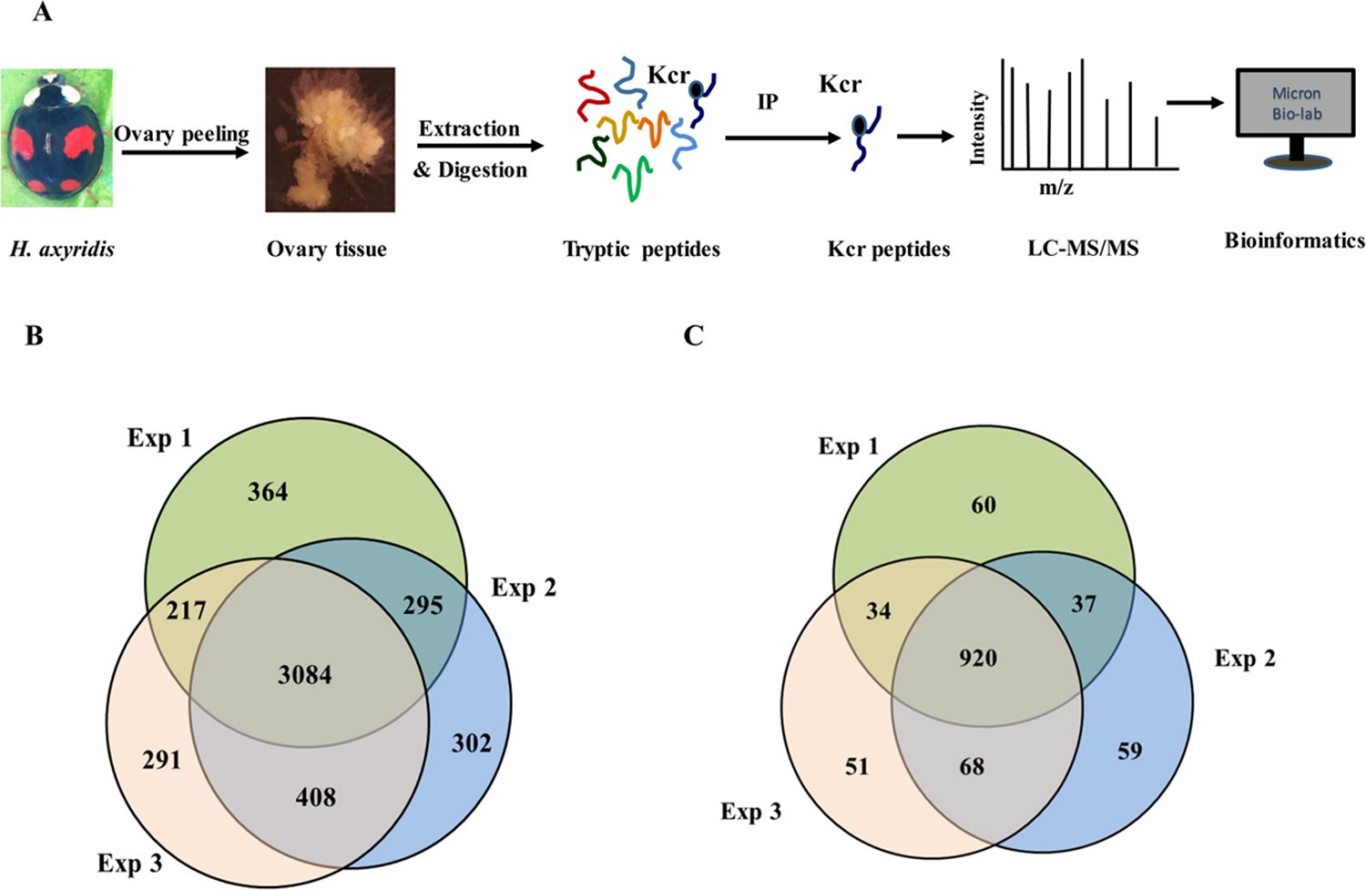
Identification of the lysine crotonylation in the ovarian tissues of *H*. *axyridis* at diapause stage. (A) Experimental design and overall workflow. (B) Venn diagram of the identified crotonylated sites. (C) Venn diagram of the identified crotonylated proteins.

In the presented study, a systematic Kcr identification was performed in the ovarian tissue of *H*. *axyridis* at reproductive diapause stage, with the purpose of revealing the potential role of Kcr in insect reproductive diapause, ovarian development and female reproductive biology. The overall experimental design was shown in Fig 1A, which consisted of 6 steps. In brief, *H*. *axyridis* was laboratorial reared and introduced into reproductive diapause phase with short-day photoperiod treatment. Then the ovary tissues were carefully peeled for Kcr sample preparation. Following protein extraction and trypsin digestion, an affinity enrichment process was performed to capture Kcr peptides. Finally, the Kcr peptides were analyzed by LC-MS/MS and interpreted with bioinformatics tools. All the MS data were deposited to ProteomeXchange Consortium via the PRIDE partner repository (http://www.proteomexchange.org, accession number PXD024421).

The development arrestment of internal reproductive organs is one of the most remarkable features of insects’ diapause [23]. To ensure the *H*. *axyridis* had remained diapause stage after short-day photoperiod induction, a morphological observation for the internal reproductive organs was conducted (S1 Fig). The internal reproductive organs in diapause *H*. *axyridis* (S1A Fig) were markedly inhibited compared with that in reproductive *H*. *axyridis* (S1B Fig). The ovary was infertile and no recognizable egg chambers or yolk were observed in the ovarioles. In brief, the *H*. *axyridis* had entered in a diapause state after induction.

Altogether, 4961 Kcr sites in 1229 proteins were identified in three biological replicates and 3084 sites in 920 proteins were repeatedly identified (Fig 1B and 1C), showing Kcr was a widespread PTM in *H*. *axyridis* ovarian tissue. All the Kcr sites and proteins were listed in supplementary information (S1 Table).

To validate the MS data, a western blotting detection for the Kcr proteins in *H*. *axyridis* ovarian tissue was performed with pan anti-Kcr antibody (S2 Fig). In accordance with a plenty of Kcr sites and proteins in MS data, diverse bands with various molecular weights were observed, further indicated the wide distribution of Kcr modification in the ovarian tissue of diapause *H*. *axyridis*.

The numbers of identified Kcr site and proteins in others species and tissues have been reviewed in previous reports [12, 34]. Without doubt, there are large variances in the statistic data among different species and tissues, which could be attributed to some factors such as the different intrinsic Kcr level of proteins, experiment material treatment difference, protein expression species and/or tissues specificity, and preferential distribution of diverse crotonyltransferase and decrotonylase.

### Sites properties analysis of the Kcr peptides

**Fig 2:**
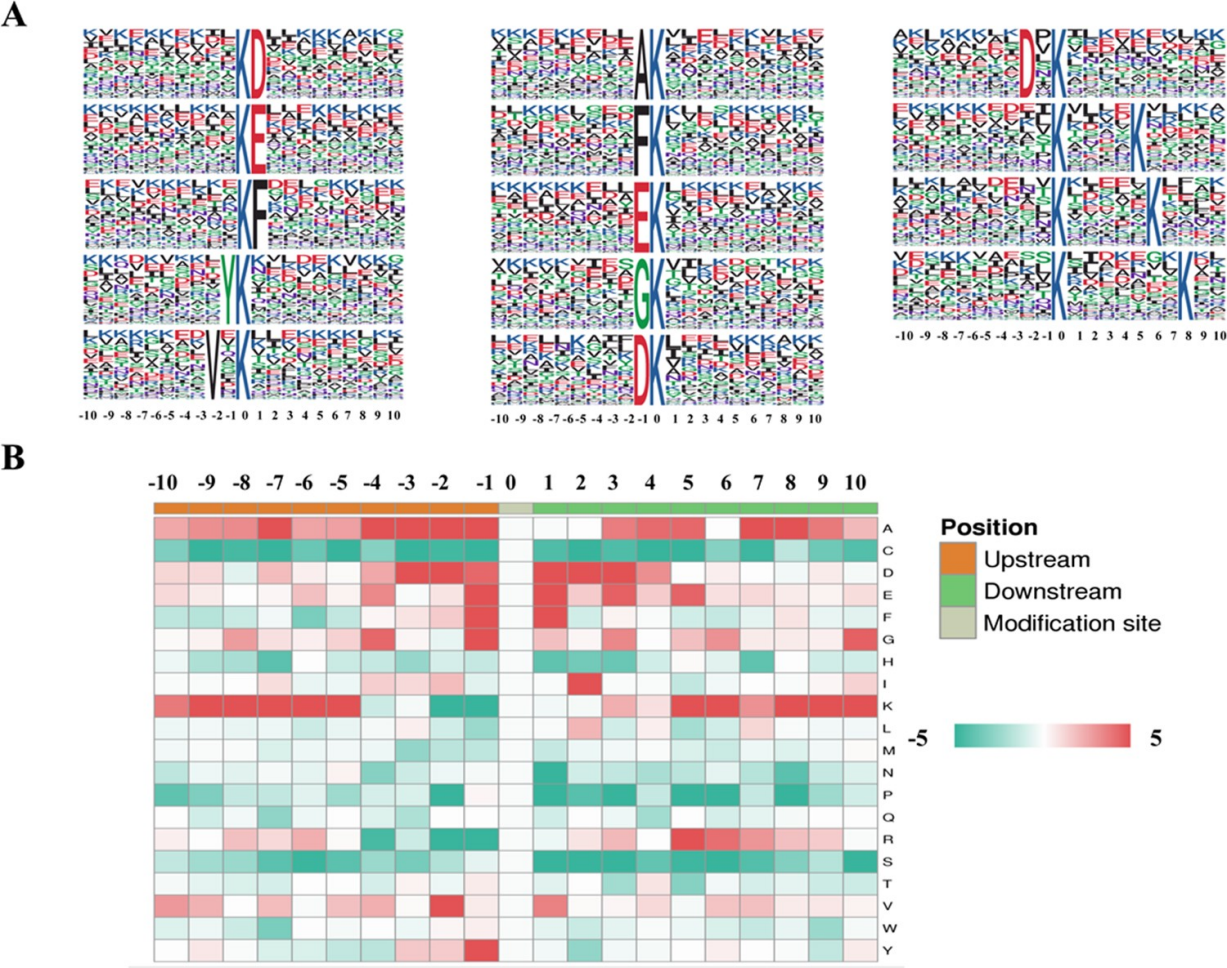
Structure analyses of the crotonylated peptides. (A) Crotonylation sequence motifs analysis. The letter height represents the frequency of that amino acid residue at that position. The K in the middle position indicates the cronylated lysine. (B) Heat map analysis. Red indicated the amino acid is significantly enriched while green indicates the amino acid is significantly reduced.

To evaluate the features of the Kcr peptides in *H*. *axyridis* ovarian tissue, motifs in all identified crotonylated peptides were analyzed. As shown in Fig 2A, a total of 14 conserved motifs were extracted. The 14 motifs can be classified into four subgroups according to the chemical properties of conserved amino aides surrounding Kcr sites. Five acidic amino acids (aspartate, D; glutamate, E) containing sequence moifs were obtained, including KcrD, KcrE, DKcr, EKcr and D*Kcr (Kcr indicates a crotonylated lysine, * indicates a random amino acid residue), imply acidic amino acids are more compatible with Kcr sites. Besides, 3 basic amino acid (lysine, K) containing motifs (Kcr****K, Kcr*****K, Kcr*******K) and 4 hydrophobic amino acids (phenylalanine, F; valine, V; tyrosine, Y) containing motifs (YKcr, V*Kcr, FKcr, KcrF) were extracted. Moreover, 2 motifs with resides bearing smaller side chain groups (glycine, G; alanine,A) were screened (AKcr, GKcr).

Consisted with the motif results, the aforementioned conserved residues were also significantly enriched in the positions surrounding the Kcr sites in the heat map analysis of the distribution frequency of various amino acids from -10 to +10 positions adjacent Kcr sites (Fig 2B). Enrichment of D was observed from -3 to +3 regions and enrichment of E was observed at -1, +1, +3 and +5 positions. K was overrepresented at the relatively farther positions (-10 to -5 and +5 to +10) from Kcr site. F,Y and V were mainly aligned to ± 1 and/or ± 2 positions. A and G were frequently distributed at multiple positions around Kcr sites.

We compared our results with other studies among various species and tissues. It seems that the site properties of Kcr peptides showed some similarity among diverse species in the aspects of both sequence motif and amino acid distribution heat map [9–11, 18, 19, 27]. For instance, D/E containing motifs were frequently observed and D/E were significant enriched at the very near position of Kcr sites in different species; K containing motifs at the relatively far positions around Kcr were another common motifs among various species and tissues, whose corresponding heat map results were dominantly enriched at -10 to -5 and + 5 to +10 positions.

### Function classification and subcellular location analysis

**Fig 3:**
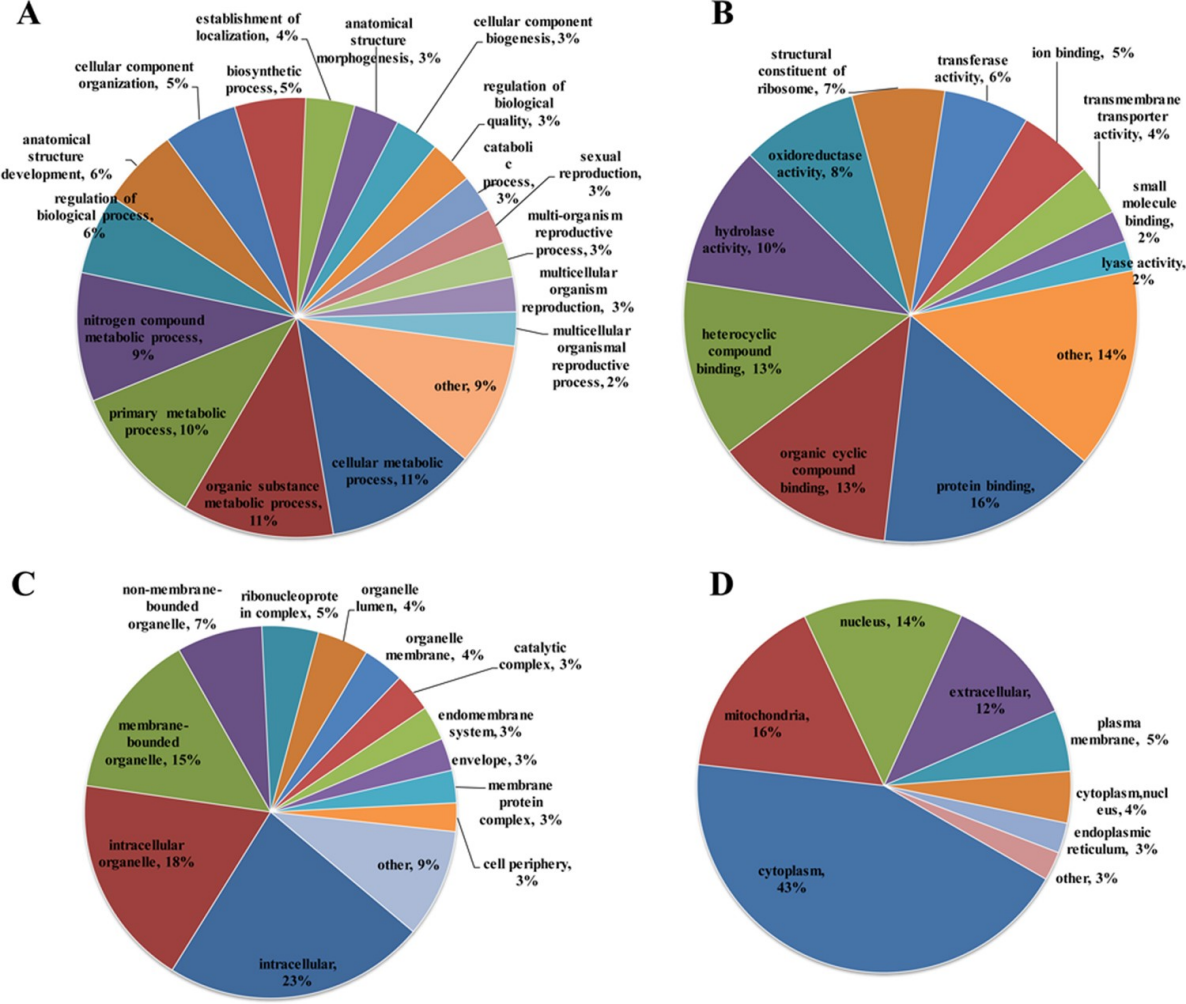
Classification and subcellular localization analysis. (A) Biological processes classification. (B) Molecular function classification. (C) Cellular component classification. (D) Subcellular localization.

Based on the Gene Ontology (GO) annotation, function classification on the ontology of biological process, molecular function and cellular components was performed to primarily reveal the functions of Kcr modified proteins in the reproduction arrested *H*. *axyridis* ovary. As shown in Fig 3A–3C, Kcr proteins showed a wide range of distribution in the analyzed categories. However, because of the limitations of qualitative proteomics, the uncovered GO terms under diapause stage possibly not specific for diapause regulation. In biological process category (Fig 3A), metabolism related processes (48%) accounted for the largest ratio, which included cellular metabolism (11%), organic substance metabolism (11%), primary metabolism (10%), nitrogen compound metabolism (9%), biosynthetic process (5%) and catabolic process (3%). Cellular component/anatomical structure organizing and development related processes (21%) were another major group where Kcr proteins were distributed, including anatomical structure development (6%), cellular component organization (5%), localization establishment (4%), anatomical structure morphogenesis (3%) and cellular component biogenesis (3%). What’s noticeable was that 4 reproduction related processes (sexual reproduction, multi-organism reproductive process, multicellular organism reproduction and multicellular organismal reproductive process) were observed whose sum percentage reached 14%. In addition, 9% Kcr proteins were aligned to biological regulation, consisted of regulation of biological process (6%) and regulation of regulation of biological quality (3%). Other biological processes related Kcr proteins accounted for 9%. The result indicated the material metabolism, cellular component/anatomical structure morphogenesis, development and organizing, and reproduction related biological processes of ovary may be influenced by Kcr modification in *H*. *axyridis*.

In the level of molecular function, the majority of Kcr proteins were binding and enzyme/transporter activity related (Fig 3B), whose sum percentage was 49% and 30%, respectively. Binding related functions involved in the binding to protein (16%), organic cyclic compound (13%), heterocyclic compound (13%), ion (5%) and small molecule (2%). Relevant enzyme/transporter groups contained hydrolase (10%), oxidoreductase (8%), transferase (6%), trans-membrane transporter (4%) and lyase (2%). Besides, 7% Kcr proteins were assigned to structural constituents of ribosome and 14% Kcr proteins were classified into others group.

For cellular component analysis, the Kcr proteins were mainly distributed at intracellular (23%), intracellular organelle (18%) and membrane-bounded organelle (15%). Follow are non-membrane-bounded organelle (7%). In addition, some subcellular complex located Kcr proteins were detected whose sum proportion reached 11%, including ribonucleoprotein complex (5%), catalytic complex (3%) and membrane protein complex (3%). Moreover, 14% organelle structure or component related Kcr proteins were found, such as organelle lumen (4%), organelle membrane (4%), endomembrane system (3%) and envelope (3%). Cell periphery located Kcr proteins were quite less; whose percentage is only 3%. Other cellular components located Kcr proteins accounted for 9% in all the identified proteins. The cellular component analysis showed Kcr proteins were widely distributed at diverse cellular components, subcellular structure and complex.

Subcellular localization result (Fig 3D) showed the cytoplasm proteins were the dominant Kcr proteins (43%), whose proportion was much higher than the others. Following were mitochondria (16%), nucleus (14%) and extracellular (12%). Other subcellular compartments located Kcr proteins were much less, such as 5% plasma membrane (5%) endoplasmic reticulum located proteins (3%).

### GO and KEGG enrichment analysis

**Fig 4:**
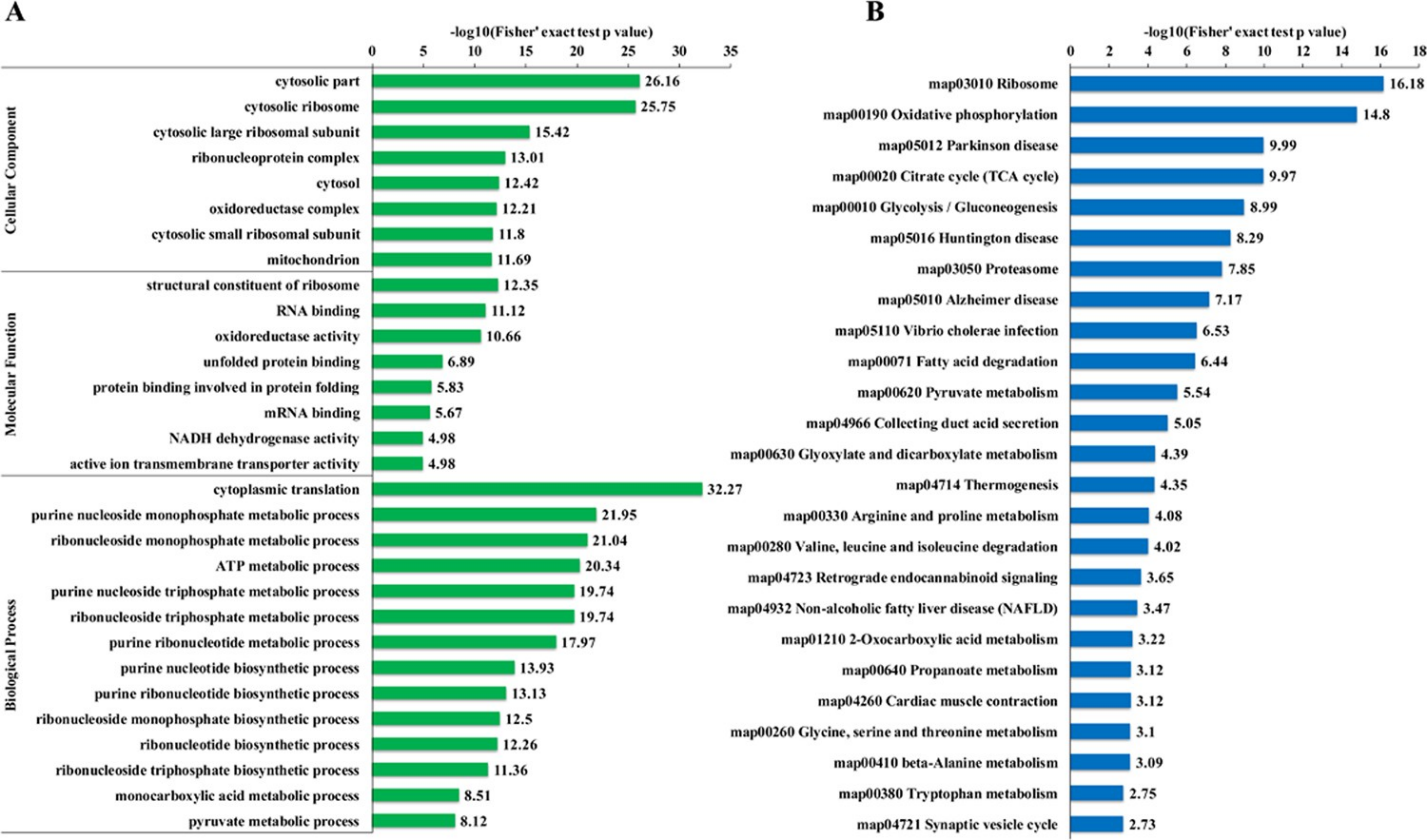
Enrichment analyses. (A) GO annotation based enrichment in the category of biological process, molecular function and cellular components. (B) KEGG pathway based enrichments analysis.

To comprehensively demonstrate the function and role of Kcr proteins in *H*. *axyridis* ovarian, GO and Kyoto Encyclopedia of Genes and Genomes (KEGG) pathway annotation based enrichment analysis were performed (Fig 4). In cellular component level (Fig 4A), cytosolic part was the most significantly enriched component, implying biological events within cytosolic part appeared was influenced by Kcr modification. Besides, ribosome related components were markedly enriched, which included cytosolic ribosome, ribonucleoprotein complex, cytosolic large ribosomal subunit and small ribosomal subunit. On the category of molecular function, the top three dramatically enriched functions were structural constituent of ribosome, RNA binding and oxidoreductase activity. Besides, two protein binding related terms were enriched. The enriched structural constituent of ribosome, RNA/protein binding terms suggested translation and protein biosynthesis related processes probably was affected by Kcr in *H*. *axyridis* ovarian tissues, which was consistent with multiple ribosome related terms in cellular component analysis. Previous study in pluripotent stem cells showed a similar observation that RNA binding proteins are widely crotonylation modified [35]. Moreover, in the biological process level, cytoplasmic translation displayed the highest enrichment score. Notably, as many as 11 nucleotide metabolism or biosynthesis related processes were markedly enriched, especially purine ribonucleotide related terms. In the category of biochemistry, amino acid adenylation, which the metabolism of adenosine triphosphate (ATP) and adenosine monophosphate (AMP) are involved, is the initial procedure of translation and protein biosynthesis [36]. These dramatically enriched purine ribonucleotide related biological processes imply the amino acid adenylation process in *H*. *axyridis* ovarian tissues probable was influenced by Kcr modification, and further translation and protein biosynthesis were regulated.

In the KEGG pathway analysis (Fig 4B and S2 Table), ribosome was the top significantly enriched pathway, which was consistent with the dramatically enriched ribosome and translation related GO terms in GO enrichment analysis (Fig 4A). In addition, protein degradation related proteasome pathway; and several amino acid metabolism related pathway were enriched as well. The involved amino acids include arginine, proline, valine, leucine, isoleucine, glycine, serine, threonine, alanine and tryptophan. Pathways associated to glycometabolism and energy production were also obtained, such as oxidative phosphorylation, citrate cycle (TCA cycle), glycolysis/gluconeogenesis, fatty acid degradation and pyruvate metabolism, suggesting Kcr may participate in carbohydrate metabolism and energy production adjustment in *H*. *axyridis* ovarian tissues. Notably, several disease progressions related pathways were observed which included Parkinson disease, Huntington disease, Alzheimer disease, Vibrio cholerae infection and non-alcoholic fatty liver disease (NAFLD).

Although a plenty of GO terms and KEGG pathways were enriched under diapause stage, but these items possibly not specific for diapause regulation due to limitations of the qualitative analysis.

### Kcr influenced diverse biological processes and metabolism pathways in *H*. *axyridis* ovarian tissues

Previous transcriptomics and proteomics analysis have shown some genes and proteins participated in *H*. *axyridis* diapause progression [26, 37]. Here our systemic Krc analysis in the ovarian tissues at proteomics level indicated Kcr modification possibly involved in *H*. *axyridis* ovary development and diapause related processes through mediating multiple biological processes and metabolism pathways in ovarian tissues.

Carbohydrate metabolism and energy production are critical in almost every life activity of living organism as it serves the material and energy fundament role. Our study found Kcr may affect carbohydrate metabolism and energy production processes in *H*. *axyridis* ovarian tissues as some carbohydrate metabolism and energy production related pathways such as glycolysis, TCA cycle and oxidative phosphorylation were significantly enriched (Fig 4B).

**Fig 5:**
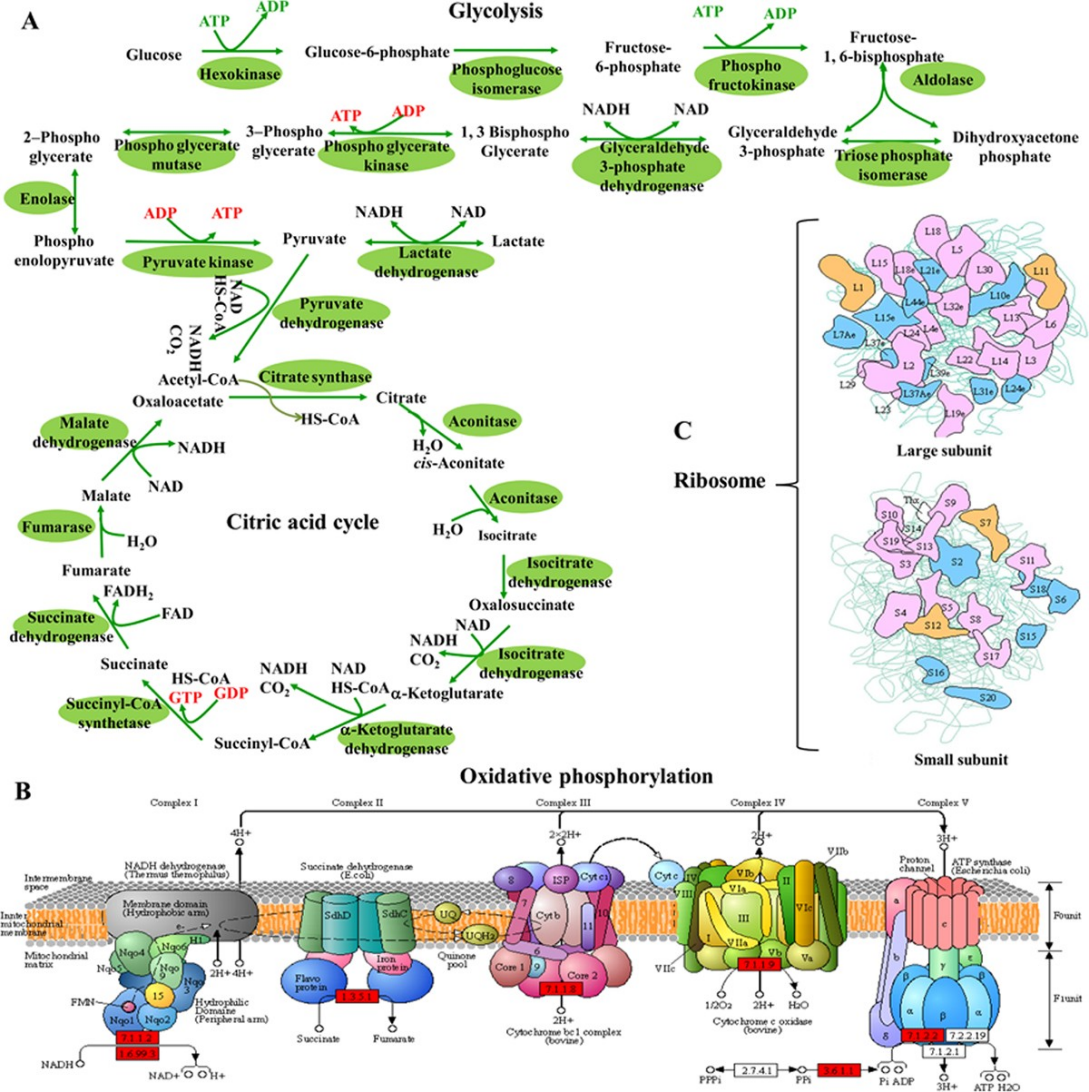
Representative significantly enriched KEGG pathways. (A) Glycolysis and Citrate cycle, the crotonylated enzymes are indicated with green background. (B) Oxidative phosphorylation, the crotonylated subunits, components and domains are labeled with red frame. (C) Ribosome, the crotonylated subunits and components are indicated with patches of colour. These figures were prepared referring KEGG pathway map images (https://www.kegg.jp/kegg/kegg3a.html; map00010, map00020, map00190 and map03010) with permission. The details of the crotonylated enzymes, functional protein subunits/components/domains refer to S2 Table.

As represented in Fig 5A, all the key enzymes involved in glycolysis and TCA cycle including hexokinase, phosphoglycerate mutase, glyceraldehyde-3-phosphate dehydrogenase, pyruvate kinase, pyruvate dehydrogenase, citrate synthase, isocitrate dehydrogenase, succinate dehydrogenase and malate dehydrogenase were Kcr modified on some subunits, domains or isoforms. The majority of these enzymes were Kcr modified with multiple lysine sites, and some even exhibited more than 10 Kcr sites, such as glyceraldehyde-3-phosphate dehydrogenase (S2 Table). Kcr modified glycolysis and TCA cycle related proteins have also been reported in Nicotiana tabacum, Carica papaya L., *Toxoplasma gondii* parasites, mouse and *Streptomyces roseosporus*, indicating Kcr modification is a conservative carbohydrate metabolism regulation pattern [18, 21, 34, 38, 39]. Apart from glycolysis and TCA cycle related Kcr proteins, some Kcr were located on the subunits or components of the five key complexes (complex I-V) of oxidative phosphorylation and electron transport chain which consist of NADH dehydrogenase (I), succinate dehydrogenase (II), cytochrome bc1 complex (III), cytochrome c oxidase (IV) and ATP synthase (V) (Fig 5B). Previous studies in rice and *T*. *gondii* ME49 also reported Kcr on oxidative phosphorylation complexes [27, 34]. The presented study expanded the study of Kcr regulated oxidative phosphorylation in insect species.

The crotonyl group on the subunits/components/domains/ of these carbohydrate and energy metabolism related enzymes and function complexes perhaps affected the gathering or assembly of these enzymes/complexes and mediated the interactions among the subunits/components/domains through its steric effect and rigidity, planar configuration [40], and finally resulted in the alteration of overall enzyme activity and efficiency. However, further biochemical experiments are necessary to validate this assumption.

Gene translation and protein biosynthesis is of vital importance to maintain the survival of cells. Previous studies have shown ribosome activities and protein metabolism were the preferred potential targets of Kcr regulated and controlled cellular events in diverse species and tissues [11, 13, 14, 21, 27, 34]. In the present study, the markedly enriched translation and ribosome related GO terms (Fig 4A) and dramatically enriched ribosome pathway and various amino acid metabolism related pathways (Fig 4B) suggest translation and protein biosynthesis processes may also be influenced by Kcr modification in the ovary tissues of *H*. *axyridis*. As shown in Fig 5C, Kcr modification was detected in diverse domains and components of both the large subunit and the small unit of ribosome. A total of 302 Kcr sites in 69 ribosome proteins were detected (S2 Table). We infer these Kcr site on ribosome protein components probably interfered the interactions among these subunits and domains, as well as the interactions between ribosome and mRNA or translation factors. Then the ovary development was arrested and the beetle entered diapause stage. Further studies are needed to verify this deduction.

Apart from the directly interplay to ribosome activities and translation events, substrate level regulation, namely the regulation of amino acid metabolism, may be another mechanism of Kcr influenced translation and protein biosynthesis in *H*. *axyridis* as a variety of amino acid metabolism related pathways were observed (Fig 4B). Kcr affected amino acid biosynthesis have been reported in diverse plant species [18, 19, 21].

In the KEGG pathway enrichment analysis, we found diverse disease progression related pathways were dramatically enriched, especially neurodegenerative disease pathways (Fig 4B), which was a noticeable phenomenon. The detailed disease related pathways included Parkinson disease, Huntington disease, Alzheimer disease, Vibrio cholerae infection, Non-alcoholic fatty liver disease (NAFLD). Previous studies have illustrated Kcr modification involved in diverse disease related processes, such as depressive disorder, various cancer progression, HIV latency and acute kidney injury [15, 41, 42]. Moreover, a transcriptome analysis in the ovaries of adult *H*. *axyridis* (Pallas) also enriched a disease related KEGG pathway, endocrine and metabolic disease [43]. It could be speculated that Kcr mediated *H*. *axyridis* diapause may share some common mechanism with Kcr modulated disease progression. This phenomenon may be reasonable as diapause is an approach which insect coping adverse stresses [24]; disease could be regarded as an adverse stress to some extent. On the other hand, reproductive stagnation under adverse environmental conditions could be considered as “insect get sickness” in some sense; nevertheless scholars don’t mention this statement in general.

In addition, a previous RNA-sequence study in the testis and male accessory gland of *H*. *axyridis* have revealed a variety of disease related pathways, such as neurodegenerative diseases, cardiovascular diseases, infectious diseases, cancers and immune diseases [44]. In our study, the dramatically enriched disease related study imply the development and diapause regulation of female reproductive tissues in *H*. *axyridis* may also be influenced by some disease progression related pathways, of which Kcr modification were involved. We infer the activities of nervous cells in the ovarian tissue may also participate in Kcr regulated ovary development and diapause. However, further studies are needed to evidence this hypothesis.

## Conclusion

In the present study, the global Kcr in the ovarian tissues of *H*. *axyridis* was performed. To our knowledge, this is the first lysine crotonylome analysis in the insect species, as well as in the genital system of female. Our study expanded the scope of Kcr in living organism. A total of 3084 Kcr site in 920 proteins were repeatedly identified in *H*. *axyridis* ovarian tissue. These Kcr modified proteins showed multiple subcellular location distribution and involved in diverse biological processes and metabolism pathways. Cytoplasm was the top subcellular compartment where Kcr proteins were localized. Carbohydrate and energy metabolism related processes such as TCA cycle, glycolysis and oxidative phosphorylation appeared be affected by Kcr modification in *H*. *axyridis* ovarian tissue. In addition, translation and proteins biosynthesis regulation is another approach of Kcr involved ovary development adjustment in *H*. *axyridis* and the underlying mechanism perhaps associated with Kcr affected ribosome activities and amino acid metabolism. Moreover, Kcr mediated H. axyridis development and diapause may share some common mechanism with Kcr modulated disease progression. Our work may serve as a useful reference for the functional demonstration of Kcr in female reproductive science and in insect biology.

## Supporting information

S1 Fig Western blot detection for Kcr proteins in the ovarian tissue of diapause *H*. *axyridis*. Two replicates were performed.
(PDF)

S2 Fig Morphological observation for the internal reproductive organs of diapause and reproductive *H*. *axyridis*. (A) Ovary of diapause *H*. *axyridis*. (B) Ovary of reproductive *H*. *axyridis*.
(PDF)

S1 Table The list of all the identified crotonylated sites and proteins in the ovarian tissue of *H*. *axyridis* at diapause stage within three replicates. (DOCX)

S2 Table The detailed information of the significantly enriched KEGG pathways. (DOCX)
