# Supplementary figures and images for: Qualitative lysine crotonylome analysis in the ovarian tissue of Harmonia axyridis (Pallas)
Source: PLoS One. 2021 Oct 18;16(10):e0258371. doi: 10.1371/journal.pone.0258371 (PMC8523065; doi:10.1371/journal.pone.0258371)

**A**

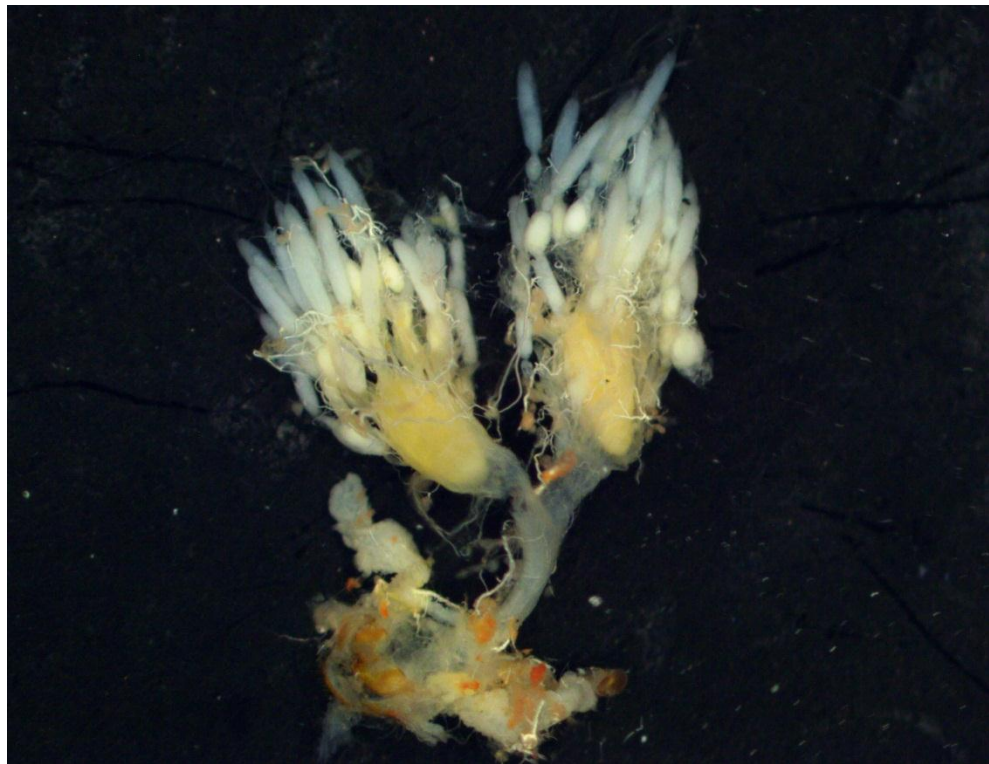

**B**

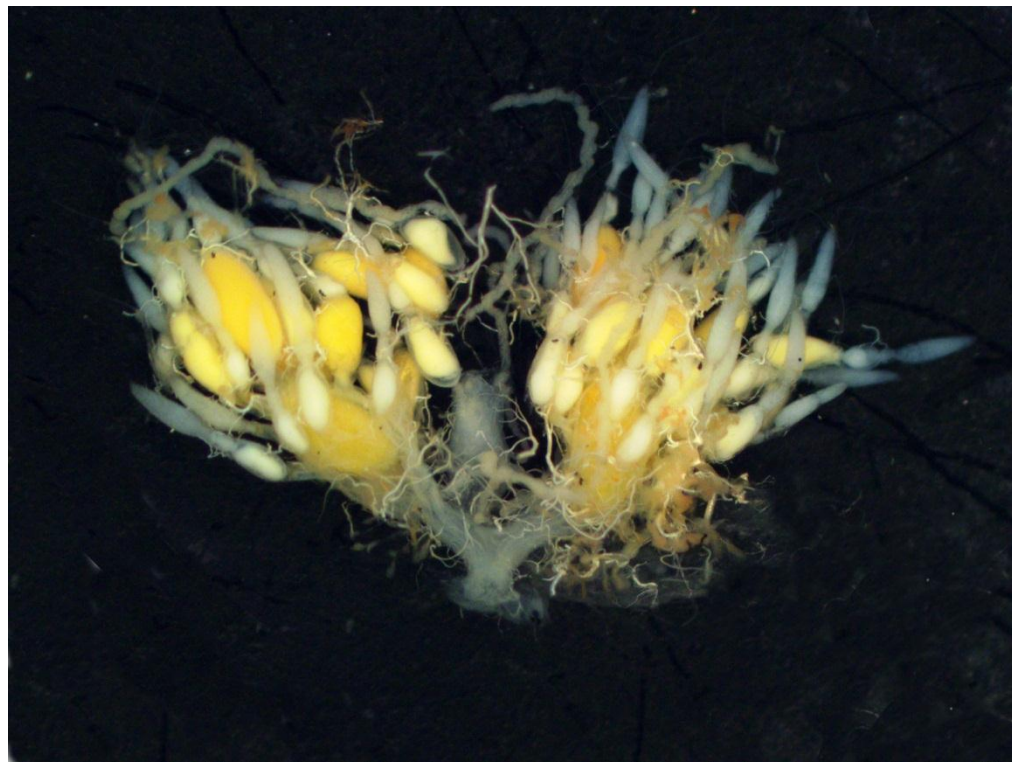

Supplement: S1 Fig — Two replicates were performed. (PDF) [file pone.0258371.s001.pdf]

Rep-1 Rep-2

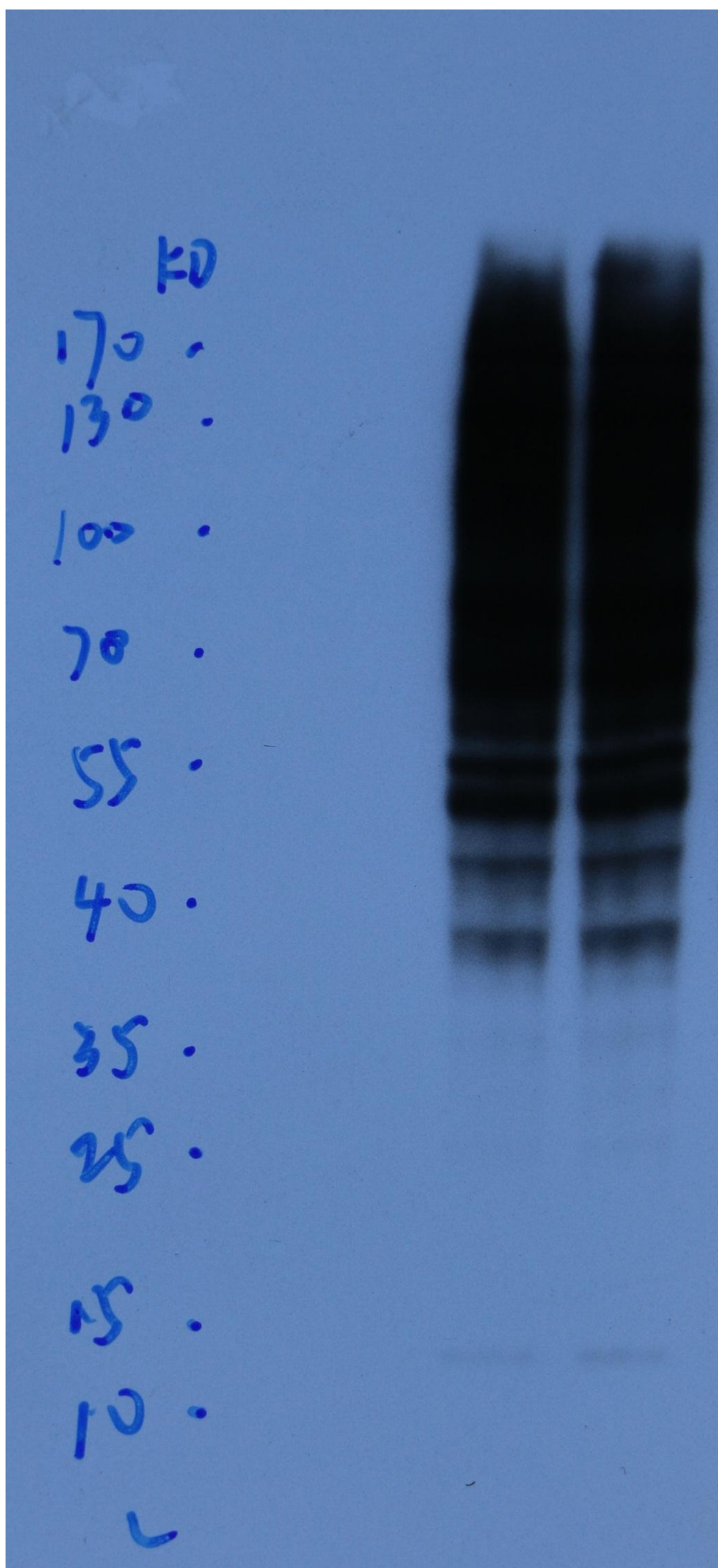

Supplement: S2 Fig — (A) Ovary of diapause H. axyridis. (B) Ovary of reproductive H. axyridis. (PDF) [file pone.0258371.s002.pdf]
