# Supplementary material for: Qualitative lysine crotonylome analysis in the ovarian tissue of Harmonia axyridis (Pallas)
Source: PLoS One. 2021 Oct 18;16(10):e0258371. doi: 10.1371/journal.pone.0258371 (PMC8523065; doi:10.1371/journal.pone.0258371)
Supplement: S1 Table — (DOCX) [file pone.0258371.s003.docx]

**Table S1** The list of all the identified lysine crotonylated (Kcr) sites and proteins in the ovarian tissue of H. axyridis at diapause stage within three replicates.

| **Protein accession** | **Protein description** | **Kac sites** | **Times of identificatin in replications** |
| --- | --- | --- | --- |
| Gene.10 | PREDICTED: flotillin-1 isoform X2 [Aethina tumida] | 294 | 3 |
| Gene.10 | PREDICTED: flotillin-1 isoform X2 [Aethina tumida] | 349 | 3 |
| Gene.10 | PREDICTED: flotillin-1 isoform X2 [Aethina tumida] | 379 | 3 |
| Gene.10 | PREDICTED: flotillin-1 isoform X2 [Aethina tumida] | 299 | 3 |
| Gene.1012 | inositol monophosphatase 1 isoform X2 [Leptinotarsa decemlineata] | 145 | 3 |
| Gene.1012 | inositol monophosphatase 1 isoform X2 [Leptinotarsa decemlineata] | 138 | 3 |
| Gene.1024 | annexin B9 isoform X2 [Anoplophora glabripennis] | 74 | 3 |
| Gene.1024 | annexin B9 isoform X2 [Anoplophora glabripennis] | 291 | 3 |
| Gene.1024 | annexin B9 isoform X2 [Anoplophora glabripennis] | 263 | 3 |
| Gene.1024 | annexin B9 isoform X2 [Anoplophora glabripennis] | 108 | 3 |
| Gene.1024 | annexin B9 isoform X2 [Anoplophora glabripennis] | 184 | 3 |
| Gene.1024 | annexin B9 isoform X2 [Anoplophora glabripennis] | 353 | 3 |
| Gene.1024 | annexin B9 isoform X2 [Anoplophora glabripennis] | 103 | 3 |
| Gene.1024 | annexin B9 isoform X2 [Anoplophora glabripennis] | 335 | 3 |
| Gene.1024 | annexin B9 isoform X2 [Anoplophora glabripennis] | 187 | 3 |
| Gene.1024 | annexin B9 isoform X2 [Anoplophora glabripennis] | 311 | 3 |
| Gene.1024 | annexin B9 isoform X2 [Anoplophora glabripennis] | 195 | 3 |
| Gene.1038 | Uncharacterized protein | 240 | 3 |
| Gene.1038 | Uncharacterized protein | 120 | 3 |
| Gene.1048 | FK506-binding protein 2 [Onthophagus taurus] | 46 | 3 |
| Gene.1048 | FK506-binding protein 2 [Onthophagus taurus] | 175 | 3 |
| Gene.1050 | PREDICTED: 2-hydroxyacylsphingosine 1-beta-galactosyltransferase-like [Tribolium castaneum] | 121 | 3 |
| Gene.1053 | 2-acylglycerol O-acyltransferase 1-like [Onthophagus taurus] | 108 | 3 |
| Gene.1059 | PREDICTED: UBX domain-containing protein 4 [Tribolium castaneum] | 347 | 3 |
| Gene.1059 | PREDICTED: UBX domain-containing protein 4 [Tribolium castaneum] | 121 | 3 |
| Gene.106 | Uncharacterized protein | 166 | 3 |
| Gene.1067 | Uncharacterized protein | 212 | 3 |
| Gene.1067 | Uncharacterized protein | 187 | 3 |
| Gene.1071 | PREDICTED: delta-1-pyrroline-5-carboxylate dehydrogenase, mitochondrial [Tribolium castaneum] | 104 | 3 |
| Gene.1071 | PREDICTED: delta-1-pyrroline-5-carboxylate dehydrogenase, mitochondrial [Tribolium castaneum] | 109 | 3 |
| Gene.1071 | PREDICTED: delta-1-pyrroline-5-carboxylate dehydrogenase, mitochondrial [Tribolium castaneum] | 98 | 3 |
| Gene.1071 | PREDICTED: delta-1-pyrroline-5-carboxylate dehydrogenase, mitochondrial [Tribolium castaneum] | 370 | 3 |
| Gene.1071 | PREDICTED: delta-1-pyrroline-5-carboxylate dehydrogenase, mitochondrial [Tribolium castaneum] | 183 | 3 |
| Gene.1071 | PREDICTED: delta-1-pyrroline-5-carboxylate dehydrogenase, mitochondrial [Tribolium castaneum] | 76 | 3 |
| Gene.1071 | PREDICTED: delta-1-pyrroline-5-carboxylate dehydrogenase, mitochondrial [Tribolium castaneum] | 47 | 3 |
| Gene.1071 | PREDICTED: delta-1-pyrroline-5-carboxylate dehydrogenase, mitochondrial [Tribolium castaneum] | 79 | 3 |
| Gene.1073 | PREDICTED: succinate dehydrogenase [ubiquinone] flavoprotein subunit, mitochondrial isoform X1 [Tribolium castaneum] | 88 | 3 |
| Gene.1073 | PREDICTED: succinate dehydrogenase [ubiquinone] flavoprotein subunit, mitochondrial isoform X1 [Tribolium castaneum] | 546 | 3 |
| Gene.1073 | PREDICTED: succinate dehydrogenase [ubiquinone] flavoprotein subunit, mitochondrial isoform X1 [Tribolium castaneum] | 529 | 3 |
| Gene.1073 | PREDICTED: succinate dehydrogenase [ubiquinone] flavoprotein subunit, mitochondrial isoform X1 [Tribolium castaneum] | 543 | 3 |
| Gene.1073 | PREDICTED: succinate dehydrogenase [ubiquinone] flavoprotein subunit, mitochondrial isoform X1 [Tribolium castaneum] | 632 | 3 |
| Gene.1073 | PREDICTED: succinate dehydrogenase [ubiquinone] flavoprotein subunit, mitochondrial isoform X1 [Tribolium castaneum] | 175 | 3 |
| Gene.1073 | PREDICTED: succinate dehydrogenase [ubiquinone] flavoprotein subunit, mitochondrial isoform X1 [Tribolium castaneum] | 331 | 3 |
| Gene.1088 | ATP-dependent RNA helicase belle, partial [Asbolus verrucosus] | 163 | 3 |
| Gene.110 | CH and/or Calponin domain containing protein [Asbolus verrucosus] | 124 | 3 |
| Gene.110 | CH and/or Calponin domain containing protein [Asbolus verrucosus] | 74 | 3 |
| Gene.110 | CH and/or Calponin domain containing protein [Asbolus verrucosus] | 142 | 3 |
| Gene.110 | CH and/or Calponin domain containing protein [Asbolus verrucosus] | 65 | 3 |
| Gene.1126 | PREDICTED: puromycin-sensitive aminopeptidase [Nicrophorus vespilloides] | 50 | 3 |
| Gene.1126 | PREDICTED: puromycin-sensitive aminopeptidase [Nicrophorus vespilloides] | 632 | 3 |
| Gene.1126 | PREDICTED: puromycin-sensitive aminopeptidase [Nicrophorus vespilloides] | 341 | 3 |
| Gene.1126 | PREDICTED: puromycin-sensitive aminopeptidase [Nicrophorus vespilloides] | 567 | 3 |
| Gene.1126 | PREDICTED: puromycin-sensitive aminopeptidase [Nicrophorus vespilloides] | 556 | 3 |
| Gene.1126 | PREDICTED: puromycin-sensitive aminopeptidase [Nicrophorus vespilloides] | 529 | 3 |
| Gene.1126 | PREDICTED: puromycin-sensitive aminopeptidase [Nicrophorus vespilloides] | 678 | 3 |
| Gene.1130 | uncharacterized protein BDFB_004851, partial [Asbolus verrucosus] | 49 | 3 |
| Gene.1159 | hypothetical protein DAPPUDRAFT_58831 [Daphnia pulex] | 94 | 3 |
| Gene.1160 | ribosomal protein S19e [Hister sp. APV-2005] | 38 | 3 |
| Gene.1160 | ribosomal protein S19e [Hister sp. APV-2005] | 97 | 3 |
| Gene.1160 | ribosomal protein S19e [Hister sp. APV-2005] | 7 | 3 |
| Gene.1160 | ribosomal protein S19e [Hister sp. APV-2005] | 139 | 3 |
| Gene.1160 | ribosomal protein S19e [Hister sp. APV-2005] | 43 | 3 |
| Gene.1160 | ribosomal protein S19e [Hister sp. APV-2005] | 23 | 3 |
| Gene.1161 | PREDICTED: UTP--glucose-1-phosphate uridylyltransferase isoform X1 [Tribolium castaneum] | 74 | 3 |
| Gene.1161 | PREDICTED: UTP--glucose-1-phosphate uridylyltransferase isoform X1 [Tribolium castaneum] | 71 | 3 |
| Gene.1161 | PREDICTED: UTP--glucose-1-phosphate uridylyltransferase isoform X1 [Tribolium castaneum] | 424 | 3 |
| Gene.1161 | PREDICTED: UTP--glucose-1-phosphate uridylyltransferase isoform X1 [Tribolium castaneum] | 305 | 3 |
| Gene.1161 | PREDICTED: UTP--glucose-1-phosphate uridylyltransferase isoform X1 [Tribolium castaneum] | 294 | 3 |
| Gene.1161 | PREDICTED: UTP--glucose-1-phosphate uridylyltransferase isoform X1 [Tribolium castaneum] | 434 | 3 |
| Gene.1161 | PREDICTED: UTP--glucose-1-phosphate uridylyltransferase isoform X1 [Tribolium castaneum] | 312 | 3 |
| Gene.1161 | PREDICTED: UTP--glucose-1-phosphate uridylyltransferase isoform X1 [Tribolium castaneum] | 284 | 3 |
| Gene.1161 | PREDICTED: UTP--glucose-1-phosphate uridylyltransferase isoform X1 [Tribolium castaneum] | 19 | 3 |
| Gene.1175 | PREDICTED: 26S proteasome non-ATPase regulatory subunit 12 [Aethina tumida] | 210 | 3 |
| Gene.1175 | PREDICTED: 26S proteasome non-ATPase regulatory subunit 12 [Aethina tumida] | 121 | 3 |
| Gene.1199 | PREDICTED: protein suppressor of forked [Tribolium castaneum] | 40 | 3 |
| Gene.1212 | leukotriene A-4 hydrolase isoform X1 [Anoplophora glabripennis] | 384 | 3 |
| Gene.1212 | leukotriene A-4 hydrolase isoform X1 [Anoplophora glabripennis] | 416 | 3 |
| Gene.1218 | histone H2B-like [Zootermopsis nevadensis] | 92 | 3 |
| Gene.1218 | histone H2B-like [Zootermopsis nevadensis] | 123 | 3 |
| Gene.1218 | histone H2B-like [Zootermopsis nevadensis] | 27 | 3 |
| Gene.1218 | histone H2B-like [Zootermopsis nevadensis] | 127 | 3 |
| Gene.1218 | histone H2B-like [Zootermopsis nevadensis] | 53 | 3 |
| Gene.1218 | histone H2B-like [Zootermopsis nevadensis] | 115 | 3 |
| Gene.1218 | histone H2B-like [Zootermopsis nevadensis] | 41 | 3 |
| Gene.1218 | histone H2B-like [Zootermopsis nevadensis] | 31 | 3 |
| Gene.1219 | 46 kDa FK506-binding nuclear protein [Anoplophora glabripennis] | 343 | 3 |
| Gene.1223 | eukaryotic translation initiation factor 3 subunit M [Anoplophora glabripennis] | 67 | 3 |
| Gene.1224 | PREDICTED: multidrug resistance protein 1 [Tribolium castaneum] | 985 | 3 |
| Gene.1224 | PREDICTED: multidrug resistance protein 1 [Tribolium castaneum] | 393 | 3 |
| Gene.1224 | PREDICTED: multidrug resistance protein 1 [Tribolium castaneum] | 534 | 3 |
| Gene.1224 | PREDICTED: multidrug resistance protein 1 [Tribolium castaneum] | 576 | 3 |
| Gene.1224 | PREDICTED: multidrug resistance protein 1 [Tribolium castaneum] | 268 | 3 |
| Gene.1224 | PREDICTED: multidrug resistance protein 1 [Tribolium castaneum] | 1158 | 3 |
| Gene.123 | PREDICTED: UMP-CMP kinase 2, mitochondrial [Tribolium castaneum] | 277 | 3 |
| Gene.1244 | cytochrome P450 CYP9Z401 [Cryptolaemus montrouzieri] | 89 | 3 |
| Gene.1244 | cytochrome P450 CYP9Z401 [Cryptolaemus montrouzieri] | 141 | 3 |
| Gene.1253 | PREDICTED: sedoheptulokinase isoform X2 [Tribolium castaneum] | 112 | 3 |
| Gene.1253 | PREDICTED: sedoheptulokinase isoform X2 [Tribolium castaneum] | 356 | 3 |
| Gene.1253 | PREDICTED: sedoheptulokinase isoform X2 [Tribolium castaneum] | 73 | 3 |
| Gene.1264 | PREDICTED: protein disulfide-isomerase [Tribolium castaneum] | 365 | 3 |
| Gene.1264 | PREDICTED: protein disulfide-isomerase [Tribolium castaneum] | 67 | 3 |
| Gene.1264 | PREDICTED: protein disulfide-isomerase [Tribolium castaneum] | 207 | 3 |
| Gene.1264 | PREDICTED: protein disulfide-isomerase [Tribolium castaneum] | 443 | 3 |
| Gene.1264 | PREDICTED: protein disulfide-isomerase [Tribolium castaneum] | 70 | 3 |
| Gene.1264 | PREDICTED: protein disulfide-isomerase [Tribolium castaneum] | 307 | 3 |
| Gene.1264 | PREDICTED: protein disulfide-isomerase [Tribolium castaneum] | 80 | 3 |
| Gene.1264 | PREDICTED: protein disulfide-isomerase [Tribolium castaneum] | 435 | 3 |
| Gene.1264 | PREDICTED: protein disulfide-isomerase [Tribolium castaneum] | 269 | 3 |
| Gene.1264 | PREDICTED: protein disulfide-isomerase [Tribolium castaneum] | 212 | 3 |
| Gene.1264 | PREDICTED: protein disulfide-isomerase [Tribolium castaneum] | 349 | 3 |
| Gene.1264 | PREDICTED: protein disulfide-isomerase [Tribolium castaneum] | 351 | 3 |
| Gene.1264 | PREDICTED: protein disulfide-isomerase [Tribolium castaneum] | 378 | 3 |
| Gene.1264 | PREDICTED: protein disulfide-isomerase [Tribolium castaneum] | 325 | 3 |
| Gene.1264 | PREDICTED: protein disulfide-isomerase [Tribolium castaneum] | 105 | 3 |
| Gene.1264 | PREDICTED: protein disulfide-isomerase [Tribolium castaneum] | 473 | 3 |
| Gene.1264 | PREDICTED: protein disulfide-isomerase [Tribolium castaneum] | 75 | 3 |
| Gene.1264 | PREDICTED: protein disulfide-isomerase [Tribolium castaneum] | 451 | 3 |
| Gene.1264 | PREDICTED: protein disulfide-isomerase [Tribolium castaneum] | 137 | 3 |
| Gene.1264 | PREDICTED: protein disulfide-isomerase [Tribolium castaneum] | 408 | 3 |
| Gene.1264 | PREDICTED: protein disulfide-isomerase [Tribolium castaneum] | 447 | 3 |
| Gene.1264 | PREDICTED: protein disulfide-isomerase [Tribolium castaneum] | 414 | 3 |
| Gene.1264 | PREDICTED: protein disulfide-isomerase [Tribolium castaneum] | 340 | 3 |
| Gene.1264 | PREDICTED: protein disulfide-isomerase [Tribolium castaneum] | 423 | 3 |
| Gene.1264 | PREDICTED: protein disulfide-isomerase [Tribolium castaneum] | 140 | 3 |
| Gene.1295 | PREDICTED: lamin Dm0 isoform X1 [Aethina tumida] | 274 | 3 |
| Gene.1295 | PREDICTED: lamin Dm0 isoform X1 [Aethina tumida] | 486 | 3 |
| Gene.1295 | PREDICTED: lamin Dm0 isoform X1 [Aethina tumida] | 458 | 3 |
| Gene.1295 | PREDICTED: lamin Dm0 isoform X1 [Aethina tumida] | 455 | 3 |
| Gene.1295 | PREDICTED: lamin Dm0 isoform X1 [Aethina tumida] | 111 | 3 |
| Gene.1295 | PREDICTED: lamin Dm0 isoform X1 [Aethina tumida] | 167 | 3 |
| Gene.1295 | PREDICTED: lamin Dm0 isoform X1 [Aethina tumida] | 205 | 3 |
| Gene.1295 | PREDICTED: lamin Dm0 isoform X1 [Aethina tumida] | 448 | 3 |
| Gene.1295 | PREDICTED: lamin Dm0 isoform X1 [Aethina tumida] | 244 | 3 |
| Gene.1295 | PREDICTED: lamin Dm0 isoform X1 [Aethina tumida] | 144 | 3 |
| Gene.1295 | PREDICTED: lamin Dm0 isoform X1 [Aethina tumida] | 101 | 3 |
| Gene.1295 | PREDICTED: lamin Dm0 isoform X1 [Aethina tumida] | 329 | 3 |
| Gene.130 | PREDICTED: T-complex protein 1 subunit beta [Tribolium castaneum] | 119 | 3 |
| Gene.130 | PREDICTED: T-complex protein 1 subunit beta [Tribolium castaneum] | 271 | 3 |
| Gene.130 | PREDICTED: T-complex protein 1 subunit beta [Tribolium castaneum] | 11 | 3 |
| Gene.130 | PREDICTED: T-complex protein 1 subunit beta [Tribolium castaneum] | 499 | 3 |
| Gene.130 | PREDICTED: T-complex protein 1 subunit beta [Tribolium castaneum] | 229 | 3 |
| Gene.130 | PREDICTED: T-complex protein 1 subunit beta [Tribolium castaneum] | 262 | 3 |
| Gene.1305 | hypothetical protein AMK59_6936 [Oryctes borbonicus] | 104 | 3 |
| Gene.1305 | hypothetical protein AMK59_6936 [Oryctes borbonicus] | 162 | 3 |
| Gene.1305 | hypothetical protein AMK59_6936 [Oryctes borbonicus] | 214 | 3 |
| Gene.1329 | PREDICTED: thioredoxin-2 [Dendroctonus ponderosae] | 6 | 3 |
| Gene.1329 | PREDICTED: thioredoxin-2 [Dendroctonus ponderosae] | 8 | 3 |
| Gene.1329 | PREDICTED: thioredoxin-2 [Dendroctonus ponderosae] | 98 | 3 |
| Gene.1329 | PREDICTED: thioredoxin-2 [Dendroctonus ponderosae] | 96 | 3 |
| Gene.1329 | PREDICTED: thioredoxin-2 [Dendroctonus ponderosae] | 41 | 3 |
| Gene.1333 | soma ferritin [Asbolus verrucosus] | 74 | 3 |
| Gene.1333 | soma ferritin [Asbolus verrucosus] | 59 | 3 |
| Gene.1333 | soma ferritin [Asbolus verrucosus] | 155 | 3 |
| Gene.1333 | soma ferritin [Asbolus verrucosus] | 55 | 3 |
| Gene.1354 | PREDICTED: putative aminopeptidase W07G4.4 [Nicrophorus vespilloides] | 170 | 3 |
| Gene.1354 | PREDICTED: putative aminopeptidase W07G4.4 [Nicrophorus vespilloides] | 251 | 3 |
| Gene.1354 | PREDICTED: putative aminopeptidase W07G4.4 [Nicrophorus vespilloides] | 118 | 3 |
| Gene.1357 | Uncharacterized protein | 89 | 3 |
| Gene.1358 | Uncharacterized protein | 50 | 3 |
| Gene.1358 | Uncharacterized protein | 34 | 3 |
| Gene.1358 | Uncharacterized protein | 40 | 3 |
| Gene.1361 | PREDICTED: glycogenin-1 isoform X3 [Aethina tumida] | 702 | 3 |
| Gene.1365 | PREDICTED: maltase 2 isoform X3 [Tribolium castaneum] | 453 | 3 |
| Gene.1388 | PREDICTED: excitatory amino acid transporter 3-like [Aethina tumida] | 38 | 3 |
| Gene.1388 | PREDICTED: excitatory amino acid transporter 3-like [Aethina tumida] | 472 | 3 |
| Gene.1388 | PREDICTED: excitatory amino acid transporter 3-like [Aethina tumida] | 175 | 3 |
| Gene.1391 | PREDICTED: protein lethal(2)essential for life [Tribolium castaneum] | 78 | 3 |
| Gene.1392 | hypothetical protein WN55_01867 [Dufourea novaeangliae] | 166 | 3 |
| Gene.1392 | hypothetical protein WN55_01867 [Dufourea novaeangliae] | 117 | 3 |
| Gene.1392 | hypothetical protein WN55_01867 [Dufourea novaeangliae] | 43 | 3 |
| Gene.1392 | hypothetical protein WN55_01867 [Dufourea novaeangliae] | 249 | 3 |
| Gene.1392 | hypothetical protein WN55_01867 [Dufourea novaeangliae] | 79 | 3 |
| Gene.1392 | hypothetical protein WN55_01867 [Dufourea novaeangliae] | 20 | 3 |
| Gene.1398 | PREDICTED: multifunctional protein ADE2 [Aethina tumida] | 84 | 3 |
| Gene.1398 | PREDICTED: multifunctional protein ADE2 [Aethina tumida] | 130 | 3 |
| Gene.1398 | PREDICTED: multifunctional protein ADE2 [Aethina tumida] | 428 | 3 |
| Gene.1398 | PREDICTED: multifunctional protein ADE2 [Aethina tumida] | 258 | 3 |
| Gene.1398 | PREDICTED: multifunctional protein ADE2 [Aethina tumida] | 22 | 3 |
| Gene.1398 | PREDICTED: multifunctional protein ADE2 [Aethina tumida] | 232 | 3 |
| Gene.1398 | PREDICTED: multifunctional protein ADE2 [Aethina tumida] | 24 | 3 |
| Gene.1398 | PREDICTED: multifunctional protein ADE2 [Aethina tumida] | 422 | 3 |
| Gene.1399 | PREDICTED: GDP-L-fucose synthase-like [Aethina tumida] | 285 | 3 |
| Gene.1403 | Protein LSM14 homolog B-like Protein [Tribolium castaneum] | 424 | 3 |
| Gene.1403 | Protein LSM14 homolog B-like Protein [Tribolium castaneum] | 497 | 3 |
| Gene.1403 | Protein LSM14 homolog B-like Protein [Tribolium castaneum] | 347 | 3 |
| Gene.1403 | Protein LSM14 homolog B-like Protein [Tribolium castaneum] | 17 | 3 |
| Gene.1403 | Protein LSM14 homolog B-like Protein [Tribolium castaneum] | 417 | 3 |
| Gene.1403 | Protein LSM14 homolog B-like Protein [Tribolium castaneum] | 73 | 3 |
| Gene.1403 | Protein LSM14 homolog B-like Protein [Tribolium castaneum] | 338 | 3 |
| Gene.141 | adenosine kinase [Anoplophora glabripennis] | 251 | 3 |
| Gene.1417 | PREDICTED: dihydrolipoyllysine-residue succinyltransferase component of 2-oxoglutarate dehydrogenase complex, mitochondrial [Tribolium castaneum] | 124 | 3 |
| Gene.1428 | PREDICTED: prostaglandin E synthase 3 [Nicrophorus vespilloides] | 38 | 3 |
| Gene.143 | cytochrome c oxidase subunit IV [Tribolium castaneum] | 69 | 3 |
| Gene.143 | cytochrome c oxidase subunit IV [Tribolium castaneum] | 180 | 3 |
| Gene.143 | cytochrome c oxidase subunit IV [Tribolium castaneum] | 94 | 3 |
| Gene.143 | cytochrome c oxidase subunit IV [Tribolium castaneum] | 184 | 3 |
| Gene.1438 | cytochrome b5 [Anoplophora glabripennis] | 75 | 3 |
| Gene.1438 | cytochrome b5 [Anoplophora glabripennis] | 13 | 3 |
| Gene.1451 | PREDICTED: uncharacterized protein LOC109597373 isoform X2 [Aethina tumida] | 344 | 3 |
| Gene.1454 | PREDICTED: endocuticle structural glycoprotein SgAbd-8-like [Aethina tumida] | 72 | 3 |
| Gene.1458 | juvenile hormone epoxide hydrolase [Coccinella septempunctata] | 334 | 3 |
| Gene.1467 | imaginal disc growth factor 2 precursor [Tribolium castaneum] | 160 | 3 |
| Gene.1468 | PREDICTED: dolichyl-diphosphooligosaccharide--protein glycosyltransferase subunit STT3B [Aethina tumida] | 748 | 3 |
| Gene.1476 | zinc finger protein on ecdysone puffs, partial [Asbolus verrucosus] | 276 | 3 |
| Gene.1478 | PREDICTED: fasciclin-1 [Aethina tumida] | 208 | 3 |
| Gene.1482 | flotillin-2 [Onthophagus taurus] | 344 | 3 |
| Gene.1482 | flotillin-2 [Onthophagus taurus] | 335 | 3 |
| Gene.1482 | flotillin-2 [Onthophagus taurus] | 211 | 3 |
| Gene.1486 | Prefoldin domain containing protein [Asbolus verrucosus] | 134 | 3 |
| Gene.1489 | aspartate aminotransferase, mitochondrial [Asbolus verrucosus] | 334 | 3 |
| Gene.1489 | aspartate aminotransferase, mitochondrial [Asbolus verrucosus] | 189 | 3 |
| Gene.1489 | aspartate aminotransferase, mitochondrial [Asbolus verrucosus] | 332 | 3 |
| Gene.1489 | aspartate aminotransferase, mitochondrial [Asbolus verrucosus] | 295 | 3 |
| Gene.1489 | aspartate aminotransferase, mitochondrial [Asbolus verrucosus] | 91 | 3 |
| Gene.1489 | aspartate aminotransferase, mitochondrial [Asbolus verrucosus] | 401 | 3 |
| Gene.1489 | aspartate aminotransferase, mitochondrial [Asbolus verrucosus] | 87 | 3 |
| Gene.1489 | aspartate aminotransferase, mitochondrial [Asbolus verrucosus] | 174 | 3 |
| Gene.1489 | aspartate aminotransferase, mitochondrial [Asbolus verrucosus] | 393 | 3 |
| Gene.150 | PREDICTED: eukaryotic translation initiation factor 5A [Dendroctonus ponderosae] | 116 | 3 |
| Gene.150 | PREDICTED: eukaryotic translation initiation factor 5A [Dendroctonus ponderosae] | 134 | 3 |
| Gene.1500 | dolichyl-diphosphooligosaccharide--protein glycosyltransferase 48 kDa subunit [Anoplophora glabripennis] | 46 | 3 |
| Gene.1501 | cathepsin B precursor [Leptinotarsa decemlineata] | 158 | 3 |
| Gene.1501 | cathepsin B precursor [Leptinotarsa decemlineata] | 165 | 3 |
| Gene.1501 | cathepsin B precursor [Leptinotarsa decemlineata] | 37 | 3 |
| Gene.1502 | C1 family cathepsin B32 [Tenebrio molitor] | 167 | 3 |
| Gene.1513 | reticulon-1-A isoform X2 [Leptinotarsa decemlineata] | 119 | 3 |
| Gene.1513 | reticulon-1-A isoform X2 [Leptinotarsa decemlineata] | 225 | 3 |
| Gene.1513 | reticulon-1-A isoform X2 [Leptinotarsa decemlineata] | 57 | 3 |
| Gene.1513 | reticulon-1-A isoform X2 [Leptinotarsa decemlineata] | 232 | 3 |
| Gene.1513 | reticulon-1-A isoform X2 [Leptinotarsa decemlineata] | 111 | 3 |
| Gene.1524 | V-type proton ATPase subunit B [Galleria mellonella] | 445 | 3 |
| Gene.1528 | PREDICTED: protein SCO1 homolog, mitochondrial [Aethina tumida] | 165 | 3 |
| Gene.1530 | PREDICTED: farnesyl pyrophosphate synthase-like [Atta cephalotes] | 354 | 3 |
| Gene.1530 | PREDICTED: farnesyl pyrophosphate synthase-like [Atta cephalotes] | 323 | 3 |
| Gene.1530 | PREDICTED: farnesyl pyrophosphate synthase-like [Atta cephalotes] | 260 | 3 |
| Gene.1530 | PREDICTED: farnesyl pyrophosphate synthase-like [Atta cephalotes] | 187 | 3 |
| Gene.1532 | PREDICTED: glyoxalase domain-containing protein 4 [Tribolium castaneum] | 201 | 3 |
| Gene.1532 | PREDICTED: glyoxalase domain-containing protein 4 [Tribolium castaneum] | 256 | 3 |
| Gene.1532 | PREDICTED: glyoxalase domain-containing protein 4 [Tribolium castaneum] | 14 | 3 |
| Gene.1533 | PREDICTED: hexokinase type 2 isoform X2 [Tribolium castaneum] | 280 | 3 |
| Gene.1533 | PREDICTED: hexokinase type 2 isoform X2 [Tribolium castaneum] | 73 | 3 |
| Gene.1534 | PREDICTED: T-complex protein 1 subunit gamma [Tribolium castaneum] | 173 | 3 |
| Gene.1534 | PREDICTED: T-complex protein 1 subunit gamma [Tribolium castaneum] | 369 | 3 |
| Gene.1534 | PREDICTED: T-complex protein 1 subunit gamma [Tribolium castaneum] | 179 | 3 |
| Gene.1534 | PREDICTED: T-complex protein 1 subunit gamma [Tribolium castaneum] | 292 | 3 |
| Gene.1534 | PREDICTED: T-complex protein 1 subunit gamma [Tribolium castaneum] | 441 | 3 |
| Gene.1539 | TROVE domain containing protein [Asbolus verrucosus] | 209 | 3 |
| Gene.1539 | TROVE domain containing protein [Asbolus verrucosus] | 357 | 3 |
| Gene.154 | PREDICTED: 26S protease regulatory subunit 7 [Tribolium castaneum] | 112 | 3 |
| Gene.154 | PREDICTED: 26S protease regulatory subunit 7 [Tribolium castaneum] | 17 | 3 |
| Gene.1542 | PREDICTED: ATP synthase subunit alpha, mitochondrial [Tribolium castaneum] | 237 | 3 |
| Gene.1542 | PREDICTED: ATP synthase subunit alpha, mitochondrial [Tribolium castaneum] | 130 | 3 |
| Gene.1542 | PREDICTED: ATP synthase subunit alpha, mitochondrial [Tribolium castaneum] | 63 | 3 |
| Gene.1542 | PREDICTED: ATP synthase subunit alpha, mitochondrial [Tribolium castaneum] | 71 | 3 |
| Gene.1542 | PREDICTED: ATP synthase subunit alpha, mitochondrial [Tribolium castaneum] | 165 | 3 |
| Gene.1542 | PREDICTED: ATP synthase subunit alpha, mitochondrial [Tribolium castaneum] | 314 | 3 |
| Gene.1542 | PREDICTED: ATP synthase subunit alpha, mitochondrial [Tribolium castaneum] | 537 | 3 |
| Gene.1542 | PREDICTED: ATP synthase subunit alpha, mitochondrial [Tribolium castaneum] | 514 | 3 |
| Gene.1542 | PREDICTED: ATP synthase subunit alpha, mitochondrial [Tribolium castaneum] | 526 | 3 |
| Gene.1542 | PREDICTED: ATP synthase subunit alpha, mitochondrial [Tribolium castaneum] | 422 | 3 |
| Gene.1542 | PREDICTED: ATP synthase subunit alpha, mitochondrial [Tribolium castaneum] | 159 | 3 |
| Gene.1542 | PREDICTED: ATP synthase subunit alpha, mitochondrial [Tribolium castaneum] | 228 | 3 |
| Gene.1542 | PREDICTED: ATP synthase subunit alpha, mitochondrial [Tribolium castaneum] | 303 | 3 |
| Gene.1542 | PREDICTED: ATP synthase subunit alpha, mitochondrial [Tribolium castaneum] | 518 | 3 |
| Gene.1542 | PREDICTED: ATP synthase subunit alpha, mitochondrial [Tribolium castaneum] | 425 | 3 |
| Gene.1542 | PREDICTED: ATP synthase subunit alpha, mitochondrial [Tribolium castaneum] | 501 | 3 |
| Gene.1542 | PREDICTED: ATP synthase subunit alpha, mitochondrial [Tribolium castaneum] | 529 | 3 |
| Gene.1542 | PREDICTED: ATP synthase subunit alpha, mitochondrial [Tribolium castaneum] | 432 | 3 |
| Gene.1542 | PREDICTED: ATP synthase subunit alpha, mitochondrial [Tribolium castaneum] | 496 | 3 |
| Gene.1542 | PREDICTED: ATP synthase subunit alpha, mitochondrial [Tribolium castaneum] | 507 | 3 |
| Gene.1561 | PREDICTED: uncharacterized protein LOC661483 [Tribolium castaneum] | 237 | 3 |
| Gene.1564 | chickadee [Tribolium castaneum] | 40 | 3 |
| Gene.1570 | GRASP55 65, PDZ 2, and/or DUF605 domain containing protein [Asbolus verrucosus] | 50 | 3 |
| Gene.1579 | putative gut cathepsin D-like aspartic protease [Callosobruchus maculatus] | 118 | 3 |
| Gene.1579 | putative gut cathepsin D-like aspartic protease [Callosobruchus maculatus] | 310 | 3 |
| Gene.1580 | PREDICTED: aminomethyltransferase, mitochondrial [Tribolium castaneum] | 170 | 3 |
| Gene.1580 | PREDICTED: aminomethyltransferase, mitochondrial [Tribolium castaneum] | 338 | 3 |
| Gene.1584 | 26S proteasome non-ATPase regulatory subunit 6 [Leptinotarsa decemlineata] | 2 | 3 |
| Gene.1599 | glycerol-3-phosphate dehydrogenase [NAD(+)], cytoplasmic isoform X1 [Anoplophora glabripennis] | 34 | 3 |
| Gene.1599 | glycerol-3-phosphate dehydrogenase [NAD(+)], cytoplasmic isoform X1 [Anoplophora glabripennis] | 321 | 3 |
| Gene.1599 | glycerol-3-phosphate dehydrogenase [NAD(+)], cytoplasmic isoform X1 [Anoplophora glabripennis] | 65 | 3 |
| Gene.1613 | Dper\GL12416-PA-like protein [Anopheles sinensis] | 163 | 3 |
| Gene.1613 | Dper\GL12416-PA-like protein [Anopheles sinensis] | 401 | 3 |
| Gene.1613 | Dper\GL12416-PA-like protein [Anopheles sinensis] | 60 | 3 |
| Gene.1613 | Dper\GL12416-PA-like protein [Anopheles sinensis] | 370 | 3 |
| Gene.1613 | Dper\GL12416-PA-like protein [Anopheles sinensis] | 326 | 3 |
| Gene.1613 | Dper\GL12416-PA-like protein [Anopheles sinensis] | 96 | 3 |
| Gene.1613 | Dper\GL12416-PA-like protein [Anopheles sinensis] | 394 | 3 |
| Gene.1613 | Dper\GL12416-PA-like protein [Anopheles sinensis] | 336 | 3 |
| Gene.162 | Uncharacterized protein | 127 | 3 |
| Gene.162 | Uncharacterized protein | 142 | 3 |
| Gene.162 | Uncharacterized protein | 169 | 3 |
| Gene.1633 | PREDICTED: fructose-bisphosphate aldolase [Tribolium castaneum] | 42 | 3 |
| Gene.1633 | PREDICTED: fructose-bisphosphate aldolase [Tribolium castaneum] | 147 | 3 |
| Gene.1633 | PREDICTED: fructose-bisphosphate aldolase [Tribolium castaneum] | 28 | 3 |
| Gene.164 | PREDICTED: guanine nucleotide-binding protein subunit beta-1 [Tribolium castaneum] | 78 | 3 |
| Gene.1645 | PREDICTED: facilitated trehalose transporter Tret1-like [Aethina tumida] | 30 | 3 |
| Gene.1653 | PREDICTED: hydroxyacid oxidase 1 [Tribolium castaneum] | 193 | 3 |
| Gene.166 | held out wings [Tribolium castaneum] | 182 | 3 |
| Gene.1660 | hypothetical protein TcasGA2_TC031159 [Tribolium castaneum] | 68 | 3 |
| Gene.1660 | hypothetical protein TcasGA2_TC031159 [Tribolium castaneum] | 114 | 3 |
| Gene.1667 | PREDICTED: tubulin beta-1 chain [Tribolium castaneum] | 324 | 3 |
| Gene.1667 | PREDICTED: tubulin beta-1 chain [Tribolium castaneum] | 58 | 3 |
| Gene.1679 | Uncharacterized protein | 274 | 3 |
| Gene.1679 | Uncharacterized protein | 238 | 3 |
| Gene.1679 | Uncharacterized protein | 130 | 3 |
| Gene.1679 | Uncharacterized protein | 204 | 3 |
| Gene.1679 | Uncharacterized protein | 120 | 3 |
| Gene.1679 | Uncharacterized protein | 243 | 3 |
| Gene.1679 | Uncharacterized protein | 209 | 3 |
| Gene.1679 | Uncharacterized protein | 215 | 3 |
| Gene.1679 | Uncharacterized protein | 201 | 3 |
| Gene.1679 | Uncharacterized protein | 93 | 3 |
| Gene.1679 | Uncharacterized protein | 219 | 3 |
| Gene.1679 | Uncharacterized protein | 125 | 3 |
| Gene.1679 | Uncharacterized protein | 229 | 3 |
| Gene.169 | PREDICTED: 4-hydroxybutyrate coenzyme A transferase [Tribolium castaneum] | 189 | 3 |
| Gene.169 | PREDICTED: 4-hydroxybutyrate coenzyme A transferase [Tribolium castaneum] | 397 | 3 |
| Gene.169 | PREDICTED: 4-hydroxybutyrate coenzyme A transferase [Tribolium castaneum] | 181 | 3 |
| Gene.170 | RNA-binding protein lark [Asbolus verrucosus] | 35 | 3 |
| Gene.170 | RNA-binding protein lark [Asbolus verrucosus] | 32 | 3 |
| Gene.170 | RNA-binding protein lark [Asbolus verrucosus] | 101 | 3 |
| Gene.170 | RNA-binding protein lark [Asbolus verrucosus] | 43 | 3 |
| Gene.1711 | Uncharacterized protein | 116 | 3 |
| Gene.1711 | Uncharacterized protein | 102 | 3 |
| Gene.1711 | Uncharacterized protein | 129 | 3 |
| Gene.172 | beta-actin, partial [Cotesia chilonis] | 216 | 3 |
| Gene.172 | beta-actin, partial [Cotesia chilonis] | 114 | 3 |
| Gene.172 | beta-actin, partial [Cotesia chilonis] | 51 | 3 |
| Gene.172 | beta-actin, partial [Cotesia chilonis] | 62 | 3 |
| Gene.1725 | neprilysin-2-like [Leptinotarsa decemlineata] | 424 | 3 |
| Gene.1725 | neprilysin-2-like [Leptinotarsa decemlineata] | 449 | 3 |
| Gene.1725 | neprilysin-2-like [Leptinotarsa decemlineata] | 258 | 3 |
| Gene.1725 | neprilysin-2-like [Leptinotarsa decemlineata] | 358 | 3 |
| Gene.1745 | PREDICTED: probable medium-chain specific acyl-CoA dehydrogenase, mitochondrial [Tribolium castaneum] | 333 | 3 |
| Gene.1745 | PREDICTED: probable medium-chain specific acyl-CoA dehydrogenase, mitochondrial [Tribolium castaneum] | 63 | 3 |
| Gene.1745 | PREDICTED: probable medium-chain specific acyl-CoA dehydrogenase, mitochondrial [Tribolium castaneum] | 206 | 3 |
| Gene.1745 | PREDICTED: probable medium-chain specific acyl-CoA dehydrogenase, mitochondrial [Tribolium castaneum] | 342 | 3 |
| Gene.1745 | PREDICTED: probable medium-chain specific acyl-CoA dehydrogenase, mitochondrial [Tribolium castaneum] | 253 | 3 |
| Gene.1745 | PREDICTED: probable medium-chain specific acyl-CoA dehydrogenase, mitochondrial [Tribolium castaneum] | 273 | 3 |
| Gene.1745 | PREDICTED: probable medium-chain specific acyl-CoA dehydrogenase, mitochondrial [Tribolium castaneum] | 295 | 3 |
| Gene.1745 | PREDICTED: probable medium-chain specific acyl-CoA dehydrogenase, mitochondrial [Tribolium castaneum] | 211 | 3 |
| Gene.1745 | PREDICTED: probable medium-chain specific acyl-CoA dehydrogenase, mitochondrial [Tribolium castaneum] | 265 | 3 |
| Gene.1745 | PREDICTED: probable medium-chain specific acyl-CoA dehydrogenase, mitochondrial [Tribolium castaneum] | 73 | 3 |
| Gene.175 | PREDICTED: probable citrate synthase 2, mitochondrial [Tribolium castaneum] | 78 | 3 |
| Gene.175 | PREDICTED: probable citrate synthase 2, mitochondrial [Tribolium castaneum] | 193 | 3 |
| Gene.175 | PREDICTED: probable citrate synthase 2, mitochondrial [Tribolium castaneum] | 331 | 3 |
| Gene.175 | PREDICTED: probable citrate synthase 2, mitochondrial [Tribolium castaneum] | 324 | 3 |
| Gene.175 | PREDICTED: probable citrate synthase 2, mitochondrial [Tribolium castaneum] | 397 | 3 |
| Gene.175 | PREDICTED: probable citrate synthase 2, mitochondrial [Tribolium castaneum] | 461 | 3 |
| Gene.175 | PREDICTED: probable citrate synthase 2, mitochondrial [Tribolium castaneum] | 141 | 3 |
| Gene.1768 | PREDICTED: translation elongation factor 2 [Aethina tumida] | 238 | 3 |
| Gene.1768 | PREDICTED: translation elongation factor 2 [Aethina tumida] | 32 | 3 |
| Gene.1768 | PREDICTED: translation elongation factor 2 [Aethina tumida] | 50 | 3 |
| Gene.1768 | PREDICTED: translation elongation factor 2 [Aethina tumida] | 412 | 3 |
| Gene.1768 | PREDICTED: translation elongation factor 2 [Aethina tumida] | 259 | 3 |
| Gene.1768 | PREDICTED: translation elongation factor 2 [Aethina tumida] | 245 | 3 |
| Gene.1768 | PREDICTED: translation elongation factor 2 [Aethina tumida] | 484 | 3 |
| Gene.1768 | PREDICTED: translation elongation factor 2 [Aethina tumida] | 475 | 3 |
| Gene.1768 | PREDICTED: translation elongation factor 2 [Aethina tumida] | 425 | 3 |
| Gene.1768 | PREDICTED: translation elongation factor 2 [Aethina tumida] | 319 | 3 |
| Gene.1768 | PREDICTED: translation elongation factor 2 [Aethina tumida] | 431 | 3 |
| Gene.1768 | PREDICTED: translation elongation factor 2 [Aethina tumida] | 481 | 3 |
| Gene.1768 | PREDICTED: translation elongation factor 2 [Aethina tumida] | 624 | 3 |
| Gene.1768 | PREDICTED: translation elongation factor 2 [Aethina tumida] | 557 | 3 |
| Gene.1778 | EcKinase, DUF1679, and/or APH domain containing protein [Asbolus verrucosus] | 319 | 3 |
| Gene.1779 | PREDICTED: lambda-crystallin [Tribolium castaneum] | 101 | 3 |
| Gene.1779 | PREDICTED: lambda-crystallin [Tribolium castaneum] | 263 | 3 |
| Gene.1789 | thioredoxin domain-containing protein 5 [Asbolus verrucosus] | 324 | 3 |
| Gene.179 | dipeptidyl peptidase 3 [Anoplophora glabripennis] | 332 | 3 |
| Gene.1792 | PREDICTED: zinc finger RNA-binding protein isoform X2 [Aethina tumida] | 524 | 3 |
| Gene.1810 | mitochondrial inner membrane protein OXA1L isoform X1 [Anoplophora glabripennis] | 467 | 3 |
| Gene.1814 | PREDICTED: probable 39S ribosomal protein L45, mitochondrial [Nicrophorus vespilloides] | 131 | 3 |
| Gene.182 | cytosolic non-specific dipeptidase [Anoplophora glabripennis] | 52 | 3 |
| Gene.182 | cytosolic non-specific dipeptidase [Anoplophora glabripennis] | 453 | 3 |
| Gene.182 | cytosolic non-specific dipeptidase [Anoplophora glabripennis] | 127 | 3 |
| Gene.182 | cytosolic non-specific dipeptidase [Anoplophora glabripennis] | 342 | 3 |
| Gene.182 | cytosolic non-specific dipeptidase [Anoplophora glabripennis] | 40 | 3 |
| Gene.182 | cytosolic non-specific dipeptidase [Anoplophora glabripennis] | 338 | 3 |
| Gene.182 | cytosolic non-specific dipeptidase [Anoplophora glabripennis] | 288 | 3 |
| Gene.1821 | PREDICTED: glycerophosphodiester phosphodiesterase 1 [Tribolium castaneum] | 198 | 3 |
| Gene.1845 | PREDICTED: glycogen synthase kinase-3 beta isoform X10 [Tribolium castaneum] | 92 | 3 |
| Gene.1845 | PREDICTED: glycogen synthase kinase-3 beta isoform X10 [Tribolium castaneum] | 87 | 3 |
| Gene.1846 | ATP synthase subunit beta, mitochondrial [Tribolium castaneum] | 128 | 3 |
| Gene.1846 | ATP synthase subunit beta, mitochondrial [Tribolium castaneum] | 497 | 3 |
| Gene.1846 | ATP synthase subunit beta, mitochondrial [Tribolium castaneum] | 263 | 3 |
| Gene.1846 | ATP synthase subunit beta, mitochondrial [Tribolium castaneum] | 202 | 3 |
| Gene.1846 | ATP synthase subunit beta, mitochondrial [Tribolium castaneum] | 268 | 3 |
| Gene.1846 | ATP synthase subunit beta, mitochondrial [Tribolium castaneum] | 430 | 3 |
| Gene.1846 | ATP synthase subunit beta, mitochondrial [Tribolium castaneum] | 205 | 3 |
| Gene.1862 | PREDICTED: trifunctional enzyme subunit beta, mitochondrial [Tribolium castaneum] | 271 | 3 |
| Gene.1862 | PREDICTED: trifunctional enzyme subunit beta, mitochondrial [Tribolium castaneum] | 251 | 3 |
| Gene.1862 | PREDICTED: trifunctional enzyme subunit beta, mitochondrial [Tribolium castaneum] | 83 | 3 |
| Gene.1862 | PREDICTED: trifunctional enzyme subunit beta, mitochondrial [Tribolium castaneum] | 328 | 3 |
| Gene.1862 | PREDICTED: trifunctional enzyme subunit beta, mitochondrial [Tribolium castaneum] | 267 | 3 |
| Gene.1862 | PREDICTED: trifunctional enzyme subunit beta, mitochondrial [Tribolium castaneum] | 78 | 3 |
| Gene.1862 | PREDICTED: trifunctional enzyme subunit beta, mitochondrial [Tribolium castaneum] | 402 | 3 |
| Gene.1862 | PREDICTED: trifunctional enzyme subunit beta, mitochondrial [Tribolium castaneum] | 395 | 3 |
| Gene.1862 | PREDICTED: trifunctional enzyme subunit beta, mitochondrial [Tribolium castaneum] | 45 | 3 |
| Gene.1862 | PREDICTED: trifunctional enzyme subunit beta, mitochondrial [Tribolium castaneum] | 410 | 3 |
| Gene.187 | PREDICTED: uncharacterized protein LOC106106346 [Papilio polytes] | 235 | 3 |
| Gene.1871 | endoribonuclease Dicer [Anoplophora glabripennis] | 452 | 3 |
| Gene.1876 | aquaporin AQPAn.G isoform X1 [Anoplophora glabripennis] | 16 | 3 |
| Gene.1876 | aquaporin AQPAn.G isoform X1 [Anoplophora glabripennis] | 5 | 3 |
| Gene.1878 | MA3 domain containing protein [Asbolus verrucosus] | 254 | 3 |
| Gene.1878 | MA3 domain containing protein [Asbolus verrucosus] | 248 | 3 |
| Gene.189 | mitochondrial import receptor subunit TOM70 [Agrilus planipennis] | 83 | 3 |
| Gene.1891 | hypothetical protein AMK59_3112 [Oryctes borbonicus] | 103 | 3 |
| Gene.1891 | hypothetical protein AMK59_3112 [Oryctes borbonicus] | 88 | 3 |
| Gene.1891 | hypothetical protein AMK59_3112 [Oryctes borbonicus] | 71 | 3 |
| Gene.1891 | hypothetical protein AMK59_3112 [Oryctes borbonicus] | 96 | 3 |
| Gene.1892 | PREDICTED: enolase [Musca domestica] | 74 | 3 |
| Gene.1892 | PREDICTED: enolase [Musca domestica] | 221 | 3 |
| Gene.1892 | PREDICTED: enolase [Musca domestica] | 225 | 3 |
| Gene.1892 | PREDICTED: enolase [Musca domestica] | 80 | 3 |
| Gene.1892 | PREDICTED: enolase [Musca domestica] | 205 | 3 |
| Gene.1892 | PREDICTED: enolase [Musca domestica] | 66 | 3 |
| Gene.1892 | PREDICTED: enolase [Musca domestica] | 133 | 3 |
| Gene.1895 | heat shock protein 70 [Harmonia axyridis] | 271 | 3 |
| Gene.1895 | heat shock protein 70 [Harmonia axyridis] | 365 | 3 |
| Gene.1895 | heat shock protein 70 [Harmonia axyridis] | 26 | 3 |
| Gene.1895 | heat shock protein 70 [Harmonia axyridis] | 475 | 3 |
| Gene.1895 | heat shock protein 70 [Harmonia axyridis] | 438 | 3 |
| Gene.1895 | heat shock protein 70 [Harmonia axyridis] | 42 | 3 |
| Gene.1895 | heat shock protein 70 [Harmonia axyridis] | 22 | 3 |
| Gene.1895 | heat shock protein 70 [Harmonia axyridis] | 473 | 3 |
| Gene.1895 | heat shock protein 70 [Harmonia axyridis] | 233 | 3 |
| Gene.1895 | heat shock protein 70 [Harmonia axyridis] | 426 | 3 |
| Gene.1895 | heat shock protein 70 [Harmonia axyridis] | 40 | 3 |
| Gene.1895 | heat shock protein 70 [Harmonia axyridis] | 509 | 3 |
| Gene.1895 | heat shock protein 70 [Harmonia axyridis] | 160 | 3 |
| Gene.1895 | heat shock protein 70 [Harmonia axyridis] | 421 | 3 |
| Gene.1895 | heat shock protein 70 [Harmonia axyridis] | 414 | 3 |
| Gene.1895 | heat shock protein 70 [Harmonia axyridis] | 242 | 3 |
| Gene.1899 | 40S ribosomal protein S18 [Agrilus planipennis] | 87 | 3 |
| Gene.1899 | 40S ribosomal protein S18 [Agrilus planipennis] | 103 | 3 |
| Gene.1899 | 40S ribosomal protein S18 [Agrilus planipennis] | 97 | 3 |
| Gene.1899 | 40S ribosomal protein S18 [Agrilus planipennis] | 62 | 3 |
| Gene.1899 | 40S ribosomal protein S18 [Agrilus planipennis] | 115 | 3 |
| Gene.1899 | 40S ribosomal protein S18 [Agrilus planipennis] | 55 | 3 |
| Gene.1900 | Neprilysin-2-like Protein [Tribolium castaneum] | 478 | 3 |
| Gene.1904 | PREDICTED: cathepsin B [Tribolium castaneum] | 209 | 3 |
| Gene.1912 | PREDICTED: NADH dehydrogenase [ubiquinone] 1 beta subcomplex subunit 9 [Aethina tumida] | 120 | 3 |
| Gene.1912 | PREDICTED: NADH dehydrogenase [ubiquinone] 1 beta subcomplex subunit 9 [Aethina tumida] | 51 | 3 |
| Gene.1914 | 3-hydroxyisobutyryl-CoA hydrolase, mitochondrial isoform X1 [Anoplophora glabripennis] | 68 | 3 |
| Gene.1916 | RRM 1 domain containing protein [Asbolus verrucosus] | 64 | 3 |
| Gene.1917 | PREDICTED: calnexin [Tribolium castaneum] | 93 | 3 |
| Gene.1917 | PREDICTED: calnexin [Tribolium castaneum] | 502 | 3 |
| Gene.1921 | obg-like ATPase 1 [Anoplophora glabripennis] | 166 | 3 |
| Gene.1921 | obg-like ATPase 1 [Anoplophora glabripennis] | 282 | 3 |
| Gene.1921 | obg-like ATPase 1 [Anoplophora glabripennis] | 188 | 3 |
| Gene.1921 | obg-like ATPase 1 [Anoplophora glabripennis] | 247 | 3 |
| Gene.1932 | PREDICTED: 2-oxoglutarate dehydrogenase, mitochondrial isoform X2 [Tribolium castaneum] | 198 | 3 |
| Gene.1932 | PREDICTED: 2-oxoglutarate dehydrogenase, mitochondrial isoform X2 [Tribolium castaneum] | 188 | 3 |
| Gene.1932 | PREDICTED: 2-oxoglutarate dehydrogenase, mitochondrial isoform X2 [Tribolium castaneum] | 389 | 3 |
| Gene.1932 | PREDICTED: 2-oxoglutarate dehydrogenase, mitochondrial isoform X2 [Tribolium castaneum] | 177 | 3 |
| Gene.1932 | PREDICTED: 2-oxoglutarate dehydrogenase, mitochondrial isoform X2 [Tribolium castaneum] | 616 | 3 |
| Gene.1945 | phosphoserine phosphatase [Asbolus verrucosus] | 74 | 3 |
| Gene.195 | uncharacterized protein LOC108733652 [Agrilus planipennis] | 236 | 3 |
| Gene.1951 | PREDICTED: TPPP family protein CG45057-like [Aethina tumida] | 81 | 3 |
| Gene.1951 | PREDICTED: TPPP family protein CG45057-like [Aethina tumida] | 98 | 3 |
| Gene.1958 | PREDICTED: fimbrin isoform X1 [Tribolium castaneum] | 598 | 3 |
| Gene.1958 | PREDICTED: fimbrin isoform X1 [Tribolium castaneum] | 436 | 3 |
| Gene.1958 | PREDICTED: fimbrin isoform X1 [Tribolium castaneum] | 320 | 3 |
| Gene.1958 | PREDICTED: fimbrin isoform X1 [Tribolium castaneum] | 432 | 3 |
| Gene.1958 | PREDICTED: fimbrin isoform X1 [Tribolium castaneum] | 47 | 3 |
| Gene.196 | PREDICTED: triosephosphate isomerase isoform X1 [Aethina tumida] | 197 | 3 |
| Gene.196 | PREDICTED: triosephosphate isomerase isoform X1 [Aethina tumida] | 25 | 3 |
| Gene.196 | PREDICTED: triosephosphate isomerase isoform X1 [Aethina tumida] | 67 | 3 |
| Gene.196 | PREDICTED: triosephosphate isomerase isoform X1 [Aethina tumida] | 189 | 3 |
| Gene.196 | PREDICTED: triosephosphate isomerase isoform X1 [Aethina tumida] | 217 | 3 |
| Gene.196 | PREDICTED: triosephosphate isomerase isoform X1 [Aethina tumida] | 118 | 3 |
| Gene.196 | PREDICTED: triosephosphate isomerase isoform X1 [Aethina tumida] | 70 | 3 |
| Gene.196 | PREDICTED: triosephosphate isomerase isoform X1 [Aethina tumida] | 83 | 3 |
| Gene.196 | PREDICTED: triosephosphate isomerase isoform X1 [Aethina tumida] | 12 | 3 |
| Gene.196 | PREDICTED: triosephosphate isomerase isoform X1 [Aethina tumida] | 173 | 3 |
| Gene.1982 | Tetratricopeptide repeat-containing protein, partial [Oryctes borbonicus] | 463 | 3 |
| Gene.1982 | Tetratricopeptide repeat-containing protein, partial [Oryctes borbonicus] | 318 | 3 |
| Gene.1982 | Tetratricopeptide repeat-containing protein, partial [Oryctes borbonicus] | 587 | 3 |
| Gene.199 | PREDICTED: histone-lysine N-methyltransferase setd3 [Aethina tumida] | 66 | 3 |
| Gene.1990 | heat shock protein 10.87 [Harmonia axyridis] | 99 | 3 |
| Gene.1990 | heat shock protein 10.87 [Harmonia axyridis] | 55 | 3 |
| Gene.2012 | ribosomal protein L18, partial [Harmonia axyridis] | 39 | 3 |
| Gene.2012 | ribosomal protein L18, partial [Harmonia axyridis] | 51 | 3 |
| Gene.2012 | ribosomal protein L18, partial [Harmonia axyridis] | 54 | 3 |
| Gene.2014 | hypothetical protein AMK59_1391, partial [Oryctes borbonicus] | 118 | 3 |
| Gene.2014 | hypothetical protein AMK59_1391, partial [Oryctes borbonicus] | 425 | 3 |
| Gene.2016 | PREDICTED: 40S ribosomal protein S4 isoform X1 [Aethina tumida] | 63 | 3 |
| Gene.2016 | PREDICTED: 40S ribosomal protein S4 isoform X1 [Aethina tumida] | 120 | 3 |
| Gene.2016 | PREDICTED: 40S ribosomal protein S4 isoform X1 [Aethina tumida] | 134 | 3 |
| Gene.2016 | PREDICTED: 40S ribosomal protein S4 isoform X1 [Aethina tumida] | 245 | 3 |
| Gene.2016 | PREDICTED: 40S ribosomal protein S4 isoform X1 [Aethina tumida] | 230 | 3 |
| Gene.2016 | PREDICTED: 40S ribosomal protein S4 isoform X1 [Aethina tumida] | 106 | 3 |
| Gene.2016 | PREDICTED: 40S ribosomal protein S4 isoform X1 [Aethina tumida] | 37 | 3 |
| Gene.2016 | PREDICTED: 40S ribosomal protein S4 isoform X1 [Aethina tumida] | 16 | 3 |
| Gene.2017 | ATP synthase subunit O, mitochondrial [Asbolus verrucosus] | 103 | 3 |
| Gene.2017 | ATP synthase subunit O, mitochondrial [Asbolus verrucosus] | 182 | 3 |
| Gene.2017 | ATP synthase subunit O, mitochondrial [Asbolus verrucosus] | 91 | 3 |
| Gene.2017 | ATP synthase subunit O, mitochondrial [Asbolus verrucosus] | 184 | 3 |
| Gene.2017 | ATP synthase subunit O, mitochondrial [Asbolus verrucosus] | 121 | 3 |
| Gene.202 | hypothetical protein YQE_03922, partial [Dendroctonus ponderosae] | 116 | 3 |
| Gene.2034 | NADH dehydrogenase [ubiquinone] flavoprotein 1, mitochondrial [Tribolium castaneum] | 381 | 3 |
| Gene.2034 | NADH dehydrogenase [ubiquinone] flavoprotein 1, mitochondrial [Tribolium castaneum] | 471 | 3 |
| Gene.2034 | NADH dehydrogenase [ubiquinone] flavoprotein 1, mitochondrial [Tribolium castaneum] | 47 | 3 |
| Gene.2037 | ATPase family AAA domain-containing protein 3A homolog [Leptinotarsa decemlineata] | 272 | 3 |
| Gene.204 | ribosomal protein L28, partial [Harmonia axyridis] | 92 | 3 |
| Gene.204 | ribosomal protein L28, partial [Harmonia axyridis] | 26 | 3 |
| Gene.204 | ribosomal protein L28, partial [Harmonia axyridis] | 55 | 3 |
| Gene.2052 | UDP-glucuronosyltransferase 2C1-like, partial [Asbolus verrucosus] | 422 | 3 |
| Gene.2064 | PREDICTED: trans-1,2-dihydrobenzene-1,2-diol dehydrogenase [Tribolium castaneum] | 292 | 3 |
| Gene.210 | elongation factor 1 alpha, partial [Harmonia axyridis] | 385 | 3 |
| Gene.210 | elongation factor 1 alpha, partial [Harmonia axyridis] | 330 | 3 |
| Gene.210 | elongation factor 1 alpha, partial [Harmonia axyridis] | 41 | 3 |
| Gene.210 | elongation factor 1 alpha, partial [Harmonia axyridis] | 392 | 3 |
| Gene.210 | elongation factor 1 alpha, partial [Harmonia axyridis] | 212 | 3 |
| Gene.210 | elongation factor 1 alpha, partial [Harmonia axyridis] | 154 | 3 |
| Gene.210 | elongation factor 1 alpha, partial [Harmonia axyridis] | 450 | 3 |
| Gene.210 | elongation factor 1 alpha, partial [Harmonia axyridis] | 179 | 3 |
| Gene.210 | elongation factor 1 alpha, partial [Harmonia axyridis] | 444 | 3 |
| Gene.210 | elongation factor 1 alpha, partial [Harmonia axyridis] | 30 | 3 |
| Gene.210 | elongation factor 1 alpha, partial [Harmonia axyridis] | 255 | 3 |
| Gene.210 | elongation factor 1 alpha, partial [Harmonia axyridis] | 44 | 3 |
| Gene.2113 | 60S ribosomal protein L31 [Blattella germanica] | 112 | 3 |
| Gene.2113 | 60S ribosomal protein L31 [Blattella germanica] | 72 | 3 |
| Gene.2124 | PREDICTED: sorbitol dehydrogenase [Tribolium castaneum] | 210 | 3 |
| Gene.2124 | PREDICTED: sorbitol dehydrogenase [Tribolium castaneum] | 83 | 3 |
| Gene.2124 | PREDICTED: sorbitol dehydrogenase [Tribolium castaneum] | 330 | 3 |
| Gene.2124 | PREDICTED: sorbitol dehydrogenase [Tribolium castaneum] | 322 | 3 |
| Gene.2124 | PREDICTED: sorbitol dehydrogenase [Tribolium castaneum] | 316 | 3 |
| Gene.2124 | PREDICTED: sorbitol dehydrogenase [Tribolium castaneum] | 324 | 3 |
| Gene.2124 | PREDICTED: sorbitol dehydrogenase [Tribolium castaneum] | 344 | 3 |
| Gene.2124 | PREDICTED: sorbitol dehydrogenase [Tribolium castaneum] | 219 | 3 |
| Gene.2124 | PREDICTED: sorbitol dehydrogenase [Tribolium castaneum] | 351 | 3 |
| Gene.2134 | PREDICTED: 40S ribosomal protein S7 [Tribolium castaneum] | 174 | 3 |
| Gene.2134 | PREDICTED: 40S ribosomal protein S7 [Tribolium castaneum] | 53 | 3 |
| Gene.2134 | PREDICTED: 40S ribosomal protein S7 [Tribolium castaneum] | 64 | 3 |
| Gene.2134 | PREDICTED: 40S ribosomal protein S7 [Tribolium castaneum] | 70 | 3 |
| Gene.2134 | PREDICTED: 40S ribosomal protein S7 [Tribolium castaneum] | 151 | 3 |
| Gene.2134 | PREDICTED: 40S ribosomal protein S7 [Tribolium castaneum] | 143 | 3 |
| Gene.2140 | PREDICTED: UDP-glucuronosyltransferase 2B10-like [Tribolium castaneum] | 401 | 3 |
| Gene.2140 | PREDICTED: UDP-glucuronosyltransferase 2B10-like [Tribolium castaneum] | 80 | 3 |
| Gene.2140 | PREDICTED: UDP-glucuronosyltransferase 2B10-like [Tribolium castaneum] | 425 | 3 |
| Gene.2140 | PREDICTED: UDP-glucuronosyltransferase 2B10-like [Tribolium castaneum] | 410 | 3 |
| Gene.2144 | PREDICTED: succinyl-CoA ligase subunit alpha, mitochondrial [Tribolium castaneum] | 50 | 3 |
| Gene.2144 | PREDICTED: succinyl-CoA ligase subunit alpha, mitochondrial [Tribolium castaneum] | 304 | 3 |
| Gene.2144 | PREDICTED: succinyl-CoA ligase subunit alpha, mitochondrial [Tribolium castaneum] | 57 | 3 |
| Gene.2144 | PREDICTED: succinyl-CoA ligase subunit alpha, mitochondrial [Tribolium castaneum] | 77 | 3 |
| Gene.2144 | PREDICTED: succinyl-CoA ligase subunit alpha, mitochondrial [Tribolium castaneum] | 89 | 3 |
| Gene.2149 | heat shock protein, partial [Cryptolaemus montrouzieri] | 359 | 3 |
| Gene.2149 | heat shock protein, partial [Cryptolaemus montrouzieri] | 387 | 3 |
| Gene.2149 | heat shock protein, partial [Cryptolaemus montrouzieri] | 75 | 3 |
| Gene.2149 | heat shock protein, partial [Cryptolaemus montrouzieri] | 156 | 3 |
| Gene.2149 | heat shock protein, partial [Cryptolaemus montrouzieri] | 108 | 3 |
| Gene.2149 | heat shock protein, partial [Cryptolaemus montrouzieri] | 417 | 3 |
| Gene.2149 | heat shock protein, partial [Cryptolaemus montrouzieri] | 202 | 3 |
| Gene.2149 | heat shock protein, partial [Cryptolaemus montrouzieri] | 469 | 3 |
| Gene.2149 | heat shock protein, partial [Cryptolaemus montrouzieri] | 72 | 3 |
| Gene.2149 | heat shock protein, partial [Cryptolaemus montrouzieri] | 125 | 3 |
| Gene.2149 | heat shock protein, partial [Cryptolaemus montrouzieri] | 89 | 3 |
| Gene.2149 | heat shock protein, partial [Cryptolaemus montrouzieri] | 133 | 3 |
| Gene.2161 | Calcium-binding mitochondrial carrier protein Aralar1-like Protein [Tribolium castaneum] | 470 | 3 |
| Gene.2167 | PREDICTED: 15-hydroxyprostaglandin dehydrogenase [NAD(+)] [Tribolium castaneum] | 59 | 3 |
| Gene.2167 | PREDICTED: 15-hydroxyprostaglandin dehydrogenase [NAD(+)] [Tribolium castaneum] | 233 | 3 |
| Gene.2167 | PREDICTED: 15-hydroxyprostaglandin dehydrogenase [NAD(+)] [Tribolium castaneum] | 53 | 3 |
| Gene.2167 | PREDICTED: 15-hydroxyprostaglandin dehydrogenase [NAD(+)] [Tribolium castaneum] | 64 | 3 |
| Gene.2167 | PREDICTED: 15-hydroxyprostaglandin dehydrogenase [NAD(+)] [Tribolium castaneum] | 45 | 3 |
| Gene.2168 | eukaryotic translation initiation factor 5 [Anoplophora glabripennis] | 266 | 3 |
| Gene.2175 | PREDICTED: 2-hydroxyacylsphingosine 1-beta-galactosyltransferase-like [Tribolium castaneum] | 192 | 3 |
| Gene.2176 | UDP-glucuronosyltransferase 1-9-like isoform X2 [Leptinotarsa decemlineata] | 48 | 3 |
| Gene.2178 | UDPGT and/or Glyco tran 28 C domain containing protein [Asbolus verrucosus] | 47 | 3 |
| Gene.2182 | PREDICTED: palmitoyl-protein thioesterase 1 [Tribolium castaneum] | 293 | 3 |
| Gene.2197 | PREDICTED: peroxisomal multifunctional enzyme type 2 isoform X1 [Tribolium castaneum] | 664 | 3 |
| Gene.2197 | PREDICTED: peroxisomal multifunctional enzyme type 2 isoform X1 [Tribolium castaneum] | 138 | 3 |
| Gene.2197 | PREDICTED: peroxisomal multifunctional enzyme type 2 isoform X1 [Tribolium castaneum] | 275 | 3 |
| Gene.2197 | PREDICTED: peroxisomal multifunctional enzyme type 2 isoform X1 [Tribolium castaneum] | 638 | 3 |
| Gene.2197 | PREDICTED: peroxisomal multifunctional enzyme type 2 isoform X1 [Tribolium castaneum] | 647 | 3 |
| Gene.2199 | nuclear protein localization protein 4 homolog [Anoplophora glabripennis] | 315 | 3 |
| Gene.220 | PREDICTED: synaptic vesicle glycoprotein 2A [Tribolium castaneum] | 226 | 3 |
| Gene.2236 | Glutamate dehydrogenase, mitochondrial-like Protein [Tribolium castaneum] | 516 | 3 |
| Gene.2236 | Glutamate dehydrogenase, mitochondrial-like Protein [Tribolium castaneum] | 412 | 3 |
| Gene.2236 | Glutamate dehydrogenase, mitochondrial-like Protein [Tribolium castaneum] | 492 | 3 |
| Gene.2236 | Glutamate dehydrogenase, mitochondrial-like Protein [Tribolium castaneum] | 457 | 3 |
| Gene.2236 | Glutamate dehydrogenase, mitochondrial-like Protein [Tribolium castaneum] | 87 | 3 |
| Gene.2236 | Glutamate dehydrogenase, mitochondrial-like Protein [Tribolium castaneum] | 186 | 3 |
| Gene.2236 | Glutamate dehydrogenase, mitochondrial-like Protein [Tribolium castaneum] | 372 | 3 |
| Gene.2236 | Glutamate dehydrogenase, mitochondrial-like Protein [Tribolium castaneum] | 477 | 3 |
| Gene.2236 | Glutamate dehydrogenase, mitochondrial-like Protein [Tribolium castaneum] | 500 | 3 |
| Gene.2236 | Glutamate dehydrogenase, mitochondrial-like Protein [Tribolium castaneum] | 82 | 3 |
| Gene.2236 | Glutamate dehydrogenase, mitochondrial-like Protein [Tribolium castaneum] | 195 | 3 |
| Gene.2242 | hypothetical protein AMK59_6191, partial [Oryctes borbonicus] | 411 | 3 |
| Gene.2263 | probable isocitrate dehydrogenase [NAD] subunit alpha, mitochondrial isoform X1 [Anoplophora glabripennis] | 360 | 3 |
| Gene.2263 | probable isocitrate dehydrogenase [NAD] subunit alpha, mitochondrial isoform X1 [Anoplophora glabripennis] | 90 | 3 |
| Gene.2281 | PREDICTED: probable phospholipid hydroperoxide glutathione peroxidase isoform X1 [Aethina tumida] | 95 | 3 |
| Gene.2281 | PREDICTED: probable phospholipid hydroperoxide glutathione peroxidase isoform X1 [Aethina tumida] | 9 | 3 |
| Gene.2281 | PREDICTED: probable phospholipid hydroperoxide glutathione peroxidase isoform X1 [Aethina tumida] | 100 | 3 |
| Gene.2281 | PREDICTED: probable phospholipid hydroperoxide glutathione peroxidase isoform X1 [Aethina tumida] | 12 | 3 |
| Gene.229 | PREDICTED: probable phospholipid hydroperoxide glutathione peroxidase [Tribolium castaneum] | 159 | 3 |
| Gene.229 | PREDICTED: probable phospholipid hydroperoxide glutathione peroxidase [Tribolium castaneum] | 156 | 3 |
| Gene.229 | PREDICTED: probable phospholipid hydroperoxide glutathione peroxidase [Tribolium castaneum] | 183 | 3 |
| Gene.229 | PREDICTED: probable phospholipid hydroperoxide glutathione peroxidase [Tribolium castaneum] | 198 | 3 |
| Gene.229 | PREDICTED: probable phospholipid hydroperoxide glutathione peroxidase [Tribolium castaneum] | 19 | 3 |
| Gene.229 | PREDICTED: probable phospholipid hydroperoxide glutathione peroxidase [Tribolium castaneum] | 126 | 3 |
| Gene.2309 | Gamma-interferon-inducible lysosomal thiol reductase-like Protein [Tribolium castaneum] | 138 | 3 |
| Gene.2317 | PREDICTED: myosin-2 essential light chain isoform X2 [Tribolium castaneum] | 49 | 3 |
| Gene.2317 | PREDICTED: myosin-2 essential light chain isoform X2 [Tribolium castaneum] | 94 | 3 |
| Gene.2318 | PREDICTED: LOW QUALITY PROTEIN: V-type proton ATPase 116 kDa subunit a-like [Aethina tumida] | 109 | 3 |
| Gene.2318 | PREDICTED: LOW QUALITY PROTEIN: V-type proton ATPase 116 kDa subunit a-like [Aethina tumida] | 50 | 3 |
| Gene.2318 | PREDICTED: LOW QUALITY PROTEIN: V-type proton ATPase 116 kDa subunit a-like [Aethina tumida] | 522 | 3 |
| Gene.2318 | PREDICTED: LOW QUALITY PROTEIN: V-type proton ATPase 116 kDa subunit a-like [Aethina tumida] | 266 | 3 |
| Gene.2318 | PREDICTED: LOW QUALITY PROTEIN: V-type proton ATPase 116 kDa subunit a-like [Aethina tumida] | 76 | 3 |
| Gene.2330 | guanine nucleotide-binding protein subunit beta-like protein [Anoplophora glabripennis] | 55 | 3 |
| Gene.2348 | ras-related protein Rab-5B isoform X1 [Leptinotarsa decemlineata] | 116 | 3 |
| Gene.2357 | eukaryotic translation initiation factor 4 gamma 3-like, partial [Asbolus verrucosus] | 165 | 3 |
| Gene.2357 | eukaryotic translation initiation factor 4 gamma 3-like, partial [Asbolus verrucosus] | 739 | 3 |
| Gene.2362 | uncharacterized protein LOC108904898 isoform X1 [Anoplophora glabripennis] | 32 | 3 |
| Gene.2362 | uncharacterized protein LOC108904898 isoform X1 [Anoplophora glabripennis] | 28 | 3 |
| Gene.2362 | uncharacterized protein LOC108904898 isoform X1 [Anoplophora glabripennis] | 85 | 3 |
| Gene.2362 | uncharacterized protein LOC108904898 isoform X1 [Anoplophora glabripennis] | 94 | 3 |
| Gene.2362 | uncharacterized protein LOC108904898 isoform X1 [Anoplophora glabripennis] | 102 | 3 |
| Gene.2362 | uncharacterized protein LOC108904898 isoform X1 [Anoplophora glabripennis] | 78 | 3 |
| Gene.2362 | uncharacterized protein LOC108904898 isoform X1 [Anoplophora glabripennis] | 49 | 3 |
| Gene.2365 | PREDICTED: maternal protein tudor isoform X1 [Tribolium castaneum] | 225 | 3 |
| Gene.2365 | PREDICTED: maternal protein tudor isoform X1 [Tribolium castaneum] | 1258 | 3 |
| Gene.2368 | PREDICTED: filamin-A isoform X6 [Tribolium castaneum] | 2143 | 3 |
| Gene.2368 | PREDICTED: filamin-A isoform X6 [Tribolium castaneum] | 1801 | 3 |
| Gene.2368 | PREDICTED: filamin-A isoform X6 [Tribolium castaneum] | 585 | 3 |
| Gene.2368 | PREDICTED: filamin-A isoform X6 [Tribolium castaneum] | 1534 | 3 |
| Gene.2368 | PREDICTED: filamin-A isoform X6 [Tribolium castaneum] | 2141 | 3 |
| Gene.2368 | PREDICTED: filamin-A isoform X6 [Tribolium castaneum] | 49 | 3 |
| Gene.2368 | PREDICTED: filamin-A isoform X6 [Tribolium castaneum] | 867 | 3 |
| Gene.2368 | PREDICTED: filamin-A isoform X6 [Tribolium castaneum] | 2100 | 3 |
| Gene.237 | PREDICTED: uncharacterized protein LOC661814 [Tribolium castaneum] | 59 | 3 |
| Gene.237 | PREDICTED: uncharacterized protein LOC661814 [Tribolium castaneum] | 28 | 3 |
| Gene.237 | PREDICTED: uncharacterized protein LOC661814 [Tribolium castaneum] | 57 | 3 |
| Gene.237 | PREDICTED: uncharacterized protein LOC661814 [Tribolium castaneum] | 30 | 3 |
| Gene.237 | PREDICTED: uncharacterized protein LOC661814 [Tribolium castaneum] | 91 | 3 |
| Gene.237 | PREDICTED: uncharacterized protein LOC661814 [Tribolium castaneum] | 211 | 3 |
| Gene.2372 | cytochrome P450 CYP9Z401 [Cryptolaemus montrouzieri] | 119 | 3 |
| Gene.2372 | cytochrome P450 CYP9Z401 [Cryptolaemus montrouzieri] | 138 | 3 |
| Gene.2375 | glyceraldehyde-3-phosphate, partial [Harmonia axyridis] | 90 | 3 |
| Gene.2375 | glyceraldehyde-3-phosphate, partial [Harmonia axyridis] | 330 | 3 |
| Gene.2375 | glyceraldehyde-3-phosphate, partial [Harmonia axyridis] | 264 | 3 |
| Gene.2375 | glyceraldehyde-3-phosphate, partial [Harmonia axyridis] | 84 | 3 |
| Gene.2375 | glyceraldehyde-3-phosphate, partial [Harmonia axyridis] | 252 | 3 |
| Gene.2375 | glyceraldehyde-3-phosphate, partial [Harmonia axyridis] | 228 | 3 |
| Gene.2375 | glyceraldehyde-3-phosphate, partial [Harmonia axyridis] | 58 | 3 |
| Gene.2375 | glyceraldehyde-3-phosphate, partial [Harmonia axyridis] | 163 | 3 |
| Gene.2375 | glyceraldehyde-3-phosphate, partial [Harmonia axyridis] | 73 | 3 |
| Gene.2375 | glyceraldehyde-3-phosphate, partial [Harmonia axyridis] | 66 | 3 |
| Gene.2375 | glyceraldehyde-3-phosphate, partial [Harmonia axyridis] | 260 | 3 |
| Gene.2375 | glyceraldehyde-3-phosphate, partial [Harmonia axyridis] | 195 | 3 |
| Gene.2375 | glyceraldehyde-3-phosphate, partial [Harmonia axyridis] | 223 | 3 |
| Gene.2402 | mitochondrial import receptor subunit TOM20 homolog B-like [Leptinotarsa decemlineata] | 39 | 3 |
| Gene.2408 | PREDICTED: four and a half LIM domains protein 2 isoform X5 [Tribolium castaneum] | 238 | 3 |
| Gene.2408 | PREDICTED: four and a half LIM domains protein 2 isoform X5 [Tribolium castaneum] | 128 | 3 |
| Gene.2408 | PREDICTED: four and a half LIM domains protein 2 isoform X5 [Tribolium castaneum] | 138 | 3 |
| Gene.2408 | PREDICTED: four and a half LIM domains protein 2 isoform X5 [Tribolium castaneum] | 253 | 3 |
| Gene.2408 | PREDICTED: four and a half LIM domains protein 2 isoform X5 [Tribolium castaneum] | 305 | 3 |
| Gene.2408 | PREDICTED: four and a half LIM domains protein 2 isoform X5 [Tribolium castaneum] | 263 | 3 |
| Gene.2408 | PREDICTED: four and a half LIM domains protein 2 isoform X5 [Tribolium castaneum] | 279 | 3 |
| Gene.2408 | PREDICTED: four and a half LIM domains protein 2 isoform X5 [Tribolium castaneum] | 143 | 3 |
| Gene.2408 | PREDICTED: four and a half LIM domains protein 2 isoform X5 [Tribolium castaneum] | 243 | 3 |
| Gene.2418 | uncharacterized protein LOC111509962 [Leptinotarsa decemlineata] | 80 | 3 |
| Gene.2438 | PREDICTED: double-stranded RNA-specific editase Adar-like isoform X1 [Aethina tumida] | 286 | 3 |
| Gene.2438 | PREDICTED: double-stranded RNA-specific editase Adar-like isoform X1 [Aethina tumida] | 56 | 3 |
| Gene.246 | PREDICTED: V-type proton ATPase subunit d [Tribolium castaneum] | 39 | 3 |
| Gene.2466 | PREDICTED: LOW QUALITY PROTEIN: extended synaptotagmin-2-like [Aethina tumida] | 14 | 3 |
| Gene.2469 | PREDICTED: flightin [Tribolium castaneum] | 129 | 3 |
| Gene.2469 | PREDICTED: flightin [Tribolium castaneum] | 79 | 3 |
| Gene.2472 | PREDICTED: natterin-4-like isoform X2 [Tribolium castaneum] | 102 | 3 |
| Gene.2484 | PREDICTED: fatty-acid amide hydrolase 2-B [Tribolium castaneum] | 91 | 3 |
| Gene.2493 | PREDICTED: vesicular glutamate transporter 2.2 [Tribolium castaneum] | 246 | 3 |
| Gene.2505 | PREDICTED: 1-acyl-sn-glycerol-3-phosphate acyltransferase delta [Tribolium castaneum] | 291 | 3 |
| Gene.2505 | PREDICTED: 1-acyl-sn-glycerol-3-phosphate acyltransferase delta [Tribolium castaneum] | 287 | 3 |
| Gene.2517 | effete, isoform A [Drosophila melanogaster] | 133 | 3 |
| Gene.2522 | cytochrome c oxidase subunit 5A, mitochondrial [Anoplophora glabripennis] | 46 | 3 |
| Gene.2522 | cytochrome c oxidase subunit 5A, mitochondrial [Anoplophora glabripennis] | 101 | 3 |
| Gene.2522 | cytochrome c oxidase subunit 5A, mitochondrial [Anoplophora glabripennis] | 51 | 3 |
| Gene.2533 | PREDICTED: putative aldehyde dehydrogenase family 7 member A1 homolog [Aethina tumida] | 286 | 3 |
| Gene.2533 | PREDICTED: putative aldehyde dehydrogenase family 7 member A1 homolog [Aethina tumida] | 400 | 3 |
| Gene.2533 | PREDICTED: putative aldehyde dehydrogenase family 7 member A1 homolog [Aethina tumida] | 41 | 3 |
| Gene.2533 | PREDICTED: putative aldehyde dehydrogenase family 7 member A1 homolog [Aethina tumida] | 56 | 3 |
| Gene.2533 | PREDICTED: putative aldehyde dehydrogenase family 7 member A1 homolog [Aethina tumida] | 382 | 3 |
| Gene.2533 | PREDICTED: putative aldehyde dehydrogenase family 7 member A1 homolog [Aethina tumida] | 73 | 3 |
| Gene.2533 | PREDICTED: putative aldehyde dehydrogenase family 7 member A1 homolog [Aethina tumida] | 62 | 3 |
| Gene.2533 | PREDICTED: putative aldehyde dehydrogenase family 7 member A1 homolog [Aethina tumida] | 65 | 3 |
| Gene.2560 | protein transport protein Sec24C [Anoplophora glabripennis] | 1198 | 3 |
| Gene.2563 | PREDICTED: aldose reductase [Tribolium castaneum] | 97 | 3 |
| Gene.2565 | PREDICTED: protein hu-li tai shao isoform X2 [Tribolium castaneum] | 567 | 3 |
| Gene.2565 | PREDICTED: protein hu-li tai shao isoform X2 [Tribolium castaneum] | 582 | 3 |
| Gene.2569 | 1,5-anhydro-D-fructose reductase-like Protein [Tribolium castaneum] | 27 | 3 |
| Gene.2569 | 1,5-anhydro-D-fructose reductase-like Protein [Tribolium castaneum] | 70 | 3 |
| Gene.2569 | 1,5-anhydro-D-fructose reductase-like Protein [Tribolium castaneum] | 9 | 3 |
| Gene.2569 | 1,5-anhydro-D-fructose reductase-like Protein [Tribolium castaneum] | 315 | 3 |
| Gene.2569 | 1,5-anhydro-D-fructose reductase-like Protein [Tribolium castaneum] | 60 | 3 |
| Gene.2569 | 1,5-anhydro-D-fructose reductase-like Protein [Tribolium castaneum] | 4 | 3 |
| Gene.2569 | 1,5-anhydro-D-fructose reductase-like Protein [Tribolium castaneum] | 56 | 3 |
| Gene.2569 | 1,5-anhydro-D-fructose reductase-like Protein [Tribolium castaneum] | 169 | 3 |
| Gene.2582 | rho GDP-dissociation inhibitor 1 [Anoplophora glabripennis] | 127 | 3 |
| Gene.2582 | rho GDP-dissociation inhibitor 1 [Anoplophora glabripennis] | 45 | 3 |
| Gene.2583 | rRNA 2'-O-methyltransferase fibrillarin [Agrilus planipennis] | 306 | 3 |
| Gene.2599 | PREDICTED: sodium/potassium-transporting ATPase subunit alpha isoform X3 [Tribolium castaneum] | 765 | 3 |
| Gene.2605 | UV excision repair protein RAD23 homolog B [Agrilus planipennis] | 34 | 3 |
| Gene.2605 | UV excision repair protein RAD23 homolog B [Agrilus planipennis] | 46 | 3 |
| Gene.2605 | UV excision repair protein RAD23 homolog B [Agrilus planipennis] | 68 | 3 |
| Gene.261 | PREDICTED: bifunctional purine biosynthesis protein PURH [Tribolium castaneum] | 149 | 3 |
| Gene.261 | PREDICTED: bifunctional purine biosynthesis protein PURH [Tribolium castaneum] | 501 | 3 |
| Gene.261 | PREDICTED: bifunctional purine biosynthesis protein PURH [Tribolium castaneum] | 461 | 3 |
| Gene.261 | PREDICTED: bifunctional purine biosynthesis protein PURH [Tribolium castaneum] | 66 | 3 |
| Gene.261 | PREDICTED: bifunctional purine biosynthesis protein PURH [Tribolium castaneum] | 89 | 3 |
| Gene.261 | PREDICTED: bifunctional purine biosynthesis protein PURH [Tribolium castaneum] | 477 | 3 |
| Gene.261 | PREDICTED: bifunctional purine biosynthesis protein PURH [Tribolium castaneum] | 266 | 3 |
| Gene.2624 | PREDICTED: NADH dehydrogenase [ubiquinone] 1 alpha subcomplex subunit 5 [Tribolium castaneum] | 70 | 3 |
| Gene.2626 | Eukaryotic translation initiation factor 3 subunit B-like Protein [Tribolium castaneum] | 133 | 3 |
| Gene.2646 | beta actin [Polyrhachis vicina] | 99 | 3 |
| Gene.2646 | beta actin [Polyrhachis vicina] | 88 | 3 |
| Gene.2646 | beta actin [Polyrhachis vicina] | 64 | 3 |
| Gene.2646 | beta actin [Polyrhachis vicina] | 101 | 3 |
| Gene.2650 | PREDICTED: succinyl-CoA ligase [ADP-forming] subunit beta, mitochondrial [Tribolium castaneum] | 237 | 3 |
| Gene.2650 | PREDICTED: succinyl-CoA ligase [ADP-forming] subunit beta, mitochondrial [Tribolium castaneum] | 350 | 3 |
| Gene.2650 | PREDICTED: succinyl-CoA ligase [ADP-forming] subunit beta, mitochondrial [Tribolium castaneum] | 221 | 3 |
| Gene.2650 | PREDICTED: succinyl-CoA ligase [ADP-forming] subunit beta, mitochondrial [Tribolium castaneum] | 400 | 3 |
| Gene.2650 | PREDICTED: succinyl-CoA ligase [ADP-forming] subunit beta, mitochondrial [Tribolium castaneum] | 389 | 3 |
| Gene.2650 | PREDICTED: succinyl-CoA ligase [ADP-forming] subunit beta, mitochondrial [Tribolium castaneum] | 288 | 3 |
| Gene.2650 | PREDICTED: succinyl-CoA ligase [ADP-forming] subunit beta, mitochondrial [Tribolium castaneum] | 214 | 3 |
| Gene.2650 | PREDICTED: succinyl-CoA ligase [ADP-forming] subunit beta, mitochondrial [Tribolium castaneum] | 405 | 3 |
| Gene.2650 | PREDICTED: succinyl-CoA ligase [ADP-forming] subunit beta, mitochondrial [Tribolium castaneum] | 62 | 3 |
| Gene.2650 | PREDICTED: succinyl-CoA ligase [ADP-forming] subunit beta, mitochondrial [Tribolium castaneum] | 223 | 3 |
| Gene.2650 | PREDICTED: succinyl-CoA ligase [ADP-forming] subunit beta, mitochondrial [Tribolium castaneum] | 82 | 3 |
| Gene.2656 | PREDICTED: aspartate aminotransferase, cytoplasmic [Tribolium castaneum] | 56 | 3 |
| Gene.2656 | PREDICTED: aspartate aminotransferase, cytoplasmic [Tribolium castaneum] | 141 | 3 |
| Gene.2682 | heterogeneous nuclear ribonucleoprotein K isoform X1 [Anoplophora glabripennis] | 324 | 3 |
| Gene.2683 | PREDICTED: proteasome subunit beta type-2 [Tribolium castaneum] | 34 | 3 |
| Gene.2683 | PREDICTED: proteasome subunit beta type-2 [Tribolium castaneum] | 37 | 3 |
| Gene.2683 | PREDICTED: proteasome subunit beta type-2 [Tribolium castaneum] | 148 | 3 |
| Gene.2683 | PREDICTED: proteasome subunit beta type-2 [Tribolium castaneum] | 78 | 3 |
| Gene.2684 | PREDICTED: minor histocompatibility antigen H13 [Tribolium castaneum] | 60 | 3 |
| Gene.2689 | CoaE and/or CTP transf 2 domain containing protein [Asbolus verrucosus] | 127 | 3 |
| Gene.2689 | CoaE and/or CTP transf 2 domain containing protein [Asbolus verrucosus] | 154 | 3 |
| Gene.269 | PREDICTED: cytosol aminopeptidase isoform X2 [Tribolium castaneum] | 241 | 3 |
| Gene.269 | PREDICTED: cytosol aminopeptidase isoform X2 [Tribolium castaneum] | 58 | 3 |
| Gene.2703 | PREDICTED: very-long-chain 3-oxoacyl-CoA reductase [Aethina tumida] | 109 | 3 |
| Gene.2703 | PREDICTED: very-long-chain 3-oxoacyl-CoA reductase [Aethina tumida] | 45 | 3 |
| Gene.272 | PREDICTED: aspartic proteinase A2 [Tribolium castaneum] | 395 | 3 |
| Gene.272 | PREDICTED: aspartic proteinase A2 [Tribolium castaneum] | 344 | 3 |
| Gene.272 | PREDICTED: aspartic proteinase A2 [Tribolium castaneum] | 239 | 3 |
| Gene.2724 | PREDICTED: endoplasmin [Tribolium castaneum] | 29 | 3 |
| Gene.2724 | PREDICTED: endoplasmin [Tribolium castaneum] | 2 | 3 |
| Gene.2725 | PREDICTED: endoplasmin [Tribolium castaneum] | 549 | 3 |
| Gene.2725 | PREDICTED: endoplasmin [Tribolium castaneum] | 554 | 3 |
| Gene.2725 | PREDICTED: endoplasmin [Tribolium castaneum] | 81 | 3 |
| Gene.2725 | PREDICTED: endoplasmin [Tribolium castaneum] | 103 | 3 |
| Gene.2725 | PREDICTED: endoplasmin [Tribolium castaneum] | 490 | 3 |
| Gene.2749 | PREDICTED: aldehyde dehydrogenase, mitochondrial [Tribolium castaneum] | 84 | 3 |
| Gene.2749 | PREDICTED: aldehyde dehydrogenase, mitochondrial [Tribolium castaneum] | 370 | 3 |
| Gene.2749 | PREDICTED: aldehyde dehydrogenase, mitochondrial [Tribolium castaneum] | 446 | 3 |
| Gene.2749 | PREDICTED: aldehyde dehydrogenase, mitochondrial [Tribolium castaneum] | 151 | 3 |
| Gene.2749 | PREDICTED: aldehyde dehydrogenase, mitochondrial [Tribolium castaneum] | 423 | 3 |
| Gene.2749 | PREDICTED: aldehyde dehydrogenase, mitochondrial [Tribolium castaneum] | 140 | 3 |
| Gene.2752 | PREDICTED: valine--tRNA ligase [Aethina tumida] | 368 | 3 |
| Gene.2752 | PREDICTED: valine--tRNA ligase [Aethina tumida] | 432 | 3 |
| Gene.2764 | PREDICTED: alpha-L-fucosidase [Nicrophorus vespilloides] | 209 | 3 |
| Gene.2767 | ribosomal protein L10, partial [Harmonia axyridis] | 83 | 3 |
| Gene.2767 | ribosomal protein L10, partial [Harmonia axyridis] | 91 | 3 |
| Gene.2767 | ribosomal protein L10, partial [Harmonia axyridis] | 184 | 3 |
| Gene.2767 | ribosomal protein L10, partial [Harmonia axyridis] | 47 | 3 |
| Gene.2767 | ribosomal protein L10, partial [Harmonia axyridis] | 154 | 3 |
| Gene.2768 | probable peptidyl-tRNA hydrolase 2 [Anoplophora glabripennis] | 133 | 3 |
| Gene.2788 | aubergine [Diabrotica virgifera virgifera] | 252 | 3 |
| Gene.2788 | aubergine [Diabrotica virgifera virgifera] | 476 | 3 |
| Gene.2788 | aubergine [Diabrotica virgifera virgifera] | 458 | 3 |
| Gene.2788 | aubergine [Diabrotica virgifera virgifera] | 523 | 3 |
| Gene.2788 | aubergine [Diabrotica virgifera virgifera] | 632 | 3 |
| Gene.2788 | aubergine [Diabrotica virgifera virgifera] | 746 | 3 |
| Gene.2788 | aubergine [Diabrotica virgifera virgifera] | 491 | 3 |
| Gene.2788 | aubergine [Diabrotica virgifera virgifera] | 501 | 3 |
| Gene.2788 | aubergine [Diabrotica virgifera virgifera] | 596 | 3 |
| Gene.2788 | aubergine [Diabrotica virgifera virgifera] | 483 | 3 |
| Gene.2788 | aubergine [Diabrotica virgifera virgifera] | 610 | 3 |
| Gene.2788 | aubergine [Diabrotica virgifera virgifera] | 312 | 3 |
| Gene.2788 | aubergine [Diabrotica virgifera virgifera] | 623 | 3 |
| Gene.2800 | PREDICTED: hydroxysteroid dehydrogenase-like protein 2 [Aethina tumida] | 149 | 3 |
| Gene.2800 | PREDICTED: hydroxysteroid dehydrogenase-like protein 2 [Aethina tumida] | 74 | 3 |
| Gene.2800 | PREDICTED: hydroxysteroid dehydrogenase-like protein 2 [Aethina tumida] | 166 | 3 |
| Gene.2800 | PREDICTED: hydroxysteroid dehydrogenase-like protein 2 [Aethina tumida] | 61 | 3 |
| Gene.2800 | PREDICTED: hydroxysteroid dehydrogenase-like protein 2 [Aethina tumida] | 43 | 3 |
| Gene.2800 | PREDICTED: hydroxysteroid dehydrogenase-like protein 2 [Aethina tumida] | 270 | 3 |
| Gene.2804 | follistatin isoform X1 [Anoplophora glabripennis] | 199 | 3 |
| Gene.281 | tropomyosin-1, isoforms 9A/A/B isoform X4 [Anoplophora glabripennis] | 350 | 3 |
| Gene.281 | tropomyosin-1, isoforms 9A/A/B isoform X4 [Anoplophora glabripennis] | 367 | 3 |
| Gene.281 | tropomyosin-1, isoforms 9A/A/B isoform X4 [Anoplophora glabripennis] | 409 | 3 |
| Gene.283 | sideroflexin-3 [Asbolus verrucosus] | 81 | 3 |
| Gene.283 | sideroflexin-3 [Asbolus verrucosus] | 95 | 3 |
| Gene.2830 | PREDICTED: LOW QUALITY PROTEIN: clathrin heavy chain [Aethina tumida] | 84 | 3 |
| Gene.2830 | PREDICTED: LOW QUALITY PROTEIN: clathrin heavy chain [Aethina tumida] | 103 | 3 |
| Gene.2830 | PREDICTED: LOW QUALITY PROTEIN: clathrin heavy chain [Aethina tumida] | 902 | 3 |
| Gene.2830 | PREDICTED: LOW QUALITY PROTEIN: clathrin heavy chain [Aethina tumida] | 1619 | 3 |
| Gene.2841 | ornithine aminotransferase, mitochondrial-like [Leptinotarsa decemlineata] | 88 | 3 |
| Gene.2841 | ornithine aminotransferase, mitochondrial-like [Leptinotarsa decemlineata] | 115 | 3 |
| Gene.2857 | C-1-tetrahydrofolate synthase, cytoplasmic [Onthophagus taurus] | 615 | 3 |
| Gene.2857 | C-1-tetrahydrofolate synthase, cytoplasmic [Onthophagus taurus] | 549 | 3 |
| Gene.2857 | C-1-tetrahydrofolate synthase, cytoplasmic [Onthophagus taurus] | 563 | 3 |
| Gene.2857 | C-1-tetrahydrofolate synthase, cytoplasmic [Onthophagus taurus] | 779 | 3 |
| Gene.288 | PREDICTED: isocitrate dehydrogenase [NAD] subunit beta, mitochondrial [Dendroctonus ponderosae] | 42 | 3 |
| Gene.288 | PREDICTED: isocitrate dehydrogenase [NAD] subunit beta, mitochondrial [Dendroctonus ponderosae] | 193 | 3 |
| Gene.2884 | PREDICTED: cathepsin L1 [Tribolium castaneum] | 100 | 3 |
| Gene.290 | takeout, partial [Asbolus verrucosus] | 47 | 3 |
| Gene.2913 | PREDICTED: glutaryl-CoA dehydrogenase, mitochondrial [Nicrophorus vespilloides] | 82 | 3 |
| Gene.2918 | plasminogen activator inhibitor 1 RNA-binding protein isoform X3 [Anoplophora glabripennis] | 57 | 3 |
| Gene.2918 | plasminogen activator inhibitor 1 RNA-binding protein isoform X3 [Anoplophora glabripennis] | 298 | 3 |
| Gene.2918 | plasminogen activator inhibitor 1 RNA-binding protein isoform X3 [Anoplophora glabripennis] | 45 | 3 |
| Gene.2918 | plasminogen activator inhibitor 1 RNA-binding protein isoform X3 [Anoplophora glabripennis] | 264 | 3 |
| Gene.2921 | oxygen-dependent coproporphyrinogen-III oxidase [Anoplophora glabripennis] | 100 | 3 |
| Gene.2933 | isocitrate dehydrogenase [NADP], mitochondrial-like [Onthophagus taurus] | 266 | 3 |
| Gene.2933 | isocitrate dehydrogenase [NADP], mitochondrial-like [Onthophagus taurus] | 401 | 3 |
| Gene.2933 | isocitrate dehydrogenase [NADP], mitochondrial-like [Onthophagus taurus] | 294 | 3 |
| Gene.2933 | isocitrate dehydrogenase [NADP], mitochondrial-like [Onthophagus taurus] | 197 | 3 |
| Gene.2933 | isocitrate dehydrogenase [NADP], mitochondrial-like [Onthophagus taurus] | 272 | 3 |
| Gene.2933 | isocitrate dehydrogenase [NADP], mitochondrial-like [Onthophagus taurus] | 39 | 3 |
| Gene.2933 | isocitrate dehydrogenase [NADP], mitochondrial-like [Onthophagus taurus] | 194 | 3 |
| Gene.2933 | isocitrate dehydrogenase [NADP], mitochondrial-like [Onthophagus taurus] | 183 | 3 |
| Gene.2933 | isocitrate dehydrogenase [NADP], mitochondrial-like [Onthophagus taurus] | 273 | 3 |
| Gene.2933 | isocitrate dehydrogenase [NADP], mitochondrial-like [Onthophagus taurus] | 98 | 3 |
| Gene.2933 | isocitrate dehydrogenase [NADP], mitochondrial-like [Onthophagus taurus] | 254 | 3 |
| Gene.2933 | isocitrate dehydrogenase [NADP], mitochondrial-like [Onthophagus taurus] | 172 | 3 |
| Gene.2944 | cytochrome P450 6FP7 [Propylea japonica] | 144 | 3 |
| Gene.2958 | alpha actinin [Coleomegilla maculata] | 401 | 3 |
| Gene.2958 | alpha actinin [Coleomegilla maculata] | 763 | 3 |
| Gene.2970 | apolipophorin, partial [Asbolus verrucosus] | 921 | 3 |
| Gene.298 | Csa-calmodulin 3, partial [Cupiennius salei] | 116 | 3 |
| Gene.298 | Csa-calmodulin 3, partial [Cupiennius salei] | 95 | 3 |
| Gene.298 | Csa-calmodulin 3, partial [Cupiennius salei] | 78 | 3 |
| Gene.298 | Csa-calmodulin 3, partial [Cupiennius salei] | 31 | 3 |
| Gene.2983 | coatomer subunit gamma [Asbolus verrucosus] | 80 | 3 |
| Gene.2983 | coatomer subunit gamma [Asbolus verrucosus] | 389 | 3 |
| Gene.2997 | PREDICTED: pyruvate kinase-like isoform X3 [Dendroctonus ponderosae] | 50 | 3 |
| Gene.2997 | PREDICTED: pyruvate kinase-like isoform X3 [Dendroctonus ponderosae] | 492 | 3 |
| Gene.2997 | PREDICTED: pyruvate kinase-like isoform X3 [Dendroctonus ponderosae] | 310 | 3 |
| Gene.2997 | PREDICTED: pyruvate kinase-like isoform X3 [Dendroctonus ponderosae] | 57 | 3 |
| Gene.2997 | PREDICTED: pyruvate kinase-like isoform X3 [Dendroctonus ponderosae] | 94 | 3 |
| Gene.2997 | PREDICTED: pyruvate kinase-like isoform X3 [Dendroctonus ponderosae] | 377 | 3 |
| Gene.2997 | PREDICTED: pyruvate kinase-like isoform X3 [Dendroctonus ponderosae] | 135 | 3 |
| Gene.2997 | PREDICTED: pyruvate kinase-like isoform X3 [Dendroctonus ponderosae] | 145 | 3 |
| Gene.2997 | PREDICTED: pyruvate kinase-like isoform X3 [Dendroctonus ponderosae] | 264 | 3 |
| Gene.2997 | PREDICTED: pyruvate kinase-like isoform X3 [Dendroctonus ponderosae] | 255 | 3 |
| Gene.2997 | PREDICTED: pyruvate kinase-like isoform X3 [Dendroctonus ponderosae] | 140 | 3 |
| Gene.2997 | PREDICTED: pyruvate kinase-like isoform X3 [Dendroctonus ponderosae] | 478 | 3 |
| Gene.3029 | PREDICTED: actin-interacting protein 1 isoform X2 [Tribolium castaneum] | 90 | 3 |
| Gene.305 | PREDICTED: elongation factor 1-alpha 1 isoform X2 [Ovis aries musimon] | 376 | 3 |
| Gene.3058 | PREDICTED: cleavage and polyadenylation specificity factor subunit CG7185 isoform X1 [Nicrophorus vespilloides] | 113 | 3 |
| Gene.3063 | PREDICTED: uncharacterized protein LOC108560181 [Nicrophorus vespilloides] | 178 | 3 |
| Gene.3069 | PREDICTED: glycine dehydrogenase (decarboxylating), mitochondrial [Tribolium castaneum] | 40 | 3 |
| Gene.3069 | PREDICTED: glycine dehydrogenase (decarboxylating), mitochondrial [Tribolium castaneum] | 107 | 3 |
| Gene.3075 | dihydrolipoyl dehydrogenase, mitochondrial [Anoplophora glabripennis] | 259 | 3 |
| Gene.3075 | dihydrolipoyl dehydrogenase, mitochondrial [Anoplophora glabripennis] | 118 | 3 |
| Gene.3075 | dihydrolipoyl dehydrogenase, mitochondrial [Anoplophora glabripennis] | 162 | 3 |
| Gene.3075 | dihydrolipoyl dehydrogenase, mitochondrial [Anoplophora glabripennis] | 139 | 3 |
| Gene.3075 | dihydrolipoyl dehydrogenase, mitochondrial [Anoplophora glabripennis] | 273 | 3 |
| Gene.3076 | PREDICTED: inositol-3-phosphate synthase [Tribolium castaneum] | 302 | 3 |
| Gene.308 | PREDICTED: 3-ketoacyl-CoA thiolase, mitochondrial [Tribolium castaneum] | 6 | 3 |
| Gene.308 | PREDICTED: 3-ketoacyl-CoA thiolase, mitochondrial [Tribolium castaneum] | 27 | 3 |
| Gene.308 | PREDICTED: 3-ketoacyl-CoA thiolase, mitochondrial [Tribolium castaneum] | 276 | 3 |
| Gene.308 | PREDICTED: 3-ketoacyl-CoA thiolase, mitochondrial [Tribolium castaneum] | 342 | 3 |
| Gene.308 | PREDICTED: 3-ketoacyl-CoA thiolase, mitochondrial [Tribolium castaneum] | 193 | 3 |
| Gene.308 | PREDICTED: 3-ketoacyl-CoA thiolase, mitochondrial [Tribolium castaneum] | 78 | 3 |
| Gene.308 | PREDICTED: 3-ketoacyl-CoA thiolase, mitochondrial [Tribolium castaneum] | 202 | 3 |
| Gene.308 | PREDICTED: 3-ketoacyl-CoA thiolase, mitochondrial [Tribolium castaneum] | 14 | 3 |
| Gene.308 | PREDICTED: 3-ketoacyl-CoA thiolase, mitochondrial [Tribolium castaneum] | 211 | 3 |
| Gene.308 | PREDICTED: 3-ketoacyl-CoA thiolase, mitochondrial [Tribolium castaneum] | 174 | 3 |
| Gene.308 | PREDICTED: 3-ketoacyl-CoA thiolase, mitochondrial [Tribolium castaneum] | 337 | 3 |
| Gene.308 | PREDICTED: 3-ketoacyl-CoA thiolase, mitochondrial [Tribolium castaneum] | 169 | 3 |
| Gene.310 | 60S ribosomal protein L10a [Monochamus alternatus] | 130 | 3 |
| Gene.310 | 60S ribosomal protein L10a [Monochamus alternatus] | 207 | 3 |
| Gene.310 | 60S ribosomal protein L10a [Monochamus alternatus] | 106 | 3 |
| Gene.310 | 60S ribosomal protein L10a [Monochamus alternatus] | 156 | 3 |
| Gene.3155 | PREDICTED: putative epidermal cell surface receptor isoform X3 [Aethina tumida] | 197 | 3 |
| Gene.316 | heat shock 70 kDa protein cognate 5-like protein [Epicauta chinensis] | 127 | 3 |
| Gene.316 | heat shock 70 kDa protein cognate 5-like protein [Epicauta chinensis] | 412 | 3 |
| Gene.316 | heat shock 70 kDa protein cognate 5-like protein [Epicauta chinensis] | 612 | 3 |
| Gene.316 | heat shock 70 kDa protein cognate 5-like protein [Epicauta chinensis] | 241 | 3 |
| Gene.316 | heat shock 70 kDa protein cognate 5-like protein [Epicauta chinensis] | 632 | 3 |
| Gene.316 | heat shock 70 kDa protein cognate 5-like protein [Epicauta chinensis] | 607 | 3 |
| Gene.316 | heat shock 70 kDa protein cognate 5-like protein [Epicauta chinensis] | 307 | 3 |
| Gene.316 | heat shock 70 kDa protein cognate 5-like protein [Epicauta chinensis] | 617 | 3 |
| Gene.316 | heat shock 70 kDa protein cognate 5-like protein [Epicauta chinensis] | 677 | 3 |
| Gene.316 | heat shock 70 kDa protein cognate 5-like protein [Epicauta chinensis] | 352 | 3 |
| Gene.316 | heat shock 70 kDa protein cognate 5-like protein [Epicauta chinensis] | 144 | 3 |
| Gene.316 | heat shock 70 kDa protein cognate 5-like protein [Epicauta chinensis] | 378 | 3 |
| Gene.316 | heat shock 70 kDa protein cognate 5-like protein [Epicauta chinensis] | 82 | 3 |
| Gene.316 | heat shock 70 kDa protein cognate 5-like protein [Epicauta chinensis] | 149 | 3 |
| Gene.316 | heat shock 70 kDa protein cognate 5-like protein [Epicauta chinensis] | 182 | 3 |
| Gene.316 | heat shock 70 kDa protein cognate 5-like protein [Epicauta chinensis] | 367 | 3 |
| Gene.316 | heat shock 70 kDa protein cognate 5-like protein [Epicauta chinensis] | 619 | 3 |
| Gene.316 | heat shock 70 kDa protein cognate 5-like protein [Epicauta chinensis] | 140 | 3 |
| Gene.3177 | Hemocyanin C and/or Tyrosinase domain containing protein [Asbolus verrucosus] | 33 | 3 |
| Gene.3177 | Hemocyanin C and/or Tyrosinase domain containing protein [Asbolus verrucosus] | 280 | 3 |
| Gene.3186 | facilitated trehalose transporter Tret1-like isoform X2 [Anoplophora glabripennis] | 345 | 3 |
| Gene.3186 | facilitated trehalose transporter Tret1-like isoform X2 [Anoplophora glabripennis] | 315 | 3 |
| Gene.3186 | facilitated trehalose transporter Tret1-like isoform X2 [Anoplophora glabripennis] | 571 | 3 |
| Gene.3186 | facilitated trehalose transporter Tret1-like isoform X2 [Anoplophora glabripennis] | 558 | 3 |
| Gene.3191 | PREDICTED: uncharacterized protein LOC108566679 isoform X1 [Nicrophorus vespilloides] | 200 | 3 |
| Gene.3208 | phosphoglucose isomerase [Colias eurytheme] | 28 | 3 |
| Gene.3208 | phosphoglucose isomerase [Colias eurytheme] | 462 | 3 |
| Gene.3208 | phosphoglucose isomerase [Colias eurytheme] | 41 | 3 |
| Gene.3218 | galactokinase-like [Agrilus planipennis] | 185 | 3 |
| Gene.3223 | Aldose reductase-like Protein [Tribolium castaneum] | 72 | 3 |
| Gene.3223 | Aldose reductase-like Protein [Tribolium castaneum] | 264 | 3 |
| Gene.3223 | Aldose reductase-like Protein [Tribolium castaneum] | 167 | 3 |
| Gene.3223 | Aldose reductase-like Protein [Tribolium castaneum] | 202 | 3 |
| Gene.3232 | PREDICTED: transketolase-like protein 2 isoform X1 [Tribolium castaneum] | 21 | 3 |
| Gene.3232 | PREDICTED: transketolase-like protein 2 isoform X1 [Tribolium castaneum] | 546 | 3 |
| Gene.3232 | PREDICTED: transketolase-like protein 2 isoform X1 [Tribolium castaneum] | 102 | 3 |
| Gene.3232 | PREDICTED: transketolase-like protein 2 isoform X1 [Tribolium castaneum] | 347 | 3 |
| Gene.3232 | PREDICTED: transketolase-like protein 2 isoform X1 [Tribolium castaneum] | 314 | 3 |
| Gene.3232 | PREDICTED: transketolase-like protein 2 isoform X1 [Tribolium castaneum] | 331 | 3 |
| Gene.3232 | PREDICTED: transketolase-like protein 2 isoform X1 [Tribolium castaneum] | 469 | 3 |
| Gene.3232 | PREDICTED: transketolase-like protein 2 isoform X1 [Tribolium castaneum] | 495 | 3 |
| Gene.3232 | PREDICTED: transketolase-like protein 2 isoform X1 [Tribolium castaneum] | 244 | 3 |
| Gene.3232 | PREDICTED: transketolase-like protein 2 isoform X1 [Tribolium castaneum] | 144 | 3 |
| Gene.3232 | PREDICTED: transketolase-like protein 2 isoform X1 [Tribolium castaneum] | 16 | 3 |
| Gene.3232 | PREDICTED: transketolase-like protein 2 isoform X1 [Tribolium castaneum] | 235 | 3 |
| Gene.3232 | PREDICTED: transketolase-like protein 2 isoform X1 [Tribolium castaneum] | 6 | 3 |
| Gene.3232 | PREDICTED: transketolase-like protein 2 isoform X1 [Tribolium castaneum] | 334 | 3 |
| Gene.3232 | PREDICTED: transketolase-like protein 2 isoform X1 [Tribolium castaneum] | 542 | 3 |
| Gene.3232 | PREDICTED: transketolase-like protein 2 isoform X1 [Tribolium castaneum] | 253 | 3 |
| Gene.3232 | PREDICTED: transketolase-like protein 2 isoform X1 [Tribolium castaneum] | 589 | 3 |
| Gene.3232 | PREDICTED: transketolase-like protein 2 isoform X1 [Tribolium castaneum] | 614 | 3 |
| Gene.3232 | PREDICTED: transketolase-like protein 2 isoform X1 [Tribolium castaneum] | 59 | 3 |
| Gene.3232 | PREDICTED: transketolase-like protein 2 isoform X1 [Tribolium castaneum] | 10 | 3 |
| Gene.3243 | cytochrome P450 monooxygenase [Tribolium castaneum] | 276 | 3 |
| Gene.3247 | PREDICTED: phosphoenolpyruvate carboxykinase [GTP] isoform X1 [Tribolium castaneum] | 93 | 3 |
| Gene.3252 | chitooligosaccharidolytic beta-N-acetylglucosaminidase isoform X2 [Anoplophora glabripennis] | 101 | 3 |
| Gene.3273 | uncharacterized protein BDFB_009872 [Asbolus verrucosus] | 171 | 3 |
| Gene.3292 | Esterase-6-like Protein [Tribolium castaneum] | 376 | 3 |
| Gene.3292 | Esterase-6-like Protein [Tribolium castaneum] | 485 | 3 |
| Gene.331 | PREDICTED: ferritin subunit [Aethina tumida] | 206 | 3 |
| Gene.331 | PREDICTED: ferritin subunit [Aethina tumida] | 185 | 3 |
| Gene.3339 | testis-expressed protein 2 isoform X2 [Anoplophora glabripennis] | 465 | 3 |
| Gene.3344 | Proteasome domain containing protein [Asbolus verrucosus] | 198 | 3 |
| Gene.3358 | putative 60S acidic ribosomal protein P1, partial [Cotesia chilonis] | 57 | 3 |
| Gene.3368 | PREDICTED: cytochrome P450 4C1 [Tribolium castaneum] | 69 | 3 |
| Gene.3368 | PREDICTED: cytochrome P450 4C1 [Tribolium castaneum] | 251 | 3 |
| Gene.3368 | PREDICTED: cytochrome P450 4C1 [Tribolium castaneum] | 455 | 3 |
| Gene.3370 | PREDICTED: probable NADH dehydrogenase [ubiquinone] 1 alpha subcomplex subunit 12 [Tribolium castaneum] | 40 | 3 |
| Gene.3370 | PREDICTED: probable NADH dehydrogenase [ubiquinone] 1 alpha subcomplex subunit 12 [Tribolium castaneum] | 48 | 3 |
| Gene.3373 | PREDICTED: uncharacterized protein LOC109597117 [Aethina tumida] | 80 | 3 |
| Gene.3385 | hypothetical protein BDFB_003224 [Asbolus verrucosus] | 44 | 3 |
| Gene.3401 | glutathione S-transferase-like [Anoplophora glabripennis] | 74 | 3 |
| Gene.3401 | glutathione S-transferase-like [Anoplophora glabripennis] | 105 | 3 |
| Gene.3401 | glutathione S-transferase-like [Anoplophora glabripennis] | 113 | 3 |
| Gene.342 | PREDICTED: 40S ribosomal protein S9 [Tribolium castaneum] | 67 | 3 |
| Gene.342 | PREDICTED: 40S ribosomal protein S9 [Tribolium castaneum] | 70 | 3 |
| Gene.342 | PREDICTED: 40S ribosomal protein S9 [Tribolium castaneum] | 94 | 3 |
| Gene.342 | PREDICTED: 40S ribosomal protein S9 [Tribolium castaneum] | 117 | 3 |
| Gene.342 | PREDICTED: 40S ribosomal protein S9 [Tribolium castaneum] | 48 | 3 |
| Gene.3421 | PREDICTED: 4-coumarate--CoA ligase 1 [Tribolium castaneum] | 536 | 3 |
| Gene.3421 | PREDICTED: 4-coumarate--CoA ligase 1 [Tribolium castaneum] | 541 | 3 |
| Gene.3424 | sugar transporter 11 [Tenebrio molitor] | 220 | 3 |
| Gene.3441 | PREDICTED: 23 kDa integral membrane protein-like [Aethina tumida] | 124 | 3 |
| Gene.3441 | PREDICTED: 23 kDa integral membrane protein-like [Aethina tumida] | 115 | 3 |
| Gene.3447 | PREDICTED: congested-like trachea protein [Tribolium castaneum] | 163 | 3 |
| Gene.3447 | PREDICTED: congested-like trachea protein [Tribolium castaneum] | 72 | 3 |
| Gene.3447 | PREDICTED: congested-like trachea protein [Tribolium castaneum] | 167 | 3 |
| Gene.3447 | PREDICTED: congested-like trachea protein [Tribolium castaneum] | 258 | 3 |
| Gene.3459 | motile sperm domain-containing protein 2-like [Anoplophora glabripennis] | 324 | 3 |
| Gene.3460 | PREDICTED: LOW QUALITY PROTEIN: papilin [Tribolium castaneum] | 1629 | 3 |
| Gene.3475 | PREDICTED: thioredoxin, mitochondrial-like [Aethina tumida] | 40 | 3 |
| Gene.3475 | PREDICTED: thioredoxin, mitochondrial-like [Aethina tumida] | 30 | 3 |
| Gene.3475 | PREDICTED: thioredoxin, mitochondrial-like [Aethina tumida] | 73 | 3 |
| Gene.3475 | PREDICTED: thioredoxin, mitochondrial-like [Aethina tumida] | 82 | 3 |
| Gene.3481 | NADH dehydrogenase [ubiquinone] 1 alpha subcomplex subunit 8 [Asbolus verrucosus] | 53 | 3 |
| Gene.3482 | V-type proton ATPase catalytic subunit A [Leptinotarsa decemlineata] | 588 | 3 |
| Gene.3482 | V-type proton ATPase catalytic subunit A [Leptinotarsa decemlineata] | 130 | 3 |
| Gene.3482 | V-type proton ATPase catalytic subunit A [Leptinotarsa decemlineata] | 533 | 3 |
| Gene.3482 | V-type proton ATPase catalytic subunit A [Leptinotarsa decemlineata] | 593 | 3 |
| Gene.3482 | V-type proton ATPase catalytic subunit A [Leptinotarsa decemlineata] | 584 | 3 |
| Gene.3482 | V-type proton ATPase catalytic subunit A [Leptinotarsa decemlineata] | 513 | 3 |
| Gene.3489 | PREDICTED: acetyl-CoA carboxylase isoform X1 [Tribolium castaneum] | 2124 | 3 |
| Gene.3489 | PREDICTED: acetyl-CoA carboxylase isoform X1 [Tribolium castaneum] | 2067 | 3 |
| Gene.3489 | PREDICTED: acetyl-CoA carboxylase isoform X1 [Tribolium castaneum] | 387 | 3 |
| Gene.3489 | PREDICTED: acetyl-CoA carboxylase isoform X1 [Tribolium castaneum] | 1848 | 3 |
| Gene.3489 | PREDICTED: acetyl-CoA carboxylase isoform X1 [Tribolium castaneum] | 2305 | 3 |
| Gene.3489 | PREDICTED: acetyl-CoA carboxylase isoform X1 [Tribolium castaneum] | 1964 | 3 |
| Gene.3489 | PREDICTED: acetyl-CoA carboxylase isoform X1 [Tribolium castaneum] | 167 | 3 |
| Gene.3489 | PREDICTED: acetyl-CoA carboxylase isoform X1 [Tribolium castaneum] | 341 | 3 |
| Gene.3489 | PREDICTED: acetyl-CoA carboxylase isoform X1 [Tribolium castaneum] | 1645 | 3 |
| Gene.3489 | PREDICTED: acetyl-CoA carboxylase isoform X1 [Tribolium castaneum] | 1389 | 3 |
| Gene.3489 | PREDICTED: acetyl-CoA carboxylase isoform X1 [Tribolium castaneum] | 2131 | 3 |
| Gene.3489 | PREDICTED: acetyl-CoA carboxylase isoform X1 [Tribolium castaneum] | 179 | 3 |
| Gene.3489 | PREDICTED: acetyl-CoA carboxylase isoform X1 [Tribolium castaneum] | 1820 | 3 |
| Gene.3489 | PREDICTED: acetyl-CoA carboxylase isoform X1 [Tribolium castaneum] | 383 | 3 |
| Gene.3489 | PREDICTED: acetyl-CoA carboxylase isoform X1 [Tribolium castaneum] | 1264 | 3 |
| Gene.3489 | PREDICTED: acetyl-CoA carboxylase isoform X1 [Tribolium castaneum] | 1412 | 3 |
| Gene.3489 | PREDICTED: acetyl-CoA carboxylase isoform X1 [Tribolium castaneum] | 2235 | 3 |
| Gene.3489 | PREDICTED: acetyl-CoA carboxylase isoform X1 [Tribolium castaneum] | 746 | 3 |
| Gene.3489 | PREDICTED: acetyl-CoA carboxylase isoform X1 [Tribolium castaneum] | 2342 | 3 |
| Gene.3489 | PREDICTED: acetyl-CoA carboxylase isoform X1 [Tribolium castaneum] | 1379 | 3 |
| Gene.3489 | PREDICTED: acetyl-CoA carboxylase isoform X1 [Tribolium castaneum] | 1369 | 3 |
| Gene.3489 | PREDICTED: acetyl-CoA carboxylase isoform X1 [Tribolium castaneum] | 2245 | 3 |
| Gene.3498 | 40S ribosomal protein S8 [Anoplophora glabripennis] | 128 | 3 |
| Gene.3498 | 40S ribosomal protein S8 [Anoplophora glabripennis] | 200 | 3 |
| Gene.3498 | 40S ribosomal protein S8 [Anoplophora glabripennis] | 37 | 3 |
| Gene.3498 | 40S ribosomal protein S8 [Anoplophora glabripennis] | 139 | 3 |
| Gene.3498 | 40S ribosomal protein S8 [Anoplophora glabripennis] | 54 | 3 |
| Gene.3500 | sphingosine-1-phosphate lyase [Asbolus verrucosus] | 152 | 3 |
| Gene.3500 | sphingosine-1-phosphate lyase [Asbolus verrucosus] | 415 | 3 |
| Gene.3504 | cytochrome P450 monooxygenase CYP6BQ37 [Tenebrio molitor] | 174 | 3 |
| Gene.3504 | cytochrome P450 monooxygenase CYP6BQ37 [Tenebrio molitor] | 144 | 3 |
| Gene.3511 | cystathionine gamma-lyase-like [Anoplophora glabripennis] | 158 | 3 |
| Gene.3511 | cystathionine gamma-lyase-like [Anoplophora glabripennis] | 57 | 3 |
| Gene.3511 | cystathionine gamma-lyase-like [Anoplophora glabripennis] | 82 | 3 |
| Gene.3518 | vitellogenin 2 [Harmonia axyridis] | 718 | 3 |
| Gene.3518 | vitellogenin 2 [Harmonia axyridis] | 1299 | 3 |
| Gene.3518 | vitellogenin 2 [Harmonia axyridis] | 612 | 3 |
| Gene.3518 | vitellogenin 2 [Harmonia axyridis] | 839 | 3 |
| Gene.3518 | vitellogenin 2 [Harmonia axyridis] | 845 | 3 |
| Gene.3518 | vitellogenin 2 [Harmonia axyridis] | 1420 | 3 |
| Gene.3518 | vitellogenin 2 [Harmonia axyridis] | 764 | 3 |
| Gene.3518 | vitellogenin 2 [Harmonia axyridis] | 513 | 3 |
| Gene.3518 | vitellogenin 2 [Harmonia axyridis] | 1601 | 3 |
| Gene.3518 | vitellogenin 2 [Harmonia axyridis] | 1354 | 3 |
| Gene.3518 | vitellogenin 2 [Harmonia axyridis] | 1445 | 3 |
| Gene.3518 | vitellogenin 2 [Harmonia axyridis] | 1172 | 3 |
| Gene.3518 | vitellogenin 2 [Harmonia axyridis] | 1037 | 3 |
| Gene.3518 | vitellogenin 2 [Harmonia axyridis] | 243 | 3 |
| Gene.3518 | vitellogenin 2 [Harmonia axyridis] | 348 | 3 |
| Gene.3518 | vitellogenin 2 [Harmonia axyridis] | 1106 | 3 |
| Gene.3518 | vitellogenin 2 [Harmonia axyridis] | 477 | 3 |
| Gene.3518 | vitellogenin 2 [Harmonia axyridis] | 1131 | 3 |
| Gene.3522 | vitellogenin 1 [Harmonia axyridis] | 271 | 3 |
| Gene.354 | saccharopine dehydrogenase-like [Asbolus verrucosus] | 231 | 3 |
| Gene.354 | saccharopine dehydrogenase-like [Asbolus verrucosus] | 265 | 3 |
| Gene.354 | saccharopine dehydrogenase-like [Asbolus verrucosus] | 23 | 3 |
| Gene.354 | saccharopine dehydrogenase-like [Asbolus verrucosus] | 346 | 3 |
| Gene.354 | saccharopine dehydrogenase-like [Asbolus verrucosus] | 331 | 3 |
| Gene.354 | saccharopine dehydrogenase-like [Asbolus verrucosus] | 355 | 3 |
| Gene.3547 | dolichyl-diphosphooligosaccharide--protein glycosyltransferase subunit 2 [Anoplophora glabripennis] | 158 | 3 |
| Gene.3547 | dolichyl-diphosphooligosaccharide--protein glycosyltransferase subunit 2 [Anoplophora glabripennis] | 495 | 3 |
| Gene.3547 | dolichyl-diphosphooligosaccharide--protein glycosyltransferase subunit 2 [Anoplophora glabripennis] | 289 | 3 |
| Gene.3547 | dolichyl-diphosphooligosaccharide--protein glycosyltransferase subunit 2 [Anoplophora glabripennis] | 125 | 3 |
| Gene.3547 | dolichyl-diphosphooligosaccharide--protein glycosyltransferase subunit 2 [Anoplophora glabripennis] | 144 | 3 |
| Gene.3547 | dolichyl-diphosphooligosaccharide--protein glycosyltransferase subunit 2 [Anoplophora glabripennis] | 390 | 3 |
| Gene.3548 | E3 SUMO-protein ligase RanBP2 [Anoplophora glabripennis] | 1106 | 3 |
| Gene.3558 | PREDICTED: probable aconitate hydratase, mitochondrial [Tribolium castaneum] | 697 | 3 |
| Gene.3558 | PREDICTED: probable aconitate hydratase, mitochondrial [Tribolium castaneum] | 519 | 3 |
| Gene.3558 | PREDICTED: probable aconitate hydratase, mitochondrial [Tribolium castaneum] | 63 | 3 |
| Gene.3558 | PREDICTED: probable aconitate hydratase, mitochondrial [Tribolium castaneum] | 328 | 3 |
| Gene.3558 | PREDICTED: probable aconitate hydratase, mitochondrial [Tribolium castaneum] | 523 | 3 |
| Gene.3558 | PREDICTED: probable aconitate hydratase, mitochondrial [Tribolium castaneum] | 331 | 3 |
| Gene.3558 | PREDICTED: probable aconitate hydratase, mitochondrial [Tribolium castaneum] | 315 | 3 |
| Gene.3558 | PREDICTED: probable aconitate hydratase, mitochondrial [Tribolium castaneum] | 244 | 3 |
| Gene.3558 | PREDICTED: probable aconitate hydratase, mitochondrial [Tribolium castaneum] | 419 | 3 |
| Gene.3558 | PREDICTED: probable aconitate hydratase, mitochondrial [Tribolium castaneum] | 708 | 3 |
| Gene.3558 | PREDICTED: probable aconitate hydratase, mitochondrial [Tribolium castaneum] | 44 | 3 |
| Gene.3558 | PREDICTED: probable aconitate hydratase, mitochondrial [Tribolium castaneum] | 725 | 3 |
| Gene.3558 | PREDICTED: probable aconitate hydratase, mitochondrial [Tribolium castaneum] | 599 | 3 |
| Gene.3558 | PREDICTED: probable aconitate hydratase, mitochondrial [Tribolium castaneum] | 235 | 3 |
| Gene.3558 | PREDICTED: probable aconitate hydratase, mitochondrial [Tribolium castaneum] | 240 | 3 |
| Gene.3558 | PREDICTED: probable aconitate hydratase, mitochondrial [Tribolium castaneum] | 252 | 3 |
| Gene.3558 | PREDICTED: probable aconitate hydratase, mitochondrial [Tribolium castaneum] | 85 | 3 |
| Gene.3558 | PREDICTED: probable aconitate hydratase, mitochondrial [Tribolium castaneum] | 569 | 3 |
| Gene.3558 | PREDICTED: probable aconitate hydratase, mitochondrial [Tribolium castaneum] | 531 | 3 |
| Gene.3558 | PREDICTED: probable aconitate hydratase, mitochondrial [Tribolium castaneum] | 417 | 3 |
| Gene.3558 | PREDICTED: probable aconitate hydratase, mitochondrial [Tribolium castaneum] | 731 | 3 |
| Gene.3558 | PREDICTED: probable aconitate hydratase, mitochondrial [Tribolium castaneum] | 377 | 3 |
| Gene.3558 | PREDICTED: probable aconitate hydratase, mitochondrial [Tribolium castaneum] | 47 | 3 |
| Gene.3558 | PREDICTED: probable aconitate hydratase, mitochondrial [Tribolium castaneum] | 54 | 3 |
| Gene.3583 | 4-coumarate--CoA ligase-like [Leptinotarsa decemlineata] | 440 | 3 |
| Gene.3583 | 4-coumarate--CoA ligase-like [Leptinotarsa decemlineata] | 412 | 3 |
| Gene.3583 | 4-coumarate--CoA ligase-like [Leptinotarsa decemlineata] | 326 | 3 |
| Gene.3583 | 4-coumarate--CoA ligase-like [Leptinotarsa decemlineata] | 381 | 3 |
| Gene.3583 | 4-coumarate--CoA ligase-like [Leptinotarsa decemlineata] | 583 | 3 |
| Gene.3583 | 4-coumarate--CoA ligase-like [Leptinotarsa decemlineata] | 550 | 3 |
| Gene.3601 | PREDICTED: sulfotransferase 1A1 [Nicrophorus vespilloides] | 259 | 3 |
| Gene.3601 | PREDICTED: sulfotransferase 1A1 [Nicrophorus vespilloides] | 118 | 3 |
| Gene.3601 | PREDICTED: sulfotransferase 1A1 [Nicrophorus vespilloides] | 37 | 3 |
| Gene.3601 | PREDICTED: sulfotransferase 1A1 [Nicrophorus vespilloides] | 292 | 3 |
| Gene.3601 | PREDICTED: sulfotransferase 1A1 [Nicrophorus vespilloides] | 318 | 3 |
| Gene.3623 | PREDICTED: uncharacterized protein LOC109539019 [Dendroctonus ponderosae] | 51 | 3 |
| Gene.3651 | uncharacterized protein LOC108907894 isoform X4 [Anoplophora glabripennis] | 170 | 3 |
| Gene.3651 | uncharacterized protein LOC108907894 isoform X4 [Anoplophora glabripennis] | 139 | 3 |
| Gene.3651 | uncharacterized protein LOC108907894 isoform X4 [Anoplophora glabripennis] | 122 | 3 |
| Gene.3651 | uncharacterized protein LOC108907894 isoform X4 [Anoplophora glabripennis] | 114 | 3 |
| Gene.3656 | PREDICTED: prisilkin-39 [Tribolium castaneum] | 35 | 3 |
| Gene.3656 | PREDICTED: prisilkin-39 [Tribolium castaneum] | 21 | 3 |
| Gene.3656 | PREDICTED: prisilkin-39 [Tribolium castaneum] | 174 | 3 |
| Gene.3656 | PREDICTED: prisilkin-39 [Tribolium castaneum] | 165 | 3 |
| Gene.3656 | PREDICTED: prisilkin-39 [Tribolium castaneum] | 13 | 3 |
| Gene.3656 | PREDICTED: prisilkin-39 [Tribolium castaneum] | 313 | 3 |
| Gene.3663 | PREDICTED: uncharacterized protein LOC659539 isoform X1 [Tribolium castaneum] | 206 | 3 |
| Gene.3676 | hypothetical protein D910_04355 [Dendroctonus ponderosae] | 39 | 3 |
| Gene.3676 | hypothetical protein D910_04355 [Dendroctonus ponderosae] | 225 | 3 |
| Gene.3676 | hypothetical protein D910_04355 [Dendroctonus ponderosae] | 118 | 3 |
| Gene.3676 | hypothetical protein D910_04355 [Dendroctonus ponderosae] | 287 | 3 |
| Gene.3676 | hypothetical protein D910_04355 [Dendroctonus ponderosae] | 266 | 3 |
| Gene.3676 | hypothetical protein D910_04355 [Dendroctonus ponderosae] | 263 | 3 |
| Gene.3676 | hypothetical protein D910_04355 [Dendroctonus ponderosae] | 231 | 3 |
| Gene.3676 | hypothetical protein D910_04355 [Dendroctonus ponderosae] | 125 | 3 |
| Gene.3676 | hypothetical protein D910_04355 [Dendroctonus ponderosae] | 157 | 3 |
| Gene.3698 | Uncharacterized protein | 64 | 3 |
| Gene.3708 | phosphate carrier protein, mitochondrial-like [Leptinotarsa decemlineata] | 289 | 3 |
| Gene.3708 | phosphate carrier protein, mitochondrial-like [Leptinotarsa decemlineata] | 102 | 3 |
| Gene.3708 | phosphate carrier protein, mitochondrial-like [Leptinotarsa decemlineata] | 307 | 3 |
| Gene.3708 | phosphate carrier protein, mitochondrial-like [Leptinotarsa decemlineata] | 299 | 3 |
| Gene.3709 | 60S ribosomal protein L27 [Anoplophora glabripennis] | 27 | 3 |
| Gene.3709 | 60S ribosomal protein L27 [Anoplophora glabripennis] | 98 | 3 |
| Gene.3709 | 60S ribosomal protein L27 [Anoplophora glabripennis] | 127 | 3 |
| Gene.3709 | 60S ribosomal protein L27 [Anoplophora glabripennis] | 132 | 3 |
| Gene.3709 | 60S ribosomal protein L27 [Anoplophora glabripennis] | 101 | 3 |
| Gene.3709 | 60S ribosomal protein L27 [Anoplophora glabripennis] | 73 | 3 |
| Gene.3715 | tropomyosin-1, isoforms 33/34 isoform X1 [Leptinotarsa decemlineata] | 128 | 3 |
| Gene.3715 | tropomyosin-1, isoforms 33/34 isoform X1 [Leptinotarsa decemlineata] | 124 | 3 |
| Gene.3715 | tropomyosin-1, isoforms 33/34 isoform X1 [Leptinotarsa decemlineata] | 113 | 3 |
| Gene.3721 | PREDICTED: glutamine synthetase-like [Aethina tumida] | 212 | 3 |
| Gene.3721 | PREDICTED: glutamine synthetase-like [Aethina tumida] | 39 | 3 |
| Gene.3721 | PREDICTED: glutamine synthetase-like [Aethina tumida] | 227 | 3 |
| Gene.3721 | PREDICTED: glutamine synthetase-like [Aethina tumida] | 208 | 3 |
| Gene.3721 | PREDICTED: glutamine synthetase-like [Aethina tumida] | 195 | 3 |
| Gene.3730 | PREDICTED: protein amalgam isoform X1 [Tribolium castaneum] | 382 | 3 |
| Gene.3730 | PREDICTED: protein amalgam isoform X1 [Tribolium castaneum] | 422 | 3 |
| Gene.3731 | PREDICTED: UDP-glucuronosyltransferase 2B2 [Tribolium castaneum] | 221 | 3 |
| Gene.3734 | PREDICTED: UDP-glucuronosyltransferase 2B7 isoform X1 [Tribolium castaneum] | 395 | 3 |
| Gene.3734 | PREDICTED: UDP-glucuronosyltransferase 2B7 isoform X1 [Tribolium castaneum] | 400 | 3 |
| Gene.3734 | PREDICTED: UDP-glucuronosyltransferase 2B7 isoform X1 [Tribolium castaneum] | 305 | 3 |
| Gene.3734 | PREDICTED: UDP-glucuronosyltransferase 2B7 isoform X1 [Tribolium castaneum] | 406 | 3 |
| Gene.3734 | PREDICTED: UDP-glucuronosyltransferase 2B7 isoform X1 [Tribolium castaneum] | 378 | 3 |
| Gene.3736 | PREDICTED: UDP-glucose 4-epimerase isoform X2 [Dendroctonus ponderosae] | 240 | 3 |
| Gene.3736 | PREDICTED: UDP-glucose 4-epimerase isoform X2 [Dendroctonus ponderosae] | 76 | 3 |
| Gene.3736 | PREDICTED: UDP-glucose 4-epimerase isoform X2 [Dendroctonus ponderosae] | 295 | 3 |
| Gene.3747 | piwi-like protein Ago3 [Anoplophora glabripennis] | 377 | 3 |
| Gene.3753 | PREDICTED: uncharacterized protein C15orf61 [Dendroctonus ponderosae] | 8 | 3 |
| Gene.3753 | PREDICTED: uncharacterized protein C15orf61 [Dendroctonus ponderosae] | 69 | 3 |
| Gene.3753 | PREDICTED: uncharacterized protein C15orf61 [Dendroctonus ponderosae] | 99 | 3 |
| Gene.3753 | PREDICTED: uncharacterized protein C15orf61 [Dendroctonus ponderosae] | 95 | 3 |
| Gene.3753 | PREDICTED: uncharacterized protein C15orf61 [Dendroctonus ponderosae] | 122 | 3 |
| Gene.3753 | PREDICTED: uncharacterized protein C15orf61 [Dendroctonus ponderosae] | 62 | 3 |
| Gene.3753 | PREDICTED: uncharacterized protein C15orf61 [Dendroctonus ponderosae] | 79 | 3 |
| Gene.3776 | PREDICTED: scaffold attachment factor B1 isoform X1 [Tribolium castaneum] | 272 | 3 |
| Gene.3776 | PREDICTED: scaffold attachment factor B1 isoform X1 [Tribolium castaneum] | 400 | 3 |
| Gene.3790 | PREDICTED: 60S ribosomal protein L3 [Tribolium castaneum] | 124 | 3 |
| Gene.3790 | PREDICTED: 60S ribosomal protein L3 [Tribolium castaneum] | 233 | 3 |
| Gene.3790 | PREDICTED: 60S ribosomal protein L3 [Tribolium castaneum] | 370 | 3 |
| Gene.3790 | PREDICTED: 60S ribosomal protein L3 [Tribolium castaneum] | 298 | 3 |
| Gene.3790 | PREDICTED: 60S ribosomal protein L3 [Tribolium castaneum] | 360 | 3 |
| Gene.3790 | PREDICTED: 60S ribosomal protein L3 [Tribolium castaneum] | 173 | 3 |
| Gene.3790 | PREDICTED: 60S ribosomal protein L3 [Tribolium castaneum] | 136 | 3 |
| Gene.3790 | PREDICTED: 60S ribosomal protein L3 [Tribolium castaneum] | 140 | 3 |
| Gene.3793 | maltase 2, partial [Asbolus verrucosus] | 109 | 3 |
| Gene.3795 | NADH-ubiquinone oxidoreductase 75 kDa subunit, mitochondrial [Asbolus verrucosus] | 307 | 3 |
| Gene.3795 | NADH-ubiquinone oxidoreductase 75 kDa subunit, mitochondrial [Asbolus verrucosus] | 626 | 3 |
| Gene.3795 | NADH-ubiquinone oxidoreductase 75 kDa subunit, mitochondrial [Asbolus verrucosus] | 622 | 3 |
| Gene.3795 | NADH-ubiquinone oxidoreductase 75 kDa subunit, mitochondrial [Asbolus verrucosus] | 172 | 3 |
| Gene.3795 | NADH-ubiquinone oxidoreductase 75 kDa subunit, mitochondrial [Asbolus verrucosus] | 166 | 3 |
| Gene.3795 | NADH-ubiquinone oxidoreductase 75 kDa subunit, mitochondrial [Asbolus verrucosus] | 691 | 3 |
| Gene.3795 | NADH-ubiquinone oxidoreductase 75 kDa subunit, mitochondrial [Asbolus verrucosus] | 541 | 3 |
| Gene.3807 | scavenger receptor protein [Tribolium castaneum] | 269 | 3 |
| Gene.3807 | scavenger receptor protein [Tribolium castaneum] | 180 | 3 |
| Gene.3807 | scavenger receptor protein [Tribolium castaneum] | 544 | 3 |
| Gene.3807 | scavenger receptor protein [Tribolium castaneum] | 344 | 3 |
| Gene.3807 | scavenger receptor protein [Tribolium castaneum] | 355 | 3 |
| Gene.381 | PREDICTED: nuclear migration protein nudC [Tribolium castaneum] | 270 | 3 |
| Gene.3816 | ATP-dependent RNA helicase vasa-like [Leptinotarsa decemlineata] | 137 | 3 |
| Gene.3816 | ATP-dependent RNA helicase vasa-like [Leptinotarsa decemlineata] | 453 | 3 |
| Gene.3816 | ATP-dependent RNA helicase vasa-like [Leptinotarsa decemlineata] | 542 | 3 |
| Gene.3816 | ATP-dependent RNA helicase vasa-like [Leptinotarsa decemlineata] | 433 | 3 |
| Gene.3816 | ATP-dependent RNA helicase vasa-like [Leptinotarsa decemlineata] | 299 | 3 |
| Gene.3838 | PREDICTED: pyruvate carboxylase, mitochondrial isoform X3 [Tribolium castaneum] | 152 | 3 |
| Gene.3838 | PREDICTED: pyruvate carboxylase, mitochondrial isoform X3 [Tribolium castaneum] | 1041 | 3 |
| Gene.3838 | PREDICTED: pyruvate carboxylase, mitochondrial isoform X3 [Tribolium castaneum] | 107 | 3 |
| Gene.3838 | PREDICTED: pyruvate carboxylase, mitochondrial isoform X3 [Tribolium castaneum] | 434 | 3 |
| Gene.3838 | PREDICTED: pyruvate carboxylase, mitochondrial isoform X3 [Tribolium castaneum] | 944 | 3 |
| Gene.3838 | PREDICTED: pyruvate carboxylase, mitochondrial isoform X3 [Tribolium castaneum] | 937 | 3 |
| Gene.3838 | PREDICTED: pyruvate carboxylase, mitochondrial isoform X3 [Tribolium castaneum] | 742 | 3 |
| Gene.3838 | PREDICTED: pyruvate carboxylase, mitochondrial isoform X3 [Tribolium castaneum] | 144 | 3 |
| Gene.3838 | PREDICTED: pyruvate carboxylase, mitochondrial isoform X3 [Tribolium castaneum] | 1113 | 3 |
| Gene.3838 | PREDICTED: pyruvate carboxylase, mitochondrial isoform X3 [Tribolium castaneum] | 290 | 3 |
| Gene.3838 | PREDICTED: pyruvate carboxylase, mitochondrial isoform X3 [Tribolium castaneum] | 297 | 3 |
| Gene.3838 | PREDICTED: pyruvate carboxylase, mitochondrial isoform X3 [Tribolium castaneum] | 935 | 3 |
| Gene.3838 | PREDICTED: pyruvate carboxylase, mitochondrial isoform X3 [Tribolium castaneum] | 1005 | 3 |
| Gene.3838 | PREDICTED: pyruvate carboxylase, mitochondrial isoform X3 [Tribolium castaneum] | 998 | 3 |
| Gene.3838 | PREDICTED: pyruvate carboxylase, mitochondrial isoform X3 [Tribolium castaneum] | 760 | 3 |
| Gene.3838 | PREDICTED: pyruvate carboxylase, mitochondrial isoform X3 [Tribolium castaneum] | 273 | 3 |
| Gene.3838 | PREDICTED: pyruvate carboxylase, mitochondrial isoform X3 [Tribolium castaneum] | 442 | 3 |
| Gene.3838 | PREDICTED: pyruvate carboxylase, mitochondrial isoform X3 [Tribolium castaneum] | 758 | 3 |
| Gene.3838 | PREDICTED: pyruvate carboxylase, mitochondrial isoform X3 [Tribolium castaneum] | 751 | 3 |
| Gene.3838 | PREDICTED: pyruvate carboxylase, mitochondrial isoform X3 [Tribolium castaneum] | 209 | 3 |
| Gene.3838 | PREDICTED: pyruvate carboxylase, mitochondrial isoform X3 [Tribolium castaneum] | 1106 | 3 |
| Gene.3838 | PREDICTED: pyruvate carboxylase, mitochondrial isoform X3 [Tribolium castaneum] | 556 | 3 |
| Gene.3838 | PREDICTED: pyruvate carboxylase, mitochondrial isoform X3 [Tribolium castaneum] | 177 | 3 |
| Gene.3838 | PREDICTED: pyruvate carboxylase, mitochondrial isoform X3 [Tribolium castaneum] | 62 | 3 |
| Gene.3866 | 60S ribosomal protein L26 [Agrilus planipennis] | 42 | 3 |
| Gene.3866 | 60S ribosomal protein L26 [Agrilus planipennis] | 69 | 3 |
| Gene.3866 | 60S ribosomal protein L26 [Agrilus planipennis] | 11 | 3 |
| Gene.3866 | 60S ribosomal protein L26 [Agrilus planipennis] | 83 | 3 |
| Gene.3866 | 60S ribosomal protein L26 [Agrilus planipennis] | 75 | 3 |
| Gene.3866 | 60S ribosomal protein L26 [Agrilus planipennis] | 144 | 3 |
| Gene.3866 | 60S ribosomal protein L26 [Agrilus planipennis] | 138 | 3 |
| Gene.3866 | 60S ribosomal protein L26 [Agrilus planipennis] | 114 | 3 |
| Gene.3873 | PREDICTED: muscle LIM protein Mlp84B isoform X1 [Tribolium castaneum] | 22 | 3 |
| Gene.3875 | PREDICTED: probable methylmalonate-semialdehyde dehydrogenase [acylating], mitochondrial [Aethina tumida] | 350 | 3 |
| Gene.3875 | PREDICTED: probable methylmalonate-semialdehyde dehydrogenase [acylating], mitochondrial [Aethina tumida] | 241 | 3 |
| Gene.3875 | PREDICTED: probable methylmalonate-semialdehyde dehydrogenase [acylating], mitochondrial [Aethina tumida] | 253 | 3 |
| Gene.3897 | PREDICTED: prosaposin [Tribolium castaneum] | 106 | 3 |
| Gene.3901 | aldose 1-epimerase-like [Leptinotarsa decemlineata] | 291 | 3 |
| Gene.391 | ubiquitin carboxyl-terminal hydrolase [Asbolus verrucosus] | 123 | 3 |
| Gene.391 | ubiquitin carboxyl-terminal hydrolase [Asbolus verrucosus] | 196 | 3 |
| Gene.391 | ubiquitin carboxyl-terminal hydrolase [Asbolus verrucosus] | 211 | 3 |
| Gene.3911 | PREDICTED: LOW QUALITY PROTEIN: heat shock 70 kDa protein 4 [Aethina tumida] | 276 | 3 |
| Gene.3911 | PREDICTED: LOW QUALITY PROTEIN: heat shock 70 kDa protein 4 [Aethina tumida] | 239 | 3 |
| Gene.3912 | PREDICTED: LOW QUALITY PROTEIN: heat shock 70 kDa protein 4 [Aethina tumida] | 254 | 3 |
| Gene.3912 | PREDICTED: LOW QUALITY PROTEIN: heat shock 70 kDa protein 4 [Aethina tumida] | 354 | 3 |
| Gene.3918 | microtubule-actin cross-linking factor 1 isoform X5 [Anoplophora glabripennis] | 425 | 3 |
| Gene.3939 | PREDICTED: fragile X mental retardation syndrome-related protein 1 isoform X2 [Tribolium castaneum] | 147 | 3 |
| Gene.3939 | PREDICTED: fragile X mental retardation syndrome-related protein 1 isoform X2 [Tribolium castaneum] | 376 | 3 |
| Gene.3939 | PREDICTED: fragile X mental retardation syndrome-related protein 1 isoform X2 [Tribolium castaneum] | 279 | 3 |
| Gene.3939 | PREDICTED: fragile X mental retardation syndrome-related protein 1 isoform X2 [Tribolium castaneum] | 301 | 3 |
| Gene.3947 | PREDICTED: delta-1-pyrroline-5-carboxylate synthase [Tribolium castaneum] | 476 | 3 |
| Gene.3947 | PREDICTED: delta-1-pyrroline-5-carboxylate synthase [Tribolium castaneum] | 330 | 3 |
| Gene.3947 | PREDICTED: delta-1-pyrroline-5-carboxylate synthase [Tribolium castaneum] | 207 | 3 |
| Gene.3947 | PREDICTED: delta-1-pyrroline-5-carboxylate synthase [Tribolium castaneum] | 607 | 3 |
| Gene.3947 | PREDICTED: delta-1-pyrroline-5-carboxylate synthase [Tribolium castaneum] | 531 | 3 |
| Gene.3947 | PREDICTED: delta-1-pyrroline-5-carboxylate synthase [Tribolium castaneum] | 425 | 3 |
| Gene.3947 | PREDICTED: delta-1-pyrroline-5-carboxylate synthase [Tribolium castaneum] | 353 | 3 |
| Gene.3947 | PREDICTED: delta-1-pyrroline-5-carboxylate synthase [Tribolium castaneum] | 321 | 3 |
| Gene.3947 | PREDICTED: delta-1-pyrroline-5-carboxylate synthase [Tribolium castaneum] | 318 | 3 |
| Gene.3947 | PREDICTED: delta-1-pyrroline-5-carboxylate synthase [Tribolium castaneum] | 432 | 3 |
| Gene.3948 | PREDICTED: delta-1-pyrroline-5-carboxylate synthase [Nicrophorus vespilloides] | 34 | 3 |
| Gene.3948 | PREDICTED: delta-1-pyrroline-5-carboxylate synthase [Nicrophorus vespilloides] | 31 | 3 |
| Gene.3972 | pyruvate dehydrogenase E1 component subunit beta, mitochondrial [Leptinotarsa decemlineata] | 185 | 3 |
| Gene.3972 | pyruvate dehydrogenase E1 component subunit beta, mitochondrial [Leptinotarsa decemlineata] | 228 | 3 |
| Gene.3972 | pyruvate dehydrogenase E1 component subunit beta, mitochondrial [Leptinotarsa decemlineata] | 260 | 3 |
| Gene.3999 | NAD binding 2 domain containing protein [Asbolus verrucosus] | 84 | 3 |
| Gene.3999 | NAD binding 2 domain containing protein [Asbolus verrucosus] | 217 | 3 |
| Gene.3999 | NAD binding 2 domain containing protein [Asbolus verrucosus] | 207 | 3 |
| Gene.3999 | NAD binding 2 domain containing protein [Asbolus verrucosus] | 275 | 3 |
| Gene.400 | serine--tRNA ligase, cytoplasmic [Leptinotarsa decemlineata] | 262 | 3 |
| Gene.4011 | fatty acid synthase [Coccinella septempunctata] | 1002 | 3 |
| Gene.4011 | fatty acid synthase [Coccinella septempunctata] | 503 | 3 |
| Gene.4011 | fatty acid synthase [Coccinella septempunctata] | 2371 | 3 |
| Gene.4011 | fatty acid synthase [Coccinella septempunctata] | 460 | 3 |
| Gene.4011 | fatty acid synthase [Coccinella septempunctata] | 1887 | 3 |
| Gene.4011 | fatty acid synthase [Coccinella septempunctata] | 526 | 3 |
| Gene.4011 | fatty acid synthase [Coccinella septempunctata] | 674 | 3 |
| Gene.4011 | fatty acid synthase [Coccinella septempunctata] | 998 | 3 |
| Gene.4011 | fatty acid synthase [Coccinella septempunctata] | 273 | 3 |
| Gene.4011 | fatty acid synthase [Coccinella septempunctata] | 1122 | 3 |
| Gene.4011 | fatty acid synthase [Coccinella septempunctata] | 1881 | 3 |
| Gene.4011 | fatty acid synthase [Coccinella septempunctata] | 699 | 3 |
| Gene.4011 | fatty acid synthase [Coccinella septempunctata] | 450 | 3 |
| Gene.4011 | fatty acid synthase [Coccinella septempunctata] | 2094 | 3 |
| Gene.4011 | fatty acid synthase [Coccinella septempunctata] | 1744 | 3 |
| Gene.4011 | fatty acid synthase [Coccinella septempunctata] | 2077 | 3 |
| Gene.4011 | fatty acid synthase [Coccinella septempunctata] | 1597 | 3 |
| Gene.4011 | fatty acid synthase [Coccinella septempunctata] | 987 | 3 |
| Gene.4011 | fatty acid synthase [Coccinella septempunctata] | 1892 | 3 |
| Gene.4011 | fatty acid synthase [Coccinella septempunctata] | 990 | 3 |
| Gene.4011 | fatty acid synthase [Coccinella septempunctata] | 2121 | 3 |
| Gene.4011 | fatty acid synthase [Coccinella septempunctata] | 707 | 3 |
| Gene.4011 | fatty acid synthase [Coccinella septempunctata] | 1016 | 3 |
| Gene.4011 | fatty acid synthase [Coccinella septempunctata] | 494 | 3 |
| Gene.4011 | fatty acid synthase [Coccinella septempunctata] | 1398 | 3 |
| Gene.4011 | fatty acid synthase [Coccinella septempunctata] | 1740 | 3 |
| Gene.4011 | fatty acid synthase [Coccinella septempunctata] | 1453 | 3 |
| Gene.4015 | activated RNA polymerase II transcriptional coactivator p15 [Asbolus verrucosus] | 50 | 3 |
| Gene.4015 | activated RNA polymerase II transcriptional coactivator p15 [Asbolus verrucosus] | 85 | 3 |
| Gene.4015 | activated RNA polymerase II transcriptional coactivator p15 [Asbolus verrucosus] | 45 | 3 |
| Gene.4016 | AAEL010007-PA [Aedes aegypti] | 403 | 3 |
| Gene.4018 | glutamyl aminopeptidase-like isoform X2 [Leptinotarsa decemlineata] | 408 | 3 |
| Gene.4022 | protein singed [Anoplophora glabripennis] | 443 | 3 |
| Gene.4027 | Annexin domain containing protein, partial [Asbolus verrucosus] | 245 | 3 |
| Gene.4027 | Annexin domain containing protein, partial [Asbolus verrucosus] | 264 | 3 |
| Gene.4027 | Annexin domain containing protein, partial [Asbolus verrucosus] | 351 | 3 |
| Gene.4035 | Uncharacterized protein | 11 | 3 |
| Gene.4067 | PREDICTED: programmed cell death 6-interacting protein [Tribolium castaneum] | 95 | 3 |
| Gene.4067 | PREDICTED: programmed cell death 6-interacting protein [Tribolium castaneum] | 449 | 3 |
| Gene.4067 | PREDICTED: programmed cell death 6-interacting protein [Tribolium castaneum] | 208 | 3 |
| Gene.4067 | PREDICTED: programmed cell death 6-interacting protein [Tribolium castaneum] | 100 | 3 |
| Gene.4078 | uncharacterized protein LOC108917852 [Anoplophora glabripennis] | 413 | 3 |
| Gene.4078 | uncharacterized protein LOC108917852 [Anoplophora glabripennis] | 34 | 3 |
| Gene.4078 | uncharacterized protein LOC108917852 [Anoplophora glabripennis] | 63 | 3 |
| Gene.4078 | uncharacterized protein LOC108917852 [Anoplophora glabripennis] | 374 | 3 |
| Gene.4078 | uncharacterized protein LOC108917852 [Anoplophora glabripennis] | 507 | 3 |
| Gene.4078 | uncharacterized protein LOC108917852 [Anoplophora glabripennis] | 80 | 3 |
| Gene.4081 | Ribosomal L19e domain containing protein [Asbolus verrucosus] | 204 | 3 |
| Gene.4081 | Ribosomal L19e domain containing protein [Asbolus verrucosus] | 139 | 3 |
| Gene.4081 | Ribosomal L19e domain containing protein [Asbolus verrucosus] | 144 | 3 |
| Gene.4081 | Ribosomal L19e domain containing protein [Asbolus verrucosus] | 164 | 3 |
| Gene.4087 | 28S ribosomal protein S28, mitochondrial [Anoplophora glabripennis] | 82 | 3 |
| Gene.4100 | PREDICTED: 40S ribosomal protein S14 [Aethina tumida] | 61 | 3 |
| Gene.4100 | PREDICTED: 40S ribosomal protein S14 [Aethina tumida] | 125 | 3 |
| Gene.4113 | succinyl-CoA:3-ketoacid coenzyme A transferase 1, mitochondrial, partial [Asbolus verrucosus] | 171 | 3 |
| Gene.4132 | phosphoribosylformylglycinamidine synthase [Anoplophora glabripennis] | 216 | 3 |
| Gene.4132 | phosphoribosylformylglycinamidine synthase [Anoplophora glabripennis] | 82 | 3 |
| Gene.4132 | phosphoribosylformylglycinamidine synthase [Anoplophora glabripennis] | 454 | 3 |
| Gene.4134 | PREDICTED: AMP deaminase 2 isoform X6 [Tribolium castaneum] | 445 | 3 |
| Gene.4134 | PREDICTED: AMP deaminase 2 isoform X6 [Tribolium castaneum] | 356 | 3 |
| Gene.4134 | PREDICTED: AMP deaminase 2 isoform X6 [Tribolium castaneum] | 187 | 3 |
| Gene.4149 | PREDICTED: LOW QUALITY PROTEIN: 26S proteasome non-ATPase regulatory subunit 1-like [Aethina tumida] | 310 | 3 |
| Gene.4163 | Uncharacterized protein | 131 | 3 |
| Gene.4163 | Uncharacterized protein | 71 | 3 |
| Gene.4163 | Uncharacterized protein | 108 | 3 |
| Gene.4163 | Uncharacterized protein | 91 | 3 |
| Gene.4163 | Uncharacterized protein | 68 | 3 |
| Gene.4163 | Uncharacterized protein | 23 | 3 |
| Gene.4176 | Uncharacterized protein | 71 | 3 |
| Gene.4178 | mitochondrial 2-oxoglutarate/malate carrier protein [Asbolus verrucosus] | 250 | 3 |
| Gene.4178 | mitochondrial 2-oxoglutarate/malate carrier protein [Asbolus verrucosus] | 258 | 3 |
| Gene.4183 | PREDICTED: proton-coupled amino acid transporter 1 isoform X1 [Tribolium castaneum] | 20 | 3 |
| Gene.4183 | PREDICTED: proton-coupled amino acid transporter 1 isoform X1 [Tribolium castaneum] | 15 | 3 |
| Gene.4190 | V-type proton ATPase subunit H [Asbolus verrucosus] | 409 | 3 |
| Gene.42 | cytochrome P450 monooxygenase CYP6BQ37 [Tenebrio molitor] | 175 | 3 |
| Gene.4205 | uncharacterized protein LOC108911898 [Anoplophora glabripennis] | 535 | 3 |
| Gene.4205 | uncharacterized protein LOC108911898 [Anoplophora glabripennis] | 254 | 3 |
| Gene.4221 | Prefoldin 2 domain containing protein [Asbolus verrucosus] | 43 | 3 |
| Gene.4230 | A disintegrin and metalloproteinase with thrombospondin motifs adt-1-like [Anoplophora glabripennis] | 884 | 3 |
| Gene.4244 | PREDICTED: protein NipSnap-like [Aethina tumida] | 136 | 3 |
| Gene.4244 | PREDICTED: protein NipSnap-like [Aethina tumida] | 53 | 3 |
| Gene.4244 | PREDICTED: protein NipSnap-like [Aethina tumida] | 143 | 3 |
| Gene.425 | glutathione S-transferase 1-1 [Anoplophora glabripennis] | 128 | 3 |
| Gene.425 | glutathione S-transferase 1-1 [Anoplophora glabripennis] | 131 | 3 |
| Gene.425 | glutathione S-transferase 1-1 [Anoplophora glabripennis] | 215 | 3 |
| Gene.425 | glutathione S-transferase 1-1 [Anoplophora glabripennis] | 201 | 3 |
| Gene.425 | glutathione S-transferase 1-1 [Anoplophora glabripennis] | 207 | 3 |
| Gene.425 | glutathione S-transferase 1-1 [Anoplophora glabripennis] | 186 | 3 |
| Gene.425 | glutathione S-transferase 1-1 [Anoplophora glabripennis] | 37 | 3 |
| Gene.4254 | isocitrate dehydrogenase [NAD] subunit gamma, mitochondrial isoform X2 [Anoplophora glabripennis] | 234 | 3 |
| Gene.426 | PREDICTED: LOW QUALITY PROTEIN: amidophosphoribosyltransferase-like [Aethina tumida] | 445 | 3 |
| Gene.4262 | sarcosine dehydrogenase, mitochondrial [Asbolus verrucosus] | 272 | 3 |
| Gene.4262 | sarcosine dehydrogenase, mitochondrial [Asbolus verrucosus] | 636 | 3 |
| Gene.4262 | sarcosine dehydrogenase, mitochondrial [Asbolus verrucosus] | 165 | 3 |
| Gene.4262 | sarcosine dehydrogenase, mitochondrial [Asbolus verrucosus] | 451 | 3 |
| Gene.4262 | sarcosine dehydrogenase, mitochondrial [Asbolus verrucosus] | 480 | 3 |
| Gene.4262 | sarcosine dehydrogenase, mitochondrial [Asbolus verrucosus] | 358 | 3 |
| Gene.4262 | sarcosine dehydrogenase, mitochondrial [Asbolus verrucosus] | 499 | 3 |
| Gene.4262 | sarcosine dehydrogenase, mitochondrial [Asbolus verrucosus] | 444 | 3 |
| Gene.4271 | PREDICTED: NADP-dependent malic enzyme isoform X1 [Megachile rotundata] | 163 | 3 |
| Gene.4271 | PREDICTED: NADP-dependent malic enzyme isoform X1 [Megachile rotundata] | 171 | 3 |
| Gene.4272 | PREDICTED: LOW QUALITY PROTEIN: NADP-dependent malic enzyme-like [Aethina tumida] | 220 | 3 |
| Gene.4272 | PREDICTED: LOW QUALITY PROTEIN: NADP-dependent malic enzyme-like [Aethina tumida] | 20 | 3 |
| Gene.4276 | superoxide dismutase, partial [Harmonia axyridis] | 152 | 3 |
| Gene.4276 | superoxide dismutase, partial [Harmonia axyridis] | 74 | 3 |
| Gene.4276 | superoxide dismutase, partial [Harmonia axyridis] | 4 | 3 |
| Gene.4276 | superoxide dismutase, partial [Harmonia axyridis] | 106 | 3 |
| Gene.4276 | superoxide dismutase, partial [Harmonia axyridis] | 134 | 3 |
| Gene.4285 | PREDICTED: LOW QUALITY PROTEIN: pyruvate kinase-like [Aethina tumida] | 93 | 3 |
| Gene.4285 | PREDICTED: LOW QUALITY PROTEIN: pyruvate kinase-like [Aethina tumida] | 245 | 3 |
| Gene.429 | 40S ribosomal protein S16 [Anoplophora glabripennis] | 109 | 3 |
| Gene.429 | 40S ribosomal protein S16 [Anoplophora glabripennis] | 49 | 3 |
| Gene.429 | 40S ribosomal protein S16 [Anoplophora glabripennis] | 64 | 3 |
| Gene.429 | 40S ribosomal protein S16 [Anoplophora glabripennis] | 94 | 3 |
| Gene.429 | 40S ribosomal protein S16 [Anoplophora glabripennis] | 102 | 3 |
| Gene.429 | 40S ribosomal protein S16 [Anoplophora glabripennis] | 135 | 3 |
| Gene.4298 | electron transfer flavoprotein-ubiquinone oxidoreductase, mitochondrial [Leptinotarsa decemlineata] | 11 | 3 |
| Gene.4299 | electron transfer flavoprotein-ubiquinone oxidoreductase, mitochondrial [Anoplophora glabripennis] | 210 | 3 |
| Gene.4299 | electron transfer flavoprotein-ubiquinone oxidoreductase, mitochondrial [Anoplophora glabripennis] | 45 | 3 |
| Gene.4299 | electron transfer flavoprotein-ubiquinone oxidoreductase, mitochondrial [Anoplophora glabripennis] | 133 | 3 |
| Gene.4299 | electron transfer flavoprotein-ubiquinone oxidoreductase, mitochondrial [Anoplophora glabripennis] | 140 | 3 |
| Gene.4299 | electron transfer flavoprotein-ubiquinone oxidoreductase, mitochondrial [Anoplophora glabripennis] | 331 | 3 |
| Gene.4299 | electron transfer flavoprotein-ubiquinone oxidoreductase, mitochondrial [Anoplophora glabripennis] | 121 | 3 |
| Gene.4302 | long-chain-fatty-acid--CoA ligase 4 isoform X1 [Agrilus planipennis] | 243 | 3 |
| Gene.4302 | long-chain-fatty-acid--CoA ligase 4 isoform X1 [Agrilus planipennis] | 416 | 3 |
| Gene.4302 | long-chain-fatty-acid--CoA ligase 4 isoform X1 [Agrilus planipennis] | 411 | 3 |
| Gene.4302 | long-chain-fatty-acid--CoA ligase 4 isoform X1 [Agrilus planipennis] | 263 | 3 |
| Gene.4303 | PREDICTED: LOW QUALITY PROTEIN: long-chain-fatty-acid--CoA ligase 4 [Aethina tumida] | 171 | 3 |
| Gene.4326 | hemocytin [Agrilus planipennis] | 62 | 3 |
| Gene.4326 | hemocytin [Agrilus planipennis] | 171 | 3 |
| Gene.4335 | alpha-aminoadipic semialdehyde synthase, mitochondrial [Anoplophora glabripennis] | 148 | 3 |
| Gene.4352 | PREDICTED: calcium-transporting ATPase sarcoplasmic/endoplasmic reticulum type isoform X1 [Tribolium castaneum] | 128 | 3 |
| Gene.4352 | PREDICTED: calcium-transporting ATPase sarcoplasmic/endoplasmic reticulum type isoform X1 [Tribolium castaneum] | 514 | 3 |
| Gene.4352 | PREDICTED: calcium-transporting ATPase sarcoplasmic/endoplasmic reticulum type isoform X1 [Tribolium castaneum] | 30 | 3 |
| Gene.4357 | 60S ribosomal protein L37 [Onthophagus taurus] | 64 | 3 |
| Gene.4357 | 60S ribosomal protein L37 [Onthophagus taurus] | 55 | 3 |
| Gene.4366 | phosphoglucomutase-2 [Leptinotarsa decemlineata] | 47 | 3 |
| Gene.4368 | ATP-binding cassette sub-family G member 4-like protein [Harmonia axyridis] | 122 | 3 |
| Gene.4369 | ATP-binding cassette sub-family G member 4-like protein [Harmonia axyridis] | 65 | 3 |
| Gene.4379 | cathepsin L precursor [Tribolium castaneum] | 57 | 3 |
| Gene.4384 | Twitchin-like Protein [Tribolium castaneum] | 161 | 3 |
| Gene.4392 | Membrane metallo-endopeptidase-like 1, partial [Habropoda laboriosa] | 281 | 3 |
| Gene.4392 | Membrane metallo-endopeptidase-like 1, partial [Habropoda laboriosa] | 224 | 3 |
| Gene.4392 | Membrane metallo-endopeptidase-like 1, partial [Habropoda laboriosa] | 193 | 3 |
| Gene.4396 | PREDICTED: 2-hydroxyacylsphingosine 1-beta-galactosyltransferase-like [Tribolium castaneum] | 69 | 3 |
| Gene.4396 | PREDICTED: 2-hydroxyacylsphingosine 1-beta-galactosyltransferase-like [Tribolium castaneum] | 317 | 3 |
| Gene.4396 | PREDICTED: 2-hydroxyacylsphingosine 1-beta-galactosyltransferase-like [Tribolium castaneum] | 259 | 3 |
| Gene.4396 | PREDICTED: 2-hydroxyacylsphingosine 1-beta-galactosyltransferase-like [Tribolium castaneum] | 49 | 3 |
| Gene.4404 | peroxisomal acyl-coenzyme A oxidase 3 isoform X1 [Anoplophora glabripennis] | 26 | 3 |
| Gene.4417 | glycogen phosphorylase [Harmonia axyridis] | 205 | 3 |
| Gene.4417 | glycogen phosphorylase [Harmonia axyridis] | 92 | 3 |
| Gene.4417 | glycogen phosphorylase [Harmonia axyridis] | 120 | 3 |
| Gene.4417 | glycogen phosphorylase [Harmonia axyridis] | 134 | 3 |
| Gene.4417 | glycogen phosphorylase [Harmonia axyridis] | 288 | 3 |
| Gene.4417 | glycogen phosphorylase [Harmonia axyridis] | 44 | 3 |
| Gene.4417 | glycogen phosphorylase [Harmonia axyridis] | 113 | 3 |
| Gene.4419 | glycogen phosphorylase [Harmonia axyridis] | 291 | 3 |
| Gene.4419 | glycogen phosphorylase [Harmonia axyridis] | 177 | 3 |
| Gene.4419 | glycogen phosphorylase [Harmonia axyridis] | 10 | 3 |
| Gene.4419 | glycogen phosphorylase [Harmonia axyridis] | 78 | 3 |
| Gene.4419 | glycogen phosphorylase [Harmonia axyridis] | 249 | 3 |
| Gene.4419 | glycogen phosphorylase [Harmonia axyridis] | 29 | 3 |
| Gene.4432 | PREDICTED: major facilitator superfamily domain-containing protein 9 [Tribolium castaneum] | 209 | 3 |
| Gene.4439 | Basement membrane-specific heparan sulfate proteoglycan core protein-like Protein [Tribolium castaneum] | 159 | 3 |
| Gene.4439 | Basement membrane-specific heparan sulfate proteoglycan core protein-like Protein [Tribolium castaneum] | 770 | 3 |
| Gene.4439 | Basement membrane-specific heparan sulfate proteoglycan core protein-like Protein [Tribolium castaneum] | 51 | 3 |
| Gene.4439 | Basement membrane-specific heparan sulfate proteoglycan core protein-like Protein [Tribolium castaneum] | 1234 | 3 |
| Gene.4447 | PREDICTED: NAD(P) transhydrogenase, mitochondrial [Tribolium castaneum] | 87 | 3 |
| Gene.4453 | lipase 3 [Cephus cinctus] | 14 | 3 |
| Gene.4476 | Ribosomal S17e domain containing protein [Asbolus verrucosus] | 21 | 3 |
| Gene.4476 | Ribosomal S17e domain containing protein [Asbolus verrucosus] | 56 | 3 |
| Gene.4476 | Ribosomal S17e domain containing protein [Asbolus verrucosus] | 51 | 3 |
| Gene.4476 | Ribosomal S17e domain containing protein [Asbolus verrucosus] | 26 | 3 |
| Gene.4476 | Ribosomal S17e domain containing protein [Asbolus verrucosus] | 79 | 3 |
| Gene.4480 | PREDICTED: eukaryotic translation initiation factor 3 subunit A [Tribolium castaneum] | 775 | 3 |
| Gene.4480 | PREDICTED: eukaryotic translation initiation factor 3 subunit A [Tribolium castaneum] | 374 | 3 |
| Gene.453 | DEAD-box ATP-dependent RNA helicase 20-like [Onthophagus taurus] | 382 | 3 |
| Gene.4531 | PREDICTED: glutamate synthase 1 [NADH], chloroplastic isoform X2 [Tribolium castaneum] | 280 | 3 |
| Gene.455 | ATP-binding cassette sub-family E member 1 [Anoplophora glabripennis] | 189 | 3 |
| Gene.4581 | PREDICTED: trifunctional enzyme subunit alpha, mitochondrial [Tribolium castaneum] | 385 | 3 |
| Gene.4581 | PREDICTED: trifunctional enzyme subunit alpha, mitochondrial [Tribolium castaneum] | 118 | 3 |
| Gene.4581 | PREDICTED: trifunctional enzyme subunit alpha, mitochondrial [Tribolium castaneum] | 280 | 3 |
| Gene.4581 | PREDICTED: trifunctional enzyme subunit alpha, mitochondrial [Tribolium castaneum] | 199 | 3 |
| Gene.4581 | PREDICTED: trifunctional enzyme subunit alpha, mitochondrial [Tribolium castaneum] | 249 | 3 |
| Gene.4581 | PREDICTED: trifunctional enzyme subunit alpha, mitochondrial [Tribolium castaneum] | 401 | 3 |
| Gene.4581 | PREDICTED: trifunctional enzyme subunit alpha, mitochondrial [Tribolium castaneum] | 408 | 3 |
| Gene.4581 | PREDICTED: trifunctional enzyme subunit alpha, mitochondrial [Tribolium castaneum] | 126 | 3 |
| Gene.4582 | PREDICTED: lachesin [Tribolium castaneum] | 295 | 3 |
| Gene.4583 | PREDICTED: ATP-citrate synthase isoform X1 [Tribolium castaneum] | 90 | 3 |
| Gene.4583 | PREDICTED: ATP-citrate synthase isoform X1 [Tribolium castaneum] | 53 | 3 |
| Gene.4583 | PREDICTED: ATP-citrate synthase isoform X1 [Tribolium castaneum] | 40 | 3 |
| Gene.4583 | PREDICTED: ATP-citrate synthase isoform X1 [Tribolium castaneum] | 957 | 3 |
| Gene.4583 | PREDICTED: ATP-citrate synthase isoform X1 [Tribolium castaneum] | 531 | 3 |
| Gene.4583 | PREDICTED: ATP-citrate synthase isoform X1 [Tribolium castaneum] | 537 | 3 |
| Gene.4583 | PREDICTED: ATP-citrate synthase isoform X1 [Tribolium castaneum] | 545 | 3 |
| Gene.4583 | PREDICTED: ATP-citrate synthase isoform X1 [Tribolium castaneum] | 766 | 3 |
| Gene.4583 | PREDICTED: ATP-citrate synthase isoform X1 [Tribolium castaneum] | 321 | 3 |
| Gene.4583 | PREDICTED: ATP-citrate synthase isoform X1 [Tribolium castaneum] | 1071 | 3 |
| Gene.4583 | PREDICTED: ATP-citrate synthase isoform X1 [Tribolium castaneum] | 140 | 3 |
| Gene.4593 | spectrin beta chain isoform X1 [Anoplophora glabripennis] | 418 | 3 |
| Gene.4593 | spectrin beta chain isoform X1 [Anoplophora glabripennis] | 458 | 3 |
| Gene.4593 | spectrin beta chain isoform X1 [Anoplophora glabripennis] | 1089 | 3 |
| Gene.4593 | spectrin beta chain isoform X1 [Anoplophora glabripennis] | 425 | 3 |
| Gene.4593 | spectrin beta chain isoform X1 [Anoplophora glabripennis] | 716 | 3 |
| Gene.4593 | spectrin beta chain isoform X1 [Anoplophora glabripennis] | 1220 | 3 |
| Gene.4593 | spectrin beta chain isoform X1 [Anoplophora glabripennis] | 1077 | 3 |
| Gene.4593 | spectrin beta chain isoform X1 [Anoplophora glabripennis] | 1396 | 3 |
| Gene.4593 | spectrin beta chain isoform X1 [Anoplophora glabripennis] | 1501 | 3 |
| Gene.460 | PREDICTED: bifunctional glutamate/proline--tRNA ligase [Nicrophorus vespilloides] | 336 | 3 |
| Gene.4608 | PREDICTED: ELAV-like protein 3 isoform X2 [Tribolium castaneum] | 99 | 3 |
| Gene.461 | PREDICTED: protein transport protein Sec61 subunit alpha isoform 2 [Tribolium castaneum] | 392 | 3 |
| Gene.461 | PREDICTED: protein transport protein Sec61 subunit alpha isoform 2 [Tribolium castaneum] | 107 | 3 |
| Gene.4622 | putative cysteine proteinase CG12163 [Leptinotarsa decemlineata] | 449 | 3 |
| Gene.4641 | vigilin [Asbolus verrucosus] | 128 | 3 |
| Gene.4641 | vigilin [Asbolus verrucosus] | 138 | 3 |
| Gene.4653 | Uncharacterized protein | 424 | 3 |
| Gene.4653 | Uncharacterized protein | 1156 | 3 |
| Gene.4653 | Uncharacterized protein | 267 | 3 |
| Gene.4653 | Uncharacterized protein | 940 | 3 |
| Gene.4653 | Uncharacterized protein | 378 | 3 |
| Gene.4653 | Uncharacterized protein | 1164 | 3 |
| Gene.468 | retinal dehydrogenase 1, partial [Asbolus verrucosus] | 27 | 3 |
| Gene.468 | retinal dehydrogenase 1, partial [Asbolus verrucosus] | 427 | 3 |
| Gene.468 | retinal dehydrogenase 1, partial [Asbolus verrucosus] | 131 | 3 |
| Gene.468 | retinal dehydrogenase 1, partial [Asbolus verrucosus] | 20 | 3 |
| Gene.4714 | PREDICTED: 60S ribosomal protein L8 [Polistes canadensis] | 97 | 3 |
| Gene.4714 | PREDICTED: 60S ribosomal protein L8 [Polistes canadensis] | 76 | 3 |
| Gene.4714 | PREDICTED: 60S ribosomal protein L8 [Polistes canadensis] | 43 | 3 |
| Gene.4718 | AGAP001345-PA [Anopheles gambiae str. PEST] | 67 | 3 |
| Gene.4718 | AGAP001345-PA [Anopheles gambiae str. PEST] | 28 | 3 |
| Gene.4718 | AGAP001345-PA [Anopheles gambiae str. PEST] | 260 | 3 |
| Gene.4718 | AGAP001345-PA [Anopheles gambiae str. PEST] | 12 | 3 |
| Gene.4720 | hexamerin 3 precursor [Tribolium castaneum] | 37 | 3 |
| Gene.4720 | hexamerin 3 precursor [Tribolium castaneum] | 183 | 3 |
| Gene.473 | T-complex protein 1 subunit zeta [Asbolus verrucosus] | 263 | 3 |
| Gene.473 | T-complex protein 1 subunit zeta [Asbolus verrucosus] | 400 | 3 |
| Gene.473 | T-complex protein 1 subunit zeta [Asbolus verrucosus] | 220 | 3 |
| Gene.473 | T-complex protein 1 subunit zeta [Asbolus verrucosus] | 477 | 3 |
| Gene.473 | T-complex protein 1 subunit zeta [Asbolus verrucosus] | 513 | 3 |
| Gene.475 | PREDICTED: myosin heavy chain 95F isoform X1 [Tribolium castaneum] | 535 | 3 |
| Gene.475 | PREDICTED: myosin heavy chain 95F isoform X1 [Tribolium castaneum] | 688 | 3 |
| Gene.475 | PREDICTED: myosin heavy chain 95F isoform X1 [Tribolium castaneum] | 189 | 3 |
| Gene.475 | PREDICTED: myosin heavy chain 95F isoform X1 [Tribolium castaneum] | 45 | 3 |
| Gene.4752 | hexamerin 3 precursor [Tribolium castaneum] | 565 | 3 |
| Gene.4752 | hexamerin 3 precursor [Tribolium castaneum] | 552 | 3 |
| Gene.4752 | hexamerin 3 precursor [Tribolium castaneum] | 121 | 3 |
| Gene.4752 | hexamerin 3 precursor [Tribolium castaneum] | 125 | 3 |
| Gene.4752 | hexamerin 3 precursor [Tribolium castaneum] | 582 | 3 |
| Gene.4752 | hexamerin 3 precursor [Tribolium castaneum] | 305 | 3 |
| Gene.4752 | hexamerin 3 precursor [Tribolium castaneum] | 108 | 3 |
| Gene.4752 | hexamerin 3 precursor [Tribolium castaneum] | 316 | 3 |
| Gene.4752 | hexamerin 3 precursor [Tribolium castaneum] | 60 | 3 |
| Gene.4752 | hexamerin 3 precursor [Tribolium castaneum] | 177 | 3 |
| Gene.4752 | hexamerin 3 precursor [Tribolium castaneum] | 196 | 3 |
| Gene.4752 | hexamerin 3 precursor [Tribolium castaneum] | 113 | 3 |
| Gene.4752 | hexamerin 3 precursor [Tribolium castaneum] | 574 | 3 |
| Gene.4754 | NADPH--cytochrome P450 reductase isoform X1 [Anoplophora glabripennis] | 42 | 3 |
| Gene.4756 | NADPH--cytochrome P450 reductase, partial [Asbolus verrucosus] | 71 | 3 |
| Gene.4786 | paramyosin, long form isoform X1 [Leptinotarsa decemlineata] | 204 | 3 |
| Gene.4786 | paramyosin, long form isoform X1 [Leptinotarsa decemlineata] | 174 | 3 |
| Gene.4786 | paramyosin, long form isoform X1 [Leptinotarsa decemlineata] | 216 | 3 |
| Gene.4786 | paramyosin, long form isoform X1 [Leptinotarsa decemlineata] | 9 | 3 |
| Gene.4786 | paramyosin, long form isoform X1 [Leptinotarsa decemlineata] | 301 | 3 |
| Gene.4786 | paramyosin, long form isoform X1 [Leptinotarsa decemlineata] | 222 | 3 |
| Gene.4787 | paramyosin, long form isoform X2 [Leptinotarsa decemlineata] | 56 | 3 |
| Gene.4787 | paramyosin, long form isoform X2 [Leptinotarsa decemlineata] | 206 | 3 |
| Gene.4787 | paramyosin, long form isoform X2 [Leptinotarsa decemlineata] | 229 | 3 |
| Gene.4787 | paramyosin, long form isoform X2 [Leptinotarsa decemlineata] | 253 | 3 |
| Gene.4787 | paramyosin, long form isoform X2 [Leptinotarsa decemlineata] | 245 | 3 |
| Gene.4787 | paramyosin, long form isoform X2 [Leptinotarsa decemlineata] | 193 | 3 |
| Gene.4842 | PREDICTED: laminin subunit gamma-1 isoform X1 [Tribolium castaneum] | 1047 | 3 |
| Gene.4848 | uncharacterized protein LOC108911535 isoform X1 [Anoplophora glabripennis] | 77 | 3 |
| Gene.4867 | PREDICTED: spectrin alpha chain isoform X4 [Tribolium castaneum] | 127 | 3 |
| Gene.4867 | PREDICTED: spectrin alpha chain isoform X4 [Tribolium castaneum] | 370 | 3 |
| Gene.4867 | PREDICTED: spectrin alpha chain isoform X4 [Tribolium castaneum] | 734 | 3 |
| Gene.4867 | PREDICTED: spectrin alpha chain isoform X4 [Tribolium castaneum] | 453 | 3 |
| Gene.4867 | PREDICTED: spectrin alpha chain isoform X4 [Tribolium castaneum] | 912 | 3 |
| Gene.4867 | PREDICTED: spectrin alpha chain isoform X4 [Tribolium castaneum] | 1018 | 3 |
| Gene.4867 | PREDICTED: spectrin alpha chain isoform X4 [Tribolium castaneum] | 2070 | 3 |
| Gene.4867 | PREDICTED: spectrin alpha chain isoform X4 [Tribolium castaneum] | 1412 | 3 |
| Gene.4867 | PREDICTED: spectrin alpha chain isoform X4 [Tribolium castaneum] | 1138 | 3 |
| Gene.4867 | PREDICTED: spectrin alpha chain isoform X4 [Tribolium castaneum] | 1656 | 3 |
| Gene.4867 | PREDICTED: spectrin alpha chain isoform X4 [Tribolium castaneum] | 779 | 3 |
| Gene.4867 | PREDICTED: spectrin alpha chain isoform X4 [Tribolium castaneum] | 1922 | 3 |
| Gene.4867 | PREDICTED: spectrin alpha chain isoform X4 [Tribolium castaneum] | 1455 | 3 |
| Gene.4867 | PREDICTED: spectrin alpha chain isoform X4 [Tribolium castaneum] | 2295 | 3 |
| Gene.4867 | PREDICTED: spectrin alpha chain isoform X4 [Tribolium castaneum] | 287 | 3 |
| Gene.4867 | PREDICTED: spectrin alpha chain isoform X4 [Tribolium castaneum] | 266 | 3 |
| Gene.4867 | PREDICTED: spectrin alpha chain isoform X4 [Tribolium castaneum] | 991 | 3 |
| Gene.4867 | PREDICTED: spectrin alpha chain isoform X4 [Tribolium castaneum] | 2218 | 3 |
| Gene.4867 | PREDICTED: spectrin alpha chain isoform X4 [Tribolium castaneum] | 851 | 3 |
| Gene.4867 | PREDICTED: spectrin alpha chain isoform X4 [Tribolium castaneum] | 1051 | 3 |
| Gene.4867 | PREDICTED: spectrin alpha chain isoform X4 [Tribolium castaneum] | 1165 | 3 |
| Gene.4867 | PREDICTED: spectrin alpha chain isoform X4 [Tribolium castaneum] | 1042 | 3 |
| Gene.4867 | PREDICTED: spectrin alpha chain isoform X4 [Tribolium castaneum] | 356 | 3 |
| Gene.4867 | PREDICTED: spectrin alpha chain isoform X4 [Tribolium castaneum] | 247 | 3 |
| Gene.4926 | translocon-associated protein subunit beta [Leptinotarsa decemlineata] | 32 | 3 |
| Gene.4926 | translocon-associated protein subunit beta [Leptinotarsa decemlineata] | 118 | 3 |
| Gene.4934 | apolipophorins [Anoplophora glabripennis] | 1195 | 3 |
| Gene.4934 | apolipophorins [Anoplophora glabripennis] | 263 | 3 |
| Gene.4934 | apolipophorins [Anoplophora glabripennis] | 266 | 3 |
| Gene.4934 | apolipophorins [Anoplophora glabripennis] | 99 | 3 |
| Gene.4934 | apolipophorins [Anoplophora glabripennis] | 162 | 3 |
| Gene.4934 | apolipophorins [Anoplophora glabripennis] | 292 | 3 |
| Gene.4934 | apolipophorins [Anoplophora glabripennis] | 338 | 3 |
| Gene.4934 | apolipophorins [Anoplophora glabripennis] | 1167 | 3 |
| Gene.4934 | apolipophorins [Anoplophora glabripennis] | 222 | 3 |
| Gene.4934 | apolipophorins [Anoplophora glabripennis] | 304 | 3 |
| Gene.4934 | apolipophorins [Anoplophora glabripennis] | 156 | 3 |
| Gene.4934 | apolipophorins [Anoplophora glabripennis] | 1078 | 3 |
| Gene.4934 | apolipophorins [Anoplophora glabripennis] | 299 | 3 |
| Gene.4934 | apolipophorins [Anoplophora glabripennis] | 277 | 3 |
| Gene.4934 | apolipophorins [Anoplophora glabripennis] | 556 | 3 |
| Gene.4934 | apolipophorins [Anoplophora glabripennis] | 975 | 3 |
| Gene.4934 | apolipophorins [Anoplophora glabripennis] | 980 | 3 |
| Gene.4934 | apolipophorins [Anoplophora glabripennis] | 855 | 3 |
| Gene.4934 | apolipophorins [Anoplophora glabripennis] | 150 | 3 |
| Gene.4935 | apolipophorins [Anoplophora glabripennis] | 38 | 3 |
| Gene.4935 | apolipophorins [Anoplophora glabripennis] | 50 | 3 |
| Gene.4936 | apolipophorin, partial [Asbolus verrucosus] | 98 | 3 |
| Gene.4936 | apolipophorin, partial [Asbolus verrucosus] | 88 | 3 |
| Gene.4936 | apolipophorin, partial [Asbolus verrucosus] | 49 | 3 |
| Gene.4972 | PREDICTED: three prime repair exonuclease 2-like [Aethina tumida] | 167 | 3 |
| Gene.4973 | PREDICTED: myosin heavy chain, muscle isoform X18 [Tribolium castaneum] | 1579 | 3 |
| Gene.4973 | PREDICTED: myosin heavy chain, muscle isoform X18 [Tribolium castaneum] | 1173 | 3 |
| Gene.4973 | PREDICTED: myosin heavy chain, muscle isoform X18 [Tribolium castaneum] | 1457 | 3 |
| Gene.4973 | PREDICTED: myosin heavy chain, muscle isoform X18 [Tribolium castaneum] | 1332 | 3 |
| Gene.4973 | PREDICTED: myosin heavy chain, muscle isoform X18 [Tribolium castaneum] | 907 | 3 |
| Gene.4973 | PREDICTED: myosin heavy chain, muscle isoform X18 [Tribolium castaneum] | 1641 | 3 |
| Gene.4973 | PREDICTED: myosin heavy chain, muscle isoform X18 [Tribolium castaneum] | 599 | 3 |
| Gene.4973 | PREDICTED: myosin heavy chain, muscle isoform X18 [Tribolium castaneum] | 1390 | 3 |
| Gene.4973 | PREDICTED: myosin heavy chain, muscle isoform X18 [Tribolium castaneum] | 84 | 3 |
| Gene.4973 | PREDICTED: myosin heavy chain, muscle isoform X18 [Tribolium castaneum] | 1005 | 3 |
| Gene.4973 | PREDICTED: myosin heavy chain, muscle isoform X18 [Tribolium castaneum] | 1919 | 3 |
| Gene.4973 | PREDICTED: myosin heavy chain, muscle isoform X18 [Tribolium castaneum] | 895 | 3 |
| Gene.4973 | PREDICTED: myosin heavy chain, muscle isoform X18 [Tribolium castaneum] | 450 | 3 |
| Gene.4973 | PREDICTED: myosin heavy chain, muscle isoform X18 [Tribolium castaneum] | 551 | 3 |
| Gene.4973 | PREDICTED: myosin heavy chain, muscle isoform X18 [Tribolium castaneum] | 879 | 3 |
| Gene.4973 | PREDICTED: myosin heavy chain, muscle isoform X18 [Tribolium castaneum] | 1416 | 3 |
| Gene.4973 | PREDICTED: myosin heavy chain, muscle isoform X18 [Tribolium castaneum] | 611 | 3 |
| Gene.4973 | PREDICTED: myosin heavy chain, muscle isoform X18 [Tribolium castaneum] | 1109 | 3 |
| Gene.4973 | PREDICTED: myosin heavy chain, muscle isoform X18 [Tribolium castaneum] | 721 | 3 |
| Gene.4973 | PREDICTED: myosin heavy chain, muscle isoform X18 [Tribolium castaneum] | 1838 | 3 |
| Gene.4973 | PREDICTED: myosin heavy chain, muscle isoform X18 [Tribolium castaneum] | 1262 | 3 |
| Gene.4973 | PREDICTED: myosin heavy chain, muscle isoform X18 [Tribolium castaneum] | 1444 | 3 |
| Gene.4973 | PREDICTED: myosin heavy chain, muscle isoform X18 [Tribolium castaneum] | 1277 | 3 |
| Gene.4973 | PREDICTED: myosin heavy chain, muscle isoform X18 [Tribolium castaneum] | 1247 | 3 |
| Gene.4973 | PREDICTED: myosin heavy chain, muscle isoform X18 [Tribolium castaneum] | 971 | 3 |
| Gene.4973 | PREDICTED: myosin heavy chain, muscle isoform X18 [Tribolium castaneum] | 851 | 3 |
| Gene.4973 | PREDICTED: myosin heavy chain, muscle isoform X18 [Tribolium castaneum] | 1316 | 3 |
| Gene.4973 | PREDICTED: myosin heavy chain, muscle isoform X18 [Tribolium castaneum] | 1374 | 3 |
| Gene.4973 | PREDICTED: myosin heavy chain, muscle isoform X18 [Tribolium castaneum] | 951 | 3 |
| Gene.4973 | PREDICTED: myosin heavy chain, muscle isoform X18 [Tribolium castaneum] | 1354 | 3 |
| Gene.4973 | PREDICTED: myosin heavy chain, muscle isoform X18 [Tribolium castaneum] | 429 | 3 |
| Gene.4973 | PREDICTED: myosin heavy chain, muscle isoform X18 [Tribolium castaneum] | 1103 | 3 |
| Gene.4973 | PREDICTED: myosin heavy chain, muscle isoform X18 [Tribolium castaneum] | 1093 | 3 |
| Gene.4973 | PREDICTED: myosin heavy chain, muscle isoform X18 [Tribolium castaneum] | 1451 | 3 |
| Gene.4973 | PREDICTED: myosin heavy chain, muscle isoform X18 [Tribolium castaneum] | 1791 | 3 |
| Gene.4973 | PREDICTED: myosin heavy chain, muscle isoform X18 [Tribolium castaneum] | 940 | 3 |
| Gene.4988 | ATP-binding cassette sub-family A member 3 [Agrilus planipennis] | 247 | 3 |
| Gene.500 | alanine aminotransferase 1 [Anoplophora glabripennis] | 540 | 3 |
| Gene.500 | alanine aminotransferase 1 [Anoplophora glabripennis] | 316 | 3 |
| Gene.500 | alanine aminotransferase 1 [Anoplophora glabripennis] | 107 | 3 |
| Gene.500 | alanine aminotransferase 1 [Anoplophora glabripennis] | 545 | 3 |
| Gene.500 | alanine aminotransferase 1 [Anoplophora glabripennis] | 434 | 3 |
| Gene.500 | alanine aminotransferase 1 [Anoplophora glabripennis] | 285 | 3 |
| Gene.5015 | multidrug resistance-associated protein 4 [Anoplophora glabripennis] | 46 | 3 |
| Gene.5015 | multidrug resistance-associated protein 4 [Anoplophora glabripennis] | 916 | 3 |
| Gene.5015 | multidrug resistance-associated protein 4 [Anoplophora glabripennis] | 1141 | 3 |
| Gene.502 | acid phosphatase, partial [Cryptolaemus montrouzieri] | 147 | 3 |
| Gene.502 | acid phosphatase, partial [Cryptolaemus montrouzieri] | 141 | 3 |
| Gene.502 | acid phosphatase, partial [Cryptolaemus montrouzieri] | 260 | 3 |
| Gene.5034 | PREDICTED: LOW QUALITY PROTEIN: cytoplasmic aconitate hydratase-like [Aethina tumida] | 83 | 3 |
| Gene.5051 | spectrin alpha chain, non-erythrocytic 1, partial [Asbolus verrucosus] | 640 | 3 |
| Gene.5051 | spectrin alpha chain, non-erythrocytic 1, partial [Asbolus verrucosus] | 1127 | 3 |
| Gene.5051 | spectrin alpha chain, non-erythrocytic 1, partial [Asbolus verrucosus] | 1230 | 3 |
| Gene.5058 | 2-oxoglutarate dehydrogenase E1 component DHKTD1 -like protein, mitochondrial, partial [Asbolus verrucosus] | 234 | 3 |
| Gene.515 | hypothetical protein D910_12346, partial [Dendroctonus ponderosae] | 49 | 3 |
| Gene.5154 | PREDICTED: hemocytin isoform X2 [Tribolium castaneum] | 2626 | 3 |
| Gene.5154 | PREDICTED: hemocytin isoform X2 [Tribolium castaneum] | 2001 | 3 |
| Gene.518 | endothelial differentiation-related factor 1 -like protein [Asbolus verrucosus] | 121 | 3 |
| Gene.5236 | E3 ubiquitin-protein ligase UBR4 [Anoplophora glabripennis] | 3311 | 3 |
| Gene.524 | PREDICTED: trifunctional purine biosynthetic protein adenosine-3 [Tribolium castaneum] | 528 | 3 |
| Gene.524 | PREDICTED: trifunctional purine biosynthetic protein adenosine-3 [Tribolium castaneum] | 19 | 3 |
| Gene.529 | dehydrogenase/reductase SDR family member 4-like [Anoplophora glabripennis] | 294 | 3 |
| Gene.529 | dehydrogenase/reductase SDR family member 4-like [Anoplophora glabripennis] | 130 | 3 |
| Gene.529 | dehydrogenase/reductase SDR family member 4-like [Anoplophora glabripennis] | 208 | 3 |
| Gene.5296 | eukaryotic translation initiation factor 2 subunit 1 [Anoplophora glabripennis] | 96 | 3 |
| Gene.5296 | eukaryotic translation initiation factor 2 subunit 1 [Anoplophora glabripennis] | 79 | 3 |
| Gene.5327 | FUN14 domain-containing protein 1 isoform X1 [Agrilus planipennis] | 141 | 3 |
| Gene.5335 | PREDICTED: interleukin enhancer-binding factor 2 homolog [Tribolium castaneum] | 294 | 3 |
| Gene.5335 | PREDICTED: interleukin enhancer-binding factor 2 homolog [Tribolium castaneum] | 160 | 3 |
| Gene.534 | alanine--glyoxylate aminotransferase 2-like [Leptinotarsa decemlineata] | 177 | 3 |
| Gene.5358 | dnaJ homolog subfamily A member 1 [Leptinotarsa decemlineata] | 280 | 3 |
| Gene.5358 | dnaJ homolog subfamily A member 1 [Leptinotarsa decemlineata] | 37 | 3 |
| Gene.5368 | transcription factor BTF3 homolog 4 [Anoplophora glabripennis] | 162 | 3 |
| Gene.5368 | transcription factor BTF3 homolog 4 [Anoplophora glabripennis] | 37 | 3 |
| Gene.5368 | transcription factor BTF3 homolog 4 [Anoplophora glabripennis] | 44 | 3 |
| Gene.5369 | PREDICTED: UMP-CMP kinase [Aethina tumida] | 84 | 3 |
| Gene.5369 | PREDICTED: UMP-CMP kinase [Aethina tumida] | 82 | 3 |
| Gene.5376 | acyl-coenzyme A thioesterase 13 [Anoplophora glabripennis] | 145 | 3 |
| Gene.5376 | acyl-coenzyme A thioesterase 13 [Anoplophora glabripennis] | 24 | 3 |
| Gene.5376 | acyl-coenzyme A thioesterase 13 [Anoplophora glabripennis] | 132 | 3 |
| Gene.5399 | PREDICTED: 60S ribosomal protein L24 [Aethina tumida] | 35 | 3 |
| Gene.5399 | PREDICTED: 60S ribosomal protein L24 [Aethina tumida] | 23 | 3 |
| Gene.5399 | PREDICTED: 60S ribosomal protein L24 [Aethina tumida] | 19 | 3 |
| Gene.5401 | transmembrane emp24 domain-containing protein bai, partial [Anoplophora glabripennis] | 81 | 3 |
| Gene.5401 | transmembrane emp24 domain-containing protein bai, partial [Anoplophora glabripennis] | 91 | 3 |
| Gene.5402 | PREDICTED: transmembrane emp24 domain-containing protein eca-like [Nicrophorus vespilloides] | 145 | 3 |
| Gene.5404 | prefoldin subunit 2 [Anoplophora glabripennis] | 104 | 3 |
| Gene.5416 | PREDICTED: proteasome activator complex subunit 3 isoform X2 [Tribolium castaneum] | 11 | 3 |
| Gene.5416 | PREDICTED: proteasome activator complex subunit 3 isoform X2 [Tribolium castaneum] | 183 | 3 |
| Gene.5420 | proteasome subunit beta type-4 [Leptinotarsa decemlineata] | 194 | 3 |
| Gene.5420 | proteasome subunit beta type-4 [Leptinotarsa decemlineata] | 108 | 3 |
| Gene.5435 | putative GABA-A receptor associated protein [Graphocephala atropunctata] | 35 | 3 |
| Gene.545 | beta-1,3-glucan-binding protein-like [Anoplophora glabripennis] | 158 | 3 |
| Gene.5465 | PREDICTED: peptidyl-prolyl cis-trans isomerase NIMA-interacting 1 [Tribolium castaneum] | 67 | 3 |
| Gene.5475 | PREDICTED: transmembrane protein 177 [Tribolium castaneum] | 253 | 3 |
| Gene.5478 | 60S ribosomal protein L7a-like [Leptinotarsa decemlineata] | 123 | 3 |
| Gene.5478 | 60S ribosomal protein L7a-like [Leptinotarsa decemlineata] | 50 | 3 |
| Gene.5478 | 60S ribosomal protein L7a-like [Leptinotarsa decemlineata] | 228 | 3 |
| Gene.5478 | 60S ribosomal protein L7a-like [Leptinotarsa decemlineata] | 36 | 3 |
| Gene.5478 | 60S ribosomal protein L7a-like [Leptinotarsa decemlineata] | 112 | 3 |
| Gene.5478 | 60S ribosomal protein L7a-like [Leptinotarsa decemlineata] | 22 | 3 |
| Gene.5478 | 60S ribosomal protein L7a-like [Leptinotarsa decemlineata] | 99 | 3 |
| Gene.5478 | 60S ribosomal protein L7a-like [Leptinotarsa decemlineata] | 150 | 3 |
| Gene.5478 | 60S ribosomal protein L7a-like [Leptinotarsa decemlineata] | 222 | 3 |
| Gene.548 | clavesin-1 isoform X1 [Agrilus planipennis] | 70 | 3 |
| Gene.548 | clavesin-1 isoform X1 [Agrilus planipennis] | 37 | 3 |
| Gene.548 | clavesin-1 isoform X1 [Agrilus planipennis] | 117 | 3 |
| Gene.5504 | chemosensory protein CSP9 [Tenebrio molitor] | 134 | 3 |
| Gene.5504 | chemosensory protein CSP9 [Tenebrio molitor] | 129 | 3 |
| Gene.5504 | chemosensory protein CSP9 [Tenebrio molitor] | 48 | 3 |
| Gene.5504 | chemosensory protein CSP9 [Tenebrio molitor] | 107 | 3 |
| Gene.5504 | chemosensory protein CSP9 [Tenebrio molitor] | 112 | 3 |
| Gene.5504 | chemosensory protein CSP9 [Tenebrio molitor] | 87 | 3 |
| Gene.5504 | chemosensory protein CSP9 [Tenebrio molitor] | 125 | 3 |
| Gene.5504 | chemosensory protein CSP9 [Tenebrio molitor] | 117 | 3 |
| Gene.5511 | PREDICTED: nucleoplasmin-like protein [Aethina tumida] | 35 | 3 |
| Gene.5511 | PREDICTED: nucleoplasmin-like protein [Aethina tumida] | 15 | 3 |
| Gene.5516 | upstream activation factor subunit spp27 [Agrilus planipennis] | 222 | 3 |
| Gene.5521 | PREDICTED: probable 39S ribosomal protein L23, mitochondrial [Tribolium castaneum] | 87 | 3 |
| Gene.5527 | dihydrofolate reductase [Asbolus verrucosus] | 139 | 3 |
| Gene.5542 | catalase [Onthophagus taurus] | 28 | 3 |
| Gene.5542 | catalase [Onthophagus taurus] | 179 | 3 |
| Gene.5542 | catalase [Onthophagus taurus] | 325 | 3 |
| Gene.5542 | catalase [Onthophagus taurus] | 48 | 3 |
| Gene.5542 | catalase [Onthophagus taurus] | 438 | 3 |
| Gene.5542 | catalase [Onthophagus taurus] | 247 | 3 |
| Gene.5545 | probable nuclear transport factor 2 isoform X1 [Agrilus planipennis] | 66 | 3 |
| Gene.5548 | coatomer subunit zeta-1 [Onthophagus taurus] | 40 | 3 |
| Gene.5561 | stomatin-like protein 2, mitochondrial [Anoplophora glabripennis] | 149 | 3 |
| Gene.5561 | stomatin-like protein 2, mitochondrial [Anoplophora glabripennis] | 335 | 3 |
| Gene.5561 | stomatin-like protein 2, mitochondrial [Anoplophora glabripennis] | 144 | 3 |
| Gene.5566 | i-type lysozyme 3 [Harmonia axyridis] | 79 | 3 |
| Gene.5580 | proteasome subunit alpha type-4 [Anoplophora glabripennis] | 205 | 3 |
| Gene.5602 | 60S ribosomal protein L13 [Anoplophora glabripennis] | 123 | 3 |
| Gene.5602 | 60S ribosomal protein L13 [Anoplophora glabripennis] | 132 | 3 |
| Gene.5602 | 60S ribosomal protein L13 [Anoplophora glabripennis] | 82 | 3 |
| Gene.5607 | sorting nexin-12 [Asbolus verrucosus] | 66 | 3 |
| Gene.5612 | translin [Leptinotarsa decemlineata] | 190 | 3 |
| Gene.5625 | ribosomal protein L32e [Hister sp. APV-2005] | 80 | 3 |
| Gene.5625 | ribosomal protein L32e [Hister sp. APV-2005] | 44 | 3 |
| Gene.5628 | PREDICTED: eukaryotic translation initiation factor 4H [Tribolium castaneum] | 65 | 3 |
| Gene.563 | PREDICTED: importin-5 [Tribolium castaneum] | 507 | 3 |
| Gene.5631 | U1 small nuclear ribonucleoprotein A [Leptinotarsa decemlineata] | 111 | 3 |
| Gene.5634 | PREDICTED: proteasome subunit beta type-6 [Tribolium castaneum] | 194 | 3 |
| Gene.5636 | ras-related protein Rab-11A [Anoplophora glabripennis] | 179 | 3 |
| Gene.5641 | PREDICTED: 40S ribosomal protein S21 [Tribolium castaneum] | 102 | 3 |
| Gene.5641 | PREDICTED: 40S ribosomal protein S21 [Tribolium castaneum] | 79 | 3 |
| Gene.5672 | isopentenyl-diphosphate Delta-isomerase 1 [Leptinotarsa decemlineata] | 197 | 3 |
| Gene.5672 | isopentenyl-diphosphate Delta-isomerase 1 [Leptinotarsa decemlineata] | 174 | 3 |
| Gene.5672 | isopentenyl-diphosphate Delta-isomerase 1 [Leptinotarsa decemlineata] | 72 | 3 |
| Gene.5672 | isopentenyl-diphosphate Delta-isomerase 1 [Leptinotarsa decemlineata] | 260 | 3 |
| Gene.5672 | isopentenyl-diphosphate Delta-isomerase 1 [Leptinotarsa decemlineata] | 65 | 3 |
| Gene.5675 | PREDICTED: secretory carrier-associated membrane protein 1 [Tribolium castaneum] | 304 | 3 |
| Gene.5705 | PREDICTED: LOW QUALITY PROTEIN: uncharacterized protein LOC109596860 [Aethina tumida] | 609 | 3 |
| Gene.5709 | cAMP-dependent protein kinase catalytic subunit [Agrilus planipennis] | 26 | 3 |
| Gene.5709 | cAMP-dependent protein kinase catalytic subunit [Agrilus planipennis] | 257 | 3 |
| Gene.5711 | proteasome subunit alpha type-7-1 [Anoplophora glabripennis] | 238 | 3 |
| Gene.5711 | proteasome subunit alpha type-7-1 [Anoplophora glabripennis] | 187 | 3 |
| Gene.5711 | proteasome subunit alpha type-7-1 [Anoplophora glabripennis] | 54 | 3 |
| Gene.5711 | proteasome subunit alpha type-7-1 [Anoplophora glabripennis] | 176 | 3 |
| Gene.572 | PREDICTED: phosphoglycerate kinase [Tribolium castaneum] | 84 | 3 |
| Gene.572 | PREDICTED: phosphoglycerate kinase [Tribolium castaneum] | 129 | 3 |
| Gene.572 | PREDICTED: phosphoglycerate kinase [Tribolium castaneum] | 323 | 3 |
| Gene.572 | PREDICTED: phosphoglycerate kinase [Tribolium castaneum] | 89 | 3 |
| Gene.572 | PREDICTED: phosphoglycerate kinase [Tribolium castaneum] | 10 | 3 |
| Gene.572 | PREDICTED: phosphoglycerate kinase [Tribolium castaneum] | 351 | 3 |
| Gene.572 | PREDICTED: phosphoglycerate kinase [Tribolium castaneum] | 16 | 3 |
| Gene.572 | PREDICTED: phosphoglycerate kinase [Tribolium castaneum] | 5 | 3 |
| Gene.5733 | PREDICTED: prohibitin-2 isoform X2 [Tribolium castaneum] | 237 | 3 |
| Gene.5733 | PREDICTED: prohibitin-2 isoform X2 [Tribolium castaneum] | 251 | 3 |
| Gene.5733 | PREDICTED: prohibitin-2 isoform X2 [Tribolium castaneum] | 201 | 3 |
| Gene.5733 | PREDICTED: prohibitin-2 isoform X2 [Tribolium castaneum] | 148 | 3 |
| Gene.5733 | PREDICTED: prohibitin-2 isoform X2 [Tribolium castaneum] | 117 | 3 |
| Gene.5733 | PREDICTED: prohibitin-2 isoform X2 [Tribolium castaneum] | 62 | 3 |
| Gene.5734 | electron transfer flavoprotein subunit alpha, mitochondrial, partial [Asbolus verrucosus] | 296 | 3 |
| Gene.5734 | electron transfer flavoprotein subunit alpha, mitochondrial, partial [Asbolus verrucosus] | 46 | 3 |
| Gene.5734 | electron transfer flavoprotein subunit alpha, mitochondrial, partial [Asbolus verrucosus] | 70 | 3 |
| Gene.5734 | electron transfer flavoprotein subunit alpha, mitochondrial, partial [Asbolus verrucosus] | 234 | 3 |
| Gene.5734 | electron transfer flavoprotein subunit alpha, mitochondrial, partial [Asbolus verrucosus] | 228 | 3 |
| Gene.5734 | electron transfer flavoprotein subunit alpha, mitochondrial, partial [Asbolus verrucosus] | 208 | 3 |
| Gene.5734 | electron transfer flavoprotein subunit alpha, mitochondrial, partial [Asbolus verrucosus] | 331 | 3 |
| Gene.5739 | PREDICTED: LOW QUALITY PROTEIN: mitochondrial import inner membrane translocase subunit TIM50-C-like [Aethina tumida] | 341 | 3 |
| Gene.5741 | 26S proteasome non-ATPase regulatory subunit 8 [Anoplophora glabripennis] | 11 | 3 |
| Gene.5746 | PREDICTED: ATP synthase subunit delta, mitochondrial [Tribolium castaneum] | 137 | 3 |
| Gene.5762 | PREDICTED: transmembrane protein 256 homolog [Tribolium castaneum] | 56 | 3 |
| Gene.5767 | NADH dehydrogenase [ubiquinone] iron-sulfur protein 5 [Anoplophora glabripennis] | 89 | 3 |
| Gene.5768 | PREDICTED: F-actin-capping protein subunit beta [Tribolium castaneum] | 235 | 3 |
| Gene.5769 | casein kinase II subunit alpha isoform X1 [Pogonomyrmex barbatus] | 244 | 3 |
| Gene.580 | PREDICTED: cytochrome b-c1 complex subunit 2, mitochondrial [Tribolium castaneum] | 231 | 3 |
| Gene.580 | PREDICTED: cytochrome b-c1 complex subunit 2, mitochondrial [Tribolium castaneum] | 248 | 3 |
| Gene.5801 | SUMO-activating enzyme subunit 2 [Anoplophora glabripennis] | 337 | 3 |
| Gene.5801 | SUMO-activating enzyme subunit 2 [Anoplophora glabripennis] | 531 | 3 |
| Gene.5803 | calcium-binding protein E63-1 [Anoplophora glabripennis] | 20 | 3 |
| Gene.5806 | PREDICTED: protein deglycase DJ-1 isoform X1 [Dendroctonus ponderosae] | 92 | 3 |
| Gene.5806 | PREDICTED: protein deglycase DJ-1 isoform X1 [Dendroctonus ponderosae] | 141 | 3 |
| Gene.5806 | PREDICTED: protein deglycase DJ-1 isoform X1 [Dendroctonus ponderosae] | 89 | 3 |
| Gene.5815 | PREDICTED: uncharacterized protein LOC663029 [Tribolium castaneum] | 276 | 3 |
| Gene.5815 | PREDICTED: uncharacterized protein LOC663029 [Tribolium castaneum] | 279 | 3 |
| Gene.5815 | PREDICTED: uncharacterized protein LOC663029 [Tribolium castaneum] | 410 | 3 |
| Gene.5822 | probable 28S ribosomal protein S6, mitochondrial isoform X1 [Onthophagus taurus] | 104 | 3 |
| Gene.5822 | probable 28S ribosomal protein S6, mitochondrial isoform X1 [Onthophagus taurus] | 46 | 3 |
| Gene.5825 | PREDICTED: bis(5'-nucleosyl)-tetraphosphatase [asymmetrical]-like [Dendroctonus ponderosae] | 65 | 3 |
| Gene.5835 | translocon-associated protein subunit delta-like [Anoplophora glabripennis] | 97 | 3 |
| Gene.5837 | NHP2-like protein 1 [Agrilus planipennis] | 43 | 3 |
| Gene.5844 | phosphoglycerate mutase 1 [Asbolus verrucosus] | 181 | 3 |
| Gene.5844 | phosphoglycerate mutase 1 [Asbolus verrucosus] | 165 | 3 |
| Gene.5844 | phosphoglycerate mutase 1 [Asbolus verrucosus] | 122 | 3 |
| Gene.5844 | phosphoglycerate mutase 1 [Asbolus verrucosus] | 68 | 3 |
| Gene.5844 | phosphoglycerate mutase 1 [Asbolus verrucosus] | 105 | 3 |
| Gene.5844 | phosphoglycerate mutase 1 [Asbolus verrucosus] | 176 | 3 |
| Gene.5850 | PREDICTED: hrp65 protein-like [Aethina tumida] | 332 | 3 |
| Gene.5850 | PREDICTED: hrp65 protein-like [Aethina tumida] | 323 | 3 |
| Gene.5850 | PREDICTED: hrp65 protein-like [Aethina tumida] | 157 | 3 |
| Gene.5850 | PREDICTED: hrp65 protein-like [Aethina tumida] | 300 | 3 |
| Gene.5857 | PREDICTED: RNA-binding protein Rsf1 [Tribolium castaneum] | 33 | 3 |
| Gene.5857 | PREDICTED: RNA-binding protein Rsf1 [Tribolium castaneum] | 7 | 3 |
| Gene.5857 | PREDICTED: RNA-binding protein Rsf1 [Tribolium castaneum] | 23 | 3 |
| Gene.5862 | V-type proton ATPase subunit F, partial [Asbolus verrucosus] | 8 | 3 |
| Gene.5862 | V-type proton ATPase subunit F, partial [Asbolus verrucosus] | 106 | 3 |
| Gene.5867 | PREDICTED: grpE protein homolog, mitochondrial-like [Aethina tumida] | 186 | 3 |
| Gene.5867 | PREDICTED: grpE protein homolog, mitochondrial-like [Aethina tumida] | 150 | 3 |
| Gene.5868 | PREDICTED: V-type proton ATPase subunit D [Polistes canadensis] | 50 | 3 |
| Gene.5869 | serine/threonine-protein phosphatase 5 [Anoplophora glabripennis] | 105 | 3 |
| Gene.5885 | NADH dehydrogenase [ubiquinone] 1 alpha subcomplex subunit 6 [Onthophagus taurus] | 94 | 3 |
| Gene.5898 | PREDICTED: 60S ribosomal protein L36 [Tribolium castaneum] | 4 | 3 |
| Gene.5898 | PREDICTED: 60S ribosomal protein L36 [Tribolium castaneum] | 64 | 3 |
| Gene.5899 | proteasome subunit beta type-3 [Anoplophora glabripennis] | 77 | 3 |
| Gene.5900 | PREDICTED: eukaryotic initiation factor 4A-III [Aethina tumida] | 196 | 3 |
| Gene.5903 | Z9 acyl-CoA desaturase B [Tribolium castaneum] | 309 | 3 |
| Gene.5904 | eukaryotic translation initiation factor 2 subunit 2 [Asbolus verrucosus] | 174 | 3 |
| Gene.5910 | PREDICTED: NADH dehydrogenase [ubiquinone] 1 alpha subcomplex subunit 10, mitochondrial [Tribolium castaneum] | 270 | 3 |
| Gene.5928 | PREDICTED: 40S ribosomal protein S3a [Aethina tumida] | 207 | 3 |
| Gene.5928 | PREDICTED: 40S ribosomal protein S3a [Aethina tumida] | 194 | 3 |
| Gene.5928 | PREDICTED: 40S ribosomal protein S3a [Aethina tumida] | 97 | 3 |
| Gene.5928 | PREDICTED: 40S ribosomal protein S3a [Aethina tumida] | 199 | 3 |
| Gene.5928 | PREDICTED: 40S ribosomal protein S3a [Aethina tumida] | 249 | 3 |
| Gene.5928 | PREDICTED: 40S ribosomal protein S3a [Aethina tumida] | 121 | 3 |
| Gene.5928 | PREDICTED: 40S ribosomal protein S3a [Aethina tumida] | 211 | 3 |
| Gene.5928 | PREDICTED: 40S ribosomal protein S3a [Aethina tumida] | 174 | 3 |
| Gene.5928 | PREDICTED: 40S ribosomal protein S3a [Aethina tumida] | 72 | 3 |
| Gene.5928 | PREDICTED: 40S ribosomal protein S3a [Aethina tumida] | 43 | 3 |
| Gene.5928 | PREDICTED: 40S ribosomal protein S3a [Aethina tumida] | 55 | 3 |
| Gene.5941 | PREDICTED: LOW QUALITY PROTEIN: 26S proteasome non-ATPase regulatory subunit 13 [Aethina tumida] | 141 | 3 |
| Gene.595 | troponin T, skeletal muscle isoform X5 [Leptinotarsa decemlineata] | 170 | 3 |
| Gene.595 | troponin T, skeletal muscle isoform X5 [Leptinotarsa decemlineata] | 124 | 3 |
| Gene.595 | troponin T, skeletal muscle isoform X5 [Leptinotarsa decemlineata] | 85 | 3 |
| Gene.595 | troponin T, skeletal muscle isoform X5 [Leptinotarsa decemlineata] | 36 | 3 |
| Gene.595 | troponin T, skeletal muscle isoform X5 [Leptinotarsa decemlineata] | 139 | 3 |
| Gene.595 | troponin T, skeletal muscle isoform X5 [Leptinotarsa decemlineata] | 226 | 3 |
| Gene.595 | troponin T, skeletal muscle isoform X5 [Leptinotarsa decemlineata] | 176 | 3 |
| Gene.595 | troponin T, skeletal muscle isoform X5 [Leptinotarsa decemlineata] | 60 | 3 |
| Gene.595 | troponin T, skeletal muscle isoform X5 [Leptinotarsa decemlineata] | 212 | 3 |
| Gene.595 | troponin T, skeletal muscle isoform X5 [Leptinotarsa decemlineata] | 105 | 3 |
| Gene.5965 | PREDICTED: superoxide dismutase [Mn] 1, mitochondrial-like [Aethina tumida] | 63 | 3 |
| Gene.5965 | PREDICTED: superoxide dismutase [Mn] 1, mitochondrial-like [Aethina tumida] | 68 | 3 |
| Gene.5969 | Ribose-phosphate pyrophosphokinase 1-like Protein [Tribolium castaneum] | 151 | 3 |
| Gene.5969 | Ribose-phosphate pyrophosphokinase 1-like Protein [Tribolium castaneum] | 263 | 3 |
| Gene.5973 | ribosomal protein S13, partial [Harmonia axyridis] | 27 | 3 |
| Gene.5973 | ribosomal protein S13, partial [Harmonia axyridis] | 34 | 3 |
| Gene.5973 | ribosomal protein S13, partial [Harmonia axyridis] | 70 | 3 |
| Gene.5973 | ribosomal protein S13, partial [Harmonia axyridis] | 9 | 3 |
| Gene.5975 | mitochondrial import inner membrane translocase subunit TIM44 [Anoplophora glabripennis] | 67 | 3 |
| Gene.5975 | mitochondrial import inner membrane translocase subunit TIM44 [Anoplophora glabripennis] | 16 | 3 |
| Gene.5990 | PREDICTED: LOW QUALITY PROTEIN: enoyl-CoA delta isomerase 1, mitochondrial-like [Aethina tumida] | 243 | 3 |
| Gene.5990 | PREDICTED: LOW QUALITY PROTEIN: enoyl-CoA delta isomerase 1, mitochondrial-like [Aethina tumida] | 73 | 3 |
| Gene.5992 | PREDICTED: GTP:AMP phosphotransferase AK3, mitochondrial [Tribolium castaneum] | 163 | 3 |
| Gene.5992 | PREDICTED: GTP:AMP phosphotransferase AK3, mitochondrial [Tribolium castaneum] | 205 | 3 |
| Gene.5992 | PREDICTED: GTP:AMP phosphotransferase AK3, mitochondrial [Tribolium castaneum] | 52 | 3 |
| Gene.5992 | PREDICTED: GTP:AMP phosphotransferase AK3, mitochondrial [Tribolium castaneum] | 216 | 3 |
| Gene.5992 | PREDICTED: GTP:AMP phosphotransferase AK3, mitochondrial [Tribolium castaneum] | 224 | 3 |
| Gene.5992 | PREDICTED: GTP:AMP phosphotransferase AK3, mitochondrial [Tribolium castaneum] | 188 | 3 |
| Gene.5992 | PREDICTED: GTP:AMP phosphotransferase AK3, mitochondrial [Tribolium castaneum] | 167 | 3 |
| Gene.5994 | PREDICTED: LOW QUALITY PROTEIN: histidine triad nucleotide-binding protein 1-like [Aethina tumida] | 53 | 3 |
| Gene.5994 | PREDICTED: LOW QUALITY PROTEIN: histidine triad nucleotide-binding protein 1-like [Aethina tumida] | 64 | 3 |
| Gene.6001 | proteasome subunit alpha type-6 [Anoplophora glabripennis] | 181 | 3 |
| Gene.6001 | proteasome subunit alpha type-6 [Anoplophora glabripennis] | 171 | 3 |
| Gene.6011 | F-actin-capping protein subunit alpha [Anoplophora glabripennis] | 202 | 3 |
| Gene.6014 | PREDICTED: ubiquitin-fold modifier-conjugating enzyme 1 [Tribolium castaneum] | 29 | 3 |
| Gene.6019 | PREDICTED: membrane-associated progesterone receptor component 1 [Tribolium castaneum] | 77 | 3 |
| Gene.6019 | PREDICTED: membrane-associated progesterone receptor component 1 [Tribolium castaneum] | 61 | 3 |
| Gene.6019 | PREDICTED: membrane-associated progesterone receptor component 1 [Tribolium castaneum] | 157 | 3 |
| Gene.6024 | peroxiredoxin 1-like [Leptinotarsa decemlineata] | 186 | 3 |
| Gene.6024 | peroxiredoxin 1-like [Leptinotarsa decemlineata] | 178 | 3 |
| Gene.6024 | peroxiredoxin 1-like [Leptinotarsa decemlineata] | 105 | 3 |
| Gene.6024 | peroxiredoxin 1-like [Leptinotarsa decemlineata] | 133 | 3 |
| Gene.6024 | peroxiredoxin 1-like [Leptinotarsa decemlineata] | 14 | 3 |
| Gene.6033 | uncharacterized protein LOC108740116 [Agrilus planipennis] | 8 | 3 |
| Gene.6041 | eukaryotic translation initiation factor 3 subunit L [Anoplophora glabripennis] | 365 | 3 |
| Gene.6077 | Spectrin, SH3 1, and/or DUF3584 domain containing protein, partial [Asbolus verrucosus] | 8 | 3 |
| Gene.6077 | Spectrin, SH3 1, and/or DUF3584 domain containing protein, partial [Asbolus verrucosus] | 11 | 3 |
| Gene.6077 | Spectrin, SH3 1, and/or DUF3584 domain containing protein, partial [Asbolus verrucosus] | 34 | 3 |
| Gene.6077 | Spectrin, SH3 1, and/or DUF3584 domain containing protein, partial [Asbolus verrucosus] | 85 | 3 |
| Gene.6077 | Spectrin, SH3 1, and/or DUF3584 domain containing protein, partial [Asbolus verrucosus] | 77 | 3 |
| Gene.6087 | calumenin [Asbolus verrucosus] | 68 | 3 |
| Gene.6087 | calumenin [Asbolus verrucosus] | 235 | 3 |
| Gene.6087 | calumenin [Asbolus verrucosus] | 82 | 3 |
| Gene.609 | tropomodulin-1 isoform X5 [Leptinotarsa decemlineata] | 239 | 3 |
| Gene.609 | tropomodulin-1 isoform X5 [Leptinotarsa decemlineata] | 242 | 3 |
| Gene.6095 | PREDICTED: 12 kDa FK506-binding protein [Tribolium castaneum] | 67 | 3 |
| Gene.6110 | PREDICTED: probable very-long-chain enoyl-CoA reductase art-1 [Aethina tumida] | 64 | 3 |
| Gene.6110 | PREDICTED: probable very-long-chain enoyl-CoA reductase art-1 [Aethina tumida] | 289 | 3 |
| Gene.6110 | PREDICTED: probable very-long-chain enoyl-CoA reductase art-1 [Aethina tumida] | 37 | 3 |
| Gene.6119 | PREDICTED: maternal protein exuperantia-1 [Tribolium castaneum] | 95 | 3 |
| Gene.6119 | PREDICTED: maternal protein exuperantia-1 [Tribolium castaneum] | 160 | 3 |
| Gene.6119 | PREDICTED: maternal protein exuperantia-1 [Tribolium castaneum] | 263 | 3 |
| Gene.6119 | PREDICTED: maternal protein exuperantia-1 [Tribolium castaneum] | 20 | 3 |
| Gene.6119 | PREDICTED: maternal protein exuperantia-1 [Tribolium castaneum] | 302 | 3 |
| Gene.6119 | PREDICTED: maternal protein exuperantia-1 [Tribolium castaneum] | 312 | 3 |
| Gene.6119 | PREDICTED: maternal protein exuperantia-1 [Tribolium castaneum] | 317 | 3 |
| Gene.6119 | PREDICTED: maternal protein exuperantia-1 [Tribolium castaneum] | 201 | 3 |
| Gene.6119 | PREDICTED: maternal protein exuperantia-1 [Tribolium castaneum] | 219 | 3 |
| Gene.6119 | PREDICTED: maternal protein exuperantia-1 [Tribolium castaneum] | 320 | 3 |
| Gene.6119 | PREDICTED: maternal protein exuperantia-1 [Tribolium castaneum] | 101 | 3 |
| Gene.6119 | PREDICTED: maternal protein exuperantia-1 [Tribolium castaneum] | 29 | 3 |
| Gene.6128 | protein HGV2-like isoform X1 [Leptinotarsa decemlineata] | 306 | 3 |
| Gene.6128 | protein HGV2-like isoform X1 [Leptinotarsa decemlineata] | 30 | 3 |
| Gene.6128 | protein HGV2-like isoform X1 [Leptinotarsa decemlineata] | 270 | 3 |
| Gene.6131 | ruvB-like 2 [Asbolus verrucosus] | 394 | 3 |
| Gene.6135 | PREDICTED: NADH dehydrogenase [ubiquinone] 1 alpha subcomplex subunit 13-like [Aethina tumida] | 95 | 3 |
| Gene.6145 | PREDICTED: pentatricopeptide repeat-containing protein 2, mitochondrial [Tribolium castaneum] | 84 | 3 |
| Gene.6145 | PREDICTED: pentatricopeptide repeat-containing protein 2, mitochondrial [Tribolium castaneum] | 58 | 3 |
| Gene.6165 | 40S ribosomal protein S20 [Pogonomyrmex barbatus] | 38 | 3 |
| Gene.6165 | 40S ribosomal protein S20 [Pogonomyrmex barbatus] | 60 | 3 |
| Gene.6165 | 40S ribosomal protein S20 [Pogonomyrmex barbatus] | 4 | 3 |
| Gene.6165 | 40S ribosomal protein S20 [Pogonomyrmex barbatus] | 76 | 3 |
| Gene.6167 | PREDICTED: 39S ribosomal protein L12, mitochondrial [Tribolium castaneum] | 137 | 3 |
| Gene.6167 | PREDICTED: 39S ribosomal protein L12, mitochondrial [Tribolium castaneum] | 168 | 3 |
| Gene.6167 | PREDICTED: 39S ribosomal protein L12, mitochondrial [Tribolium castaneum] | 153 | 3 |
| Gene.6169 | cytochrome b-c1 complex subunit 7-like [Anoplophora glabripennis] | 77 | 3 |
| Gene.6169 | cytochrome b-c1 complex subunit 7-like [Anoplophora glabripennis] | 53 | 3 |
| Gene.6177 | CLUMA_CG005021, isoform A [Clunio marinus] | 48 | 3 |
| Gene.6181 | PREDICTED: m-AAA protease-interacting protein 1, mitochondrial [Aethina tumida] | 237 | 3 |
| Gene.6185 | PREDICTED: ancylostoma secreted protein-like [Aethina tumida] | 70 | 3 |
| Gene.6192 | PREDICTED: ecdysteroid-regulated 16 kDa protein [Tribolium castaneum] | 142 | 3 |
| Gene.6194 | PREDICTED: acidic leucine-rich nuclear phosphoprotein 32 family member A [Tribolium castaneum] | 94 | 3 |
| Gene.6194 | PREDICTED: acidic leucine-rich nuclear phosphoprotein 32 family member A [Tribolium castaneum] | 138 | 3 |
| Gene.6194 | PREDICTED: acidic leucine-rich nuclear phosphoprotein 32 family member A [Tribolium castaneum] | 132 | 3 |
| Gene.6194 | PREDICTED: acidic leucine-rich nuclear phosphoprotein 32 family member A [Tribolium castaneum] | 146 | 3 |
| Gene.6194 | PREDICTED: acidic leucine-rich nuclear phosphoprotein 32 family member A [Tribolium castaneum] | 96 | 3 |
| Gene.6196 | PREDICTED: protein l(2)37Cc [Dendroctonus ponderosae] | 196 | 3 |
| Gene.6196 | PREDICTED: protein l(2)37Cc [Dendroctonus ponderosae] | 187 | 3 |
| Gene.6196 | PREDICTED: protein l(2)37Cc [Dendroctonus ponderosae] | 150 | 3 |
| Gene.6197 | ribosomal protein L11e [Curculio glandium] | 8 | 3 |
| Gene.6197 | ribosomal protein L11e [Curculio glandium] | 173 | 3 |
| Gene.6197 | ribosomal protein L11e [Curculio glandium] | 178 | 3 |
| Gene.6197 | ribosomal protein L11e [Curculio glandium] | 187 | 3 |
| Gene.6197 | ribosomal protein L11e [Curculio glandium] | 168 | 3 |
| Gene.6197 | ribosomal protein L11e [Curculio glandium] | 47 | 3 |
| Gene.6221 | PREDICTED: histone H3.3-like [Takifugu rubripes] | 123 | 3 |
| Gene.6221 | PREDICTED: histone H3.3-like [Takifugu rubripes] | 80 | 3 |
| Gene.6222 | Pro isomerase domain containing protein [Asbolus verrucosus] | 42 | 3 |
| Gene.6222 | Pro isomerase domain containing protein [Asbolus verrucosus] | 120 | 3 |
| Gene.6222 | Pro isomerase domain containing protein [Asbolus verrucosus] | 89 | 3 |
| Gene.6222 | Pro isomerase domain containing protein [Asbolus verrucosus] | 73 | 3 |
| Gene.6222 | Pro isomerase domain containing protein [Asbolus verrucosus] | 68 | 3 |
| Gene.6222 | Pro isomerase domain containing protein [Asbolus verrucosus] | 100 | 3 |
| Gene.6234 | proteasome subunit alpha type-3 [Anoplophora glabripennis] | 57 | 3 |
| Gene.624 | PREDICTED: staphylococcal nuclease domain-containing protein 1 [Tribolium castaneum] | 578 | 3 |
| Gene.624 | PREDICTED: staphylococcal nuclease domain-containing protein 1 [Tribolium castaneum] | 877 | 3 |
| Gene.624 | PREDICTED: staphylococcal nuclease domain-containing protein 1 [Tribolium castaneum] | 712 | 3 |
| Gene.624 | PREDICTED: staphylococcal nuclease domain-containing protein 1 [Tribolium castaneum] | 751 | 3 |
| Gene.624 | PREDICTED: staphylococcal nuclease domain-containing protein 1 [Tribolium castaneum] | 168 | 3 |
| Gene.624 | PREDICTED: staphylococcal nuclease domain-containing protein 1 [Tribolium castaneum] | 331 | 3 |
| Gene.624 | PREDICTED: staphylococcal nuclease domain-containing protein 1 [Tribolium castaneum] | 315 | 3 |
| Gene.624 | PREDICTED: staphylococcal nuclease domain-containing protein 1 [Tribolium castaneum] | 814 | 3 |
| Gene.624 | PREDICTED: staphylococcal nuclease domain-containing protein 1 [Tribolium castaneum] | 871 | 3 |
| Gene.624 | PREDICTED: staphylococcal nuclease domain-containing protein 1 [Tribolium castaneum] | 642 | 3 |
| Gene.624 | PREDICTED: staphylococcal nuclease domain-containing protein 1 [Tribolium castaneum] | 459 | 3 |
| Gene.6249 | RRS1 domain containing protein [Asbolus verrucosus] | 227 | 3 |
| Gene.6252 | NADH dehydrogenase [ubiquinone] 1 beta subcomplex subunit 4 [Leptinotarsa decemlineata] | 60 | 3 |
| Gene.6252 | NADH dehydrogenase [ubiquinone] 1 beta subcomplex subunit 4 [Leptinotarsa decemlineata] | 111 | 3 |
| Gene.6254 | ubiquitin carboxyl-terminal hydrolase 14 [Leptinotarsa decemlineata] | 363 | 3 |
| Gene.6254 | ubiquitin carboxyl-terminal hydrolase 14 [Leptinotarsa decemlineata] | 226 | 3 |
| Gene.6254 | ubiquitin carboxyl-terminal hydrolase 14 [Leptinotarsa decemlineata] | 62 | 3 |
| Gene.6254 | ubiquitin carboxyl-terminal hydrolase 14 [Leptinotarsa decemlineata] | 377 | 3 |
| Gene.6254 | ubiquitin carboxyl-terminal hydrolase 14 [Leptinotarsa decemlineata] | 54 | 3 |
| Gene.6262 | probable NADH dehydrogenase [ubiquinone] flavoprotein 2, mitochondrial [Leptinotarsa decemlineata] | 104 | 3 |
| Gene.6262 | probable NADH dehydrogenase [ubiquinone] flavoprotein 2, mitochondrial [Leptinotarsa decemlineata] | 230 | 3 |
| Gene.6281 | importin subunit beta-1 isoform X1 [Anoplophora glabripennis] | 216 | 3 |
| Gene.6284 | NifU N domain containing protein [Asbolus verrucosus] | 82 | 3 |
| Gene.6285 | PREDICTED: protein krasavietz isoform X1 [Aethina tumida] | 238 | 3 |
| Gene.6292 | PREDICTED: single-stranded DNA-binding protein, mitochondrial [Tribolium castaneum] | 32 | 3 |
| Gene.6292 | PREDICTED: single-stranded DNA-binding protein, mitochondrial [Tribolium castaneum] | 100 | 3 |
| Gene.6297 | PREDICTED: V-type proton ATPase subunit C isoform X3 [Tribolium castaneum] | 155 | 3 |
| Gene.6297 | PREDICTED: V-type proton ATPase subunit C isoform X3 [Tribolium castaneum] | 139 | 3 |
| Gene.6297 | PREDICTED: V-type proton ATPase subunit C isoform X3 [Tribolium castaneum] | 267 | 3 |
| Gene.6297 | PREDICTED: V-type proton ATPase subunit C isoform X3 [Tribolium castaneum] | 262 | 3 |
| Gene.6298 | glutaredoxin-C4-like [Asbolus verrucosus] | 92 | 3 |
| Gene.6298 | glutaredoxin-C4-like [Asbolus verrucosus] | 87 | 3 |
| Gene.6298 | glutaredoxin-C4-like [Asbolus verrucosus] | 33 | 3 |
| Gene.6298 | glutaredoxin-C4-like [Asbolus verrucosus] | 97 | 3 |
| Gene.6298 | glutaredoxin-C4-like [Asbolus verrucosus] | 10 | 3 |
| Gene.6306 | serine-arginine protein 55-like [Asbolus verrucosus] | 60 | 3 |
| Gene.6306 | serine-arginine protein 55-like [Asbolus verrucosus] | 177 | 3 |
| Gene.6306 | serine-arginine protein 55-like [Asbolus verrucosus] | 122 | 3 |
| Gene.6306 | serine-arginine protein 55-like [Asbolus verrucosus] | 160 | 3 |
| Gene.6318 | NADH dehydrogenase [ubiquinone] 1 beta subcomplex subunit 3 [Leptinotarsa decemlineata] | 57 | 3 |
| Gene.6318 | NADH dehydrogenase [ubiquinone] 1 beta subcomplex subunit 3 [Leptinotarsa decemlineata] | 23 | 3 |
| Gene.6318 | NADH dehydrogenase [ubiquinone] 1 beta subcomplex subunit 3 [Leptinotarsa decemlineata] | 43 | 3 |
| Gene.6325 | uncharacterized protein Dvir_GJ22112 [Drosophila virilis] | 67 | 3 |
| Gene.6325 | uncharacterized protein Dvir_GJ22112 [Drosophila virilis] | 26 | 3 |
| Gene.6326 | RRM 1 domain containing protein [Asbolus verrucosus] | 13 | 3 |
| Gene.6339 | PREDICTED: nucleoside diphosphate kinase isoform X1 [Tribolium castaneum] | 50 | 3 |
| Gene.6339 | PREDICTED: nucleoside diphosphate kinase isoform X1 [Tribolium castaneum] | 28 | 3 |
| Gene.6339 | PREDICTED: nucleoside diphosphate kinase isoform X1 [Tribolium castaneum] | 143 | 3 |
| Gene.6339 | PREDICTED: nucleoside diphosphate kinase isoform X1 [Tribolium castaneum] | 47 | 3 |
| Gene.6348 | myosin heavy chain, muscle isoform X35 [Onthophagus taurus] | 26 | 3 |
| Gene.6352 | PREDICTED: 40S ribosomal protein S23 [Aethina tumida] | 25 | 3 |
| Gene.6352 | PREDICTED: 40S ribosomal protein S23 [Aethina tumida] | 28 | 3 |
| Gene.6352 | PREDICTED: 40S ribosomal protein S23 [Aethina tumida] | 37 | 3 |
| Gene.6352 | PREDICTED: 40S ribosomal protein S23 [Aethina tumida] | 54 | 3 |
| Gene.6352 | PREDICTED: 40S ribosomal protein S23 [Aethina tumida] | 48 | 3 |
| Gene.6354 | Uncharacterized protein | 24 | 3 |
| Gene.6354 | Uncharacterized protein | 15 | 3 |
| Gene.6361 | translationally-controlled tumor protein homolog [Leptinotarsa decemlineata] | 118 | 3 |
| Gene.6361 | translationally-controlled tumor protein homolog [Leptinotarsa decemlineata] | 83 | 3 |
| Gene.6361 | translationally-controlled tumor protein homolog [Leptinotarsa decemlineata] | 91 | 3 |
| Gene.6361 | translationally-controlled tumor protein homolog [Leptinotarsa decemlineata] | 100 | 3 |
| Gene.6361 | translationally-controlled tumor protein homolog [Leptinotarsa decemlineata] | 121 | 3 |
| Gene.6361 | translationally-controlled tumor protein homolog [Leptinotarsa decemlineata] | 113 | 3 |
| Gene.6373 | PREDICTED: mitochondrial pyruvate carrier 2-like [Aethina tumida] | 17 | 3 |
| Gene.6375 | proliferation-associated protein 2G4 [Anoplophora glabripennis] | 192 | 3 |
| Gene.6375 | proliferation-associated protein 2G4 [Anoplophora glabripennis] | 287 | 3 |
| Gene.6375 | proliferation-associated protein 2G4 [Anoplophora glabripennis] | 20 | 3 |
| Gene.6375 | proliferation-associated protein 2G4 [Anoplophora glabripennis] | 176 | 3 |
| Gene.6380 | PREDICTED: 3'(2'),5'-bisphosphate nucleotidase 1 [Nicrophorus vespilloides] | 43 | 3 |
| Gene.6388 | PREDICTED: signal peptidase complex catalytic subunit SEC11A [Tribolium castaneum] | 115 | 3 |
| Gene.6402 | Uncharacterized protein | 48 | 3 |
| Gene.6419 | glutaredoxin-3 [Agrilus planipennis] | 51 | 3 |
| Gene.6419 | glutaredoxin-3 [Agrilus planipennis] | 105 | 3 |
| Gene.6419 | glutaredoxin-3 [Agrilus planipennis] | 100 | 3 |
| Gene.6425 | PREDICTED: clathrin light chain isoform X2 [Tribolium castaneum] | 116 | 3 |
| Gene.6439 | PREDICTED: protein DEK isoform X3 [Tribolium castaneum] | 206 | 3 |
| Gene.6439 | PREDICTED: protein DEK isoform X3 [Tribolium castaneum] | 259 | 3 |
| Gene.6439 | PREDICTED: protein DEK isoform X3 [Tribolium castaneum] | 262 | 3 |
| Gene.6439 | PREDICTED: protein DEK isoform X3 [Tribolium castaneum] | 220 | 3 |
| Gene.6439 | PREDICTED: protein DEK isoform X3 [Tribolium castaneum] | 193 | 3 |
| Gene.6439 | PREDICTED: protein DEK isoform X3 [Tribolium castaneum] | 505 | 3 |
| Gene.6439 | PREDICTED: protein DEK isoform X3 [Tribolium castaneum] | 278 | 3 |
| Gene.644 | remodeling and spacing factor 1-like, partial [Asbolus verrucosus] | 173 | 3 |
| Gene.6448 | glycine cleavage system H protein, mitochondrial [Leptinotarsa decemlineata] | 158 | 3 |
| Gene.6448 | glycine cleavage system H protein, mitochondrial [Leptinotarsa decemlineata] | 154 | 3 |
| Gene.6448 | glycine cleavage system H protein, mitochondrial [Leptinotarsa decemlineata] | 45 | 3 |
| Gene.6448 | glycine cleavage system H protein, mitochondrial [Leptinotarsa decemlineata] | 146 | 3 |
| Gene.6452 | chitinase 16 [Tribolium castaneum] | 170 | 3 |
| Gene.6452 | chitinase 16 [Tribolium castaneum] | 345 | 3 |
| Gene.6452 | chitinase 16 [Tribolium castaneum] | 121 | 3 |
| Gene.6469 | putative rRNA 2'-O-methyltransferase fibrillarin, partial [Polypedilum vanderplanki] | 304 | 3 |
| Gene.6469 | putative rRNA 2'-O-methyltransferase fibrillarin, partial [Polypedilum vanderplanki] | 219 | 3 |
| Gene.6477 | ribosomal protein S25 [Chrysomela tremula] | 42 | 3 |
| Gene.6477 | ribosomal protein S25 [Chrysomela tremula] | 59 | 3 |
| Gene.6477 | ribosomal protein S25 [Chrysomela tremula] | 93 | 3 |
| Gene.6477 | ribosomal protein S25 [Chrysomela tremula] | 56 | 3 |
| Gene.6477 | ribosomal protein S25 [Chrysomela tremula] | 51 | 3 |
| Gene.6477 | ribosomal protein S25 [Chrysomela tremula] | 65 | 3 |
| Gene.6492 | PREDICTED: nucleolysin TIAR [Dendroctonus ponderosae] | 74 | 3 |
| Gene.6493 | probable transaldolase [Leptinotarsa decemlineata] | 123 | 3 |
| Gene.6493 | probable transaldolase [Leptinotarsa decemlineata] | 309 | 3 |
| Gene.6493 | probable transaldolase [Leptinotarsa decemlineata] | 156 | 3 |
| Gene.6493 | probable transaldolase [Leptinotarsa decemlineata] | 138 | 3 |
| Gene.6493 | probable transaldolase [Leptinotarsa decemlineata] | 323 | 3 |
| Gene.6493 | probable transaldolase [Leptinotarsa decemlineata] | 227 | 3 |
| Gene.6493 | probable transaldolase [Leptinotarsa decemlineata] | 209 | 3 |
| Gene.6493 | probable transaldolase [Leptinotarsa decemlineata] | 66 | 3 |
| Gene.6493 | probable transaldolase [Leptinotarsa decemlineata] | 10 | 3 |
| Gene.6493 | probable transaldolase [Leptinotarsa decemlineata] | 117 | 3 |
| Gene.6525 | regulator of microtubule dynamics protein 2 isoform X1 [Anoplophora glabripennis] | 231 | 3 |
| Gene.6545 | PREDICTED: prostaglandin E synthase 2 [Tribolium castaneum] | 309 | 3 |
| Gene.6546 | eukaryotic translation initiation factor 4A [Epicauta chinensis] | 254 | 3 |
| Gene.6546 | eukaryotic translation initiation factor 4A [Epicauta chinensis] | 72 | 3 |
| Gene.6546 | eukaryotic translation initiation factor 4A [Epicauta chinensis] | 210 | 3 |
| Gene.6546 | eukaryotic translation initiation factor 4A [Epicauta chinensis] | 20 | 3 |
| Gene.6558 | PREDICTED: polyadenylate-binding protein 2 isoform X2 [Tribolium castaneum] | 63 | 3 |
| Gene.6558 | PREDICTED: polyadenylate-binding protein 2 isoform X2 [Tribolium castaneum] | 133 | 3 |
| Gene.6559 | PREDICTED: transcriptional activator protein Pur-alpha isoform X1 [Tribolium castaneum] | 114 | 3 |
| Gene.6561 | protein phosphatase 2, regulatory subunit A, alpha isoform [Tribolium castaneum] | 544 | 3 |
| Gene.6561 | protein phosphatase 2, regulatory subunit A, alpha isoform [Tribolium castaneum] | 268 | 3 |
| Gene.6564 | peroxiredoxin-5, mitochondrial [Leptinotarsa decemlineata] | 120 | 3 |
| Gene.6564 | peroxiredoxin-5, mitochondrial [Leptinotarsa decemlineata] | 57 | 3 |
| Gene.6564 | peroxiredoxin-5, mitochondrial [Leptinotarsa decemlineata] | 133 | 3 |
| Gene.6572 | macrophage migration inhibitory factor homolog [Agrilus planipennis] | 86 | 3 |
| Gene.6572 | macrophage migration inhibitory factor homolog [Agrilus planipennis] | 78 | 3 |
| Gene.6573 | PREDICTED: 40S ribosomal protein S15 [Tribolium castaneum] | 8 | 3 |
| Gene.6573 | PREDICTED: 40S ribosomal protein S15 [Tribolium castaneum] | 73 | 3 |
| Gene.6597 | heat shock protein 21.53 [Harmonia axyridis] | 119 | 3 |
| Gene.6597 | heat shock protein 21.53 [Harmonia axyridis] | 179 | 3 |
| Gene.6597 | heat shock protein 21.53 [Harmonia axyridis] | 157 | 3 |
| Gene.6597 | heat shock protein 21.53 [Harmonia axyridis] | 43 | 3 |
| Gene.6597 | heat shock protein 21.53 [Harmonia axyridis] | 171 | 3 |
| Gene.6597 | heat shock protein 21.53 [Harmonia axyridis] | 143 | 3 |
| Gene.6598 | hypothetical protein BDFB_006472 [Asbolus verrucosus] | 215 | 3 |
| Gene.6601 | putative ribosomal protein L6 [Phaedon cochleariae] | 90 | 3 |
| Gene.6601 | putative ribosomal protein L6 [Phaedon cochleariae] | 213 | 3 |
| Gene.6601 | putative ribosomal protein L6 [Phaedon cochleariae] | 184 | 3 |
| Gene.6601 | putative ribosomal protein L6 [Phaedon cochleariae] | 100 | 3 |
| Gene.6601 | putative ribosomal protein L6 [Phaedon cochleariae] | 195 | 3 |
| Gene.6605 | hypothetical protein AMK59_7061, partial [Oryctes borbonicus] | 125 | 3 |
| Gene.6605 | hypothetical protein AMK59_7061, partial [Oryctes borbonicus] | 76 | 3 |
| Gene.6624 | 40S ribosomal protein S5 [Asbolus verrucosus] | 212 | 3 |
| Gene.664 | PREDICTED: V-type proton ATPase 116 kDa subunit a isoform 1 isoform X3 [Tribolium castaneum] | 50 | 3 |
| Gene.6649 | hypothetical protein AMK59_4881 [Oryctes borbonicus] | 247 | 3 |
| Gene.665 | PREDICTED: 1,4-alpha-glucan-branching enzyme [Aethina tumida] | 572 | 3 |
| Gene.665 | PREDICTED: 1,4-alpha-glucan-branching enzyme [Aethina tumida] | 71 | 3 |
| Gene.665 | PREDICTED: 1,4-alpha-glucan-branching enzyme [Aethina tumida] | 631 | 3 |
| Gene.665 | PREDICTED: 1,4-alpha-glucan-branching enzyme [Aethina tumida] | 143 | 3 |
| Gene.665 | PREDICTED: 1,4-alpha-glucan-branching enzyme [Aethina tumida] | 606 | 3 |
| Gene.665 | PREDICTED: 1,4-alpha-glucan-branching enzyme [Aethina tumida] | 166 | 3 |
| Gene.665 | PREDICTED: 1,4-alpha-glucan-branching enzyme [Aethina tumida] | 570 | 3 |
| Gene.665 | PREDICTED: 1,4-alpha-glucan-branching enzyme [Aethina tumida] | 105 | 3 |
| Gene.6654 | jupiter microtubule associated homolog 1-like [Leptinotarsa decemlineata] | 35 | 3 |
| Gene.6667 | PREDICTED: gem-associated protein 2 isoform X2 [Tribolium castaneum] | 129 | 3 |
| Gene.6668 | PREDICTED: antichymotrypsin-2 isoform X7 [Tribolium castaneum] | 285 | 3 |
| Gene.6672 | PREDICTED: 60S ribosomal protein L13a [Aethina tumida] | 189 | 3 |
| Gene.6672 | PREDICTED: 60S ribosomal protein L13a [Aethina tumida] | 108 | 3 |
| Gene.6672 | PREDICTED: 60S ribosomal protein L13a [Aethina tumida] | 51 | 3 |
| Gene.6672 | PREDICTED: 60S ribosomal protein L13a [Aethina tumida] | 17 | 3 |
| Gene.6672 | PREDICTED: 60S ribosomal protein L13a [Aethina tumida] | 58 | 3 |
| Gene.6672 | PREDICTED: 60S ribosomal protein L13a [Aethina tumida] | 153 | 3 |
| Gene.6672 | PREDICTED: 60S ribosomal protein L13a [Aethina tumida] | 87 | 3 |
| Gene.6672 | PREDICTED: 60S ribosomal protein L13a [Aethina tumida] | 30 | 3 |
| Gene.6674 | PREDICTED: malate dehydrogenase, mitochondrial [Tribolium castaneum] | 238 | 3 |
| Gene.6674 | PREDICTED: malate dehydrogenase, mitochondrial [Tribolium castaneum] | 90 | 3 |
| Gene.6674 | PREDICTED: malate dehydrogenase, mitochondrial [Tribolium castaneum] | 323 | 3 |
| Gene.6674 | PREDICTED: malate dehydrogenase, mitochondrial [Tribolium castaneum] | 184 | 3 |
| Gene.6674 | PREDICTED: malate dehydrogenase, mitochondrial [Tribolium castaneum] | 163 | 3 |
| Gene.6674 | PREDICTED: malate dehydrogenase, mitochondrial [Tribolium castaneum] | 77 | 3 |
| Gene.6674 | PREDICTED: malate dehydrogenase, mitochondrial [Tribolium castaneum] | 125 | 3 |
| Gene.6674 | PREDICTED: malate dehydrogenase, mitochondrial [Tribolium castaneum] | 335 | 3 |
| Gene.6674 | PREDICTED: malate dehydrogenase, mitochondrial [Tribolium castaneum] | 86 | 3 |
| Gene.6679 | Isocitrate dehydrogenase [NADP] [Operophtera brumata] | 345 | 3 |
| Gene.6679 | Isocitrate dehydrogenase [NADP] [Operophtera brumata] | 240 | 3 |
| Gene.6679 | Isocitrate dehydrogenase [NADP] [Operophtera brumata] | 26 | 3 |
| Gene.6692 | D-3-phosphoglycerate dehydrogenase, partial [Asbolus verrucosus] | 59 | 3 |
| Gene.6692 | D-3-phosphoglycerate dehydrogenase, partial [Asbolus verrucosus] | 182 | 3 |
| Gene.6692 | D-3-phosphoglycerate dehydrogenase, partial [Asbolus verrucosus] | 43 | 3 |
| Gene.6692 | D-3-phosphoglycerate dehydrogenase, partial [Asbolus verrucosus] | 55 | 3 |
| Gene.6704 | dehydrogenase/reductase SDR family member 11 [Asbolus verrucosus] | 60 | 3 |
| Gene.6713 | PREDICTED: protein ERGIC-53 [Tribolium castaneum] | 414 | 3 |
| Gene.6754 | apoptosis-inducing factor 1, mitochondrial [Leptinotarsa decemlineata] | 217 | 3 |
| Gene.6754 | apoptosis-inducing factor 1, mitochondrial [Leptinotarsa decemlineata] | 543 | 3 |
| Gene.6759 | hypothetical protein AMK59_2413 [Oryctes borbonicus] | 54 | 3 |
| Gene.6774 | PREDICTED: basigin isoform X1 [Nicrophorus vespilloides] | 281 | 3 |
| Gene.6774 | PREDICTED: basigin isoform X1 [Nicrophorus vespilloides] | 181 | 3 |
| Gene.6774 | PREDICTED: basigin isoform X1 [Nicrophorus vespilloides] | 64 | 3 |
| Gene.6782 | LIM and SH3 domain protein Lasp-like Protein [Tribolium castaneum] | 96 | 3 |
| Gene.6791 | thymosin beta isoform X2 [Leptinotarsa decemlineata] | 22 | 3 |
| Gene.6791 | thymosin beta isoform X2 [Leptinotarsa decemlineata] | 161 | 3 |
| Gene.6791 | thymosin beta isoform X2 [Leptinotarsa decemlineata] | 34 | 3 |
| Gene.6791 | thymosin beta isoform X2 [Leptinotarsa decemlineata] | 29 | 3 |
| Gene.6798 | putative ATP-dependent RNA helicase me31b [Anoplophora glabripennis] | 204 | 3 |
| Gene.6798 | putative ATP-dependent RNA helicase me31b [Anoplophora glabripennis] | 436 | 3 |
| Gene.6798 | putative ATP-dependent RNA helicase me31b [Anoplophora glabripennis] | 68 | 3 |
| Gene.6798 | putative ATP-dependent RNA helicase me31b [Anoplophora glabripennis] | 425 | 3 |
| Gene.6798 | putative ATP-dependent RNA helicase me31b [Anoplophora glabripennis] | 430 | 3 |
| Gene.6798 | putative ATP-dependent RNA helicase me31b [Anoplophora glabripennis] | 315 | 3 |
| Gene.6798 | putative ATP-dependent RNA helicase me31b [Anoplophora glabripennis] | 421 | 3 |
| Gene.6798 | putative ATP-dependent RNA helicase me31b [Anoplophora glabripennis] | 98 | 3 |
| Gene.6798 | putative ATP-dependent RNA helicase me31b [Anoplophora glabripennis] | 37 | 3 |
| Gene.6798 | putative ATP-dependent RNA helicase me31b [Anoplophora glabripennis] | 195 | 3 |
| Gene.68 | PREDICTED: prolyl endopeptidase isoform X1 [Tribolium castaneum] | 468 | 3 |
| Gene.68 | PREDICTED: prolyl endopeptidase isoform X1 [Tribolium castaneum] | 376 | 3 |
| Gene.6801 | hypothetical protein TcasGA2_TC031075 [Tribolium castaneum] | 229 | 3 |
| Gene.6801 | hypothetical protein TcasGA2_TC031075 [Tribolium castaneum] | 230 | 3 |
| Gene.6803 | PREDICTED: paramyosin, long form-like [Plutella xylostella] | 76 | 3 |
| Gene.6803 | PREDICTED: paramyosin, long form-like [Plutella xylostella] | 96 | 3 |
| Gene.6805 | peptidyl-prolyl cis-trans isomerase [Leptinotarsa decemlineata] | 205 | 3 |
| Gene.6805 | peptidyl-prolyl cis-trans isomerase [Leptinotarsa decemlineata] | 130 | 3 |
| Gene.6805 | peptidyl-prolyl cis-trans isomerase [Leptinotarsa decemlineata] | 85 | 3 |
| Gene.6805 | peptidyl-prolyl cis-trans isomerase [Leptinotarsa decemlineata] | 194 | 3 |
| Gene.6806 | PREDICTED: NADH-cytochrome b5 reductase 2 isoform X2 [Tribolium castaneum] | 130 | 3 |
| Gene.6806 | PREDICTED: NADH-cytochrome b5 reductase 2 isoform X2 [Tribolium castaneum] | 175 | 3 |
| Gene.6806 | PREDICTED: NADH-cytochrome b5 reductase 2 isoform X2 [Tribolium castaneum] | 65 | 3 |
| Gene.6809 | 60S ribosomal protein L12 [Lucilia cuprina] | 40 | 3 |
| Gene.6809 | 60S ribosomal protein L12 [Lucilia cuprina] | 114 | 3 |
| Gene.6809 | 60S ribosomal protein L12 [Lucilia cuprina] | 86 | 3 |
| Gene.6809 | 60S ribosomal protein L12 [Lucilia cuprina] | 54 | 3 |
| Gene.681 | methylglutaconyl-CoA hydratase, mitochondrial [Asbolus verrucosus] | 85 | 3 |
| Gene.6811 | PREDICTED: uncharacterized protein LOC109595590 [Aethina tumida] | 383 | 3 |
| Gene.6827 | myosin light chain alkali, partial [Asbolus verrucosus] | 61 | 3 |
| Gene.6827 | myosin light chain alkali, partial [Asbolus verrucosus] | 16 | 3 |
| Gene.6845 | PREDICTED: 39S ribosomal protein L28, mitochondrial [Tribolium castaneum] | 31 | 3 |
| Gene.6879 | V-type proton ATPase subunit E [Leptinotarsa decemlineata] | 59 | 3 |
| Gene.6879 | V-type proton ATPase subunit E [Leptinotarsa decemlineata] | 42 | 3 |
| Gene.6879 | V-type proton ATPase subunit E [Leptinotarsa decemlineata] | 156 | 3 |
| Gene.6879 | V-type proton ATPase subunit E [Leptinotarsa decemlineata] | 68 | 3 |
| Gene.6879 | V-type proton ATPase subunit E [Leptinotarsa decemlineata] | 10 | 3 |
| Gene.6885 | PREDICTED: RNA-binding protein cabeza [Tribolium castaneum] | 149 | 3 |
| Gene.6894 | PREDICTED: adenylate kinase [Tribolium castaneum] | 67 | 3 |
| Gene.6894 | PREDICTED: adenylate kinase [Tribolium castaneum] | 232 | 3 |
| Gene.6894 | PREDICTED: adenylate kinase [Tribolium castaneum] | 188 | 3 |
| Gene.6894 | PREDICTED: adenylate kinase [Tribolium castaneum] | 121 | 3 |
| Gene.6924 | PREDICTED: 40S ribosomal protein SA [Tribolium castaneum] | 11 | 3 |
| Gene.6924 | PREDICTED: 40S ribosomal protein SA [Tribolium castaneum] | 212 | 3 |
| Gene.6924 | PREDICTED: 40S ribosomal protein SA [Tribolium castaneum] | 57 | 3 |
| Gene.6924 | PREDICTED: 40S ribosomal protein SA [Tribolium castaneum] | 89 | 3 |
| Gene.6924 | PREDICTED: 40S ribosomal protein SA [Tribolium castaneum] | 16 | 3 |
| Gene.6928 | PREDICTED: transitional endoplasmic reticulum ATPase TER94 [Tribolium castaneum] | 22 | 3 |
| Gene.6928 | PREDICTED: transitional endoplasmic reticulum ATPase TER94 [Tribolium castaneum] | 672 | 3 |
| Gene.6928 | PREDICTED: transitional endoplasmic reticulum ATPase TER94 [Tribolium castaneum] | 662 | 3 |
| Gene.6928 | PREDICTED: transitional endoplasmic reticulum ATPase TER94 [Tribolium castaneum] | 509 | 3 |
| Gene.6928 | PREDICTED: transitional endoplasmic reticulum ATPase TER94 [Tribolium castaneum] | 569 | 3 |
| Gene.6958 | PREDICTED: S-methyl-5'-thioadenosine phosphorylase isoform X2 [Nicrophorus vespilloides] | 236 | 3 |
| Gene.696 | ATP-dependent RNA helicase p62, partial [Asbolus verrucosus] | 298 | 3 |
| Gene.6962 | PREDICTED: vesicle-associated membrane protein-associated protein B [Tribolium castaneum] | 17 | 3 |
| Gene.6967 | 60S ribosomal protein L27a [Trachymyrmex cornetzi] | 18 | 3 |
| Gene.6967 | 60S ribosomal protein L27a [Trachymyrmex cornetzi] | 73 | 3 |
| Gene.6967 | 60S ribosomal protein L27a [Trachymyrmex cornetzi] | 10 | 3 |
| Gene.6967 | 60S ribosomal protein L27a [Trachymyrmex cornetzi] | 68 | 3 |
| Gene.6967 | 60S ribosomal protein L27a [Trachymyrmex cornetzi] | 31 | 3 |
| Gene.6967 | 60S ribosomal protein L27a [Trachymyrmex cornetzi] | 54 | 3 |
| Gene.6969 | kynurenine--oxoglutarate transaminase 3 [Anoplophora glabripennis] | 8 | 3 |
| Gene.6969 | kynurenine--oxoglutarate transaminase 3 [Anoplophora glabripennis] | 154 | 3 |
| Gene.6977 | AFG3-like protein 2 [Anoplophora glabripennis] | 627 | 3 |
| Gene.6977 | AFG3-like protein 2 [Anoplophora glabripennis] | 707 | 3 |
| Gene.6977 | AFG3-like protein 2 [Anoplophora glabripennis] | 489 | 3 |
| Gene.6977 | AFG3-like protein 2 [Anoplophora glabripennis] | 297 | 3 |
| Gene.6981 | PREDICTED: receptor expression-enhancing protein 5-like [Aethina tumida] | 182 | 3 |
| Gene.6988 | REPAT29 [Spodoptera exigua] | 41 | 3 |
| Gene.6989 | 26S proteasome non-ATPase regulatory subunit 7 [Onthophagus taurus] | 212 | 3 |
| Gene.6997 | PREDICTED: glyoxylate reductase/hydroxypyruvate reductase [Dendroctonus ponderosae] | 91 | 3 |
| Gene.7002 | ribosomal protein L23 [Chrysomela tremula] | 123 | 3 |
| Gene.7002 | ribosomal protein L23 [Chrysomela tremula] | 13 | 3 |
| Gene.7002 | ribosomal protein L23 [Chrysomela tremula] | 113 | 3 |
| Gene.7003 | Chromo domain containing protein, partial [Asbolus verrucosus] | 319 | 3 |
| Gene.7003 | Chromo domain containing protein, partial [Asbolus verrucosus] | 270 | 3 |
| Gene.7019 | PREDICTED: ultraviolet-B receptor UVR8 [Tribolium castaneum] | 147 | 3 |
| Gene.7021 | PREDICTED: prefoldin subunit 6 [Tribolium castaneum] | 58 | 3 |
| Gene.7035 | NHP2-like protein 1 [Agrilus planipennis] | 43 | 3 |
| Gene.7038 | 60S ribosomal protein L18a [Leptinotarsa decemlineata] | 25 | 3 |
| Gene.7038 | 60S ribosomal protein L18a [Leptinotarsa decemlineata] | 58 | 3 |
| Gene.7038 | 60S ribosomal protein L18a [Leptinotarsa decemlineata] | 87 | 3 |
| Gene.7038 | 60S ribosomal protein L18a [Leptinotarsa decemlineata] | 166 | 3 |
| Gene.7038 | 60S ribosomal protein L18a [Leptinotarsa decemlineata] | 93 | 3 |
| Gene.7038 | 60S ribosomal protein L18a [Leptinotarsa decemlineata] | 46 | 3 |
| Gene.7038 | 60S ribosomal protein L18a [Leptinotarsa decemlineata] | 82 | 3 |
| Gene.704 | polyadenylate-binding protein 4-like [Anoplophora glabripennis] | 104 | 3 |
| Gene.704 | polyadenylate-binding protein 4-like [Anoplophora glabripennis] | 286 | 3 |
| Gene.704 | polyadenylate-binding protein 4-like [Anoplophora glabripennis] | 226 | 3 |
| Gene.704 | polyadenylate-binding protein 4-like [Anoplophora glabripennis] | 78 | 3 |
| Gene.7060 | MICOS complex subunit MIC13 homolog QIL1 isoform X1 [Anoplophora glabripennis] | 118 | 3 |
| Gene.7082 | PREDICTED: atlastin isoform X1 [Tribolium castaneum] | 427 | 3 |
| Gene.7085 | PREDICTED: serine/arginine-rich splicing factor 1A [Tribolium castaneum] | 22 | 3 |
| Gene.7085 | PREDICTED: serine/arginine-rich splicing factor 1A [Tribolium castaneum] | 33 | 3 |
| Gene.709 | ribosomal protein S15, partial [Harmonia axyridis] | 60 | 3 |
| Gene.709 | ribosomal protein S15, partial [Harmonia axyridis] | 19 | 3 |
| Gene.7091 | Proteasome domain containing protein [Asbolus verrucosus] | 217 | 3 |
| Gene.7108 | unknown [Dendroctonus ponderosae] | 54 | 3 |
| Gene.7112 | uroporphyrinogen decarboxylase [Anoplophora glabripennis] | 116 | 3 |
| Gene.7117 | T-complex protein 1 subunit alpha [Anoplophora glabripennis] | 403 | 3 |
| Gene.7117 | T-complex protein 1 subunit alpha [Anoplophora glabripennis] | 535 | 3 |
| Gene.7117 | T-complex protein 1 subunit alpha [Anoplophora glabripennis] | 188 | 3 |
| Gene.7117 | T-complex protein 1 subunit alpha [Anoplophora glabripennis] | 275 | 3 |
| Gene.7129 | PREDICTED: fumarylacetoacetate hydrolase domain-containing protein 2 [Aethina tumida] | 230 | 3 |
| Gene.7129 | PREDICTED: fumarylacetoacetate hydrolase domain-containing protein 2 [Aethina tumida] | 121 | 3 |
| Gene.713 | PREDICTED: probable dolichol-phosphate mannosyltransferase [Tribolium castaneum] | 183 | 3 |
| Gene.7131 | ras-related protein Rab-32 isoform X1 [Anoplophora glabripennis] | 267 | 3 |
| Gene.7132 | 60S ribosomal protein L23a-like, partial [Asbolus verrucosus] | 203 | 3 |
| Gene.7134 | PREDICTED: ATP synthase subunit d, mitochondrial [Tribolium castaneum] | 95 | 3 |
| Gene.7134 | PREDICTED: ATP synthase subunit d, mitochondrial [Tribolium castaneum] | 105 | 3 |
| Gene.7147 | PREDICTED: N-alpha-acetyltransferase 16, NatA auxiliary subunit isoform X2 [Tribolium castaneum] | 371 | 3 |
| Gene.7147 | PREDICTED: N-alpha-acetyltransferase 16, NatA auxiliary subunit isoform X2 [Tribolium castaneum] | 363 | 3 |
| Gene.7166 | PREDICTED: rab GDP dissociation inhibitor alpha [Aethina tumida] | 433 | 3 |
| Gene.7166 | PREDICTED: rab GDP dissociation inhibitor alpha [Aethina tumida] | 111 | 3 |
| Gene.7166 | PREDICTED: rab GDP dissociation inhibitor alpha [Aethina tumida] | 117 | 3 |
| Gene.7213 | PREDICTED: protein 4.1 homolog isoform X2 [Dendroctonus ponderosae] | 524 | 3 |
| Gene.7216 | PREDICTED: microtubule-associated protein RP/EB family member 1 isoform X2 [Tribolium castaneum] | 83 | 3 |
| Gene.7216 | PREDICTED: microtubule-associated protein RP/EB family member 1 isoform X2 [Tribolium castaneum] | 89 | 3 |
| Gene.7218 | MICOS complex subunit Mic60 isoform X1 [Leptinotarsa decemlineata] | 387 | 3 |
| Gene.7218 | MICOS complex subunit Mic60 isoform X1 [Leptinotarsa decemlineata] | 151 | 3 |
| Gene.7218 | MICOS complex subunit Mic60 isoform X1 [Leptinotarsa decemlineata] | 275 | 3 |
| Gene.7218 | MICOS complex subunit Mic60 isoform X1 [Leptinotarsa decemlineata] | 480 | 3 |
| Gene.7218 | MICOS complex subunit Mic60 isoform X1 [Leptinotarsa decemlineata] | 220 | 3 |
| Gene.7221 | PREDICTED: ras-related protein Rab-1A [Tribolium castaneum] | 128 | 3 |
| Gene.7221 | PREDICTED: ras-related protein Rab-1A [Tribolium castaneum] | 187 | 3 |
| Gene.7236 | Uncharacterized protein | 96 | 3 |
| Gene.7236 | Uncharacterized protein | 29 | 3 |
| Gene.7237 | Uncharacterized protein | 72 | 3 |
| Gene.7237 | Uncharacterized protein | 26 | 3 |
| Gene.7241 | uridine diphosphate glucose pyrophosphatase [Anoplophora glabripennis] | 19 | 3 |
| Gene.725 | PREDICTED: myosin regulatory light chain 2 [Aethina tumida] | 66 | 3 |
| Gene.725 | PREDICTED: myosin regulatory light chain 2 [Aethina tumida] | 62 | 3 |
| Gene.7264 | heat shock protein 90, partial [Harmonia axyridis] | 559 | 3 |
| Gene.7264 | heat shock protein 90, partial [Harmonia axyridis] | 643 | 3 |
| Gene.7264 | heat shock protein 90, partial [Harmonia axyridis] | 67 | 3 |
| Gene.7264 | heat shock protein 90, partial [Harmonia axyridis] | 571 | 3 |
| Gene.7264 | heat shock protein 90, partial [Harmonia axyridis] | 217 | 3 |
| Gene.7264 | heat shock protein 90, partial [Harmonia axyridis] | 341 | 3 |
| Gene.7264 | heat shock protein 90, partial [Harmonia axyridis] | 475 | 3 |
| Gene.7264 | heat shock protein 90, partial [Harmonia axyridis] | 464 | 3 |
| Gene.7264 | heat shock protein 90, partial [Harmonia axyridis] | 269 | 3 |
| Gene.7264 | heat shock protein 90, partial [Harmonia axyridis] | 212 | 3 |
| Gene.7264 | heat shock protein 90, partial [Harmonia axyridis] | 72 | 3 |
| Gene.7264 | heat shock protein 90, partial [Harmonia axyridis] | 444 | 3 |
| Gene.7264 | heat shock protein 90, partial [Harmonia axyridis] | 532 | 3 |
| Gene.7264 | heat shock protein 90, partial [Harmonia axyridis] | 525 | 3 |
| Gene.7264 | heat shock protein 90, partial [Harmonia axyridis] | 105 | 3 |
| Gene.7264 | heat shock protein 90, partial [Harmonia axyridis] | 553 | 3 |
| Gene.7264 | heat shock protein 90, partial [Harmonia axyridis] | 562 | 3 |
| Gene.7264 | heat shock protein 90, partial [Harmonia axyridis] | 280 | 3 |
| Gene.7264 | heat shock protein 90, partial [Harmonia axyridis] | 259 | 3 |
| Gene.7264 | heat shock protein 90, partial [Harmonia axyridis] | 51 | 3 |
| Gene.7264 | heat shock protein 90, partial [Harmonia axyridis] | 429 | 3 |
| Gene.7264 | heat shock protein 90, partial [Harmonia axyridis] | 184 | 3 |
| Gene.7264 | heat shock protein 90, partial [Harmonia axyridis] | 601 | 3 |
| Gene.7264 | heat shock protein 90, partial [Harmonia axyridis] | 568 | 3 |
| Gene.7264 | heat shock protein 90, partial [Harmonia axyridis] | 544 | 3 |
| Gene.7264 | heat shock protein 90, partial [Harmonia axyridis] | 278 | 3 |
| Gene.7264 | heat shock protein 90, partial [Harmonia axyridis] | 393 | 3 |
| Gene.7264 | heat shock protein 90, partial [Harmonia axyridis] | 432 | 3 |
| Gene.7264 | heat shock protein 90, partial [Harmonia axyridis] | 405 | 3 |
| Gene.7264 | heat shock protein 90, partial [Harmonia axyridis] | 195 | 3 |
| Gene.7270 | 40S ribosomal protein S11 isoform X1 [Leptinotarsa decemlineata] | 161 | 3 |
| Gene.7270 | 40S ribosomal protein S11 isoform X1 [Leptinotarsa decemlineata] | 75 | 3 |
| Gene.7270 | 40S ribosomal protein S11 isoform X1 [Leptinotarsa decemlineata] | 62 | 3 |
| Gene.7270 | 40S ribosomal protein S11 isoform X1 [Leptinotarsa decemlineata] | 153 | 3 |
| Gene.7276 | protein disulfide-isomerase A3 [Asbolus verrucosus] | 170 | 3 |
| Gene.7276 | protein disulfide-isomerase A3 [Asbolus verrucosus] | 210 | 3 |
| Gene.7276 | protein disulfide-isomerase A3 [Asbolus verrucosus] | 165 | 3 |
| Gene.7276 | protein disulfide-isomerase A3 [Asbolus verrucosus] | 134 | 3 |
| Gene.7276 | protein disulfide-isomerase A3 [Asbolus verrucosus] | 268 | 3 |
| Gene.7276 | protein disulfide-isomerase A3 [Asbolus verrucosus] | 78 | 3 |
| Gene.7276 | protein disulfide-isomerase A3 [Asbolus verrucosus] | 449 | 3 |
| Gene.7276 | protein disulfide-isomerase A3 [Asbolus verrucosus] | 62 | 3 |
| Gene.7276 | protein disulfide-isomerase A3 [Asbolus verrucosus] | 247 | 3 |
| Gene.7283 | Uncharacterized protein | 87 | 3 |
| Gene.7283 | Uncharacterized protein | 96 | 3 |
| Gene.7283 | Uncharacterized protein | 107 | 3 |
| Gene.730 | long-chain-fatty-acid--CoA ligase ACSBG2 [Anoplophora glabripennis] | 308 | 3 |
| Gene.730 | long-chain-fatty-acid--CoA ligase ACSBG2 [Anoplophora glabripennis] | 161 | 3 |
| Gene.730 | long-chain-fatty-acid--CoA ligase ACSBG2 [Anoplophora glabripennis] | 318 | 3 |
| Gene.7312 | alpha-soluble NSF attachment protein [Anoplophora glabripennis] | 201 | 3 |
| Gene.7312 | alpha-soluble NSF attachment protein [Anoplophora glabripennis] | 91 | 3 |
| Gene.7326 | serine hydroxymethyltransferase, cytosolic isoform X1 [Anoplophora glabripennis] | 464 | 3 |
| Gene.7326 | serine hydroxymethyltransferase, cytosolic isoform X1 [Anoplophora glabripennis] | 475 | 3 |
| Gene.7356 | PREDICTED: peroxiredoxin-1 [Tribolium castaneum] | 69 | 3 |
| Gene.7356 | PREDICTED: peroxiredoxin-1 [Tribolium castaneum] | 96 | 3 |
| Gene.7360 | PREDICTED: uncharacterized protein LOC663797 [Tribolium castaneum] | 244 | 3 |
| Gene.7360 | PREDICTED: uncharacterized protein LOC663797 [Tribolium castaneum] | 250 | 3 |
| Gene.7366 | PREDICTED: V-type proton ATPase subunit G [Nicrophorus vespilloides] | 21 | 3 |
| Gene.7366 | PREDICTED: V-type proton ATPase subunit G [Nicrophorus vespilloides] | 37 | 3 |
| Gene.7366 | PREDICTED: V-type proton ATPase subunit G [Nicrophorus vespilloides] | 61 | 3 |
| Gene.7371 | PBP GOBP domain containing protein [Asbolus verrucosus] | 101 | 3 |
| Gene.7371 | PBP GOBP domain containing protein [Asbolus verrucosus] | 94 | 3 |
| Gene.7371 | PBP GOBP domain containing protein [Asbolus verrucosus] | 91 | 3 |
| Gene.7376 | PREDICTED: LOW QUALITY PROTEIN: leucine-rich PPR motif-containing protein, mitochondrial [Aethina tumida] | 81 | 3 |
| Gene.7388 | short-chain specific acyl-CoA dehydrogenase, mitochondrial [Anoplophora glabripennis] | 298 | 3 |
| Gene.7388 | short-chain specific acyl-CoA dehydrogenase, mitochondrial [Anoplophora glabripennis] | 330 | 3 |
| Gene.7388 | short-chain specific acyl-CoA dehydrogenase, mitochondrial [Anoplophora glabripennis] | 51 | 3 |
| Gene.7388 | short-chain specific acyl-CoA dehydrogenase, mitochondrial [Anoplophora glabripennis] | 218 | 3 |
| Gene.7388 | short-chain specific acyl-CoA dehydrogenase, mitochondrial [Anoplophora glabripennis] | 327 | 3 |
| Gene.7388 | short-chain specific acyl-CoA dehydrogenase, mitochondrial [Anoplophora glabripennis] | 212 | 3 |
| Gene.7388 | short-chain specific acyl-CoA dehydrogenase, mitochondrial [Anoplophora glabripennis] | 294 | 3 |
| Gene.7388 | short-chain specific acyl-CoA dehydrogenase, mitochondrial [Anoplophora glabripennis] | 335 | 3 |
| Gene.7395 | titin [Asbolus verrucosus] | 169 | 3 |
| Gene.7399 | 3-hydroxyisobutyrate dehydrogenase, mitochondrial [Leptinotarsa decemlineata] | 59 | 3 |
| Gene.7399 | 3-hydroxyisobutyrate dehydrogenase, mitochondrial [Leptinotarsa decemlineata] | 130 | 3 |
| Gene.7399 | 3-hydroxyisobutyrate dehydrogenase, mitochondrial [Leptinotarsa decemlineata] | 49 | 3 |
| Gene.7399 | 3-hydroxyisobutyrate dehydrogenase, mitochondrial [Leptinotarsa decemlineata] | 105 | 3 |
| Gene.7399 | 3-hydroxyisobutyrate dehydrogenase, mitochondrial [Leptinotarsa decemlineata] | 301 | 3 |
| Gene.7400 | PREDICTED: nucleolar protein 58 [Aethina tumida] | 77 | 3 |
| Gene.7400 | PREDICTED: nucleolar protein 58 [Aethina tumida] | 153 | 3 |
| Gene.7409 | PREDICTED: T-complex protein 1 subunit delta [Tribolium castaneum] | 203 | 3 |
| Gene.7409 | PREDICTED: T-complex protein 1 subunit delta [Tribolium castaneum] | 234 | 3 |
| Gene.7409 | PREDICTED: T-complex protein 1 subunit delta [Tribolium castaneum] | 14 | 3 |
| Gene.7410 | 60S ribosomal protein L14, partial [Asbolus verrucosus] | 71 | 3 |
| Gene.7410 | 60S ribosomal protein L14, partial [Asbolus verrucosus] | 75 | 3 |
| Gene.7410 | 60S ribosomal protein L14, partial [Asbolus verrucosus] | 105 | 3 |
| Gene.7410 | 60S ribosomal protein L14, partial [Asbolus verrucosus] | 143 | 3 |
| Gene.742 | NADH dehydrogenase [ubiquinone] 1 alpha subcomplex subunit 9, mitochondrial [Anoplophora glabripennis] | 137 | 3 |
| Gene.742 | NADH dehydrogenase [ubiquinone] 1 alpha subcomplex subunit 9, mitochondrial [Anoplophora glabripennis] | 142 | 3 |
| Gene.742 | NADH dehydrogenase [ubiquinone] 1 alpha subcomplex subunit 9, mitochondrial [Anoplophora glabripennis] | 278 | 3 |
| Gene.742 | NADH dehydrogenase [ubiquinone] 1 alpha subcomplex subunit 9, mitochondrial [Anoplophora glabripennis] | 268 | 3 |
| Gene.7424 | carbonyl reductase [NADPH] 3 [Anoplophora glabripennis] | 4 | 3 |
| Gene.7424 | carbonyl reductase [NADPH] 3 [Anoplophora glabripennis] | 72 | 3 |
| Gene.7429 | eukaryotic peptide chain release factor subunit 1 [Anoplophora glabripennis] | 16 | 3 |
| Gene.7429 | eukaryotic peptide chain release factor subunit 1 [Anoplophora glabripennis] | 249 | 3 |
| Gene.744 | PREDICTED: putative RNA-binding protein 15B [Tribolium castaneum] | 615 | 3 |
| Gene.744 | PREDICTED: putative RNA-binding protein 15B [Tribolium castaneum] | 103 | 3 |
| Gene.7443 | PREDICTED: regulator of chromosome condensation [Tribolium castaneum] | 347 | 3 |
| Gene.7445 | PREDICTED: 26S protease regulatory subunit 6B [Nicrophorus vespilloides] | 392 | 3 |
| Gene.7445 | PREDICTED: 26S protease regulatory subunit 6B [Nicrophorus vespilloides] | 388 | 3 |
| Gene.7482 | failed axon connections isoform X1 [Anoplophora glabripennis] | 27 | 3 |
| Gene.7482 | failed axon connections isoform X1 [Anoplophora glabripennis] | 341 | 3 |
| Gene.7482 | failed axon connections isoform X1 [Anoplophora glabripennis] | 184 | 3 |
| Gene.7482 | failed axon connections isoform X1 [Anoplophora glabripennis] | 353 | 3 |
| Gene.7482 | failed axon connections isoform X1 [Anoplophora glabripennis] | 18 | 3 |
| Gene.7482 | failed axon connections isoform X1 [Anoplophora glabripennis] | 375 | 3 |
| Gene.7482 | failed axon connections isoform X1 [Anoplophora glabripennis] | 96 | 3 |
| Gene.7482 | failed axon connections isoform X1 [Anoplophora glabripennis] | 44 | 3 |
| Gene.7492 | uncharacterized protein LOC108912786 [Anoplophora glabripennis] | 271 | 3 |
| Gene.7492 | uncharacterized protein LOC108912786 [Anoplophora glabripennis] | 69 | 3 |
| Gene.7501 | nucleosome assembly protein 1-like 1-B [Anoplophora glabripennis] | 244 | 3 |
| Gene.7501 | nucleosome assembly protein 1-like 1-B [Anoplophora glabripennis] | 133 | 3 |
| Gene.7502 | GMP reductase 1-like [Anoplophora glabripennis] | 86 | 3 |
| Gene.7502 | GMP reductase 1-like [Anoplophora glabripennis] | 13 | 3 |
| Gene.7503 | ribosomal protein S3 [Sitophilus oryzae] | 8 | 3 |
| Gene.7507 | Uncharacterized protein | 39 | 3 |
| Gene.752 | thiamin pyrophosphokinase 1 [Anoplophora glabripennis] | 132 | 3 |
| Gene.7520 | complement component 1 Q subcomponent-binding protein, mitochondrial [Anoplophora glabripennis] | 238 | 3 |
| Gene.7520 | complement component 1 Q subcomponent-binding protein, mitochondrial [Anoplophora glabripennis] | 160 | 3 |
| Gene.7520 | complement component 1 Q subcomponent-binding protein, mitochondrial [Anoplophora glabripennis] | 105 | 3 |
| Gene.7520 | complement component 1 Q subcomponent-binding protein, mitochondrial [Anoplophora glabripennis] | 107 | 3 |
| Gene.7526 | ras-related protein Rac1 [Anoplophora glabripennis] | 96 | 3 |
| Gene.7529 | coatomer subunit alpha [Anoplophora glabripennis] | 12 | 3 |
| Gene.7551 | PREDICTED: non-specific lipid-transfer protein [Aethina tumida] | 269 | 3 |
| Gene.7551 | PREDICTED: non-specific lipid-transfer protein [Aethina tumida] | 119 | 3 |
| Gene.7551 | PREDICTED: non-specific lipid-transfer protein [Aethina tumida] | 170 | 3 |
| Gene.7565 | heat shock protein 21.62 [Harmonia axyridis] | 74 | 3 |
| Gene.7565 | heat shock protein 21.62 [Harmonia axyridis] | 106 | 3 |
| Gene.7565 | heat shock protein 21.62 [Harmonia axyridis] | 129 | 3 |
| Gene.7565 | heat shock protein 21.62 [Harmonia axyridis] | 135 | 3 |
| Gene.7566 | PREDICTED: protein disulfide-isomerase A6 [Tribolium castaneum] | 87 | 3 |
| Gene.7566 | PREDICTED: protein disulfide-isomerase A6 [Tribolium castaneum] | 226 | 3 |
| Gene.7566 | PREDICTED: protein disulfide-isomerase A6 [Tribolium castaneum] | 220 | 3 |
| Gene.7576 | ATP-grasp 2 and/or Ligase CoA domain containing protein [Asbolus verrucosus] | 83 | 3 |
| Gene.7576 | ATP-grasp 2 and/or Ligase CoA domain containing protein [Asbolus verrucosus] | 202 | 3 |
| Gene.7576 | ATP-grasp 2 and/or Ligase CoA domain containing protein [Asbolus verrucosus] | 249 | 3 |
| Gene.7576 | ATP-grasp 2 and/or Ligase CoA domain containing protein [Asbolus verrucosus] | 336 | 3 |
| Gene.7576 | ATP-grasp 2 and/or Ligase CoA domain containing protein [Asbolus verrucosus] | 320 | 3 |
| Gene.7576 | ATP-grasp 2 and/or Ligase CoA domain containing protein [Asbolus verrucosus] | 366 | 3 |
| Gene.7576 | ATP-grasp 2 and/or Ligase CoA domain containing protein [Asbolus verrucosus] | 56 | 3 |
| Gene.7576 | ATP-grasp 2 and/or Ligase CoA domain containing protein [Asbolus verrucosus] | 329 | 3 |
| Gene.7604 | chromobox protein homolog 3 [Anoplophora glabripennis] | 270 | 3 |
| Gene.7624 | nucleolin 1 [Anoplophora glabripennis] | 212 | 3 |
| Gene.7624 | nucleolin 1 [Anoplophora glabripennis] | 412 | 3 |
| Gene.7624 | nucleolin 1 [Anoplophora glabripennis] | 320 | 3 |
| Gene.7624 | nucleolin 1 [Anoplophora glabripennis] | 420 | 3 |
| Gene.7644 | PREDICTED: aldose reductase [Tribolium castaneum] | 70 | 3 |
| Gene.7644 | PREDICTED: aldose reductase [Tribolium castaneum] | 61 | 3 |
| Gene.7644 | PREDICTED: aldose reductase [Tribolium castaneum] | 151 | 3 |
| Gene.7655 | ATP synthase subunit b, mitochondrial [Leptinotarsa decemlineata] | 170 | 3 |
| Gene.7655 | ATP synthase subunit b, mitochondrial [Leptinotarsa decemlineata] | 233 | 3 |
| Gene.7655 | ATP synthase subunit b, mitochondrial [Leptinotarsa decemlineata] | 53 | 3 |
| Gene.7655 | ATP synthase subunit b, mitochondrial [Leptinotarsa decemlineata] | 194 | 3 |
| Gene.7655 | ATP synthase subunit b, mitochondrial [Leptinotarsa decemlineata] | 119 | 3 |
| Gene.7655 | ATP synthase subunit b, mitochondrial [Leptinotarsa decemlineata] | 158 | 3 |
| Gene.7655 | ATP synthase subunit b, mitochondrial [Leptinotarsa decemlineata] | 154 | 3 |
| Gene.7676 | PREDICTED: guanine nucleotide-binding protein G(q) subunit alpha isoform X1 [Tribolium castaneum] | 53 | 3 |
| Gene.7693 | PREDICTED: tryptophan--tRNA ligase, cytoplasmic [Tribolium castaneum] | 64 | 3 |
| Gene.77 | cathepsin L [Anoplophora glabripennis] | 36 | 3 |
| Gene.77 | cathepsin L [Anoplophora glabripennis] | 134 | 3 |
| Gene.77 | cathepsin L [Anoplophora glabripennis] | 57 | 3 |
| Gene.77 | cathepsin L [Anoplophora glabripennis] | 211 | 3 |
| Gene.77 | cathepsin L [Anoplophora glabripennis] | 200 | 3 |
| Gene.77 | cathepsin L [Anoplophora glabripennis] | 216 | 3 |
| Gene.7703 | GTP-binding nuclear protein Ran [Onthophagus taurus] | 98 | 3 |
| Gene.7703 | GTP-binding nuclear protein Ran [Onthophagus taurus] | 11 | 3 |
| Gene.7703 | GTP-binding nuclear protein Ran [Onthophagus taurus] | 70 | 3 |
| Gene.7709 | PREDICTED: LOW QUALITY PROTEIN: adenylosuccinate lyase [Aethina tumida] | 146 | 3 |
| Gene.7714 | heat shock protein beta-1-like [Oncorhynchus mykiss] | 67 | 3 |
| Gene.7714 | heat shock protein beta-1-like [Oncorhynchus mykiss] | 45 | 3 |
| Gene.7714 | heat shock protein beta-1-like [Oncorhynchus mykiss] | 51 | 3 |
| Gene.7721 | PREDICTED: RNA-binding protein squid isoform X2 [Tribolium castaneum] | 38 | 3 |
| Gene.7721 | PREDICTED: RNA-binding protein squid isoform X2 [Tribolium castaneum] | 32 | 3 |
| Gene.7721 | PREDICTED: RNA-binding protein squid isoform X2 [Tribolium castaneum] | 43 | 3 |
| Gene.7734 | PREDICTED: LOW QUALITY PROTEIN: adenosylhomocysteinase [Aethina tumida] | 408 | 3 |
| Gene.7734 | PREDICTED: LOW QUALITY PROTEIN: adenosylhomocysteinase [Aethina tumida] | 188 | 3 |
| Gene.7734 | PREDICTED: LOW QUALITY PROTEIN: adenosylhomocysteinase [Aethina tumida] | 388 | 3 |
| Gene.7771 | electron transfer flavoprotein subunit beta [Agrilus planipennis] | 104 | 3 |
| Gene.7771 | electron transfer flavoprotein subunit beta [Agrilus planipennis] | 161 | 3 |
| Gene.7771 | electron transfer flavoprotein subunit beta [Agrilus planipennis] | 114 | 3 |
| Gene.7771 | electron transfer flavoprotein subunit beta [Agrilus planipennis] | 108 | 3 |
| Gene.7771 | electron transfer flavoprotein subunit beta [Agrilus planipennis] | 17 | 3 |
| Gene.7771 | electron transfer flavoprotein subunit beta [Agrilus planipennis] | 92 | 3 |
| Gene.7771 | electron transfer flavoprotein subunit beta [Agrilus planipennis] | 172 | 3 |
| Gene.7771 | electron transfer flavoprotein subunit beta [Agrilus planipennis] | 224 | 3 |
| Gene.7777 | PREDICTED: asparagine--tRNA ligase, cytoplasmic [Tribolium castaneum] | 247 | 3 |
| Gene.7782 | guanine nucleotide-binding protein subunit beta-like protein isoform X2 [Agrilus planipennis] | 38 | 3 |
| Gene.7782 | guanine nucleotide-binding protein subunit beta-like protein isoform X2 [Agrilus planipennis] | 57 | 3 |
| Gene.7782 | guanine nucleotide-binding protein subunit beta-like protein isoform X2 [Agrilus planipennis] | 96 | 3 |
| Gene.7782 | guanine nucleotide-binding protein subunit beta-like protein isoform X2 [Agrilus planipennis] | 44 | 3 |
| Gene.7784 | PREDICTED: ubiquilin-1 [Tribolium castaneum] | 53 | 3 |
| Gene.7794 | PREDICTED: sulfide:quinone oxidoreductase, mitochondrial [Tribolium castaneum] | 95 | 3 |
| Gene.7808 | PREDICTED: THUMP domain-containing protein 1 homolog [Aethina tumida] | 215 | 3 |
| Gene.7808 | PREDICTED: THUMP domain-containing protein 1 homolog [Aethina tumida] | 115 | 3 |
| Gene.7808 | PREDICTED: THUMP domain-containing protein 1 homolog [Aethina tumida] | 220 | 3 |
| Gene.7808 | PREDICTED: THUMP domain-containing protein 1 homolog [Aethina tumida] | 153 | 3 |
| Gene.7809 | pollen-specific leucine-rich repeat extensin-like protein 1 [Asbolus verrucosus] | 17 | 3 |
| Gene.7815 | probable pyruvate dehydrogenase E1 component subunit alpha, mitochondrial isoform X2 [Leptinotarsa decemlineata] | 315 | 3 |
| Gene.7815 | probable pyruvate dehydrogenase E1 component subunit alpha, mitochondrial isoform X2 [Leptinotarsa decemlineata] | 345 | 3 |
| Gene.7815 | probable pyruvate dehydrogenase E1 component subunit alpha, mitochondrial isoform X2 [Leptinotarsa decemlineata] | 77 | 3 |
| Gene.7815 | probable pyruvate dehydrogenase E1 component subunit alpha, mitochondrial isoform X2 [Leptinotarsa decemlineata] | 317 | 3 |
| Gene.7815 | probable pyruvate dehydrogenase E1 component subunit alpha, mitochondrial isoform X2 [Leptinotarsa decemlineata] | 330 | 3 |
| Gene.7816 | ubiquitin carboxyl-terminal hydrolase 7 isoform X1 [Anoplophora glabripennis] | 393 | 3 |
| Gene.7816 | ubiquitin carboxyl-terminal hydrolase 7 isoform X1 [Anoplophora glabripennis] | 1110 | 3 |
| Gene.7824 | 60S ribosomal protein L37a [Agrilus planipennis] | 22 | 3 |
| Gene.7824 | 60S ribosomal protein L37a [Agrilus planipennis] | 99 | 3 |
| Gene.7824 | 60S ribosomal protein L37a [Agrilus planipennis] | 53 | 3 |
| Gene.7840 | PREDICTED: cytochrome c1, heme protein, mitochondrial [Tribolium castaneum] | 189 | 3 |
| Gene.7840 | PREDICTED: cytochrome c1, heme protein, mitochondrial [Tribolium castaneum] | 44 | 3 |
| Gene.7860 | PREDICTED: lipid storage droplets surface-binding protein 1 isoform X1 [Aethina tumida] | 245 | 3 |
| Gene.7860 | PREDICTED: lipid storage droplets surface-binding protein 1 isoform X1 [Aethina tumida] | 300 | 3 |
| Gene.7871 | hypothetical protein D910_03374 [Dendroctonus ponderosae] | 182 | 3 |
| Gene.7871 | hypothetical protein D910_03374 [Dendroctonus ponderosae] | 151 | 3 |
| Gene.7871 | hypothetical protein D910_03374 [Dendroctonus ponderosae] | 117 | 3 |
| Gene.7878 | alcohol dehydrogenase class-3 [Leptinotarsa decemlineata] | 6 | 3 |
| Gene.7878 | alcohol dehydrogenase class-3 [Leptinotarsa decemlineata] | 340 | 3 |
| Gene.7889 | apoptosis-inducing factor 3 [Asbolus verrucosus] | 399 | 3 |
| Gene.789 | PREDICTED: putative hydroxypyruvate isomerase isoform X2 [Aethina tumida] | 116 | 3 |
| Gene.789 | PREDICTED: putative hydroxypyruvate isomerase isoform X2 [Aethina tumida] | 144 | 3 |
| Gene.789 | PREDICTED: putative hydroxypyruvate isomerase isoform X2 [Aethina tumida] | 100 | 3 |
| Gene.789 | PREDICTED: putative hydroxypyruvate isomerase isoform X2 [Aethina tumida] | 105 | 3 |
| Gene.789 | PREDICTED: putative hydroxypyruvate isomerase isoform X2 [Aethina tumida] | 110 | 3 |
| Gene.7891 | ubiquitin-40S ribosomal protein S27a [Onthophagus taurus] | 27 | 3 |
| Gene.7891 | ubiquitin-40S ribosomal protein S27a [Onthophagus taurus] | 6 | 3 |
| Gene.7891 | ubiquitin-40S ribosomal protein S27a [Onthophagus taurus] | 11 | 3 |
| Gene.7891 | ubiquitin-40S ribosomal protein S27a [Onthophagus taurus] | 33 | 3 |
| Gene.7891 | ubiquitin-40S ribosomal protein S27a [Onthophagus taurus] | 107 | 3 |
| Gene.7891 | ubiquitin-40S ribosomal protein S27a [Onthophagus taurus] | 48 | 3 |
| Gene.7891 | ubiquitin-40S ribosomal protein S27a [Onthophagus taurus] | 99 | 3 |
| Gene.7891 | ubiquitin-40S ribosomal protein S27a [Onthophagus taurus] | 89 | 3 |
| Gene.7891 | ubiquitin-40S ribosomal protein S27a [Onthophagus taurus] | 113 | 3 |
| Gene.7892 | Polyubiquitin 11, partial [Trichinella nelsoni] | 3 | 3 |
| Gene.7907 | Uncharacterized protein | 112 | 3 |
| Gene.7907 | Uncharacterized protein | 90 | 3 |
| Gene.7917 | ribosomal protein S3 [Tenebrio molitor] | 75 | 3 |
| Gene.7917 | ribosomal protein S3 [Tenebrio molitor] | 7 | 3 |
| Gene.7917 | ribosomal protein S3 [Tenebrio molitor] | 10 | 3 |
| Gene.792 | imaginal disc growth factor 4 precursor [Tribolium castaneum] | 88 | 3 |
| Gene.792 | imaginal disc growth factor 4 precursor [Tribolium castaneum] | 194 | 3 |
| Gene.7929 | PREDICTED: heat shock 70 kDa protein cognate 3 [Dendroctonus ponderosae] | 370 | 3 |
| Gene.7929 | PREDICTED: heat shock 70 kDa protein cognate 3 [Dendroctonus ponderosae] | 593 | 3 |
| Gene.7929 | PREDICTED: heat shock 70 kDa protein cognate 3 [Dendroctonus ponderosae] | 523 | 3 |
| Gene.7929 | PREDICTED: heat shock 70 kDa protein cognate 3 [Dendroctonus ponderosae] | 579 | 3 |
| Gene.7929 | PREDICTED: heat shock 70 kDa protein cognate 3 [Dendroctonus ponderosae] | 543 | 3 |
| Gene.7929 | PREDICTED: heat shock 70 kDa protein cognate 3 [Dendroctonus ponderosae] | 617 | 3 |
| Gene.7929 | PREDICTED: heat shock 70 kDa protein cognate 3 [Dendroctonus ponderosae] | 119 | 3 |
| Gene.7929 | PREDICTED: heat shock 70 kDa protein cognate 3 [Dendroctonus ponderosae] | 154 | 3 |
| Gene.7929 | PREDICTED: heat shock 70 kDa protein cognate 3 [Dendroctonus ponderosae] | 481 | 3 |
| Gene.7929 | PREDICTED: heat shock 70 kDa protein cognate 3 [Dendroctonus ponderosae] | 376 | 3 |
| Gene.7929 | PREDICTED: heat shock 70 kDa protein cognate 3 [Dendroctonus ponderosae] | 344 | 3 |
| Gene.7929 | PREDICTED: heat shock 70 kDa protein cognate 3 [Dendroctonus ponderosae] | 352 | 3 |
| Gene.7929 | PREDICTED: heat shock 70 kDa protein cognate 3 [Dendroctonus ponderosae] | 553 | 3 |
| Gene.7929 | PREDICTED: heat shock 70 kDa protein cognate 3 [Dendroctonus ponderosae] | 194 | 3 |
| Gene.7929 | PREDICTED: heat shock 70 kDa protein cognate 3 [Dendroctonus ponderosae] | 138 | 3 |
| Gene.7929 | PREDICTED: heat shock 70 kDa protein cognate 3 [Dendroctonus ponderosae] | 326 | 3 |
| Gene.7929 | PREDICTED: heat shock 70 kDa protein cognate 3 [Dendroctonus ponderosae] | 213 | 3 |
| Gene.7929 | PREDICTED: heat shock 70 kDa protein cognate 3 [Dendroctonus ponderosae] | 97 | 3 |
| Gene.7929 | PREDICTED: heat shock 70 kDa protein cognate 3 [Dendroctonus ponderosae] | 601 | 3 |
| Gene.7929 | PREDICTED: heat shock 70 kDa protein cognate 3 [Dendroctonus ponderosae] | 163 | 3 |
| Gene.7929 | PREDICTED: heat shock 70 kDa protein cognate 3 [Dendroctonus ponderosae] | 382 | 3 |
| Gene.7929 | PREDICTED: heat shock 70 kDa protein cognate 3 [Dendroctonus ponderosae] | 603 | 3 |
| Gene.793 | PREDICTED: eukaryotic translation initiation factor 2 subunit 3, X-linked isoform X2 [Tribolium castaneum] | 60 | 3 |
| Gene.793 | PREDICTED: eukaryotic translation initiation factor 2 subunit 3, X-linked isoform X2 [Tribolium castaneum] | 18 | 3 |
| Gene.793 | PREDICTED: eukaryotic translation initiation factor 2 subunit 3, X-linked isoform X2 [Tribolium castaneum] | 91 | 3 |
| Gene.793 | PREDICTED: eukaryotic translation initiation factor 2 subunit 3, X-linked isoform X2 [Tribolium castaneum] | 422 | 3 |
| Gene.7933 | tudor domain-containing protein 7 isoform X1 [Anoplophora glabripennis] | 483 | 3 |
| Gene.7938 | PREDICTED: spermine synthase isoform X1 [Tribolium castaneum] | 42 | 3 |
| Gene.7938 | PREDICTED: spermine synthase isoform X1 [Tribolium castaneum] | 302 | 3 |
| Gene.7938 | PREDICTED: spermine synthase isoform X1 [Tribolium castaneum] | 305 | 3 |
| Gene.7938 | PREDICTED: spermine synthase isoform X1 [Tribolium castaneum] | 148 | 3 |
| Gene.7945 | fatty acid-binding protein, muscle isoform X2 [Anoplophora glabripennis] | 119 | 3 |
| Gene.7945 | fatty acid-binding protein, muscle isoform X2 [Anoplophora glabripennis] | 87 | 3 |
| Gene.7945 | fatty acid-binding protein, muscle isoform X2 [Anoplophora glabripennis] | 130 | 3 |
| Gene.7945 | fatty acid-binding protein, muscle isoform X2 [Anoplophora glabripennis] | 125 | 3 |
| Gene.7945 | fatty acid-binding protein, muscle isoform X2 [Anoplophora glabripennis] | 144 | 3 |
| Gene.7945 | fatty acid-binding protein, muscle isoform X2 [Anoplophora glabripennis] | 73 | 3 |
| Gene.7945 | fatty acid-binding protein, muscle isoform X2 [Anoplophora glabripennis] | 94 | 3 |
| Gene.7954 | eukaryotic translation initiation factor 3 subunit C [Anoplophora glabripennis] | 433 | 3 |
| Gene.7954 | eukaryotic translation initiation factor 3 subunit C [Anoplophora glabripennis] | 837 | 3 |
| Gene.7954 | eukaryotic translation initiation factor 3 subunit C [Anoplophora glabripennis] | 744 | 3 |
| Gene.7958 | U6 snRNA-associated Sm-like protein LSm7 [Asbolus verrucosus] | 34 | 3 |
| Gene.7958 | U6 snRNA-associated Sm-like protein LSm7 [Asbolus verrucosus] | 39 | 3 |
| Gene.7965 | dihydropyrimidinase [Anoplophora glabripennis] | 152 | 3 |
| Gene.7965 | dihydropyrimidinase [Anoplophora glabripennis] | 461 | 3 |
| Gene.7965 | dihydropyrimidinase [Anoplophora glabripennis] | 346 | 3 |
| Gene.7967 | uncharacterized protein LOC108916348 [Anoplophora glabripennis] | 37 | 3 |
| Gene.7968 | uncharacterized protein LOC110861194 [Folsomia candida] | 127 | 3 |
| Gene.7995 | PREDICTED: phosphorylated adapter RNA export protein [Tribolium castaneum] | 194 | 3 |
| Gene.7997 | Y-box factor homolog isoform X3 [Leptinotarsa decemlineata] | 149 | 3 |
| Gene.7997 | Y-box factor homolog isoform X3 [Leptinotarsa decemlineata] | 39 | 3 |
| Gene.7997 | Y-box factor homolog isoform X3 [Leptinotarsa decemlineata] | 34 | 3 |
| Gene.7997 | Y-box factor homolog isoform X3 [Leptinotarsa decemlineata] | 28 | 3 |
| Gene.7997 | Y-box factor homolog isoform X3 [Leptinotarsa decemlineata] | 63 | 3 |
| Gene.8007 | B-cell receptor-associated protein 31 [Anoplophora glabripennis] | 158 | 3 |
| Gene.8007 | B-cell receptor-associated protein 31 [Anoplophora glabripennis] | 181 | 3 |
| Gene.8007 | B-cell receptor-associated protein 31 [Anoplophora glabripennis] | 214 | 3 |
| Gene.8007 | B-cell receptor-associated protein 31 [Anoplophora glabripennis] | 37 | 3 |
| Gene.8007 | B-cell receptor-associated protein 31 [Anoplophora glabripennis] | 168 | 3 |
| Gene.8016 | UCR TM, Rieske, and/or Ubiq-Cytc-red N domain containing protein [Asbolus verrucosus] | 180 | 3 |
| Gene.8016 | UCR TM, Rieske, and/or Ubiq-Cytc-red N domain containing protein [Asbolus verrucosus] | 171 | 3 |
| Gene.8016 | UCR TM, Rieske, and/or Ubiq-Cytc-red N domain containing protein [Asbolus verrucosus] | 176 | 3 |
| Gene.8016 | UCR TM, Rieske, and/or Ubiq-Cytc-red N domain containing protein [Asbolus verrucosus] | 110 | 3 |
| Gene.8017 | glycogen synthase [Harmonia axyridis] | 315 | 3 |
| Gene.8017 | glycogen synthase [Harmonia axyridis] | 298 | 3 |
| Gene.8017 | glycogen synthase [Harmonia axyridis] | 247 | 3 |
| Gene.8021 | ribosomal protein L4e [Biphyllus lunatus] | 271 | 3 |
| Gene.8021 | ribosomal protein L4e [Biphyllus lunatus] | 286 | 3 |
| Gene.8021 | ribosomal protein L4e [Biphyllus lunatus] | 261 | 3 |
| Gene.8021 | ribosomal protein L4e [Biphyllus lunatus] | 168 | 3 |
| Gene.8021 | ribosomal protein L4e [Biphyllus lunatus] | 358 | 3 |
| Gene.8021 | ribosomal protein L4e [Biphyllus lunatus] | 109 | 3 |
| Gene.8021 | ribosomal protein L4e [Biphyllus lunatus] | 265 | 3 |
| Gene.8021 | ribosomal protein L4e [Biphyllus lunatus] | 216 | 3 |
| Gene.8021 | ribosomal protein L4e [Biphyllus lunatus] | 242 | 3 |
| Gene.8027 | uncharacterized protein LOC111502893 [Leptinotarsa decemlineata] | 163 | 3 |
| Gene.8038 | PREDICTED: bleomycin hydrolase [Tribolium castaneum] | 16 | 3 |
| Gene.8047 | PREDICTED: proteasome subunit beta type-7-like [Aethina tumida] | 233 | 3 |
| Gene.8065 | PREDICTED: OCIA domain-containing protein 1 [Tribolium castaneum] | 13 | 3 |
| Gene.8065 | PREDICTED: OCIA domain-containing protein 1 [Tribolium castaneum] | 199 | 3 |
| Gene.8074 | PREDICTED: gamma-interferon-inducible lysosomal thiol reductase [Tribolium castaneum] | 142 | 3 |
| Gene.8074 | PREDICTED: gamma-interferon-inducible lysosomal thiol reductase [Tribolium castaneum] | 148 | 3 |
| Gene.8074 | PREDICTED: gamma-interferon-inducible lysosomal thiol reductase [Tribolium castaneum] | 242 | 3 |
| Gene.8081 | ATP synthase subunit gamma, mitochondrial [Leptinotarsa decemlineata] | 119 | 3 |
| Gene.8081 | ATP synthase subunit gamma, mitochondrial [Leptinotarsa decemlineata] | 125 | 3 |
| Gene.8081 | ATP synthase subunit gamma, mitochondrial [Leptinotarsa decemlineata] | 111 | 3 |
| Gene.8081 | ATP synthase subunit gamma, mitochondrial [Leptinotarsa decemlineata] | 78 | 3 |
| Gene.8081 | ATP synthase subunit gamma, mitochondrial [Leptinotarsa decemlineata] | 54 | 3 |
| Gene.8087 | PREDICTED: adenylosuccinate synthetase-like [Aethina tumida] | 464 | 3 |
| Gene.8087 | PREDICTED: adenylosuccinate synthetase-like [Aethina tumida] | 472 | 3 |
| Gene.8087 | PREDICTED: adenylosuccinate synthetase-like [Aethina tumida] | 140 | 3 |
| Gene.8091 | translation initiation factor eIF-2B subunit alpha [Leptinotarsa decemlineata] | 104 | 3 |
| Gene.8096 | PREDICTED: ADP,ATP carrier protein 1 [Tribolium castaneum] | 271 | 3 |
| Gene.8096 | PREDICTED: ADP,ATP carrier protein 1 [Tribolium castaneum] | 74 | 3 |
| Gene.8096 | PREDICTED: ADP,ATP carrier protein 1 [Tribolium castaneum] | 274 | 3 |
| Gene.8096 | PREDICTED: ADP,ATP carrier protein 1 [Tribolium castaneum] | 63 | 3 |
| Gene.8096 | PREDICTED: ADP,ATP carrier protein 1 [Tribolium castaneum] | 21 | 3 |
| Gene.8096 | PREDICTED: ADP,ATP carrier protein 1 [Tribolium castaneum] | 263 | 3 |
| Gene.8096 | PREDICTED: ADP,ATP carrier protein 1 [Tribolium castaneum] | 107 | 3 |
| Gene.8096 | PREDICTED: ADP,ATP carrier protein 1 [Tribolium castaneum] | 158 | 3 |
| Gene.8096 | PREDICTED: ADP,ATP carrier protein 1 [Tribolium castaneum] | 60 | 3 |
| Gene.8096 | PREDICTED: ADP,ATP carrier protein 1 [Tribolium castaneum] | 177 | 3 |
| Gene.8096 | PREDICTED: ADP,ATP carrier protein 1 [Tribolium castaneum] | 257 | 3 |
| Gene.8096 | PREDICTED: ADP,ATP carrier protein 1 [Tribolium castaneum] | 54 | 3 |
| Gene.8098 | hypothetical protein, partial [Rhynchophorus ferrugineus] | 53 | 3 |
| Gene.8098 | hypothetical protein, partial [Rhynchophorus ferrugineus] | 93 | 3 |
| Gene.8098 | hypothetical protein, partial [Rhynchophorus ferrugineus] | 106 | 3 |
| Gene.8098 | hypothetical protein, partial [Rhynchophorus ferrugineus] | 64 | 3 |
| Gene.8098 | hypothetical protein, partial [Rhynchophorus ferrugineus] | 97 | 3 |
| Gene.8098 | hypothetical protein, partial [Rhynchophorus ferrugineus] | 148 | 3 |
| Gene.8098 | hypothetical protein, partial [Rhynchophorus ferrugineus] | 167 | 3 |
| Gene.8102 | glucosidase 2 subunit beta-like [Leptinotarsa decemlineata] | 183 | 3 |
| Gene.8104 | PREDICTED: high mobility group protein DSP1 [Nicrophorus vespilloides] | 398 | 3 |
| Gene.8104 | PREDICTED: high mobility group protein DSP1 [Nicrophorus vespilloides] | 382 | 3 |
| Gene.8104 | PREDICTED: high mobility group protein DSP1 [Nicrophorus vespilloides] | 300 | 3 |
| Gene.8104 | PREDICTED: high mobility group protein DSP1 [Nicrophorus vespilloides] | 396 | 3 |
| Gene.8115 | mitochondrial-processing peptidase subunit beta [Anoplophora glabripennis] | 443 | 3 |
| Gene.8115 | mitochondrial-processing peptidase subunit beta [Anoplophora glabripennis] | 318 | 3 |
| Gene.8127 | phospholipid scramblase 2 isoform X1 [Anoplophora glabripennis] | 256 | 3 |
| Gene.8135 | PREDICTED: calreticulin [Tribolium castaneum] | 203 | 3 |
| Gene.8135 | PREDICTED: calreticulin [Tribolium castaneum] | 359 | 3 |
| Gene.8135 | PREDICTED: calreticulin [Tribolium castaneum] | 206 | 3 |
| Gene.8135 | PREDICTED: calreticulin [Tribolium castaneum] | 61 | 3 |
| Gene.8135 | PREDICTED: calreticulin [Tribolium castaneum] | 108 | 3 |
| Gene.8135 | PREDICTED: calreticulin [Tribolium castaneum] | 52 | 3 |
| Gene.8135 | PREDICTED: calreticulin [Tribolium castaneum] | 354 | 3 |
| Gene.8135 | PREDICTED: calreticulin [Tribolium castaneum] | 45 | 3 |
| Gene.8135 | PREDICTED: calreticulin [Tribolium castaneum] | 37 | 3 |
| Gene.8135 | PREDICTED: calreticulin [Tribolium castaneum] | 275 | 3 |
| Gene.8135 | PREDICTED: calreticulin [Tribolium castaneum] | 31 | 3 |
| Gene.8139 | sepiapterin reductase [Anoplophora glabripennis] | 247 | 3 |
| Gene.814 | PREDICTED: uncharacterized protein LOC109597149 isoform X2 [Aethina tumida] | 70 | 3 |
| Gene.814 | PREDICTED: uncharacterized protein LOC109597149 isoform X2 [Aethina tumida] | 121 | 3 |
| Gene.8161 | PREDICTED: LOW QUALITY PROTEIN: elongation factor 1-gamma [Aethina tumida] | 421 | 3 |
| Gene.8161 | PREDICTED: LOW QUALITY PROTEIN: elongation factor 1-gamma [Aethina tumida] | 294 | 3 |
| Gene.8161 | PREDICTED: LOW QUALITY PROTEIN: elongation factor 1-gamma [Aethina tumida] | 39 | 3 |
| Gene.8161 | PREDICTED: LOW QUALITY PROTEIN: elongation factor 1-gamma [Aethina tumida] | 198 | 3 |
| Gene.8161 | PREDICTED: LOW QUALITY PROTEIN: elongation factor 1-gamma [Aethina tumida] | 432 | 3 |
| Gene.8161 | PREDICTED: LOW QUALITY PROTEIN: elongation factor 1-gamma [Aethina tumida] | 30 | 3 |
| Gene.8161 | PREDICTED: LOW QUALITY PROTEIN: elongation factor 1-gamma [Aethina tumida] | 285 | 3 |
| Gene.8161 | PREDICTED: LOW QUALITY PROTEIN: elongation factor 1-gamma [Aethina tumida] | 55 | 3 |
| Gene.8161 | PREDICTED: LOW QUALITY PROTEIN: elongation factor 1-gamma [Aethina tumida] | 404 | 3 |
| Gene.8167 | malate dehydrogenase, cytoplasmic [Leptinotarsa decemlineata] | 149 | 3 |
| Gene.8167 | malate dehydrogenase, cytoplasmic [Leptinotarsa decemlineata] | 259 | 3 |
| Gene.8167 | malate dehydrogenase, cytoplasmic [Leptinotarsa decemlineata] | 118 | 3 |
| Gene.8167 | malate dehydrogenase, cytoplasmic [Leptinotarsa decemlineata] | 265 | 3 |
| Gene.8167 | malate dehydrogenase, cytoplasmic [Leptinotarsa decemlineata] | 317 | 3 |
| Gene.8167 | malate dehydrogenase, cytoplasmic [Leptinotarsa decemlineata] | 214 | 3 |
| Gene.8167 | malate dehydrogenase, cytoplasmic [Leptinotarsa decemlineata] | 229 | 3 |
| Gene.8167 | malate dehydrogenase, cytoplasmic [Leptinotarsa decemlineata] | 110 | 3 |
| Gene.8194 | N-acetylneuraminate lyase-like isoform X2 [Anoplophora glabripennis] | 84 | 3 |
| Gene.8194 | N-acetylneuraminate lyase-like isoform X2 [Anoplophora glabripennis] | 312 | 3 |
| Gene.825 | TPR 11 domain containing protein [Asbolus verrucosus] | 109 | 3 |
| Gene.825 | TPR 11 domain containing protein [Asbolus verrucosus] | 182 | 3 |
| Gene.825 | TPR 11 domain containing protein [Asbolus verrucosus] | 54 | 3 |
| Gene.8252 | T-complex protein 1 subunit eta [Anoplophora glabripennis] | 145 | 3 |
| Gene.8257 | 56 kDa early-staged encapsulation-inducing protein [Tenebrio molitor] | 40 | 3 |
| Gene.8257 | 56 kDa early-staged encapsulation-inducing protein [Tenebrio molitor] | 139 | 3 |
| Gene.8257 | 56 kDa early-staged encapsulation-inducing protein [Tenebrio molitor] | 68 | 3 |
| Gene.8257 | 56 kDa early-staged encapsulation-inducing protein [Tenebrio molitor] | 103 | 3 |
| Gene.8257 | 56 kDa early-staged encapsulation-inducing protein [Tenebrio molitor] | 320 | 3 |
| Gene.8257 | 56 kDa early-staged encapsulation-inducing protein [Tenebrio molitor] | 46 | 3 |
| Gene.8257 | 56 kDa early-staged encapsulation-inducing protein [Tenebrio molitor] | 132 | 3 |
| Gene.8261 | PREDICTED: cytochrome c oxidase subunit 6C-like [Dendroctonus ponderosae] | 92 | 3 |
| Gene.8261 | PREDICTED: cytochrome c oxidase subunit 6C-like [Dendroctonus ponderosae] | 88 | 3 |
| Gene.8261 | PREDICTED: cytochrome c oxidase subunit 6C-like [Dendroctonus ponderosae] | 40 | 3 |
| Gene.8271 | transmembrane 9 superfamily member 2 [Agrilus planipennis] | 413 | 3 |
| Gene.8272 | extracellular globin-E1-like isoform X1 [Leptinotarsa decemlineata] | 60 | 3 |
| Gene.8272 | extracellular globin-E1-like isoform X1 [Leptinotarsa decemlineata] | 39 | 3 |
| Gene.8288 | cofilin/actin-depolymerizing factor homolog [Anoplophora glabripennis] | 19 | 3 |
| Gene.8288 | cofilin/actin-depolymerizing factor homolog [Anoplophora glabripennis] | 41 | 3 |
| Gene.8316 | PREDICTED: inorganic pyrophosphatase isoform X2 [Tribolium castaneum] | 102 | 3 |
| Gene.8316 | PREDICTED: inorganic pyrophosphatase isoform X2 [Tribolium castaneum] | 273 | 3 |
| Gene.8316 | PREDICTED: inorganic pyrophosphatase isoform X2 [Tribolium castaneum] | 239 | 3 |
| Gene.8316 | PREDICTED: inorganic pyrophosphatase isoform X2 [Tribolium castaneum] | 108 | 3 |
| Gene.8316 | PREDICTED: inorganic pyrophosphatase isoform X2 [Tribolium castaneum] | 236 | 3 |
| Gene.8316 | PREDICTED: inorganic pyrophosphatase isoform X2 [Tribolium castaneum] | 270 | 3 |
| Gene.8318 | PGM PMM I domain containing protein [Asbolus verrucosus] | 197 | 3 |
| Gene.8318 | PGM PMM I domain containing protein [Asbolus verrucosus] | 466 | 3 |
| Gene.8318 | PGM PMM I domain containing protein [Asbolus verrucosus] | 204 | 3 |
| Gene.8318 | PGM PMM I domain containing protein [Asbolus verrucosus] | 486 | 3 |
| Gene.8318 | PGM PMM I domain containing protein [Asbolus verrucosus] | 9 | 3 |
| Gene.8318 | PGM PMM I domain containing protein [Asbolus verrucosus] | 489 | 3 |
| Gene.8318 | PGM PMM I domain containing protein [Asbolus verrucosus] | 348 | 3 |
| Gene.8318 | PGM PMM I domain containing protein [Asbolus verrucosus] | 421 | 3 |
| Gene.8318 | PGM PMM I domain containing protein [Asbolus verrucosus] | 278 | 3 |
| Gene.8318 | PGM PMM I domain containing protein [Asbolus verrucosus] | 352 | 3 |
| Gene.8318 | PGM PMM I domain containing protein [Asbolus verrucosus] | 459 | 3 |
| Gene.8318 | PGM PMM I domain containing protein [Asbolus verrucosus] | 151 | 3 |
| Gene.8318 | PGM PMM I domain containing protein [Asbolus verrucosus] | 449 | 3 |
| Gene.8322 | PREDICTED: uncharacterized protein LOC109595590 [Aethina tumida] | 37 | 3 |
| Gene.8322 | PREDICTED: uncharacterized protein LOC109595590 [Aethina tumida] | 13 | 3 |
| Gene.8322 | PREDICTED: uncharacterized protein LOC109595590 [Aethina tumida] | 223 | 3 |
| Gene.8322 | PREDICTED: uncharacterized protein LOC109595590 [Aethina tumida] | 404 | 3 |
| Gene.8326 | heat shock protein 16.25 [Harmonia axyridis] | 104 | 3 |
| Gene.8333 | PREDICTED: 60S ribosomal protein L5 [Tribolium castaneum] | 27 | 3 |
| Gene.8333 | PREDICTED: 60S ribosomal protein L5 [Tribolium castaneum] | 188 | 3 |
| Gene.8333 | PREDICTED: 60S ribosomal protein L5 [Tribolium castaneum] | 220 | 3 |
| Gene.8333 | PREDICTED: 60S ribosomal protein L5 [Tribolium castaneum] | 48 | 3 |
| Gene.8333 | PREDICTED: 60S ribosomal protein L5 [Tribolium castaneum] | 277 | 3 |
| Gene.8333 | PREDICTED: 60S ribosomal protein L5 [Tribolium castaneum] | 164 | 3 |
| Gene.8333 | PREDICTED: 60S ribosomal protein L5 [Tribolium castaneum] | 73 | 3 |
| Gene.8333 | PREDICTED: 60S ribosomal protein L5 [Tribolium castaneum] | 43 | 3 |
| Gene.8333 | PREDICTED: 60S ribosomal protein L5 [Tribolium castaneum] | 5 | 3 |
| Gene.8337 | PREDICTED: cytochrome c oxidase subunit 5B, mitochondrial [Tribolium castaneum] | 50 | 3 |
| Gene.8337 | PREDICTED: cytochrome c oxidase subunit 5B, mitochondrial [Tribolium castaneum] | 44 | 3 |
| Gene.834 | PREDICTED: very long-chain specific acyl-CoA dehydrogenase, mitochondrial [Tribolium castaneum] | 39 | 3 |
| Gene.834 | PREDICTED: very long-chain specific acyl-CoA dehydrogenase, mitochondrial [Tribolium castaneum] | 486 | 3 |
| Gene.834 | PREDICTED: very long-chain specific acyl-CoA dehydrogenase, mitochondrial [Tribolium castaneum] | 253 | 3 |
| Gene.834 | PREDICTED: very long-chain specific acyl-CoA dehydrogenase, mitochondrial [Tribolium castaneum] | 277 | 3 |
| Gene.834 | PREDICTED: very long-chain specific acyl-CoA dehydrogenase, mitochondrial [Tribolium castaneum] | 617 | 3 |
| Gene.834 | PREDICTED: very long-chain specific acyl-CoA dehydrogenase, mitochondrial [Tribolium castaneum] | 107 | 3 |
| Gene.834 | PREDICTED: very long-chain specific acyl-CoA dehydrogenase, mitochondrial [Tribolium castaneum] | 604 | 3 |
| Gene.834 | PREDICTED: very long-chain specific acyl-CoA dehydrogenase, mitochondrial [Tribolium castaneum] | 535 | 3 |
| Gene.834 | PREDICTED: very long-chain specific acyl-CoA dehydrogenase, mitochondrial [Tribolium castaneum] | 598 | 3 |
| Gene.834 | PREDICTED: very long-chain specific acyl-CoA dehydrogenase, mitochondrial [Tribolium castaneum] | 499 | 3 |
| Gene.834 | PREDICTED: very long-chain specific acyl-CoA dehydrogenase, mitochondrial [Tribolium castaneum] | 101 | 3 |
| Gene.834 | PREDICTED: very long-chain specific acyl-CoA dehydrogenase, mitochondrial [Tribolium castaneum] | 178 | 3 |
| Gene.834 | PREDICTED: very long-chain specific acyl-CoA dehydrogenase, mitochondrial [Tribolium castaneum] | 611 | 3 |
| Gene.834 | PREDICTED: very long-chain specific acyl-CoA dehydrogenase, mitochondrial [Tribolium castaneum] | 65 | 3 |
| Gene.8344 | PREDICTED: MAP7 domain-containing protein 1-like isoform X5 [Dendroctonus ponderosae] | 620 | 3 |
| Gene.8351 | probable cysteine desulfurase, mitochondrial [Anoplophora glabripennis] | 97 | 3 |
| Gene.8352 | PREDICTED: CUGBP Elav-like family member 1 isoform X1 [Tribolium castaneum] | 284 | 3 |
| Gene.8382 | GST [Lygus lineolaris] | 77 | 3 |
| Gene.8382 | GST [Lygus lineolaris] | 80 | 3 |
| Gene.8382 | GST [Lygus lineolaris] | 31 | 3 |
| Gene.8388 | ADP-ribosylation factor 1 isoform X1 [Penaeus vannamei] | 104 | 3 |
| Gene.8388 | ADP-ribosylation factor 1 isoform X1 [Penaeus vannamei] | 36 | 3 |
| Gene.839 | PREDICTED: ATPase WRNIP1 [Tribolium castaneum] | 304 | 3 |
| Gene.839 | PREDICTED: ATPase WRNIP1 [Tribolium castaneum] | 251 | 3 |
| Gene.839 | PREDICTED: ATPase WRNIP1 [Tribolium castaneum] | 232 | 3 |
| Gene.8399 | ribosomal protein S24e [Meladema coriacea] | 100 | 3 |
| Gene.8403 | PREDICTED: myosin heavy chain, non-muscle isoform X1 [Dendroctonus ponderosae] | 22 | 3 |
| Gene.8403 | PREDICTED: myosin heavy chain, non-muscle isoform X1 [Dendroctonus ponderosae] | 492 | 3 |
| Gene.8403 | PREDICTED: myosin heavy chain, non-muscle isoform X1 [Dendroctonus ponderosae] | 206 | 3 |
| Gene.8407 | PREDICTED: voltage-dependent anion-selective channel isoform X1 [Tribolium castaneum] | 27 | 3 |
| Gene.8407 | PREDICTED: voltage-dependent anion-selective channel isoform X1 [Tribolium castaneum] | 60 | 3 |
| Gene.8407 | PREDICTED: voltage-dependent anion-selective channel isoform X1 [Tribolium castaneum] | 11 | 3 |
| Gene.8407 | PREDICTED: voltage-dependent anion-selective channel isoform X1 [Tribolium castaneum] | 64 | 3 |
| Gene.8408 | troponin C isoform X3 [Anoplophora glabripennis] | 45 | 3 |
| Gene.8408 | troponin C isoform X3 [Anoplophora glabripennis] | 86 | 3 |
| Gene.8418 | PREDICTED: 15-hydroxyprostaglandin dehydrogenase [NAD(+)]-like [Aethina tumida] | 74 | 3 |
| Gene.8424 | PREDICTED: NAD/NADP-dependent betaine aldehyde dehydrogenase [Tribolium castaneum] | 34 | 3 |
| Gene.8424 | PREDICTED: NAD/NADP-dependent betaine aldehyde dehydrogenase [Tribolium castaneum] | 532 | 3 |
| Gene.8427 | PREDICTED: EH domain-containing protein 3-like [Tribolium castaneum] | 296 | 3 |
| Gene.8427 | PREDICTED: EH domain-containing protein 3-like [Tribolium castaneum] | 210 | 3 |
| Gene.8430 | putative juvenile hormone acid methyltransferase, partial [Polypedilum vanderplanki] | 6 | 3 |
| Gene.8430 | putative juvenile hormone acid methyltransferase, partial [Polypedilum vanderplanki] | 90 | 3 |
| Gene.8430 | putative juvenile hormone acid methyltransferase, partial [Polypedilum vanderplanki] | 95 | 3 |
| Gene.8430 | putative juvenile hormone acid methyltransferase, partial [Polypedilum vanderplanki] | 186 | 3 |
| Gene.8430 | putative juvenile hormone acid methyltransferase, partial [Polypedilum vanderplanki] | 100 | 3 |
| Gene.8430 | putative juvenile hormone acid methyltransferase, partial [Polypedilum vanderplanki] | 82 | 3 |
| Gene.8445 | nascent polypeptide-associated complex subunit alpha-like isoform X1 [Anoplophora glabripennis] | 152 | 3 |
| Gene.8450 | unknown [Dendroctonus ponderosae] | 159 | 3 |
| Gene.8450 | unknown [Dendroctonus ponderosae] | 265 | 3 |
| Gene.8450 | unknown [Dendroctonus ponderosae] | 68 | 3 |
| Gene.8450 | unknown [Dendroctonus ponderosae] | 267 | 3 |
| Gene.8450 | unknown [Dendroctonus ponderosae] | 220 | 3 |
| Gene.8456 | RNA binding protein [Oryctes borbonicus] | 36 | 3 |
| Gene.8465 | Uncharacterized protein | 205 | 3 |
| Gene.8465 | Uncharacterized protein | 202 | 3 |
| Gene.8465 | Uncharacterized protein | 258 | 3 |
| Gene.8474 | Uncharacterized protein | 304 | 3 |
| Gene.8474 | Uncharacterized protein | 409 | 3 |
| Gene.8474 | Uncharacterized protein | 224 | 3 |
| Gene.8481 | PREDICTED: arginine kinase isoform X1 [Tribolium castaneum] | 237 | 3 |
| Gene.8481 | PREDICTED: arginine kinase isoform X1 [Tribolium castaneum] | 310 | 3 |
| Gene.8481 | PREDICTED: arginine kinase isoform X1 [Tribolium castaneum] | 210 | 3 |
| Gene.8481 | PREDICTED: arginine kinase isoform X1 [Tribolium castaneum] | 38 | 3 |
| Gene.8481 | PREDICTED: arginine kinase isoform X1 [Tribolium castaneum] | 201 | 3 |
| Gene.8481 | PREDICTED: arginine kinase isoform X1 [Tribolium castaneum] | 349 | 3 |
| Gene.8481 | PREDICTED: arginine kinase isoform X1 [Tribolium castaneum] | 45 | 3 |
| Gene.8481 | PREDICTED: arginine kinase isoform X1 [Tribolium castaneum] | 372 | 3 |
| Gene.8481 | PREDICTED: arginine kinase isoform X1 [Tribolium castaneum] | 196 | 3 |
| Gene.8502 | PREDICTED: LOW QUALITY PROTEIN: probable H/ACA ribonucleoprotein complex subunit 1 [Aethina tumida] | 104 | 3 |
| Gene.8502 | PREDICTED: LOW QUALITY PROTEIN: probable H/ACA ribonucleoprotein complex subunit 1 [Aethina tumida] | 107 | 3 |
| Gene.8507 | moesin/ezrin/radixin homolog 1 isoform X2 [Anoplophora glabripennis] | 373 | 3 |
| Gene.8507 | moesin/ezrin/radixin homolog 1 isoform X2 [Anoplophora glabripennis] | 400 | 3 |
| Gene.8507 | moesin/ezrin/radixin homolog 1 isoform X2 [Anoplophora glabripennis] | 417 | 3 |
| Gene.8507 | moesin/ezrin/radixin homolog 1 isoform X2 [Anoplophora glabripennis] | 79 | 3 |
| Gene.8507 | moesin/ezrin/radixin homolog 1 isoform X2 [Anoplophora glabripennis] | 527 | 3 |
| Gene.8507 | moesin/ezrin/radixin homolog 1 isoform X2 [Anoplophora glabripennis] | 431 | 3 |
| Gene.8507 | moesin/ezrin/radixin homolog 1 isoform X2 [Anoplophora glabripennis] | 254 | 3 |
| Gene.8507 | moesin/ezrin/radixin homolog 1 isoform X2 [Anoplophora glabripennis] | 379 | 3 |
| Gene.8516 | PREDICTED: NADH-ubiquinone oxidoreductase 49 kDa subunit [Tribolium castaneum] | 373 | 3 |
| Gene.8516 | PREDICTED: NADH-ubiquinone oxidoreductase 49 kDa subunit [Tribolium castaneum] | 310 | 3 |
| Gene.8516 | PREDICTED: NADH-ubiquinone oxidoreductase 49 kDa subunit [Tribolium castaneum] | 368 | 3 |
| Gene.8518 | PREDICTED: dolichyl-diphosphooligosaccharide--protein glycosyltransferase subunit 1 [Tribolium castaneum] | 397 | 3 |
| Gene.8522 | 40S ribosomal protein S2 [Harpegnathos saltator] | 52 | 3 |
| Gene.8522 | 40S ribosomal protein S2 [Harpegnathos saltator] | 174 | 3 |
| Gene.8522 | 40S ribosomal protein S2 [Harpegnathos saltator] | 95 | 3 |
| Gene.8522 | 40S ribosomal protein S2 [Harpegnathos saltator] | 198 | 3 |
| Gene.8522 | 40S ribosomal protein S2 [Harpegnathos saltator] | 101 | 3 |
| Gene.8522 | 40S ribosomal protein S2 [Harpegnathos saltator] | 132 | 3 |
| Gene.8523 | 40S ribosomal protein S2 [Ctenocephalides felis] | 56 | 3 |
| Gene.8533 | PREDICTED: 60S acidic ribosomal protein P0 [Aethina tumida] | 149 | 3 |
| Gene.8533 | PREDICTED: 60S acidic ribosomal protein P0 [Aethina tumida] | 6 | 3 |
| Gene.8533 | PREDICTED: 60S acidic ribosomal protein P0 [Aethina tumida] | 106 | 3 |
| Gene.8533 | PREDICTED: 60S acidic ribosomal protein P0 [Aethina tumida] | 101 | 3 |
| Gene.8533 | PREDICTED: 60S acidic ribosomal protein P0 [Aethina tumida] | 10 | 3 |
| Gene.8586 | PREDICTED: putative cystathionine gamma-lyase 2 [Aethina tumida] | 68 | 3 |
| Gene.8593 | 26S proteasome regulatory subunit 6A-B [Anoplophora glabripennis] | 32 | 3 |
| Gene.8593 | 26S proteasome regulatory subunit 6A-B [Anoplophora glabripennis] | 252 | 3 |
| Gene.8594 | PREDICTED: 60S ribosomal protein L35a [Tribolium castaneum] | 128 | 3 |
| Gene.8594 | PREDICTED: 60S ribosomal protein L35a [Tribolium castaneum] | 75 | 3 |
| Gene.8594 | PREDICTED: 60S ribosomal protein L35a [Tribolium castaneum] | 38 | 3 |
| Gene.8594 | PREDICTED: 60S ribosomal protein L35a [Tribolium castaneum] | 106 | 3 |
| Gene.8594 | PREDICTED: 60S ribosomal protein L35a [Tribolium castaneum] | 66 | 3 |
| Gene.8594 | PREDICTED: 60S ribosomal protein L35a [Tribolium castaneum] | 45 | 3 |
| Gene.8594 | PREDICTED: 60S ribosomal protein L35a [Tribolium castaneum] | 13 | 3 |
| Gene.8594 | PREDICTED: 60S ribosomal protein L35a [Tribolium castaneum] | 19 | 3 |
| Gene.8594 | PREDICTED: 60S ribosomal protein L35a [Tribolium castaneum] | 82 | 3 |
| Gene.8615 | uncharacterized protein BDFB_013993 [Asbolus verrucosus] | 149 | 3 |
| Gene.8615 | uncharacterized protein BDFB_013993 [Asbolus verrucosus] | 298 | 3 |
| Gene.8615 | uncharacterized protein BDFB_013993 [Asbolus verrucosus] | 326 | 3 |
| Gene.8615 | uncharacterized protein BDFB_013993 [Asbolus verrucosus] | 239 | 3 |
| Gene.8615 | uncharacterized protein BDFB_013993 [Asbolus verrucosus] | 316 | 3 |
| Gene.8615 | uncharacterized protein BDFB_013993 [Asbolus verrucosus] | 327 | 3 |
| Gene.8615 | uncharacterized protein BDFB_013993 [Asbolus verrucosus] | 180 | 3 |
| Gene.8615 | uncharacterized protein BDFB_013993 [Asbolus verrucosus] | 216 | 3 |
| Gene.8615 | uncharacterized protein BDFB_013993 [Asbolus verrucosus] | 223 | 3 |
| Gene.8616 | dihydrolipoyllysine-residue acetyltransferase component of pyruvate dehydrogenase complex, mitochondrial isoform X1 [Anoplophora glabripennis] | 445 | 3 |
| Gene.8616 | dihydrolipoyllysine-residue acetyltransferase component of pyruvate dehydrogenase complex, mitochondrial isoform X1 [Anoplophora glabripennis] | 131 | 3 |
| Gene.8616 | dihydrolipoyllysine-residue acetyltransferase component of pyruvate dehydrogenase complex, mitochondrial isoform X1 [Anoplophora glabripennis] | 474 | 3 |
| Gene.8616 | dihydrolipoyllysine-residue acetyltransferase component of pyruvate dehydrogenase complex, mitochondrial isoform X1 [Anoplophora glabripennis] | 156 | 3 |
| Gene.8616 | dihydrolipoyllysine-residue acetyltransferase component of pyruvate dehydrogenase complex, mitochondrial isoform X1 [Anoplophora glabripennis] | 380 | 3 |
| Gene.8616 | dihydrolipoyllysine-residue acetyltransferase component of pyruvate dehydrogenase complex, mitochondrial isoform X1 [Anoplophora glabripennis] | 388 | 3 |
| Gene.8621 | low molecular weight phosphotyrosine protein phosphatase 1-like [Leptinotarsa decemlineata] | 139 | 3 |
| Gene.8624 | eukaryotic translation initiation factor 3 subunit E [Anoplophora glabripennis] | 81 | 3 |
| Gene.8624 | eukaryotic translation initiation factor 3 subunit E [Anoplophora glabripennis] | 41 | 3 |
| Gene.864 | stress-induced-phosphoprotein 1-like [Asbolus verrucosus] | 166 | 3 |
| Gene.864 | stress-induced-phosphoprotein 1-like [Asbolus verrucosus] | 181 | 3 |
| Gene.864 | stress-induced-phosphoprotein 1-like [Asbolus verrucosus] | 116 | 3 |
| Gene.864 | stress-induced-phosphoprotein 1-like [Asbolus verrucosus] | 56 | 3 |
| Gene.864 | stress-induced-phosphoprotein 1-like [Asbolus verrucosus] | 139 | 3 |
| Gene.864 | stress-induced-phosphoprotein 1-like [Asbolus verrucosus] | 248 | 3 |
| Gene.8646 | transformer-2 protein homolog beta [Tribolium castaneum] | 215 | 3 |
| Gene.865 | calcyphosin-like protein isoform X3 [Helicoverpa armigera] | 154 | 3 |
| Gene.865 | calcyphosin-like protein isoform X3 [Helicoverpa armigera] | 177 | 3 |
| Gene.865 | calcyphosin-like protein isoform X3 [Helicoverpa armigera] | 167 | 3 |
| Gene.8655 | PREDICTED: transcription factor A, mitochondrial [Tribolium castaneum] | 74 | 3 |
| Gene.8655 | PREDICTED: transcription factor A, mitochondrial [Tribolium castaneum] | 203 | 3 |
| Gene.8656 | soluble calcium-activated nucleotidase 1 [Asbolus verrucosus] | 205 | 3 |
| Gene.8656 | soluble calcium-activated nucleotidase 1 [Asbolus verrucosus] | 212 | 3 |
| Gene.8656 | soluble calcium-activated nucleotidase 1 [Asbolus verrucosus] | 361 | 3 |
| Gene.8656 | soluble calcium-activated nucleotidase 1 [Asbolus verrucosus] | 121 | 3 |
| Gene.8672 | carnitine O-palmitoyltransferase 2, mitochondrial-like [Leptinotarsa decemlineata] | 302 | 3 |
| Gene.8673 | succinate dehydrogenase [ubiquinone] iron-sulfur subunit, mitochondrial, partial [Asbolus verrucosus] | 48 | 3 |
| Gene.8678 | PREDICTED: apoptosis inhibitor 5 [Aethina tumida] | 397 | 3 |
| Gene.8704 | PREDICTED: protein D2-like isoform X1 [Aethina tumida] | 128 | 3 |
| Gene.8704 | PREDICTED: protein D2-like isoform X1 [Aethina tumida] | 144 | 3 |
| Gene.8704 | PREDICTED: protein D2-like isoform X1 [Aethina tumida] | 41 | 3 |
| Gene.8707 | PREDICTED: heterogeneous nuclear ribonucleoprotein Q isoform X1 [Tribolium castaneum] | 35 | 3 |
| Gene.8707 | PREDICTED: heterogeneous nuclear ribonucleoprotein Q isoform X1 [Tribolium castaneum] | 365 | 3 |
| Gene.8707 | PREDICTED: heterogeneous nuclear ribonucleoprotein Q isoform X1 [Tribolium castaneum] | 127 | 3 |
| Gene.8707 | PREDICTED: heterogeneous nuclear ribonucleoprotein Q isoform X1 [Tribolium castaneum] | 39 | 3 |
| Gene.8707 | PREDICTED: heterogeneous nuclear ribonucleoprotein Q isoform X1 [Tribolium castaneum] | 20 | 3 |
| Gene.8707 | PREDICTED: heterogeneous nuclear ribonucleoprotein Q isoform X1 [Tribolium castaneum] | 45 | 3 |
| Gene.8707 | PREDICTED: heterogeneous nuclear ribonucleoprotein Q isoform X1 [Tribolium castaneum] | 340 | 3 |
| Gene.8707 | PREDICTED: heterogeneous nuclear ribonucleoprotein Q isoform X1 [Tribolium castaneum] | 355 | 3 |
| Gene.8719 | soma ferritin [Agrilus planipennis] | 109 | 3 |
| Gene.8719 | soma ferritin [Agrilus planipennis] | 189 | 3 |
| Gene.8719 | soma ferritin [Agrilus planipennis] | 106 | 3 |
| Gene.8719 | soma ferritin [Agrilus planipennis] | 97 | 3 |
| Gene.8719 | soma ferritin [Agrilus planipennis] | 115 | 3 |
| Gene.8719 | soma ferritin [Agrilus planipennis] | 153 | 3 |
| Gene.872 | mesencephalic astrocyte-derived neurotrophic factor homolog [Leptinotarsa decemlineata] | 35 | 3 |
| Gene.8727 | PREDICTED: proline dehydrogenase 1, mitochondrial isoform X2 [Dendroctonus ponderosae] | 296 | 3 |
| Gene.8727 | PREDICTED: proline dehydrogenase 1, mitochondrial isoform X2 [Dendroctonus ponderosae] | 343 | 3 |
| Gene.8730 | PREDICTED: D-arabinitol dehydrogenase 1 [Tribolium castaneum] | 286 | 3 |
| Gene.8730 | PREDICTED: D-arabinitol dehydrogenase 1 [Tribolium castaneum] | 328 | 3 |
| Gene.8730 | PREDICTED: D-arabinitol dehydrogenase 1 [Tribolium castaneum] | 344 | 3 |
| Gene.8730 | PREDICTED: D-arabinitol dehydrogenase 1 [Tribolium castaneum] | 97 | 3 |
| Gene.8730 | PREDICTED: D-arabinitol dehydrogenase 1 [Tribolium castaneum] | 335 | 3 |
| Gene.8730 | PREDICTED: D-arabinitol dehydrogenase 1 [Tribolium castaneum] | 338 | 3 |
| Gene.874 | PREDICTED: uncharacterized protein LOC655336 isoform X1 [Tribolium castaneum] | 108 | 3 |
| Gene.8744 | elongation factor 1-beta'-like [Onthophagus taurus] | 149 | 3 |
| Gene.8744 | elongation factor 1-beta'-like [Onthophagus taurus] | 32 | 3 |
| Gene.8744 | elongation factor 1-beta'-like [Onthophagus taurus] | 39 | 3 |
| Gene.8744 | elongation factor 1-beta'-like [Onthophagus taurus] | 83 | 3 |
| Gene.8744 | elongation factor 1-beta'-like [Onthophagus taurus] | 7 | 3 |
| Gene.8744 | elongation factor 1-beta'-like [Onthophagus taurus] | 153 | 3 |
| Gene.8744 | elongation factor 1-beta'-like [Onthophagus taurus] | 22 | 3 |
| Gene.8744 | elongation factor 1-beta'-like [Onthophagus taurus] | 60 | 3 |
| Gene.8744 | elongation factor 1-beta'-like [Onthophagus taurus] | 43 | 3 |
| Gene.8744 | elongation factor 1-beta'-like [Onthophagus taurus] | 140 | 3 |
| Gene.8745 | uncharacterized protein LOC108915763 [Anoplophora glabripennis] | 46 | 3 |
| Gene.8745 | uncharacterized protein LOC108915763 [Anoplophora glabripennis] | 51 | 3 |
| Gene.8751 | PREDICTED: keratin, type I cytoskeletal 9 [Tribolium castaneum] | 251 | 3 |
| Gene.8756 | uncharacterized protein Dvir_GJ16722 [Drosophila virilis] | 84 | 3 |
| Gene.8756 | uncharacterized protein Dvir_GJ16722 [Drosophila virilis] | 10 | 3 |
| Gene.8758 | PREDICTED: la protein homolog [Tribolium castaneum] | 197 | 3 |
| Gene.8758 | PREDICTED: la protein homolog [Tribolium castaneum] | 204 | 3 |
| Gene.8758 | PREDICTED: la protein homolog [Tribolium castaneum] | 313 | 3 |
| Gene.8758 | PREDICTED: la protein homolog [Tribolium castaneum] | 180 | 3 |
| Gene.8758 | PREDICTED: la protein homolog [Tribolium castaneum] | 317 | 3 |
| Gene.8758 | PREDICTED: la protein homolog [Tribolium castaneum] | 232 | 3 |
| Gene.8758 | PREDICTED: la protein homolog [Tribolium castaneum] | 224 | 3 |
| Gene.8768 | PREDICTED: flavin reductase (NADPH) [Tribolium castaneum] | 92 | 3 |
| Gene.8768 | PREDICTED: flavin reductase (NADPH) [Tribolium castaneum] | 87 | 3 |
| Gene.8768 | PREDICTED: flavin reductase (NADPH) [Tribolium castaneum] | 46 | 3 |
| Gene.8771 | PREDICTED: NADH dehydrogenase [ubiquinone] iron-sulfur protein 6, mitochondrial [Tribolium castaneum] | 54 | 3 |
| Gene.8787 | UDPGT domain containing protein [Asbolus verrucosus] | 296 | 3 |
| Gene.8787 | UDPGT domain containing protein [Asbolus verrucosus] | 442 | 3 |
| Gene.8787 | UDPGT domain containing protein [Asbolus verrucosus] | 208 | 3 |
| Gene.8801 | hypothetical protein WN51_04145 [Melipona quadrifasciata] | 123 | 3 |
| Gene.8801 | hypothetical protein WN51_04145 [Melipona quadrifasciata] | 92 | 3 |
| Gene.8801 | hypothetical protein WN51_04145 [Melipona quadrifasciata] | 249 | 3 |
| Gene.8801 | hypothetical protein WN51_04145 [Melipona quadrifasciata] | 336 | 3 |
| Gene.8815 | Uncharacterized protein | 147 | 3 |
| Gene.8815 | Uncharacterized protein | 128 | 3 |
| Gene.8815 | Uncharacterized protein | 155 | 3 |
| Gene.8815 | Uncharacterized protein | 90 | 3 |
| Gene.8815 | Uncharacterized protein | 70 | 3 |
| Gene.8815 | Uncharacterized protein | 183 | 3 |
| Gene.8815 | Uncharacterized protein | 122 | 3 |
| Gene.8815 | Uncharacterized protein | 144 | 3 |
| Gene.8815 | Uncharacterized protein | 133 | 3 |
| Gene.8815 | Uncharacterized protein | 96 | 3 |
| Gene.8819 | hydroxyacyl-coenzyme A dehydrogenase, mitochondrial [Anoplophora glabripennis] | 124 | 3 |
| Gene.8819 | hydroxyacyl-coenzyme A dehydrogenase, mitochondrial [Anoplophora glabripennis] | 272 | 3 |
| Gene.8819 | hydroxyacyl-coenzyme A dehydrogenase, mitochondrial [Anoplophora glabripennis] | 64 | 3 |
| Gene.8819 | hydroxyacyl-coenzyme A dehydrogenase, mitochondrial [Anoplophora glabripennis] | 75 | 3 |
| Gene.8819 | hydroxyacyl-coenzyme A dehydrogenase, mitochondrial [Anoplophora glabripennis] | 280 | 3 |
| Gene.8819 | hydroxyacyl-coenzyme A dehydrogenase, mitochondrial [Anoplophora glabripennis] | 234 | 3 |
| Gene.8819 | hydroxyacyl-coenzyme A dehydrogenase, mitochondrial [Anoplophora glabripennis] | 80 | 3 |
| Gene.8819 | hydroxyacyl-coenzyme A dehydrogenase, mitochondrial [Anoplophora glabripennis] | 119 | 3 |
| Gene.8819 | hydroxyacyl-coenzyme A dehydrogenase, mitochondrial [Anoplophora glabripennis] | 93 | 3 |
| Gene.8819 | hydroxyacyl-coenzyme A dehydrogenase, mitochondrial [Anoplophora glabripennis] | 293 | 3 |
| Gene.8825 | T-complex protein 1 subunit theta [Anoplophora glabripennis] | 161 | 3 |
| Gene.8825 | T-complex protein 1 subunit theta [Anoplophora glabripennis] | 233 | 3 |
| Gene.8825 | T-complex protein 1 subunit theta [Anoplophora glabripennis] | 440 | 3 |
| Gene.8825 | T-complex protein 1 subunit theta [Anoplophora glabripennis] | 318 | 3 |
| Gene.8825 | T-complex protein 1 subunit theta [Anoplophora glabripennis] | 7 | 3 |
| Gene.8825 | T-complex protein 1 subunit theta [Anoplophora glabripennis] | 400 | 3 |
| Gene.8830 | PREDICTED: probable 26S proteasome non-ATPase regulatory subunit 3 [Tribolium castaneum] | 461 | 3 |
| Gene.8844 | ubiquitin carboxyl-terminal hydrolase 5 [Anoplophora glabripennis] | 306 | 3 |
| Gene.8848 | PREDICTED: fumarylacetoacetase [Aethina tumida] | 384 | 3 |
| Gene.8853 | PREDICTED: selenide, water dikinase [Tribolium castaneum] | 54 | 3 |
| Gene.8854 | clavesin-1 isoform X2 [Anoplophora glabripennis] | 200 | 3 |
| Gene.8854 | clavesin-1 isoform X2 [Anoplophora glabripennis] | 53 | 3 |
| Gene.8856 | eukaryotic peptide chain release factor GTP-binding subunit ERF3A [Asbolus verrucosus] | 566 | 3 |
| Gene.8862 | protein UBASH3A homolog isoform X2 [Onthophagus taurus] | 244 | 3 |
| Gene.8862 | protein UBASH3A homolog isoform X2 [Onthophagus taurus] | 114 | 3 |
| Gene.8862 | protein UBASH3A homolog isoform X2 [Onthophagus taurus] | 249 | 3 |
| Gene.8866 | hypothetical protein TcasGA2_TC014619 [Tribolium castaneum] | 42 | 3 |
| Gene.8866 | hypothetical protein TcasGA2_TC014619 [Tribolium castaneum] | 60 | 3 |
| Gene.8866 | hypothetical protein TcasGA2_TC014619 [Tribolium castaneum] | 133 | 3 |
| Gene.8868 | UBX domain-containing protein 1 [Anoplophora glabripennis] | 128 | 3 |
| Gene.8868 | UBX domain-containing protein 1 [Anoplophora glabripennis] | 151 | 3 |
| Gene.887 | 60S ribosomal protein L44 [Solenopsis invicta] | 42 | 3 |
| Gene.887 | 60S ribosomal protein L44 [Solenopsis invicta] | 73 | 3 |
| Gene.8873 | PREDICTED: fumarate hydratase, mitochondrial [Tribolium castaneum] | 99 | 3 |
| Gene.8873 | PREDICTED: fumarate hydratase, mitochondrial [Tribolium castaneum] | 57 | 3 |
| Gene.8873 | PREDICTED: fumarate hydratase, mitochondrial [Tribolium castaneum] | 106 | 3 |
| Gene.8873 | PREDICTED: fumarate hydratase, mitochondrial [Tribolium castaneum] | 45 | 3 |
| Gene.8873 | PREDICTED: fumarate hydratase, mitochondrial [Tribolium castaneum] | 122 | 3 |
| Gene.8873 | PREDICTED: fumarate hydratase, mitochondrial [Tribolium castaneum] | 218 | 3 |
| Gene.8903 | PREDICTED: T-complex protein 1 subunit epsilon [Tribolium castaneum] | 147 | 3 |
| Gene.8903 | PREDICTED: T-complex protein 1 subunit epsilon [Tribolium castaneum] | 215 | 3 |
| Gene.8903 | PREDICTED: T-complex protein 1 subunit epsilon [Tribolium castaneum] | 203 | 3 |
| Gene.8903 | PREDICTED: T-complex protein 1 subunit epsilon [Tribolium castaneum] | 353 | 3 |
| Gene.8903 | PREDICTED: T-complex protein 1 subunit epsilon [Tribolium castaneum] | 276 | 3 |
| Gene.8912 | PREDICTED: 3-oxoacyl-[acyl-carrier-protein] reductase FabG [Tribolium castaneum] | 79 | 3 |
| Gene.8917 | PREDICTED: ester hydrolase C11orf54 homolog isoform X1 [Tribolium castaneum] | 83 | 3 |
| Gene.8917 | PREDICTED: ester hydrolase C11orf54 homolog isoform X1 [Tribolium castaneum] | 13 | 3 |
| Gene.8917 | PREDICTED: ester hydrolase C11orf54 homolog isoform X1 [Tribolium castaneum] | 78 | 3 |
| Gene.8928 | 60S ribosomal protein L15 [Leptinotarsa decemlineata] | 147 | 3 |
| Gene.8928 | 60S ribosomal protein L15 [Leptinotarsa decemlineata] | 176 | 3 |
| Gene.8928 | 60S ribosomal protein L15 [Leptinotarsa decemlineata] | 5 | 3 |
| Gene.8938 | 14-3-3 epsilon [Tenebrio molitor] | 10 | 3 |
| Gene.8938 | 14-3-3 epsilon [Tenebrio molitor] | 78 | 3 |
| Gene.8938 | 14-3-3 epsilon [Tenebrio molitor] | 12 | 3 |
| Gene.8946 | probable phosphatase phospho2 isoform X1 [Anoplophora glabripennis] | 216 | 3 |
| Gene.8952 | threonine--tRNA ligase, cytoplasmic isoform X1 [Anoplophora glabripennis] | 229 | 3 |
| Gene.8952 | threonine--tRNA ligase, cytoplasmic isoform X1 [Anoplophora glabripennis] | 299 | 3 |
| Gene.8963 | 14-3-3 protein zeta isoform X2 [Anoplophora glabripennis] | 6 | 3 |
| Gene.8963 | 14-3-3 protein zeta isoform X2 [Anoplophora glabripennis] | 123 | 3 |
| Gene.8963 | 14-3-3 protein zeta isoform X2 [Anoplophora glabripennis] | 71 | 3 |
| Gene.8963 | 14-3-3 protein zeta isoform X2 [Anoplophora glabripennis] | 83 | 3 |
| Gene.8963 | 14-3-3 protein zeta isoform X2 [Anoplophora glabripennis] | 118 | 3 |
| Gene.8963 | 14-3-3 protein zeta isoform X2 [Anoplophora glabripennis] | 111 | 3 |
| Gene.8963 | 14-3-3 protein zeta isoform X2 [Anoplophora glabripennis] | 160 | 3 |
| Gene.8963 | 14-3-3 protein zeta isoform X2 [Anoplophora glabripennis] | 14 | 3 |
| Gene.8963 | 14-3-3 protein zeta isoform X2 [Anoplophora glabripennis] | 52 | 3 |
| Gene.8963 | 14-3-3 protein zeta isoform X2 [Anoplophora glabripennis] | 30 | 3 |
| Gene.8963 | 14-3-3 protein zeta isoform X2 [Anoplophora glabripennis] | 125 | 3 |
| Gene.8967 | PREDICTED: ubiquitin-conjugating enzyme E2 variant 1 [Aethina tumida] | 83 | 3 |
| Gene.8971 | conserved hypothetical protein [Culex quinquefasciatus] | 36 | 3 |
| Gene.8976 | PREDICTED: putative esterase isoform X1 [Tribolium castaneum] | 263 | 3 |
| Gene.8978 | tRNA-specific adenosine deaminase 1-like Protein [Tribolium castaneum] | 69 | 3 |
| Gene.8982 | endocuticle structural glycoprotein SgAbd-8-like [Asbolus verrucosus] | 87 | 3 |
| Gene.8983 | PREDICTED: endocuticle structural glycoprotein SgAbd-4 [Tribolium castaneum] | 52 | 3 |
| Gene.8983 | PREDICTED: endocuticle structural glycoprotein SgAbd-4 [Tribolium castaneum] | 60 | 3 |
| Gene.8989 | PREDICTED: aldehyde dehydrogenase, dimeric NADP-preferring-like [Aethina tumida] | 179 | 3 |
| Gene.8989 | PREDICTED: aldehyde dehydrogenase, dimeric NADP-preferring-like [Aethina tumida] | 438 | 3 |
| Gene.8989 | PREDICTED: aldehyde dehydrogenase, dimeric NADP-preferring-like [Aethina tumida] | 258 | 3 |
| Gene.8992 | sideroflexin-2 [Anoplophora glabripennis] | 38 | 3 |
| Gene.8992 | sideroflexin-2 [Anoplophora glabripennis] | 22 | 3 |
| Gene.8992 | sideroflexin-2 [Anoplophora glabripennis] | 305 | 3 |
| Gene.8992 | sideroflexin-2 [Anoplophora glabripennis] | 322 | 3 |
| Gene.8992 | sideroflexin-2 [Anoplophora glabripennis] | 44 | 3 |
| Gene.8992 | sideroflexin-2 [Anoplophora glabripennis] | 82 | 3 |
| Gene.8995 | PREDICTED: 60S ribosomal protein L9 [Megachile rotundata] | 119 | 3 |
| Gene.8995 | PREDICTED: 60S ribosomal protein L9 [Megachile rotundata] | 172 | 3 |
| Gene.8995 | PREDICTED: 60S ribosomal protein L9 [Megachile rotundata] | 39 | 3 |
| Gene.8995 | PREDICTED: 60S ribosomal protein L9 [Megachile rotundata] | 57 | 3 |
| Gene.8995 | PREDICTED: 60S ribosomal protein L9 [Megachile rotundata] | 125 | 3 |
| Gene.8995 | PREDICTED: 60S ribosomal protein L9 [Megachile rotundata] | 89 | 3 |
| Gene.8998 | RecName: Full=Alpha-amylase; AltName: Full=1,4-alpha-D-glucan glucanohydrolase | 322 | 3 |
| Gene.8998 | RecName: Full=Alpha-amylase; AltName: Full=1,4-alpha-D-glucan glucanohydrolase | 226 | 3 |
| Gene.901 | PREDICTED: plasma membrane calcium-transporting ATPase 2 isoform X2 [Tribolium castaneum] | 772 | 3 |
| Gene.901 | PREDICTED: plasma membrane calcium-transporting ATPase 2 isoform X2 [Tribolium castaneum] | 166 | 3 |
| Gene.901 | PREDICTED: plasma membrane calcium-transporting ATPase 2 isoform X2 [Tribolium castaneum] | 16 | 3 |
| Gene.9010 | PREDICTED: TATA-binding protein-associated factor 2N-like isoform X2 [Aethina tumida] | 76 | 3 |
| Gene.9033 | 60S ribosomal protein L7 [Anoplophora glabripennis] | 154 | 3 |
| Gene.9033 | 60S ribosomal protein L7 [Anoplophora glabripennis] | 151 | 3 |
| Gene.9033 | 60S ribosomal protein L7 [Anoplophora glabripennis] | 41 | 3 |
| Gene.9033 | 60S ribosomal protein L7 [Anoplophora glabripennis] | 62 | 3 |
| Gene.9040 | PREDICTED: microsomal glutathione S-transferase 1 [Tribolium castaneum] | 57 | 3 |
| Gene.9040 | PREDICTED: microsomal glutathione S-transferase 1 [Tribolium castaneum] | 55 | 3 |
| Gene.9046 | hsp90 co-chaperone Cdc37 [Anoplophora glabripennis] | 97 | 3 |
| Gene.9057 | PREDICTED: pyrroline-5-carboxylate reductase [Tribolium castaneum] | 151 | 3 |
| Gene.9057 | PREDICTED: pyrroline-5-carboxylate reductase [Tribolium castaneum] | 144 | 3 |
| Gene.9057 | PREDICTED: pyrroline-5-carboxylate reductase [Tribolium castaneum] | 232 | 3 |
| Gene.9064 | mitochondrial amidoxime-reducing component 1-like [Anoplophora glabripennis] | 159 | 3 |
| Gene.9064 | mitochondrial amidoxime-reducing component 1-like [Anoplophora glabripennis] | 205 | 3 |
| Gene.9064 | mitochondrial amidoxime-reducing component 1-like [Anoplophora glabripennis] | 53 | 3 |
| Gene.9071 | nucleolar protein 56 [Leptinotarsa decemlineata] | 194 | 3 |
| Gene.9071 | nucleolar protein 56 [Leptinotarsa decemlineata] | 19 | 3 |
| Gene.919 | ribosomal protein S6 [Chrysomela tremula] | 159 | 3 |
| Gene.919 | ribosomal protein S6 [Chrysomela tremula] | 149 | 3 |
| Gene.919 | ribosomal protein S6 [Chrysomela tremula] | 165 | 3 |
| Gene.919 | ribosomal protein S6 [Chrysomela tremula] | 51 | 3 |
| Gene.919 | ribosomal protein S6 [Chrysomela tremula] | 23 | 3 |
| Gene.919 | ribosomal protein S6 [Chrysomela tremula] | 58 | 3 |
| Gene.919 | ribosomal protein S6 [Chrysomela tremula] | 14 | 3 |
| Gene.919 | ribosomal protein S6 [Chrysomela tremula] | 211 | 3 |
| Gene.922 | acetyl-CoA acetyltransferase, mitochondrial, partial [Asbolus verrucosus] | 243 | 3 |
| Gene.922 | acetyl-CoA acetyltransferase, mitochondrial, partial [Asbolus verrucosus] | 252 | 3 |
| Gene.922 | acetyl-CoA acetyltransferase, mitochondrial, partial [Asbolus verrucosus] | 214 | 3 |
| Gene.922 | acetyl-CoA acetyltransferase, mitochondrial, partial [Asbolus verrucosus] | 249 | 3 |
| Gene.922 | acetyl-CoA acetyltransferase, mitochondrial, partial [Asbolus verrucosus] | 293 | 3 |
| Gene.924 | 26S proteasome non-ATPase regulatory subunit 11 [Asbolus verrucosus] | 274 | 3 |
| Gene.924 | 26S proteasome non-ATPase regulatory subunit 11 [Asbolus verrucosus] | 344 | 3 |
| Gene.924 | 26S proteasome non-ATPase regulatory subunit 11 [Asbolus verrucosus] | 355 | 3 |
| Gene.924 | 26S proteasome non-ATPase regulatory subunit 11 [Asbolus verrucosus] | 20 | 3 |
| Gene.924 | 26S proteasome non-ATPase regulatory subunit 11 [Asbolus verrucosus] | 110 | 3 |
| Gene.931 | PREDICTED: glutamine--fructose-6-phosphate aminotransferase [isomerizing] 2 isoform X1 [Tribolium castaneum] | 184 | 3 |
| Gene.95 | Uncharacterized protein | 84 | 3 |
| Gene.950 | heat shock protein 75 kDa, mitochondrial, partial [Asbolus verrucosus] | 115 | 3 |
| Gene.955 | PREDICTED: presequence protease, mitochondrial [Tribolium castaneum] | 363 | 3 |
| Gene.96 | Uncharacterized protein | 35 | 3 |
| Gene.960 | probable phosphoserine aminotransferase [Anoplophora glabripennis] | 51 | 3 |
| Gene.963 | uncharacterized protein BDFB_008975, partial [Asbolus verrucosus] | 537 | 3 |
| Gene.97 | far upstream element-binding protein 1 isoform X1 [Anoplophora glabripennis] | 261 | 3 |
| Gene.97 | far upstream element-binding protein 1 isoform X1 [Anoplophora glabripennis] | 368 | 3 |
| Gene.97 | far upstream element-binding protein 1 isoform X1 [Anoplophora glabripennis] | 270 | 3 |
| Gene.970 | short-chain specific acyl-CoA dehydrogenase, mitochondrial-like [Leptinotarsa decemlineata] | 56 | 3 |
| Gene.970 | short-chain specific acyl-CoA dehydrogenase, mitochondrial-like [Leptinotarsa decemlineata] | 193 | 3 |
| Gene.986 | PREDICTED: aldehyde dehydrogenase, dimeric NADP-preferring [Tribolium castaneum] | 265 | 3 |
| Gene.986 | PREDICTED: aldehyde dehydrogenase, dimeric NADP-preferring [Tribolium castaneum] | 452 | 3 |
| Gene.986 | PREDICTED: aldehyde dehydrogenase, dimeric NADP-preferring [Tribolium castaneum] | 19 | 3 |
| Gene.992 | Uncharacterized protein | 120 | 3 |
| Gene.993 | puromycin-sensitive aminopeptidase-like protein isoform X1 [Anoplophora glabripennis] | 249 | 3 |
| Gene.994 | PREDICTED: myelin expression factor 2 [Tribolium castaneum] | 513 | 3 |
| Gene.996 | PREDICTED: myelin expression factor 2 [Tribolium castaneum] | 456 | 3 |
| Gene.996 | PREDICTED: myelin expression factor 2 [Tribolium castaneum] | 492 | 3 |
| Gene.996 | PREDICTED: myelin expression factor 2 [Tribolium castaneum] | 227 | 3 |
| Gene.10 | PREDICTED: flotillin-1 isoform X2 [Aethina tumida] | 421 | 2 |
| Gene.1024 | annexin B9 isoform X2 [Anoplophora glabripennis] | 279 | 2 |
| Gene.1024 | annexin B9 isoform X2 [Anoplophora glabripennis] | 180 | 2 |
| Gene.1024 | annexin B9 isoform X2 [Anoplophora glabripennis] | 338 | 2 |
| Gene.106 | Uncharacterized protein | 235 | 2 |
| Gene.1067 | Uncharacterized protein | 196 | 2 |
| Gene.1073 | PREDICTED: succinate dehydrogenase [ubiquinone] flavoprotein subunit, mitochondrial isoform X1 [Tribolium castaneum] | 140 | 2 |
| Gene.1126 | PREDICTED: puromycin-sensitive aminopeptidase [Nicrophorus vespilloides] | 465 | 2 |
| Gene.1126 | PREDICTED: puromycin-sensitive aminopeptidase [Nicrophorus vespilloides] | 573 | 2 |
| Gene.1158 | PREDICTED: uncharacterized protein LOC109537982 isoform X2 [Dendroctonus ponderosae] | 332 | 2 |
| Gene.1161 | PREDICTED: UTP--glucose-1-phosphate uridylyltransferase isoform X1 [Tribolium castaneum] | 432 | 2 |
| Gene.1251 | thymidylate kinase isoform X2 [Anoplophora glabripennis] | 28 | 2 |
| Gene.1253 | PREDICTED: sedoheptulokinase isoform X2 [Tribolium castaneum] | 350 | 2 |
| Gene.1253 | PREDICTED: sedoheptulokinase isoform X2 [Tribolium castaneum] | 443 | 2 |
| Gene.1253 | PREDICTED: sedoheptulokinase isoform X2 [Tribolium castaneum] | 49 | 2 |
| Gene.1253 | PREDICTED: sedoheptulokinase isoform X2 [Tribolium castaneum] | 423 | 2 |
| Gene.1264 | PREDICTED: protein disulfide-isomerase [Tribolium castaneum] | 167 | 2 |
| Gene.1264 | PREDICTED: protein disulfide-isomerase [Tribolium castaneum] | 95 | 2 |
| Gene.1295 | PREDICTED: lamin Dm0 isoform X1 [Aethina tumida] | 211 | 2 |
| Gene.1295 | PREDICTED: lamin Dm0 isoform X1 [Aethina tumida] | 151 | 2 |
| Gene.1297 | PREDICTED: S-phase kinase-associated protein 1 [Dendroctonus ponderosae] | 65 | 2 |
| Gene.130 | PREDICTED: T-complex protein 1 subunit beta [Tribolium castaneum] | 153 | 2 |
| Gene.1357 | Uncharacterized protein | 52 | 2 |
| Gene.1361 | PREDICTED: glycogenin-1 isoform X3 [Aethina tumida] | 229 | 2 |
| Gene.1392 | hypothetical protein WN55_01867 [Dufourea novaeangliae] | 194 | 2 |
| Gene.1398 | PREDICTED: multifunctional protein ADE2 [Aethina tumida] | 30 | 2 |
| Gene.1426 | glycine--tRNA ligase [Anoplophora glabripennis] | 103 | 2 |
| Gene.1468 | PREDICTED: dolichyl-diphosphooligosaccharide--protein glycosyltransferase subunit STT3B [Aethina tumida] | 638 | 2 |
| Gene.1476 | zinc finger protein on ecdysone puffs, partial [Asbolus verrucosus] | 294 | 2 |
| Gene.1476 | zinc finger protein on ecdysone puffs, partial [Asbolus verrucosus] | 462 | 2 |
| Gene.1482 | flotillin-2 [Onthophagus taurus] | 304 | 2 |
| Gene.1489 | aspartate aminotransferase, mitochondrial [Asbolus verrucosus] | 182 | 2 |
| Gene.1489 | aspartate aminotransferase, mitochondrial [Asbolus verrucosus] | 167 | 2 |
| Gene.1489 | aspartate aminotransferase, mitochondrial [Asbolus verrucosus] | 177 | 2 |
| Gene.1524 | V-type proton ATPase subunit B [Galleria mellonella] | 198 | 2 |
| Gene.1528 | PREDICTED: protein SCO1 homolog, mitochondrial [Aethina tumida] | 70 | 2 |
| Gene.1532 | PREDICTED: glyoxalase domain-containing protein 4 [Tribolium castaneum] | 106 | 2 |
| Gene.1533 | PREDICTED: hexokinase type 2 isoform X2 [Tribolium castaneum] | 134 | 2 |
| Gene.154 | PREDICTED: 26S protease regulatory subunit 7 [Tribolium castaneum] | 118 | 2 |
| Gene.154 | PREDICTED: 26S protease regulatory subunit 7 [Tribolium castaneum] | 409 | 2 |
| Gene.1580 | PREDICTED: aminomethyltransferase, mitochondrial [Tribolium castaneum] | 165 | 2 |
| Gene.1588 | cullin-associated NEDD8-dissociated protein 1 [Anoplophora glabripennis] | 582 | 2 |
| Gene.1653 | PREDICTED: hydroxyacid oxidase 1 [Tribolium castaneum] | 300 | 2 |
| Gene.170 | RNA-binding protein lark [Asbolus verrucosus] | 184 | 2 |
| Gene.1711 | Uncharacterized protein | 37 | 2 |
| Gene.1725 | neprilysin-2-like [Leptinotarsa decemlineata] | 113 | 2 |
| Gene.1725 | neprilysin-2-like [Leptinotarsa decemlineata] | 430 | 2 |
| Gene.1740 | LOW QUALITY PROTEIN: eukaryotic translation initiation factor 4E transporter [Anoplophora glabripennis] | 342 | 2 |
| Gene.175 | PREDICTED: probable citrate synthase 2, mitochondrial [Tribolium castaneum] | 45 | 2 |
| Gene.1768 | PREDICTED: translation elongation factor 2 [Aethina tumida] | 240 | 2 |
| Gene.1768 | PREDICTED: translation elongation factor 2 [Aethina tumida] | 498 | 2 |
| Gene.1789 | thioredoxin domain-containing protein 5 [Asbolus verrucosus] | 115 | 2 |
| Gene.179 | dipeptidyl peptidase 3 [Anoplophora glabripennis] | 202 | 2 |
| Gene.1814 | PREDICTED: probable 39S ribosomal protein L45, mitochondrial [Nicrophorus vespilloides] | 163 | 2 |
| Gene.1846 | ATP synthase subunit beta, mitochondrial [Tribolium castaneum] | 493 | 2 |
| Gene.1862 | PREDICTED: trifunctional enzyme subunit beta, mitochondrial [Tribolium castaneum] | 213 | 2 |
| Gene.1862 | PREDICTED: trifunctional enzyme subunit beta, mitochondrial [Tribolium castaneum] | 413 | 2 |
| Gene.1871 | endoribonuclease Dicer [Anoplophora glabripennis] | 163 | 2 |
| Gene.1878 | MA3 domain containing protein [Asbolus verrucosus] | 261 | 2 |
| Gene.1892 | PREDICTED: enolase [Musca domestica] | 60 | 2 |
| Gene.1895 | heat shock protein 70 [Harmonia axyridis] | 464 | 2 |
| Gene.1895 | heat shock protein 70 [Harmonia axyridis] | 16 | 2 |
| Gene.1899 | 40S ribosomal protein S18 [Agrilus planipennis] | 159 | 2 |
| Gene.1917 | PREDICTED: calnexin [Tribolium castaneum] | 453 | 2 |
| Gene.1917 | PREDICTED: calnexin [Tribolium castaneum] | 383 | 2 |
| Gene.1921 | obg-like ATPase 1 [Anoplophora glabripennis] | 355 | 2 |
| Gene.1932 | PREDICTED: 2-oxoglutarate dehydrogenase, mitochondrial isoform X2 [Tribolium castaneum] | 913 | 2 |
| Gene.1932 | PREDICTED: 2-oxoglutarate dehydrogenase, mitochondrial isoform X2 [Tribolium castaneum] | 907 | 2 |
| Gene.195 | uncharacterized protein LOC108733652 [Agrilus planipennis] | 33 | 2 |
| Gene.195 | uncharacterized protein LOC108733652 [Agrilus planipennis] | 214 | 2 |
| Gene.1958 | PREDICTED: fimbrin isoform X1 [Tribolium castaneum] | 66 | 2 |
| Gene.196 | PREDICTED: triosephosphate isomerase isoform X1 [Aethina tumida] | 88 | 2 |
| Gene.1982 | Tetratricopeptide repeat-containing protein, partial [Oryctes borbonicus] | 338 | 2 |
| Gene.1990 | heat shock protein 10.87 [Harmonia axyridis] | 39 | 2 |
| Gene.1990 | heat shock protein 10.87 [Harmonia axyridis] | 65 | 2 |
| Gene.2014 | hypothetical protein AMK59_1391, partial [Oryctes borbonicus] | 376 | 2 |
| Gene.2016 | PREDICTED: 40S ribosomal protein S4 isoform X1 [Aethina tumida] | 233 | 2 |
| Gene.2017 | ATP synthase subunit O, mitochondrial [Asbolus verrucosus] | 87 | 2 |
| Gene.2037 | ATPase family AAA domain-containing protein 3A homolog [Leptinotarsa decemlineata] | 378 | 2 |
| Gene.210 | elongation factor 1 alpha, partial [Harmonia axyridis] | 36 | 2 |
| Gene.210 | elongation factor 1 alpha, partial [Harmonia axyridis] | 453 | 2 |
| Gene.2113 | 60S ribosomal protein L31 [Blattella germanica] | 67 | 2 |
| Gene.2113 | 60S ribosomal protein L31 [Blattella germanica] | 36 | 2 |
| Gene.2124 | PREDICTED: sorbitol dehydrogenase [Tribolium castaneum] | 60 | 2 |
| Gene.2140 | PREDICTED: UDP-glucuronosyltransferase 2B10-like [Tribolium castaneum] | 321 | 2 |
| Gene.2144 | PREDICTED: succinyl-CoA ligase subunit alpha, mitochondrial [Tribolium castaneum] | 65 | 2 |
| Gene.2164 | pre-mRNA-processing factor 19 [Onthophagus taurus] | 128 | 2 |
| Gene.2167 | PREDICTED: 15-hydroxyprostaglandin dehydrogenase [NAD(+)] [Tribolium castaneum] | 32 | 2 |
| Gene.2176 | UDP-glucuronosyltransferase 1-9-like isoform X2 [Leptinotarsa decemlineata] | 67 | 2 |
| Gene.2192 | small glutamine-rich tetratricopeptide repeat-containing protein alpha-like [Pseudomyrmex gracilis] | 128 | 2 |
| Gene.2197 | PREDICTED: peroxisomal multifunctional enzyme type 2 isoform X1 [Tribolium castaneum] | 34 | 2 |
| Gene.2227 | PREDICTED: ubiquitin-like modifier-activating enzyme 1 [Tribolium castaneum] | 865 | 2 |
| Gene.2236 | Glutamate dehydrogenase, mitochondrial-like Protein [Tribolium castaneum] | 524 | 2 |
| Gene.2236 | Glutamate dehydrogenase, mitochondrial-like Protein [Tribolium castaneum] | 75 | 2 |
| Gene.2236 | Glutamate dehydrogenase, mitochondrial-like Protein [Tribolium castaneum] | 392 | 2 |
| Gene.2263 | probable isocitrate dehydrogenase [NAD] subunit alpha, mitochondrial isoform X1 [Anoplophora glabripennis] | 348 | 2 |
| Gene.2283 | Hemocyanin C domain containing protein [Asbolus verrucosus] | 73 | 2 |
| Gene.229 | PREDICTED: probable phospholipid hydroperoxide glutathione peroxidase [Tribolium castaneum] | 208 | 2 |
| Gene.229 | PREDICTED: probable phospholipid hydroperoxide glutathione peroxidase [Tribolium castaneum] | 121 | 2 |
| Gene.2318 | PREDICTED: LOW QUALITY PROTEIN: V-type proton ATPase 116 kDa subunit a-like [Aethina tumida] | 74 | 2 |
| Gene.2318 | PREDICTED: LOW QUALITY PROTEIN: V-type proton ATPase 116 kDa subunit a-like [Aethina tumida] | 711 | 2 |
| Gene.2362 | uncharacterized protein LOC108904898 isoform X1 [Anoplophora glabripennis] | 98 | 2 |
| Gene.2362 | uncharacterized protein LOC108904898 isoform X1 [Anoplophora glabripennis] | 87 | 2 |
| Gene.2363 | 26S proteasome non-ATPase regulatory subunit 2 [Anoplophora glabripennis] | 326 | 2 |
| Gene.237 | PREDICTED: uncharacterized protein LOC661814 [Tribolium castaneum] | 73 | 2 |
| Gene.2372 | cytochrome P450 CYP9Z401 [Cryptolaemus montrouzieri] | 60 | 2 |
| Gene.2372 | cytochrome P450 CYP9Z401 [Cryptolaemus montrouzieri] | 55 | 2 |
| Gene.2375 | glyceraldehyde-3-phosphate, partial [Harmonia axyridis] | 74 | 2 |
| Gene.2375 | glyceraldehyde-3-phosphate, partial [Harmonia axyridis] | 108 | 2 |
| Gene.2375 | glyceraldehyde-3-phosphate, partial [Harmonia axyridis] | 168 | 2 |
| Gene.2412 | PREDICTED: talin-1 isoform X2 [Tribolium castaneum] | 431 | 2 |
| Gene.2466 | PREDICTED: LOW QUALITY PROTEIN: extended synaptotagmin-2-like [Aethina tumida] | 535 | 2 |
| Gene.2517 | effete, isoform A [Drosophila melanogaster] | 8 | 2 |
| Gene.2519 | importin-7 [Anoplophora glabripennis] | 509 | 2 |
| Gene.2562 | hypothetical protein AMK59_6989 [Oryctes borbonicus] | 125 | 2 |
| Gene.2562 | hypothetical protein AMK59_6989 [Oryctes borbonicus] | 68 | 2 |
| Gene.2562 | hypothetical protein AMK59_6989 [Oryctes borbonicus] | 168 | 2 |
| Gene.2563 | PREDICTED: aldose reductase [Tribolium castaneum] | 104 | 2 |
| Gene.2565 | PREDICTED: protein hu-li tai shao isoform X2 [Tribolium castaneum] | 37 | 2 |
| Gene.2568 | coatomer subunit delta [Anoplophora glabripennis] | 335 | 2 |
| Gene.2569 | 1,5-anhydro-D-fructose reductase-like Protein [Tribolium castaneum] | 312 | 2 |
| Gene.2583 | rRNA 2'-O-methyltransferase fibrillarin [Agrilus planipennis] | 139 | 2 |
| Gene.2587 | Uncharacterized protein | 241 | 2 |
| Gene.2650 | PREDICTED: succinyl-CoA ligase [ADP-forming] subunit beta, mitochondrial [Tribolium castaneum] | 67 | 2 |
| Gene.2650 | PREDICTED: succinyl-CoA ligase [ADP-forming] subunit beta, mitochondrial [Tribolium castaneum] | 77 | 2 |
| Gene.2650 | PREDICTED: succinyl-CoA ligase [ADP-forming] subunit beta, mitochondrial [Tribolium castaneum] | 132 | 2 |
| Gene.2689 | CoaE and/or CTP transf 2 domain containing protein [Asbolus verrucosus] | 157 | 2 |
| Gene.2689 | CoaE and/or CTP transf 2 domain containing protein [Asbolus verrucosus] | 143 | 2 |
| Gene.2695 | PREDICTED: translocon-associated protein subunit alpha [Nicrophorus vespilloides] | 104 | 2 |
| Gene.2724 | PREDICTED: endoplasmin [Tribolium castaneum] | 48 | 2 |
| Gene.2725 | PREDICTED: endoplasmin [Tribolium castaneum] | 56 | 2 |
| Gene.2725 | PREDICTED: endoplasmin [Tribolium castaneum] | 219 | 2 |
| Gene.2725 | PREDICTED: endoplasmin [Tribolium castaneum] | 487 | 2 |
| Gene.2749 | PREDICTED: aldehyde dehydrogenase, mitochondrial [Tribolium castaneum] | 43 | 2 |
| Gene.2764 | PREDICTED: alpha-L-fucosidase [Nicrophorus vespilloides] | 200 | 2 |
| Gene.2767 | ribosomal protein L10, partial [Harmonia axyridis] | 39 | 2 |
| Gene.2767 | ribosomal protein L10, partial [Harmonia axyridis] | 87 | 2 |
| Gene.2767 | ribosomal protein L10, partial [Harmonia axyridis] | 187 | 2 |
| Gene.2767 | ribosomal protein L10, partial [Harmonia axyridis] | 110 | 2 |
| Gene.2830 | PREDICTED: LOW QUALITY PROTEIN: clathrin heavy chain [Aethina tumida] | 1354 | 2 |
| Gene.2830 | PREDICTED: LOW QUALITY PROTEIN: clathrin heavy chain [Aethina tumida] | 1216 | 2 |
| Gene.2830 | PREDICTED: LOW QUALITY PROTEIN: clathrin heavy chain [Aethina tumida] | 396 | 2 |
| Gene.2857 | C-1-tetrahydrofolate synthase, cytoplasmic [Onthophagus taurus] | 716 | 2 |
| Gene.2878 | fatty acid synthase-like [Agrilus planipennis] | 949 | 2 |
| Gene.2933 | isocitrate dehydrogenase [NADP], mitochondrial-like [Onthophagus taurus] | 387 | 2 |
| Gene.2933 | isocitrate dehydrogenase [NADP], mitochondrial-like [Onthophagus taurus] | 263 | 2 |
| Gene.2933 | isocitrate dehydrogenase [NADP], mitochondrial-like [Onthophagus taurus] | 119 | 2 |
| Gene.2958 | alpha actinin [Coleomegilla maculata] | 92 | 2 |
| Gene.2958 | alpha actinin [Coleomegilla maculata] | 98 | 2 |
| Gene.2958 | alpha actinin [Coleomegilla maculata] | 198 | 2 |
| Gene.298 | Csa-calmodulin 3, partial [Cupiennius salei] | 22 | 2 |
| Gene.2997 | PREDICTED: pyruvate kinase-like isoform X3 [Dendroctonus ponderosae] | 218 | 2 |
| Gene.2997 | PREDICTED: pyruvate kinase-like isoform X3 [Dendroctonus ponderosae] | 258 | 2 |
| Gene.3075 | dihydrolipoyl dehydrogenase, mitochondrial [Anoplophora glabripennis] | 142 | 2 |
| Gene.310 | 60S ribosomal protein L10a [Monochamus alternatus] | 147 | 2 |
| Gene.310 | 60S ribosomal protein L10a [Monochamus alternatus] | 54 | 2 |
| Gene.312 | heterogeneous nuclear ribonucleoprotein H [Asbolus verrucosus] | 161 | 2 |
| Gene.3147 | Uncharacterized protein | 210 | 2 |
| Gene.3149 | PREDICTED: aspartate--tRNA ligase, cytoplasmic [Tribolium castaneum] | 409 | 2 |
| Gene.3186 | facilitated trehalose transporter Tret1-like isoform X2 [Anoplophora glabripennis] | 335 | 2 |
| Gene.3208 | phosphoglucose isomerase [Colias eurytheme] | 67 | 2 |
| Gene.3222 | hypothetical protein YQE_08859, partial [Dendroctonus ponderosae] | 165 | 2 |
| Gene.3243 | cytochrome P450 monooxygenase [Tribolium castaneum] | 459 | 2 |
| Gene.3243 | cytochrome P450 monooxygenase [Tribolium castaneum] | 249 | 2 |
| Gene.331 | PREDICTED: ferritin subunit [Aethina tumida] | 81 | 2 |
| Gene.3380 | PREDICTED: ribonucleoside-diphosphate reductase large subunit-like [Aethina tumida] | 501 | 2 |
| Gene.3401 | glutathione S-transferase-like [Anoplophora glabripennis] | 139 | 2 |
| Gene.3460 | PREDICTED: LOW QUALITY PROTEIN: papilin [Tribolium castaneum] | 1762 | 2 |
| Gene.3460 | PREDICTED: LOW QUALITY PROTEIN: papilin [Tribolium castaneum] | 1735 | 2 |
| Gene.3466 | PREDICTED: signal transducing adapter molecule 2 [Tribolium castaneum] | 292 | 2 |
| Gene.3489 | PREDICTED: acetyl-CoA carboxylase isoform X1 [Tribolium castaneum] | 2308 | 2 |
| Gene.3489 | PREDICTED: acetyl-CoA carboxylase isoform X1 [Tribolium castaneum] | 1259 | 2 |
| Gene.3489 | PREDICTED: acetyl-CoA carboxylase isoform X1 [Tribolium castaneum] | 2341 | 2 |
| Gene.3498 | 40S ribosomal protein S8 [Anoplophora glabripennis] | 157 | 2 |
| Gene.3501 | PREDICTED: PDZ and LIM domain protein Zasp isoform X5 [Tribolium castaneum] | 151 | 2 |
| Gene.3522 | vitellogenin 1 [Harmonia axyridis] | 276 | 2 |
| Gene.354 | saccharopine dehydrogenase-like [Asbolus verrucosus] | 322 | 2 |
| Gene.354 | saccharopine dehydrogenase-like [Asbolus verrucosus] | 222 | 2 |
| Gene.3558 | PREDICTED: probable aconitate hydratase, mitochondrial [Tribolium castaneum] | 664 | 2 |
| Gene.3562 | vinculin isoform X1 [Anoplophora glabripennis] | 601 | 2 |
| Gene.3579 | transmembrane GTPase Marf [Asbolus verrucosus] | 294 | 2 |
| Gene.3581 | PREDICTED: glycerol-3-phosphate dehydrogenase, mitochondrial isoform X2 [Tribolium castaneum] | 60 | 2 |
| Gene.3583 | 4-coumarate--CoA ligase-like [Leptinotarsa decemlineata] | 595 | 2 |
| Gene.3583 | 4-coumarate--CoA ligase-like [Leptinotarsa decemlineata] | 590 | 2 |
| Gene.3627 | UDP-glucuronosyltransferase 2C1-like, partial [Asbolus verrucosus] | 49 | 2 |
| Gene.3634 | sugar transporter 14 [Tenebrio molitor] | 235 | 2 |
| Gene.3656 | PREDICTED: prisilkin-39 [Tribolium castaneum] | 92 | 2 |
| Gene.3656 | PREDICTED: prisilkin-39 [Tribolium castaneum] | 144 | 2 |
| Gene.3663 | PREDICTED: uncharacterized protein LOC659539 isoform X1 [Tribolium castaneum] | 533 | 2 |
| Gene.3663 | PREDICTED: uncharacterized protein LOC659539 isoform X1 [Tribolium castaneum] | 245 | 2 |
| Gene.3676 | hypothetical protein D910_04355 [Dendroctonus ponderosae] | 271 | 2 |
| Gene.3677 | venom polypeptide [Dolopus genitalis] | 14 | 2 |
| Gene.3708 | phosphate carrier protein, mitochondrial-like [Leptinotarsa decemlineata] | 93 | 2 |
| Gene.3708 | phosphate carrier protein, mitochondrial-like [Leptinotarsa decemlineata] | 95 | 2 |
| Gene.3708 | phosphate carrier protein, mitochondrial-like [Leptinotarsa decemlineata] | 183 | 2 |
| Gene.3709 | 60S ribosomal protein L27 [Anoplophora glabripennis] | 36 | 2 |
| Gene.3723 | PREDICTED: fructose-1,6-bisphosphatase 1 [Tribolium castaneum] | 201 | 2 |
| Gene.3731 | PREDICTED: UDP-glucuronosyltransferase 2B2 [Tribolium castaneum] | 132 | 2 |
| Gene.3734 | PREDICTED: UDP-glucuronosyltransferase 2B7 isoform X1 [Tribolium castaneum] | 294 | 2 |
| Gene.3776 | PREDICTED: scaffold attachment factor B1 isoform X1 [Tribolium castaneum] | 393 | 2 |
| Gene.3790 | PREDICTED: 60S ribosomal protein L3 [Tribolium castaneum] | 377 | 2 |
| Gene.3838 | PREDICTED: pyruvate carboxylase, mitochondrial isoform X3 [Tribolium castaneum] | 385 | 2 |
| Gene.3838 | PREDICTED: pyruvate carboxylase, mitochondrial isoform X3 [Tribolium castaneum] | 1070 | 2 |
| Gene.3838 | PREDICTED: pyruvate carboxylase, mitochondrial isoform X3 [Tribolium castaneum] | 1119 | 2 |
| Gene.3838 | PREDICTED: pyruvate carboxylase, mitochondrial isoform X3 [Tribolium castaneum] | 69 | 2 |
| Gene.3875 | PREDICTED: probable methylmalonate-semialdehyde dehydrogenase [acylating], mitochondrial [Aethina tumida] | 250 | 2 |
| Gene.3947 | PREDICTED: delta-1-pyrroline-5-carboxylate synthase [Tribolium castaneum] | 613 | 2 |
| Gene.3947 | PREDICTED: delta-1-pyrroline-5-carboxylate synthase [Tribolium castaneum] | 415 | 2 |
| Gene.3973 | PREDICTED: thioredoxin reductase 1, mitochondrial isoform X6 [Tribolium castaneum] | 32 | 2 |
| Gene.3996 | myosin-IB [Anoplophora glabripennis] | 54 | 2 |
| Gene.3999 | NAD binding 2 domain containing protein [Asbolus verrucosus] | 210 | 2 |
| Gene.400 | serine--tRNA ligase, cytoplasmic [Leptinotarsa decemlineata] | 155 | 2 |
| Gene.4011 | fatty acid synthase [Coccinella septempunctata] | 71 | 2 |
| Gene.4011 | fatty acid synthase [Coccinella septempunctata] | 2113 | 2 |
| Gene.4011 | fatty acid synthase [Coccinella septempunctata] | 1125 | 2 |
| Gene.4011 | fatty acid synthase [Coccinella septempunctata] | 1070 | 2 |
| Gene.4015 | activated RNA polymerase II transcriptional coactivator p15 [Asbolus verrucosus] | 40 | 2 |
| Gene.4016 | AAEL010007-PA [Aedes aegypti] | 180 | 2 |
| Gene.4018 | glutamyl aminopeptidase-like isoform X2 [Leptinotarsa decemlineata] | 107 | 2 |
| Gene.4035 | Uncharacterized protein | 14 | 2 |
| Gene.407 | thioredoxin-related transmembrane protein 1 [Anoplophora glabripennis] | 80 | 2 |
| Gene.4078 | uncharacterized protein LOC108917852 [Anoplophora glabripennis] | 393 | 2 |
| Gene.4113 | succinyl-CoA:3-ketoacid coenzyme A transferase 1, mitochondrial, partial [Asbolus verrucosus] | 177 | 2 |
| Gene.4113 | succinyl-CoA:3-ketoacid coenzyme A transferase 1, mitochondrial, partial [Asbolus verrucosus] | 125 | 2 |
| Gene.4132 | phosphoribosylformylglycinamidine synthase [Anoplophora glabripennis] | 277 | 2 |
| Gene.4134 | PREDICTED: AMP deaminase 2 isoform X6 [Tribolium castaneum] | 371 | 2 |
| Gene.4134 | PREDICTED: AMP deaminase 2 isoform X6 [Tribolium castaneum] | 352 | 2 |
| Gene.4178 | mitochondrial 2-oxoglutarate/malate carrier protein [Asbolus verrucosus] | 241 | 2 |
| Gene.4190 | V-type proton ATPase subunit H [Asbolus verrucosus] | 11 | 2 |
| Gene.4205 | uncharacterized protein LOC108911898 [Anoplophora glabripennis] | 532 | 2 |
| Gene.425 | glutathione S-transferase 1-1 [Anoplophora glabripennis] | 87 | 2 |
| Gene.426 | PREDICTED: LOW QUALITY PROTEIN: amidophosphoribosyltransferase-like [Aethina tumida] | 476 | 2 |
| Gene.4262 | sarcosine dehydrogenase, mitochondrial [Asbolus verrucosus] | 608 | 2 |
| Gene.4271 | PREDICTED: NADP-dependent malic enzyme isoform X1 [Megachile rotundata] | 188 | 2 |
| Gene.4272 | PREDICTED: LOW QUALITY PROTEIN: NADP-dependent malic enzyme-like [Aethina tumida] | 107 | 2 |
| Gene.4285 | PREDICTED: LOW QUALITY PROTEIN: pyruvate kinase-like [Aethina tumida] | 253 | 2 |
| Gene.429 | 40S ribosomal protein S16 [Anoplophora glabripennis] | 66 | 2 |
| Gene.4299 | electron transfer flavoprotein-ubiquinone oxidoreductase, mitochondrial [Anoplophora glabripennis] | 149 | 2 |
| Gene.4302 | long-chain-fatty-acid--CoA ligase 4 isoform X1 [Agrilus planipennis] | 155 | 2 |
| Gene.4303 | PREDICTED: LOW QUALITY PROTEIN: long-chain-fatty-acid--CoA ligase 4 [Aethina tumida] | 165 | 2 |
| Gene.4303 | PREDICTED: LOW QUALITY PROTEIN: long-chain-fatty-acid--CoA ligase 4 [Aethina tumida] | 208 | 2 |
| Gene.4311 | 39S ribosomal protein L50, mitochondrial [Anoplophora glabripennis] | 137 | 2 |
| Gene.4326 | hemocytin [Agrilus planipennis] | 467 | 2 |
| Gene.4352 | PREDICTED: calcium-transporting ATPase sarcoplasmic/endoplasmic reticulum type isoform X1 [Tribolium castaneum] | 541 | 2 |
| Gene.4352 | PREDICTED: calcium-transporting ATPase sarcoplasmic/endoplasmic reticulum type isoform X1 [Tribolium castaneum] | 218 | 2 |
| Gene.4439 | Basement membrane-specific heparan sulfate proteoglycan core protein-like Protein [Tribolium castaneum] | 509 | 2 |
| Gene.4439 | Basement membrane-specific heparan sulfate proteoglycan core protein-like Protein [Tribolium castaneum] | 150 | 2 |
| Gene.4487 | dosage compensation regulator isoform X1 [Leptinotarsa decemlineata] | 621 | 2 |
| Gene.455 | ATP-binding cassette sub-family E member 1 [Anoplophora glabripennis] | 511 | 2 |
| Gene.4581 | PREDICTED: trifunctional enzyme subunit alpha, mitochondrial [Tribolium castaneum] | 488 | 2 |
| Gene.4583 | PREDICTED: ATP-citrate synthase isoform X1 [Tribolium castaneum] | 456 | 2 |
| Gene.4593 | spectrin beta chain isoform X1 [Anoplophora glabripennis] | 240 | 2 |
| Gene.4593 | spectrin beta chain isoform X1 [Anoplophora glabripennis] | 233 | 2 |
| Gene.4593 | spectrin beta chain isoform X1 [Anoplophora glabripennis] | 375 | 2 |
| Gene.4593 | spectrin beta chain isoform X1 [Anoplophora glabripennis] | 337 | 2 |
| Gene.4653 | Uncharacterized protein | 1200 | 2 |
| Gene.468 | retinal dehydrogenase 1, partial [Asbolus verrucosus] | 14 | 2 |
| Gene.469 | AP-1 complex subunit beta-1 [Asbolus verrucosus] | 11 | 2 |
| Gene.4718 | AGAP001345-PA [Anopheles gambiae str. PEST] | 254 | 2 |
| Gene.473 | T-complex protein 1 subunit zeta [Asbolus verrucosus] | 438 | 2 |
| Gene.475 | PREDICTED: myosin heavy chain 95F isoform X1 [Tribolium castaneum] | 606 | 2 |
| Gene.4752 | hexamerin 3 precursor [Tribolium castaneum] | 721 | 2 |
| Gene.4752 | hexamerin 3 precursor [Tribolium castaneum] | 631 | 2 |
| Gene.4752 | hexamerin 3 precursor [Tribolium castaneum] | 724 | 2 |
| Gene.4754 | NADPH--cytochrome P450 reductase isoform X1 [Anoplophora glabripennis] | 360 | 2 |
| Gene.4787 | paramyosin, long form isoform X2 [Leptinotarsa decemlineata] | 224 | 2 |
| Gene.4867 | PREDICTED: spectrin alpha chain isoform X4 [Tribolium castaneum] | 72 | 2 |
| Gene.4867 | PREDICTED: spectrin alpha chain isoform X4 [Tribolium castaneum] | 525 | 2 |
| Gene.4867 | PREDICTED: spectrin alpha chain isoform X4 [Tribolium castaneum] | 463 | 2 |
| Gene.4867 | PREDICTED: spectrin alpha chain isoform X4 [Tribolium castaneum] | 1163 | 2 |
| Gene.4867 | PREDICTED: spectrin alpha chain isoform X4 [Tribolium castaneum] | 121 | 2 |
| Gene.4934 | apolipophorins [Anoplophora glabripennis] | 785 | 2 |
| Gene.4973 | PREDICTED: myosin heavy chain, muscle isoform X18 [Tribolium castaneum] | 1606 | 2 |
| Gene.4973 | PREDICTED: myosin heavy chain, muscle isoform X18 [Tribolium castaneum] | 616 | 2 |
| Gene.500 | alanine aminotransferase 1 [Anoplophora glabripennis] | 484 | 2 |
| Gene.500 | alanine aminotransferase 1 [Anoplophora glabripennis] | 478 | 2 |
| Gene.5002 | titin [Asbolus verrucosus] | 2246 | 2 |
| Gene.5034 | PREDICTED: LOW QUALITY PROTEIN: cytoplasmic aconitate hydratase-like [Aethina tumida] | 162 | 2 |
| Gene.5096 | Ankyrin-2-like Protein [Tribolium castaneum] | 1581 | 2 |
| Gene.5143 | PREDICTED: talin-1 isoform X1 [Tribolium castaneum] | 526 | 2 |
| Gene.515 | hypothetical protein D910_12346, partial [Dendroctonus ponderosae] | 38 | 2 |
| Gene.5201 | PREDICTED: histone-lysine N-methyltransferase 2C-like isoform X2 [Aethina tumida] | 158 | 2 |
| Gene.5327 | FUN14 domain-containing protein 1 isoform X1 [Agrilus planipennis] | 98 | 2 |
| Gene.5331 | PREDICTED: synaptobrevin homolog YKT6 [Tribolium castaneum] | 186 | 2 |
| Gene.5388 | PREDICTED: myophilin [Tribolium castaneum] | 144 | 2 |
| Gene.5401 | transmembrane emp24 domain-containing protein bai, partial [Anoplophora glabripennis] | 149 | 2 |
| Gene.5402 | PREDICTED: transmembrane emp24 domain-containing protein eca-like [Nicrophorus vespilloides] | 82 | 2 |
| Gene.5416 | PREDICTED: proteasome activator complex subunit 3 isoform X2 [Tribolium castaneum] | 43 | 2 |
| Gene.5445 | PREDICTED: probable peroxisomal acyl-coenzyme A oxidase 1 [Aethina tumida] | 12 | 2 |
| Gene.5478 | 60S ribosomal protein L7a-like [Leptinotarsa decemlineata] | 74 | 2 |
| Gene.5498 | 40S ribosomal protein S10b [Anoplophora glabripennis] | 63 | 2 |
| Gene.5498 | 40S ribosomal protein S10b [Anoplophora glabripennis] | 138 | 2 |
| Gene.5498 | 40S ribosomal protein S10b [Anoplophora glabripennis] | 30 | 2 |
| Gene.5511 | PREDICTED: nucleoplasmin-like protein [Aethina tumida] | 67 | 2 |
| Gene.5602 | 60S ribosomal protein L13 [Anoplophora glabripennis] | 211 | 2 |
| Gene.5675 | PREDICTED: secretory carrier-associated membrane protein 1 [Tribolium castaneum] | 290 | 2 |
| Gene.572 | PREDICTED: phosphoglycerate kinase [Tribolium castaneum] | 250 | 2 |
| Gene.572 | PREDICTED: phosphoglycerate kinase [Tribolium castaneum] | 52 | 2 |
| Gene.572 | PREDICTED: phosphoglycerate kinase [Tribolium castaneum] | 144 | 2 |
| Gene.5733 | PREDICTED: prohibitin-2 isoform X2 [Tribolium castaneum] | 12 | 2 |
| Gene.5733 | PREDICTED: prohibitin-2 isoform X2 [Tribolium castaneum] | 5 | 2 |
| Gene.5734 | electron transfer flavoprotein subunit alpha, mitochondrial, partial [Asbolus verrucosus] | 118 | 2 |
| Gene.5739 | PREDICTED: LOW QUALITY PROTEIN: mitochondrial import inner membrane translocase subunit TIM50-C-like [Aethina tumida] | 261 | 2 |
| Gene.5746 | PREDICTED: ATP synthase subunit delta, mitochondrial [Tribolium castaneum] | 42 | 2 |
| Gene.5770 | PREDICTED: 26S protease regulatory subunit 10B [Nicrophorus vespilloides] | 317 | 2 |
| Gene.580 | PREDICTED: cytochrome b-c1 complex subunit 2, mitochondrial [Tribolium castaneum] | 354 | 2 |
| Gene.5803 | calcium-binding protein E63-1 [Anoplophora glabripennis] | 114 | 2 |
| Gene.5850 | PREDICTED: hrp65 protein-like [Aethina tumida] | 384 | 2 |
| Gene.5850 | PREDICTED: hrp65 protein-like [Aethina tumida] | 344 | 2 |
| Gene.5868 | PREDICTED: V-type proton ATPase subunit D [Polistes canadensis] | 55 | 2 |
| Gene.5891 | prefoldin subunit 3-like [Anoplophora glabripennis] | 6 | 2 |
| Gene.5896 | PREDICTED: cdc42 homolog [Tribolium castaneum] | 128 | 2 |
| Gene.5896 | PREDICTED: cdc42 homolog [Tribolium castaneum] | 144 | 2 |
| Gene.5899 | proteasome subunit beta type-3 [Anoplophora glabripennis] | 198 | 2 |
| Gene.5928 | PREDICTED: 40S ribosomal protein S3a [Aethina tumida] | 239 | 2 |
| Gene.595 | troponin T, skeletal muscle isoform X5 [Leptinotarsa decemlineata] | 94 | 2 |
| Gene.5969 | Ribose-phosphate pyrophosphokinase 1-like Protein [Tribolium castaneum] | 227 | 2 |
| Gene.5991 | PREDICTED: NADH dehydrogenase [ubiquinone] 1 beta subcomplex subunit 10 [Tribolium castaneum] | 47 | 2 |
| Gene.5994 | PREDICTED: LOW QUALITY PROTEIN: histidine triad nucleotide-binding protein 1-like [Aethina tumida] | 131 | 2 |
| Gene.5994 | PREDICTED: LOW QUALITY PROTEIN: histidine triad nucleotide-binding protein 1-like [Aethina tumida] | 73 | 2 |
| Gene.6014 | PREDICTED: ubiquitin-fold modifier-conjugating enzyme 1 [Tribolium castaneum] | 166 | 2 |
| Gene.6041 | eukaryotic translation initiation factor 3 subunit L [Anoplophora glabripennis] | 530 | 2 |
| Gene.6041 | eukaryotic translation initiation factor 3 subunit L [Anoplophora glabripennis] | 268 | 2 |
| Gene.6128 | protein HGV2-like isoform X1 [Leptinotarsa decemlineata] | 14 | 2 |
| Gene.6152 | serine/threonine-protein phosphatase 2A 56 kDa regulatory subunit epsilon isoform isoform X1 [Anoplophora glabripennis] | 203 | 2 |
| Gene.6191 | PREDICTED: Na(+)/H(+) exchange regulatory cofactor NHE-RF2 [Aethina tumida] | 42 | 2 |
| Gene.6222 | Pro isomerase domain containing protein [Asbolus verrucosus] | 80 | 2 |
| Gene.6232 | PREDICTED: muscle M-line assembly protein unc-89-like [Aethina tumida] | 57 | 2 |
| Gene.6234 | proteasome subunit alpha type-3 [Anoplophora glabripennis] | 205 | 2 |
| Gene.624 | PREDICTED: staphylococcal nuclease domain-containing protein 1 [Tribolium castaneum] | 323 | 2 |
| Gene.6253 | PREDICTED: ras-like GTP-binding protein Rho1 [Aethina tumida] | 197 | 2 |
| Gene.6255 | probable prefoldin subunit 4 isoform X1 [Anoplophora glabripennis] | 46 | 2 |
| Gene.6285 | PREDICTED: protein krasavietz isoform X1 [Aethina tumida] | 283 | 2 |
| Gene.6297 | PREDICTED: V-type proton ATPase subunit C isoform X3 [Tribolium castaneum] | 238 | 2 |
| Gene.6297 | PREDICTED: V-type proton ATPase subunit C isoform X3 [Tribolium castaneum] | 171 | 2 |
| Gene.6334 | PREDICTED: patj homolog isoform X1 [Tribolium castaneum] | 51 | 2 |
| Gene.6334 | PREDICTED: patj homolog isoform X1 [Tribolium castaneum] | 65 | 2 |
| Gene.6375 | proliferation-associated protein 2G4 [Anoplophora glabripennis] | 31 | 2 |
| Gene.6402 | Uncharacterized protein | 60 | 2 |
| Gene.6439 | PREDICTED: protein DEK isoform X3 [Tribolium castaneum] | 246 | 2 |
| Gene.6439 | PREDICTED: protein DEK isoform X3 [Tribolium castaneum] | 460 | 2 |
| Gene.6493 | probable transaldolase [Leptinotarsa decemlineata] | 295 | 2 |
| Gene.660 | PREDICTED: SH3 domain-binding glutamic acid-rich protein homolog [Tribolium castaneum] | 30 | 2 |
| Gene.6601 | putative ribosomal protein L6 [Phaedon cochleariae] | 251 | 2 |
| Gene.6601 | putative ribosomal protein L6 [Phaedon cochleariae] | 67 | 2 |
| Gene.6601 | putative ribosomal protein L6 [Phaedon cochleariae] | 53 | 2 |
| Gene.6601 | putative ribosomal protein L6 [Phaedon cochleariae] | 73 | 2 |
| Gene.6660 | probable 2-oxoglutarate dehydrogenase E1 component DHKTD1 homolog, mitochondrial [Leptinotarsa decemlineata] | 62 | 2 |
| Gene.6679 | Isocitrate dehydrogenase [NADP] [Operophtera brumata] | 125 | 2 |
| Gene.6689 | eukaryotic translation initiation factor 3 subunit G [Asbolus verrucosus] | 105 | 2 |
| Gene.6692 | D-3-phosphoglycerate dehydrogenase, partial [Asbolus verrucosus] | 32 | 2 |
| Gene.6692 | D-3-phosphoglycerate dehydrogenase, partial [Asbolus verrucosus] | 181 | 2 |
| Gene.6721 | nuclear valosin-containing protein-like isoform X1 [Anoplophora glabripennis] | 486 | 2 |
| Gene.68 | PREDICTED: prolyl endopeptidase isoform X1 [Tribolium castaneum] | 240 | 2 |
| Gene.6809 | 60S ribosomal protein L12 [Lucilia cuprina] | 48 | 2 |
| Gene.6827 | myosin light chain alkali, partial [Asbolus verrucosus] | 42 | 2 |
| Gene.6878 | SUMO-conjugating enzyme UBC9-B [Agrilus planipennis] | 65 | 2 |
| Gene.6879 | V-type proton ATPase subunit E [Leptinotarsa decemlineata] | 52 | 2 |
| Gene.6895 | PREDICTED: acyl-CoA-binding domain-containing protein 5 [Aethina tumida] | 231 | 2 |
| Gene.6924 | PREDICTED: 40S ribosomal protein SA [Tribolium castaneum] | 216 | 2 |
| Gene.6958 | PREDICTED: S-methyl-5'-thioadenosine phosphorylase isoform X2 [Nicrophorus vespilloides] | 241 | 2 |
| Gene.6958 | PREDICTED: S-methyl-5'-thioadenosine phosphorylase isoform X2 [Nicrophorus vespilloides] | 175 | 2 |
| Gene.6967 | 60S ribosomal protein L27a [Trachymyrmex cornetzi] | 60 | 2 |
| Gene.6969 | kynurenine--oxoglutarate transaminase 3 [Anoplophora glabripennis] | 363 | 2 |
| Gene.6977 | AFG3-like protein 2 [Anoplophora glabripennis] | 605 | 2 |
| Gene.6977 | AFG3-like protein 2 [Anoplophora glabripennis] | 295 | 2 |
| Gene.6997 | PREDICTED: glyoxylate reductase/hydroxypyruvate reductase [Dendroctonus ponderosae] | 201 | 2 |
| Gene.7 | ATP-dependent RNA helicase WM6, partial [Asbolus verrucosus] | 136 | 2 |
| Gene.7019 | PREDICTED: ultraviolet-B receptor UVR8 [Tribolium castaneum] | 27 | 2 |
| Gene.7038 | 60S ribosomal protein L18a [Leptinotarsa decemlineata] | 38 | 2 |
| Gene.7038 | 60S ribosomal protein L18a [Leptinotarsa decemlineata] | 178 | 2 |
| Gene.704 | polyadenylate-binding protein 4-like [Anoplophora glabripennis] | 261 | 2 |
| Gene.704 | polyadenylate-binding protein 4-like [Anoplophora glabripennis] | 301 | 2 |
| Gene.7116 | PREDICTED: 26S protease regulatory subunit 4 [Nicrophorus vespilloides] | 96 | 2 |
| Gene.7125 | Ribosomal L7Ae domain containing protein [Asbolus verrucosus] | 86 | 2 |
| Gene.7129 | PREDICTED: fumarylacetoacetate hydrolase domain-containing protein 2 [Aethina tumida] | 38 | 2 |
| Gene.7132 | 60S ribosomal protein L23a-like, partial [Asbolus verrucosus] | 238 | 2 |
| Gene.7190 | PREDICTED: 14 kDa phosphohistidine phosphatase-like [Dendroctonus ponderosae] | 100 | 2 |
| Gene.7216 | PREDICTED: microtubule-associated protein RP/EB family member 1 isoform X2 [Tribolium castaneum] | 224 | 2 |
| Gene.7218 | MICOS complex subunit Mic60 isoform X1 [Leptinotarsa decemlineata] | 257 | 2 |
| Gene.7236 | Uncharacterized protein | 100 | 2 |
| Gene.7237 | Uncharacterized protein | 76 | 2 |
| Gene.725 | PREDICTED: myosin regulatory light chain 2 [Aethina tumida] | 51 | 2 |
| Gene.730 | long-chain-fatty-acid--CoA ligase ACSBG2 [Anoplophora glabripennis] | 299 | 2 |
| Gene.7309 | PREDICTED: methionine aminopeptidase 2 [Tribolium castaneum] | 306 | 2 |
| Gene.7309 | PREDICTED: methionine aminopeptidase 2 [Tribolium castaneum] | 255 | 2 |
| Gene.7356 | PREDICTED: peroxiredoxin-1 [Tribolium castaneum] | 51 | 2 |
| Gene.7378 | PREDICTED: delta(3,5)-Delta(2,4)-dienoyl-CoA isomerase, mitochondrial [Tribolium castaneum] | 196 | 2 |
| Gene.738 | PREDICTED: elongation factor Tu, mitochondrial [Aethina tumida] | 90 | 2 |
| Gene.738 | PREDICTED: elongation factor Tu, mitochondrial [Aethina tumida] | 107 | 2 |
| Gene.7388 | short-chain specific acyl-CoA dehydrogenase, mitochondrial [Anoplophora glabripennis] | 46 | 2 |
| Gene.7409 | PREDICTED: T-complex protein 1 subunit delta [Tribolium castaneum] | 316 | 2 |
| Gene.742 | NADH dehydrogenase [ubiquinone] 1 alpha subcomplex subunit 9, mitochondrial [Anoplophora glabripennis] | 239 | 2 |
| Gene.742 | NADH dehydrogenase [ubiquinone] 1 alpha subcomplex subunit 9, mitochondrial [Anoplophora glabripennis] | 157 | 2 |
| Gene.7424 | carbonyl reductase [NADPH] 3 [Anoplophora glabripennis] | 171 | 2 |
| Gene.7482 | failed axon connections isoform X1 [Anoplophora glabripennis] | 32 | 2 |
| Gene.7482 | failed axon connections isoform X1 [Anoplophora glabripennis] | 57 | 2 |
| Gene.7502 | GMP reductase 1-like [Anoplophora glabripennis] | 291 | 2 |
| Gene.7513 | uncharacterized protein LOC111511572 [Leptinotarsa decemlineata] | 169 | 2 |
| Gene.7551 | PREDICTED: non-specific lipid-transfer protein [Aethina tumida] | 502 | 2 |
| Gene.7565 | heat shock protein 21.62 [Harmonia axyridis] | 110 | 2 |
| Gene.7566 | PREDICTED: protein disulfide-isomerase A6 [Tribolium castaneum] | 197 | 2 |
| Gene.7626 | putative oxidoreductase GLYR1 homolog [Anoplophora glabripennis] | 71 | 2 |
| Gene.7626 | putative oxidoreductase GLYR1 homolog [Anoplophora glabripennis] | 96 | 2 |
| Gene.7644 | PREDICTED: aldose reductase [Tribolium castaneum] | 262 | 2 |
| Gene.7671 | 4-aminobutyrate aminotransferase, mitochondrial [Anoplophora glabripennis] | 220 | 2 |
| Gene.77 | cathepsin L [Anoplophora glabripennis] | 229 | 2 |
| Gene.7734 | PREDICTED: LOW QUALITY PROTEIN: adenosylhomocysteinase [Aethina tumida] | 226 | 2 |
| Gene.7777 | PREDICTED: asparagine--tRNA ligase, cytoplasmic [Tribolium castaneum] | 79 | 2 |
| Gene.78 | PREDICTED: ATP-dependent RNA helicase p62-like [Aethina tumida] | 386 | 2 |
| Gene.7808 | PREDICTED: THUMP domain-containing protein 1 homolog [Aethina tumida] | 117 | 2 |
| Gene.7808 | PREDICTED: THUMP domain-containing protein 1 homolog [Aethina tumida] | 175 | 2 |
| Gene.7809 | pollen-specific leucine-rich repeat extensin-like protein 1 [Asbolus verrucosus] | 124 | 2 |
| Gene.7815 | probable pyruvate dehydrogenase E1 component subunit alpha, mitochondrial isoform X2 [Leptinotarsa decemlineata] | 57 | 2 |
| Gene.7816 | ubiquitin carboxyl-terminal hydrolase 7 isoform X1 [Anoplophora glabripennis] | 385 | 2 |
| Gene.7840 | PREDICTED: cytochrome c1, heme protein, mitochondrial [Tribolium castaneum] | 184 | 2 |
| Gene.7860 | PREDICTED: lipid storage droplets surface-binding protein 1 isoform X1 [Aethina tumida] | 213 | 2 |
| Gene.7878 | alcohol dehydrogenase class-3 [Leptinotarsa decemlineata] | 23 | 2 |
| Gene.7878 | alcohol dehydrogenase class-3 [Leptinotarsa decemlineata] | 235 | 2 |
| Gene.789 | PREDICTED: putative hydroxypyruvate isomerase isoform X2 [Aethina tumida] | 186 | 2 |
| Gene.7917 | ribosomal protein S3 [Tenebrio molitor] | 62 | 2 |
| Gene.792 | imaginal disc growth factor 4 precursor [Tribolium castaneum] | 121 | 2 |
| Gene.7929 | PREDICTED: heat shock 70 kDa protein cognate 3 [Dendroctonus ponderosae] | 573 | 2 |
| Gene.7954 | eukaryotic translation initiation factor 3 subunit C [Anoplophora glabripennis] | 154 | 2 |
| Gene.7954 | eukaryotic translation initiation factor 3 subunit C [Anoplophora glabripennis] | 245 | 2 |
| Gene.7997 | Y-box factor homolog isoform X3 [Leptinotarsa decemlineata] | 51 | 2 |
| Gene.8021 | ribosomal protein L4e [Biphyllus lunatus] | 184 | 2 |
| Gene.8021 | ribosomal protein L4e [Biphyllus lunatus] | 60 | 2 |
| Gene.8087 | PREDICTED: adenylosuccinate synthetase-like [Aethina tumida] | 191 | 2 |
| Gene.8087 | PREDICTED: adenylosuccinate synthetase-like [Aethina tumida] | 239 | 2 |
| Gene.8096 | PREDICTED: ADP,ATP carrier protein 1 [Tribolium castaneum] | 174 | 2 |
| Gene.810 | PREDICTED: 60S ribosomal protein L17 [Aethina tumida] | 62 | 2 |
| Gene.810 | PREDICTED: 60S ribosomal protein L17 [Aethina tumida] | 55 | 2 |
| Gene.8102 | glucosidase 2 subunit beta-like [Leptinotarsa decemlineata] | 164 | 2 |
| Gene.8135 | PREDICTED: calreticulin [Tribolium castaneum] | 41 | 2 |
| Gene.8135 | PREDICTED: calreticulin [Tribolium castaneum] | 76 | 2 |
| Gene.8167 | malate dehydrogenase, cytoplasmic [Leptinotarsa decemlineata] | 297 | 2 |
| Gene.8218 | PREDICTED: 26S proteasome non-ATPase regulatory subunit 14 isoform X1 [Aethina tumida] | 258 | 2 |
| Gene.8220 | glutathione S-transferase omega-1-like [Asbolus verrucosus] | 205 | 2 |
| Gene.8229 | Pseudouridine-5'-phosphate glycosidase-like Protein [Tribolium castaneum] | 37 | 2 |
| Gene.8252 | T-complex protein 1 subunit eta [Anoplophora glabripennis] | 47 | 2 |
| Gene.8257 | 56 kDa early-staged encapsulation-inducing protein [Tenebrio molitor] | 136 | 2 |
| Gene.8288 | cofilin/actin-depolymerizing factor homolog [Anoplophora glabripennis] | 12 | 2 |
| Gene.8316 | PREDICTED: inorganic pyrophosphatase isoform X2 [Tribolium castaneum] | 86 | 2 |
| Gene.8319 | serrate RNA effector molecule homolog isoform X3 [Anoplophora glabripennis] | 132 | 2 |
| Gene.8322 | PREDICTED: uncharacterized protein LOC109595590 [Aethina tumida] | 46 | 2 |
| Gene.8333 | PREDICTED: 60S ribosomal protein L5 [Tribolium castaneum] | 41 | 2 |
| Gene.8333 | PREDICTED: 60S ribosomal protein L5 [Tribolium castaneum] | 178 | 2 |
| Gene.8382 | GST [Lygus lineolaris] | 87 | 2 |
| Gene.839 | PREDICTED: ATPase WRNIP1 [Tribolium castaneum] | 224 | 2 |
| Gene.8403 | PREDICTED: myosin heavy chain, non-muscle isoform X1 [Dendroctonus ponderosae] | 465 | 2 |
| Gene.8415 | Uncharacterized protein | 226 | 2 |
| Gene.8424 | PREDICTED: NAD/NADP-dependent betaine aldehyde dehydrogenase [Tribolium castaneum] | 96 | 2 |
| Gene.8427 | PREDICTED: EH domain-containing protein 3-like [Tribolium castaneum] | 367 | 2 |
| Gene.8427 | PREDICTED: EH domain-containing protein 3-like [Tribolium castaneum] | 426 | 2 |
| Gene.8430 | putative juvenile hormone acid methyltransferase, partial [Polypedilum vanderplanki] | 85 | 2 |
| Gene.8430 | putative juvenile hormone acid methyltransferase, partial [Polypedilum vanderplanki] | 9 | 2 |
| Gene.8450 | unknown [Dendroctonus ponderosae] | 82 | 2 |
| Gene.8474 | Uncharacterized protein | 831 | 2 |
| Gene.8474 | Uncharacterized protein | 199 | 2 |
| Gene.8478 | PREDICTED: uncharacterized protein LOC656540 [Tribolium castaneum] | 144 | 2 |
| Gene.8481 | PREDICTED: arginine kinase isoform X1 [Tribolium castaneum] | 276 | 2 |
| Gene.8481 | PREDICTED: arginine kinase isoform X1 [Tribolium castaneum] | 167 | 2 |
| Gene.8507 | moesin/ezrin/radixin homolog 1 isoform X2 [Anoplophora glabripennis] | 448 | 2 |
| Gene.8533 | PREDICTED: 60S acidic ribosomal protein P0 [Aethina tumida] | 270 | 2 |
| Gene.8583 | PREDICTED: heterogeneous nuclear ribonucleoprotein U-like protein 2 isoform X2 [Tribolium castaneum] | 276 | 2 |
| Gene.8593 | 26S proteasome regulatory subunit 6A-B [Anoplophora glabripennis] | 48 | 2 |
| Gene.8597 | PREDICTED: protein ABHD4-like isoform X2 [Aethina tumida] | 78 | 2 |
| Gene.8615 | uncharacterized protein BDFB_013993 [Asbolus verrucosus] | 319 | 2 |
| Gene.8615 | uncharacterized protein BDFB_013993 [Asbolus verrucosus] | 66 | 2 |
| Gene.8624 | eukaryotic translation initiation factor 3 subunit E [Anoplophora glabripennis] | 112 | 2 |
| Gene.864 | stress-induced-phosphoprotein 1-like [Asbolus verrucosus] | 191 | 2 |
| Gene.8673 | succinate dehydrogenase [ubiquinone] iron-sulfur subunit, mitochondrial, partial [Asbolus verrucosus] | 160 | 2 |
| Gene.8673 | succinate dehydrogenase [ubiquinone] iron-sulfur subunit, mitochondrial, partial [Asbolus verrucosus] | 168 | 2 |
| Gene.8707 | PREDICTED: heterogeneous nuclear ribonucleoprotein Q isoform X1 [Tribolium castaneum] | 129 | 2 |
| Gene.8707 | PREDICTED: heterogeneous nuclear ribonucleoprotein Q isoform X1 [Tribolium castaneum] | 231 | 2 |
| Gene.8716 | ATP-dependent RNA helicase p62, partial [Asbolus verrucosus] | 397 | 2 |
| Gene.8716 | ATP-dependent RNA helicase p62, partial [Asbolus verrucosus] | 278 | 2 |
| Gene.872 | mesencephalic astrocyte-derived neurotrophic factor homolog [Leptinotarsa decemlineata] | 45 | 2 |
| Gene.8727 | PREDICTED: proline dehydrogenase 1, mitochondrial isoform X2 [Dendroctonus ponderosae] | 202 | 2 |
| Gene.8751 | PREDICTED: keratin, type I cytoskeletal 9 [Tribolium castaneum] | 140 | 2 |
| Gene.8756 | uncharacterized protein Dvir_GJ16722 [Drosophila virilis] | 91 | 2 |
| Gene.8758 | PREDICTED: la protein homolog [Tribolium castaneum] | 214 | 2 |
| Gene.8758 | PREDICTED: la protein homolog [Tribolium castaneum] | 329 | 2 |
| Gene.8768 | PREDICTED: flavin reductase (NADPH) [Tribolium castaneum] | 123 | 2 |
| Gene.8801 | hypothetical protein WN51_04145 [Melipona quadrifasciata] | 252 | 2 |
| Gene.8801 | hypothetical protein WN51_04145 [Melipona quadrifasciata] | 279 | 2 |
| Gene.8815 | Uncharacterized protein | 176 | 2 |
| Gene.8815 | Uncharacterized protein | 113 | 2 |
| Gene.8819 | hydroxyacyl-coenzyme A dehydrogenase, mitochondrial [Anoplophora glabripennis] | 304 | 2 |
| Gene.8840 | signal recognition particle subunit SRP72 [Asbolus verrucosus] | 7 | 2 |
| Gene.8844 | ubiquitin carboxyl-terminal hydrolase 5 [Anoplophora glabripennis] | 158 | 2 |
| Gene.8853 | PREDICTED: selenide, water dikinase [Tribolium castaneum] | 369 | 2 |
| Gene.8853 | PREDICTED: selenide, water dikinase [Tribolium castaneum] | 406 | 2 |
| Gene.8856 | eukaryotic peptide chain release factor GTP-binding subunit ERF3A [Asbolus verrucosus] | 445 | 2 |
| Gene.8873 | PREDICTED: fumarate hydratase, mitochondrial [Tribolium castaneum] | 164 | 2 |
| Gene.8938 | 14-3-3 epsilon [Tenebrio molitor] | 69 | 2 |
| Gene.8952 | threonine--tRNA ligase, cytoplasmic isoform X1 [Anoplophora glabripennis] | 206 | 2 |
| Gene.8972 | peritrophic membrane chitin binding protein [Culex quinquefasciatus] | 44 | 2 |
| Gene.8989 | PREDICTED: aldehyde dehydrogenase, dimeric NADP-preferring-like [Aethina tumida] | 424 | 2 |
| Gene.8992 | sideroflexin-2 [Anoplophora glabripennis] | 68 | 2 |
| Gene.900 | Uncharacterized protein | 59 | 2 |
| Gene.901 | PREDICTED: plasma membrane calcium-transporting ATPase 2 isoform X2 [Tribolium castaneum] | 766 | 2 |
| Gene.9010 | PREDICTED: TATA-binding protein-associated factor 2N-like isoform X2 [Aethina tumida] | 71 | 2 |
| Gene.922 | acetyl-CoA acetyltransferase, mitochondrial, partial [Asbolus verrucosus] | 205 | 2 |
| Gene.924 | 26S proteasome non-ATPase regulatory subunit 11 [Asbolus verrucosus] | 50 | 2 |
| Gene.95 | Uncharacterized protein | 66 | 2 |
| Gene.956 | Perilipin domain containing protein [Asbolus verrucosus] | 87 | 2 |
| Gene.956 | Perilipin domain containing protein [Asbolus verrucosus] | 168 | 2 |
| Gene.960 | probable phosphoserine aminotransferase [Anoplophora glabripennis] | 54 | 2 |
| Gene.986 | PREDICTED: aldehyde dehydrogenase, dimeric NADP-preferring [Tribolium castaneum] | 259 | 2 |
| Gene.1032 | double-stranded RNA-binding protein Staufen -like protein 2 [Asbolus verrucosus] | 566 | 2 |
| Gene.1038 | Uncharacterized protein | 190 | 2 |
| Gene.1059 | PREDICTED: UBX domain-containing protein 4 [Tribolium castaneum] | 274 | 2 |
| Gene.1059 | PREDICTED: UBX domain-containing protein 4 [Tribolium castaneum] | 126 | 2 |
| Gene.106 | Uncharacterized protein | 86 | 2 |
| Gene.1073 | PREDICTED: succinate dehydrogenase [ubiquinone] flavoprotein subunit, mitochondrial isoform X1 [Tribolium castaneum] | 643 | 2 |
| Gene.1103 | S-adenosylmethionine synthase isoform X1 [Agrilus planipennis] | 87 | 2 |
| Gene.1103 | S-adenosylmethionine synthase isoform X1 [Agrilus planipennis] | 169 | 2 |
| Gene.1103 | S-adenosylmethionine synthase isoform X1 [Agrilus planipennis] | 368 | 2 |
| Gene.1196 | pyrroline-5-carboxylate reductase [Leptinotarsa decemlineata] | 32 | 2 |
| Gene.1219 | 46 kDa FK506-binding nuclear protein [Anoplophora glabripennis] | 18 | 2 |
| Gene.1223 | eukaryotic translation initiation factor 3 subunit M [Anoplophora glabripennis] | 292 | 2 |
| Gene.1223 | eukaryotic translation initiation factor 3 subunit M [Anoplophora glabripennis] | 54 | 2 |
| Gene.1224 | PREDICTED: multidrug resistance protein 1 [Tribolium castaneum] | 396 | 2 |
| Gene.1264 | PREDICTED: protein disulfide-isomerase [Tribolium castaneum] | 339 | 2 |
| Gene.1264 | PREDICTED: protein disulfide-isomerase [Tribolium castaneum] | 83 | 2 |
| Gene.1295 | PREDICTED: lamin Dm0 isoform X1 [Aethina tumida] | 492 | 2 |
| Gene.1295 | PREDICTED: lamin Dm0 isoform X1 [Aethina tumida] | 240 | 2 |
| Gene.1305 | hypothetical protein AMK59_6936 [Oryctes borbonicus] | 190 | 2 |
| Gene.1392 | hypothetical protein WN55_01867 [Dufourea novaeangliae] | 238 | 2 |
| Gene.1392 | hypothetical protein WN55_01867 [Dufourea novaeangliae] | 210 | 2 |
| Gene.1403 | Protein LSM14 homolog B-like Protein [Tribolium castaneum] | 361 | 2 |
| Gene.1428 | PREDICTED: prostaglandin E synthase 3 [Nicrophorus vespilloides] | 44 | 2 |
| Gene.1434 | PREDICTED: PDZ domain-containing protein GIPC3 [Tribolium castaneum] | 181 | 2 |
| Gene.1478 | PREDICTED: fasciclin-1 [Aethina tumida] | 229 | 2 |
| Gene.1482 | flotillin-2 [Onthophagus taurus] | 176 | 2 |
| Gene.1486 | Prefoldin domain containing protein [Asbolus verrucosus] | 110 | 2 |
| Gene.150 | PREDICTED: eukaryotic translation initiation factor 5A [Dendroctonus ponderosae] | 87 | 2 |
| Gene.1530 | PREDICTED: farnesyl pyrophosphate synthase-like [Atta cephalotes] | 70 | 2 |
| Gene.1532 | PREDICTED: glyoxalase domain-containing protein 4 [Tribolium castaneum] | 157 | 2 |
| Gene.1532 | PREDICTED: glyoxalase domain-containing protein 4 [Tribolium castaneum] | 78 | 2 |
| Gene.1534 | PREDICTED: T-complex protein 1 subunit gamma [Tribolium castaneum] | 93 | 2 |
| Gene.1542 | PREDICTED: ATP synthase subunit alpha, mitochondrial [Tribolium castaneum] | 124 | 2 |
| Gene.1542 | PREDICTED: ATP synthase subunit alpha, mitochondrial [Tribolium castaneum] | 259 | 2 |
| Gene.1588 | cullin-associated NEDD8-dissociated protein 1 [Anoplophora glabripennis] | 626 | 2 |
| Gene.1679 | Uncharacterized protein | 104 | 2 |
| Gene.1679 | Uncharacterized protein | 139 | 2 |
| Gene.1684 | juvenile hormone acid O-methyltransferase [Ceratitis capitata] | 30 | 2 |
| Gene.1695 | PREDICTED: LIM domain and actin-binding protein 1-like isoform X16 [Aethina tumida] | 614 | 2 |
| Gene.1723 | uncharacterized protein LOC108912517 [Anoplophora glabripennis] | 69 | 2 |
| Gene.1745 | PREDICTED: probable medium-chain specific acyl-CoA dehydrogenase, mitochondrial [Tribolium castaneum] | 389 | 2 |
| Gene.175 | PREDICTED: probable citrate synthase 2, mitochondrial [Tribolium castaneum] | 42 | 2 |
| Gene.1768 | PREDICTED: translation elongation factor 2 [Aethina tumida] | 310 | 2 |
| Gene.1768 | PREDICTED: translation elongation factor 2 [Aethina tumida] | 406 | 2 |
| Gene.179 | dipeptidyl peptidase 3 [Anoplophora glabripennis] | 209 | 2 |
| Gene.179 | dipeptidyl peptidase 3 [Anoplophora glabripennis] | 219 | 2 |
| Gene.179 | dipeptidyl peptidase 3 [Anoplophora glabripennis] | 80 | 2 |
| Gene.1792 | PREDICTED: zinc finger RNA-binding protein isoform X2 [Aethina tumida] | 579 | 2 |
| Gene.18 | PREDICTED: estradiol 17-beta-dehydrogenase 8 isoform X1 [Aethina tumida] | 209 | 2 |
| Gene.18 | PREDICTED: estradiol 17-beta-dehydrogenase 8 isoform X1 [Aethina tumida] | 173 | 2 |
| Gene.182 | cytosolic non-specific dipeptidase [Anoplophora glabripennis] | 376 | 2 |
| Gene.1862 | PREDICTED: trifunctional enzyme subunit beta, mitochondrial [Tribolium castaneum] | 261 | 2 |
| Gene.1862 | PREDICTED: trifunctional enzyme subunit beta, mitochondrial [Tribolium castaneum] | 285 | 2 |
| Gene.1891 | hypothetical protein AMK59_3112 [Oryctes borbonicus] | 132 | 2 |
| Gene.1958 | PREDICTED: fimbrin isoform X1 [Tribolium castaneum] | 80 | 2 |
| Gene.1982 | Tetratricopeptide repeat-containing protein, partial [Oryctes borbonicus] | 1131 | 2 |
| Gene.1991 | PREDICTED: LOW QUALITY PROTEIN: multidrug resistance-associated protein 4-like [Aethina tumida] | 86 | 2 |
| Gene.2016 | PREDICTED: 40S ribosomal protein S4 isoform X1 [Aethina tumida] | 211 | 2 |
| Gene.2017 | ATP synthase subunit O, mitochondrial [Asbolus verrucosus] | 116 | 2 |
| Gene.204 | ribosomal protein L28, partial [Harmonia axyridis] | 117 | 2 |
| Gene.2079 | PREDICTED: adenylyl cyclase-associated protein 1 isoform X2 [Tribolium castaneum] | 211 | 2 |
| Gene.2113 | 60S ribosomal protein L31 [Blattella germanica] | 28 | 2 |
| Gene.2114 | PREDICTED: aminoacylase-1 [Tribolium castaneum] | 67 | 2 |
| Gene.2124 | PREDICTED: sorbitol dehydrogenase [Tribolium castaneum] | 333 | 2 |
| Gene.2124 | PREDICTED: sorbitol dehydrogenase [Tribolium castaneum] | 103 | 2 |
| Gene.2134 | PREDICTED: 40S ribosomal protein S7 [Tribolium castaneum] | 165 | 2 |
| Gene.2134 | PREDICTED: 40S ribosomal protein S7 [Tribolium castaneum] | 156 | 2 |
| Gene.2134 | PREDICTED: 40S ribosomal protein S7 [Tribolium castaneum] | 46 | 2 |
| Gene.2161 | Calcium-binding mitochondrial carrier protein Aralar1-like Protein [Tribolium castaneum] | 81 | 2 |
| Gene.2176 | UDP-glucuronosyltransferase 1-9-like isoform X2 [Leptinotarsa decemlineata] | 101 | 2 |
| Gene.2192 | small glutamine-rich tetratricopeptide repeat-containing protein alpha-like [Pseudomyrmex gracilis] | 171 | 2 |
| Gene.2223 | PREDICTED: nodal modulator 1 [Tribolium castaneum] | 41 | 2 |
| Gene.2236 | Glutamate dehydrogenase, mitochondrial-like Protein [Tribolium castaneum] | 454 | 2 |
| Gene.2365 | PREDICTED: maternal protein tudor isoform X1 [Tribolium castaneum] | 1257 | 2 |
| Gene.2365 | PREDICTED: maternal protein tudor isoform X1 [Tribolium castaneum] | 955 | 2 |
| Gene.2368 | PREDICTED: filamin-A isoform X6 [Tribolium castaneum] | 1428 | 2 |
| Gene.2373 | uncharacterized protein LOC108904004 [Anoplophora glabripennis] | 363 | 2 |
| Gene.2375 | glyceraldehyde-3-phosphate, partial [Harmonia axyridis] | 94 | 2 |
| Gene.2505 | PREDICTED: 1-acyl-sn-glycerol-3-phosphate acyltransferase delta [Tribolium castaneum] | 281 | 2 |
| Gene.2525 | Protein anon-37Cs-like Protein [Tribolium castaneum] | 287 | 2 |
| Gene.2533 | PREDICTED: putative aldehyde dehydrogenase family 7 member A1 homolog [Aethina tumida] | 339 | 2 |
| Gene.2533 | PREDICTED: putative aldehyde dehydrogenase family 7 member A1 homolog [Aethina tumida] | 264 | 2 |
| Gene.2562 | hypothetical protein AMK59_6989 [Oryctes borbonicus] | 28 | 2 |
| Gene.2565 | PREDICTED: protein hu-li tai shao isoform X2 [Tribolium castaneum] | 385 | 2 |
| Gene.2568 | coatomer subunit delta [Anoplophora glabripennis] | 363 | 2 |
| Gene.2587 | Uncharacterized protein | 203 | 2 |
| Gene.2605 | UV excision repair protein RAD23 homolog B [Agrilus planipennis] | 52 | 2 |
| Gene.2644 | PREDICTED: uncharacterized protein LOC103313592 [Tribolium castaneum] | 222 | 2 |
| Gene.2703 | PREDICTED: very-long-chain 3-oxoacyl-CoA reductase [Aethina tumida] | 84 | 2 |
| Gene.2749 | PREDICTED: aldehyde dehydrogenase, mitochondrial [Tribolium castaneum] | 81 | 2 |
| Gene.2767 | ribosomal protein L10, partial [Harmonia axyridis] | 130 | 2 |
| Gene.2767 | ribosomal protein L10, partial [Harmonia axyridis] | 28 | 2 |
| Gene.2814 | B-cell lymphoma/leukemia 11A isoform X1 [Anoplophora glabripennis] | 4 | 2 |
| Gene.2830 | PREDICTED: LOW QUALITY PROTEIN: clathrin heavy chain [Aethina tumida] | 644 | 2 |
| Gene.2847 | microtubule-actin cross-linking factor 1 isoform X7 [Anoplophora glabripennis] | 583 | 2 |
| Gene.2884 | PREDICTED: cathepsin L1 [Tribolium castaneum] | 120 | 2 |
| Gene.2913 | PREDICTED: glutaryl-CoA dehydrogenase, mitochondrial [Nicrophorus vespilloides] | 188 | 2 |
| Gene.2918 | plasminogen activator inhibitor 1 RNA-binding protein isoform X3 [Anoplophora glabripennis] | 391 | 2 |
| Gene.2933 | isocitrate dehydrogenase [NADP], mitochondrial-like [Onthophagus taurus] | 204 | 2 |
| Gene.2958 | alpha actinin [Coleomegilla maculata] | 347 | 2 |
| Gene.2958 | alpha actinin [Coleomegilla maculata] | 106 | 2 |
| Gene.2958 | alpha actinin [Coleomegilla maculata] | 405 | 2 |
| Gene.2970 | apolipophorin, partial [Asbolus verrucosus] | 219 | 2 |
| Gene.2997 | PREDICTED: pyruvate kinase-like isoform X3 [Dendroctonus ponderosae] | 160 | 2 |
| Gene.3030 | PREDICTED: actin-interacting protein 1 isoform X2 [Tribolium castaneum] | 62 | 2 |
| Gene.310 | 60S ribosomal protein L10a [Monochamus alternatus] | 118 | 2 |
| Gene.312 | heterogeneous nuclear ribonucleoprotein H [Asbolus verrucosus] | 87 | 2 |
| Gene.316 | heat shock 70 kDa protein cognate 5-like protein [Epicauta chinensis] | 101 | 2 |
| Gene.3177 | Hemocyanin C and/or Tyrosinase domain containing protein [Asbolus verrucosus] | 189 | 2 |
| Gene.3177 | Hemocyanin C and/or Tyrosinase domain containing protein [Asbolus verrucosus] | 514 | 2 |
| Gene.3223 | Aldose reductase-like Protein [Tribolium castaneum] | 56 | 2 |
| Gene.326 | PREDICTED: uncharacterized protein LOC660521 [Tribolium castaneum] | 89 | 2 |
| Gene.33 | PREDICTED: eukaryotic translation initiation factor 3 subunit I [Tribolium castaneum] | 293 | 2 |
| Gene.331 | PREDICTED: ferritin subunit [Aethina tumida] | 180 | 2 |
| Gene.3368 | PREDICTED: cytochrome P450 4C1 [Tribolium castaneum] | 278 | 2 |
| Gene.3380 | PREDICTED: ribonucleoside-diphosphate reductase large subunit-like [Aethina tumida] | 96 | 2 |
| Gene.34 | PREDICTED: short/branched chain specific acyl-CoA dehydrogenase, mitochondrial [Aethina tumida] | 66 | 2 |
| Gene.3482 | V-type proton ATPase catalytic subunit A [Leptinotarsa decemlineata] | 217 | 2 |
| Gene.3489 | PREDICTED: acetyl-CoA carboxylase isoform X1 [Tribolium castaneum] | 450 | 2 |
| Gene.3489 | PREDICTED: acetyl-CoA carboxylase isoform X1 [Tribolium castaneum] | 1394 | 2 |
| Gene.3489 | PREDICTED: acetyl-CoA carboxylase isoform X1 [Tribolium castaneum] | 1493 | 2 |
| Gene.3489 | PREDICTED: acetyl-CoA carboxylase isoform X1 [Tribolium castaneum] | 2287 | 2 |
| Gene.3505 | cytochrome P450 monooxygenase CYP6BQ37 [Tenebrio molitor] | 281 | 2 |
| Gene.351 | 6-phosphogluconolactonase [Asbolus verrucosus] | 60 | 2 |
| Gene.3518 | vitellogenin 2 [Harmonia axyridis] | 445 | 2 |
| Gene.3518 | vitellogenin 2 [Harmonia axyridis] | 351 | 2 |
| Gene.3518 | vitellogenin 2 [Harmonia axyridis] | 1231 | 2 |
| Gene.354 | saccharopine dehydrogenase-like [Asbolus verrucosus] | 427 | 2 |
| Gene.3558 | PREDICTED: probable aconitate hydratase, mitochondrial [Tribolium castaneum] | 75 | 2 |
| Gene.3558 | PREDICTED: probable aconitate hydratase, mitochondrial [Tribolium castaneum] | 751 | 2 |
| Gene.3558 | PREDICTED: probable aconitate hydratase, mitochondrial [Tribolium castaneum] | 145 | 2 |
| Gene.3558 | PREDICTED: probable aconitate hydratase, mitochondrial [Tribolium castaneum] | 528 | 2 |
| Gene.3571 | cytochrome P450, partial [Cryptolaemus montrouzieri] | 154 | 2 |
| Gene.3583 | 4-coumarate--CoA ligase-like [Leptinotarsa decemlineata] | 215 | 2 |
| Gene.3601 | PREDICTED: sulfotransferase 1A1 [Nicrophorus vespilloides] | 145 | 2 |
| Gene.3601 | PREDICTED: sulfotransferase 1A1 [Nicrophorus vespilloides] | 279 | 2 |
| Gene.3608 | chitinase 3 precursor [Tribolium castaneum] | 89 | 2 |
| Gene.3698 | Uncharacterized protein | 46 | 2 |
| Gene.3715 | tropomyosin-1, isoforms 33/34 isoform X1 [Leptinotarsa decemlineata] | 168 | 2 |
| Gene.3730 | PREDICTED: protein amalgam isoform X1 [Tribolium castaneum] | 210 | 2 |
| Gene.3747 | piwi-like protein Ago3 [Anoplophora glabripennis] | 470 | 2 |
| Gene.3770 | hypothetical protein D910_09337 [Dendroctonus ponderosae] | 74 | 2 |
| Gene.379 | PREDICTED: uncharacterized protein LOC659867 [Tribolium castaneum] | 163 | 2 |
| Gene.3790 | PREDICTED: 60S ribosomal protein L3 [Tribolium castaneum] | 366 | 2 |
| Gene.3838 | PREDICTED: pyruvate carboxylase, mitochondrial isoform X3 [Tribolium castaneum] | 237 | 2 |
| Gene.3838 | PREDICTED: pyruvate carboxylase, mitochondrial isoform X3 [Tribolium castaneum] | 922 | 2 |
| Gene.3901 | aldose 1-epimerase-like [Leptinotarsa decemlineata] | 298 | 2 |
| Gene.391 | ubiquitin carboxyl-terminal hydrolase [Asbolus verrucosus] | 19 | 2 |
| Gene.3911 | PREDICTED: LOW QUALITY PROTEIN: heat shock 70 kDa protein 4 [Aethina tumida] | 194 | 2 |
| Gene.3912 | PREDICTED: LOW QUALITY PROTEIN: heat shock 70 kDa protein 4 [Aethina tumida] | 188 | 2 |
| Gene.3947 | PREDICTED: delta-1-pyrroline-5-carboxylate synthase [Tribolium castaneum] | 437 | 2 |
| Gene.3947 | PREDICTED: delta-1-pyrroline-5-carboxylate synthase [Tribolium castaneum] | 356 | 2 |
| Gene.3973 | PREDICTED: thioredoxin reductase 1, mitochondrial isoform X6 [Tribolium castaneum] | 68 | 2 |
| Gene.4011 | fatty acid synthase [Coccinella septempunctata] | 937 | 2 |
| Gene.4011 | fatty acid synthase [Coccinella septempunctata] | 1448 | 2 |
| Gene.4017 | Glutamyl aminopeptidase-like Protein [Tribolium castaneum] | 164 | 2 |
| Gene.4067 | PREDICTED: programmed cell death 6-interacting protein [Tribolium castaneum] | 314 | 2 |
| Gene.4078 | uncharacterized protein LOC108917852 [Anoplophora glabripennis] | 532 | 2 |
| Gene.4081 | Ribosomal L19e domain containing protein [Asbolus verrucosus] | 150 | 2 |
| Gene.4113 | succinyl-CoA:3-ketoacid coenzyme A transferase 1, mitochondrial, partial [Asbolus verrucosus] | 114 | 2 |
| Gene.4132 | phosphoribosylformylglycinamidine synthase [Anoplophora glabripennis] | 1054 | 2 |
| Gene.4149 | PREDICTED: LOW QUALITY PROTEIN: 26S proteasome non-ATPase regulatory subunit 1-like [Aethina tumida] | 287 | 2 |
| Gene.4221 | Prefoldin 2 domain containing protein [Asbolus verrucosus] | 36 | 2 |
| Gene.4244 | PREDICTED: protein NipSnap-like [Aethina tumida] | 168 | 2 |
| Gene.426 | PREDICTED: LOW QUALITY PROTEIN: amidophosphoribosyltransferase-like [Aethina tumida] | 117 | 2 |
| Gene.4262 | sarcosine dehydrogenase, mitochondrial [Asbolus verrucosus] | 287 | 2 |
| Gene.4262 | sarcosine dehydrogenase, mitochondrial [Asbolus verrucosus] | 507 | 2 |
| Gene.4272 | PREDICTED: LOW QUALITY PROTEIN: NADP-dependent malic enzyme-like [Aethina tumida] | 13 | 2 |
| Gene.4272 | PREDICTED: LOW QUALITY PROTEIN: NADP-dependent malic enzyme-like [Aethina tumida] | 218 | 2 |
| Gene.4285 | PREDICTED: LOW QUALITY PROTEIN: pyruvate kinase-like [Aethina tumida] | 376 | 2 |
| Gene.429 | 40S ribosomal protein S16 [Anoplophora glabripennis] | 113 | 2 |
| Gene.4299 | electron transfer flavoprotein-ubiquinone oxidoreductase, mitochondrial [Anoplophora glabripennis] | 80 | 2 |
| Gene.4314 | UDP-glucuronosyltransferase 2C1-like, partial [Asbolus verrucosus] | 270 | 2 |
| Gene.4335 | alpha-aminoadipic semialdehyde synthase, mitochondrial [Anoplophora glabripennis] | 153 | 2 |
| Gene.4384 | Twitchin-like Protein [Tribolium castaneum] | 1315 | 2 |
| Gene.4396 | PREDICTED: 2-hydroxyacylsphingosine 1-beta-galactosyltransferase-like [Tribolium castaneum] | 60 | 2 |
| Gene.4417 | glycogen phosphorylase [Harmonia axyridis] | 183 | 2 |
| Gene.4419 | glycogen phosphorylase [Harmonia axyridis] | 42 | 2 |
| Gene.4531 | PREDICTED: glutamate synthase 1 [NADH], chloroplastic isoform X2 [Tribolium castaneum] | 443 | 2 |
| Gene.4576 | PREDICTED: NADH dehydrogenase [ubiquinone] iron-sulfur protein 3, mitochondrial [Tribolium castaneum] | 52 | 2 |
| Gene.4580 | PREDICTED: trifunctional enzyme subunit alpha, mitochondrial [Tribolium castaneum] | 94 | 2 |
| Gene.4583 | PREDICTED: ATP-citrate synthase isoform X1 [Tribolium castaneum] | 83 | 2 |
| Gene.4593 | spectrin beta chain isoform X1 [Anoplophora glabripennis] | 1394 | 2 |
| Gene.4593 | spectrin beta chain isoform X1 [Anoplophora glabripennis] | 297 | 2 |
| Gene.4609 | mucin-2 isoform X3 [Anoplophora glabripennis] | 584 | 2 |
| Gene.4653 | Uncharacterized protein | 246 | 2 |
| Gene.4752 | hexamerin 3 precursor [Tribolium castaneum] | 656 | 2 |
| Gene.4764 | fatty acid synthase [Coccinella septempunctata] | 208 | 2 |
| Gene.4867 | PREDICTED: spectrin alpha chain isoform X4 [Tribolium castaneum] | 445 | 2 |
| Gene.4867 | PREDICTED: spectrin alpha chain isoform X4 [Tribolium castaneum] | 181 | 2 |
| Gene.4887 | PREDICTED: lysosomal alpha-mannosidase isoform X2 [Nicrophorus vespilloides] | 202 | 2 |
| Gene.4926 | translocon-associated protein subunit beta [Leptinotarsa decemlineata] | 37 | 2 |
| Gene.4934 | apolipophorins [Anoplophora glabripennis] | 982 | 2 |
| Gene.4934 | apolipophorins [Anoplophora glabripennis] | 1190 | 2 |
| Gene.4934 | apolipophorins [Anoplophora glabripennis] | 1158 | 2 |
| Gene.4973 | PREDICTED: myosin heavy chain, muscle isoform X18 [Tribolium castaneum] | 1250 | 2 |
| Gene.4973 | PREDICTED: myosin heavy chain, muscle isoform X18 [Tribolium castaneum] | 1212 | 2 |
| Gene.4973 | PREDICTED: myosin heavy chain, muscle isoform X18 [Tribolium castaneum] | 257 | 2 |
| Gene.500 | alanine aminotransferase 1 [Anoplophora glabripennis] | 488 | 2 |
| Gene.500 | alanine aminotransferase 1 [Anoplophora glabripennis] | 442 | 2 |
| Gene.5015 | multidrug resistance-associated protein 4 [Anoplophora glabripennis] | 1070 | 2 |
| Gene.5030 | cytoplasmic aconitate hydratase-like isoform X1 [Anoplophora glabripennis] | 164 | 2 |
| Gene.5108 | microsomal triglyceride transfer protein large subunit [Anoplophora glabripennis] | 462 | 2 |
| Gene.526 | signal recognition particle subunit SRP68 [Anoplophora glabripennis] | 405 | 2 |
| Gene.5354 | NADH dehydrogenase [ubiquinone] 1 beta subcomplex subunit 5, mitochondrial [Anoplophora glabripennis] | 154 | 2 |
| Gene.5354 | NADH dehydrogenase [ubiquinone] 1 beta subcomplex subunit 5, mitochondrial [Anoplophora glabripennis] | 134 | 2 |
| Gene.5391 | hypothetical protein AMK59_4850 [Oryctes borbonicus] | 149 | 2 |
| Gene.5391 | hypothetical protein AMK59_4850 [Oryctes borbonicus] | 87 | 2 |
| Gene.5402 | PREDICTED: transmembrane emp24 domain-containing protein eca-like [Nicrophorus vespilloides] | 217 | 2 |
| Gene.5478 | 60S ribosomal protein L7a-like [Leptinotarsa decemlineata] | 28 | 2 |
| Gene.548 | clavesin-1 isoform X1 [Agrilus planipennis] | 77 | 2 |
| Gene.5511 | PREDICTED: nucleoplasmin-like protein [Aethina tumida] | 25 | 2 |
| Gene.5542 | catalase [Onthophagus taurus] | 115 | 2 |
| Gene.5542 | catalase [Onthophagus taurus] | 506 | 2 |
| Gene.5580 | proteasome subunit alpha type-4 [Anoplophora glabripennis] | 210 | 2 |
| Gene.5580 | proteasome subunit alpha type-4 [Anoplophora glabripennis] | 195 | 2 |
| Gene.5580 | proteasome subunit alpha type-4 [Anoplophora glabripennis] | 54 | 2 |
| Gene.5602 | 60S ribosomal protein L13 [Anoplophora glabripennis] | 52 | 2 |
| Gene.5604 | ribosomal protein L35 [Chrysomela tremula] | 35 | 2 |
| Gene.5619 | CLUMA_CG011895, isoform A [Clunio marinus] | 70 | 2 |
| Gene.5622 | probable N-acetyltransferase san [Anoplophora glabripennis] | 37 | 2 |
| Gene.5625 | ribosomal protein L32e [Hister sp. APV-2005] | 19 | 2 |
| Gene.563 | PREDICTED: importin-5 [Tribolium castaneum] | 622 | 2 |
| Gene.5636 | ras-related protein Rab-11A [Anoplophora glabripennis] | 140 | 2 |
| Gene.5705 | PREDICTED: LOW QUALITY PROTEIN: uncharacterized protein LOC109596860 [Aethina tumida] | 602 | 2 |
| Gene.5733 | PREDICTED: prohibitin-2 isoform X2 [Tribolium castaneum] | 217 | 2 |
| Gene.5741 | 26S proteasome non-ATPase regulatory subunit 8 [Anoplophora glabripennis] | 196 | 2 |
| Gene.5770 | PREDICTED: 26S protease regulatory subunit 10B [Nicrophorus vespilloides] | 209 | 2 |
| Gene.5801 | SUMO-activating enzyme subunit 2 [Anoplophora glabripennis] | 537 | 2 |
| Gene.5825 | PREDICTED: bis(5'-nucleosyl)-tetraphosphatase [asymmetrical]-like [Dendroctonus ponderosae] | 61 | 2 |
| Gene.5862 | V-type proton ATPase subunit F, partial [Asbolus verrucosus] | 57 | 2 |
| Gene.5910 | PREDICTED: NADH dehydrogenase [ubiquinone] 1 alpha subcomplex subunit 10, mitochondrial [Tribolium castaneum] | 55 | 2 |
| Gene.5928 | PREDICTED: 40S ribosomal protein S3a [Aethina tumida] | 37 | 2 |
| Gene.5932 | PREDICTED: LOW QUALITY PROTEIN: probable 39S ribosomal protein L24, mitochondrial [Aethina tumida] | 204 | 2 |
| Gene.5969 | Ribose-phosphate pyrophosphokinase 1-like Protein [Tribolium castaneum] | 146 | 2 |
| Gene.5973 | ribosomal protein S13, partial [Harmonia axyridis] | 132 | 2 |
| Gene.5992 | PREDICTED: GTP:AMP phosphotransferase AK3, mitochondrial [Tribolium castaneum] | 211 | 2 |
| Gene.5994 | PREDICTED: LOW QUALITY PROTEIN: histidine triad nucleotide-binding protein 1-like [Aethina tumida] | 100 | 2 |
| Gene.6019 | PREDICTED: membrane-associated progesterone receptor component 1 [Tribolium castaneum] | 117 | 2 |
| Gene.6024 | peroxiredoxin 1-like [Leptinotarsa decemlineata] | 189 | 2 |
| Gene.6050 | protein RER1 isoform X1 [Anoplophora glabripennis] | 13 | 2 |
| Gene.6073 | PREDICTED: cold shock domain-containing protein CG9705 [Aethina tumida] | 109 | 2 |
| Gene.6131 | ruvB-like 2 [Asbolus verrucosus] | 200 | 2 |
| Gene.6131 | ruvB-like 2 [Asbolus verrucosus] | 364 | 2 |
| Gene.6186 | succinate dehydrogenase cytochrome b560 subunit, mitochondrial [Anoplophora glabripennis] | 63 | 2 |
| Gene.6192 | PREDICTED: ecdysteroid-regulated 16 kDa protein [Tribolium castaneum] | 91 | 2 |
| Gene.6194 | PREDICTED: acidic leucine-rich nuclear phosphoprotein 32 family member A [Tribolium castaneum] | 18 | 2 |
| Gene.6196 | PREDICTED: protein l(2)37Cc [Dendroctonus ponderosae] | 203 | 2 |
| Gene.6197 | ribosomal protein L11e [Curculio glandium] | 61 | 2 |
| Gene.6197 | ribosomal protein L11e [Curculio glandium] | 87 | 2 |
| Gene.624 | PREDICTED: staphylococcal nuclease domain-containing protein 1 [Tribolium castaneum] | 306 | 2 |
| Gene.624 | PREDICTED: staphylococcal nuclease domain-containing protein 1 [Tribolium castaneum] | 581 | 2 |
| Gene.624 | PREDICTED: staphylococcal nuclease domain-containing protein 1 [Tribolium castaneum] | 362 | 2 |
| Gene.6252 | NADH dehydrogenase [ubiquinone] 1 beta subcomplex subunit 4 [Leptinotarsa decemlineata] | 32 | 2 |
| Gene.6258 | muscle-specific protein 20 [Leptinotarsa decemlineata] | 129 | 2 |
| Gene.6284 | NifU N domain containing protein [Asbolus verrucosus] | 120 | 2 |
| Gene.6325 | uncharacterized protein Dvir_GJ22112 [Drosophila virilis] | 13 | 2 |
| Gene.6326 | RRM 1 domain containing protein [Asbolus verrucosus] | 110 | 2 |
| Gene.6339 | PREDICTED: nucleoside diphosphate kinase isoform X1 [Tribolium castaneum] | 39 | 2 |
| Gene.6375 | proliferation-associated protein 2G4 [Anoplophora glabripennis] | 18 | 2 |
| Gene.6380 | PREDICTED: 3'(2'),5'-bisphosphate nucleotidase 1 [Nicrophorus vespilloides] | 40 | 2 |
| Gene.6439 | PREDICTED: protein DEK isoform X3 [Tribolium castaneum] | 228 | 2 |
| Gene.6439 | PREDICTED: protein DEK isoform X3 [Tribolium castaneum] | 242 | 2 |
| Gene.6474 | hypothetical protein AMK59_6986 [Oryctes borbonicus] | 142 | 2 |
| Gene.6508 | PREDICTED: acyl carrier protein, mitochondrial isoform X1 [Dendroctonus ponderosae] | 128 | 2 |
| Gene.6543 | PREDICTED: phenylalanine--tRNA ligase alpha subunit [Aethina tumida] | 122 | 2 |
| Gene.6561 | protein phosphatase 2, regulatory subunit A, alpha isoform [Tribolium castaneum] | 109 | 2 |
| Gene.6561 | protein phosphatase 2, regulatory subunit A, alpha isoform [Tribolium castaneum] | 307 | 2 |
| Gene.6573 | PREDICTED: 40S ribosomal protein S15 [Tribolium castaneum] | 78 | 2 |
| Gene.6597 | heat shock protein 21.53 [Harmonia axyridis] | 149 | 2 |
| Gene.665 | PREDICTED: 1,4-alpha-glucan-branching enzyme [Aethina tumida] | 515 | 2 |
| Gene.6654 | jupiter microtubule associated homolog 1-like [Leptinotarsa decemlineata] | 84 | 2 |
| Gene.6679 | Isocitrate dehydrogenase [NADP] [Operophtera brumata] | 239 | 2 |
| Gene.6679 | Isocitrate dehydrogenase [NADP] [Operophtera brumata] | 139 | 2 |
| Gene.6700 | lactoylglutathione lyase [Anoplophora glabripennis] | 141 | 2 |
| Gene.6726 | PREDICTED: NSFL1 cofactor p47 [Nicrophorus vespilloides] | 94 | 2 |
| Gene.6752 | mitochondrial fission 1 protein [Anoplophora glabripennis] | 46 | 2 |
| Gene.6774 | PREDICTED: basigin isoform X1 [Nicrophorus vespilloides] | 128 | 2 |
| Gene.6774 | PREDICTED: basigin isoform X1 [Nicrophorus vespilloides] | 214 | 2 |
| Gene.6791 | thymosin beta isoform X2 [Leptinotarsa decemlineata] | 41 | 2 |
| Gene.6795 | Ubiquinol-cytochrome-c reductase complex assembly factor 1-like Protein [Tribolium castaneum] | 72 | 2 |
| Gene.68 | PREDICTED: prolyl endopeptidase isoform X1 [Tribolium castaneum] | 196 | 2 |
| Gene.6805 | peptidyl-prolyl cis-trans isomerase [Leptinotarsa decemlineata] | 98 | 2 |
| Gene.6805 | peptidyl-prolyl cis-trans isomerase [Leptinotarsa decemlineata] | 136 | 2 |
| Gene.6894 | PREDICTED: adenylate kinase [Tribolium castaneum] | 70 | 2 |
| Gene.6895 | PREDICTED: acyl-CoA-binding domain-containing protein 5 [Aethina tumida] | 237 | 2 |
| Gene.6918 | ECH domain containing protein [Asbolus verrucosus] | 166 | 2 |
| Gene.6928 | PREDICTED: transitional endoplasmic reticulum ATPase TER94 [Tribolium castaneum] | 64 | 2 |
| Gene.6928 | PREDICTED: transitional endoplasmic reticulum ATPase TER94 [Tribolium castaneum] | 700 | 2 |
| Gene.6977 | AFG3-like protein 2 [Anoplophora glabripennis] | 598 | 2 |
| Gene.6981 | PREDICTED: receptor expression-enhancing protein 5-like [Aethina tumida] | 18 | 2 |
| Gene.6981 | PREDICTED: receptor expression-enhancing protein 5-like [Aethina tumida] | 160 | 2 |
| Gene.6996 | PREDICTED: LOW QUALITY PROTEIN: short-chain dehydrogenase/reductase family 16C member 6-like [Aethina tumida] | 270 | 2 |
| Gene.7019 | PREDICTED: ultraviolet-B receptor UVR8 [Tribolium castaneum] | 314 | 2 |
| Gene.7021 | PREDICTED: prefoldin subunit 6 [Tribolium castaneum] | 8 | 2 |
| Gene.704 | polyadenylate-binding protein 4-like [Anoplophora glabripennis] | 139 | 2 |
| Gene.7092 | hypothetical protein AMK59_7915, partial [Oryctes borbonicus] | 32 | 2 |
| Gene.7096 | PREDICTED: S-formylglutathione hydrolase [Aethina tumida] | 142 | 2 |
| Gene.7108 | unknown [Dendroctonus ponderosae] | 392 | 2 |
| Gene.7117 | T-complex protein 1 subunit alpha [Anoplophora glabripennis] | 469 | 2 |
| Gene.7125 | Ribosomal L7Ae domain containing protein [Asbolus verrucosus] | 107 | 2 |
| Gene.7131 | ras-related protein Rab-32 isoform X1 [Anoplophora glabripennis] | 196 | 2 |
| Gene.7166 | PREDICTED: rab GDP dissociation inhibitor alpha [Aethina tumida] | 102 | 2 |
| Gene.7166 | PREDICTED: rab GDP dissociation inhibitor alpha [Aethina tumida] | 264 | 2 |
| Gene.7216 | PREDICTED: microtubule-associated protein RP/EB family member 1 isoform X2 [Tribolium castaneum] | 64 | 2 |
| Gene.7218 | MICOS complex subunit Mic60 isoform X1 [Leptinotarsa decemlineata] | 164 | 2 |
| Gene.7236 | Uncharacterized protein | 49 | 2 |
| Gene.7264 | heat shock protein 90, partial [Harmonia axyridis] | 257 | 2 |
| Gene.7264 | heat shock protein 90, partial [Harmonia axyridis] | 422 | 2 |
| Gene.7276 | protein disulfide-isomerase A3 [Asbolus verrucosus] | 188 | 2 |
| Gene.7326 | serine hydroxymethyltransferase, cytosolic isoform X1 [Anoplophora glabripennis] | 297 | 2 |
| Gene.7366 | PREDICTED: V-type proton ATPase subunit G [Nicrophorus vespilloides] | 56 | 2 |
| Gene.7376 | PREDICTED: LOW QUALITY PROTEIN: leucine-rich PPR motif-containing protein, mitochondrial [Aethina tumida] | 96 | 2 |
| Gene.738 | PREDICTED: elongation factor Tu, mitochondrial [Aethina tumida] | 365 | 2 |
| Gene.738 | PREDICTED: elongation factor Tu, mitochondrial [Aethina tumida] | 458 | 2 |
| Gene.7388 | short-chain specific acyl-CoA dehydrogenase, mitochondrial [Anoplophora glabripennis] | 60 | 2 |
| Gene.7409 | PREDICTED: T-complex protein 1 subunit delta [Tribolium castaneum] | 36 | 2 |
| Gene.7424 | carbonyl reductase [NADPH] 3 [Anoplophora glabripennis] | 261 | 2 |
| Gene.7445 | PREDICTED: 26S protease regulatory subunit 6B [Nicrophorus vespilloides] | 229 | 2 |
| Gene.7482 | failed axon connections isoform X1 [Anoplophora glabripennis] | 346 | 2 |
| Gene.7522 | elongation factor Ts, mitochondrial, partial [Asbolus verrucosus] | 309 | 2 |
| Gene.7551 | PREDICTED: non-specific lipid-transfer protein [Aethina tumida] | 164 | 2 |
| Gene.7580 | lipid droplet-associated hydrolase [Anoplophora glabripennis] | 95 | 2 |
| Gene.7604 | chromobox protein homolog 3 [Anoplophora glabripennis] | 294 | 2 |
| Gene.7624 | nucleolin 1 [Anoplophora glabripennis] | 355 | 2 |
| Gene.7676 | PREDICTED: guanine nucleotide-binding protein G(q) subunit alpha isoform X1 [Tribolium castaneum] | 68 | 2 |
| Gene.7682 | transmembrane emp24 domain-containing protein 2 [Anoplophora glabripennis] | 129 | 2 |
| Gene.7685 | Uncharacterized protein | 77 | 2 |
| Gene.77 | cathepsin L [Anoplophora glabripennis] | 222 | 2 |
| Gene.7703 | GTP-binding nuclear protein Ran [Onthophagus taurus] | 36 | 2 |
| Gene.7709 | PREDICTED: LOW QUALITY PROTEIN: adenylosuccinate lyase [Aethina tumida] | 172 | 2 |
| Gene.7709 | PREDICTED: LOW QUALITY PROTEIN: adenylosuccinate lyase [Aethina tumida] | 417 | 2 |
| Gene.7709 | PREDICTED: LOW QUALITY PROTEIN: adenylosuccinate lyase [Aethina tumida] | 404 | 2 |
| Gene.7743 | uncharacterized protein LOC110861146 [Folsomia candida] | 30 | 2 |
| Gene.7794 | PREDICTED: sulfide:quinone oxidoreductase, mitochondrial [Tribolium castaneum] | 44 | 2 |
| Gene.7816 | ubiquitin carboxyl-terminal hydrolase 7 isoform X1 [Anoplophora glabripennis] | 321 | 2 |
| Gene.7825 | PREDICTED: importin subunit alpha-4 [Tribolium castaneum] | 386 | 2 |
| Gene.7860 | PREDICTED: lipid storage droplets surface-binding protein 1 isoform X1 [Aethina tumida] | 85 | 2 |
| Gene.7871 | hypothetical protein D910_03374 [Dendroctonus ponderosae] | 212 | 2 |
| Gene.789 | PREDICTED: putative hydroxypyruvate isomerase isoform X2 [Aethina tumida] | 147 | 2 |
| Gene.7917 | ribosomal protein S3 [Tenebrio molitor] | 108 | 2 |
| Gene.7929 | PREDICTED: heat shock 70 kDa protein cognate 3 [Dendroctonus ponderosae] | 268 | 2 |
| Gene.7929 | PREDICTED: heat shock 70 kDa protein cognate 3 [Dendroctonus ponderosae] | 591 | 2 |
| Gene.7938 | PREDICTED: spermine synthase isoform X1 [Tribolium castaneum] | 194 | 2 |
| Gene.7938 | PREDICTED: spermine synthase isoform X1 [Tribolium castaneum] | 235 | 2 |
| Gene.7945 | fatty acid-binding protein, muscle isoform X2 [Anoplophora glabripennis] | 83 | 2 |
| Gene.7945 | fatty acid-binding protein, muscle isoform X2 [Anoplophora glabripennis] | 38 | 2 |
| Gene.7965 | dihydropyrimidinase [Anoplophora glabripennis] | 49 | 2 |
| Gene.7965 | dihydropyrimidinase [Anoplophora glabripennis] | 45 | 2 |
| Gene.7997 | Y-box factor homolog isoform X3 [Leptinotarsa decemlineata] | 22 | 2 |
| Gene.8007 | B-cell receptor-associated protein 31 [Anoplophora glabripennis] | 203 | 2 |
| Gene.8081 | ATP synthase subunit gamma, mitochondrial [Leptinotarsa decemlineata] | 132 | 2 |
| Gene.8081 | ATP synthase subunit gamma, mitochondrial [Leptinotarsa decemlineata] | 175 | 2 |
| Gene.8135 | PREDICTED: calreticulin [Tribolium castaneum] | 283 | 2 |
| Gene.8135 | PREDICTED: calreticulin [Tribolium castaneum] | 99 | 2 |
| Gene.814 | PREDICTED: uncharacterized protein LOC109597149 isoform X2 [Aethina tumida] | 108 | 2 |
| Gene.8252 | T-complex protein 1 subunit eta [Anoplophora glabripennis] | 320 | 2 |
| Gene.8252 | T-complex protein 1 subunit eta [Anoplophora glabripennis] | 425 | 2 |
| Gene.8316 | PREDICTED: inorganic pyrophosphatase isoform X2 [Tribolium castaneum] | 263 | 2 |
| Gene.8316 | PREDICTED: inorganic pyrophosphatase isoform X2 [Tribolium castaneum] | 256 | 2 |
| Gene.8322 | PREDICTED: uncharacterized protein LOC109595590 [Aethina tumida] | 229 | 2 |
| Gene.8339 | PREDICTED: GTP cyclohydrolase 1 [Diuraphis noxia] | 137 | 2 |
| Gene.8399 | ribosomal protein S24e [Meladema coriacea] | 89 | 2 |
| Gene.8415 | Uncharacterized protein | 114 | 2 |
| Gene.8427 | PREDICTED: EH domain-containing protein 3-like [Tribolium castaneum] | 374 | 2 |
| Gene.8441 | PREDICTED: uncharacterized protein LOC657683 [Tribolium castaneum] | 148 | 2 |
| Gene.8445 | nascent polypeptide-associated complex subunit alpha-like isoform X1 [Anoplophora glabripennis] | 118 | 2 |
| Gene.8452 | DCN1-like protein 4 [Agrilus planipennis] | 123 | 2 |
| Gene.8474 | Uncharacterized protein | 310 | 2 |
| Gene.8481 | PREDICTED: arginine kinase isoform X1 [Tribolium castaneum] | 61 | 2 |
| Gene.8507 | moesin/ezrin/radixin homolog 1 isoform X2 [Anoplophora glabripennis] | 262 | 2 |
| Gene.8533 | PREDICTED: 60S acidic ribosomal protein P0 [Aethina tumida] | 274 | 2 |
| Gene.8536 | PREDICTED: LOW QUALITY PROTEIN: isovaleryl-CoA dehydrogenase, mitochondrial [Tribolium castaneum] | 310 | 2 |
| Gene.8583 | PREDICTED: heterogeneous nuclear ribonucleoprotein U-like protein 2 isoform X2 [Tribolium castaneum] | 196 | 2 |
| Gene.8586 | PREDICTED: putative cystathionine gamma-lyase 2 [Aethina tumida] | 72 | 2 |
| Gene.8593 | 26S proteasome regulatory subunit 6A-B [Anoplophora glabripennis] | 276 | 2 |
| Gene.8594 | PREDICTED: 60S ribosomal protein L35a [Tribolium castaneum] | 24 | 2 |
| Gene.8616 | dihydrolipoyllysine-residue acetyltransferase component of pyruvate dehydrogenase complex, mitochondrial isoform X1 [Anoplophora glabripennis] | 383 | 2 |
| Gene.8624 | eukaryotic translation initiation factor 3 subunit E [Anoplophora glabripennis] | 385 | 2 |
| Gene.8673 | succinate dehydrogenase [ubiquinone] iron-sulfur subunit, mitochondrial, partial [Asbolus verrucosus] | 221 | 2 |
| Gene.8707 | PREDICTED: heterogeneous nuclear ribonucleoprotein Q isoform X1 [Tribolium castaneum] | 373 | 2 |
| Gene.8756 | uncharacterized protein Dvir_GJ16722 [Drosophila virilis] | 32 | 2 |
| Gene.8758 | PREDICTED: la protein homolog [Tribolium castaneum] | 291 | 2 |
| Gene.8787 | UDPGT domain containing protein [Asbolus verrucosus] | 295 | 2 |
| Gene.8825 | T-complex protein 1 subunit theta [Anoplophora glabripennis] | 289 | 2 |
| Gene.8830 | PREDICTED: probable 26S proteasome non-ATPase regulatory subunit 3 [Tribolium castaneum] | 382 | 2 |
| Gene.8840 | signal recognition particle subunit SRP72 [Asbolus verrucosus] | 291 | 2 |
| Gene.8844 | ubiquitin carboxyl-terminal hydrolase 5 [Anoplophora glabripennis] | 410 | 2 |
| Gene.8856 | eukaryotic peptide chain release factor GTP-binding subunit ERF3A [Asbolus verrucosus] | 572 | 2 |
| Gene.8862 | protein UBASH3A homolog isoform X2 [Onthophagus taurus] | 213 | 2 |
| Gene.8862 | protein UBASH3A homolog isoform X2 [Onthophagus taurus] | 14 | 2 |
| Gene.8873 | PREDICTED: fumarate hydratase, mitochondrial [Tribolium castaneum] | 434 | 2 |
| Gene.8903 | PREDICTED: T-complex protein 1 subunit epsilon [Tribolium castaneum] | 266 | 2 |
| Gene.8917 | PREDICTED: ester hydrolase C11orf54 homolog isoform X1 [Tribolium castaneum] | 88 | 2 |
| Gene.8938 | 14-3-3 epsilon [Tenebrio molitor] | 118 | 2 |
| Gene.8989 | PREDICTED: aldehyde dehydrogenase, dimeric NADP-preferring-like [Aethina tumida] | 277 | 2 |
| Gene.8995 | PREDICTED: 60S ribosomal protein L9 [Megachile rotundata] | 84 | 2 |
| Gene.9023 | uncharacterized protein LOC108903465 [Anoplophora glabripennis] | 100 | 2 |
| Gene.9034 | 60S ribosomal protein L7 [Anoplophora glabripennis] | 74 | 2 |
| Gene.9046 | hsp90 co-chaperone Cdc37 [Anoplophora glabripennis] | 249 | 2 |
| Gene.9064 | mitochondrial amidoxime-reducing component 1-like [Anoplophora glabripennis] | 107 | 2 |
| Gene.924 | 26S proteasome non-ATPase regulatory subunit 11 [Asbolus verrucosus] | 302 | 2 |
| Gene.924 | 26S proteasome non-ATPase regulatory subunit 11 [Asbolus verrucosus] | 177 | 2 |
| Gene.95 | Uncharacterized protein | 173 | 2 |
| Gene.950 | heat shock protein 75 kDa, mitochondrial, partial [Asbolus verrucosus] | 121 | 2 |
| Gene.960 | probable phosphoserine aminotransferase [Anoplophora glabripennis] | 118 | 2 |
| Gene.996 | PREDICTED: myelin expression factor 2 [Tribolium castaneum] | 274 | 2 |
| Gene.996 | PREDICTED: myelin expression factor 2 [Tribolium castaneum] | 497 | 2 |
| Gene.1048 | FK506-binding protein 2 [Onthophagus taurus] | 189 | 1 |
| Gene.106 | Uncharacterized protein | 170 | 1 |
| Gene.1149 | PREDICTED: protein takeout-like [Aethina tumida] | 118 | 1 |
| Gene.1158 | PREDICTED: uncharacterized protein LOC109537982 isoform X2 [Dendroctonus ponderosae] | 235 | 1 |
| Gene.1200 | cytosolic iron-sulfur protein assembly protein Ciao1 [Asbolus verrucosus] | 271 | 1 |
| Gene.1211 | adh short, KR, NAD binding 10, and/or Epimerase domain containing protein [Asbolus verrucosus] | 120 | 1 |
| Gene.1212 | leukotriene A-4 hydrolase isoform X1 [Anoplophora glabripennis] | 369 | 1 |
| Gene.1264 | PREDICTED: protein disulfide-isomerase [Tribolium castaneum] | 327 | 1 |
| Gene.130 | PREDICTED: T-complex protein 1 subunit beta [Tribolium castaneum] | 235 | 1 |
| Gene.1358 | Uncharacterized protein | 26 | 1 |
| Gene.1391 | PREDICTED: protein lethal(2)essential for life [Tribolium castaneum] | 109 | 1 |
| Gene.1398 | PREDICTED: multifunctional protein ADE2 [Aethina tumida] | 41 | 1 |
| Gene.1403 | Protein LSM14 homolog B-like Protein [Tribolium castaneum] | 531 | 1 |
| Gene.1403 | Protein LSM14 homolog B-like Protein [Tribolium castaneum] | 521 | 1 |
| Gene.141 | adenosine kinase [Anoplophora glabripennis] | 102 | 1 |
| Gene.1434 | PREDICTED: PDZ domain-containing protein GIPC3 [Tribolium castaneum] | 184 | 1 |
| Gene.1489 | aspartate aminotransferase, mitochondrial [Asbolus verrucosus] | 56 | 1 |
| Gene.1513 | reticulon-1-A isoform X2 [Leptinotarsa decemlineata] | 241 | 1 |
| Gene.152 | PREDICTED: LOW QUALITY PROTEIN: uncharacterized protein LOC109596484 [Aethina tumida] | 33 | 1 |
| Gene.1539 | TROVE domain containing protein [Asbolus verrucosus] | 531 | 1 |
| Gene.154 | PREDICTED: 26S protease regulatory subunit 7 [Tribolium castaneum] | 25 | 1 |
| Gene.154 | PREDICTED: 26S protease regulatory subunit 7 [Tribolium castaneum] | 22 | 1 |
| Gene.1542 | PREDICTED: ATP synthase subunit alpha, mitochondrial [Tribolium castaneum] | 192 | 1 |
| Gene.1559 | PREDICTED: signal recognition particle 54 kDa protein [Tribolium castaneum] | 311 | 1 |
| Gene.1575 | Ubiquitin-conjugating enzyme [Oryctes borbonicus] | 153 | 1 |
| Gene.1660 | hypothetical protein TcasGA2_TC031159 [Tribolium castaneum] | 307 | 1 |
| Gene.170 | RNA-binding protein lark [Asbolus verrucosus] | 20 | 1 |
| Gene.1724 | cyclic GMP-AMP synthase-like isoform X1 [Leptinotarsa decemlineata] | 67 | 1 |
| Gene.1740 | LOW QUALITY PROTEIN: eukaryotic translation initiation factor 4E transporter [Anoplophora glabripennis] | 324 | 1 |
| Gene.1779 | PREDICTED: lambda-crystallin [Tribolium castaneum] | 266 | 1 |
| Gene.1789 | thioredoxin domain-containing protein 5 [Asbolus verrucosus] | 82 | 1 |
| Gene.179 | dipeptidyl peptidase 3 [Anoplophora glabripennis] | 642 | 1 |
| Gene.18 | PREDICTED: estradiol 17-beta-dehydrogenase 8 isoform X1 [Aethina tumida] | 43 | 1 |
| Gene.182 | cytosolic non-specific dipeptidase [Anoplophora glabripennis] | 90 | 1 |
| Gene.1821 | PREDICTED: glycerophosphodiester phosphodiesterase 1 [Tribolium castaneum] | 340 | 1 |
| Gene.1871 | endoribonuclease Dicer [Anoplophora glabripennis] | 45 | 1 |
| Gene.1891 | hypothetical protein AMK59_3112 [Oryctes borbonicus] | 4 | 1 |
| Gene.1895 | heat shock protein 70 [Harmonia axyridis] | 453 | 1 |
| Gene.1899 | 40S ribosomal protein S18 [Agrilus planipennis] | 32 | 1 |
| Gene.1900 | Neprilysin-2-like Protein [Tribolium castaneum] | 376 | 1 |
| Gene.1912 | PREDICTED: NADH dehydrogenase [ubiquinone] 1 beta subcomplex subunit 9 [Aethina tumida] | 62 | 1 |
| Gene.1914 | 3-hydroxyisobutyryl-CoA hydrolase, mitochondrial isoform X1 [Anoplophora glabripennis] | 370 | 1 |
| Gene.1921 | obg-like ATPase 1 [Anoplophora glabripennis] | 324 | 1 |
| Gene.1931 | PREDICTED: hydroxysteroid dehydrogenase-like protein 1 isoform X2 [Tribolium castaneum] | 126 | 1 |
| Gene.1932 | PREDICTED: 2-oxoglutarate dehydrogenase, mitochondrial isoform X2 [Tribolium castaneum] | 880 | 1 |
| Gene.1936 | PREDICTED: cAMP-specific 3',5'-cyclic phosphodiesterase 4A-like isoform X3 [Tribolium castaneum] | 682 | 1 |
| Gene.196 | PREDICTED: triosephosphate isomerase isoform X1 [Aethina tumida] | 152 | 1 |
| Gene.2004 | PREDICTED: ras-like protein 3 [Tribolium castaneum] | 117 | 1 |
| Gene.2016 | PREDICTED: 40S ribosomal protein S4 isoform X1 [Aethina tumida] | 53 | 1 |
| Gene.2034 | NADH dehydrogenase [ubiquinone] flavoprotein 1, mitochondrial [Tribolium castaneum] | 80 | 1 |
| Gene.2037 | ATPase family AAA domain-containing protein 3A homolog [Leptinotarsa decemlineata] | 94 | 1 |
| Gene.2060 | PREDICTED: HBS1-like protein [Tribolium castaneum] | 122 | 1 |
| Gene.2091 | dihydrofolate reductase [Asbolus verrucosus] | 78 | 1 |
| Gene.2098 | PREDICTED: cytochrome P450 9e2-like [Nicrophorus vespilloides] | 174 | 1 |
| Gene.2140 | PREDICTED: UDP-glucuronosyltransferase 2B10-like [Tribolium castaneum] | 331 | 1 |
| Gene.2144 | PREDICTED: succinyl-CoA ligase subunit alpha, mitochondrial [Tribolium castaneum] | 298 | 1 |
| Gene.2144 | PREDICTED: succinyl-CoA ligase subunit alpha, mitochondrial [Tribolium castaneum] | 322 | 1 |
| Gene.2149 | heat shock protein, partial [Cryptolaemus montrouzieri] | 205 | 1 |
| Gene.2149 | heat shock protein, partial [Cryptolaemus montrouzieri] | 87 | 1 |
| Gene.2190 | probable 28S ribosomal protein S25, mitochondrial [Anoplophora glabripennis] | 112 | 1 |
| Gene.2195 | selenium-binding protein 1 [Asbolus verrucosus] | 374 | 1 |
| Gene.2211 | PREDICTED: L-xylulose reductase-like [Aethina tumida] | 26 | 1 |
| Gene.2223 | PREDICTED: nodal modulator 1 [Tribolium castaneum] | 85 | 1 |
| Gene.2226 | protein spinster isoform X2 [Leptinotarsa decemlineata] | 53 | 1 |
| Gene.2227 | PREDICTED: ubiquitin-like modifier-activating enzyme 1 [Tribolium castaneum] | 525 | 1 |
| Gene.2256 | nucleolar and coiled-body phosphoprotein 1-like isoform X2 [Leptinotarsa decemlineata] | 32 | 1 |
| Gene.229 | PREDICTED: probable phospholipid hydroperoxide glutathione peroxidase [Tribolium castaneum] | 195 | 1 |
| Gene.2290 | malonyl-CoA-acyl carrier protein transacylase, mitochondrial, partial [Asbolus verrucosus] | 137 | 1 |
| Gene.2304 | PREDICTED: ferrochelatase, mitochondrial [Tribolium castaneum] | 140 | 1 |
| Gene.2330 | guanine nucleotide-binding protein subunit beta-like protein [Anoplophora glabripennis] | 48 | 1 |
| Gene.2348 | ras-related protein Rab-5B isoform X1 [Leptinotarsa decemlineata] | 140 | 1 |
| Gene.2352 | PREDICTED: patronin isoform X1 [Tribolium castaneum] | 133 | 1 |
| Gene.2352 | PREDICTED: patronin isoform X1 [Tribolium castaneum] | 135 | 1 |
| Gene.2363 | 26S proteasome non-ATPase regulatory subunit 2 [Anoplophora glabripennis] | 527 | 1 |
| Gene.2368 | PREDICTED: filamin-A isoform X6 [Tribolium castaneum] | 2148 | 1 |
| Gene.2375 | glyceraldehyde-3-phosphate, partial [Harmonia axyridis] | 119 | 1 |
| Gene.2388 | spermine oxidase, partial [Asbolus verrucosus] | 128 | 1 |
| Gene.2466 | PREDICTED: LOW QUALITY PROTEIN: extended synaptotagmin-2-like [Aethina tumida] | 85 | 1 |
| Gene.2469 | PREDICTED: flightin [Tribolium castaneum] | 132 | 1 |
| Gene.2484 | PREDICTED: fatty-acid amide hydrolase 2-B [Tribolium castaneum] | 94 | 1 |
| Gene.2562 | hypothetical protein AMK59_6989 [Oryctes borbonicus] | 63 | 1 |
| Gene.2569 | 1,5-anhydro-D-fructose reductase-like Protein [Tribolium castaneum] | 152 | 1 |
| Gene.2583 | rRNA 2'-O-methyltransferase fibrillarin [Agrilus planipennis] | 217 | 1 |
| Gene.2587 | Uncharacterized protein | 346 | 1 |
| Gene.2587 | Uncharacterized protein | 235 | 1 |
| Gene.2636 | ADP ribosyl GH and/or G-gamma domain containing protein [Asbolus verrucosus] | 60 | 1 |
| Gene.2656 | PREDICTED: aspartate aminotransferase, cytoplasmic [Tribolium castaneum] | 317 | 1 |
| Gene.2683 | PREDICTED: proteasome subunit beta type-2 [Tribolium castaneum] | 161 | 1 |
| Gene.2683 | PREDICTED: proteasome subunit beta type-2 [Tribolium castaneum] | 68 | 1 |
| Gene.2725 | PREDICTED: endoplasmin [Tribolium castaneum] | 478 | 1 |
| Gene.2800 | PREDICTED: hydroxysteroid dehydrogenase-like protein 2 [Aethina tumida] | 56 | 1 |
| Gene.2801 | tyrosine-protein phosphatase non-receptor type 14 isoform X1 [Anoplophora glabripennis] | 89 | 1 |
| Gene.2841 | ornithine aminotransferase, mitochondrial-like [Leptinotarsa decemlineata] | 389 | 1 |
| Gene.2878 | fatty acid synthase-like [Agrilus planipennis] | 152 | 1 |
| Gene.2918 | plasminogen activator inhibitor 1 RNA-binding protein isoform X3 [Anoplophora glabripennis] | 272 | 1 |
| Gene.2933 | isocitrate dehydrogenase [NADP], mitochondrial-like [Onthophagus taurus] | 57 | 1 |
| Gene.2958 | alpha actinin [Coleomegilla maculata] | 843 | 1 |
| Gene.3063 | PREDICTED: uncharacterized protein LOC108560181 [Nicrophorus vespilloides] | 148 | 1 |
| Gene.3069 | PREDICTED: glycine dehydrogenase (decarboxylating), mitochondrial [Tribolium castaneum] | 99 | 1 |
| Gene.320 | probable serine/threonine-protein kinase dyrk2 isoform X3 [Anoplophora glabripennis] | 459 | 1 |
| Gene.3208 | phosphoglucose isomerase [Colias eurytheme] | 559 | 1 |
| Gene.3223 | Aldose reductase-like Protein [Tribolium castaneum] | 243 | 1 |
| Gene.3232 | PREDICTED: transketolase-like protein 2 isoform X1 [Tribolium castaneum] | 263 | 1 |
| Gene.3243 | cytochrome P450 monooxygenase [Tribolium castaneum] | 411 | 1 |
| Gene.3247 | PREDICTED: phosphoenolpyruvate carboxykinase [GTP] isoform X1 [Tribolium castaneum] | 396 | 1 |
| Gene.3417 | inter-alpha-trypsin inhibitor heavy chain H4-like, partial [Asbolus verrucosus] | 84 | 1 |
| Gene.3417 | inter-alpha-trypsin inhibitor heavy chain H4-like, partial [Asbolus verrucosus] | 540 | 1 |
| Gene.342 | PREDICTED: 40S ribosomal protein S9 [Tribolium castaneum] | 53 | 1 |
| Gene.342 | PREDICTED: 40S ribosomal protein S9 [Tribolium castaneum] | 23 | 1 |
| Gene.3482 | V-type proton ATPase catalytic subunit A [Leptinotarsa decemlineata] | 12 | 1 |
| Gene.3489 | PREDICTED: acetyl-CoA carboxylase isoform X1 [Tribolium castaneum] | 1616 | 1 |
| Gene.3489 | PREDICTED: acetyl-CoA carboxylase isoform X1 [Tribolium castaneum] | 897 | 1 |
| Gene.3489 | PREDICTED: acetyl-CoA carboxylase isoform X1 [Tribolium castaneum] | 2381 | 1 |
| Gene.3501 | PREDICTED: PDZ and LIM domain protein Zasp isoform X5 [Tribolium castaneum] | 484 | 1 |
| Gene.351 | 6-phosphogluconolactonase [Asbolus verrucosus] | 7 | 1 |
| Gene.3518 | vitellogenin 2 [Harmonia axyridis] | 603 | 1 |
| Gene.3522 | vitellogenin 1 [Harmonia axyridis] | 91 | 1 |
| Gene.354 | saccharopine dehydrogenase-like [Asbolus verrucosus] | 27 | 1 |
| Gene.3545 | PREDICTED: uncharacterized protein LOC103312213 [Tribolium castaneum] | 146 | 1 |
| Gene.3545 | PREDICTED: uncharacterized protein LOC103312213 [Tribolium castaneum] | 143 | 1 |
| Gene.3548 | E3 SUMO-protein ligase RanBP2 [Anoplophora glabripennis] | 455 | 1 |
| Gene.3558 | PREDICTED: probable aconitate hydratase, mitochondrial [Tribolium castaneum] | 613 | 1 |
| Gene.3676 | hypothetical protein D910_04355 [Dendroctonus ponderosae] | 120 | 1 |
| Gene.3676 | hypothetical protein D910_04355 [Dendroctonus ponderosae] | 195 | 1 |
| Gene.3731 | PREDICTED: UDP-glucuronosyltransferase 2B2 [Tribolium castaneum] | 85 | 1 |
| Gene.376 | Galactosyl T domain containing protein [Asbolus verrucosus] | 7 | 1 |
| Gene.3795 | NADH-ubiquinone oxidoreductase 75 kDa subunit, mitochondrial [Asbolus verrucosus] | 680 | 1 |
| Gene.3807 | scavenger receptor protein [Tribolium castaneum] | 264 | 1 |
| Gene.3838 | PREDICTED: pyruvate carboxylase, mitochondrial isoform X3 [Tribolium castaneum] | 35 | 1 |
| Gene.3838 | PREDICTED: pyruvate carboxylase, mitochondrial isoform X3 [Tribolium castaneum] | 565 | 1 |
| Gene.3838 | PREDICTED: pyruvate carboxylase, mitochondrial isoform X3 [Tribolium castaneum] | 727 | 1 |
| Gene.3838 | PREDICTED: pyruvate carboxylase, mitochondrial isoform X3 [Tribolium castaneum] | 253 | 1 |
| Gene.3875 | PREDICTED: probable methylmalonate-semialdehyde dehydrogenase [acylating], mitochondrial [Aethina tumida] | 330 | 1 |
| Gene.3918 | microtubule-actin cross-linking factor 1 isoform X5 [Anoplophora glabripennis] | 336 | 1 |
| Gene.3947 | PREDICTED: delta-1-pyrroline-5-carboxylate synthase [Tribolium castaneum] | 85 | 1 |
| Gene.3947 | PREDICTED: delta-1-pyrroline-5-carboxylate synthase [Tribolium castaneum] | 458 | 1 |
| Gene.3948 | PREDICTED: delta-1-pyrroline-5-carboxylate synthase [Nicrophorus vespilloides] | 18 | 1 |
| Gene.3959 | PREDICTED: synaptic vesicle glycoprotein 2B [Tribolium castaneum] | 174 | 1 |
| Gene.3990 | 40S ribosomal protein S26 [Anoplophora glabripennis] | 66 | 1 |
| Gene.3996 | myosin-IB [Anoplophora glabripennis] | 285 | 1 |
| Gene.4011 | fatty acid synthase [Coccinella septempunctata] | 2320 | 1 |
| Gene.4011 | fatty acid synthase [Coccinella septempunctata] | 1029 | 1 |
| Gene.4015 | activated RNA polymerase II transcriptional coactivator p15 [Asbolus verrucosus] | 52 | 1 |
| Gene.4018 | glutamyl aminopeptidase-like isoform X2 [Leptinotarsa decemlineata] | 425 | 1 |
| Gene.4113 | succinyl-CoA:3-ketoacid coenzyme A transferase 1, mitochondrial, partial [Asbolus verrucosus] | 198 | 1 |
| Gene.4134 | PREDICTED: AMP deaminase 2 isoform X6 [Tribolium castaneum] | 411 | 1 |
| Gene.4134 | PREDICTED: AMP deaminase 2 isoform X6 [Tribolium castaneum] | 676 | 1 |
| Gene.4149 | PREDICTED: LOW QUALITY PROTEIN: 26S proteasome non-ATPase regulatory subunit 1-like [Aethina tumida] | 313 | 1 |
| Gene.4183 | PREDICTED: proton-coupled amino acid transporter 1 isoform X1 [Tribolium castaneum] | 7 | 1 |
| Gene.4215 | trehalose 6-phosphate synthase [Harmonia axyridis] | 259 | 1 |
| Gene.4254 | isocitrate dehydrogenase [NAD] subunit gamma, mitochondrial isoform X2 [Anoplophora glabripennis] | 206 | 1 |
| Gene.426 | PREDICTED: LOW QUALITY PROTEIN: amidophosphoribosyltransferase-like [Aethina tumida] | 405 | 1 |
| Gene.4299 | electron transfer flavoprotein-ubiquinone oxidoreductase, mitochondrial [Anoplophora glabripennis] | 344 | 1 |
| Gene.4352 | PREDICTED: calcium-transporting ATPase sarcoplasmic/endoplasmic reticulum type isoform X1 [Tribolium castaneum] | 549 | 1 |
| Gene.4384 | Twitchin-like Protein [Tribolium castaneum] | 773 | 1 |
| Gene.455 | ATP-binding cassette sub-family E member 1 [Anoplophora glabripennis] | 593 | 1 |
| Gene.4583 | PREDICTED: ATP-citrate synthase isoform X1 [Tribolium castaneum] | 65 | 1 |
| Gene.4592 | pre-mRNA-processing factor 39-like isoform X2 [Leptinotarsa decemlineata] | 198 | 1 |
| Gene.4593 | spectrin beta chain isoform X1 [Anoplophora glabripennis] | 649 | 1 |
| Gene.4593 | spectrin beta chain isoform X1 [Anoplophora glabripennis] | 269 | 1 |
| Gene.4608 | PREDICTED: ELAV-like protein 3 isoform X2 [Tribolium castaneum] | 344 | 1 |
| Gene.4609 | mucin-2 isoform X3 [Anoplophora glabripennis] | 578 | 1 |
| Gene.4683 | dyslexia-associated protein KIAA0319-like protein [Anoplophora glabripennis] | 246 | 1 |
| Gene.469 | AP-1 complex subunit beta-1 [Asbolus verrucosus] | 854 | 1 |
| Gene.4714 | PREDICTED: 60S ribosomal protein L8 [Polistes canadensis] | 80 | 1 |
| Gene.4718 | AGAP001345-PA [Anopheles gambiae str. PEST] | 152 | 1 |
| Gene.4720 | hexamerin 3 precursor [Tribolium castaneum] | 55 | 1 |
| Gene.473 | T-complex protein 1 subunit zeta [Asbolus verrucosus] | 141 | 1 |
| Gene.473 | T-complex protein 1 subunit zeta [Asbolus verrucosus] | 270 | 1 |
| Gene.4752 | hexamerin 3 precursor [Tribolium castaneum] | 158 | 1 |
| Gene.4787 | paramyosin, long form isoform X2 [Leptinotarsa decemlineata] | 178 | 1 |
| Gene.4867 | PREDICTED: spectrin alpha chain isoform X4 [Tribolium castaneum] | 455 | 1 |
| Gene.4867 | PREDICTED: spectrin alpha chain isoform X4 [Tribolium castaneum] | 517 | 1 |
| Gene.4867 | PREDICTED: spectrin alpha chain isoform X4 [Tribolium castaneum] | 145 | 1 |
| Gene.4867 | PREDICTED: spectrin alpha chain isoform X4 [Tribolium castaneum] | 541 | 1 |
| Gene.4867 | PREDICTED: spectrin alpha chain isoform X4 [Tribolium castaneum] | 649 | 1 |
| Gene.4867 | PREDICTED: spectrin alpha chain isoform X4 [Tribolium castaneum] | 691 | 1 |
| Gene.4934 | apolipophorins [Anoplophora glabripennis] | 297 | 1 |
| Gene.4934 | apolipophorins [Anoplophora glabripennis] | 815 | 1 |
| Gene.4957 | Protein mesh-like Protein [Tribolium castaneum] | 505 | 1 |
| Gene.4973 | PREDICTED: myosin heavy chain, muscle isoform X18 [Tribolium castaneum] | 1781 | 1 |
| Gene.4973 | PREDICTED: myosin heavy chain, muscle isoform X18 [Tribolium castaneum] | 44 | 1 |
| Gene.4973 | PREDICTED: myosin heavy chain, muscle isoform X18 [Tribolium castaneum] | 1459 | 1 |
| Gene.4973 | PREDICTED: myosin heavy chain, muscle isoform X18 [Tribolium castaneum] | 87 | 1 |
| Gene.4973 | PREDICTED: myosin heavy chain, muscle isoform X18 [Tribolium castaneum] | 1016 | 1 |
| Gene.500 | alanine aminotransferase 1 [Anoplophora glabripennis] | 170 | 1 |
| Gene.5002 | titin [Asbolus verrucosus] | 1780 | 1 |
| Gene.5018 | hypothetical protein TcasGA2_TC034351 [Tribolium castaneum] | 524 | 1 |
| Gene.5051 | spectrin alpha chain, non-erythrocytic 1, partial [Asbolus verrucosus] | 1308 | 1 |
| Gene.5104 | PREDICTED: dihydropyrimidine dehydrogenase [NADP(+)] isoform X2 [Tribolium castaneum] | 749 | 1 |
| Gene.5151 | glycogen debranching enzyme [Leptinotarsa decemlineata] | 936 | 1 |
| Gene.524 | PREDICTED: trifunctional purine biosynthetic protein adenosine-3 [Tribolium castaneum] | 466 | 1 |
| Gene.526 | signal recognition particle subunit SRP68 [Anoplophora glabripennis] | 206 | 1 |
| Gene.5335 | PREDICTED: interleukin enhancer-binding factor 2 homolog [Tribolium castaneum] | 128 | 1 |
| Gene.5399 | PREDICTED: 60S ribosomal protein L24 [Aethina tumida] | 69 | 1 |
| Gene.5401 | transmembrane emp24 domain-containing protein bai, partial [Anoplophora glabripennis] | 96 | 1 |
| Gene.5435 | putative GABA-A receptor associated protein [Graphocephala atropunctata] | 20 | 1 |
| Gene.5447 | probable small nuclear ribonucleoprotein Sm D2 [Anoplophora glabripennis] | 91 | 1 |
| Gene.548 | clavesin-1 isoform X1 [Agrilus planipennis] | 112 | 1 |
| Gene.548 | clavesin-1 isoform X1 [Agrilus planipennis] | 99 | 1 |
| Gene.5542 | catalase [Onthophagus taurus] | 243 | 1 |
| Gene.5561 | stomatin-like protein 2, mitochondrial [Anoplophora glabripennis] | 225 | 1 |
| Gene.5561 | stomatin-like protein 2, mitochondrial [Anoplophora glabripennis] | 78 | 1 |
| Gene.5602 | 60S ribosomal protein L13 [Anoplophora glabripennis] | 136 | 1 |
| Gene.5602 | 60S ribosomal protein L13 [Anoplophora glabripennis] | 105 | 1 |
| Gene.5612 | translin [Leptinotarsa decemlineata] | 82 | 1 |
| Gene.5636 | ras-related protein Rab-11A [Anoplophora glabripennis] | 58 | 1 |
| Gene.572 | PREDICTED: phosphoglycerate kinase [Tribolium castaneum] | 289 | 1 |
| Gene.5850 | PREDICTED: hrp65 protein-like [Aethina tumida] | 185 | 1 |
| Gene.5868 | PREDICTED: V-type proton ATPase subunit D [Polistes canadensis] | 215 | 1 |
| Gene.5898 | PREDICTED: 60S ribosomal protein L36 [Tribolium castaneum] | 57 | 1 |
| Gene.5904 | eukaryotic translation initiation factor 2 subunit 2 [Asbolus verrucosus] | 249 | 1 |
| Gene.5910 | PREDICTED: NADH dehydrogenase [ubiquinone] 1 alpha subcomplex subunit 10, mitochondrial [Tribolium castaneum] | 388 | 1 |
| Gene.5941 | PREDICTED: LOW QUALITY PROTEIN: 26S proteasome non-ATPase regulatory subunit 13 [Aethina tumida] | 307 | 1 |
| Gene.5973 | ribosomal protein S13, partial [Harmonia axyridis] | 112 | 1 |
| Gene.5994 | PREDICTED: LOW QUALITY PROTEIN: histidine triad nucleotide-binding protein 1-like [Aethina tumida] | 68 | 1 |
| Gene.6001 | proteasome subunit alpha type-6 [Anoplophora glabripennis] | 102 | 1 |
| Gene.6018 | Beta-parvin-like Protein [Tribolium castaneum] | 86 | 1 |
| Gene.6023 | uncharacterized protein LOC108917395 [Anoplophora glabripennis] | 127 | 1 |
| Gene.6124 | PREDICTED: 60S ribosomal protein L17 [Aethina tumida] | 55 | 1 |
| Gene.6128 | protein HGV2-like isoform X1 [Leptinotarsa decemlineata] | 278 | 1 |
| Gene.613 | uncharacterized protein BDFB_007853 [Asbolus verrucosus] | 26 | 1 |
| Gene.6135 | PREDICTED: NADH dehydrogenase [ubiquinone] 1 alpha subcomplex subunit 13-like [Aethina tumida] | 59 | 1 |
| Gene.6165 | 40S ribosomal protein S20 [Pogonomyrmex barbatus] | 45 | 1 |
| Gene.6186 | succinate dehydrogenase cytochrome b560 subunit, mitochondrial [Anoplophora glabripennis] | 69 | 1 |
| Gene.6210 | serine/threonine-protein phosphatase Pgam5, mitochondrial [Asbolus verrucosus] | 86 | 1 |
| Gene.627 | dnaJ homolog subfamily C member 3 [Anoplophora glabripennis] | 442 | 1 |
| Gene.6354 | Uncharacterized protein | 84 | 1 |
| Gene.6433 | PREDICTED: LOW QUALITY PROTEIN: coatomer subunit beta' [Aethina tumida] | 64 | 1 |
| Gene.6439 | PREDICTED: protein DEK isoform X3 [Tribolium castaneum] | 235 | 1 |
| Gene.6448 | glycine cleavage system H protein, mitochondrial [Leptinotarsa decemlineata] | 112 | 1 |
| Gene.6508 | PREDICTED: acyl carrier protein, mitochondrial isoform X1 [Dendroctonus ponderosae] | 80 | 1 |
| Gene.6546 | eukaryotic translation initiation factor 4A [Epicauta chinensis] | 13 | 1 |
| Gene.6546 | eukaryotic translation initiation factor 4A [Epicauta chinensis] | 311 | 1 |
| Gene.6559 | PREDICTED: transcriptional activator protein Pur-alpha isoform X1 [Tribolium castaneum] | 96 | 1 |
| Gene.658 | Uncharacterized protein | 140 | 1 |
| Gene.6605 | hypothetical protein AMK59_7061, partial [Oryctes borbonicus] | 69 | 1 |
| Gene.6605 | hypothetical protein AMK59_7061, partial [Oryctes borbonicus] | 65 | 1 |
| Gene.6607 | Uncharacterized protein | 114 | 1 |
| Gene.665 | PREDICTED: 1,4-alpha-glucan-branching enzyme [Aethina tumida] | 264 | 1 |
| Gene.6679 | Isocitrate dehydrogenase [NADP] [Operophtera brumata] | 221 | 1 |
| Gene.6679 | Isocitrate dehydrogenase [NADP] [Operophtera brumata] | 114 | 1 |
| Gene.6692 | D-3-phosphoglycerate dehydrogenase, partial [Asbolus verrucosus] | 155 | 1 |
| Gene.6721 | nuclear valosin-containing protein-like isoform X1 [Anoplophora glabripennis] | 435 | 1 |
| Gene.6801 | hypothetical protein TcasGA2_TC031075 [Tribolium castaneum] | 308 | 1 |
| Gene.686 | cullin-3 [Anoplophora glabripennis] | 239 | 1 |
| Gene.6885 | PREDICTED: RNA-binding protein cabeza [Tribolium castaneum] | 104 | 1 |
| Gene.6895 | PREDICTED: acyl-CoA-binding domain-containing protein 5 [Aethina tumida] | 60 | 1 |
| Gene.6924 | PREDICTED: 40S ribosomal protein SA [Tribolium castaneum] | 53 | 1 |
| Gene.6969 | kynurenine--oxoglutarate transaminase 3 [Anoplophora glabripennis] | 180 | 1 |
| Gene.6969 | kynurenine--oxoglutarate transaminase 3 [Anoplophora glabripennis] | 89 | 1 |
| Gene.6977 | AFG3-like protein 2 [Anoplophora glabripennis] | 179 | 1 |
| Gene.6992 | PREDICTED: 7-methylguanosine phosphate-specific 5'-nucleotidase isoform X1 [Tribolium castaneum] | 38 | 1 |
| Gene.6992 | PREDICTED: 7-methylguanosine phosphate-specific 5'-nucleotidase isoform X1 [Tribolium castaneum] | 228 | 1 |
| Gene.6997 | PREDICTED: glyoxylate reductase/hydroxypyruvate reductase [Dendroctonus ponderosae] | 203 | 1 |
| Gene.6997 | PREDICTED: glyoxylate reductase/hydroxypyruvate reductase [Dendroctonus ponderosae] | 256 | 1 |
| Gene.7038 | 60S ribosomal protein L18a [Leptinotarsa decemlineata] | 173 | 1 |
| Gene.7082 | PREDICTED: atlastin isoform X1 [Tribolium castaneum] | 303 | 1 |
| Gene.7108 | unknown [Dendroctonus ponderosae] | 396 | 1 |
| Gene.7216 | PREDICTED: microtubule-associated protein RP/EB family member 1 isoform X2 [Tribolium castaneum] | 216 | 1 |
| Gene.7237 | Uncharacterized protein | 65 | 1 |
| Gene.7264 | heat shock protein 90, partial [Harmonia axyridis] | 485 | 1 |
| Gene.7276 | protein disulfide-isomerase A3 [Asbolus verrucosus] | 144 | 1 |
| Gene.730 | long-chain-fatty-acid--CoA ligase ACSBG2 [Anoplophora glabripennis] | 67 | 1 |
| Gene.730 | long-chain-fatty-acid--CoA ligase ACSBG2 [Anoplophora glabripennis] | 342 | 1 |
| Gene.7309 | PREDICTED: methionine aminopeptidase 2 [Tribolium castaneum] | 138 | 1 |
| Gene.7318 | mitogen-activated protein kinase 1 [Anoplophora glabripennis] | 82 | 1 |
| Gene.7326 | serine hydroxymethyltransferase, cytosolic isoform X1 [Anoplophora glabripennis] | 337 | 1 |
| Gene.7378 | PREDICTED: delta(3,5)-Delta(2,4)-dienoyl-CoA isomerase, mitochondrial [Tribolium castaneum] | 125 | 1 |
| Gene.738 | PREDICTED: elongation factor Tu, mitochondrial [Aethina tumida] | 301 | 1 |
| Gene.7387 | PREDICTED: gelsolin [Tribolium castaneum] | 63 | 1 |
| Gene.7399 | 3-hydroxyisobutyrate dehydrogenase, mitochondrial [Leptinotarsa decemlineata] | 218 | 1 |
| Gene.7409 | PREDICTED: T-complex protein 1 subunit delta [Tribolium castaneum] | 333 | 1 |
| Gene.7422 | chemosensory protein 2 [Bactrocera minax] | 110 | 1 |
| Gene.7424 | carbonyl reductase [NADPH] 3 [Anoplophora glabripennis] | 177 | 1 |
| Gene.7443 | PREDICTED: regulator of chromosome condensation [Tribolium castaneum] | 21 | 1 |
| Gene.7482 | failed axon connections isoform X1 [Anoplophora glabripennis] | 46 | 1 |
| Gene.7551 | PREDICTED: non-specific lipid-transfer protein [Aethina tumida] | 29 | 1 |
| Gene.7567 | enoyl-CoA hydratase domain-containing protein 3, mitochondrial [Leptinotarsa decemlineata] | 104 | 1 |
| Gene.7567 | enoyl-CoA hydratase domain-containing protein 3, mitochondrial [Leptinotarsa decemlineata] | 47 | 1 |
| Gene.7615 | coatomer subunit epsilon [Anoplophora glabripennis] | 310 | 1 |
| Gene.7636 | PREDICTED: actin-related protein 2 isoform X2 [Aethina tumida] | 384 | 1 |
| Gene.7655 | ATP synthase subunit b, mitochondrial [Leptinotarsa decemlineata] | 203 | 1 |
| Gene.7655 | ATP synthase subunit b, mitochondrial [Leptinotarsa decemlineata] | 143 | 1 |
| Gene.7674 | histone deacetylase complex subunit SAP18 [Anoplophora glabripennis] | 116 | 1 |
| Gene.7693 | PREDICTED: tryptophan--tRNA ligase, cytoplasmic [Tribolium castaneum] | 41 | 1 |
| Gene.77 | cathepsin L [Anoplophora glabripennis] | 119 | 1 |
| Gene.77 | cathepsin L [Anoplophora glabripennis] | 49 | 1 |
| Gene.7715 | PREDICTED: phospholipid phosphatase 6 [Tribolium castaneum] | 25 | 1 |
| Gene.7734 | PREDICTED: LOW QUALITY PROTEIN: adenosylhomocysteinase [Aethina tumida] | 426 | 1 |
| Gene.7734 | PREDICTED: LOW QUALITY PROTEIN: adenosylhomocysteinase [Aethina tumida] | 318 | 1 |
| Gene.7771 | electron transfer flavoprotein subunit beta [Agrilus planipennis] | 174 | 1 |
| Gene.7808 | PREDICTED: THUMP domain-containing protein 1 homolog [Aethina tumida] | 83 | 1 |
| Gene.7824 | 60S ribosomal protein L37a [Agrilus planipennis] | 67 | 1 |
| Gene.7929 | PREDICTED: heat shock 70 kDa protein cognate 3 [Dendroctonus ponderosae] | 446 | 1 |
| Gene.7929 | PREDICTED: heat shock 70 kDa protein cognate 3 [Dendroctonus ponderosae] | 82 | 1 |
| Gene.7938 | PREDICTED: spermine synthase isoform X1 [Tribolium castaneum] | 187 | 1 |
| Gene.7938 | PREDICTED: spermine synthase isoform X1 [Tribolium castaneum] | 48 | 1 |
| Gene.7938 | PREDICTED: spermine synthase isoform X1 [Tribolium castaneum] | 153 | 1 |
| Gene.7943 | heat shock protein 21.56 [Harmonia axyridis] | 151 | 1 |
| Gene.7953 | protein ABHD11 isoform X1 [Anoplophora glabripennis] | 80 | 1 |
| Gene.7954 | eukaryotic translation initiation factor 3 subunit C [Anoplophora glabripennis] | 513 | 1 |
| Gene.7967 | uncharacterized protein LOC108916348 [Anoplophora glabripennis] | 56 | 1 |
| Gene.8017 | glycogen synthase [Harmonia axyridis] | 137 | 1 |
| Gene.8021 | ribosomal protein L4e [Biphyllus lunatus] | 339 | 1 |
| Gene.8021 | ribosomal protein L4e [Biphyllus lunatus] | 166 | 1 |
| Gene.8021 | ribosomal protein L4e [Biphyllus lunatus] | 178 | 1 |
| Gene.8021 | ribosomal protein L4e [Biphyllus lunatus] | 297 | 1 |
| Gene.8027 | uncharacterized protein LOC111502893 [Leptinotarsa decemlineata] | 40 | 1 |
| Gene.8046 | PREDICTED: sulfotransferase 1C4 [Tribolium castaneum] | 148 | 1 |
| Gene.8081 | ATP synthase subunit gamma, mitochondrial [Leptinotarsa decemlineata] | 143 | 1 |
| Gene.8087 | PREDICTED: adenylosuccinate synthetase-like [Aethina tumida] | 443 | 1 |
| Gene.8102 | glucosidase 2 subunit beta-like [Leptinotarsa decemlineata] | 397 | 1 |
| Gene.8102 | glucosidase 2 subunit beta-like [Leptinotarsa decemlineata] | 169 | 1 |
| Gene.8135 | PREDICTED: calreticulin [Tribolium castaneum] | 77 | 1 |
| Gene.814 | PREDICTED: uncharacterized protein LOC109597149 isoform X2 [Aethina tumida] | 75 | 1 |
| Gene.817 | PREDICTED: hydroxysteroid dehydrogenase-like protein 2 [Tribolium castaneum] | 62 | 1 |
| Gene.8272 | extracellular globin-E1-like isoform X1 [Leptinotarsa decemlineata] | 70 | 1 |
| Gene.8322 | PREDICTED: uncharacterized protein LOC109595590 [Aethina tumida] | 185 | 1 |
| Gene.8333 | PREDICTED: 60S ribosomal protein L5 [Tribolium castaneum] | 265 | 1 |
| Gene.8333 | PREDICTED: 60S ribosomal protein L5 [Tribolium castaneum] | 256 | 1 |
| Gene.8333 | PREDICTED: 60S ribosomal protein L5 [Tribolium castaneum] | 255 | 1 |
| Gene.834 | PREDICTED: very long-chain specific acyl-CoA dehydrogenase, mitochondrial [Tribolium castaneum] | 218 | 1 |
| Gene.8351 | probable cysteine desulfurase, mitochondrial [Anoplophora glabripennis] | 37 | 1 |
| Gene.8352 | PREDICTED: CUGBP Elav-like family member 1 isoform X1 [Tribolium castaneum] | 468 | 1 |
| Gene.8403 | PREDICTED: myosin heavy chain, non-muscle isoform X1 [Dendroctonus ponderosae] | 354 | 1 |
| Gene.8407 | PREDICTED: voltage-dependent anion-selective channel isoform X1 [Tribolium castaneum] | 87 | 1 |
| Gene.8408 | troponin C isoform X3 [Anoplophora glabripennis] | 97 | 1 |
| Gene.8427 | PREDICTED: EH domain-containing protein 3-like [Tribolium castaneum] | 462 | 1 |
| Gene.8432 | PREDICTED: laminin subunit alpha [Tribolium castaneum] | 388 | 1 |
| Gene.8474 | Uncharacterized protein | 253 | 1 |
| Gene.8507 | moesin/ezrin/radixin homolog 1 isoform X2 [Anoplophora glabripennis] | 162 | 1 |
| Gene.8516 | PREDICTED: NADH-ubiquinone oxidoreductase 49 kDa subunit [Tribolium castaneum] | 138 | 1 |
| Gene.8518 | PREDICTED: dolichyl-diphosphooligosaccharide--protein glycosyltransferase subunit 1 [Tribolium castaneum] | 114 | 1 |
| Gene.8518 | PREDICTED: dolichyl-diphosphooligosaccharide--protein glycosyltransferase subunit 1 [Tribolium castaneum] | 108 | 1 |
| Gene.8529 | NADH dehydrogenase [ubiquinone] 1 beta subcomplex subunit 7 [Anoplophora glabripennis] | 16 | 1 |
| Gene.8533 | PREDICTED: 60S acidic ribosomal protein P0 [Aethina tumida] | 66 | 1 |
| Gene.8571 | Innexin domain containing protein [Asbolus verrucosus] | 351 | 1 |
| Gene.8594 | PREDICTED: 60S ribosomal protein L35a [Tribolium castaneum] | 59 | 1 |
| Gene.8615 | uncharacterized protein BDFB_013993 [Asbolus verrucosus] | 63 | 1 |
| Gene.8619 | PREDICTED: LOW QUALITY PROTEIN: probable methylcrotonoyl-CoA carboxylase beta chain, mitochondrial [Aethina tumida] | 523 | 1 |
| Gene.8707 | PREDICTED: heterogeneous nuclear ribonucleoprotein Q isoform X1 [Tribolium castaneum] | 26 | 1 |
| Gene.8738 | activator of 90 kDa heat shock protein ATPase homolog 1 [Leptinotarsa decemlineata] | 216 | 1 |
| Gene.8756 | uncharacterized protein Dvir_GJ16722 [Drosophila virilis] | 58 | 1 |
| Gene.8787 | UDPGT domain containing protein [Asbolus verrucosus] | 97 | 1 |
| Gene.8793 | PREDICTED: nicalin-1 [Tribolium castaneum] | 469 | 1 |
| Gene.8819 | hydroxyacyl-coenzyme A dehydrogenase, mitochondrial [Anoplophora glabripennis] | 204 | 1 |
| Gene.8840 | signal recognition particle subunit SRP72 [Asbolus verrucosus] | 432 | 1 |
| Gene.8854 | clavesin-1 isoform X2 [Anoplophora glabripennis] | 99 | 1 |
| Gene.8873 | PREDICTED: fumarate hydratase, mitochondrial [Tribolium castaneum] | 52 | 1 |
| Gene.8903 | PREDICTED: T-complex protein 1 subunit epsilon [Tribolium castaneum] | 356 | 1 |
| Gene.8965 | putative pre-mRNA-splicing factor ATP-dependent RNA helicase PRP1 [Leptinotarsa decemlineata] | 528 | 1 |
| Gene.8967 | PREDICTED: ubiquitin-conjugating enzyme E2 variant 1 [Aethina tumida] | 68 | 1 |
| Gene.8976 | PREDICTED: putative esterase isoform X1 [Tribolium castaneum] | 523 | 1 |
| Gene.8976 | PREDICTED: putative esterase isoform X1 [Tribolium castaneum] | 47 | 1 |
| Gene.8989 | PREDICTED: aldehyde dehydrogenase, dimeric NADP-preferring-like [Aethina tumida] | 225 | 1 |
| Gene.8989 | PREDICTED: aldehyde dehydrogenase, dimeric NADP-preferring-like [Aethina tumida] | 201 | 1 |
| Gene.8998 | RecName: Full=Alpha-amylase; AltName: Full=1,4-alpha-D-glucan glucanohydrolase | 423 | 1 |
| Gene.9016 | lissencephaly-1 homolog [Leptinotarsa decemlineata] | 2 | 1 |
| Gene.9027 | uridine 5'-monophosphate synthase [Anoplophora glabripennis] | 25 | 1 |
| Gene.9057 | PREDICTED: pyrroline-5-carboxylate reductase [Tribolium castaneum] | 117 | 1 |
| Gene.922 | acetyl-CoA acetyltransferase, mitochondrial, partial [Asbolus verrucosus] | 72 | 1 |
| Gene.924 | 26S proteasome non-ATPase regulatory subunit 11 [Asbolus verrucosus] | 409 | 1 |
| Gene.97 | far upstream element-binding protein 1 isoform X1 [Anoplophora glabripennis] | 294 | 1 |
| Gene.1024 | annexin B9 isoform X2 [Anoplophora glabripennis] | 270 | 1 |
| Gene.1115 | cystathionine gamma-lyase [Agrilus planipennis] | 281 | 1 |
| Gene.1175 | PREDICTED: 26S proteasome non-ATPase regulatory subunit 12 [Aethina tumida] | 205 | 1 |
| Gene.1219 | 46 kDa FK506-binding nuclear protein [Anoplophora glabripennis] | 330 | 1 |
| Gene.1224 | PREDICTED: multidrug resistance protein 1 [Tribolium castaneum] | 387 | 1 |
| Gene.1245 | PREDICTED: uncharacterized protein LOC663129 [Tribolium castaneum] | 182 | 1 |
| Gene.1255 | purine nucleoside phosphorylase isoform X4 [Leptinotarsa decemlineata] | 158 | 1 |
| Gene.130 | PREDICTED: T-complex protein 1 subunit beta [Tribolium castaneum] | 169 | 1 |
| Gene.1303 | PREDICTED: UDP-glucuronosyltransferase 2B7 isoform X2 [Tribolium castaneum] | 330 | 1 |
| Gene.1319 | neurochondrin homolog [Anoplophora glabripennis] | 442 | 1 |
| Gene.1329 | PREDICTED: thioredoxin-2 [Dendroctonus ponderosae] | 47 | 1 |
| Gene.1391 | PREDICTED: protein lethal(2)essential for life [Tribolium castaneum] | 113 | 1 |
| Gene.1428 | PREDICTED: prostaglandin E synthase 3 [Nicrophorus vespilloides] | 97 | 1 |
| Gene.1476 | zinc finger protein on ecdysone puffs, partial [Asbolus verrucosus] | 222 | 1 |
| Gene.1479 | PREDICTED: venom serine carboxypeptidase [Tribolium castaneum] | 280 | 1 |
| Gene.1491 | PREDICTED: glucose-6-phosphate 1-dehydrogenase isoform X1 [Aethina tumida] | 444 | 1 |
| Gene.1532 | PREDICTED: glyoxalase domain-containing protein 4 [Tribolium castaneum] | 216 | 1 |
| Gene.1579 | putative gut cathepsin D-like aspartic protease [Callosobruchus maculatus] | 318 | 1 |
| Gene.1588 | cullin-associated NEDD8-dissociated protein 1 [Anoplophora glabripennis] | 40 | 1 |
| Gene.175 | PREDICTED: probable citrate synthase 2, mitochondrial [Tribolium castaneum] | 185 | 1 |
| Gene.1768 | PREDICTED: translation elongation factor 2 [Aethina tumida] | 424 | 1 |
| Gene.1806 | PREDICTED: xaa-Pro dipeptidase isoform X2 [Tribolium castaneum] | 115 | 1 |
| Gene.1914 | 3-hydroxyisobutyryl-CoA hydrolase, mitochondrial isoform X1 [Anoplophora glabripennis] | 340 | 1 |
| Gene.1917 | PREDICTED: calnexin [Tribolium castaneum] | 66 | 1 |
| Gene.1920 | transforming growth factor-beta-induced protein ig-h3 [Asbolus verrucosus] | 449 | 1 |
| Gene.2016 | PREDICTED: 40S ribosomal protein S4 isoform X1 [Aethina tumida] | 71 | 1 |
| Gene.2114 | PREDICTED: aminoacylase-1 [Tribolium castaneum] | 225 | 1 |
| Gene.2124 | PREDICTED: sorbitol dehydrogenase [Tribolium castaneum] | 274 | 1 |
| Gene.2124 | PREDICTED: sorbitol dehydrogenase [Tribolium castaneum] | 3 | 1 |
| Gene.2140 | PREDICTED: UDP-glucuronosyltransferase 2B10-like [Tribolium castaneum] | 419 | 1 |
| Gene.2167 | PREDICTED: 15-hydroxyprostaglandin dehydrogenase [NAD(+)] [Tribolium castaneum] | 220 | 1 |
| Gene.217 | putative DD34D transposase [Bactrocera tryoni] | 93 | 1 |
| Gene.2191 | eukaryotic initiation factor 3 p66 subunit [Tribolium castaneum] | 405 | 1 |
| Gene.2192 | small glutamine-rich tetratricopeptide repeat-containing protein alpha-like [Pseudomyrmex gracilis] | 7 | 1 |
| Gene.2195 | selenium-binding protein 1 [Asbolus verrucosus] | 279 | 1 |
| Gene.2227 | PREDICTED: ubiquitin-like modifier-activating enzyme 1 [Tribolium castaneum] | 821 | 1 |
| Gene.2317 | PREDICTED: myosin-2 essential light chain isoform X2 [Tribolium castaneum] | 26 | 1 |
| Gene.2343 | PREDICTED: UPF0047 protein YjbQ [Tribolium castaneum] | 17 | 1 |
| Gene.2357 | eukaryotic translation initiation factor 4 gamma 3-like, partial [Asbolus verrucosus] | 73 | 1 |
| Gene.2362 | uncharacterized protein LOC108904898 isoform X1 [Anoplophora glabripennis] | 59 | 1 |
| Gene.2365 | PREDICTED: maternal protein tudor isoform X1 [Tribolium castaneum] | 179 | 1 |
| Gene.2368 | PREDICTED: filamin-A isoform X6 [Tribolium castaneum] | 1367 | 1 |
| Gene.237 | PREDICTED: uncharacterized protein LOC661814 [Tribolium castaneum] | 224 | 1 |
| Gene.2372 | cytochrome P450 CYP9Z401 [Cryptolaemus montrouzieri] | 240 | 1 |
| Gene.2389 | superkiller viralicidic activity 2-like 2 [Anoplophora glabripennis] | 192 | 1 |
| Gene.2408 | PREDICTED: four and a half LIM domains protein 2 isoform X5 [Tribolium castaneum] | 211 | 1 |
| Gene.246 | PREDICTED: V-type proton ATPase subunit d [Tribolium castaneum] | 303 | 1 |
| Gene.2484 | PREDICTED: fatty-acid amide hydrolase 2-B [Tribolium castaneum] | 423 | 1 |
| Gene.2514 | Uncharacterized protein | 167 | 1 |
| Gene.2562 | hypothetical protein AMK59_6989 [Oryctes borbonicus] | 76 | 1 |
| Gene.2562 | hypothetical protein AMK59_6989 [Oryctes borbonicus] | 195 | 1 |
| Gene.2569 | 1,5-anhydro-D-fructose reductase-like Protein [Tribolium castaneum] | 300 | 1 |
| Gene.2615 | PREDICTED: coatomer subunit beta [Tribolium castaneum] | 32 | 1 |
| Gene.2650 | PREDICTED: succinyl-CoA ligase [ADP-forming] subunit beta, mitochondrial [Tribolium castaneum] | 274 | 1 |
| Gene.2656 | PREDICTED: aspartate aminotransferase, cytoplasmic [Tribolium castaneum] | 208 | 1 |
| Gene.2658 | PREDICTED: LOW QUALITY PROTEIN: CAD protein [Aethina tumida] | 62 | 1 |
| Gene.2689 | CoaE and/or CTP transf 2 domain containing protein [Asbolus verrucosus] | 262 | 1 |
| Gene.2724 | PREDICTED: endoplasmin [Tribolium castaneum] | 8 | 1 |
| Gene.2749 | PREDICTED: aldehyde dehydrogenase, mitochondrial [Tribolium castaneum] | 48 | 1 |
| Gene.2764 | PREDICTED: alpha-L-fucosidase [Nicrophorus vespilloides] | 216 | 1 |
| Gene.2764 | PREDICTED: alpha-L-fucosidase [Nicrophorus vespilloides] | 125 | 1 |
| Gene.2767 | ribosomal protein L10, partial [Harmonia axyridis] | 207 | 1 |
| Gene.2768 | probable peptidyl-tRNA hydrolase 2 [Anoplophora glabripennis] | 67 | 1 |
| Gene.2788 | aubergine [Diabrotica virgifera virgifera] | 796 | 1 |
| Gene.2830 | PREDICTED: LOW QUALITY PROTEIN: clathrin heavy chain [Aethina tumida] | 463 | 1 |
| Gene.2830 | PREDICTED: LOW QUALITY PROTEIN: clathrin heavy chain [Aethina tumida] | 771 | 1 |
| Gene.2857 | C-1-tetrahydrofolate synthase, cytoplasmic [Onthophagus taurus] | 585 | 1 |
| Gene.2862 | PREDICTED: troponin C, isoform 1 [Tribolium castaneum] | 122 | 1 |
| Gene.2889 | PREDICTED: ATP-binding cassette sub-family D member 3 [Tribolium castaneum] | 57 | 1 |
| Gene.2918 | plasminogen activator inhibitor 1 RNA-binding protein isoform X3 [Anoplophora glabripennis] | 77 | 1 |
| Gene.2958 | alpha actinin [Coleomegilla maculata] | 685 | 1 |
| Gene.3029 | PREDICTED: actin-interacting protein 1 isoform X2 [Tribolium castaneum] | 81 | 1 |
| Gene.3041 | U5 small nuclear ribonucleoprotein 200 kDa helicase [Asbolus verrucosus] | 80 | 1 |
| Gene.3063 | PREDICTED: uncharacterized protein LOC108560181 [Nicrophorus vespilloides] | 104 | 1 |
| Gene.3075 | dihydrolipoyl dehydrogenase, mitochondrial [Anoplophora glabripennis] | 151 | 1 |
| Gene.3133 | PREDICTED: 5'-AMP-activated protein kinase subunit gamma-2 isoform X1 [Tribolium castaneum] | 402 | 1 |
| Gene.316 | heat shock 70 kDa protein cognate 5-like protein [Epicauta chinensis] | 683 | 1 |
| Gene.316 | heat shock 70 kDa protein cognate 5-like protein [Epicauta chinensis] | 192 | 1 |
| Gene.3208 | phosphoglucose isomerase [Colias eurytheme] | 99 | 1 |
| Gene.3223 | Aldose reductase-like Protein [Tribolium castaneum] | 36 | 1 |
| Gene.3319 | protein CASC3 [Anoplophora glabripennis] | 374 | 1 |
| Gene.3389 | PREDICTED: uncharacterized protein LOC109594034 [Aethina tumida] | 125 | 1 |
| Gene.3417 | inter-alpha-trypsin inhibitor heavy chain H4-like, partial [Asbolus verrucosus] | 82 | 1 |
| Gene.342 | PREDICTED: 40S ribosomal protein S9 [Tribolium castaneum] | 122 | 1 |
| Gene.3447 | PREDICTED: congested-like trachea protein [Tribolium castaneum] | 262 | 1 |
| Gene.3489 | PREDICTED: acetyl-CoA carboxylase isoform X1 [Tribolium castaneum] | 1083 | 1 |
| Gene.3489 | PREDICTED: acetyl-CoA carboxylase isoform X1 [Tribolium castaneum] | 2412 | 1 |
| Gene.3489 | PREDICTED: acetyl-CoA carboxylase isoform X1 [Tribolium castaneum] | 1458 | 1 |
| Gene.3489 | PREDICTED: acetyl-CoA carboxylase isoform X1 [Tribolium castaneum] | 1966 | 1 |
| Gene.3489 | PREDICTED: acetyl-CoA carboxylase isoform X1 [Tribolium castaneum] | 2112 | 1 |
| Gene.3504 | cytochrome P450 monooxygenase CYP6BQ37 [Tenebrio molitor] | 434 | 1 |
| Gene.3518 | vitellogenin 2 [Harmonia axyridis] | 1665 | 1 |
| Gene.354 | saccharopine dehydrogenase-like [Asbolus verrucosus] | 164 | 1 |
| Gene.3547 | dolichyl-diphosphooligosaccharide--protein glycosyltransferase subunit 2 [Anoplophora glabripennis] | 33 | 1 |
| Gene.3548 | E3 SUMO-protein ligase RanBP2 [Anoplophora glabripennis] | 237 | 1 |
| Gene.3562 | vinculin isoform X1 [Anoplophora glabripennis] | 290 | 1 |
| Gene.3583 | 4-coumarate--CoA ligase-like [Leptinotarsa decemlineata] | 128 | 1 |
| Gene.3616 | PREDICTED: methionine-R-sulfoxide reductase B1 isoform X1 [Tribolium castaneum] | 109 | 1 |
| Gene.3620 | Ribosomal L19e domain containing protein [Asbolus verrucosus] | 5 | 1 |
| Gene.3628 | UDPGT and/or Glyco tran 28 C domain containing protein, partial [Asbolus verrucosus] | 71 | 1 |
| Gene.3628 | UDPGT and/or Glyco tran 28 C domain containing protein, partial [Asbolus verrucosus] | 425 | 1 |
| Gene.3651 | uncharacterized protein LOC108907894 isoform X4 [Anoplophora glabripennis] | 161 | 1 |
| Gene.367 | PREDICTED: uncharacterized protein LOC661974 [Tribolium castaneum] | 429 | 1 |
| Gene.3676 | hypothetical protein D910_04355 [Dendroctonus ponderosae] | 130 | 1 |
| Gene.3747 | piwi-like protein Ago3 [Anoplophora glabripennis] | 102 | 1 |
| Gene.3747 | piwi-like protein Ago3 [Anoplophora glabripennis] | 406 | 1 |
| Gene.3747 | piwi-like protein Ago3 [Anoplophora glabripennis] | 599 | 1 |
| Gene.3790 | PREDICTED: 60S ribosomal protein L3 [Tribolium castaneum] | 397 | 1 |
| Gene.3790 | PREDICTED: 60S ribosomal protein L3 [Tribolium castaneum] | 23 | 1 |
| Gene.3807 | scavenger receptor protein [Tribolium castaneum] | 191 | 1 |
| Gene.3807 | scavenger receptor protein [Tribolium castaneum] | 58 | 1 |
| Gene.3838 | PREDICTED: pyruvate carboxylase, mitochondrial isoform X3 [Tribolium castaneum] | 763 | 1 |
| Gene.3972 | pyruvate dehydrogenase E1 component subunit beta, mitochondrial [Leptinotarsa decemlineata] | 69 | 1 |
| Gene.4011 | fatty acid synthase [Coccinella septempunctata] | 2232 | 1 |
| Gene.4011 | fatty acid synthase [Coccinella septempunctata] | 1077 | 1 |
| Gene.4030 | uncharacterized protein BDFB_004854, partial [Asbolus verrucosus] | 24 | 1 |
| Gene.4067 | PREDICTED: programmed cell death 6-interacting protein [Tribolium castaneum] | 328 | 1 |
| Gene.4117 | PREDICTED: 4-coumarate--CoA ligase 1 [Tribolium castaneum] | 111 | 1 |
| Gene.4178 | mitochondrial 2-oxoglutarate/malate carrier protein [Asbolus verrucosus] | 152 | 1 |
| Gene.4190 | V-type proton ATPase subunit H [Asbolus verrucosus] | 351 | 1 |
| Gene.4285 | PREDICTED: LOW QUALITY PROTEIN: pyruvate kinase-like [Aethina tumida] | 489 | 1 |
| Gene.4303 | PREDICTED: LOW QUALITY PROTEIN: long-chain-fatty-acid--CoA ligase 4 [Aethina tumida] | 44 | 1 |
| Gene.4314 | UDP-glucuronosyltransferase 2C1-like, partial [Asbolus verrucosus] | 280 | 1 |
| Gene.4349 | PREDICTED: 2-hydroxyacylsphingosine 1-beta-galactosyltransferase-like [Tribolium castaneum] | 47 | 1 |
| Gene.4352 | PREDICTED: calcium-transporting ATPase sarcoplasmic/endoplasmic reticulum type isoform X1 [Tribolium castaneum] | 234 | 1 |
| Gene.4392 | Membrane metallo-endopeptidase-like 1, partial [Habropoda laboriosa] | 537 | 1 |
| Gene.4396 | PREDICTED: 2-hydroxyacylsphingosine 1-beta-galactosyltransferase-like [Tribolium castaneum] | 323 | 1 |
| Gene.4439 | Basement membrane-specific heparan sulfate proteoglycan core protein-like Protein [Tribolium castaneum] | 163 | 1 |
| Gene.4476 | Ribosomal S17e domain containing protein [Asbolus verrucosus] | 39 | 1 |
| Gene.4494 | alanine--tRNA ligase, cytoplasmic [Leptinotarsa decemlineata] | 109 | 1 |
| Gene.4495 | PREDICTED: LOW QUALITY PROTEIN: alanine--tRNA ligase, cytoplasmic [Aethina tumida] | 254 | 1 |
| Gene.4552 | PREDICTED: pseudouridine-5'-phosphatase [Tribolium castaneum] | 42 | 1 |
| Gene.4581 | PREDICTED: trifunctional enzyme subunit alpha, mitochondrial [Tribolium castaneum] | 354 | 1 |
| Gene.4592 | pre-mRNA-processing factor 39-like isoform X2 [Leptinotarsa decemlineata] | 36 | 1 |
| Gene.4641 | vigilin [Asbolus verrucosus] | 17 | 1 |
| Gene.4653 | Uncharacterized protein | 1195 | 1 |
| Gene.4653 | Uncharacterized protein | 1062 | 1 |
| Gene.4720 | hexamerin 3 precursor [Tribolium castaneum] | 46 | 1 |
| Gene.4786 | paramyosin, long form isoform X1 [Leptinotarsa decemlineata] | 232 | 1 |
[truncated: 40,472 more chars]
