# Supplementary material for: Qualitative lysine crotonylome analysis in the ovarian tissue of Harmonia axyridis (Pallas)
Source: PLoS One. 2021 Oct 18;16(10):e0258371. doi: 10.1371/journal.pone.0258371 (PMC8523065; doi:10.1371/journal.pone.0258371)
Supplement: S2 Table — (DOCX) [file pone.0258371.s004.docx]

**Table S2** The detailed information of the significantly enriched KEGG pathways

| **KEGG pathway** | **Protein accession** | **Position** | **Protein description** |
| --- | --- | --- | --- |
| map00790 Folate biosynthesis | Gene.5527 | 139 | dihydrofolate reductase [Asbolus verrucosus] |
| map00790 Folate biosynthesis | Gene.7644 | 70 | PREDICTED: aldose reductase [Tribolium castaneum] |
| map00790 Folate biosynthesis | Gene.7644 | 151 | PREDICTED: aldose reductase [Tribolium castaneum] |
| map00790 Folate biosynthesis | Gene.7644 | 61 | PREDICTED: aldose reductase [Tribolium castaneum] |
| map00790 Folate biosynthesis | Gene.3223 | 72 | Aldose reductase-like Protein [Tribolium castaneum] |
| map00790 Folate biosynthesis | Gene.3223 | 264 | Aldose reductase-like Protein [Tribolium castaneum] |
| map00790 Folate biosynthesis | Gene.3223 | 202 | Aldose reductase-like Protein [Tribolium castaneum] |
| map00790 Folate biosynthesis | Gene.3223 | 167 | Aldose reductase-like Protein [Tribolium castaneum] |
| map00790 Folate biosynthesis | Gene.2563 | 97 | PREDICTED: aldose reductase [Tribolium castaneum] |
| map00790 Folate biosynthesis | Gene.8139 | 247 | sepiapterin reductase [Anoplophora glabripennis] |
| map00790 Folate biosynthesis | Gene.7424 | 4 | carbonyl reductase [NADPH] 3 [Anoplophora glabripennis] |
| map00790 Folate biosynthesis | Gene.7424 | 72 | carbonyl reductase [NADPH] 3 [Anoplophora glabripennis] |
| map04010 MAPK signaling pathway | Gene.5709 | 26 | cAMP-dependent protein kinase catalytic subunit [Agrilus planipennis] |
| map04010 MAPK signaling pathway | Gene.5709 | 257 | cAMP-dependent protein kinase catalytic subunit [Agrilus planipennis] |
| map04010 MAPK signaling pathway | Gene.7526 | 96 | ras-related protein Rac1 [Anoplophora glabripennis] |
| map04010 MAPK signaling pathway | Gene.5869 | 105 | serine/threonine-protein phosphatase 5 [Anoplophora glabripennis] |
| map04010 MAPK signaling pathway | Gene.2368 | 2143 | PREDICTED: filamin-A isoform X6 [Tribolium castaneum] |
| map04010 MAPK signaling pathway | Gene.2368 | 1801 | PREDICTED: filamin-A isoform X6 [Tribolium castaneum] |
| map04010 MAPK signaling pathway | Gene.2368 | 585 | PREDICTED: filamin-A isoform X6 [Tribolium castaneum] |
| map04010 MAPK signaling pathway | Gene.2368 | 1534 | PREDICTED: filamin-A isoform X6 [Tribolium castaneum] |
| map04010 MAPK signaling pathway | Gene.2368 | 2141 | PREDICTED: filamin-A isoform X6 [Tribolium castaneum] |
| map04010 MAPK signaling pathway | Gene.2368 | 49 | PREDICTED: filamin-A isoform X6 [Tribolium castaneum] |
| map04010 MAPK signaling pathway | Gene.2368 | 867 | PREDICTED: filamin-A isoform X6 [Tribolium castaneum] |
| map04010 MAPK signaling pathway | Gene.2368 | 2100 | PREDICTED: filamin-A isoform X6 [Tribolium castaneum] |
| map04010 MAPK signaling pathway | Gene.1895 | 365 | heat shock protein 70 [Harmonia axyridis] |
| map04010 MAPK signaling pathway | Gene.1895 | 271 | heat shock protein 70 [Harmonia axyridis] |
| map04010 MAPK signaling pathway | Gene.1895 | 26 | heat shock protein 70 [Harmonia axyridis] |
| map04010 MAPK signaling pathway | Gene.1895 | 475 | heat shock protein 70 [Harmonia axyridis] |
| map04010 MAPK signaling pathway | Gene.1895 | 438 | heat shock protein 70 [Harmonia axyridis] |
| map04010 MAPK signaling pathway | Gene.1895 | 42 | heat shock protein 70 [Harmonia axyridis] |
| map04010 MAPK signaling pathway | Gene.1895 | 22 | heat shock protein 70 [Harmonia axyridis] |
| map04010 MAPK signaling pathway | Gene.1895 | 473 | heat shock protein 70 [Harmonia axyridis] |
| map04010 MAPK signaling pathway | Gene.1895 | 233 | heat shock protein 70 [Harmonia axyridis] |
| map04010 MAPK signaling pathway | Gene.1895 | 426 | heat shock protein 70 [Harmonia axyridis] |
| map04010 MAPK signaling pathway | Gene.1895 | 40 | heat shock protein 70 [Harmonia axyridis] |
| map04010 MAPK signaling pathway | Gene.1895 | 509 | heat shock protein 70 [Harmonia axyridis] |
| map04010 MAPK signaling pathway | Gene.1895 | 160 | heat shock protein 70 [Harmonia axyridis] |
| map04010 MAPK signaling pathway | Gene.1895 | 421 | heat shock protein 70 [Harmonia axyridis] |
| map04010 MAPK signaling pathway | Gene.1895 | 414 | heat shock protein 70 [Harmonia axyridis] |
| map04010 MAPK signaling pathway | Gene.1895 | 242 | heat shock protein 70 [Harmonia axyridis] |
| map04725 Cholinergic synapse | Gene.4205 | 535 | uncharacterized protein LOC108911898 [Anoplophora glabripennis] |
| map04725 Cholinergic synapse | Gene.4205 | 254 | uncharacterized protein LOC108911898 [Anoplophora glabripennis] |
| map04725 Cholinergic synapse | Gene.5709 | 26 | cAMP-dependent protein kinase catalytic subunit [Agrilus planipennis] |
| map04725 Cholinergic synapse | Gene.5709 | 257 | cAMP-dependent protein kinase catalytic subunit [Agrilus planipennis] |
| map04725 Cholinergic synapse | Gene.7676 | 53 | PREDICTED: guanine nucleotide-binding protein G(q) subunit alpha isoform X1 [Tribolium castaneum] |
| map04725 Cholinergic synapse | Gene.164 | 78 | PREDICTED: guanine nucleotide-binding protein subunit beta-1 [Tribolium castaneum] |
| map04910 Insulin signaling pathway | Gene.10 | 294 | PREDICTED: flotillin-1 isoform X2 [Aethina tumida] |
| map04910 Insulin signaling pathway | Gene.10 | 349 | PREDICTED: flotillin-1 isoform X2 [Aethina tumida] |
| map04910 Insulin signaling pathway | Gene.10 | 379 | PREDICTED: flotillin-1 isoform X2 [Aethina tumida] |
| map04910 Insulin signaling pathway | Gene.10 | 299 | PREDICTED: flotillin-1 isoform X2 [Aethina tumida] |
| map04910 Insulin signaling pathway | Gene.4419 | 291 | glycogen phosphorylase [Harmonia axyridis] |
| map04910 Insulin signaling pathway | Gene.4419 | 177 | glycogen phosphorylase [Harmonia axyridis] |
| map04910 Insulin signaling pathway | Gene.4419 | 10 | glycogen phosphorylase [Harmonia axyridis] |
| map04910 Insulin signaling pathway | Gene.4419 | 29 | glycogen phosphorylase [Harmonia axyridis] |
| map04910 Insulin signaling pathway | Gene.4419 | 249 | glycogen phosphorylase [Harmonia axyridis] |
| map04910 Insulin signaling pathway | Gene.4419 | 78 | glycogen phosphorylase [Harmonia axyridis] |
| map04910 Insulin signaling pathway | Gene.4011 | 1002 | fatty acid synthase [Coccinella septempunctata] |
| map04910 Insulin signaling pathway | Gene.4011 | 2094 | fatty acid synthase [Coccinella septempunctata] |
| map04910 Insulin signaling pathway | Gene.4011 | 503 | fatty acid synthase [Coccinella septempunctata] |
| map04910 Insulin signaling pathway | Gene.4011 | 2077 | fatty acid synthase [Coccinella septempunctata] |
| map04910 Insulin signaling pathway | Gene.4011 | 1744 | fatty acid synthase [Coccinella septempunctata] |
| map04910 Insulin signaling pathway | Gene.4011 | 1597 | fatty acid synthase [Coccinella septempunctata] |
| map04910 Insulin signaling pathway | Gene.4011 | 987 | fatty acid synthase [Coccinella septempunctata] |
| map04910 Insulin signaling pathway | Gene.4011 | 2371 | fatty acid synthase [Coccinella septempunctata] |
| map04910 Insulin signaling pathway | Gene.4011 | 1892 | fatty acid synthase [Coccinella septempunctata] |
| map04910 Insulin signaling pathway | Gene.4011 | 1887 | fatty acid synthase [Coccinella septempunctata] |
| map04910 Insulin signaling pathway | Gene.4011 | 460 | fatty acid synthase [Coccinella septempunctata] |
| map04910 Insulin signaling pathway | Gene.4011 | 990 | fatty acid synthase [Coccinella septempunctata] |
| map04910 Insulin signaling pathway | Gene.4011 | 2121 | fatty acid synthase [Coccinella septempunctata] |
| map04910 Insulin signaling pathway | Gene.4011 | 1016 | fatty acid synthase [Coccinella septempunctata] |
| map04910 Insulin signaling pathway | Gene.4011 | 707 | fatty acid synthase [Coccinella septempunctata] |
| map04910 Insulin signaling pathway | Gene.4011 | 526 | fatty acid synthase [Coccinella septempunctata] |
| map04910 Insulin signaling pathway | Gene.4011 | 494 | fatty acid synthase [Coccinella septempunctata] |
| map04910 Insulin signaling pathway | Gene.4011 | 674 | fatty acid synthase [Coccinella septempunctata] |
| map04910 Insulin signaling pathway | Gene.4011 | 1398 | fatty acid synthase [Coccinella septempunctata] |
| map04910 Insulin signaling pathway | Gene.4011 | 998 | fatty acid synthase [Coccinella septempunctata] |
| map04910 Insulin signaling pathway | Gene.4011 | 273 | fatty acid synthase [Coccinella septempunctata] |
| map04910 Insulin signaling pathway | Gene.4011 | 1122 | fatty acid synthase [Coccinella septempunctata] |
| map04910 Insulin signaling pathway | Gene.4011 | 1881 | fatty acid synthase [Coccinella septempunctata] |
| map04910 Insulin signaling pathway | Gene.4011 | 1740 | fatty acid synthase [Coccinella septempunctata] |
| map04910 Insulin signaling pathway | Gene.4011 | 699 | fatty acid synthase [Coccinella septempunctata] |
| map04910 Insulin signaling pathway | Gene.4011 | 450 | fatty acid synthase [Coccinella septempunctata] |
| map04910 Insulin signaling pathway | Gene.4011 | 1453 | fatty acid synthase [Coccinella septempunctata] |
| map04910 Insulin signaling pathway | Gene.3489 | 2124 | PREDICTED: acetyl-CoA carboxylase isoform X1 [Tribolium castaneum] |
| map04910 Insulin signaling pathway | Gene.3489 | 2067 | PREDICTED: acetyl-CoA carboxylase isoform X1 [Tribolium castaneum] |
| map04910 Insulin signaling pathway | Gene.3489 | 387 | PREDICTED: acetyl-CoA carboxylase isoform X1 [Tribolium castaneum] |
| map04910 Insulin signaling pathway | Gene.3489 | 1848 | PREDICTED: acetyl-CoA carboxylase isoform X1 [Tribolium castaneum] |
| map04910 Insulin signaling pathway | Gene.3489 | 2305 | PREDICTED: acetyl-CoA carboxylase isoform X1 [Tribolium castaneum] |
| map04910 Insulin signaling pathway | Gene.3489 | 341 | PREDICTED: acetyl-CoA carboxylase isoform X1 [Tribolium castaneum] |
| map04910 Insulin signaling pathway | Gene.3489 | 167 | PREDICTED: acetyl-CoA carboxylase isoform X1 [Tribolium castaneum] |
| map04910 Insulin signaling pathway | Gene.3489 | 1964 | PREDICTED: acetyl-CoA carboxylase isoform X1 [Tribolium castaneum] |
| map04910 Insulin signaling pathway | Gene.3489 | 1645 | PREDICTED: acetyl-CoA carboxylase isoform X1 [Tribolium castaneum] |
| map04910 Insulin signaling pathway | Gene.3489 | 1389 | PREDICTED: acetyl-CoA carboxylase isoform X1 [Tribolium castaneum] |
| map04910 Insulin signaling pathway | Gene.3489 | 2131 | PREDICTED: acetyl-CoA carboxylase isoform X1 [Tribolium castaneum] |
| map04910 Insulin signaling pathway | Gene.3489 | 179 | PREDICTED: acetyl-CoA carboxylase isoform X1 [Tribolium castaneum] |
| map04910 Insulin signaling pathway | Gene.3489 | 383 | PREDICTED: acetyl-CoA carboxylase isoform X1 [Tribolium castaneum] |
| map04910 Insulin signaling pathway | Gene.3489 | 1820 | PREDICTED: acetyl-CoA carboxylase isoform X1 [Tribolium castaneum] |
| map04910 Insulin signaling pathway | Gene.3489 | 1264 | PREDICTED: acetyl-CoA carboxylase isoform X1 [Tribolium castaneum] |
| map04910 Insulin signaling pathway | Gene.3489 | 1412 | PREDICTED: acetyl-CoA carboxylase isoform X1 [Tribolium castaneum] |
| map04910 Insulin signaling pathway | Gene.3489 | 2235 | PREDICTED: acetyl-CoA carboxylase isoform X1 [Tribolium castaneum] |
| map04910 Insulin signaling pathway | Gene.3489 | 746 | PREDICTED: acetyl-CoA carboxylase isoform X1 [Tribolium castaneum] |
| map04910 Insulin signaling pathway | Gene.3489 | 2342 | PREDICTED: acetyl-CoA carboxylase isoform X1 [Tribolium castaneum] |
| map04910 Insulin signaling pathway | Gene.3489 | 1379 | PREDICTED: acetyl-CoA carboxylase isoform X1 [Tribolium castaneum] |
| map04910 Insulin signaling pathway | Gene.3489 | 1369 | PREDICTED: acetyl-CoA carboxylase isoform X1 [Tribolium castaneum] |
| map04910 Insulin signaling pathway | Gene.3489 | 2245 | PREDICTED: acetyl-CoA carboxylase isoform X1 [Tribolium castaneum] |
| map04910 Insulin signaling pathway | Gene.5803 | 20 | calcium-binding protein E63-1 [Anoplophora glabripennis] |
| map04910 Insulin signaling pathway | Gene.1533 | 280 | PREDICTED: hexokinase type 2 isoform X2 [Tribolium castaneum] |
| map04910 Insulin signaling pathway | Gene.1533 | 73 | PREDICTED: hexokinase type 2 isoform X2 [Tribolium castaneum] |
| map04910 Insulin signaling pathway | Gene.502 | 147 | "acid phosphatase, partial [Cryptolaemus montrouzieri]" |
| map04910 Insulin signaling pathway | Gene.502 | 141 | "acid phosphatase, partial [Cryptolaemus montrouzieri]" |
| map04910 Insulin signaling pathway | Gene.502 | 260 | "acid phosphatase, partial [Cryptolaemus montrouzieri]" |
| map04910 Insulin signaling pathway | Gene.5709 | 26 | cAMP-dependent protein kinase catalytic subunit [Agrilus planipennis] |
| map04910 Insulin signaling pathway | Gene.5709 | 257 | cAMP-dependent protein kinase catalytic subunit [Agrilus planipennis] |
| map04910 Insulin signaling pathway | Gene.298 | 116 | "Csa-calmodulin 3, partial [Cupiennius salei]" |
| map04910 Insulin signaling pathway | Gene.298 | 95 | "Csa-calmodulin 3, partial [Cupiennius salei]" |
| map04910 Insulin signaling pathway | Gene.298 | 31 | "Csa-calmodulin 3, partial [Cupiennius salei]" |
| map04910 Insulin signaling pathway | Gene.298 | 78 | "Csa-calmodulin 3, partial [Cupiennius salei]" |
| map04910 Insulin signaling pathway | Gene.1482 | 344 | flotillin-2 [Onthophagus taurus] |
| map04910 Insulin signaling pathway | Gene.1482 | 335 | flotillin-2 [Onthophagus taurus] |
| map04910 Insulin signaling pathway | Gene.1482 | 211 | flotillin-2 [Onthophagus taurus] |
| map04910 Insulin signaling pathway | Gene.1845 | 92 | PREDICTED: glycogen synthase kinase-3 beta isoform X10 [Tribolium castaneum] |
| map04910 Insulin signaling pathway | Gene.1845 | 87 | PREDICTED: glycogen synthase kinase-3 beta isoform X10 [Tribolium castaneum] |
| map04910 Insulin signaling pathway | Gene.919 | 149 | ribosomal protein S6 [Chrysomela tremula] |
| map04910 Insulin signaling pathway | Gene.919 | 159 | ribosomal protein S6 [Chrysomela tremula] |
| map04910 Insulin signaling pathway | Gene.919 | 165 | ribosomal protein S6 [Chrysomela tremula] |
| map04910 Insulin signaling pathway | Gene.919 | 51 | ribosomal protein S6 [Chrysomela tremula] |
| map04910 Insulin signaling pathway | Gene.919 | 58 | ribosomal protein S6 [Chrysomela tremula] |
| map04910 Insulin signaling pathway | Gene.919 | 211 | ribosomal protein S6 [Chrysomela tremula] |
| map04910 Insulin signaling pathway | Gene.919 | 14 | ribosomal protein S6 [Chrysomela tremula] |
| map04910 Insulin signaling pathway | Gene.919 | 23 | ribosomal protein S6 [Chrysomela tremula] |
| map04910 Insulin signaling pathway | Gene.8017 | 315 | glycogen synthase [Harmonia axyridis] |
| map04910 Insulin signaling pathway | Gene.8017 | 298 | glycogen synthase [Harmonia axyridis] |
| map04910 Insulin signaling pathway | Gene.8017 | 247 | glycogen synthase [Harmonia axyridis] |
| map04910 Insulin signaling pathway | Gene.4417 | 92 | glycogen phosphorylase [Harmonia axyridis] |
| map04910 Insulin signaling pathway | Gene.4417 | 205 | glycogen phosphorylase [Harmonia axyridis] |
| map04910 Insulin signaling pathway | Gene.4417 | 120 | glycogen phosphorylase [Harmonia axyridis] |
| map04910 Insulin signaling pathway | Gene.4417 | 134 | glycogen phosphorylase [Harmonia axyridis] |
| map04910 Insulin signaling pathway | Gene.4417 | 44 | glycogen phosphorylase [Harmonia axyridis] |
| map04910 Insulin signaling pathway | Gene.4417 | 288 | glycogen phosphorylase [Harmonia axyridis] |
| map04910 Insulin signaling pathway | Gene.4417 | 113 | glycogen phosphorylase [Harmonia axyridis] |
| map04910 Insulin signaling pathway | Gene.3247 | 93 | PREDICTED: phosphoenolpyruvate carboxykinase [GTP] isoform X1 [Tribolium castaneum] |
| map05412 Arrhythmogenic right ventricular cardiomyopathy (ARVC) | Gene.4352 | 128 | PREDICTED: calcium-transporting ATPase sarcoplasmic/endoplasmic reticulum type isoform X1 [Tribolium castaneum] |
| map05412 Arrhythmogenic right ventricular cardiomyopathy (ARVC) | Gene.4352 | 514 | PREDICTED: calcium-transporting ATPase sarcoplasmic/endoplasmic reticulum type isoform X1 [Tribolium castaneum] |
| map05412 Arrhythmogenic right ventricular cardiomyopathy (ARVC) | Gene.4352 | 30 | PREDICTED: calcium-transporting ATPase sarcoplasmic/endoplasmic reticulum type isoform X1 [Tribolium castaneum] |
| map05412 Arrhythmogenic right ventricular cardiomyopathy (ARVC) | Gene.172 | 216 | "beta-actin, partial [Cotesia chilonis]" |
| map05412 Arrhythmogenic right ventricular cardiomyopathy (ARVC) | Gene.172 | 51 | "beta-actin, partial [Cotesia chilonis]" |
| map05412 Arrhythmogenic right ventricular cardiomyopathy (ARVC) | Gene.172 | 114 | "beta-actin, partial [Cotesia chilonis]" |
| map05412 Arrhythmogenic right ventricular cardiomyopathy (ARVC) | Gene.172 | 62 | "beta-actin, partial [Cotesia chilonis]" |
| map05412 Arrhythmogenic right ventricular cardiomyopathy (ARVC) | Gene.2646 | 99 | beta actin [Polyrhachis vicina] |
| map05412 Arrhythmogenic right ventricular cardiomyopathy (ARVC) | Gene.2646 | 88 | beta actin [Polyrhachis vicina] |
| map05412 Arrhythmogenic right ventricular cardiomyopathy (ARVC) | Gene.2646 | 64 | beta actin [Polyrhachis vicina] |
| map05412 Arrhythmogenic right ventricular cardiomyopathy (ARVC) | Gene.2646 | 101 | beta actin [Polyrhachis vicina] |
| map04810 Regulation of actin cytoskeleton | Gene.8288 | 19 | cofilin/actin-depolymerizing factor homolog [Anoplophora glabripennis] |
| map04810 Regulation of actin cytoskeleton | Gene.8288 | 41 | cofilin/actin-depolymerizing factor homolog [Anoplophora glabripennis] |
| map04810 Regulation of actin cytoskeleton | Gene.1564 | 40 | chickadee [Tribolium castaneum] |
| map04810 Regulation of actin cytoskeleton | Gene.7526 | 96 | ras-related protein Rac1 [Anoplophora glabripennis] |
| map04810 Regulation of actin cytoskeleton | Gene.2958 | 401 | alpha actinin [Coleomegilla maculata] |
| map04810 Regulation of actin cytoskeleton | Gene.2958 | 763 | alpha actinin [Coleomegilla maculata] |
| map04810 Regulation of actin cytoskeleton | Gene.502 | 147 | "acid phosphatase, partial [Cryptolaemus montrouzieri]" |
| map04810 Regulation of actin cytoskeleton | Gene.502 | 141 | "acid phosphatase, partial [Cryptolaemus montrouzieri]" |
| map04810 Regulation of actin cytoskeleton | Gene.502 | 260 | "acid phosphatase, partial [Cryptolaemus montrouzieri]" |
| map04810 Regulation of actin cytoskeleton | Gene.172 | 216 | "beta-actin, partial [Cotesia chilonis]" |
| map04810 Regulation of actin cytoskeleton | Gene.172 | 51 | "beta-actin, partial [Cotesia chilonis]" |
| map04810 Regulation of actin cytoskeleton | Gene.172 | 114 | "beta-actin, partial [Cotesia chilonis]" |
| map04810 Regulation of actin cytoskeleton | Gene.172 | 62 | "beta-actin, partial [Cotesia chilonis]" |
| map04810 Regulation of actin cytoskeleton | Gene.2646 | 99 | beta actin [Polyrhachis vicina] |
| map04810 Regulation of actin cytoskeleton | Gene.2646 | 88 | beta actin [Polyrhachis vicina] |
| map04810 Regulation of actin cytoskeleton | Gene.2646 | 64 | beta actin [Polyrhachis vicina] |
| map04810 Regulation of actin cytoskeleton | Gene.2646 | 101 | beta actin [Polyrhachis vicina] |
| map05010 Alzheimer disease | Gene.1542 | 237 | "PREDICTED: ATP synthase subunit alpha, mitochondrial [Tribolium castaneum]" |
| map05010 Alzheimer disease | Gene.1542 | 130 | "PREDICTED: ATP synthase subunit alpha, mitochondrial [Tribolium castaneum]" |
| map05010 Alzheimer disease | Gene.1542 | 63 | "PREDICTED: ATP synthase subunit alpha, mitochondrial [Tribolium castaneum]" |
| map05010 Alzheimer disease | Gene.1542 | 165 | "PREDICTED: ATP synthase subunit alpha, mitochondrial [Tribolium castaneum]" |
| map05010 Alzheimer disease | Gene.1542 | 71 | "PREDICTED: ATP synthase subunit alpha, mitochondrial [Tribolium castaneum]" |
| map05010 Alzheimer disease | Gene.1542 | 314 | "PREDICTED: ATP synthase subunit alpha, mitochondrial [Tribolium castaneum]" |
| map05010 Alzheimer disease | Gene.1542 | 537 | "PREDICTED: ATP synthase subunit alpha, mitochondrial [Tribolium castaneum]" |
| map05010 Alzheimer disease | Gene.1542 | 514 | "PREDICTED: ATP synthase subunit alpha, mitochondrial [Tribolium castaneum]" |
| map05010 Alzheimer disease | Gene.1542 | 526 | "PREDICTED: ATP synthase subunit alpha, mitochondrial [Tribolium castaneum]" |
| map05010 Alzheimer disease | Gene.1542 | 422 | "PREDICTED: ATP synthase subunit alpha, mitochondrial [Tribolium castaneum]" |
| map05010 Alzheimer disease | Gene.1542 | 159 | "PREDICTED: ATP synthase subunit alpha, mitochondrial [Tribolium castaneum]" |
| map05010 Alzheimer disease | Gene.1542 | 228 | "PREDICTED: ATP synthase subunit alpha, mitochondrial [Tribolium castaneum]" |
| map05010 Alzheimer disease | Gene.1542 | 303 | "PREDICTED: ATP synthase subunit alpha, mitochondrial [Tribolium castaneum]" |
| map05010 Alzheimer disease | Gene.1542 | 518 | "PREDICTED: ATP synthase subunit alpha, mitochondrial [Tribolium castaneum]" |
| map05010 Alzheimer disease | Gene.1542 | 425 | "PREDICTED: ATP synthase subunit alpha, mitochondrial [Tribolium castaneum]" |
| map05010 Alzheimer disease | Gene.1542 | 501 | "PREDICTED: ATP synthase subunit alpha, mitochondrial [Tribolium castaneum]" |
| map05010 Alzheimer disease | Gene.1542 | 529 | "PREDICTED: ATP synthase subunit alpha, mitochondrial [Tribolium castaneum]" |
| map05010 Alzheimer disease | Gene.1542 | 432 | "PREDICTED: ATP synthase subunit alpha, mitochondrial [Tribolium castaneum]" |
| map05010 Alzheimer disease | Gene.1542 | 507 | "PREDICTED: ATP synthase subunit alpha, mitochondrial [Tribolium castaneum]" |
| map05010 Alzheimer disease | Gene.1542 | 496 | "PREDICTED: ATP synthase subunit alpha, mitochondrial [Tribolium castaneum]" |
| map05010 Alzheimer disease | Gene.5910 | 270 | "PREDICTED: NADH dehydrogenase [ubiquinone] 1 alpha subcomplex subunit 10, mitochondrial [Tribolium castaneum]" |
| map05010 Alzheimer disease | Gene.742 | 137 | "NADH dehydrogenase [ubiquinone] 1 alpha subcomplex subunit 9, mitochondrial [Anoplophora glabripennis]" |
| map05010 Alzheimer disease | Gene.742 | 278 | "NADH dehydrogenase [ubiquinone] 1 alpha subcomplex subunit 9, mitochondrial [Anoplophora glabripennis]" |
| map05010 Alzheimer disease | Gene.742 | 142 | "NADH dehydrogenase [ubiquinone] 1 alpha subcomplex subunit 9, mitochondrial [Anoplophora glabripennis]" |
| map05010 Alzheimer disease | Gene.742 | 268 | "NADH dehydrogenase [ubiquinone] 1 alpha subcomplex subunit 9, mitochondrial [Anoplophora glabripennis]" |
| map05010 Alzheimer disease | Gene.5885 | 94 | NADH dehydrogenase [ubiquinone] 1 alpha subcomplex subunit 6 [Onthophagus taurus] |
| map05010 Alzheimer disease | Gene.7655 | 158 | "ATP synthase subunit b, mitochondrial [Leptinotarsa decemlineata]" |
| map05010 Alzheimer disease | Gene.7655 | 119 | "ATP synthase subunit b, mitochondrial [Leptinotarsa decemlineata]" |
| map05010 Alzheimer disease | Gene.7655 | 170 | "ATP synthase subunit b, mitochondrial [Leptinotarsa decemlineata]" |
| map05010 Alzheimer disease | Gene.7655 | 233 | "ATP synthase subunit b, mitochondrial [Leptinotarsa decemlineata]" |
| map05010 Alzheimer disease | Gene.7655 | 154 | "ATP synthase subunit b, mitochondrial [Leptinotarsa decemlineata]" |
| map05010 Alzheimer disease | Gene.7655 | 53 | "ATP synthase subunit b, mitochondrial [Leptinotarsa decemlineata]" |
| map05010 Alzheimer disease | Gene.7655 | 194 | "ATP synthase subunit b, mitochondrial [Leptinotarsa decemlineata]" |
| map05010 Alzheimer disease | Gene.143 | 69 | cytochrome c oxidase subunit IV [Tribolium castaneum] |
| map05010 Alzheimer disease | Gene.143 | 180 | cytochrome c oxidase subunit IV [Tribolium castaneum] |
| map05010 Alzheimer disease | Gene.143 | 94 | cytochrome c oxidase subunit IV [Tribolium castaneum] |
| map05010 Alzheimer disease | Gene.143 | 184 | cytochrome c oxidase subunit IV [Tribolium castaneum] |
| map05010 Alzheimer disease | Gene.2034 | 471 | "NADH dehydrogenase [ubiquinone] flavoprotein 1, mitochondrial [Tribolium castaneum]" |
| map05010 Alzheimer disease | Gene.2034 | 381 | "NADH dehydrogenase [ubiquinone] flavoprotein 1, mitochondrial [Tribolium castaneum]" |
| map05010 Alzheimer disease | Gene.2034 | 47 | "NADH dehydrogenase [ubiquinone] flavoprotein 1, mitochondrial [Tribolium castaneum]" |
| map05010 Alzheimer disease | Gene.8016 | 180 | "UCR TM, Rieske, and/or Ubiq-Cytc-red N domain containing protein [Asbolus verrucosus]" |
| map05010 Alzheimer disease | Gene.8016 | 171 | "UCR TM, Rieske, and/or Ubiq-Cytc-red N domain containing protein [Asbolus verrucosus]" |
| map05010 Alzheimer disease | Gene.8016 | 110 | "UCR TM, Rieske, and/or Ubiq-Cytc-red N domain containing protein [Asbolus verrucosus]" |
| map05010 Alzheimer disease | Gene.8016 | 176 | "UCR TM, Rieske, and/or Ubiq-Cytc-red N domain containing protein [Asbolus verrucosus]" |
| map05010 Alzheimer disease | Gene.6135 | 95 | PREDICTED: NADH dehydrogenase [ubiquinone] 1 alpha subcomplex subunit 13-like [Aethina tumida] |
| map05010 Alzheimer disease | Gene.2375 | 84 | "glyceraldehyde-3-phosphate, partial [Harmonia axyridis]" |
| map05010 Alzheimer disease | Gene.2375 | 252 | "glyceraldehyde-3-phosphate, partial [Harmonia axyridis]" |
| map05010 Alzheimer disease | Gene.2375 | 90 | "glyceraldehyde-3-phosphate, partial [Harmonia axyridis]" |
| map05010 Alzheimer disease | Gene.2375 | 228 | "glyceraldehyde-3-phosphate, partial [Harmonia axyridis]" |
| map05010 Alzheimer disease | Gene.2375 | 330 | "glyceraldehyde-3-phosphate, partial [Harmonia axyridis]" |
| map05010 Alzheimer disease | Gene.2375 | 58 | "glyceraldehyde-3-phosphate, partial [Harmonia axyridis]" |
| map05010 Alzheimer disease | Gene.2375 | 163 | "glyceraldehyde-3-phosphate, partial [Harmonia axyridis]" |
| map05010 Alzheimer disease | Gene.2375 | 66 | "glyceraldehyde-3-phosphate, partial [Harmonia axyridis]" |
| map05010 Alzheimer disease | Gene.2375 | 73 | "glyceraldehyde-3-phosphate, partial [Harmonia axyridis]" |
| map05010 Alzheimer disease | Gene.2375 | 260 | "glyceraldehyde-3-phosphate, partial [Harmonia axyridis]" |
| map05010 Alzheimer disease | Gene.2375 | 264 | "glyceraldehyde-3-phosphate, partial [Harmonia axyridis]" |
| map05010 Alzheimer disease | Gene.2375 | 223 | "glyceraldehyde-3-phosphate, partial [Harmonia axyridis]" |
| map05010 Alzheimer disease | Gene.2375 | 195 | "glyceraldehyde-3-phosphate, partial [Harmonia axyridis]" |
| map05010 Alzheimer disease | Gene.7840 | 189 | "PREDICTED: cytochrome c1, heme protein, mitochondrial [Tribolium castaneum]" |
| map05010 Alzheimer disease | Gene.7840 | 44 | "PREDICTED: cytochrome c1, heme protein, mitochondrial [Tribolium castaneum]" |
| map05010 Alzheimer disease | Gene.8756 | 84 | uncharacterized protein Dvir_GJ16722 [Drosophila virilis] |
| map05010 Alzheimer disease | Gene.8756 | 10 | uncharacterized protein Dvir_GJ16722 [Drosophila virilis] |
| map05010 Alzheimer disease | Gene.8261 | 92 | PREDICTED: cytochrome c oxidase subunit 6C-like [Dendroctonus ponderosae] |
| map05010 Alzheimer disease | Gene.8261 | 88 | PREDICTED: cytochrome c oxidase subunit 6C-like [Dendroctonus ponderosae] |
| map05010 Alzheimer disease | Gene.8261 | 40 | PREDICTED: cytochrome c oxidase subunit 6C-like [Dendroctonus ponderosae] |
| map05010 Alzheimer disease | Gene.5746 | 137 | "PREDICTED: ATP synthase subunit delta, mitochondrial [Tribolium castaneum]" |
| map05010 Alzheimer disease | Gene.7676 | 53 | PREDICTED: guanine nucleotide-binding protein G(q) subunit alpha isoform X1 [Tribolium castaneum] |
| map05010 Alzheimer disease | Gene.8081 | 119 | "ATP synthase subunit gamma, mitochondrial [Leptinotarsa decemlineata]" |
| map05010 Alzheimer disease | Gene.8081 | 125 | "ATP synthase subunit gamma, mitochondrial [Leptinotarsa decemlineata]" |
| map05010 Alzheimer disease | Gene.8081 | 111 | "ATP synthase subunit gamma, mitochondrial [Leptinotarsa decemlineata]" |
| map05010 Alzheimer disease | Gene.8081 | 78 | "ATP synthase subunit gamma, mitochondrial [Leptinotarsa decemlineata]" |
| map05010 Alzheimer disease | Gene.8081 | 54 | "ATP synthase subunit gamma, mitochondrial [Leptinotarsa decemlineata]" |
| map05010 Alzheimer disease | Gene.6318 | 57 | NADH dehydrogenase [ubiquinone] 1 beta subcomplex subunit 3 [Leptinotarsa decemlineata] |
| map05010 Alzheimer disease | Gene.6318 | 43 | NADH dehydrogenase [ubiquinone] 1 beta subcomplex subunit 3 [Leptinotarsa decemlineata] |
| map05010 Alzheimer disease | Gene.6318 | 23 | NADH dehydrogenase [ubiquinone] 1 beta subcomplex subunit 3 [Leptinotarsa decemlineata] |
| map05010 Alzheimer disease | Gene.5803 | 20 | calcium-binding protein E63-1 [Anoplophora glabripennis] |
| map05010 Alzheimer disease | Gene.8337 | 50 | "PREDICTED: cytochrome c oxidase subunit 5B, mitochondrial [Tribolium castaneum]" |
| map05010 Alzheimer disease | Gene.8337 | 44 | "PREDICTED: cytochrome c oxidase subunit 5B, mitochondrial [Tribolium castaneum]" |
| map05010 Alzheimer disease | Gene.6252 | 60 | NADH dehydrogenase [ubiquinone] 1 beta subcomplex subunit 4 [Leptinotarsa decemlineata] |
| map05010 Alzheimer disease | Gene.6252 | 111 | NADH dehydrogenase [ubiquinone] 1 beta subcomplex subunit 4 [Leptinotarsa decemlineata] |
| map05010 Alzheimer disease | Gene.2624 | 70 | PREDICTED: NADH dehydrogenase [ubiquinone] 1 alpha subcomplex subunit 5 [Tribolium castaneum] |
| map05010 Alzheimer disease | Gene.298 | 116 | "Csa-calmodulin 3, partial [Cupiennius salei]" |
| map05010 Alzheimer disease | Gene.298 | 95 | "Csa-calmodulin 3, partial [Cupiennius salei]" |
| map05010 Alzheimer disease | Gene.298 | 31 | "Csa-calmodulin 3, partial [Cupiennius salei]" |
| map05010 Alzheimer disease | Gene.298 | 78 | "Csa-calmodulin 3, partial [Cupiennius salei]" |
| map05010 Alzheimer disease | Gene.6262 | 104 | "probable NADH dehydrogenase [ubiquinone] flavoprotein 2, mitochondrial [Leptinotarsa decemlineata]" |
| map05010 Alzheimer disease | Gene.6262 | 230 | "probable NADH dehydrogenase [ubiquinone] flavoprotein 2, mitochondrial [Leptinotarsa decemlineata]" |
| map05010 Alzheimer disease | Gene.1073 | 88 | "PREDICTED: succinate dehydrogenase [ubiquinone] flavoprotein subunit, mitochondrial isoform X1 [Tribolium castaneum]" |
| map05010 Alzheimer disease | Gene.1073 | 529 | "PREDICTED: succinate dehydrogenase [ubiquinone] flavoprotein subunit, mitochondrial isoform X1 [Tribolium castaneum]" |
| map05010 Alzheimer disease | Gene.1073 | 546 | "PREDICTED: succinate dehydrogenase [ubiquinone] flavoprotein subunit, mitochondrial isoform X1 [Tribolium castaneum]" |
| map05010 Alzheimer disease | Gene.1073 | 175 | "PREDICTED: succinate dehydrogenase [ubiquinone] flavoprotein subunit, mitochondrial isoform X1 [Tribolium castaneum]" |
| map05010 Alzheimer disease | Gene.1073 | 632 | "PREDICTED: succinate dehydrogenase [ubiquinone] flavoprotein subunit, mitochondrial isoform X1 [Tribolium castaneum]" |
| map05010 Alzheimer disease | Gene.1073 | 543 | "PREDICTED: succinate dehydrogenase [ubiquinone] flavoprotein subunit, mitochondrial isoform X1 [Tribolium castaneum]" |
| map05010 Alzheimer disease | Gene.1073 | 331 | "PREDICTED: succinate dehydrogenase [ubiquinone] flavoprotein subunit, mitochondrial isoform X1 [Tribolium castaneum]" |
| map05010 Alzheimer disease | Gene.1912 | 120 | PREDICTED: NADH dehydrogenase [ubiquinone] 1 beta subcomplex subunit 9 [Aethina tumida] |
| map05010 Alzheimer disease | Gene.1912 | 51 | PREDICTED: NADH dehydrogenase [ubiquinone] 1 beta subcomplex subunit 9 [Aethina tumida] |
| map05010 Alzheimer disease | Gene.580 | 231 | "PREDICTED: cytochrome b-c1 complex subunit 2, mitochondrial [Tribolium castaneum]" |
| map05010 Alzheimer disease | Gene.580 | 248 | "PREDICTED: cytochrome b-c1 complex subunit 2, mitochondrial [Tribolium castaneum]" |
| map05010 Alzheimer disease | Gene.6169 | 53 | cytochrome b-c1 complex subunit 7-like [Anoplophora glabripennis] |
| map05010 Alzheimer disease | Gene.6169 | 77 | cytochrome b-c1 complex subunit 7-like [Anoplophora glabripennis] |
| map05010 Alzheimer disease | Gene.3481 | 53 | NADH dehydrogenase [ubiquinone] 1 alpha subcomplex subunit 8 [Asbolus verrucosus] |
| map05010 Alzheimer disease | Gene.2017 | 103 | "ATP synthase subunit O, mitochondrial [Asbolus verrucosus]" |
| map05010 Alzheimer disease | Gene.2017 | 182 | "ATP synthase subunit O, mitochondrial [Asbolus verrucosus]" |
| map05010 Alzheimer disease | Gene.2017 | 91 | "ATP synthase subunit O, mitochondrial [Asbolus verrucosus]" |
| map05010 Alzheimer disease | Gene.2017 | 121 | "ATP synthase subunit O, mitochondrial [Asbolus verrucosus]" |
| map05010 Alzheimer disease | Gene.2017 | 184 | "ATP synthase subunit O, mitochondrial [Asbolus verrucosus]" |
| map05010 Alzheimer disease | Gene.2522 | 46 | "cytochrome c oxidase subunit 5A, mitochondrial [Anoplophora glabripennis]" |
| map05010 Alzheimer disease | Gene.2522 | 101 | "cytochrome c oxidase subunit 5A, mitochondrial [Anoplophora glabripennis]" |
| map05010 Alzheimer disease | Gene.2522 | 51 | "cytochrome c oxidase subunit 5A, mitochondrial [Anoplophora glabripennis]" |
| map05010 Alzheimer disease | Gene.1845 | 92 | PREDICTED: glycogen synthase kinase-3 beta isoform X10 [Tribolium castaneum] |
| map05010 Alzheimer disease | Gene.1845 | 87 | PREDICTED: glycogen synthase kinase-3 beta isoform X10 [Tribolium castaneum] |
| map05010 Alzheimer disease | Gene.5767 | 89 | NADH dehydrogenase [ubiquinone] iron-sulfur protein 5 [Anoplophora glabripennis] |
| map05010 Alzheimer disease | Gene.8516 | 373 | PREDICTED: NADH-ubiquinone oxidoreductase 49 kDa subunit [Tribolium castaneum] |
| map05010 Alzheimer disease | Gene.8516 | 310 | PREDICTED: NADH-ubiquinone oxidoreductase 49 kDa subunit [Tribolium castaneum] |
| map05010 Alzheimer disease | Gene.8516 | 368 | PREDICTED: NADH-ubiquinone oxidoreductase 49 kDa subunit [Tribolium castaneum] |
| map05010 Alzheimer disease | Gene.8673 | 48 | "succinate dehydrogenase [ubiquinone] iron-sulfur subunit, mitochondrial, partial [Asbolus verrucosus]" |
| map05010 Alzheimer disease | Gene.3795 | 166 | "NADH-ubiquinone oxidoreductase 75 kDa subunit, mitochondrial [Asbolus verrucosus]" |
| map05010 Alzheimer disease | Gene.3795 | 172 | "NADH-ubiquinone oxidoreductase 75 kDa subunit, mitochondrial [Asbolus verrucosus]" |
| map05010 Alzheimer disease | Gene.3795 | 691 | "NADH-ubiquinone oxidoreductase 75 kDa subunit, mitochondrial [Asbolus verrucosus]" |
| map05010 Alzheimer disease | Gene.3795 | 626 | "NADH-ubiquinone oxidoreductase 75 kDa subunit, mitochondrial [Asbolus verrucosus]" |
| map05010 Alzheimer disease | Gene.3795 | 307 | "NADH-ubiquinone oxidoreductase 75 kDa subunit, mitochondrial [Asbolus verrucosus]" |
| map05010 Alzheimer disease | Gene.3795 | 541 | "NADH-ubiquinone oxidoreductase 75 kDa subunit, mitochondrial [Asbolus verrucosus]" |
| map05010 Alzheimer disease | Gene.3795 | 622 | "NADH-ubiquinone oxidoreductase 75 kDa subunit, mitochondrial [Asbolus verrucosus]" |
| map05010 Alzheimer disease | Gene.4352 | 128 | PREDICTED: calcium-transporting ATPase sarcoplasmic/endoplasmic reticulum type isoform X1 [Tribolium castaneum] |
| map05010 Alzheimer disease | Gene.4352 | 514 | PREDICTED: calcium-transporting ATPase sarcoplasmic/endoplasmic reticulum type isoform X1 [Tribolium castaneum] |
| map05010 Alzheimer disease | Gene.4352 | 30 | PREDICTED: calcium-transporting ATPase sarcoplasmic/endoplasmic reticulum type isoform X1 [Tribolium castaneum] |
| map05010 Alzheimer disease | Gene.3063 | 178 | PREDICTED: uncharacterized protein LOC108560181 [Nicrophorus vespilloides] |
| map05010 Alzheimer disease | Gene.7134 | 95 | "PREDICTED: ATP synthase subunit d, mitochondrial [Tribolium castaneum]" |
| map05010 Alzheimer disease | Gene.7134 | 105 | "PREDICTED: ATP synthase subunit d, mitochondrial [Tribolium castaneum]" |
| map05010 Alzheimer disease | Gene.1846 | 205 | "ATP synthase subunit beta, mitochondrial [Tribolium castaneum]" |
| map05010 Alzheimer disease | Gene.1846 | 128 | "ATP synthase subunit beta, mitochondrial [Tribolium castaneum]" |
| map05010 Alzheimer disease | Gene.1846 | 497 | "ATP synthase subunit beta, mitochondrial [Tribolium castaneum]" |
| map05010 Alzheimer disease | Gene.1846 | 263 | "ATP synthase subunit beta, mitochondrial [Tribolium castaneum]" |
| map05010 Alzheimer disease | Gene.1846 | 268 | "ATP synthase subunit beta, mitochondrial [Tribolium castaneum]" |
| map05010 Alzheimer disease | Gene.1846 | 202 | "ATP synthase subunit beta, mitochondrial [Tribolium castaneum]" |
| map05010 Alzheimer disease | Gene.1846 | 430 | "ATP synthase subunit beta, mitochondrial [Tribolium castaneum]" |
| map05010 Alzheimer disease | Gene.8771 | 54 | "PREDICTED: NADH dehydrogenase [ubiquinone] iron-sulfur protein 6, mitochondrial [Tribolium castaneum]" |
| map05143 African trypanosomiasis | Gene.7676 | 53 | PREDICTED: guanine nucleotide-binding protein G(q) subunit alpha isoform X1 [Tribolium castaneum] |
| map04146 Peroxisome | Gene.529 | 130 | dehydrogenase/reductase SDR family member 4-like [Anoplophora glabripennis] |
| map04146 Peroxisome | Gene.529 | 294 | dehydrogenase/reductase SDR family member 4-like [Anoplophora glabripennis] |
| map04146 Peroxisome | Gene.529 | 208 | dehydrogenase/reductase SDR family member 4-like [Anoplophora glabripennis] |
| map04146 Peroxisome | Gene.2197 | 664 | PREDICTED: peroxisomal multifunctional enzyme type 2 isoform X1 [Tribolium castaneum] |
| map04146 Peroxisome | Gene.2197 | 138 | PREDICTED: peroxisomal multifunctional enzyme type 2 isoform X1 [Tribolium castaneum] |
| map04146 Peroxisome | Gene.2197 | 275 | PREDICTED: peroxisomal multifunctional enzyme type 2 isoform X1 [Tribolium castaneum] |
| map04146 Peroxisome | Gene.2197 | 638 | PREDICTED: peroxisomal multifunctional enzyme type 2 isoform X1 [Tribolium castaneum] |
| map04146 Peroxisome | Gene.2197 | 647 | PREDICTED: peroxisomal multifunctional enzyme type 2 isoform X1 [Tribolium castaneum] |
| map04146 Peroxisome | Gene.6024 | 178 | peroxiredoxin 1-like [Leptinotarsa decemlineata] |
| map04146 Peroxisome | Gene.6024 | 186 | peroxiredoxin 1-like [Leptinotarsa decemlineata] |
| map04146 Peroxisome | Gene.6024 | 133 | peroxiredoxin 1-like [Leptinotarsa decemlineata] |
| map04146 Peroxisome | Gene.6024 | 105 | peroxiredoxin 1-like [Leptinotarsa decemlineata] |
| map04146 Peroxisome | Gene.6024 | 14 | peroxiredoxin 1-like [Leptinotarsa decemlineata] |
| map04146 Peroxisome | Gene.5965 | 63 | "PREDICTED: superoxide dismutase [Mn] 1, mitochondrial-like [Aethina tumida]" |
| map04146 Peroxisome | Gene.5965 | 68 | "PREDICTED: superoxide dismutase [Mn] 1, mitochondrial-like [Aethina tumida]" |
| map04146 Peroxisome | Gene.6564 | 120 | "peroxiredoxin-5, mitochondrial [Leptinotarsa decemlineata]" |
| map04146 Peroxisome | Gene.6564 | 57 | "peroxiredoxin-5, mitochondrial [Leptinotarsa decemlineata]" |
| map04146 Peroxisome | Gene.6564 | 133 | "peroxiredoxin-5, mitochondrial [Leptinotarsa decemlineata]" |
| map04146 Peroxisome | Gene.4302 | 243 | long-chain-fatty-acid--CoA ligase 4 isoform X1 [Agrilus planipennis] |
| map04146 Peroxisome | Gene.4302 | 416 | long-chain-fatty-acid--CoA ligase 4 isoform X1 [Agrilus planipennis] |
| map04146 Peroxisome | Gene.4302 | 411 | long-chain-fatty-acid--CoA ligase 4 isoform X1 [Agrilus planipennis] |
| map04146 Peroxisome | Gene.4302 | 263 | long-chain-fatty-acid--CoA ligase 4 isoform X1 [Agrilus planipennis] |
| map04146 Peroxisome | Gene.7551 | 170 | PREDICTED: non-specific lipid-transfer protein [Aethina tumida] |
| map04146 Peroxisome | Gene.7551 | 119 | PREDICTED: non-specific lipid-transfer protein [Aethina tumida] |
| map04146 Peroxisome | Gene.7551 | 269 | PREDICTED: non-specific lipid-transfer protein [Aethina tumida] |
| map04146 Peroxisome | Gene.1653 | 193 | PREDICTED: hydroxyacid oxidase 1 [Tribolium castaneum] |
| map04146 Peroxisome | Gene.4303 | 171 | PREDICTED: LOW QUALITY PROTEIN: long-chain-fatty-acid--CoA ligase 4 [Aethina tumida] |
| map04146 Peroxisome | Gene.6679 | 240 | Isocitrate dehydrogenase [NADP] [Operophtera brumata] |
| map04146 Peroxisome | Gene.6679 | 345 | Isocitrate dehydrogenase [NADP] [Operophtera brumata] |
| map04146 Peroxisome | Gene.6679 | 26 | Isocitrate dehydrogenase [NADP] [Operophtera brumata] |
| map04146 Peroxisome | Gene.4276 | 74 | "superoxide dismutase, partial [Harmonia axyridis]" |
| map04146 Peroxisome | Gene.4276 | 152 | "superoxide dismutase, partial [Harmonia axyridis]" |
| map04146 Peroxisome | Gene.4276 | 4 | "superoxide dismutase, partial [Harmonia axyridis]" |
| map04146 Peroxisome | Gene.4276 | 134 | "superoxide dismutase, partial [Harmonia axyridis]" |
| map04146 Peroxisome | Gene.4276 | 106 | "superoxide dismutase, partial [Harmonia axyridis]" |
| map04146 Peroxisome | Gene.6759 | 54 | hypothetical protein AMK59_2413 [Oryctes borbonicus] |
| map04146 Peroxisome | Gene.2933 | 197 | "isocitrate dehydrogenase [NADP], mitochondrial-like [Onthophagus taurus]" |
| map04146 Peroxisome | Gene.2933 | 272 | "isocitrate dehydrogenase [NADP], mitochondrial-like [Onthophagus taurus]" |
| map04146 Peroxisome | Gene.2933 | 39 | "isocitrate dehydrogenase [NADP], mitochondrial-like [Onthophagus taurus]" |
| map04146 Peroxisome | Gene.2933 | 194 | "isocitrate dehydrogenase [NADP], mitochondrial-like [Onthophagus taurus]" |
| map04146 Peroxisome | Gene.2933 | 273 | "isocitrate dehydrogenase [NADP], mitochondrial-like [Onthophagus taurus]" |
| map04146 Peroxisome | Gene.2933 | 183 | "isocitrate dehydrogenase [NADP], mitochondrial-like [Onthophagus taurus]" |
| map04146 Peroxisome | Gene.2933 | 266 | "isocitrate dehydrogenase [NADP], mitochondrial-like [Onthophagus taurus]" |
| map04146 Peroxisome | Gene.2933 | 401 | "isocitrate dehydrogenase [NADP], mitochondrial-like [Onthophagus taurus]" |
| map04146 Peroxisome | Gene.2933 | 172 | "isocitrate dehydrogenase [NADP], mitochondrial-like [Onthophagus taurus]" |
| map04146 Peroxisome | Gene.2933 | 254 | "isocitrate dehydrogenase [NADP], mitochondrial-like [Onthophagus taurus]" |
| map04146 Peroxisome | Gene.2933 | 98 | "isocitrate dehydrogenase [NADP], mitochondrial-like [Onthophagus taurus]" |
| map04146 Peroxisome | Gene.2933 | 294 | "isocitrate dehydrogenase [NADP], mitochondrial-like [Onthophagus taurus]" |
| map04146 Peroxisome | Gene.4404 | 26 | peroxisomal acyl-coenzyme A oxidase 3 isoform X1 [Anoplophora glabripennis] |
| map04146 Peroxisome | Gene.5542 | 28 | catalase [Onthophagus taurus] |
| map04146 Peroxisome | Gene.5542 | 179 | catalase [Onthophagus taurus] |
| map04146 Peroxisome | Gene.5542 | 325 | catalase [Onthophagus taurus] |
| map04146 Peroxisome | Gene.5542 | 247 | catalase [Onthophagus taurus] |
| map04146 Peroxisome | Gene.5542 | 438 | catalase [Onthophagus taurus] |
| map04146 Peroxisome | Gene.5542 | 48 | catalase [Onthophagus taurus] |
| map05323 Rheumatoid arthritis | Gene.2318 | 109 | PREDICTED: LOW QUALITY PROTEIN: V-type proton ATPase 116 kDa subunit a-like [Aethina tumida] |
| map05323 Rheumatoid arthritis | Gene.2318 | 50 | PREDICTED: LOW QUALITY PROTEIN: V-type proton ATPase 116 kDa subunit a-like [Aethina tumida] |
| map05323 Rheumatoid arthritis | Gene.2318 | 522 | PREDICTED: LOW QUALITY PROTEIN: V-type proton ATPase 116 kDa subunit a-like [Aethina tumida] |
| map05323 Rheumatoid arthritis | Gene.2318 | 76 | PREDICTED: LOW QUALITY PROTEIN: V-type proton ATPase 116 kDa subunit a-like [Aethina tumida] |
| map05323 Rheumatoid arthritis | Gene.2318 | 266 | PREDICTED: LOW QUALITY PROTEIN: V-type proton ATPase 116 kDa subunit a-like [Aethina tumida] |
| map05323 Rheumatoid arthritis | Gene.6879 | 42 | V-type proton ATPase subunit E [Leptinotarsa decemlineata] |
| map05323 Rheumatoid arthritis | Gene.6879 | 59 | V-type proton ATPase subunit E [Leptinotarsa decemlineata] |
| map05323 Rheumatoid arthritis | Gene.6879 | 156 | V-type proton ATPase subunit E [Leptinotarsa decemlineata] |
| map05323 Rheumatoid arthritis | Gene.6879 | 10 | V-type proton ATPase subunit E [Leptinotarsa decemlineata] |
| map05323 Rheumatoid arthritis | Gene.6879 | 68 | V-type proton ATPase subunit E [Leptinotarsa decemlineata] |
| map05323 Rheumatoid arthritis | Gene.4190 | 409 | V-type proton ATPase subunit H [Asbolus verrucosus] |
| map05323 Rheumatoid arthritis | Gene.246 | 39 | PREDICTED: V-type proton ATPase subunit d [Tribolium castaneum] |
| map05323 Rheumatoid arthritis | Gene.5868 | 50 | PREDICTED: V-type proton ATPase subunit D [Polistes canadensis] |
| map05323 Rheumatoid arthritis | Gene.5815 | 276 | PREDICTED: uncharacterized protein LOC663029 [Tribolium castaneum] |
| map05323 Rheumatoid arthritis | Gene.5815 | 279 | PREDICTED: uncharacterized protein LOC663029 [Tribolium castaneum] |
| map05323 Rheumatoid arthritis | Gene.5815 | 410 | PREDICTED: uncharacterized protein LOC663029 [Tribolium castaneum] |
| map05323 Rheumatoid arthritis | Gene.77 | 200 | cathepsin L [Anoplophora glabripennis] |
| map05323 Rheumatoid arthritis | Gene.77 | 57 | cathepsin L [Anoplophora glabripennis] |
| map05323 Rheumatoid arthritis | Gene.77 | 134 | cathepsin L [Anoplophora glabripennis] |
| map05323 Rheumatoid arthritis | Gene.77 | 36 | cathepsin L [Anoplophora glabripennis] |
| map05323 Rheumatoid arthritis | Gene.77 | 216 | cathepsin L [Anoplophora glabripennis] |
| map05323 Rheumatoid arthritis | Gene.77 | 211 | cathepsin L [Anoplophora glabripennis] |
| map05323 Rheumatoid arthritis | Gene.5862 | 8 | "V-type proton ATPase subunit F, partial [Asbolus verrucosus]" |
| map05323 Rheumatoid arthritis | Gene.5862 | 106 | "V-type proton ATPase subunit F, partial [Asbolus verrucosus]" |
| map05323 Rheumatoid arthritis | Gene.7366 | 21 | PREDICTED: V-type proton ATPase subunit G [Nicrophorus vespilloides] |
| map05323 Rheumatoid arthritis | Gene.7366 | 61 | PREDICTED: V-type proton ATPase subunit G [Nicrophorus vespilloides] |
| map05323 Rheumatoid arthritis | Gene.7366 | 37 | PREDICTED: V-type proton ATPase subunit G [Nicrophorus vespilloides] |
| map05323 Rheumatoid arthritis | Gene.4379 | 57 | cathepsin L precursor [Tribolium castaneum] |
| map05323 Rheumatoid arthritis | Gene.2884 | 100 | PREDICTED: cathepsin L1 [Tribolium castaneum] |
| map05323 Rheumatoid arthritis | Gene.1524 | 445 | V-type proton ATPase subunit B [Galleria mellonella] |
| map05323 Rheumatoid arthritis | Gene.6297 | 155 | PREDICTED: V-type proton ATPase subunit C isoform X3 [Tribolium castaneum] |
| map05323 Rheumatoid arthritis | Gene.6297 | 139 | PREDICTED: V-type proton ATPase subunit C isoform X3 [Tribolium castaneum] |
| map05323 Rheumatoid arthritis | Gene.6297 | 267 | PREDICTED: V-type proton ATPase subunit C isoform X3 [Tribolium castaneum] |
| map05323 Rheumatoid arthritis | Gene.6297 | 262 | PREDICTED: V-type proton ATPase subunit C isoform X3 [Tribolium castaneum] |
| map05323 Rheumatoid arthritis | Gene.664 | 50 | PREDICTED: V-type proton ATPase 116 kDa subunit a isoform 1 isoform X3 [Tribolium castaneum] |
| map05323 Rheumatoid arthritis | Gene.3482 | 588 | V-type proton ATPase catalytic subunit A [Leptinotarsa decemlineata] |
| map05323 Rheumatoid arthritis | Gene.3482 | 533 | V-type proton ATPase catalytic subunit A [Leptinotarsa decemlineata] |
| map05323 Rheumatoid arthritis | Gene.3482 | 130 | V-type proton ATPase catalytic subunit A [Leptinotarsa decemlineata] |
| map05323 Rheumatoid arthritis | Gene.3482 | 593 | V-type proton ATPase catalytic subunit A [Leptinotarsa decemlineata] |
| map05323 Rheumatoid arthritis | Gene.3482 | 584 | V-type proton ATPase catalytic subunit A [Leptinotarsa decemlineata] |
| map05323 Rheumatoid arthritis | Gene.3482 | 513 | V-type proton ATPase catalytic subunit A [Leptinotarsa decemlineata] |
| map03030 DNA replication | Gene.6292 | 32 | "PREDICTED: single-stranded DNA-binding protein, mitochondrial [Tribolium castaneum]" |
| map03030 DNA replication | Gene.6292 | 100 | "PREDICTED: single-stranded DNA-binding protein, mitochondrial [Tribolium castaneum]" |
| map05110 Vibrio cholerae infection | Gene.8388 | 104 | ADP-ribosylation factor 1 isoform X1 [Penaeus vannamei] |
| map05110 Vibrio cholerae infection | Gene.8388 | 36 | ADP-ribosylation factor 1 isoform X1 [Penaeus vannamei] |
| map05110 Vibrio cholerae infection | Gene.2318 | 109 | PREDICTED: LOW QUALITY PROTEIN: V-type proton ATPase 116 kDa subunit a-like [Aethina tumida] |
| map05110 Vibrio cholerae infection | Gene.2318 | 50 | PREDICTED: LOW QUALITY PROTEIN: V-type proton ATPase 116 kDa subunit a-like [Aethina tumida] |
| map05110 Vibrio cholerae infection | Gene.2318 | 522 | PREDICTED: LOW QUALITY PROTEIN: V-type proton ATPase 116 kDa subunit a-like [Aethina tumida] |
| map05110 Vibrio cholerae infection | Gene.2318 | 76 | PREDICTED: LOW QUALITY PROTEIN: V-type proton ATPase 116 kDa subunit a-like [Aethina tumida] |
| map05110 Vibrio cholerae infection | Gene.2318 | 266 | PREDICTED: LOW QUALITY PROTEIN: V-type proton ATPase 116 kDa subunit a-like [Aethina tumida] |
| map05110 Vibrio cholerae infection | Gene.6879 | 42 | V-type proton ATPase subunit E [Leptinotarsa decemlineata] |
| map05110 Vibrio cholerae infection | Gene.6879 | 59 | V-type proton ATPase subunit E [Leptinotarsa decemlineata] |
| map05110 Vibrio cholerae infection | Gene.6879 | 156 | V-type proton ATPase subunit E [Leptinotarsa decemlineata] |
| map05110 Vibrio cholerae infection | Gene.6879 | 10 | V-type proton ATPase subunit E [Leptinotarsa decemlineata] |
| map05110 Vibrio cholerae infection | Gene.6879 | 68 | V-type proton ATPase subunit E [Leptinotarsa decemlineata] |
| map05110 Vibrio cholerae infection | Gene.4190 | 409 | V-type proton ATPase subunit H [Asbolus verrucosus] |
| map05110 Vibrio cholerae infection | Gene.246 | 39 | PREDICTED: V-type proton ATPase subunit d [Tribolium castaneum] |
| map05110 Vibrio cholerae infection | Gene.5868 | 50 | PREDICTED: V-type proton ATPase subunit D [Polistes canadensis] |
| map05110 Vibrio cholerae infection | Gene.5815 | 276 | PREDICTED: uncharacterized protein LOC663029 [Tribolium castaneum] |
| map05110 Vibrio cholerae infection | Gene.5815 | 279 | PREDICTED: uncharacterized protein LOC663029 [Tribolium castaneum] |
| map05110 Vibrio cholerae infection | Gene.5815 | 410 | PREDICTED: uncharacterized protein LOC663029 [Tribolium castaneum] |
| map05110 Vibrio cholerae infection | Gene.5862 | 8 | "V-type proton ATPase subunit F, partial [Asbolus verrucosus]" |
| map05110 Vibrio cholerae infection | Gene.5862 | 106 | "V-type proton ATPase subunit F, partial [Asbolus verrucosus]" |
| map05110 Vibrio cholerae infection | Gene.7366 | 21 | PREDICTED: V-type proton ATPase subunit G [Nicrophorus vespilloides] |
| map05110 Vibrio cholerae infection | Gene.7366 | 61 | PREDICTED: V-type proton ATPase subunit G [Nicrophorus vespilloides] |
| map05110 Vibrio cholerae infection | Gene.7366 | 37 | PREDICTED: V-type proton ATPase subunit G [Nicrophorus vespilloides] |
| map05110 Vibrio cholerae infection | Gene.5709 | 26 | cAMP-dependent protein kinase catalytic subunit [Agrilus planipennis] |
| map05110 Vibrio cholerae infection | Gene.5709 | 257 | cAMP-dependent protein kinase catalytic subunit [Agrilus planipennis] |
| map05110 Vibrio cholerae infection | Gene.1524 | 445 | V-type proton ATPase subunit B [Galleria mellonella] |
| map05110 Vibrio cholerae infection | Gene.6297 | 155 | PREDICTED: V-type proton ATPase subunit C isoform X3 [Tribolium castaneum] |
| map05110 Vibrio cholerae infection | Gene.6297 | 139 | PREDICTED: V-type proton ATPase subunit C isoform X3 [Tribolium castaneum] |
| map05110 Vibrio cholerae infection | Gene.6297 | 267 | PREDICTED: V-type proton ATPase subunit C isoform X3 [Tribolium castaneum] |
| map05110 Vibrio cholerae infection | Gene.6297 | 262 | PREDICTED: V-type proton ATPase subunit C isoform X3 [Tribolium castaneum] |
| map05110 Vibrio cholerae infection | Gene.461 | 392 | PREDICTED: protein transport protein Sec61 subunit alpha isoform 2 [Tribolium castaneum] |
| map05110 Vibrio cholerae infection | Gene.461 | 107 | PREDICTED: protein transport protein Sec61 subunit alpha isoform 2 [Tribolium castaneum] |
| map05110 Vibrio cholerae infection | Gene.664 | 50 | PREDICTED: V-type proton ATPase 116 kDa subunit a isoform 1 isoform X3 [Tribolium castaneum] |
| map05110 Vibrio cholerae infection | Gene.3482 | 588 | V-type proton ATPase catalytic subunit A [Leptinotarsa decemlineata] |
| map05110 Vibrio cholerae infection | Gene.3482 | 533 | V-type proton ATPase catalytic subunit A [Leptinotarsa decemlineata] |
| map05110 Vibrio cholerae infection | Gene.3482 | 130 | V-type proton ATPase catalytic subunit A [Leptinotarsa decemlineata] |
| map05110 Vibrio cholerae infection | Gene.3482 | 593 | V-type proton ATPase catalytic subunit A [Leptinotarsa decemlineata] |
| map05110 Vibrio cholerae infection | Gene.3482 | 584 | V-type proton ATPase catalytic subunit A [Leptinotarsa decemlineata] |
| map05110 Vibrio cholerae infection | Gene.3482 | 513 | V-type proton ATPase catalytic subunit A [Leptinotarsa decemlineata] |
| map05110 Vibrio cholerae infection | Gene.172 | 216 | "beta-actin, partial [Cotesia chilonis]" |
| map05110 Vibrio cholerae infection | Gene.172 | 51 | "beta-actin, partial [Cotesia chilonis]" |
| map05110 Vibrio cholerae infection | Gene.172 | 114 | "beta-actin, partial [Cotesia chilonis]" |
| map05110 Vibrio cholerae infection | Gene.172 | 62 | "beta-actin, partial [Cotesia chilonis]" |
| map05110 Vibrio cholerae infection | Gene.2646 | 99 | beta actin [Polyrhachis vicina] |
| map05110 Vibrio cholerae infection | Gene.2646 | 88 | beta actin [Polyrhachis vicina] |
| map05110 Vibrio cholerae infection | Gene.2646 | 64 | beta actin [Polyrhachis vicina] |
| map05110 Vibrio cholerae infection | Gene.2646 | 101 | beta actin [Polyrhachis vicina] |
| map05170 Human immunodeficiency virus 1 infection | Gene.8756 | 84 | uncharacterized protein Dvir_GJ16722 [Drosophila virilis] |
| map05170 Human immunodeficiency virus 1 infection | Gene.8756 | 10 | uncharacterized protein Dvir_GJ16722 [Drosophila virilis] |
| map05170 Human immunodeficiency virus 1 infection | Gene.7276 | 170 | protein disulfide-isomerase A3 [Asbolus verrucosus] |
| map05170 Human immunodeficiency virus 1 infection | Gene.7276 | 134 | protein disulfide-isomerase A3 [Asbolus verrucosus] |
| map05170 Human immunodeficiency virus 1 infection | Gene.7276 | 165 | protein disulfide-isomerase A3 [Asbolus verrucosus] |
| map05170 Human immunodeficiency virus 1 infection | Gene.7276 | 210 | protein disulfide-isomerase A3 [Asbolus verrucosus] |
| map05170 Human immunodeficiency virus 1 infection | Gene.7276 | 78 | protein disulfide-isomerase A3 [Asbolus verrucosus] |
| map05170 Human immunodeficiency virus 1 infection | Gene.7276 | 268 | protein disulfide-isomerase A3 [Asbolus verrucosus] |
| map05170 Human immunodeficiency virus 1 infection | Gene.7276 | 449 | protein disulfide-isomerase A3 [Asbolus verrucosus] |
| map05170 Human immunodeficiency virus 1 infection | Gene.7276 | 62 | protein disulfide-isomerase A3 [Asbolus verrucosus] |
| map05170 Human immunodeficiency virus 1 infection | Gene.7276 | 247 | protein disulfide-isomerase A3 [Asbolus verrucosus] |
| map05170 Human immunodeficiency virus 1 infection | Gene.8135 | 359 | PREDICTED: calreticulin [Tribolium castaneum] |
| map05170 Human immunodeficiency virus 1 infection | Gene.8135 | 203 | PREDICTED: calreticulin [Tribolium castaneum] |
| map05170 Human immunodeficiency virus 1 infection | Gene.8135 | 206 | PREDICTED: calreticulin [Tribolium castaneum] |
| map05170 Human immunodeficiency virus 1 infection | Gene.8135 | 61 | PREDICTED: calreticulin [Tribolium castaneum] |
| map05170 Human immunodeficiency virus 1 infection | Gene.8135 | 108 | PREDICTED: calreticulin [Tribolium castaneum] |
| map05170 Human immunodeficiency virus 1 infection | Gene.8135 | 52 | PREDICTED: calreticulin [Tribolium castaneum] |
| map05170 Human immunodeficiency virus 1 infection | Gene.8135 | 354 | PREDICTED: calreticulin [Tribolium castaneum] |
| map05170 Human immunodeficiency virus 1 infection | Gene.8135 | 37 | PREDICTED: calreticulin [Tribolium castaneum] |
| map05170 Human immunodeficiency virus 1 infection | Gene.8135 | 45 | PREDICTED: calreticulin [Tribolium castaneum] |
| map05170 Human immunodeficiency virus 1 infection | Gene.8135 | 275 | PREDICTED: calreticulin [Tribolium castaneum] |
| map05170 Human immunodeficiency virus 1 infection | Gene.8135 | 31 | PREDICTED: calreticulin [Tribolium castaneum] |
| map05170 Human immunodeficiency virus 1 infection | Gene.7676 | 53 | PREDICTED: guanine nucleotide-binding protein G(q) subunit alpha isoform X1 [Tribolium castaneum] |
| map05170 Human immunodeficiency virus 1 infection | Gene.7526 | 96 | ras-related protein Rac1 [Anoplophora glabripennis] |
| map05170 Human immunodeficiency virus 1 infection | Gene.164 | 78 | PREDICTED: guanine nucleotide-binding protein subunit beta-1 [Tribolium castaneum] |
| map05170 Human immunodeficiency virus 1 infection | Gene.5803 | 20 | calcium-binding protein E63-1 [Anoplophora glabripennis] |
| map05170 Human immunodeficiency virus 1 infection | Gene.8288 | 19 | cofilin/actin-depolymerizing factor homolog [Anoplophora glabripennis] |
| map05170 Human immunodeficiency virus 1 infection | Gene.8288 | 41 | cofilin/actin-depolymerizing factor homolog [Anoplophora glabripennis] |
| map05170 Human immunodeficiency virus 1 infection | Gene.298 | 116 | "Csa-calmodulin 3, partial [Cupiennius salei]" |
| map05170 Human immunodeficiency virus 1 infection | Gene.298 | 95 | "Csa-calmodulin 3, partial [Cupiennius salei]" |
| map05170 Human immunodeficiency virus 1 infection | Gene.298 | 31 | "Csa-calmodulin 3, partial [Cupiennius salei]" |
| map05170 Human immunodeficiency virus 1 infection | Gene.298 | 78 | "Csa-calmodulin 3, partial [Cupiennius salei]" |
| map00360 Phenylalanine metabolism | Gene.2656 | 56 | "PREDICTED: aspartate aminotransferase, cytoplasmic [Tribolium castaneum]" |
| map00360 Phenylalanine metabolism | Gene.2656 | 141 | "PREDICTED: aspartate aminotransferase, cytoplasmic [Tribolium castaneum]" |
| map00360 Phenylalanine metabolism | Gene.1489 | 401 | "aspartate aminotransferase, mitochondrial [Asbolus verrucosus]" |
| map00360 Phenylalanine metabolism | Gene.1489 | 87 | "aspartate aminotransferase, mitochondrial [Asbolus verrucosus]" |
| map00360 Phenylalanine metabolism | Gene.1489 | 189 | "aspartate aminotransferase, mitochondrial [Asbolus verrucosus]" |
| map00360 Phenylalanine metabolism | Gene.1489 | 334 | "aspartate aminotransferase, mitochondrial [Asbolus verrucosus]" |
| map00360 Phenylalanine metabolism | Gene.1489 | 174 | "aspartate aminotransferase, mitochondrial [Asbolus verrucosus]" |
| map00360 Phenylalanine metabolism | Gene.1489 | 393 | "aspartate aminotransferase, mitochondrial [Asbolus verrucosus]" |
| map00360 Phenylalanine metabolism | Gene.1489 | 332 | "aspartate aminotransferase, mitochondrial [Asbolus verrucosus]" |
| map00360 Phenylalanine metabolism | Gene.1489 | 91 | "aspartate aminotransferase, mitochondrial [Asbolus verrucosus]" |
| map00360 Phenylalanine metabolism | Gene.1489 | 295 | "aspartate aminotransferase, mitochondrial [Asbolus verrucosus]" |
| map00360 Phenylalanine metabolism | Gene.986 | 265 | "PREDICTED: aldehyde dehydrogenase, dimeric NADP-preferring [Tribolium castaneum]" |
| map00360 Phenylalanine metabolism | Gene.986 | 452 | "PREDICTED: aldehyde dehydrogenase, dimeric NADP-preferring [Tribolium castaneum]" |
| map00360 Phenylalanine metabolism | Gene.986 | 19 | "PREDICTED: aldehyde dehydrogenase, dimeric NADP-preferring [Tribolium castaneum]" |
| map00360 Phenylalanine metabolism | Gene.6572 | 86 | macrophage migration inhibitory factor homolog [Agrilus planipennis] |
| map00360 Phenylalanine metabolism | Gene.6572 | 78 | macrophage migration inhibitory factor homolog [Agrilus planipennis] |
| map04625 C-type lectin receptor signaling pathway | Gene.298 | 116 | "Csa-calmodulin 3, partial [Cupiennius salei]" |
| map04625 C-type lectin receptor signaling pathway | Gene.298 | 95 | "Csa-calmodulin 3, partial [Cupiennius salei]" |
| map04625 C-type lectin receptor signaling pathway | Gene.298 | 31 | "Csa-calmodulin 3, partial [Cupiennius salei]" |
| map04625 C-type lectin receptor signaling pathway | Gene.298 | 78 | "Csa-calmodulin 3, partial [Cupiennius salei]" |
| map04625 C-type lectin receptor signaling pathway | Gene.5803 | 20 | calcium-binding protein E63-1 [Anoplophora glabripennis] |
| map05225 Hepatocellular carcinoma | Gene.8382 | 77 | GST [Lygus lineolaris] |
| map05225 Hepatocellular carcinoma | Gene.8382 | 80 | GST [Lygus lineolaris] |
| map05225 Hepatocellular carcinoma | Gene.8382 | 31 | GST [Lygus lineolaris] |
| map05225 Hepatocellular carcinoma | Gene.9040 | 57 | PREDICTED: microsomal glutathione S-transferase 1 [Tribolium castaneum] |
| map05225 Hepatocellular carcinoma | Gene.9040 | 55 | PREDICTED: microsomal glutathione S-transferase 1 [Tribolium castaneum] |
| map05225 Hepatocellular carcinoma | Gene.1845 | 92 | PREDICTED: glycogen synthase kinase-3 beta isoform X10 [Tribolium castaneum] |
| map05225 Hepatocellular carcinoma | Gene.1845 | 87 | PREDICTED: glycogen synthase kinase-3 beta isoform X10 [Tribolium castaneum] |
| map05225 Hepatocellular carcinoma | Gene.425 | 128 | glutathione S-transferase 1-1 [Anoplophora glabripennis] |
| map05225 Hepatocellular carcinoma | Gene.425 | 215 | glutathione S-transferase 1-1 [Anoplophora glabripennis] |
| map05225 Hepatocellular carcinoma | Gene.425 | 131 | glutathione S-transferase 1-1 [Anoplophora glabripennis] |
| map05225 Hepatocellular carcinoma | Gene.425 | 201 | glutathione S-transferase 1-1 [Anoplophora glabripennis] |
| map05225 Hepatocellular carcinoma | Gene.425 | 207 | glutathione S-transferase 1-1 [Anoplophora glabripennis] |
| map05225 Hepatocellular carcinoma | Gene.425 | 37 | glutathione S-transferase 1-1 [Anoplophora glabripennis] |
| map05225 Hepatocellular carcinoma | Gene.425 | 186 | glutathione S-transferase 1-1 [Anoplophora glabripennis] |
| map05225 Hepatocellular carcinoma | Gene.172 | 216 | "beta-actin, partial [Cotesia chilonis]" |
| map05225 Hepatocellular carcinoma | Gene.172 | 51 | "beta-actin, partial [Cotesia chilonis]" |
| map05225 Hepatocellular carcinoma | Gene.172 | 114 | "beta-actin, partial [Cotesia chilonis]" |
| map05225 Hepatocellular carcinoma | Gene.172 | 62 | "beta-actin, partial [Cotesia chilonis]" |
| map05225 Hepatocellular carcinoma | Gene.2646 | 99 | beta actin [Polyrhachis vicina] |
| map05225 Hepatocellular carcinoma | Gene.2646 | 88 | beta actin [Polyrhachis vicina] |
| map05225 Hepatocellular carcinoma | Gene.2646 | 64 | beta actin [Polyrhachis vicina] |
| map05225 Hepatocellular carcinoma | Gene.2646 | 101 | beta actin [Polyrhachis vicina] |
| map00982 Drug metabolism - cytochrome P450 | Gene.3731 | 221 | PREDICTED: UDP-glucuronosyltransferase 2B2 [Tribolium castaneum] |
| map00982 Drug metabolism - cytochrome P450 | Gene.3734 | 395 | PREDICTED: UDP-glucuronosyltransferase 2B7 isoform X1 [Tribolium castaneum] |
| map00982 Drug metabolism - cytochrome P450 | Gene.3734 | 305 | PREDICTED: UDP-glucuronosyltransferase 2B7 isoform X1 [Tribolium castaneum] |
| map00982 Drug metabolism - cytochrome P450 | Gene.3734 | 400 | PREDICTED: UDP-glucuronosyltransferase 2B7 isoform X1 [Tribolium castaneum] |
| map00982 Drug metabolism - cytochrome P450 | Gene.3734 | 378 | PREDICTED: UDP-glucuronosyltransferase 2B7 isoform X1 [Tribolium castaneum] |
| map00982 Drug metabolism - cytochrome P450 | Gene.3734 | 406 | PREDICTED: UDP-glucuronosyltransferase 2B7 isoform X1 [Tribolium castaneum] |
| map00982 Drug metabolism - cytochrome P450 | Gene.2176 | 48 | UDP-glucuronosyltransferase 1-9-like isoform X2 [Leptinotarsa decemlineata] |
| map00982 Drug metabolism - cytochrome P450 | Gene.9040 | 57 | PREDICTED: microsomal glutathione S-transferase 1 [Tribolium castaneum] |
| map00982 Drug metabolism - cytochrome P450 | Gene.9040 | 55 | PREDICTED: microsomal glutathione S-transferase 1 [Tribolium castaneum] |
| map00982 Drug metabolism - cytochrome P450 | Gene.986 | 265 | "PREDICTED: aldehyde dehydrogenase, dimeric NADP-preferring [Tribolium castaneum]" |
| map00982 Drug metabolism - cytochrome P450 | Gene.986 | 452 | "PREDICTED: aldehyde dehydrogenase, dimeric NADP-preferring [Tribolium castaneum]" |
| map00982 Drug metabolism - cytochrome P450 | Gene.986 | 19 | "PREDICTED: aldehyde dehydrogenase, dimeric NADP-preferring [Tribolium castaneum]" |
| map00982 Drug metabolism - cytochrome P450 | Gene.7878 | 6 | alcohol dehydrogenase class-3 [Leptinotarsa decemlineata] |
| map00982 Drug metabolism - cytochrome P450 | Gene.7878 | 340 | alcohol dehydrogenase class-3 [Leptinotarsa decemlineata] |
| map00982 Drug metabolism - cytochrome P450 | Gene.8382 | 77 | GST [Lygus lineolaris] |
| map00982 Drug metabolism - cytochrome P450 | Gene.8382 | 80 | GST [Lygus lineolaris] |
| map00982 Drug metabolism - cytochrome P450 | Gene.8382 | 31 | GST [Lygus lineolaris] |
| map00982 Drug metabolism - cytochrome P450 | Gene.8787 | 296 | UDPGT domain containing protein [Asbolus verrucosus] |
| map00982 Drug metabolism - cytochrome P450 | Gene.8787 | 208 | UDPGT domain containing protein [Asbolus verrucosus] |
| map00982 Drug metabolism - cytochrome P450 | Gene.8787 | 442 | UDPGT domain containing protein [Asbolus verrucosus] |
| map00982 Drug metabolism - cytochrome P450 | Gene.2052 | 422 | "UDP-glucuronosyltransferase 2C1-like, partial [Asbolus verrucosus]" |
| map00982 Drug metabolism - cytochrome P450 | Gene.4396 | 69 | PREDICTED: 2-hydroxyacylsphingosine 1-beta-galactosyltransferase-like [Tribolium castaneum] |
| map00982 Drug metabolism - cytochrome P450 | Gene.4396 | 317 | PREDICTED: 2-hydroxyacylsphingosine 1-beta-galactosyltransferase-like [Tribolium castaneum] |
| map00982 Drug metabolism - cytochrome P450 | Gene.4396 | 49 | PREDICTED: 2-hydroxyacylsphingosine 1-beta-galactosyltransferase-like [Tribolium castaneum] |
| map00982 Drug metabolism - cytochrome P450 | Gene.4396 | 259 | PREDICTED: 2-hydroxyacylsphingosine 1-beta-galactosyltransferase-like [Tribolium castaneum] |
| map00982 Drug metabolism - cytochrome P450 | Gene.3401 | 74 | glutathione S-transferase-like [Anoplophora glabripennis] |
| map00982 Drug metabolism - cytochrome P450 | Gene.3401 | 105 | glutathione S-transferase-like [Anoplophora glabripennis] |
| map00982 Drug metabolism - cytochrome P450 | Gene.3401 | 113 | glutathione S-transferase-like [Anoplophora glabripennis] |
| map00982 Drug metabolism - cytochrome P450 | Gene.425 | 128 | glutathione S-transferase 1-1 [Anoplophora glabripennis] |
| map00982 Drug metabolism - cytochrome P450 | Gene.425 | 215 | glutathione S-transferase 1-1 [Anoplophora glabripennis] |
| map00982 Drug metabolism - cytochrome P450 | Gene.425 | 131 | glutathione S-transferase 1-1 [Anoplophora glabripennis] |
| map00982 Drug metabolism - cytochrome P450 | Gene.425 | 201 | glutathione S-transferase 1-1 [Anoplophora glabripennis] |
| map00982 Drug metabolism - cytochrome P450 | Gene.425 | 207 | glutathione S-transferase 1-1 [Anoplophora glabripennis] |
| map00982 Drug metabolism - cytochrome P450 | Gene.425 | 37 | glutathione S-transferase 1-1 [Anoplophora glabripennis] |
| map00982 Drug metabolism - cytochrome P450 | Gene.425 | 186 | glutathione S-transferase 1-1 [Anoplophora glabripennis] |
| map00982 Drug metabolism - cytochrome P450 | Gene.2140 | 401 | PREDICTED: UDP-glucuronosyltransferase 2B10-like [Tribolium castaneum] |
| map00982 Drug metabolism - cytochrome P450 | Gene.2140 | 80 | PREDICTED: UDP-glucuronosyltransferase 2B10-like [Tribolium castaneum] |
| map00982 Drug metabolism - cytochrome P450 | Gene.2140 | 425 | PREDICTED: UDP-glucuronosyltransferase 2B10-like [Tribolium castaneum] |
| map00982 Drug metabolism - cytochrome P450 | Gene.2140 | 410 | PREDICTED: UDP-glucuronosyltransferase 2B10-like [Tribolium castaneum] |
| map00982 Drug metabolism - cytochrome P450 | Gene.1050 | 121 | PREDICTED: 2-hydroxyacylsphingosine 1-beta-galactosyltransferase-like [Tribolium castaneum] |
| map00982 Drug metabolism - cytochrome P450 | Gene.2175 | 192 | PREDICTED: 2-hydroxyacylsphingosine 1-beta-galactosyltransferase-like [Tribolium castaneum] |
| map00982 Drug metabolism - cytochrome P450 | Gene.2178 | 47 | UDPGT and/or Glyco tran 28 C domain containing protein [Asbolus verrucosus] |
| map04916 Melanogenesis | Gene.5709 | 26 | cAMP-dependent protein kinase catalytic subunit [Agrilus planipennis] |
| map04916 Melanogenesis | Gene.5709 | 257 | cAMP-dependent protein kinase catalytic subunit [Agrilus planipennis] |
| map04916 Melanogenesis | Gene.298 | 116 | "Csa-calmodulin 3, partial [Cupiennius salei]" |
| map04916 Melanogenesis | Gene.298 | 95 | "Csa-calmodulin 3, partial [Cupiennius salei]" |
| map04916 Melanogenesis | Gene.298 | 31 | "Csa-calmodulin 3, partial [Cupiennius salei]" |
| map04916 Melanogenesis | Gene.298 | 78 | "Csa-calmodulin 3, partial [Cupiennius salei]" |
| map04916 Melanogenesis | Gene.1845 | 92 | PREDICTED: glycogen synthase kinase-3 beta isoform X10 [Tribolium castaneum] |
| map04916 Melanogenesis | Gene.1845 | 87 | PREDICTED: glycogen synthase kinase-3 beta isoform X10 [Tribolium castaneum] |
| map04916 Melanogenesis | Gene.7676 | 53 | PREDICTED: guanine nucleotide-binding protein G(q) subunit alpha isoform X1 [Tribolium castaneum] |
| map04916 Melanogenesis | Gene.5803 | 20 | calcium-binding protein E63-1 [Anoplophora glabripennis] |
| map00983 Drug metabolism - other enzymes | Gene.8382 | 77 | GST [Lygus lineolaris] |
| map00983 Drug metabolism - other enzymes | Gene.8382 | 80 | GST [Lygus lineolaris] |
| map00983 Drug metabolism - other enzymes | Gene.8382 | 31 | GST [Lygus lineolaris] |
| map00983 Drug metabolism - other enzymes | Gene.8787 | 296 | UDPGT domain containing protein [Asbolus verrucosus] |
| map00983 Drug metabolism - other enzymes | Gene.8787 | 208 | UDPGT domain containing protein [Asbolus verrucosus] |
| map00983 Drug metabolism - other enzymes | Gene.8787 | 442 | UDPGT domain containing protein [Asbolus verrucosus] |
| map00983 Drug metabolism - other enzymes | Gene.5369 | 84 | PREDICTED: UMP-CMP kinase [Aethina tumida] |
| map00983 Drug metabolism - other enzymes | Gene.5369 | 82 | PREDICTED: UMP-CMP kinase [Aethina tumida] |
| map00983 Drug metabolism - other enzymes | Gene.3731 | 221 | PREDICTED: UDP-glucuronosyltransferase 2B2 [Tribolium castaneum] |
| map00983 Drug metabolism - other enzymes | Gene.7965 | 152 | dihydropyrimidinase [Anoplophora glabripennis] |
| map00983 Drug metabolism - other enzymes | Gene.7965 | 461 | dihydropyrimidinase [Anoplophora glabripennis] |
| map00983 Drug metabolism - other enzymes | Gene.7965 | 346 | dihydropyrimidinase [Anoplophora glabripennis] |
| map00983 Drug metabolism - other enzymes | Gene.3734 | 395 | PREDICTED: UDP-glucuronosyltransferase 2B7 isoform X1 [Tribolium castaneum] |
| map00983 Drug metabolism - other enzymes | Gene.3734 | 305 | PREDICTED: UDP-glucuronosyltransferase 2B7 isoform X1 [Tribolium castaneum] |
| map00983 Drug metabolism - other enzymes | Gene.3734 | 400 | PREDICTED: UDP-glucuronosyltransferase 2B7 isoform X1 [Tribolium castaneum] |
| map00983 Drug metabolism - other enzymes | Gene.3734 | 378 | PREDICTED: UDP-glucuronosyltransferase 2B7 isoform X1 [Tribolium castaneum] |
| map00983 Drug metabolism - other enzymes | Gene.3734 | 406 | PREDICTED: UDP-glucuronosyltransferase 2B7 isoform X1 [Tribolium castaneum] |
| map00983 Drug metabolism - other enzymes | Gene.2052 | 422 | "UDP-glucuronosyltransferase 2C1-like, partial [Asbolus verrucosus]" |
| map00983 Drug metabolism - other enzymes | Gene.6339 | 50 | PREDICTED: nucleoside diphosphate kinase isoform X1 [Tribolium castaneum] |
| map00983 Drug metabolism - other enzymes | Gene.6339 | 28 | PREDICTED: nucleoside diphosphate kinase isoform X1 [Tribolium castaneum] |
| map00983 Drug metabolism - other enzymes | Gene.6339 | 47 | PREDICTED: nucleoside diphosphate kinase isoform X1 [Tribolium castaneum] |
| map00983 Drug metabolism - other enzymes | Gene.6339 | 143 | PREDICTED: nucleoside diphosphate kinase isoform X1 [Tribolium castaneum] |
| map00983 Drug metabolism - other enzymes | Gene.4396 | 69 | PREDICTED: 2-hydroxyacylsphingosine 1-beta-galactosyltransferase-like [Tribolium castaneum] |
| map00983 Drug metabolism - other enzymes | Gene.4396 | 317 | PREDICTED: 2-hydroxyacylsphingosine 1-beta-galactosyltransferase-like [Tribolium castaneum] |
| map00983 Drug metabolism - other enzymes | Gene.4396 | 49 | PREDICTED: 2-hydroxyacylsphingosine 1-beta-galactosyltransferase-like [Tribolium castaneum] |
| map00983 Drug metabolism - other enzymes | Gene.4396 | 259 | PREDICTED: 2-hydroxyacylsphingosine 1-beta-galactosyltransferase-like [Tribolium castaneum] |
| map00983 Drug metabolism - other enzymes | Gene.425 | 128 | glutathione S-transferase 1-1 [Anoplophora glabripennis] |
| map00983 Drug metabolism - other enzymes | Gene.425 | 215 | glutathione S-transferase 1-1 [Anoplophora glabripennis] |
| map00983 Drug metabolism - other enzymes | Gene.425 | 131 | glutathione S-transferase 1-1 [Anoplophora glabripennis] |
| map00983 Drug metabolism - other enzymes | Gene.425 | 201 | glutathione S-transferase 1-1 [Anoplophora glabripennis] |
| map00983 Drug metabolism - other enzymes | Gene.425 | 207 | glutathione S-transferase 1-1 [Anoplophora glabripennis] |
| map00983 Drug metabolism - other enzymes | Gene.425 | 37 | glutathione S-transferase 1-1 [Anoplophora glabripennis] |
| map00983 Drug metabolism - other enzymes | Gene.425 | 186 | glutathione S-transferase 1-1 [Anoplophora glabripennis] |
| map00983 Drug metabolism - other enzymes | Gene.2140 | 401 | PREDICTED: UDP-glucuronosyltransferase 2B10-like [Tribolium castaneum] |
| map00983 Drug metabolism - other enzymes | Gene.2140 | 80 | PREDICTED: UDP-glucuronosyltransferase 2B10-like [Tribolium castaneum] |
| map00983 Drug metabolism - other enzymes | Gene.2140 | 425 | PREDICTED: UDP-glucuronosyltransferase 2B10-like [Tribolium castaneum] |
| map00983 Drug metabolism - other enzymes | Gene.2140 | 410 | PREDICTED: UDP-glucuronosyltransferase 2B10-like [Tribolium castaneum] |
| map00983 Drug metabolism - other enzymes | Gene.2176 | 48 | UDP-glucuronosyltransferase 1-9-like isoform X2 [Leptinotarsa decemlineata] |
| map00983 Drug metabolism - other enzymes | Gene.9040 | 57 | PREDICTED: microsomal glutathione S-transferase 1 [Tribolium castaneum] |
| map00983 Drug metabolism - other enzymes | Gene.9040 | 55 | PREDICTED: microsomal glutathione S-transferase 1 [Tribolium castaneum] |
| map00983 Drug metabolism - other enzymes | Gene.1050 | 121 | PREDICTED: 2-hydroxyacylsphingosine 1-beta-galactosyltransferase-like [Tribolium castaneum] |
| map00983 Drug metabolism - other enzymes | Gene.2175 | 192 | PREDICTED: 2-hydroxyacylsphingosine 1-beta-galactosyltransferase-like [Tribolium castaneum] |
| map00983 Drug metabolism - other enzymes | Gene.2178 | 47 | UDPGT and/or Glyco tran 28 C domain containing protein [Asbolus verrucosus] |
| map00471 D-Glutamine and D-glutamate metabolism | Gene.2236 | 516 | "Glutamate dehydrogenase, mitochondrial-like Protein [Tribolium castaneum]" |
| map00471 D-Glutamine and D-glutamate metabolism | Gene.2236 | 412 | "Glutamate dehydrogenase, mitochondrial-like Protein [Tribolium castaneum]" |
| map00471 D-Glutamine and D-glutamate metabolism | Gene.2236 | 492 | "Glutamate dehydrogenase, mitochondrial-like Protein [Tribolium castaneum]" |
| map00471 D-Glutamine and D-glutamate metabolism | Gene.2236 | 457 | "Glutamate dehydrogenase, mitochondrial-like Protein [Tribolium castaneum]" |
| map00471 D-Glutamine and D-glutamate metabolism | Gene.2236 | 87 | "Glutamate dehydrogenase, mitochondrial-like Protein [Tribolium castaneum]" |
| map00471 D-Glutamine and D-glutamate metabolism | Gene.2236 | 186 | "Glutamate dehydrogenase, mitochondrial-like Protein [Tribolium castaneum]" |
| map00471 D-Glutamine and D-glutamate metabolism | Gene.2236 | 500 | "Glutamate dehydrogenase, mitochondrial-like Protein [Tribolium castaneum]" |
| map00471 D-Glutamine and D-glutamate metabolism | Gene.2236 | 372 | "Glutamate dehydrogenase, mitochondrial-like Protein [Tribolium castaneum]" |
| map00471 D-Glutamine and D-glutamate metabolism | Gene.2236 | 477 | "Glutamate dehydrogenase, mitochondrial-like Protein [Tribolium castaneum]" |
| map00471 D-Glutamine and D-glutamate metabolism | Gene.2236 | 195 | "Glutamate dehydrogenase, mitochondrial-like Protein [Tribolium castaneum]" |
| map00471 D-Glutamine and D-glutamate metabolism | Gene.2236 | 82 | "Glutamate dehydrogenase, mitochondrial-like Protein [Tribolium castaneum]" |
| map04931 Insulin resistance | Gene.1845 | 92 | PREDICTED: glycogen synthase kinase-3 beta isoform X10 [Tribolium castaneum] |
| map04931 Insulin resistance | Gene.1845 | 87 | PREDICTED: glycogen synthase kinase-3 beta isoform X10 [Tribolium castaneum] |
| map04931 Insulin resistance | Gene.8017 | 315 | glycogen synthase [Harmonia axyridis] |
| map04931 Insulin resistance | Gene.8017 | 298 | glycogen synthase [Harmonia axyridis] |
| map04931 Insulin resistance | Gene.8017 | 247 | glycogen synthase [Harmonia axyridis] |
| map04931 Insulin resistance | Gene.4419 | 291 | glycogen phosphorylase [Harmonia axyridis] |
| map04931 Insulin resistance | Gene.4419 | 177 | glycogen phosphorylase [Harmonia axyridis] |
| map04931 Insulin resistance | Gene.4419 | 10 | glycogen phosphorylase [Harmonia axyridis] |
| map04931 Insulin resistance | Gene.4419 | 29 | glycogen phosphorylase [Harmonia axyridis] |
| map04931 Insulin resistance | Gene.4419 | 249 | glycogen phosphorylase [Harmonia axyridis] |
| map04931 Insulin resistance | Gene.4419 | 78 | glycogen phosphorylase [Harmonia axyridis] |
| map04931 Insulin resistance | Gene.4417 | 92 | glycogen phosphorylase [Harmonia axyridis] |
| map04931 Insulin resistance | Gene.4417 | 205 | glycogen phosphorylase [Harmonia axyridis] |
| map04931 Insulin resistance | Gene.4417 | 120 | glycogen phosphorylase [Harmonia axyridis] |
| map04931 Insulin resistance | Gene.4417 | 134 | glycogen phosphorylase [Harmonia axyridis] |
| map04931 Insulin resistance | Gene.4417 | 44 | glycogen phosphorylase [Harmonia axyridis] |
| map04931 Insulin resistance | Gene.4417 | 288 | glycogen phosphorylase [Harmonia axyridis] |
| map04931 Insulin resistance | Gene.4417 | 113 | glycogen phosphorylase [Harmonia axyridis] |
| map04931 Insulin resistance | Gene.931 | 184 | PREDICTED: glutamine--fructose-6-phosphate aminotransferase [isomerizing] 2 isoform X1 [Tribolium castaneum] |
| map04931 Insulin resistance | Gene.502 | 147 | "acid phosphatase, partial [Cryptolaemus montrouzieri]" |
| map04931 Insulin resistance | Gene.502 | 141 | "acid phosphatase, partial [Cryptolaemus montrouzieri]" |
| map04931 Insulin resistance | Gene.502 | 260 | "acid phosphatase, partial [Cryptolaemus montrouzieri]" |
| map04931 Insulin resistance | Gene.3247 | 93 | PREDICTED: phosphoenolpyruvate carboxykinase [GTP] isoform X1 [Tribolium castaneum] |
| map04213 Longevity regulating pathway - multiple species | Gene.5965 | 63 | "PREDICTED: superoxide dismutase [Mn] 1, mitochondrial-like [Aethina tumida]" |
| map04213 Longevity regulating pathway - multiple species | Gene.5965 | 68 | "PREDICTED: superoxide dismutase [Mn] 1, mitochondrial-like [Aethina tumida]" |
| map04213 Longevity regulating pathway - multiple species | Gene.7565 | 74 | heat shock protein 21.62 [Harmonia axyridis] |
| map04213 Longevity regulating pathway - multiple species | Gene.7565 | 106 | heat shock protein 21.62 [Harmonia axyridis] |
| map04213 Longevity regulating pathway - multiple species | Gene.7565 | 129 | heat shock protein 21.62 [Harmonia axyridis] |
| map04213 Longevity regulating pathway - multiple species | Gene.7565 | 135 | heat shock protein 21.62 [Harmonia axyridis] |
| map04213 Longevity regulating pathway - multiple species | Gene.1391 | 78 | PREDICTED: protein lethal(2)essential for life [Tribolium castaneum] |
| map04213 Longevity regulating pathway - multiple species | Gene.5709 | 26 | cAMP-dependent protein kinase catalytic subunit [Agrilus planipennis] |
| map04213 Longevity regulating pathway - multiple species | Gene.5709 | 257 | cAMP-dependent protein kinase catalytic subunit [Agrilus planipennis] |
| map04213 Longevity regulating pathway - multiple species | Gene.4276 | 74 | "superoxide dismutase, partial [Harmonia axyridis]" |
| map04213 Longevity regulating pathway - multiple species | Gene.4276 | 152 | "superoxide dismutase, partial [Harmonia axyridis]" |
| map04213 Longevity regulating pathway - multiple species | Gene.4276 | 4 | "superoxide dismutase, partial [Harmonia axyridis]" |
| map04213 Longevity regulating pathway - multiple species | Gene.4276 | 134 | "superoxide dismutase, partial [Harmonia axyridis]" |
| map04213 Longevity regulating pathway - multiple species | Gene.4276 | 106 | "superoxide dismutase, partial [Harmonia axyridis]" |
| map04213 Longevity regulating pathway - multiple species | Gene.8326 | 104 | heat shock protein 16.25 [Harmonia axyridis] |
| map04213 Longevity regulating pathway - multiple species | Gene.6759 | 54 | hypothetical protein AMK59_2413 [Oryctes borbonicus] |
| map04213 Longevity regulating pathway - multiple species | Gene.5542 | 28 | catalase [Onthophagus taurus] |
| map04213 Longevity regulating pathway - multiple species | Gene.5542 | 179 | catalase [Onthophagus taurus] |
| map04213 Longevity regulating pathway - multiple species | Gene.5542 | 325 | catalase [Onthophagus taurus] |
| map04213 Longevity regulating pathway - multiple species | Gene.5542 | 247 | catalase [Onthophagus taurus] |
| map04213 Longevity regulating pathway - multiple species | Gene.5542 | 438 | catalase [Onthophagus taurus] |
| map04213 Longevity regulating pathway - multiple species | Gene.5542 | 48 | catalase [Onthophagus taurus] |
| map04213 Longevity regulating pathway - multiple species | Gene.1895 | 365 | heat shock protein 70 [Harmonia axyridis] |
| map04213 Longevity regulating pathway - multiple species | Gene.1895 | 271 | heat shock protein 70 [Harmonia axyridis] |
| map04213 Longevity regulating pathway - multiple species | Gene.1895 | 26 | heat shock protein 70 [Harmonia axyridis] |
| map04213 Longevity regulating pathway - multiple species | Gene.1895 | 475 | heat shock protein 70 [Harmonia axyridis] |
| map04213 Longevity regulating pathway - multiple species | Gene.1895 | 438 | heat shock protein 70 [Harmonia axyridis] |
| map04213 Longevity regulating pathway - multiple species | Gene.1895 | 42 | heat shock protein 70 [Harmonia axyridis] |
| map04213 Longevity regulating pathway - multiple species | Gene.1895 | 22 | heat shock protein 70 [Harmonia axyridis] |
| map04213 Longevity regulating pathway - multiple species | Gene.1895 | 473 | heat shock protein 70 [Harmonia axyridis] |
| map04213 Longevity regulating pathway - multiple species | Gene.1895 | 233 | heat shock protein 70 [Harmonia axyridis] |
| map04213 Longevity regulating pathway - multiple species | Gene.1895 | 426 | heat shock protein 70 [Harmonia axyridis] |
| map04213 Longevity regulating pathway - multiple species | Gene.1895 | 40 | heat shock protein 70 [Harmonia axyridis] |
| map04213 Longevity regulating pathway - multiple species | Gene.1895 | 509 | heat shock protein 70 [Harmonia axyridis] |
| map04213 Longevity regulating pathway - multiple species | Gene.1895 | 160 | heat shock protein 70 [Harmonia axyridis] |
| map04213 Longevity regulating pathway - multiple species | Gene.1895 | 421 | heat shock protein 70 [Harmonia axyridis] |
| map04213 Longevity regulating pathway - multiple species | Gene.1895 | 414 | heat shock protein 70 [Harmonia axyridis] |
| map04213 Longevity regulating pathway - multiple species | Gene.1895 | 242 | heat shock protein 70 [Harmonia axyridis] |
| map00100 Steroid biosynthesis | Gene.4453 | 14 | lipase 3 [Cephus cinctus] |
| map04218 Cellular senescence | Gene.298 | 116 | "Csa-calmodulin 3, partial [Cupiennius salei]" |
| map04218 Cellular senescence | Gene.298 | 95 | "Csa-calmodulin 3, partial [Cupiennius salei]" |
| map04218 Cellular senescence | Gene.298 | 31 | "Csa-calmodulin 3, partial [Cupiennius salei]" |
| map04218 Cellular senescence | Gene.298 | 78 | "Csa-calmodulin 3, partial [Cupiennius salei]" |
| map04218 Cellular senescence | Gene.8096 | 274 | "PREDICTED: ADP,ATP carrier protein 1 [Tribolium castaneum]" |
| map04218 Cellular senescence | Gene.8096 | 74 | "PREDICTED: ADP,ATP carrier protein 1 [Tribolium castaneum]" |
| map04218 Cellular senescence | Gene.8096 | 271 | "PREDICTED: ADP,ATP carrier protein 1 [Tribolium castaneum]" |
| map04218 Cellular senescence | Gene.8096 | 21 | "PREDICTED: ADP,ATP carrier protein 1 [Tribolium castaneum]" |
| map04218 Cellular senescence | Gene.8096 | 63 | "PREDICTED: ADP,ATP carrier protein 1 [Tribolium castaneum]" |
| map04218 Cellular senescence | Gene.8096 | 263 | "PREDICTED: ADP,ATP carrier protein 1 [Tribolium castaneum]" |
| map04218 Cellular senescence | Gene.8096 | 107 | "PREDICTED: ADP,ATP carrier protein 1 [Tribolium castaneum]" |
| map04218 Cellular senescence | Gene.8096 | 158 | "PREDICTED: ADP,ATP carrier protein 1 [Tribolium castaneum]" |
| map04218 Cellular senescence | Gene.8096 | 60 | "PREDICTED: ADP,ATP carrier protein 1 [Tribolium castaneum]" |
| map04218 Cellular senescence | Gene.8096 | 177 | "PREDICTED: ADP,ATP carrier protein 1 [Tribolium castaneum]" |
| map04218 Cellular senescence | Gene.8096 | 257 | "PREDICTED: ADP,ATP carrier protein 1 [Tribolium castaneum]" |
| map04218 Cellular senescence | Gene.8096 | 54 | "PREDICTED: ADP,ATP carrier protein 1 [Tribolium castaneum]" |
| map04218 Cellular senescence | Gene.5803 | 20 | calcium-binding protein E63-1 [Anoplophora glabripennis] |
| map04218 Cellular senescence | Gene.8098 | 93 | "hypothetical protein, partial [Rhynchophorus ferrugineus]" |
| map04218 Cellular senescence | Gene.8098 | 53 | "hypothetical protein, partial [Rhynchophorus ferrugineus]" |
| map04218 Cellular senescence | Gene.8098 | 64 | "hypothetical protein, partial [Rhynchophorus ferrugineus]" |
| map04218 Cellular senescence | Gene.8098 | 106 | "hypothetical protein, partial [Rhynchophorus ferrugineus]" |
| map04218 Cellular senescence | Gene.8098 | 97 | "hypothetical protein, partial [Rhynchophorus ferrugineus]" |
| map04218 Cellular senescence | Gene.8098 | 148 | "hypothetical protein, partial [Rhynchophorus ferrugineus]" |
| map04218 Cellular senescence | Gene.8098 | 167 | "hypothetical protein, partial [Rhynchophorus ferrugineus]" |
| map04218 Cellular senescence | Gene.502 | 147 | "acid phosphatase, partial [Cryptolaemus montrouzieri]" |
| map04218 Cellular senescence | Gene.502 | 141 | "acid phosphatase, partial [Cryptolaemus montrouzieri]" |
| map04218 Cellular senescence | Gene.502 | 260 | "acid phosphatase, partial [Cryptolaemus montrouzieri]" |
| map04218 Cellular senescence | Gene.8407 | 27 | PREDICTED: voltage-dependent anion-selective channel isoform X1 [Tribolium castaneum] |
| map04218 Cellular senescence | Gene.8407 | 11 | PREDICTED: voltage-dependent anion-selective channel isoform X1 [Tribolium castaneum] |
| map04218 Cellular senescence | Gene.8407 | 60 | PREDICTED: voltage-dependent anion-selective channel isoform X1 [Tribolium castaneum] |
| map04218 Cellular senescence | Gene.8407 | 64 | PREDICTED: voltage-dependent anion-selective channel isoform X1 [Tribolium castaneum] |
| map04919 Thyroid hormone signaling pathway | Gene.5709 | 26 | cAMP-dependent protein kinase catalytic subunit [Agrilus planipennis] |
| map04919 Thyroid hormone signaling pathway | Gene.5709 | 257 | cAMP-dependent protein kinase catalytic subunit [Agrilus planipennis] |
| map04919 Thyroid hormone signaling pathway | Gene.1845 | 92 | PREDICTED: glycogen synthase kinase-3 beta isoform X10 [Tribolium castaneum] |
| map04919 Thyroid hormone signaling pathway | Gene.1845 | 87 | PREDICTED: glycogen synthase kinase-3 beta isoform X10 [Tribolium castaneum] |
| map04919 Thyroid hormone signaling pathway | Gene.4352 | 128 | PREDICTED: calcium-transporting ATPase sarcoplasmic/endoplasmic reticulum type isoform X1 [Tribolium castaneum] |
| map04919 Thyroid hormone signaling pathway | Gene.4352 | 514 | PREDICTED: calcium-transporting ATPase sarcoplasmic/endoplasmic reticulum type isoform X1 [Tribolium castaneum] |
| map04919 Thyroid hormone signaling pathway | Gene.4352 | 30 | PREDICTED: calcium-transporting ATPase sarcoplasmic/endoplasmic reticulum type isoform X1 [Tribolium castaneum] |
| map04919 Thyroid hormone signaling pathway | Gene.2599 | 765 | PREDICTED: sodium/potassium-transporting ATPase subunit alpha isoform X3 [Tribolium castaneum] |
| map04919 Thyroid hormone signaling pathway | Gene.172 | 216 | "beta-actin, partial [Cotesia chilonis]" |
| map04919 Thyroid hormone signaling pathway | Gene.172 | 51 | "beta-actin, partial [Cotesia chilonis]" |
| map04919 Thyroid hormone signaling pathway | Gene.172 | 114 | "beta-actin, partial [Cotesia chilonis]" |
| map04919 Thyroid hormone signaling pathway | Gene.172 | 62 | "beta-actin, partial [Cotesia chilonis]" |
| map04919 Thyroid hormone signaling pathway | Gene.4973 | 1173 | "PREDICTED: myosin heavy chain, muscle isoform X18 [Tribolium castaneum]" |
| map04919 Thyroid hormone signaling pathway | Gene.4973 | 1579 | "PREDICTED: myosin heavy chain, muscle isoform X18 [Tribolium castaneum]" |
| map04919 Thyroid hormone signaling pathway | Gene.4973 | 1457 | "PREDICTED: myosin heavy chain, muscle isoform X18 [Tribolium castaneum]" |
| map04919 Thyroid hormone signaling pathway | Gene.4973 | 1332 | "PREDICTED: myosin heavy chain, muscle isoform X18 [Tribolium castaneum]" |
| map04919 Thyroid hormone signaling pathway | Gene.4973 | 907 | "PREDICTED: myosin heavy chain, muscle isoform X18 [Tribolium castaneum]" |
| map04919 Thyroid hormone signaling pathway | Gene.4973 | 1641 | "PREDICTED: myosin heavy chain, muscle isoform X18 [Tribolium castaneum]" |
| map04919 Thyroid hormone signaling pathway | Gene.4973 | 599 | "PREDICTED: myosin heavy chain, muscle isoform X18 [Tribolium castaneum]" |
| map04919 Thyroid hormone signaling pathway | Gene.4973 | 1390 | "PREDICTED: myosin heavy chain, muscle isoform X18 [Tribolium castaneum]" |
| map04919 Thyroid hormone signaling pathway | Gene.4973 | 84 | "PREDICTED: myosin heavy chain, muscle isoform X18 [Tribolium castaneum]" |
| map04919 Thyroid hormone signaling pathway | Gene.4973 | 1005 | "PREDICTED: myosin heavy chain, muscle isoform X18 [Tribolium castaneum]" |
| map04919 Thyroid hormone signaling pathway | Gene.4973 | 1919 | "PREDICTED: myosin heavy chain, muscle isoform X18 [Tribolium castaneum]" |
| map04919 Thyroid hormone signaling pathway | Gene.4973 | 895 | "PREDICTED: myosin heavy chain, muscle isoform X18 [Tribolium castaneum]" |
| map04919 Thyroid hormone signaling pathway | Gene.4973 | 450 | "PREDICTED: myosin heavy chain, muscle isoform X18 [Tribolium castaneum]" |
| map04919 Thyroid hormone signaling pathway | Gene.4973 | 551 | "PREDICTED: myosin heavy chain, muscle isoform X18 [Tribolium castaneum]" |
| map04919 Thyroid hormone signaling pathway | Gene.4973 | 879 | "PREDICTED: myosin heavy chain, muscle isoform X18 [Tribolium castaneum]" |
| map04919 Thyroid hormone signaling pathway | Gene.4973 | 1416 | "PREDICTED: myosin heavy chain, muscle isoform X18 [Tribolium castaneum]" |
| map04919 Thyroid hormone signaling pathway | Gene.4973 | 611 | "PREDICTED: myosin heavy chain, muscle isoform X18 [Tribolium castaneum]" |
| map04919 Thyroid hormone signaling pathway | Gene.4973 | 1109 | "PREDICTED: myosin heavy chain, muscle isoform X18 [Tribolium castaneum]" |
| map04919 Thyroid hormone signaling pathway | Gene.4973 | 721 | "PREDICTED: myosin heavy chain, muscle isoform X18 [Tribolium castaneum]" |
| map04919 Thyroid hormone signaling pathway | Gene.4973 | 1838 | "PREDICTED: myosin heavy chain, muscle isoform X18 [Tribolium castaneum]" |
| map04919 Thyroid hormone signaling pathway | Gene.4973 | 1262 | "PREDICTED: myosin heavy chain, muscle isoform X18 [Tribolium castaneum]" |
| map04919 Thyroid hormone signaling pathway | Gene.4973 | 1444 | "PREDICTED: myosin heavy chain, muscle isoform X18 [Tribolium castaneum]" |
| map04919 Thyroid hormone signaling pathway | Gene.4973 | 1277 | "PREDICTED: myosin heavy chain, muscle isoform X18 [Tribolium castaneum]" |
| map04919 Thyroid hormone signaling pathway | Gene.4973 | 1247 | "PREDICTED: myosin heavy chain, muscle isoform X18 [Tribolium castaneum]" |
| map04919 Thyroid hormone signaling pathway | Gene.4973 | 971 | "PREDICTED: myosin heavy chain, muscle isoform X18 [Tribolium castaneum]" |
| map04919 Thyroid hormone signaling pathway | Gene.4973 | 851 | "PREDICTED: myosin heavy chain, muscle isoform X18 [Tribolium castaneum]" |
| map04919 Thyroid hormone signaling pathway | Gene.4973 | 1316 | "PREDICTED: myosin heavy chain, muscle isoform X18 [Tribolium castaneum]" |
| map04919 Thyroid hormone signaling pathway | Gene.4973 | 1374 | "PREDICTED: myosin heavy chain, muscle isoform X18 [Tribolium castaneum]" |
| map04919 Thyroid hormone signaling pathway | Gene.4973 | 951 | "PREDICTED: myosin heavy chain, muscle isoform X18 [Tribolium castaneum]" |
| map04919 Thyroid hormone signaling pathway | Gene.4973 | 1354 | "PREDICTED: myosin heavy chain, muscle isoform X18 [Tribolium castaneum]" |
| map04919 Thyroid hormone signaling pathway | Gene.4973 | 429 | "PREDICTED: myosin heavy chain, muscle isoform X18 [Tribolium castaneum]" |
| map04919 Thyroid hormone signaling pathway | Gene.4973 | 1103 | "PREDICTED: myosin heavy chain, muscle isoform X18 [Tribolium castaneum]" |
| map04919 Thyroid hormone signaling pathway | Gene.4973 | 1093 | "PREDICTED: myosin heavy chain, muscle isoform X18 [Tribolium castaneum]" |
| map04919 Thyroid hormone signaling pathway | Gene.4973 | 1451 | "PREDICTED: myosin heavy chain, muscle isoform X18 [Tribolium castaneum]" |
| map04919 Thyroid hormone signaling pathway | Gene.4973 | 940 | "PREDICTED: myosin heavy chain, muscle isoform X18 [Tribolium castaneum]" |
| map04919 Thyroid hormone signaling pathway | Gene.4973 | 1791 | "PREDICTED: myosin heavy chain, muscle isoform X18 [Tribolium castaneum]" |
| map04919 Thyroid hormone signaling pathway | Gene.2646 | 99 | beta actin [Polyrhachis vicina] |
| map04919 Thyroid hormone signaling pathway | Gene.2646 | 88 | beta actin [Polyrhachis vicina] |
| map04919 Thyroid hormone signaling pathway | Gene.2646 | 64 | beta actin [Polyrhachis vicina] |
| map04919 Thyroid hormone signaling pathway | Gene.2646 | 101 | beta actin [Polyrhachis vicina] |
| map00770 Pantothenate and CoA biosynthesis | Gene.7965 | 152 | dihydropyrimidinase [Anoplophora glabripennis] |
| map00770 Pantothenate and CoA biosynthesis | Gene.7965 | 461 | dihydropyrimidinase [Anoplophora glabripennis] |
| map00770 Pantothenate and CoA biosynthesis | Gene.7965 | 346 | dihydropyrimidinase [Anoplophora glabripennis] |
| map00770 Pantothenate and CoA biosynthesis | Gene.2689 | 127 | CoaE and/or CTP transf 2 domain containing protein [Asbolus verrucosus] |
| map00770 Pantothenate and CoA biosynthesis | Gene.2689 | 154 | CoaE and/or CTP transf 2 domain containing protein [Asbolus verrucosus] |
| map00770 Pantothenate and CoA biosynthesis | Gene.2014 | 118 | "hypothetical protein AMK59_1391, partial [Oryctes borbonicus]" |
| map00770 Pantothenate and CoA biosynthesis | Gene.2014 | 425 | "hypothetical protein AMK59_1391, partial [Oryctes borbonicus]" |
| map05031 Amphetamine addiction | Gene.5709 | 26 | cAMP-dependent protein kinase catalytic subunit [Agrilus planipennis] |
| map05031 Amphetamine addiction | Gene.5709 | 257 | cAMP-dependent protein kinase catalytic subunit [Agrilus planipennis] |
| map05031 Amphetamine addiction | Gene.298 | 116 | "Csa-calmodulin 3, partial [Cupiennius salei]" |
| map05031 Amphetamine addiction | Gene.298 | 95 | "Csa-calmodulin 3, partial [Cupiennius salei]" |
| map05031 Amphetamine addiction | Gene.298 | 31 | "Csa-calmodulin 3, partial [Cupiennius salei]" |
| map05031 Amphetamine addiction | Gene.298 | 78 | "Csa-calmodulin 3, partial [Cupiennius salei]" |
| map05031 Amphetamine addiction | Gene.5803 | 20 | calcium-binding protein E63-1 [Anoplophora glabripennis] |
| map05031 Amphetamine addiction | Gene.502 | 147 | "acid phosphatase, partial [Cryptolaemus montrouzieri]" |
| map05031 Amphetamine addiction | Gene.502 | 141 | "acid phosphatase, partial [Cryptolaemus montrouzieri]" |
| map05031 Amphetamine addiction | Gene.502 | 260 | "acid phosphatase, partial [Cryptolaemus montrouzieri]" |
| map04714 Thermogenesis | Gene.1542 | 237 | "PREDICTED: ATP synthase subunit alpha, mitochondrial [Tribolium castaneum]" |
| map04714 Thermogenesis | Gene.1542 | 130 | "PREDICTED: ATP synthase subunit alpha, mitochondrial [Tribolium castaneum]" |
| map04714 Thermogenesis | Gene.1542 | 63 | "PREDICTED: ATP synthase subunit alpha, mitochondrial [Tribolium castaneum]" |
| map04714 Thermogenesis | Gene.1542 | 165 | "PREDICTED: ATP synthase subunit alpha, mitochondrial [Tribolium castaneum]" |
| map04714 Thermogenesis | Gene.1542 | 71 | "PREDICTED: ATP synthase subunit alpha, mitochondrial [Tribolium castaneum]" |
| map04714 Thermogenesis | Gene.1542 | 314 | "PREDICTED: ATP synthase subunit alpha, mitochondrial [Tribolium castaneum]" |
| map04714 Thermogenesis | Gene.1542 | 537 | "PREDICTED: ATP synthase subunit alpha, mitochondrial [Tribolium castaneum]" |
| map04714 Thermogenesis | Gene.1542 | 514 | "PREDICTED: ATP synthase subunit alpha, mitochondrial [Tribolium castaneum]" |
| map04714 Thermogenesis | Gene.1542 | 526 | "PREDICTED: ATP synthase subunit alpha, mitochondrial [Tribolium castaneum]" |
| map04714 Thermogenesis | Gene.1542 | 422 | "PREDICTED: ATP synthase subunit alpha, mitochondrial [Tribolium castaneum]" |
| map04714 Thermogenesis | Gene.1542 | 159 | "PREDICTED: ATP synthase subunit alpha, mitochondrial [Tribolium castaneum]" |
| map04714 Thermogenesis | Gene.1542 | 228 | "PREDICTED: ATP synthase subunit alpha, mitochondrial [Tribolium castaneum]" |
| map04714 Thermogenesis | Gene.1542 | 303 | "PREDICTED: ATP synthase subunit alpha, mitochondrial [Tribolium castaneum]" |
| map04714 Thermogenesis | Gene.1542 | 518 | "PREDICTED: ATP synthase subunit alpha, mitochondrial [Tribolium castaneum]" |
| map04714 Thermogenesis | Gene.1542 | 425 | "PREDICTED: ATP synthase subunit alpha, mitochondrial [Tribolium castaneum]" |
| map04714 Thermogenesis | Gene.1542 | 501 | "PREDICTED: ATP synthase subunit alpha, mitochondrial [Tribolium castaneum]" |
| map04714 Thermogenesis | Gene.1542 | 529 | "PREDICTED: ATP synthase subunit alpha, mitochondrial [Tribolium castaneum]" |
| map04714 Thermogenesis | Gene.1542 | 432 | "PREDICTED: ATP synthase subunit alpha, mitochondrial [Tribolium castaneum]" |
| map04714 Thermogenesis | Gene.1542 | 507 | "PREDICTED: ATP synthase subunit alpha, mitochondrial [Tribolium castaneum]" |
| map04714 Thermogenesis | Gene.1542 | 496 | "PREDICTED: ATP synthase subunit alpha, mitochondrial [Tribolium castaneum]" |
| map04714 Thermogenesis | Gene.5910 | 270 | "PREDICTED: NADH dehydrogenase [ubiquinone] 1 alpha subcomplex subunit 10, mitochondrial [Tribolium castaneum]" |
| map04714 Thermogenesis | Gene.4302 | 243 | long-chain-fatty-acid--CoA ligase 4 isoform X1 [Agrilus planipennis] |
| map04714 Thermogenesis | Gene.4302 | 416 | long-chain-fatty-acid--CoA ligase 4 isoform X1 [Agrilus planipennis] |
| map04714 Thermogenesis | Gene.4302 | 411 | long-chain-fatty-acid--CoA ligase 4 isoform X1 [Agrilus planipennis] |
| map04714 Thermogenesis | Gene.4302 | 263 | long-chain-fatty-acid--CoA ligase 4 isoform X1 [Agrilus planipennis] |
| map04714 Thermogenesis | Gene.742 | 137 | "NADH dehydrogenase [ubiquinone] 1 alpha subcomplex subunit 9, mitochondrial [Anoplophora glabripennis]" |
| map04714 Thermogenesis | Gene.742 | 278 | "NADH dehydrogenase [ubiquinone] 1 alpha subcomplex subunit 9, mitochondrial [Anoplophora glabripennis]" |
| map04714 Thermogenesis | Gene.742 | 142 | "NADH dehydrogenase [ubiquinone] 1 alpha subcomplex subunit 9, mitochondrial [Anoplophora glabripennis]" |
| map04714 Thermogenesis | Gene.742 | 268 | "NADH dehydrogenase [ubiquinone] 1 alpha subcomplex subunit 9, mitochondrial [Anoplophora glabripennis]" |
| map04714 Thermogenesis | Gene.5885 | 94 | NADH dehydrogenase [ubiquinone] 1 alpha subcomplex subunit 6 [Onthophagus taurus] |
| map04714 Thermogenesis | Gene.7655 | 158 | "ATP synthase subunit b, mitochondrial [Leptinotarsa decemlineata]" |
| map04714 Thermogenesis | Gene.7655 | 119 | "ATP synthase subunit b, mitochondrial [Leptinotarsa decemlineata]" |
| map04714 Thermogenesis | Gene.7655 | 170 | "ATP synthase subunit b, mitochondrial [Leptinotarsa decemlineata]" |
| map04714 Thermogenesis | Gene.7655 | 233 | "ATP synthase subunit b, mitochondrial [Leptinotarsa decemlineata]" |
| map04714 Thermogenesis | Gene.7655 | 154 | "ATP synthase subunit b, mitochondrial [Leptinotarsa decemlineata]" |
| map04714 Thermogenesis | Gene.7655 | 53 | "ATP synthase subunit b, mitochondrial [Leptinotarsa decemlineata]" |
| map04714 Thermogenesis | Gene.7655 | 194 | "ATP synthase subunit b, mitochondrial [Leptinotarsa decemlineata]" |
| map04714 Thermogenesis | Gene.143 | 69 | cytochrome c oxidase subunit IV [Tribolium castaneum] |
| map04714 Thermogenesis | Gene.143 | 180 | cytochrome c oxidase subunit IV [Tribolium castaneum] |
| map04714 Thermogenesis | Gene.143 | 94 | cytochrome c oxidase subunit IV [Tribolium castaneum] |
| map04714 Thermogenesis | Gene.143 | 184 | cytochrome c oxidase subunit IV [Tribolium castaneum] |
| map04714 Thermogenesis | Gene.2034 | 471 | "NADH dehydrogenase [ubiquinone] flavoprotein 1, mitochondrial [Tribolium castaneum]" |
| map04714 Thermogenesis | Gene.2034 | 381 | "NADH dehydrogenase [ubiquinone] flavoprotein 1, mitochondrial [Tribolium castaneum]" |
| map04714 Thermogenesis | Gene.2034 | 47 | "NADH dehydrogenase [ubiquinone] flavoprotein 1, mitochondrial [Tribolium castaneum]" |
| map04714 Thermogenesis | Gene.6325 | 67 | uncharacterized protein Dvir_GJ22112 [Drosophila virilis] |
| map04714 Thermogenesis | Gene.6325 | 26 | uncharacterized protein Dvir_GJ22112 [Drosophila virilis] |
| map04714 Thermogenesis | Gene.8016 | 180 | "UCR TM, Rieske, and/or Ubiq-Cytc-red N domain containing protein [Asbolus verrucosus]" |
| map04714 Thermogenesis | Gene.8016 | 171 | "UCR TM, Rieske, and/or Ubiq-Cytc-red N domain containing protein [Asbolus verrucosus]" |
| map04714 Thermogenesis | Gene.8016 | 110 | "UCR TM, Rieske, and/or Ubiq-Cytc-red N domain containing protein [Asbolus verrucosus]" |
| map04714 Thermogenesis | Gene.8016 | 176 | "UCR TM, Rieske, and/or Ubiq-Cytc-red N domain containing protein [Asbolus verrucosus]" |
| map04714 Thermogenesis | Gene.6135 | 95 | PREDICTED: NADH dehydrogenase [ubiquinone] 1 alpha subcomplex subunit 13-like [Aethina tumida] |
| map04714 Thermogenesis | Gene.7840 | 189 | "PREDICTED: cytochrome c1, heme protein, mitochondrial [Tribolium castaneum]" |
| map04714 Thermogenesis | Gene.7840 | 44 | "PREDICTED: cytochrome c1, heme protein, mitochondrial [Tribolium castaneum]" |
| map04714 Thermogenesis | Gene.172 | 216 | "beta-actin, partial [Cotesia chilonis]" |
| map04714 Thermogenesis | Gene.172 | 51 | "beta-actin, partial [Cotesia chilonis]" |
| map04714 Thermogenesis | Gene.172 | 114 | "beta-actin, partial [Cotesia chilonis]" |
| map04714 Thermogenesis | Gene.172 | 62 | "beta-actin, partial [Cotesia chilonis]" |
| map04714 Thermogenesis | Gene.2646 | 99 | beta actin [Polyrhachis vicina] |
| map04714 Thermogenesis | Gene.2646 | 88 | beta actin [Polyrhachis vicina] |
| map04714 Thermogenesis | Gene.2646 | 64 | beta actin [Polyrhachis vicina] |
| map04714 Thermogenesis | Gene.2646 | 101 | beta actin [Polyrhachis vicina] |
| map04714 Thermogenesis | Gene.8261 | 92 | PREDICTED: cytochrome c oxidase subunit 6C-like [Dendroctonus ponderosae] |
| map04714 Thermogenesis | Gene.8261 | 88 | PREDICTED: cytochrome c oxidase subunit 6C-like [Dendroctonus ponderosae] |
| map04714 Thermogenesis | Gene.8261 | 40 | PREDICTED: cytochrome c oxidase subunit 6C-like [Dendroctonus ponderosae] |
| map04714 Thermogenesis | Gene.5746 | 137 | "PREDICTED: ATP synthase subunit delta, mitochondrial [Tribolium castaneum]" |
| map04714 Thermogenesis | Gene.3370 | 40 | PREDICTED: probable NADH dehydrogenase [ubiquinone] 1 alpha subcomplex subunit 12 [Tribolium castaneum] |
| map04714 Thermogenesis | Gene.3370 | 48 | PREDICTED: probable NADH dehydrogenase [ubiquinone] 1 alpha subcomplex subunit 12 [Tribolium castaneum] |
| map04714 Thermogenesis | Gene.8081 | 119 | "ATP synthase subunit gamma, mitochondrial [Leptinotarsa decemlineata]" |
| map04714 Thermogenesis | Gene.8081 | 125 | "ATP synthase subunit gamma, mitochondrial [Leptinotarsa decemlineata]" |
| map04714 Thermogenesis | Gene.8081 | 111 | "ATP synthase subunit gamma, mitochondrial [Leptinotarsa decemlineata]" |
| map04714 Thermogenesis | Gene.8081 | 78 | "ATP synthase subunit gamma, mitochondrial [Leptinotarsa decemlineata]" |
| map04714 Thermogenesis | Gene.8081 | 54 | "ATP synthase subunit gamma, mitochondrial [Leptinotarsa decemlineata]" |
| map04714 Thermogenesis | Gene.6318 | 57 | NADH dehydrogenase [ubiquinone] 1 beta subcomplex subunit 3 [Leptinotarsa decemlineata] |
| map04714 Thermogenesis | Gene.6318 | 43 | NADH dehydrogenase [ubiquinone] 1 beta subcomplex subunit 3 [Leptinotarsa decemlineata] |
| map04714 Thermogenesis | Gene.6318 | 23 | NADH dehydrogenase [ubiquinone] 1 beta subcomplex subunit 3 [Leptinotarsa decemlineata] |
| map04714 Thermogenesis | Gene.8337 | 50 | "PREDICTED: cytochrome c oxidase subunit 5B, mitochondrial [Tribolium castaneum]" |
| map04714 Thermogenesis | Gene.8337 | 44 | "PREDICTED: cytochrome c oxidase subunit 5B, mitochondrial [Tribolium castaneum]" |
| map04714 Thermogenesis | Gene.6252 | 60 | NADH dehydrogenase [ubiquinone] 1 beta subcomplex subunit 4 [Leptinotarsa decemlineata] |
| map04714 Thermogenesis | Gene.6252 | 111 | NADH dehydrogenase [ubiquinone] 1 beta subcomplex subunit 4 [Leptinotarsa decemlineata] |
| map04714 Thermogenesis | Gene.2624 | 70 | PREDICTED: NADH dehydrogenase [ubiquinone] 1 alpha subcomplex subunit 5 [Tribolium castaneum] |
| map04714 Thermogenesis | Gene.6262 | 104 | "probable NADH dehydrogenase [ubiquinone] flavoprotein 2, mitochondrial [Leptinotarsa decemlineata]" |
| map04714 Thermogenesis | Gene.6262 | 230 | "probable NADH dehydrogenase [ubiquinone] flavoprotein 2, mitochondrial [Leptinotarsa decemlineata]" |
| map04714 Thermogenesis | Gene.1073 | 88 | "PREDICTED: succinate dehydrogenase [ubiquinone] flavoprotein subunit, mitochondrial isoform X1 [Tribolium castaneum]" |
| map04714 Thermogenesis | Gene.1073 | 529 | "PREDICTED: succinate dehydrogenase [ubiquinone] flavoprotein subunit, mitochondrial isoform X1 [Tribolium castaneum]" |
| map04714 Thermogenesis | Gene.1073 | 546 | "PREDICTED: succinate dehydrogenase [ubiquinone] flavoprotein subunit, mitochondrial isoform X1 [Tribolium castaneum]" |
| map04714 Thermogenesis | Gene.1073 | 175 | "PREDICTED: succinate dehydrogenase [ubiquinone] flavoprotein subunit, mitochondrial isoform X1 [Tribolium castaneum]" |
| map04714 Thermogenesis | Gene.1073 | 632 | "PREDICTED: succinate dehydrogenase [ubiquinone] flavoprotein subunit, mitochondrial isoform X1 [Tribolium castaneum]" |
| map04714 Thermogenesis | Gene.1073 | 543 | "PREDICTED: succinate dehydrogenase [ubiquinone] flavoprotein subunit, mitochondrial isoform X1 [Tribolium castaneum]" |
| map04714 Thermogenesis | Gene.1073 | 331 | "PREDICTED: succinate dehydrogenase [ubiquinone] flavoprotein subunit, mitochondrial isoform X1 [Tribolium castaneum]" |
| map04714 Thermogenesis | Gene.919 | 149 | ribosomal protein S6 [Chrysomela tremula] |
| map04714 Thermogenesis | Gene.919 | 159 | ribosomal protein S6 [Chrysomela tremula] |
| map04714 Thermogenesis | Gene.919 | 165 | ribosomal protein S6 [Chrysomela tremula] |
| map04714 Thermogenesis | Gene.919 | 51 | ribosomal protein S6 [Chrysomela tremula] |
| map04714 Thermogenesis | Gene.919 | 58 | ribosomal protein S6 [Chrysomela tremula] |
| map04714 Thermogenesis | Gene.919 | 211 | ribosomal protein S6 [Chrysomela tremula] |
| map04714 Thermogenesis | Gene.919 | 14 | ribosomal protein S6 [Chrysomela tremula] |
| map04714 Thermogenesis | Gene.919 | 23 | ribosomal protein S6 [Chrysomela tremula] |
| map04714 Thermogenesis | Gene.1912 | 120 | PREDICTED: NADH dehydrogenase [ubiquinone] 1 beta subcomplex subunit 9 [Aethina tumida] |
| map04714 Thermogenesis | Gene.1912 | 51 | PREDICTED: NADH dehydrogenase [ubiquinone] 1 beta subcomplex subunit 9 [Aethina tumida] |
| map04714 Thermogenesis | Gene.8672 | 302 | "carnitine O-palmitoyltransferase 2, mitochondrial-like [Leptinotarsa decemlineata]" |
| map04714 Thermogenesis | Gene.580 | 231 | "PREDICTED: cytochrome b-c1 complex subunit 2, mitochondrial [Tribolium castaneum]" |
| map04714 Thermogenesis | Gene.580 | 248 | "PREDICTED: cytochrome b-c1 complex subunit 2, mitochondrial [Tribolium castaneum]" |
| map04714 Thermogenesis | Gene.6169 | 53 | cytochrome b-c1 complex subunit 7-like [Anoplophora glabripennis] |
| map04714 Thermogenesis | Gene.6169 | 77 | cytochrome b-c1 complex subunit 7-like [Anoplophora glabripennis] |
| map04714 Thermogenesis | Gene.3481 | 53 | NADH dehydrogenase [ubiquinone] 1 alpha subcomplex subunit 8 [Asbolus verrucosus] |
| map04714 Thermogenesis | Gene.2017 | 103 | "ATP synthase subunit O, mitochondrial [Asbolus verrucosus]" |
| map04714 Thermogenesis | Gene.2017 | 182 | "ATP synthase subunit O, mitochondrial [Asbolus verrucosus]" |
| map04714 Thermogenesis | Gene.2017 | 91 | "ATP synthase subunit O, mitochondrial [Asbolus verrucosus]" |
| map04714 Thermogenesis | Gene.2017 | 121 | "ATP synthase subunit O, mitochondrial [Asbolus verrucosus]" |
| map04714 Thermogenesis | Gene.2017 | 184 | "ATP synthase subunit O, mitochondrial [Asbolus verrucosus]" |
| map04714 Thermogenesis | Gene.2522 | 46 | "cytochrome c oxidase subunit 5A, mitochondrial [Anoplophora glabripennis]" |
| map04714 Thermogenesis | Gene.2522 | 101 | "cytochrome c oxidase subunit 5A, mitochondrial [Anoplophora glabripennis]" |
| map04714 Thermogenesis | Gene.2522 | 51 | "cytochrome c oxidase subunit 5A, mitochondrial [Anoplophora glabripennis]" |
| map04714 Thermogenesis | Gene.3447 | 163 | PREDICTED: congested-like trachea protein [Tribolium castaneum] |
| map04714 Thermogenesis | Gene.3447 | 72 | PREDICTED: congested-like trachea protein [Tribolium castaneum] |
| map04714 Thermogenesis | Gene.3447 | 167 | PREDICTED: congested-like trachea protein [Tribolium castaneum] |
| map04714 Thermogenesis | Gene.3447 | 258 | PREDICTED: congested-like trachea protein [Tribolium castaneum] |
| map04714 Thermogenesis | Gene.5709 | 26 | cAMP-dependent protein kinase catalytic subunit [Agrilus planipennis] |
| map04714 Thermogenesis | Gene.5709 | 257 | cAMP-dependent protein kinase catalytic subunit [Agrilus planipennis] |
| map04714 Thermogenesis | Gene.5767 | 89 | NADH dehydrogenase [ubiquinone] iron-sulfur protein 5 [Anoplophora glabripennis] |
| map04714 Thermogenesis | Gene.8516 | 373 | PREDICTED: NADH-ubiquinone oxidoreductase 49 kDa subunit [Tribolium castaneum] |
| map04714 Thermogenesis | Gene.8516 | 310 | PREDICTED: NADH-ubiquinone oxidoreductase 49 kDa subunit [Tribolium castaneum] |
| map04714 Thermogenesis | Gene.8516 | 368 | PREDICTED: NADH-ubiquinone oxidoreductase 49 kDa subunit [Tribolium castaneum] |
| map04714 Thermogenesis | Gene.8673 | 48 | "succinate dehydrogenase [ubiquinone] iron-sulfur subunit, mitochondrial, partial [Asbolus verrucosus]" |
| map04714 Thermogenesis | Gene.3795 | 166 | "NADH-ubiquinone oxidoreductase 75 kDa subunit, mitochondrial [Asbolus verrucosus]" |
| map04714 Thermogenesis | Gene.3795 | 172 | "NADH-ubiquinone oxidoreductase 75 kDa subunit, mitochondrial [Asbolus verrucosus]" |
| map04714 Thermogenesis | Gene.3795 | 691 | "NADH-ubiquinone oxidoreductase 75 kDa subunit, mitochondrial [Asbolus verrucosus]" |
| map04714 Thermogenesis | Gene.3795 | 626 | "NADH-ubiquinone oxidoreductase 75 kDa subunit, mitochondrial [Asbolus verrucosus]" |
| map04714 Thermogenesis | Gene.3795 | 307 | "NADH-ubiquinone oxidoreductase 75 kDa subunit, mitochondrial [Asbolus verrucosus]" |
| map04714 Thermogenesis | Gene.3795 | 541 | "NADH-ubiquinone oxidoreductase 75 kDa subunit, mitochondrial [Asbolus verrucosus]" |
| map04714 Thermogenesis | Gene.3795 | 622 | "NADH-ubiquinone oxidoreductase 75 kDa subunit, mitochondrial [Asbolus verrucosus]" |
| map04714 Thermogenesis | Gene.7134 | 95 | "PREDICTED: ATP synthase subunit d, mitochondrial [Tribolium castaneum]" |
| map04714 Thermogenesis | Gene.7134 | 105 | "PREDICTED: ATP synthase subunit d, mitochondrial [Tribolium castaneum]" |
| map04714 Thermogenesis | Gene.3063 | 178 | PREDICTED: uncharacterized protein LOC108560181 [Nicrophorus vespilloides] |
| map04714 Thermogenesis | Gene.4303 | 171 | PREDICTED: LOW QUALITY PROTEIN: long-chain-fatty-acid--CoA ligase 4 [Aethina tumida] |
| map04714 Thermogenesis | Gene.1846 | 205 | "ATP synthase subunit beta, mitochondrial [Tribolium castaneum]" |
| map04714 Thermogenesis | Gene.1846 | 128 | "ATP synthase subunit beta, mitochondrial [Tribolium castaneum]" |
| map04714 Thermogenesis | Gene.1846 | 497 | "ATP synthase subunit beta, mitochondrial [Tribolium castaneum]" |
| map04714 Thermogenesis | Gene.1846 | 263 | "ATP synthase subunit beta, mitochondrial [Tribolium castaneum]" |
| map04714 Thermogenesis | Gene.1846 | 268 | "ATP synthase subunit beta, mitochondrial [Tribolium castaneum]" |
| map04714 Thermogenesis | Gene.1846 | 202 | "ATP synthase subunit beta, mitochondrial [Tribolium castaneum]" |
| map04714 Thermogenesis | Gene.1846 | 430 | "ATP synthase subunit beta, mitochondrial [Tribolium castaneum]" |
| map04714 Thermogenesis | Gene.8771 | 54 | "PREDICTED: NADH dehydrogenase [ubiquinone] iron-sulfur protein 6, mitochondrial [Tribolium castaneum]" |
| map00910 Nitrogen metabolism | Gene.3721 | 212 | PREDICTED: glutamine synthetase-like [Aethina tumida] |
| map00910 Nitrogen metabolism | Gene.3721 | 39 | PREDICTED: glutamine synthetase-like [Aethina tumida] |
| map00910 Nitrogen metabolism | Gene.3721 | 208 | PREDICTED: glutamine synthetase-like [Aethina tumida] |
| map00910 Nitrogen metabolism | Gene.3721 | 227 | PREDICTED: glutamine synthetase-like [Aethina tumida] |
| map00910 Nitrogen metabolism | Gene.3721 | 195 | PREDICTED: glutamine synthetase-like [Aethina tumida] |
| map00910 Nitrogen metabolism | Gene.4531 | 280 | "PREDICTED: glutamate synthase 1 [NADH], chloroplastic isoform X2 [Tribolium castaneum]" |
| map00910 Nitrogen metabolism | Gene.2236 | 516 | "Glutamate dehydrogenase, mitochondrial-like Protein [Tribolium castaneum]" |
| map00910 Nitrogen metabolism | Gene.2236 | 412 | "Glutamate dehydrogenase, mitochondrial-like Protein [Tribolium castaneum]" |
| map00910 Nitrogen metabolism | Gene.2236 | 492 | "Glutamate dehydrogenase, mitochondrial-like Protein [Tribolium castaneum]" |
| map00910 Nitrogen metabolism | Gene.2236 | 457 | "Glutamate dehydrogenase, mitochondrial-like Protein [Tribolium castaneum]" |
| map00910 Nitrogen metabolism | Gene.2236 | 87 | "Glutamate dehydrogenase, mitochondrial-like Protein [Tribolium castaneum]" |
| map00910 Nitrogen metabolism | Gene.2236 | 186 | "Glutamate dehydrogenase, mitochondrial-like Protein [Tribolium castaneum]" |
| map00910 Nitrogen metabolism | Gene.2236 | 500 | "Glutamate dehydrogenase, mitochondrial-like Protein [Tribolium castaneum]" |
| map00910 Nitrogen metabolism | Gene.2236 | 372 | "Glutamate dehydrogenase, mitochondrial-like Protein [Tribolium castaneum]" |
| map00910 Nitrogen metabolism | Gene.2236 | 477 | "Glutamate dehydrogenase, mitochondrial-like Protein [Tribolium castaneum]" |
| map00910 Nitrogen metabolism | Gene.2236 | 195 | "Glutamate dehydrogenase, mitochondrial-like Protein [Tribolium castaneum]" |
| map00910 Nitrogen metabolism | Gene.2236 | 82 | "Glutamate dehydrogenase, mitochondrial-like Protein [Tribolium castaneum]" |
| map00920 Sulfur metabolism | Gene.6380 | 43 | "PREDICTED: 3'(2'),5'-bisphosphate nucleotidase 1 [Nicrophorus vespilloides]" |
| map00920 Sulfur metabolism | Gene.7794 | 95 | "PREDICTED: sulfide:quinone oxidoreductase, mitochondrial [Tribolium castaneum]" |
| map04940 Type I diabetes mellitus | Gene.2149 | 359 | "heat shock protein, partial [Cryptolaemus montrouzieri]" |
| map04940 Type I diabetes mellitus | Gene.2149 | 387 | "heat shock protein, partial [Cryptolaemus montrouzieri]" |
| map04940 Type I diabetes mellitus | Gene.2149 | 156 | "heat shock protein, partial [Cryptolaemus montrouzieri]" |
| map04940 Type I diabetes mellitus | Gene.2149 | 75 | "heat shock protein, partial [Cryptolaemus montrouzieri]" |
| map04940 Type I diabetes mellitus | Gene.2149 | 108 | "heat shock protein, partial [Cryptolaemus montrouzieri]" |
| map04940 Type I diabetes mellitus | Gene.2149 | 202 | "heat shock protein, partial [Cryptolaemus montrouzieri]" |
| map04940 Type I diabetes mellitus | Gene.2149 | 417 | "heat shock protein, partial [Cryptolaemus montrouzieri]" |
| map04940 Type I diabetes mellitus | Gene.2149 | 469 | "heat shock protein, partial [Cryptolaemus montrouzieri]" |
| map04940 Type I diabetes mellitus | Gene.2149 | 72 | "heat shock protein, partial [Cryptolaemus montrouzieri]" |
| map04940 Type I diabetes mellitus | Gene.2149 | 89 | "heat shock protein, partial [Cryptolaemus montrouzieri]" |
| map04940 Type I diabetes mellitus | Gene.2149 | 125 | "heat shock protein, partial [Cryptolaemus montrouzieri]" |
| map04940 Type I diabetes mellitus | Gene.2149 | 133 | "heat shock protein, partial [Cryptolaemus montrouzieri]" |
| map05152 Tuberculosis | Gene.8756 | 84 | uncharacterized protein Dvir_GJ16722 [Drosophila virilis] |
| map05152 Tuberculosis | Gene.8756 | 10 | uncharacterized protein Dvir_GJ16722 [Drosophila virilis] |
| map05152 Tuberculosis | Gene.2318 | 109 | PREDICTED: LOW QUALITY PROTEIN: V-type proton ATPase 116 kDa subunit a-like [Aethina tumida] |
| map05152 Tuberculosis | Gene.2318 | 50 | PREDICTED: LOW QUALITY PROTEIN: V-type proton ATPase 116 kDa subunit a-like [Aethina tumida] |
| map05152 Tuberculosis | Gene.2318 | 522 | PREDICTED: LOW QUALITY PROTEIN: V-type proton ATPase 116 kDa subunit a-like [Aethina tumida] |
| map05152 Tuberculosis | Gene.2318 | 76 | PREDICTED: LOW QUALITY PROTEIN: V-type proton ATPase 116 kDa subunit a-like [Aethina tumida] |
| map05152 Tuberculosis | Gene.2318 | 266 | PREDICTED: LOW QUALITY PROTEIN: V-type proton ATPase 116 kDa subunit a-like [Aethina tumida] |
| map05152 Tuberculosis | Gene.2348 | 116 | ras-related protein Rab-5B isoform X1 [Leptinotarsa decemlineata] |
| map05152 Tuberculosis | Gene.4190 | 409 | V-type proton ATPase subunit H [Asbolus verrucosus] |
| map05152 Tuberculosis | Gene.246 | 39 | PREDICTED: V-type proton ATPase subunit d [Tribolium castaneum] |
| map05152 Tuberculosis | Gene.5815 | 276 | PREDICTED: uncharacterized protein LOC663029 [Tribolium castaneum] |
| map05152 Tuberculosis | Gene.5815 | 279 | PREDICTED: uncharacterized protein LOC663029 [Tribolium castaneum] |
| map05152 Tuberculosis | Gene.5815 | 410 | PREDICTED: uncharacterized protein LOC663029 [Tribolium castaneum] |
| map05152 Tuberculosis | Gene.5803 | 20 | calcium-binding protein E63-1 [Anoplophora glabripennis] |
| map05152 Tuberculosis | Gene.1579 | 310 | putative gut cathepsin D-like aspartic protease [Callosobruchus maculatus] |
| map05152 Tuberculosis | Gene.1579 | 118 | putative gut cathepsin D-like aspartic protease [Callosobruchus maculatus] |
| map05152 Tuberculosis | Gene.272 | 395 | PREDICTED: aspartic proteinase A2 [Tribolium castaneum] |
| map05152 Tuberculosis | Gene.272 | 344 | PREDICTED: aspartic proteinase A2 [Tribolium castaneum] |
| map05152 Tuberculosis | Gene.272 | 239 | PREDICTED: aspartic proteinase A2 [Tribolium castaneum] |
| map05152 Tuberculosis | Gene.298 | 116 | "Csa-calmodulin 3, partial [Cupiennius salei]" |
| map05152 Tuberculosis | Gene.298 | 95 | "Csa-calmodulin 3, partial [Cupiennius salei]" |
| map05152 Tuberculosis | Gene.298 | 31 | "Csa-calmodulin 3, partial [Cupiennius salei]" |
| map05152 Tuberculosis | Gene.298 | 78 | "Csa-calmodulin 3, partial [Cupiennius salei]" |
| map05152 Tuberculosis | Gene.664 | 50 | PREDICTED: V-type proton ATPase 116 kDa subunit a isoform 1 isoform X3 [Tribolium castaneum] |
| map05152 Tuberculosis | Gene.316 | 127 | heat shock 70 kDa protein cognate 5-like protein [Epicauta chinensis] |
| map05152 Tuberculosis | Gene.316 | 612 | heat shock 70 kDa protein cognate 5-like protein [Epicauta chinensis] |
| map05152 Tuberculosis | Gene.316 | 412 | heat shock 70 kDa protein cognate 5-like protein [Epicauta chinensis] |
| map05152 Tuberculosis | Gene.316 | 241 | heat shock 70 kDa protein cognate 5-like protein [Epicauta chinensis] |
| map05152 Tuberculosis | Gene.316 | 307 | heat shock 70 kDa protein cognate 5-like protein [Epicauta chinensis] |
| map05152 Tuberculosis | Gene.316 | 607 | heat shock 70 kDa protein cognate 5-like protein [Epicauta chinensis] |
| map05152 Tuberculosis | Gene.316 | 632 | heat shock 70 kDa protein cognate 5-like protein [Epicauta chinensis] |
| map05152 Tuberculosis | Gene.316 | 617 | heat shock 70 kDa protein cognate 5-like protein [Epicauta chinensis] |
| map05152 Tuberculosis | Gene.316 | 677 | heat shock 70 kDa protein cognate 5-like protein [Epicauta chinensis] |
| map05152 Tuberculosis | Gene.316 | 352 | heat shock 70 kDa protein cognate 5-like protein [Epicauta chinensis] |
| map05152 Tuberculosis | Gene.316 | 144 | heat shock 70 kDa protein cognate 5-like protein [Epicauta chinensis] |
| map05152 Tuberculosis | Gene.316 | 378 | heat shock 70 kDa protein cognate 5-like protein [Epicauta chinensis] |
| map05152 Tuberculosis | Gene.316 | 82 | heat shock 70 kDa protein cognate 5-like protein [Epicauta chinensis] |
| map05152 Tuberculosis | Gene.316 | 149 | heat shock 70 kDa protein cognate 5-like protein [Epicauta chinensis] |
| map05152 Tuberculosis | Gene.316 | 182 | heat shock 70 kDa protein cognate 5-like protein [Epicauta chinensis] |
| map05152 Tuberculosis | Gene.316 | 367 | heat shock 70 kDa protein cognate 5-like protein [Epicauta chinensis] |
| map05152 Tuberculosis | Gene.316 | 619 | heat shock 70 kDa protein cognate 5-like protein [Epicauta chinensis] |
| map05152 Tuberculosis | Gene.316 | 140 | heat shock 70 kDa protein cognate 5-like protein [Epicauta chinensis] |
| map05152 Tuberculosis | Gene.2149 | 359 | "heat shock protein, partial [Cryptolaemus montrouzieri]" |
| map05152 Tuberculosis | Gene.2149 | 387 | "heat shock protein, partial [Cryptolaemus montrouzieri]" |
| map05152 Tuberculosis | Gene.2149 | 156 | "heat shock protein, partial [Cryptolaemus montrouzieri]" |
| map05152 Tuberculosis | Gene.2149 | 75 | "heat shock protein, partial [Cryptolaemus montrouzieri]" |
| map05152 Tuberculosis | Gene.2149 | 108 | "heat shock protein, partial [Cryptolaemus montrouzieri]" |
| map05152 Tuberculosis | Gene.2149 | 202 | "heat shock protein, partial [Cryptolaemus montrouzieri]" |
| map05152 Tuberculosis | Gene.2149 | 417 | "heat shock protein, partial [Cryptolaemus montrouzieri]" |
| map05152 Tuberculosis | Gene.2149 | 469 | "heat shock protein, partial [Cryptolaemus montrouzieri]" |
| map05152 Tuberculosis | Gene.2149 | 72 | "heat shock protein, partial [Cryptolaemus montrouzieri]" |
| map05152 Tuberculosis | Gene.2149 | 89 | "heat shock protein, partial [Cryptolaemus montrouzieri]" |
| map05152 Tuberculosis | Gene.2149 | 125 | "heat shock protein, partial [Cryptolaemus montrouzieri]" |
| map05152 Tuberculosis | Gene.2149 | 133 | "heat shock protein, partial [Cryptolaemus montrouzieri]" |
| map04211 Longevity regulating pathway | Gene.5709 | 26 | cAMP-dependent protein kinase catalytic subunit [Agrilus planipennis] |
| map04211 Longevity regulating pathway | Gene.5709 | 257 | cAMP-dependent protein kinase catalytic subunit [Agrilus planipennis] |
| map04211 Longevity regulating pathway | Gene.5965 | 63 | "PREDICTED: superoxide dismutase [Mn] 1, mitochondrial-like [Aethina tumida]" |
| map04211 Longevity regulating pathway | Gene.5965 | 68 | "PREDICTED: superoxide dismutase [Mn] 1, mitochondrial-like [Aethina tumida]" |
| map04211 Longevity regulating pathway | Gene.5542 | 28 | catalase [Onthophagus taurus] |
| map04211 Longevity regulating pathway | Gene.5542 | 179 | catalase [Onthophagus taurus] |
| map04211 Longevity regulating pathway | Gene.5542 | 325 | catalase [Onthophagus taurus] |
| map04211 Longevity regulating pathway | Gene.5542 | 247 | catalase [Onthophagus taurus] |
| map04211 Longevity regulating pathway | Gene.5542 | 438 | catalase [Onthophagus taurus] |
| map04211 Longevity regulating pathway | Gene.5542 | 48 | catalase [Onthophagus taurus] |
| map00120 Primary bile acid biosynthesis | Gene.2197 | 664 | PREDICTED: peroxisomal multifunctional enzyme type 2 isoform X1 [Tribolium castaneum] |
| map00120 Primary bile acid biosynthesis | Gene.2197 | 138 | PREDICTED: peroxisomal multifunctional enzyme type 2 isoform X1 [Tribolium castaneum] |
| map00120 Primary bile acid biosynthesis | Gene.2197 | 275 | PREDICTED: peroxisomal multifunctional enzyme type 2 isoform X1 [Tribolium castaneum] |
| map00120 Primary bile acid biosynthesis | Gene.2197 | 638 | PREDICTED: peroxisomal multifunctional enzyme type 2 isoform X1 [Tribolium castaneum] |
| map00120 Primary bile acid biosynthesis | Gene.2197 | 647 | PREDICTED: peroxisomal multifunctional enzyme type 2 isoform X1 [Tribolium castaneum] |
| map00120 Primary bile acid biosynthesis | Gene.7551 | 170 | PREDICTED: non-specific lipid-transfer protein [Aethina tumida] |
| map00120 Primary bile acid biosynthesis | Gene.7551 | 119 | PREDICTED: non-specific lipid-transfer protein [Aethina tumida] |
| map00120 Primary bile acid biosynthesis | Gene.7551 | 269 | PREDICTED: non-specific lipid-transfer protein [Aethina tumida] |
| map04960 Aldosterone-regulated sodium reabsorption | Gene.2599 | 765 | PREDICTED: sodium/potassium-transporting ATPase subunit alpha isoform X3 [Tribolium castaneum] |
| map04622 RIG-I-like receptor signaling pathway | Gene.1088 | 163 | "ATP-dependent RNA helicase belle, partial [Asbolus verrucosus]" |
| map04622 RIG-I-like receptor signaling pathway | Gene.5465 | 67 | PREDICTED: peptidyl-prolyl cis-trans isomerase NIMA-interacting 1 [Tribolium castaneum] |
| map00640 Propanoate metabolism | Gene.3489 | 2124 | PREDICTED: acetyl-CoA carboxylase isoform X1 [Tribolium castaneum] |
| map00640 Propanoate metabolism | Gene.3489 | 2067 | PREDICTED: acetyl-CoA carboxylase isoform X1 [Tribolium castaneum] |
| map00640 Propanoate metabolism | Gene.3489 | 387 | PREDICTED: acetyl-CoA carboxylase isoform X1 [Tribolium castaneum] |
| map00640 Propanoate metabolism | Gene.3489 | 1848 | PREDICTED: acetyl-CoA carboxylase isoform X1 [Tribolium castaneum] |
| map00640 Propanoate metabolism | Gene.3489 | 2305 | PREDICTED: acetyl-CoA carboxylase isoform X1 [Tribolium castaneum] |
| map00640 Propanoate metabolism | Gene.3489 | 341 | PREDICTED: acetyl-CoA carboxylase isoform X1 [Tribolium castaneum] |
| map00640 Propanoate metabolism | Gene.3489 | 167 | PREDICTED: acetyl-CoA carboxylase isoform X1 [Tribolium castaneum] |
| map00640 Propanoate metabolism | Gene.3489 | 1964 | PREDICTED: acetyl-CoA carboxylase isoform X1 [Tribolium castaneum] |
| map00640 Propanoate metabolism | Gene.3489 | 1645 | PREDICTED: acetyl-CoA carboxylase isoform X1 [Tribolium castaneum] |
| map00640 Propanoate metabolism | Gene.3489 | 1389 | PREDICTED: acetyl-CoA carboxylase isoform X1 [Tribolium castaneum] |
| map00640 Propanoate metabolism | Gene.3489 | 2131 | PREDICTED: acetyl-CoA carboxylase isoform X1 [Tribolium castaneum] |
| map00640 Propanoate metabolism | Gene.3489 | 179 | PREDICTED: acetyl-CoA carboxylase isoform X1 [Tribolium castaneum] |
| map00640 Propanoate metabolism | Gene.3489 | 383 | PREDICTED: acetyl-CoA carboxylase isoform X1 [Tribolium castaneum] |
| map00640 Propanoate metabolism | Gene.3489 | 1820 | PREDICTED: acetyl-CoA carboxylase isoform X1 [Tribolium castaneum] |
| map00640 Propanoate metabolism | Gene.3489 | 1264 | PREDICTED: acetyl-CoA carboxylase isoform X1 [Tribolium castaneum] |
| map00640 Propanoate metabolism | Gene.3489 | 1412 | PREDICTED: acetyl-CoA carboxylase isoform X1 [Tribolium castaneum] |
| map00640 Propanoate metabolism | Gene.3489 | 2235 | PREDICTED: acetyl-CoA carboxylase isoform X1 [Tribolium castaneum] |
| map00640 Propanoate metabolism | Gene.3489 | 746 | PREDICTED: acetyl-CoA carboxylase isoform X1 [Tribolium castaneum] |
| map00640 Propanoate metabolism | Gene.3489 | 2342 | PREDICTED: acetyl-CoA carboxylase isoform X1 [Tribolium castaneum] |
| map00640 Propanoate metabolism | Gene.3489 | 1379 | PREDICTED: acetyl-CoA carboxylase isoform X1 [Tribolium castaneum] |
| map00640 Propanoate metabolism | Gene.3489 | 1369 | PREDICTED: acetyl-CoA carboxylase isoform X1 [Tribolium castaneum] |
| map00640 Propanoate metabolism | Gene.3489 | 2245 | PREDICTED: acetyl-CoA carboxylase isoform X1 [Tribolium castaneum] |
| map00640 Propanoate metabolism | Gene.2144 | 304 | "PREDICTED: succinyl-CoA ligase subunit alpha, mitochondrial [Tribolium castaneum]" |
| map00640 Propanoate metabolism | Gene.2144 | 50 | "PREDICTED: succinyl-CoA ligase subunit alpha, mitochondrial [Tribolium castaneum]" |
| map00640 Propanoate metabolism | Gene.2144 | 77 | "PREDICTED: succinyl-CoA ligase subunit alpha, mitochondrial [Tribolium castaneum]" |
| map00640 Propanoate metabolism | Gene.2144 | 57 | "PREDICTED: succinyl-CoA ligase subunit alpha, mitochondrial [Tribolium castaneum]" |
| map00640 Propanoate metabolism | Gene.2144 | 89 | "PREDICTED: succinyl-CoA ligase subunit alpha, mitochondrial [Tribolium castaneum]" |
| map00640 Propanoate metabolism | Gene.1914 | 68 | "3-hydroxyisobutyryl-CoA hydrolase, mitochondrial isoform X1 [Anoplophora glabripennis]" |
| map00640 Propanoate metabolism | Gene.970 | 56 | "short-chain specific acyl-CoA dehydrogenase, mitochondrial-like [Leptinotarsa decemlineata]" |
| map00640 Propanoate metabolism | Gene.970 | 193 | "short-chain specific acyl-CoA dehydrogenase, mitochondrial-like [Leptinotarsa decemlineata]" |
| map00640 Propanoate metabolism | Gene.7576 | 56 | ATP-grasp 2 and/or Ligase CoA domain containing protein [Asbolus verrucosus] |
| map00640 Propanoate metabolism | Gene.7576 | 366 | ATP-grasp 2 and/or Ligase CoA domain containing protein [Asbolus verrucosus] |
| map00640 Propanoate metabolism | Gene.7576 | 320 | ATP-grasp 2 and/or Ligase CoA domain containing protein [Asbolus verrucosus] |
| map00640 Propanoate metabolism | Gene.7576 | 83 | ATP-grasp 2 and/or Ligase CoA domain containing protein [Asbolus verrucosus] |
| map00640 Propanoate metabolism | Gene.7576 | 329 | ATP-grasp 2 and/or Ligase CoA domain containing protein [Asbolus verrucosus] |
| map00640 Propanoate metabolism | Gene.7576 | 249 | ATP-grasp 2 and/or Ligase CoA domain containing protein [Asbolus verrucosus] |
| map00640 Propanoate metabolism | Gene.7576 | 202 | ATP-grasp 2 and/or Ligase CoA domain containing protein [Asbolus verrucosus] |
| map00640 Propanoate metabolism | Gene.7576 | 336 | ATP-grasp 2 and/or Ligase CoA domain containing protein [Asbolus verrucosus] |
| map00640 Propanoate metabolism | Gene.7388 | 327 | "short-chain specific acyl-CoA dehydrogenase, mitochondrial [Anoplophora glabripennis]" |
| map00640 Propanoate metabolism | Gene.7388 | 294 | "short-chain specific acyl-CoA dehydrogenase, mitochondrial [Anoplophora glabripennis]" |
| map00640 Propanoate metabolism | Gene.7388 | 212 | "short-chain specific acyl-CoA dehydrogenase, mitochondrial [Anoplophora glabripennis]" |
| map00640 Propanoate metabolism | Gene.7388 | 330 | "short-chain specific acyl-CoA dehydrogenase, mitochondrial [Anoplophora glabripennis]" |
| map00640 Propanoate metabolism | Gene.7388 | 298 | "short-chain specific acyl-CoA dehydrogenase, mitochondrial [Anoplophora glabripennis]" |
| map00640 Propanoate metabolism | Gene.7388 | 335 | "short-chain specific acyl-CoA dehydrogenase, mitochondrial [Anoplophora glabripennis]" |
| map00640 Propanoate metabolism | Gene.7388 | 51 | "short-chain specific acyl-CoA dehydrogenase, mitochondrial [Anoplophora glabripennis]" |
| map00640 Propanoate metabolism | Gene.7388 | 218 | "short-chain specific acyl-CoA dehydrogenase, mitochondrial [Anoplophora glabripennis]" |
| map00640 Propanoate metabolism | Gene.922 | 243 | "acetyl-CoA acetyltransferase, mitochondrial, partial [Asbolus verrucosus]" |
| map00640 Propanoate metabolism | Gene.922 | 252 | "acetyl-CoA acetyltransferase, mitochondrial, partial [Asbolus verrucosus]" |
| map00640 Propanoate metabolism | Gene.922 | 214 | "acetyl-CoA acetyltransferase, mitochondrial, partial [Asbolus verrucosus]" |
| map00640 Propanoate metabolism | Gene.922 | 293 | "acetyl-CoA acetyltransferase, mitochondrial, partial [Asbolus verrucosus]" |
| map00640 Propanoate metabolism | Gene.922 | 249 | "acetyl-CoA acetyltransferase, mitochondrial, partial [Asbolus verrucosus]" |
| map00640 Propanoate metabolism | Gene.3075 | 162 | "dihydrolipoyl dehydrogenase, mitochondrial [Anoplophora glabripennis]" |
| map00640 Propanoate metabolism | Gene.3075 | 118 | "dihydrolipoyl dehydrogenase, mitochondrial [Anoplophora glabripennis]" |
| map00640 Propanoate metabolism | Gene.3075 | 259 | "dihydrolipoyl dehydrogenase, mitochondrial [Anoplophora glabripennis]" |
| map00640 Propanoate metabolism | Gene.3075 | 273 | "dihydrolipoyl dehydrogenase, mitochondrial [Anoplophora glabripennis]" |
| map00640 Propanoate metabolism | Gene.3075 | 139 | "dihydrolipoyl dehydrogenase, mitochondrial [Anoplophora glabripennis]" |
| map00640 Propanoate metabolism | Gene.1305 | 104 | hypothetical protein AMK59_6936 [Oryctes borbonicus] |
| map00640 Propanoate metabolism | Gene.1305 | 162 | hypothetical protein AMK59_6936 [Oryctes borbonicus] |
| map00640 Propanoate metabolism | Gene.1305 | 214 | hypothetical protein AMK59_6936 [Oryctes borbonicus] |
| map00640 Propanoate metabolism | Gene.4581 | 408 | "PREDICTED: trifunctional enzyme subunit alpha, mitochondrial [Tribolium castaneum]" |
| map00640 Propanoate metabolism | Gene.4581 | 401 | "PREDICTED: trifunctional enzyme subunit alpha, mitochondrial [Tribolium castaneum]" |
| map00640 Propanoate metabolism | Gene.4581 | 280 | "PREDICTED: trifunctional enzyme subunit alpha, mitochondrial [Tribolium castaneum]" |
| map00640 Propanoate metabolism | Gene.4581 | 118 | "PREDICTED: trifunctional enzyme subunit alpha, mitochondrial [Tribolium castaneum]" |
| map00640 Propanoate metabolism | Gene.4581 | 385 | "PREDICTED: trifunctional enzyme subunit alpha, mitochondrial [Tribolium castaneum]" |
| map00640 Propanoate metabolism | Gene.4581 | 199 | "PREDICTED: trifunctional enzyme subunit alpha, mitochondrial [Tribolium castaneum]" |
| map00640 Propanoate metabolism | Gene.4581 | 249 | "PREDICTED: trifunctional enzyme subunit alpha, mitochondrial [Tribolium castaneum]" |
| map00640 Propanoate metabolism | Gene.4581 | 126 | "PREDICTED: trifunctional enzyme subunit alpha, mitochondrial [Tribolium castaneum]" |
| map00640 Propanoate metabolism | Gene.4404 | 26 | peroxisomal acyl-coenzyme A oxidase 3 isoform X1 [Anoplophora glabripennis] |
| map00640 Propanoate metabolism | Gene.2650 | 350 | "PREDICTED: succinyl-CoA ligase [ADP-forming] subunit beta, mitochondrial [Tribolium castaneum]" |
| map00640 Propanoate metabolism | Gene.2650 | 237 | "PREDICTED: succinyl-CoA ligase [ADP-forming] subunit beta, mitochondrial [Tribolium castaneum]" |
| map00640 Propanoate metabolism | Gene.2650 | 221 | "PREDICTED: succinyl-CoA ligase [ADP-forming] subunit beta, mitochondrial [Tribolium castaneum]" |
| map00640 Propanoate metabolism | Gene.2650 | 389 | "PREDICTED: succinyl-CoA ligase [ADP-forming] subunit beta, mitochondrial [Tribolium castaneum]" |
| map00640 Propanoate metabolism | Gene.2650 | 400 | "PREDICTED: succinyl-CoA ligase [ADP-forming] subunit beta, mitochondrial [Tribolium castaneum]" |
| map00640 Propanoate metabolism | Gene.2650 | 288 | "PREDICTED: succinyl-CoA ligase [ADP-forming] subunit beta, mitochondrial [Tribolium castaneum]" |
| map00640 Propanoate metabolism | Gene.2650 | 214 | "PREDICTED: succinyl-CoA ligase [ADP-forming] subunit beta, mitochondrial [Tribolium castaneum]" |
| map00640 Propanoate metabolism | Gene.2650 | 405 | "PREDICTED: succinyl-CoA ligase [ADP-forming] subunit beta, mitochondrial [Tribolium castaneum]" |
| map00640 Propanoate metabolism | Gene.2650 | 62 | "PREDICTED: succinyl-CoA ligase [ADP-forming] subunit beta, mitochondrial [Tribolium castaneum]" |
| map00640 Propanoate metabolism | Gene.2650 | 82 | "PREDICTED: succinyl-CoA ligase [ADP-forming] subunit beta, mitochondrial [Tribolium castaneum]" |
| map00640 Propanoate metabolism | Gene.2650 | 223 | "PREDICTED: succinyl-CoA ligase [ADP-forming] subunit beta, mitochondrial [Tribolium castaneum]" |
| map00640 Propanoate metabolism | Gene.3875 | 350 | "PREDICTED: probable methylmalonate-semialdehyde dehydrogenase [acylating], mitochondrial [Aethina tumida]" |
| map00640 Propanoate metabolism | Gene.3875 | 241 | "PREDICTED: probable methylmalonate-semialdehyde dehydrogenase [acylating], mitochondrial [Aethina tumida]" |
| map00640 Propanoate metabolism | Gene.3875 | 253 | "PREDICTED: probable methylmalonate-semialdehyde dehydrogenase [acylating], mitochondrial [Aethina tumida]" |
| map00830 Retinol metabolism | Gene.529 | 130 | dehydrogenase/reductase SDR family member 4-like [Anoplophora glabripennis] |
| map00830 Retinol metabolism | Gene.529 | 294 | dehydrogenase/reductase SDR family member 4-like [Anoplophora glabripennis] |
| map00830 Retinol metabolism | Gene.529 | 208 | dehydrogenase/reductase SDR family member 4-like [Anoplophora glabripennis] |
| map00830 Retinol metabolism | Gene.8787 | 296 | UDPGT domain containing protein [Asbolus verrucosus] |
| map00830 Retinol metabolism | Gene.8787 | 208 | UDPGT domain containing protein [Asbolus verrucosus] |
| map00830 Retinol metabolism | Gene.8787 | 442 | UDPGT domain containing protein [Asbolus verrucosus] |
| map00830 Retinol metabolism | Gene.3731 | 221 | PREDICTED: UDP-glucuronosyltransferase 2B2 [Tribolium castaneum] |
| map00830 Retinol metabolism | Gene.3734 | 395 | PREDICTED: UDP-glucuronosyltransferase 2B7 isoform X1 [Tribolium castaneum] |
| map00830 Retinol metabolism | Gene.3734 | 305 | PREDICTED: UDP-glucuronosyltransferase 2B7 isoform X1 [Tribolium castaneum] |
| map00830 Retinol metabolism | Gene.3734 | 400 | PREDICTED: UDP-glucuronosyltransferase 2B7 isoform X1 [Tribolium castaneum] |
| map00830 Retinol metabolism | Gene.3734 | 378 | PREDICTED: UDP-glucuronosyltransferase 2B7 isoform X1 [Tribolium castaneum] |
| map00830 Retinol metabolism | Gene.3734 | 406 | PREDICTED: UDP-glucuronosyltransferase 2B7 isoform X1 [Tribolium castaneum] |
| map00830 Retinol metabolism | Gene.2052 | 422 | "UDP-glucuronosyltransferase 2C1-like, partial [Asbolus verrucosus]" |
| map00830 Retinol metabolism | Gene.4396 | 69 | PREDICTED: 2-hydroxyacylsphingosine 1-beta-galactosyltransferase-like [Tribolium castaneum] |
| map00830 Retinol metabolism | Gene.4396 | 317 | PREDICTED: 2-hydroxyacylsphingosine 1-beta-galactosyltransferase-like [Tribolium castaneum] |
| map00830 Retinol metabolism | Gene.4396 | 49 | PREDICTED: 2-hydroxyacylsphingosine 1-beta-galactosyltransferase-like [Tribolium castaneum] |
| map00830 Retinol metabolism | Gene.4396 | 259 | PREDICTED: 2-hydroxyacylsphingosine 1-beta-galactosyltransferase-like [Tribolium castaneum] |
| map00830 Retinol metabolism | Gene.2140 | 401 | PREDICTED: UDP-glucuronosyltransferase 2B10-like [Tribolium castaneum] |
| map00830 Retinol metabolism | Gene.2140 | 80 | PREDICTED: UDP-glucuronosyltransferase 2B10-like [Tribolium castaneum] |
| map00830 Retinol metabolism | Gene.2140 | 425 | PREDICTED: UDP-glucuronosyltransferase 2B10-like [Tribolium castaneum] |
| map00830 Retinol metabolism | Gene.2140 | 410 | PREDICTED: UDP-glucuronosyltransferase 2B10-like [Tribolium castaneum] |
| map00830 Retinol metabolism | Gene.2176 | 48 | UDP-glucuronosyltransferase 1-9-like isoform X2 [Leptinotarsa decemlineata] |
| map00830 Retinol metabolism | Gene.1050 | 121 | PREDICTED: 2-hydroxyacylsphingosine 1-beta-galactosyltransferase-like [Tribolium castaneum] |
| map00830 Retinol metabolism | Gene.2175 | 192 | PREDICTED: 2-hydroxyacylsphingosine 1-beta-galactosyltransferase-like [Tribolium castaneum] |
| map00830 Retinol metabolism | Gene.7878 | 6 | alcohol dehydrogenase class-3 [Leptinotarsa decemlineata] |
| map00830 Retinol metabolism | Gene.7878 | 340 | alcohol dehydrogenase class-3 [Leptinotarsa decemlineata] |
| map00830 Retinol metabolism | Gene.2178 | 47 | UDPGT and/or Glyco tran 28 C domain containing protein [Asbolus verrucosus] |
| map05206 MicroRNAs in cancer | Gene.4022 | 443 | protein singed [Anoplophora glabripennis] |
| map05206 MicroRNAs in cancer | Gene.1392 | 166 | hypothetical protein WN55_01867 [Dufourea novaeangliae] |
| map05206 MicroRNAs in cancer | Gene.1392 | 43 | hypothetical protein WN55_01867 [Dufourea novaeangliae] |
| map05206 MicroRNAs in cancer | Gene.1392 | 117 | hypothetical protein WN55_01867 [Dufourea novaeangliae] |
| map05206 MicroRNAs in cancer | Gene.1392 | 249 | hypothetical protein WN55_01867 [Dufourea novaeangliae] |
| map05206 MicroRNAs in cancer | Gene.1392 | 20 | hypothetical protein WN55_01867 [Dufourea novaeangliae] |
| map05206 MicroRNAs in cancer | Gene.1392 | 79 | hypothetical protein WN55_01867 [Dufourea novaeangliae] |
| map05206 MicroRNAs in cancer | Gene.2682 | 324 | heterogeneous nuclear ribonucleoprotein K isoform X1 [Anoplophora glabripennis] |
| map05206 MicroRNAs in cancer | Gene.1878 | 254 | MA3 domain containing protein [Asbolus verrucosus] |
| map05206 MicroRNAs in cancer | Gene.1878 | 248 | MA3 domain containing protein [Asbolus verrucosus] |
| map05206 MicroRNAs in cancer | Gene.1224 | 985 | PREDICTED: multidrug resistance protein 1 [Tribolium castaneum] |
| map05206 MicroRNAs in cancer | Gene.1224 | 393 | PREDICTED: multidrug resistance protein 1 [Tribolium castaneum] |
| map05206 MicroRNAs in cancer | Gene.1224 | 534 | PREDICTED: multidrug resistance protein 1 [Tribolium castaneum] |
| map05206 MicroRNAs in cancer | Gene.1224 | 268 | PREDICTED: multidrug resistance protein 1 [Tribolium castaneum] |
| map05206 MicroRNAs in cancer | Gene.1224 | 576 | PREDICTED: multidrug resistance protein 1 [Tribolium castaneum] |
| map05206 MicroRNAs in cancer | Gene.1224 | 1158 | PREDICTED: multidrug resistance protein 1 [Tribolium castaneum] |
| map05206 MicroRNAs in cancer | Gene.1871 | 452 | endoribonuclease Dicer [Anoplophora glabripennis] |
| map05206 MicroRNAs in cancer | Gene.281 | 367 | "tropomyosin-1, isoforms 9A/A/B isoform X4 [Anoplophora glabripennis]" |
| map05206 MicroRNAs in cancer | Gene.281 | 350 | "tropomyosin-1, isoforms 9A/A/B isoform X4 [Anoplophora glabripennis]" |
| map05206 MicroRNAs in cancer | Gene.281 | 409 | "tropomyosin-1, isoforms 9A/A/B isoform X4 [Anoplophora glabripennis]" |
| map04973 Carbohydrate digestion and absorption | Gene.8998 | 226 | "RecName: Full=Alpha-amylase; AltName: Full=1,4-alpha-D-glucan glucanohydrolase" |
| map04973 Carbohydrate digestion and absorption | Gene.8998 | 322 | "RecName: Full=Alpha-amylase; AltName: Full=1,4-alpha-D-glucan glucanohydrolase" |
| map04973 Carbohydrate digestion and absorption | Gene.2599 | 765 | PREDICTED: sodium/potassium-transporting ATPase subunit alpha isoform X3 [Tribolium castaneum] |
| map04973 Carbohydrate digestion and absorption | Gene.1533 | 280 | PREDICTED: hexokinase type 2 isoform X2 [Tribolium castaneum] |
| map04973 Carbohydrate digestion and absorption | Gene.1533 | 73 | PREDICTED: hexokinase type 2 isoform X2 [Tribolium castaneum] |
| map05100 Bacterial invasion of epithelial cells | Gene.7526 | 96 | ras-related protein Rac1 [Anoplophora glabripennis] |
| map05100 Bacterial invasion of epithelial cells | Gene.6425 | 116 | PREDICTED: clathrin light chain isoform X2 [Tribolium castaneum] |
| map05100 Bacterial invasion of epithelial cells | Gene.2830 | 84 | PREDICTED: LOW QUALITY PROTEIN: clathrin heavy chain [Aethina tumida] |
| map05100 Bacterial invasion of epithelial cells | Gene.2830 | 103 | PREDICTED: LOW QUALITY PROTEIN: clathrin heavy chain [Aethina tumida] |
| map05100 Bacterial invasion of epithelial cells | Gene.2830 | 902 | PREDICTED: LOW QUALITY PROTEIN: clathrin heavy chain [Aethina tumida] |
| map05100 Bacterial invasion of epithelial cells | Gene.2830 | 1619 | PREDICTED: LOW QUALITY PROTEIN: clathrin heavy chain [Aethina tumida] |
| map05100 Bacterial invasion of epithelial cells | Gene.172 | 216 | "beta-actin, partial [Cotesia chilonis]" |
| map05100 Bacterial invasion of epithelial cells | Gene.172 | 51 | "beta-actin, partial [Cotesia chilonis]" |
| map05100 Bacterial invasion of epithelial cells | Gene.172 | 114 | "beta-actin, partial [Cotesia chilonis]" |
| map05100 Bacterial invasion of epithelial cells | Gene.172 | 62 | "beta-actin, partial [Cotesia chilonis]" |
| map05100 Bacterial invasion of epithelial cells | Gene.2646 | 99 | beta actin [Polyrhachis vicina] |
| map05100 Bacterial invasion of epithelial cells | Gene.2646 | 88 | beta actin [Polyrhachis vicina] |
| map05100 Bacterial invasion of epithelial cells | Gene.2646 | 64 | beta actin [Polyrhachis vicina] |
| map05100 Bacterial invasion of epithelial cells | Gene.2646 | 101 | beta actin [Polyrhachis vicina] |
| map00010 Glycolysis / Gluconeogenesis | Gene.7815 | 345 | "probable pyruvate dehydrogenase E1 component subunit alpha, mitochondrial isoform X2 [Leptinotarsa decemlineata]" |
| map00010 Glycolysis / Gluconeogenesis | Gene.7815 | 315 | "probable pyruvate dehydrogenase E1 component subunit alpha, mitochondrial isoform X2 [Leptinotarsa decemlineata]" |
| map00010 Glycolysis / Gluconeogenesis | Gene.7815 | 317 | "probable pyruvate dehydrogenase E1 component subunit alpha, mitochondrial isoform X2 [Leptinotarsa decemlineata]" |
| map00010 Glycolysis / Gluconeogenesis | Gene.7815 | 77 | "probable pyruvate dehydrogenase E1 component subunit alpha, mitochondrial isoform X2 [Leptinotarsa decemlineata]" |
| map00010 Glycolysis / Gluconeogenesis | Gene.7815 | 330 | "probable pyruvate dehydrogenase E1 component subunit alpha, mitochondrial isoform X2 [Leptinotarsa decemlineata]" |
| map00010 Glycolysis / Gluconeogenesis | Gene.2997 | 50 | PREDICTED: pyruvate kinase-like isoform X3 [Dendroctonus ponderosae] |
| map00010 Glycolysis / Gluconeogenesis | Gene.2997 | 57 | PREDICTED: pyruvate kinase-like isoform X3 [Dendroctonus ponderosae] |
| map00010 Glycolysis / Gluconeogenesis | Gene.2997 | 310 | PREDICTED: pyruvate kinase-like isoform X3 [Dendroctonus ponderosae] |
| map00010 Glycolysis / Gluconeogenesis | Gene.2997 | 492 | PREDICTED: pyruvate kinase-like isoform X3 [Dendroctonus ponderosae] |
| map00010 Glycolysis / Gluconeogenesis | Gene.2997 | 94 | PREDICTED: pyruvate kinase-like isoform X3 [Dendroctonus ponderosae] |
| map00010 Glycolysis / Gluconeogenesis | Gene.2997 | 135 | PREDICTED: pyruvate kinase-like isoform X3 [Dendroctonus ponderosae] |
| map00010 Glycolysis / Gluconeogenesis | Gene.2997 | 377 | PREDICTED: pyruvate kinase-like isoform X3 [Dendroctonus ponderosae] |
| map00010 Glycolysis / Gluconeogenesis | Gene.2997 | 145 | PREDICTED: pyruvate kinase-like isoform X3 [Dendroctonus ponderosae] |
| map00010 Glycolysis / Gluconeogenesis | Gene.2997 | 264 | PREDICTED: pyruvate kinase-like isoform X3 [Dendroctonus ponderosae] |
| map00010 Glycolysis / Gluconeogenesis | Gene.2997 | 255 | PREDICTED: pyruvate kinase-like isoform X3 [Dendroctonus ponderosae] |
| map00010 Glycolysis / Gluconeogenesis | Gene.2997 | 140 | PREDICTED: pyruvate kinase-like isoform X3 [Dendroctonus ponderosae] |
| map00010 Glycolysis / Gluconeogenesis | Gene.2997 | 478 | PREDICTED: pyruvate kinase-like isoform X3 [Dendroctonus ponderosae] |
| map00010 Glycolysis / Gluconeogenesis | Gene.2533 | 286 | PREDICTED: putative aldehyde dehydrogenase family 7 member A1 homolog [Aethina tumida] |
| map00010 Glycolysis / Gluconeogenesis | Gene.2533 | 400 | PREDICTED: putative aldehyde dehydrogenase family 7 member A1 homolog [Aethina tumida] |
| map00010 Glycolysis / Gluconeogenesis | Gene.2533 | 41 | PREDICTED: putative aldehyde dehydrogenase family 7 member A1 homolog [Aethina tumida] |
| map00010 Glycolysis / Gluconeogenesis | Gene.2533 | 56 | PREDICTED: putative aldehyde dehydrogenase family 7 member A1 homolog [Aethina tumida] |
| map00010 Glycolysis / Gluconeogenesis | Gene.2533 | 382 | PREDICTED: putative aldehyde dehydrogenase family 7 member A1 homolog [Aethina tumida] |
| map00010 Glycolysis / Gluconeogenesis | Gene.2533 | 73 | PREDICTED: putative aldehyde dehydrogenase family 7 member A1 homolog [Aethina tumida] |
| map00010 Glycolysis / Gluconeogenesis | Gene.2533 | 62 | PREDICTED: putative aldehyde dehydrogenase family 7 member A1 homolog [Aethina tumida] |
| map00010 Glycolysis / Gluconeogenesis | Gene.2533 | 65 | PREDICTED: putative aldehyde dehydrogenase family 7 member A1 homolog [Aethina tumida] |
| map00010 Glycolysis / Gluconeogenesis | Gene.1633 | 147 | PREDICTED: fructose-bisphosphate aldolase [Tribolium castaneum] |
| map00010 Glycolysis / Gluconeogenesis | Gene.1633 | 42 | PREDICTED: fructose-bisphosphate aldolase [Tribolium castaneum] |
| map00010 Glycolysis / Gluconeogenesis | Gene.1633 | 28 | PREDICTED: fructose-bisphosphate aldolase [Tribolium castaneum] |
| map00010 Glycolysis / Gluconeogenesis | Gene.1533 | 280 | PREDICTED: hexokinase type 2 isoform X2 [Tribolium castaneum] |
| map00010 Glycolysis / Gluconeogenesis | Gene.1533 | 73 | PREDICTED: hexokinase type 2 isoform X2 [Tribolium castaneum] |
| map00010 Glycolysis / Gluconeogenesis | Gene.572 | 84 | PREDICTED: phosphoglycerate kinase [Tribolium castaneum] |
| map00010 Glycolysis / Gluconeogenesis | Gene.572 | 89 | PREDICTED: phosphoglycerate kinase [Tribolium castaneum] |
| map00010 Glycolysis / Gluconeogenesis | Gene.572 | 16 | PREDICTED: phosphoglycerate kinase [Tribolium castaneum] |
| map00010 Glycolysis / Gluconeogenesis | Gene.572 | 351 | PREDICTED: phosphoglycerate kinase [Tribolium castaneum] |
| map00010 Glycolysis / Gluconeogenesis | Gene.572 | 10 | PREDICTED: phosphoglycerate kinase [Tribolium castaneum] |
| map00010 Glycolysis / Gluconeogenesis | Gene.572 | 323 | PREDICTED: phosphoglycerate kinase [Tribolium castaneum] |
| map00010 Glycolysis / Gluconeogenesis | Gene.572 | 129 | PREDICTED: phosphoglycerate kinase [Tribolium castaneum] |
| map00010 Glycolysis / Gluconeogenesis | Gene.572 | 5 | PREDICTED: phosphoglycerate kinase [Tribolium castaneum] |
| map00010 Glycolysis / Gluconeogenesis | Gene.3901 | 291 | aldose 1-epimerase-like [Leptinotarsa decemlineata] |
| map00010 Glycolysis / Gluconeogenesis | Gene.3208 | 28 | phosphoglucose isomerase [Colias eurytheme] |
| map00010 Glycolysis / Gluconeogenesis | Gene.3208 | 462 | phosphoglucose isomerase [Colias eurytheme] |
| map00010 Glycolysis / Gluconeogenesis | Gene.3208 | 41 | phosphoglucose isomerase [Colias eurytheme] |
| map00010 Glycolysis / Gluconeogenesis | Gene.3075 | 162 | "dihydrolipoyl dehydrogenase, mitochondrial [Anoplophora glabripennis]" |
| map00010 Glycolysis / Gluconeogenesis | Gene.3075 | 118 | "dihydrolipoyl dehydrogenase, mitochondrial [Anoplophora glabripennis]" |
| map00010 Glycolysis / Gluconeogenesis | Gene.3075 | 259 | "dihydrolipoyl dehydrogenase, mitochondrial [Anoplophora glabripennis]" |
| map00010 Glycolysis / Gluconeogenesis | Gene.3075 | 273 | "dihydrolipoyl dehydrogenase, mitochondrial [Anoplophora glabripennis]" |
| map00010 Glycolysis / Gluconeogenesis | Gene.3075 | 139 | "dihydrolipoyl dehydrogenase, mitochondrial [Anoplophora glabripennis]" |
| map00010 Glycolysis / Gluconeogenesis | Gene.1891 | 103 | hypothetical protein AMK59_3112 [Oryctes borbonicus] |
| map00010 Glycolysis / Gluconeogenesis | Gene.1891 | 88 | hypothetical protein AMK59_3112 [Oryctes borbonicus] |
| map00010 Glycolysis / Gluconeogenesis | Gene.1891 | 71 | hypothetical protein AMK59_3112 [Oryctes borbonicus] |
| map00010 Glycolysis / Gluconeogenesis | Gene.1891 | 96 | hypothetical protein AMK59_3112 [Oryctes borbonicus] |
| map00010 Glycolysis / Gluconeogenesis | Gene.196 | 25 | PREDICTED: triosephosphate isomerase isoform X1 [Aethina tumida] |
| map00010 Glycolysis / Gluconeogenesis | Gene.196 | 197 | PREDICTED: triosephosphate isomerase isoform X1 [Aethina tumida] |
| map00010 Glycolysis / Gluconeogenesis | Gene.196 | 189 | PREDICTED: triosephosphate isomerase isoform X1 [Aethina tumida] |
| map00010 Glycolysis / Gluconeogenesis | Gene.196 | 67 | PREDICTED: triosephosphate isomerase isoform X1 [Aethina tumida] |
| map00010 Glycolysis / Gluconeogenesis | Gene.196 | 217 | PREDICTED: triosephosphate isomerase isoform X1 [Aethina tumida] |
| map00010 Glycolysis / Gluconeogenesis | Gene.196 | 83 | PREDICTED: triosephosphate isomerase isoform X1 [Aethina tumida] |
| map00010 Glycolysis / Gluconeogenesis | Gene.196 | 70 | PREDICTED: triosephosphate isomerase isoform X1 [Aethina tumida] |
| map00010 Glycolysis / Gluconeogenesis | Gene.196 | 118 | PREDICTED: triosephosphate isomerase isoform X1 [Aethina tumida] |
| map00010 Glycolysis / Gluconeogenesis | Gene.196 | 12 | PREDICTED: triosephosphate isomerase isoform X1 [Aethina tumida] |
| map00010 Glycolysis / Gluconeogenesis | Gene.196 | 173 | PREDICTED: triosephosphate isomerase isoform X1 [Aethina tumida] |
| map00010 Glycolysis / Gluconeogenesis | Gene.986 | 265 | "PREDICTED: aldehyde dehydrogenase, dimeric NADP-preferring [Tribolium castaneum]" |
| map00010 Glycolysis / Gluconeogenesis | Gene.986 | 452 | "PREDICTED: aldehyde dehydrogenase, dimeric NADP-preferring [Tribolium castaneum]" |
| map00010 Glycolysis / Gluconeogenesis | Gene.986 | 19 | "PREDICTED: aldehyde dehydrogenase, dimeric NADP-preferring [Tribolium castaneum]" |
| map00010 Glycolysis / Gluconeogenesis | Gene.8989 | 179 | "PREDICTED: aldehyde dehydrogenase, dimeric NADP-preferring-like [Aethina tumida]" |
| map00010 Glycolysis / Gluconeogenesis | Gene.8989 | 258 | "PREDICTED: aldehyde dehydrogenase, dimeric NADP-preferring-like [Aethina tumida]" |
| map00010 Glycolysis / Gluconeogenesis | Gene.8989 | 438 | "PREDICTED: aldehyde dehydrogenase, dimeric NADP-preferring-like [Aethina tumida]" |
| map00010 Glycolysis / Gluconeogenesis | Gene.468 | 427 | "retinal dehydrogenase 1, partial [Asbolus verrucosus]" |
| map00010 Glycolysis / Gluconeogenesis | Gene.468 | 27 | "retinal dehydrogenase 1, partial [Asbolus verrucosus]" |
| map00010 Glycolysis / Gluconeogenesis | Gene.468 | 131 | "retinal dehydrogenase 1, partial [Asbolus verrucosus]" |
| map00010 Glycolysis / Gluconeogenesis | Gene.468 | 20 | "retinal dehydrogenase 1, partial [Asbolus verrucosus]" |
| map00010 Glycolysis / Gluconeogenesis | Gene.2375 | 84 | "glyceraldehyde-3-phosphate, partial [Harmonia axyridis]" |
| map00010 Glycolysis / Gluconeogenesis | Gene.2375 | 252 | "glyceraldehyde-3-phosphate, partial [Harmonia axyridis]" |
| map00010 Glycolysis / Gluconeogenesis | Gene.2375 | 90 | "glyceraldehyde-3-phosphate, partial [Harmonia axyridis]" |
| map00010 Glycolysis / Gluconeogenesis | Gene.2375 | 228 | "glyceraldehyde-3-phosphate, partial [Harmonia axyridis]" |
| map00010 Glycolysis / Gluconeogenesis | Gene.2375 | 330 | "glyceraldehyde-3-phosphate, partial [Harmonia axyridis]" |
| map00010 Glycolysis / Gluconeogenesis | Gene.2375 | 58 | "glyceraldehyde-3-phosphate, partial [Harmonia axyridis]" |
| map00010 Glycolysis / Gluconeogenesis | Gene.2375 | 163 | "glyceraldehyde-3-phosphate, partial [Harmonia axyridis]" |
| map00010 Glycolysis / Gluconeogenesis | Gene.2375 | 66 | "glyceraldehyde-3-phosphate, partial [Harmonia axyridis]" |
| map00010 Glycolysis / Gluconeogenesis | Gene.2375 | 73 | "glyceraldehyde-3-phosphate, partial [Harmonia axyridis]" |
| map00010 Glycolysis / Gluconeogenesis | Gene.2375 | 260 | "glyceraldehyde-3-phosphate, partial [Harmonia axyridis]" |
| map00010 Glycolysis / Gluconeogenesis | Gene.2375 | 264 | "glyceraldehyde-3-phosphate, partial [Harmonia axyridis]" |
| map00010 Glycolysis / Gluconeogenesis | Gene.2375 | 223 | "glyceraldehyde-3-phosphate, partial [Harmonia axyridis]" |
| map00010 Glycolysis / Gluconeogenesis | Gene.2375 | 195 | "glyceraldehyde-3-phosphate, partial [Harmonia axyridis]" |
| map00010 Glycolysis / Gluconeogenesis | Gene.1892 | 205 | PREDICTED: enolase [Musca domestica] |
| map00010 Glycolysis / Gluconeogenesis | Gene.1892 | 74 | PREDICTED: enolase [Musca domestica] |
| map00010 Glycolysis / Gluconeogenesis | Gene.1892 | 221 | PREDICTED: enolase [Musca domestica] |
| map00010 Glycolysis / Gluconeogenesis | Gene.1892 | 225 | PREDICTED: enolase [Musca domestica] |
| map00010 Glycolysis / Gluconeogenesis | Gene.1892 | 66 | PREDICTED: enolase [Musca domestica] |
| map00010 Glycolysis / Gluconeogenesis | Gene.1892 | 80 | PREDICTED: enolase [Musca domestica] |
| map00010 Glycolysis / Gluconeogenesis | Gene.1892 | 133 | PREDICTED: enolase [Musca domestica] |
| map00010 Glycolysis / Gluconeogenesis | Gene.8424 | 34 | PREDICTED: NAD/NADP-dependent betaine aldehyde dehydrogenase [Tribolium castaneum] |
| map00010 Glycolysis / Gluconeogenesis | Gene.8424 | 532 | PREDICTED: NAD/NADP-dependent betaine aldehyde dehydrogenase [Tribolium castaneum] |
| map00010 Glycolysis / Gluconeogenesis | Gene.7878 | 6 | alcohol dehydrogenase class-3 [Leptinotarsa decemlineata] |
| map00010 Glycolysis / Gluconeogenesis | Gene.7878 | 340 | alcohol dehydrogenase class-3 [Leptinotarsa decemlineata] |
| map00010 Glycolysis / Gluconeogenesis | Gene.3247 | 93 | PREDICTED: phosphoenolpyruvate carboxykinase [GTP] isoform X1 [Tribolium castaneum] |
| map00010 Glycolysis / Gluconeogenesis | Gene.8318 | 197 | PGM PMM I domain containing protein [Asbolus verrucosus] |
| map00010 Glycolysis / Gluconeogenesis | Gene.8318 | 466 | PGM PMM I domain containing protein [Asbolus verrucosus] |
| map00010 Glycolysis / Gluconeogenesis | Gene.8318 | 204 | PGM PMM I domain containing protein [Asbolus verrucosus] |
| map00010 Glycolysis / Gluconeogenesis | Gene.8318 | 486 | PGM PMM I domain containing protein [Asbolus verrucosus] |
| map00010 Glycolysis / Gluconeogenesis | Gene.8318 | 489 | PGM PMM I domain containing protein [Asbolus verrucosus] |
| map00010 Glycolysis / Gluconeogenesis | Gene.8318 | 9 | PGM PMM I domain containing protein [Asbolus verrucosus] |
| map00010 Glycolysis / Gluconeogenesis | Gene.8318 | 348 | PGM PMM I domain containing protein [Asbolus verrucosus] |
| map00010 Glycolysis / Gluconeogenesis | Gene.8318 | 421 | PGM PMM I domain containing protein [Asbolus verrucosus] |
| map00010 Glycolysis / Gluconeogenesis | Gene.8318 | 352 | PGM PMM I domain containing protein [Asbolus verrucosus] |
| map00010 Glycolysis / Gluconeogenesis | Gene.8318 | 278 | PGM PMM I domain containing protein [Asbolus verrucosus] |
| map00010 Glycolysis / Gluconeogenesis | Gene.8318 | 151 | PGM PMM I domain containing protein [Asbolus verrucosus] |
| map00010 Glycolysis / Gluconeogenesis | Gene.8318 | 459 | PGM PMM I domain containing protein [Asbolus verrucosus] |
| map00010 Glycolysis / Gluconeogenesis | Gene.8318 | 449 | PGM PMM I domain containing protein [Asbolus verrucosus] |
| map00010 Glycolysis / Gluconeogenesis | Gene.4285 | 93 | PREDICTED: LOW QUALITY PROTEIN: pyruvate kinase-like [Aethina tumida] |
| map00010 Glycolysis / Gluconeogenesis | Gene.4285 | 245 | PREDICTED: LOW QUALITY PROTEIN: pyruvate kinase-like [Aethina tumida] |
| map00010 Glycolysis / Gluconeogenesis | Gene.4366 | 47 | phosphoglucomutase-2 [Leptinotarsa decemlineata] |
| map00010 Glycolysis / Gluconeogenesis | Gene.2569 | 315 | "1,5-anhydro-D-fructose reductase-like Protein [Tribolium castaneum]" |
| map00010 Glycolysis / Gluconeogenesis | Gene.2569 | 27 | "1,5-anhydro-D-fructose reductase-like Protein [Tribolium castaneum]" |
| map00010 Glycolysis / Gluconeogenesis | Gene.2569 | 4 | "1,5-anhydro-D-fructose reductase-like Protein [Tribolium castaneum]" |
| map00010 Glycolysis / Gluconeogenesis | Gene.2569 | 60 | "1,5-anhydro-D-fructose reductase-like Protein [Tribolium castaneum]" |
| map00010 Glycolysis / Gluconeogenesis | Gene.2569 | 56 | "1,5-anhydro-D-fructose reductase-like Protein [Tribolium castaneum]" |
| map00010 Glycolysis / Gluconeogenesis | Gene.2569 | 70 | "1,5-anhydro-D-fructose reductase-like Protein [Tribolium castaneum]" |
| map00010 Glycolysis / Gluconeogenesis | Gene.2569 | 169 | "1,5-anhydro-D-fructose reductase-like Protein [Tribolium castaneum]" |
| map00010 Glycolysis / Gluconeogenesis | Gene.2569 | 9 | "1,5-anhydro-D-fructose reductase-like Protein [Tribolium castaneum]" |
| map00010 Glycolysis / Gluconeogenesis | Gene.2749 | 84 | "PREDICTED: aldehyde dehydrogenase, mitochondrial [Tribolium castaneum]" |
| map00010 Glycolysis / Gluconeogenesis | Gene.2749 | 370 | "PREDICTED: aldehyde dehydrogenase, mitochondrial [Tribolium castaneum]" |
| map00010 Glycolysis / Gluconeogenesis | Gene.2749 | 446 | "PREDICTED: aldehyde dehydrogenase, mitochondrial [Tribolium castaneum]" |
| map00010 Glycolysis / Gluconeogenesis | Gene.2749 | 151 | "PREDICTED: aldehyde dehydrogenase, mitochondrial [Tribolium castaneum]" |
| map00010 Glycolysis / Gluconeogenesis | Gene.2749 | 140 | "PREDICTED: aldehyde dehydrogenase, mitochondrial [Tribolium castaneum]" |
| map00010 Glycolysis / Gluconeogenesis | Gene.2749 | 423 | "PREDICTED: aldehyde dehydrogenase, mitochondrial [Tribolium castaneum]" |
| map00010 Glycolysis / Gluconeogenesis | Gene.3972 | 228 | "pyruvate dehydrogenase E1 component subunit beta, mitochondrial [Leptinotarsa decemlineata]" |
| map00010 Glycolysis / Gluconeogenesis | Gene.3972 | 185 | "pyruvate dehydrogenase E1 component subunit beta, mitochondrial [Leptinotarsa decemlineata]" |
| map00010 Glycolysis / Gluconeogenesis | Gene.3972 | 260 | "pyruvate dehydrogenase E1 component subunit beta, mitochondrial [Leptinotarsa decemlineata]" |
| map00010 Glycolysis / Gluconeogenesis | Gene.5844 | 181 | phosphoglycerate mutase 1 [Asbolus verrucosus] |
| map00010 Glycolysis / Gluconeogenesis | Gene.5844 | 165 | phosphoglycerate mutase 1 [Asbolus verrucosus] |
| map00010 Glycolysis / Gluconeogenesis | Gene.5844 | 68 | phosphoglycerate mutase 1 [Asbolus verrucosus] |
| map00010 Glycolysis / Gluconeogenesis | Gene.5844 | 122 | phosphoglycerate mutase 1 [Asbolus verrucosus] |
| map00010 Glycolysis / Gluconeogenesis | Gene.5844 | 105 | phosphoglycerate mutase 1 [Asbolus verrucosus] |
| map00010 Glycolysis / Gluconeogenesis | Gene.5844 | 176 | phosphoglycerate mutase 1 [Asbolus verrucosus] |
| map00010 Glycolysis / Gluconeogenesis | Gene.8616 | 445 | "dihydrolipoyllysine-residue acetyltransferase component of pyruvate dehydrogenase complex, mitochondrial isoform X1 [Anoplophora glabripennis]" |
| map00010 Glycolysis / Gluconeogenesis | Gene.8616 | 131 | "dihydrolipoyllysine-residue acetyltransferase component of pyruvate dehydrogenase complex, mitochondrial isoform X1 [Anoplophora glabripennis]" |
| map00010 Glycolysis / Gluconeogenesis | Gene.8616 | 474 | "dihydrolipoyllysine-residue acetyltransferase component of pyruvate dehydrogenase complex, mitochondrial isoform X1 [Anoplophora glabripennis]" |
| map00010 Glycolysis / Gluconeogenesis | Gene.8616 | 156 | "dihydrolipoyllysine-residue acetyltransferase component of pyruvate dehydrogenase complex, mitochondrial isoform X1 [Anoplophora glabripennis]" |
| map00010 Glycolysis / Gluconeogenesis | Gene.8616 | 388 | "dihydrolipoyllysine-residue acetyltransferase component of pyruvate dehydrogenase complex, mitochondrial isoform X1 [Anoplophora glabripennis]" |
| map00010 Glycolysis / Gluconeogenesis | Gene.8616 | 380 | "dihydrolipoyllysine-residue acetyltransferase component of pyruvate dehydrogenase complex, mitochondrial isoform X1 [Anoplophora glabripennis]" |
| map05414 Dilated cardiomyopathy (DCM) | Gene.5709 | 26 | cAMP-dependent protein kinase catalytic subunit [Agrilus planipennis] |
| map05414 Dilated cardiomyopathy (DCM) | Gene.5709 | 257 | cAMP-dependent protein kinase catalytic subunit [Agrilus planipennis] |
| map05414 Dilated cardiomyopathy (DCM) | Gene.1392 | 166 | hypothetical protein WN55_01867 [Dufourea novaeangliae] |
| map05414 Dilated cardiomyopathy (DCM) | Gene.1392 | 43 | hypothetical protein WN55_01867 [Dufourea novaeangliae] |
| map05414 Dilated cardiomyopathy (DCM) | Gene.1392 | 117 | hypothetical protein WN55_01867 [Dufourea novaeangliae] |
| map05414 Dilated cardiomyopathy (DCM) | Gene.1392 | 249 | hypothetical protein WN55_01867 [Dufourea novaeangliae] |
| map05414 Dilated cardiomyopathy (DCM) | Gene.1392 | 20 | hypothetical protein WN55_01867 [Dufourea novaeangliae] |
| map05414 Dilated cardiomyopathy (DCM) | Gene.1392 | 79 | hypothetical protein WN55_01867 [Dufourea novaeangliae] |
| map05414 Dilated cardiomyopathy (DCM) | Gene.4352 | 128 | PREDICTED: calcium-transporting ATPase sarcoplasmic/endoplasmic reticulum type isoform X1 [Tribolium castaneum] |
| map05414 Dilated cardiomyopathy (DCM) | Gene.4352 | 514 | PREDICTED: calcium-transporting ATPase sarcoplasmic/endoplasmic reticulum type isoform X1 [Tribolium castaneum] |
| map05414 Dilated cardiomyopathy (DCM) | Gene.4352 | 30 | PREDICTED: calcium-transporting ATPase sarcoplasmic/endoplasmic reticulum type isoform X1 [Tribolium castaneum] |
| map05414 Dilated cardiomyopathy (DCM) | Gene.281 | 367 | "tropomyosin-1, isoforms 9A/A/B isoform X4 [Anoplophora glabripennis]" |
| map05414 Dilated cardiomyopathy (DCM) | Gene.281 | 350 | "tropomyosin-1, isoforms 9A/A/B isoform X4 [Anoplophora glabripennis]" |
| map05414 Dilated cardiomyopathy (DCM) | Gene.281 | 409 | "tropomyosin-1, isoforms 9A/A/B isoform X4 [Anoplophora glabripennis]" |
| map05414 Dilated cardiomyopathy (DCM) | Gene.172 | 216 | "beta-actin, partial [Cotesia chilonis]" |
| map05414 Dilated cardiomyopathy (DCM) | Gene.172 | 51 | "beta-actin, partial [Cotesia chilonis]" |
| map05414 Dilated cardiomyopathy (DCM) | Gene.172 | 114 | "beta-actin, partial [Cotesia chilonis]" |
| map05414 Dilated cardiomyopathy (DCM) | Gene.172 | 62 | "beta-actin, partial [Cotesia chilonis]" |
| map05414 Dilated cardiomyopathy (DCM) | Gene.4973 | 1173 | "PREDICTED: myosin heavy chain, muscle isoform X18 [Tribolium castaneum]" |
| map05414 Dilated cardiomyopathy (DCM) | Gene.4973 | 1579 | "PREDICTED: myosin heavy chain, muscle isoform X18 [Tribolium castaneum]" |
| map05414 Dilated cardiomyopathy (DCM) | Gene.4973 | 1457 | "PREDICTED: myosin heavy chain, muscle isoform X18 [Tribolium castaneum]" |
| map05414 Dilated cardiomyopathy (DCM) | Gene.4973 | 1332 | "PREDICTED: myosin heavy chain, muscle isoform X18 [Tribolium castaneum]" |
| map05414 Dilated cardiomyopathy (DCM) | Gene.4973 | 907 | "PREDICTED: myosin heavy chain, muscle isoform X18 [Tribolium castaneum]" |
| map05414 Dilated cardiomyopathy (DCM) | Gene.4973 | 1641 | "PREDICTED: myosin heavy chain, muscle isoform X18 [Tribolium castaneum]" |
| map05414 Dilated cardiomyopathy (DCM) | Gene.4973 | 599 | "PREDICTED: myosin heavy chain, muscle isoform X18 [Tribolium castaneum]" |
| map05414 Dilated cardiomyopathy (DCM) | Gene.4973 | 1390 | "PREDICTED: myosin heavy chain, muscle isoform X18 [Tribolium castaneum]" |
| map05414 Dilated cardiomyopathy (DCM) | Gene.4973 | 84 | "PREDICTED: myosin heavy chain, muscle isoform X18 [Tribolium castaneum]" |
| map05414 Dilated cardiomyopathy (DCM) | Gene.4973 | 1005 | "PREDICTED: myosin heavy chain, muscle isoform X18 [Tribolium castaneum]" |
| map05414 Dilated cardiomyopathy (DCM) | Gene.4973 | 1919 | "PREDICTED: myosin heavy chain, muscle isoform X18 [Tribolium castaneum]" |
| map05414 Dilated cardiomyopathy (DCM) | Gene.4973 | 895 | "PREDICTED: myosin heavy chain, muscle isoform X18 [Tribolium castaneum]" |
| map05414 Dilated cardiomyopathy (DCM) | Gene.4973 | 450 | "PREDICTED: myosin heavy chain, muscle isoform X18 [Tribolium castaneum]" |
| map05414 Dilated cardiomyopathy (DCM) | Gene.4973 | 551 | "PREDICTED: myosin heavy chain, muscle isoform X18 [Tribolium castaneum]" |
| map05414 Dilated cardiomyopathy (DCM) | Gene.4973 | 879 | "PREDICTED: myosin heavy chain, muscle isoform X18 [Tribolium castaneum]" |
| map05414 Dilated cardiomyopathy (DCM) | Gene.4973 | 1416 | "PREDICTED: myosin heavy chain, muscle isoform X18 [Tribolium castaneum]" |
| map05414 Dilated cardiomyopathy (DCM) | Gene.4973 | 611 | "PREDICTED: myosin heavy chain, muscle isoform X18 [Tribolium castaneum]" |
| map05414 Dilated cardiomyopathy (DCM) | Gene.4973 | 1109 | "PREDICTED: myosin heavy chain, muscle isoform X18 [Tribolium castaneum]" |
| map05414 Dilated cardiomyopathy (DCM) | Gene.4973 | 721 | "PREDICTED: myosin heavy chain, muscle isoform X18 [Tribolium castaneum]" |
| map05414 Dilated cardiomyopathy (DCM) | Gene.4973 | 1838 | "PREDICTED: myosin heavy chain, muscle isoform X18 [Tribolium castaneum]" |
| map05414 Dilated cardiomyopathy (DCM) | Gene.4973 | 1262 | "PREDICTED: myosin heavy chain, muscle isoform X18 [Tribolium castaneum]" |
| map05414 Dilated cardiomyopathy (DCM) | Gene.4973 | 1444 | "PREDICTED: myosin heavy chain, muscle isoform X18 [Tribolium castaneum]" |
| map05414 Dilated cardiomyopathy (DCM) | Gene.4973 | 1277 | "PREDICTED: myosin heavy chain, muscle isoform X18 [Tribolium castaneum]" |
| map05414 Dilated cardiomyopathy (DCM) | Gene.4973 | 1247 | "PREDICTED: myosin heavy chain, muscle isoform X18 [Tribolium castaneum]" |
| map05414 Dilated cardiomyopathy (DCM) | Gene.4973 | 971 | "PREDICTED: myosin heavy chain, muscle isoform X18 [Tribolium castaneum]" |
| map05414 Dilated cardiomyopathy (DCM) | Gene.4973 | 851 | "PREDICTED: myosin heavy chain, muscle isoform X18 [Tribolium castaneum]" |
| map05414 Dilated cardiomyopathy (DCM) | Gene.4973 | 1316 | "PREDICTED: myosin heavy chain, muscle isoform X18 [Tribolium castaneum]" |
| map05414 Dilated cardiomyopathy (DCM) | Gene.4973 | 1374 | "PREDICTED: myosin heavy chain, muscle isoform X18 [Tribolium castaneum]" |
| map05414 Dilated cardiomyopathy (DCM) | Gene.4973 | 951 | "PREDICTED: myosin heavy chain, muscle isoform X18 [Tribolium castaneum]" |
| map05414 Dilated cardiomyopathy (DCM) | Gene.4973 | 1354 | "PREDICTED: myosin heavy chain, muscle isoform X18 [Tribolium castaneum]" |
| map05414 Dilated cardiomyopathy (DCM) | Gene.4973 | 429 | "PREDICTED: myosin heavy chain, muscle isoform X18 [Tribolium castaneum]" |
| map05414 Dilated cardiomyopathy (DCM) | Gene.4973 | 1103 | "PREDICTED: myosin heavy chain, muscle isoform X18 [Tribolium castaneum]" |
| map05414 Dilated cardiomyopathy (DCM) | Gene.4973 | 1093 | "PREDICTED: myosin heavy chain, muscle isoform X18 [Tribolium castaneum]" |
| map05414 Dilated cardiomyopathy (DCM) | Gene.4973 | 1451 | "PREDICTED: myosin heavy chain, muscle isoform X18 [Tribolium castaneum]" |
| map05414 Dilated cardiomyopathy (DCM) | Gene.4973 | 940 | "PREDICTED: myosin heavy chain, muscle isoform X18 [Tribolium castaneum]" |
| map05414 Dilated cardiomyopathy (DCM) | Gene.4973 | 1791 | "PREDICTED: myosin heavy chain, muscle isoform X18 [Tribolium castaneum]" |
| map05414 Dilated cardiomyopathy (DCM) | Gene.2646 | 99 | beta actin [Polyrhachis vicina] |
| map05414 Dilated cardiomyopathy (DCM) | Gene.2646 | 88 | beta actin [Polyrhachis vicina] |
| map05414 Dilated cardiomyopathy (DCM) | Gene.2646 | 64 | beta actin [Polyrhachis vicina] |
| map05414 Dilated cardiomyopathy (DCM) | Gene.2646 | 101 | beta actin [Polyrhachis vicina] |
| map05214 Glioma | Gene.298 | 116 | "Csa-calmodulin 3, partial [Cupiennius salei]" |
| map05214 Glioma | Gene.298 | 95 | "Csa-calmodulin 3, partial [Cupiennius salei]" |
| map05214 Glioma | Gene.298 | 31 | "Csa-calmodulin 3, partial [Cupiennius salei]" |
| map05214 Glioma | Gene.298 | 78 | "Csa-calmodulin 3, partial [Cupiennius salei]" |
| map05214 Glioma | Gene.5803 | 20 | calcium-binding protein E63-1 [Anoplophora glabripennis] |
| map05133 Pertussis | Gene.8288 | 19 | cofilin/actin-depolymerizing factor homolog [Anoplophora glabripennis] |
| map05133 Pertussis | Gene.8288 | 41 | cofilin/actin-depolymerizing factor homolog [Anoplophora glabripennis] |
| map05133 Pertussis | Gene.298 | 116 | "Csa-calmodulin 3, partial [Cupiennius salei]" |
| map05133 Pertussis | Gene.298 | 95 | "Csa-calmodulin 3, partial [Cupiennius salei]" |
| map05133 Pertussis | Gene.298 | 31 | "Csa-calmodulin 3, partial [Cupiennius salei]" |
| map05133 Pertussis | Gene.298 | 78 | "Csa-calmodulin 3, partial [Cupiennius salei]" |
| map05133 Pertussis | Gene.5803 | 20 | calcium-binding protein E63-1 [Anoplophora glabripennis] |
| map05230 Central carbon metabolism in cancer | Gene.3972 | 228 | "pyruvate dehydrogenase E1 component subunit beta, mitochondrial [Leptinotarsa decemlineata]" |
| map05230 Central carbon metabolism in cancer | Gene.3972 | 185 | "pyruvate dehydrogenase E1 component subunit beta, mitochondrial [Leptinotarsa decemlineata]" |
| map05230 Central carbon metabolism in cancer | Gene.3972 | 260 | "pyruvate dehydrogenase E1 component subunit beta, mitochondrial [Leptinotarsa decemlineata]" |
| map05230 Central carbon metabolism in cancer | Gene.7815 | 345 | "probable pyruvate dehydrogenase E1 component subunit alpha, mitochondrial isoform X2 [Leptinotarsa decemlineata]" |
| map05230 Central carbon metabolism in cancer | Gene.7815 | 315 | "probable pyruvate dehydrogenase E1 component subunit alpha, mitochondrial isoform X2 [Leptinotarsa decemlineata]" |
| map05230 Central carbon metabolism in cancer | Gene.7815 | 317 | "probable pyruvate dehydrogenase E1 component subunit alpha, mitochondrial isoform X2 [Leptinotarsa decemlineata]" |
| map05230 Central carbon metabolism in cancer | Gene.7815 | 77 | "probable pyruvate dehydrogenase E1 component subunit alpha, mitochondrial isoform X2 [Leptinotarsa decemlineata]" |
| map05230 Central carbon metabolism in cancer | Gene.7815 | 330 | "probable pyruvate dehydrogenase E1 component subunit alpha, mitochondrial isoform X2 [Leptinotarsa decemlineata]" |
| map05230 Central carbon metabolism in cancer | Gene.4285 | 93 | PREDICTED: LOW QUALITY PROTEIN: pyruvate kinase-like [Aethina tumida] |
| map05230 Central carbon metabolism in cancer | Gene.4285 | 245 | PREDICTED: LOW QUALITY PROTEIN: pyruvate kinase-like [Aethina tumida] |
| map05230 Central carbon metabolism in cancer | Gene.2997 | 50 | PREDICTED: pyruvate kinase-like isoform X3 [Dendroctonus ponderosae] |
| map05230 Central carbon metabolism in cancer | Gene.2997 | 57 | PREDICTED: pyruvate kinase-like isoform X3 [Dendroctonus ponderosae] |
| map05230 Central carbon metabolism in cancer | Gene.2997 | 310 | PREDICTED: pyruvate kinase-like isoform X3 [Dendroctonus ponderosae] |
| map05230 Central carbon metabolism in cancer | Gene.2997 | 492 | PREDICTED: pyruvate kinase-like isoform X3 [Dendroctonus ponderosae] |
| map05230 Central carbon metabolism in cancer | Gene.2997 | 94 | PREDICTED: pyruvate kinase-like isoform X3 [Dendroctonus ponderosae] |
| map05230 Central carbon metabolism in cancer | Gene.2997 | 135 | PREDICTED: pyruvate kinase-like isoform X3 [Dendroctonus ponderosae] |
| map05230 Central carbon metabolism in cancer | Gene.2997 | 377 | PREDICTED: pyruvate kinase-like isoform X3 [Dendroctonus ponderosae] |
| map05230 Central carbon metabolism in cancer | Gene.2997 | 145 | PREDICTED: pyruvate kinase-like isoform X3 [Dendroctonus ponderosae] |
| map05230 Central carbon metabolism in cancer | Gene.2997 | 264 | PREDICTED: pyruvate kinase-like isoform X3 [Dendroctonus ponderosae] |
| map05230 Central carbon metabolism in cancer | Gene.2997 | 255 | PREDICTED: pyruvate kinase-like isoform X3 [Dendroctonus ponderosae] |
| map05230 Central carbon metabolism in cancer | Gene.2997 | 140 | PREDICTED: pyruvate kinase-like isoform X3 [Dendroctonus ponderosae] |
| map05230 Central carbon metabolism in cancer | Gene.2997 | 478 | PREDICTED: pyruvate kinase-like isoform X3 [Dendroctonus ponderosae] |
| map05230 Central carbon metabolism in cancer | Gene.5844 | 181 | phosphoglycerate mutase 1 [Asbolus verrucosus] |
| map05230 Central carbon metabolism in cancer | Gene.5844 | 165 | phosphoglycerate mutase 1 [Asbolus verrucosus] |
| map05230 Central carbon metabolism in cancer | Gene.5844 | 68 | phosphoglycerate mutase 1 [Asbolus verrucosus] |
| map05230 Central carbon metabolism in cancer | Gene.5844 | 122 | phosphoglycerate mutase 1 [Asbolus verrucosus] |
| map05230 Central carbon metabolism in cancer | Gene.5844 | 105 | phosphoglycerate mutase 1 [Asbolus verrucosus] |
| map05230 Central carbon metabolism in cancer | Gene.5844 | 176 | phosphoglycerate mutase 1 [Asbolus verrucosus] |
| map05230 Central carbon metabolism in cancer | Gene.1533 | 280 | PREDICTED: hexokinase type 2 isoform X2 [Tribolium castaneum] |
| map05230 Central carbon metabolism in cancer | Gene.1533 | 73 | PREDICTED: hexokinase type 2 isoform X2 [Tribolium castaneum] |
| map00400 Phenylalanine, tyrosine and tryptophan biosynthesis | Gene.2656 | 56 | "PREDICTED: aspartate aminotransferase, cytoplasmic [Tribolium castaneum]" |
| map00400 Phenylalanine, tyrosine and tryptophan biosynthesis | Gene.2656 | 141 | "PREDICTED: aspartate aminotransferase, cytoplasmic [Tribolium castaneum]" |
| map00400 Phenylalanine, tyrosine and tryptophan biosynthesis | Gene.1489 | 401 | "aspartate aminotransferase, mitochondrial [Asbolus verrucosus]" |
| map00400 Phenylalanine, tyrosine and tryptophan biosynthesis | Gene.1489 | 87 | "aspartate aminotransferase, mitochondrial [Asbolus verrucosus]" |
| map00400 Phenylalanine, tyrosine and tryptophan biosynthesis | Gene.1489 | 189 | "aspartate aminotransferase, mitochondrial [Asbolus verrucosus]" |
| map00400 Phenylalanine, tyrosine and tryptophan biosynthesis | Gene.1489 | 334 | "aspartate aminotransferase, mitochondrial [Asbolus verrucosus]" |
| map00400 Phenylalanine, tyrosine and tryptophan biosynthesis | Gene.1489 | 174 | "aspartate aminotransferase, mitochondrial [Asbolus verrucosus]" |
| map00400 Phenylalanine, tyrosine and tryptophan biosynthesis | Gene.1489 | 393 | "aspartate aminotransferase, mitochondrial [Asbolus verrucosus]" |
| map00400 Phenylalanine, tyrosine and tryptophan biosynthesis | Gene.1489 | 332 | "aspartate aminotransferase, mitochondrial [Asbolus verrucosus]" |
| map00400 Phenylalanine, tyrosine and tryptophan biosynthesis | Gene.1489 | 91 | "aspartate aminotransferase, mitochondrial [Asbolus verrucosus]" |
| map00400 Phenylalanine, tyrosine and tryptophan biosynthesis | Gene.1489 | 295 | "aspartate aminotransferase, mitochondrial [Asbolus verrucosus]" |
| map00531 Glycosaminoglycan degradation | Gene.3252 | 101 | chitooligosaccharidolytic beta-N-acetylglucosaminidase isoform X2 [Anoplophora glabripennis] |
| map04972 Pancreatic secretion | Gene.8998 | 226 | "RecName: Full=Alpha-amylase; AltName: Full=1,4-alpha-D-glucan glucanohydrolase" |
| map04972 Pancreatic secretion | Gene.8998 | 322 | "RecName: Full=Alpha-amylase; AltName: Full=1,4-alpha-D-glucan glucanohydrolase" |
| map04972 Pancreatic secretion | Gene.7676 | 53 | PREDICTED: guanine nucleotide-binding protein G(q) subunit alpha isoform X1 [Tribolium castaneum] |
| map04972 Pancreatic secretion | Gene.7526 | 96 | ras-related protein Rac1 [Anoplophora glabripennis] |
| map04972 Pancreatic secretion | Gene.4352 | 128 | PREDICTED: calcium-transporting ATPase sarcoplasmic/endoplasmic reticulum type isoform X1 [Tribolium castaneum] |
| map04972 Pancreatic secretion | Gene.4352 | 514 | PREDICTED: calcium-transporting ATPase sarcoplasmic/endoplasmic reticulum type isoform X1 [Tribolium castaneum] |
| map04972 Pancreatic secretion | Gene.4352 | 30 | PREDICTED: calcium-transporting ATPase sarcoplasmic/endoplasmic reticulum type isoform X1 [Tribolium castaneum] |
| map04972 Pancreatic secretion | Gene.2599 | 765 | PREDICTED: sodium/potassium-transporting ATPase subunit alpha isoform X3 [Tribolium castaneum] |
| map04972 Pancreatic secretion | Gene.5636 | 179 | ras-related protein Rab-11A [Anoplophora glabripennis] |
| map04972 Pancreatic secretion | Gene.901 | 772 | PREDICTED: plasma membrane calcium-transporting ATPase 2 isoform X2 [Tribolium castaneum] |
| map04972 Pancreatic secretion | Gene.901 | 166 | PREDICTED: plasma membrane calcium-transporting ATPase 2 isoform X2 [Tribolium castaneum] |
| map04972 Pancreatic secretion | Gene.901 | 16 | PREDICTED: plasma membrane calcium-transporting ATPase 2 isoform X2 [Tribolium castaneum] |
| map03320 PPAR signaling pathway | Gene.8672 | 302 | "carnitine O-palmitoyltransferase 2, mitochondrial-like [Leptinotarsa decemlineata]" |
| map03320 PPAR signaling pathway | Gene.7892 | 3 | "Polyubiquitin 11, partial [Trichinella nelsoni]" |
| map03320 PPAR signaling pathway | Gene.4302 | 243 | long-chain-fatty-acid--CoA ligase 4 isoform X1 [Agrilus planipennis] |
| map03320 PPAR signaling pathway | Gene.4302 | 416 | long-chain-fatty-acid--CoA ligase 4 isoform X1 [Agrilus planipennis] |
| map03320 PPAR signaling pathway | Gene.4302 | 411 | long-chain-fatty-acid--CoA ligase 4 isoform X1 [Agrilus planipennis] |
| map03320 PPAR signaling pathway | Gene.4302 | 263 | long-chain-fatty-acid--CoA ligase 4 isoform X1 [Agrilus planipennis] |
| map03320 PPAR signaling pathway | Gene.7551 | 170 | PREDICTED: non-specific lipid-transfer protein [Aethina tumida] |
| map03320 PPAR signaling pathway | Gene.7551 | 119 | PREDICTED: non-specific lipid-transfer protein [Aethina tumida] |
| map03320 PPAR signaling pathway | Gene.7551 | 269 | PREDICTED: non-specific lipid-transfer protein [Aethina tumida] |
| map03320 PPAR signaling pathway | Gene.730 | 308 | long-chain-fatty-acid--CoA ligase ACSBG2 [Anoplophora glabripennis] |
| map03320 PPAR signaling pathway | Gene.730 | 161 | long-chain-fatty-acid--CoA ligase ACSBG2 [Anoplophora glabripennis] |
| map03320 PPAR signaling pathway | Gene.730 | 318 | long-chain-fatty-acid--CoA ligase ACSBG2 [Anoplophora glabripennis] |
| map03320 PPAR signaling pathway | Gene.7860 | 245 | PREDICTED: lipid storage droplets surface-binding protein 1 isoform X1 [Aethina tumida] |
| map03320 PPAR signaling pathway | Gene.7860 | 300 | PREDICTED: lipid storage droplets surface-binding protein 1 isoform X1 [Aethina tumida] |
| map03320 PPAR signaling pathway | Gene.4303 | 171 | PREDICTED: LOW QUALITY PROTEIN: long-chain-fatty-acid--CoA ligase 4 [Aethina tumida] |
| map03320 PPAR signaling pathway | Gene.4272 | 220 | PREDICTED: LOW QUALITY PROTEIN: NADP-dependent malic enzyme-like [Aethina tumida] |
| map03320 PPAR signaling pathway | Gene.4272 | 20 | PREDICTED: LOW QUALITY PROTEIN: NADP-dependent malic enzyme-like [Aethina tumida] |
| map03320 PPAR signaling pathway | Gene.7945 | 119 | "fatty acid-binding protein, muscle isoform X2 [Anoplophora glabripennis]" |
| map03320 PPAR signaling pathway | Gene.7945 | 130 | "fatty acid-binding protein, muscle isoform X2 [Anoplophora glabripennis]" |
| map03320 PPAR signaling pathway | Gene.7945 | 87 | "fatty acid-binding protein, muscle isoform X2 [Anoplophora glabripennis]" |
| map03320 PPAR signaling pathway | Gene.7945 | 94 | "fatty acid-binding protein, muscle isoform X2 [Anoplophora glabripennis]" |
| map03320 PPAR signaling pathway | Gene.7945 | 73 | "fatty acid-binding protein, muscle isoform X2 [Anoplophora glabripennis]" |
| map03320 PPAR signaling pathway | Gene.7945 | 144 | "fatty acid-binding protein, muscle isoform X2 [Anoplophora glabripennis]" |
| map03320 PPAR signaling pathway | Gene.7945 | 125 | "fatty acid-binding protein, muscle isoform X2 [Anoplophora glabripennis]" |
| map03320 PPAR signaling pathway | Gene.5903 | 309 | Z9 acyl-CoA desaturase B [Tribolium castaneum] |
| map03320 PPAR signaling pathway | Gene.4404 | 26 | peroxisomal acyl-coenzyme A oxidase 3 isoform X1 [Anoplophora glabripennis] |
| map03320 PPAR signaling pathway | Gene.4271 | 163 | PREDICTED: NADP-dependent malic enzyme isoform X1 [Megachile rotundata] |
| map03320 PPAR signaling pathway | Gene.4271 | 171 | PREDICTED: NADP-dependent malic enzyme isoform X1 [Megachile rotundata] |
| map03320 PPAR signaling pathway | Gene.1745 | 333 | "PREDICTED: probable medium-chain specific acyl-CoA dehydrogenase, mitochondrial [Tribolium castaneum]" |
| map03320 PPAR signaling pathway | Gene.1745 | 63 | "PREDICTED: probable medium-chain specific acyl-CoA dehydrogenase, mitochondrial [Tribolium castaneum]" |
| map03320 PPAR signaling pathway | Gene.1745 | 342 | "PREDICTED: probable medium-chain specific acyl-CoA dehydrogenase, mitochondrial [Tribolium castaneum]" |
| map03320 PPAR signaling pathway | Gene.1745 | 206 | "PREDICTED: probable medium-chain specific acyl-CoA dehydrogenase, mitochondrial [Tribolium castaneum]" |
| map03320 PPAR signaling pathway | Gene.1745 | 273 | "PREDICTED: probable medium-chain specific acyl-CoA dehydrogenase, mitochondrial [Tribolium castaneum]" |
| map03320 PPAR signaling pathway | Gene.1745 | 253 | "PREDICTED: probable medium-chain specific acyl-CoA dehydrogenase, mitochondrial [Tribolium castaneum]" |
| map03320 PPAR signaling pathway | Gene.1745 | 295 | "PREDICTED: probable medium-chain specific acyl-CoA dehydrogenase, mitochondrial [Tribolium castaneum]" |
| map03320 PPAR signaling pathway | Gene.1745 | 211 | "PREDICTED: probable medium-chain specific acyl-CoA dehydrogenase, mitochondrial [Tribolium castaneum]" |
| map03320 PPAR signaling pathway | Gene.1745 | 265 | "PREDICTED: probable medium-chain specific acyl-CoA dehydrogenase, mitochondrial [Tribolium castaneum]" |
| map03320 PPAR signaling pathway | Gene.1745 | 73 | "PREDICTED: probable medium-chain specific acyl-CoA dehydrogenase, mitochondrial [Tribolium castaneum]" |
| map03320 PPAR signaling pathway | Gene.3247 | 93 | PREDICTED: phosphoenolpyruvate carboxykinase [GTP] isoform X1 [Tribolium castaneum] |
| map05200 Pathways in cancer | Gene.8873 | 99 | "PREDICTED: fumarate hydratase, mitochondrial [Tribolium castaneum]" |
| map05200 Pathways in cancer | Gene.8873 | 106 | "PREDICTED: fumarate hydratase, mitochondrial [Tribolium castaneum]" |
| map05200 Pathways in cancer | Gene.8873 | 57 | "PREDICTED: fumarate hydratase, mitochondrial [Tribolium castaneum]" |
| map05200 Pathways in cancer | Gene.8873 | 45 | "PREDICTED: fumarate hydratase, mitochondrial [Tribolium castaneum]" |
| map05200 Pathways in cancer | Gene.8873 | 122 | "PREDICTED: fumarate hydratase, mitochondrial [Tribolium castaneum]" |
| map05200 Pathways in cancer | Gene.8873 | 218 | "PREDICTED: fumarate hydratase, mitochondrial [Tribolium castaneum]" |
| map05200 Pathways in cancer | Gene.7526 | 96 | ras-related protein Rac1 [Anoplophora glabripennis] |
| map05200 Pathways in cancer | Gene.164 | 78 | PREDICTED: guanine nucleotide-binding protein subunit beta-1 [Tribolium castaneum] |
| map05200 Pathways in cancer | Gene.2724 | 2 | PREDICTED: endoplasmin [Tribolium castaneum] |
| map05200 Pathways in cancer | Gene.2724 | 29 | PREDICTED: endoplasmin [Tribolium castaneum] |
| map05200 Pathways in cancer | Gene.5709 | 26 | cAMP-dependent protein kinase catalytic subunit [Agrilus planipennis] |
| map05200 Pathways in cancer | Gene.5709 | 257 | cAMP-dependent protein kinase catalytic subunit [Agrilus planipennis] |
| map05200 Pathways in cancer | Gene.9040 | 57 | PREDICTED: microsomal glutathione S-transferase 1 [Tribolium castaneum] |
| map05200 Pathways in cancer | Gene.9040 | 55 | PREDICTED: microsomal glutathione S-transferase 1 [Tribolium castaneum] |
| map05200 Pathways in cancer | Gene.1845 | 92 | PREDICTED: glycogen synthase kinase-3 beta isoform X10 [Tribolium castaneum] |
| map05200 Pathways in cancer | Gene.1845 | 87 | PREDICTED: glycogen synthase kinase-3 beta isoform X10 [Tribolium castaneum] |
| map05200 Pathways in cancer | Gene.7264 | 643 | "heat shock protein 90, partial [Harmonia axyridis]" |
| map05200 Pathways in cancer | Gene.7264 | 559 | "heat shock protein 90, partial [Harmonia axyridis]" |
| map05200 Pathways in cancer | Gene.7264 | 571 | "heat shock protein 90, partial [Harmonia axyridis]" |
| map05200 Pathways in cancer | Gene.7264 | 67 | "heat shock protein 90, partial [Harmonia axyridis]" |
| map05200 Pathways in cancer | Gene.7264 | 217 | "heat shock protein 90, partial [Harmonia axyridis]" |
| map05200 Pathways in cancer | Gene.7264 | 475 | "heat shock protein 90, partial [Harmonia axyridis]" |
| map05200 Pathways in cancer | Gene.7264 | 341 | "heat shock protein 90, partial [Harmonia axyridis]" |
| map05200 Pathways in cancer | Gene.7264 | 269 | "heat shock protein 90, partial [Harmonia axyridis]" |
| map05200 Pathways in cancer | Gene.7264 | 464 | "heat shock protein 90, partial [Harmonia axyridis]" |
| map05200 Pathways in cancer | Gene.7264 | 212 | "heat shock protein 90, partial [Harmonia axyridis]" |
| map05200 Pathways in cancer | Gene.7264 | 72 | "heat shock protein 90, partial [Harmonia axyridis]" |
| map05200 Pathways in cancer | Gene.7264 | 532 | "heat shock protein 90, partial [Harmonia axyridis]" |
| map05200 Pathways in cancer | Gene.7264 | 444 | "heat shock protein 90, partial [Harmonia axyridis]" |
| map05200 Pathways in cancer | Gene.7264 | 105 | "heat shock protein 90, partial [Harmonia axyridis]" |
| map05200 Pathways in cancer | Gene.7264 | 525 | "heat shock protein 90, partial [Harmonia axyridis]" |
| map05200 Pathways in cancer | Gene.7264 | 553 | "heat shock protein 90, partial [Harmonia axyridis]" |
| map05200 Pathways in cancer | Gene.7264 | 562 | "heat shock protein 90, partial [Harmonia axyridis]" |
| map05200 Pathways in cancer | Gene.7264 | 259 | "heat shock protein 90, partial [Harmonia axyridis]" |
| map05200 Pathways in cancer | Gene.7264 | 280 | "heat shock protein 90, partial [Harmonia axyridis]" |
| map05200 Pathways in cancer | Gene.7264 | 51 | "heat shock protein 90, partial [Harmonia axyridis]" |
| map05200 Pathways in cancer | Gene.7264 | 429 | "heat shock protein 90, partial [Harmonia axyridis]" |
| map05200 Pathways in cancer | Gene.7264 | 601 | "heat shock protein 90, partial [Harmonia axyridis]" |
| map05200 Pathways in cancer | Gene.7264 | 184 | "heat shock protein 90, partial [Harmonia axyridis]" |
| map05200 Pathways in cancer | Gene.7264 | 568 | "heat shock protein 90, partial [Harmonia axyridis]" |
| map05200 Pathways in cancer | Gene.7264 | 393 | "heat shock protein 90, partial [Harmonia axyridis]" |
| map05200 Pathways in cancer | Gene.7264 | 278 | "heat shock protein 90, partial [Harmonia axyridis]" |
| map05200 Pathways in cancer | Gene.7264 | 544 | "heat shock protein 90, partial [Harmonia axyridis]" |
| map05200 Pathways in cancer | Gene.7264 | 432 | "heat shock protein 90, partial [Harmonia axyridis]" |
| map05200 Pathways in cancer | Gene.7264 | 405 | "heat shock protein 90, partial [Harmonia axyridis]" |
| map05200 Pathways in cancer | Gene.7264 | 195 | "heat shock protein 90, partial [Harmonia axyridis]" |
| map05200 Pathways in cancer | Gene.8382 | 77 | GST [Lygus lineolaris] |
| map05200 Pathways in cancer | Gene.8382 | 80 | GST [Lygus lineolaris] |
| map05200 Pathways in cancer | Gene.8382 | 31 | GST [Lygus lineolaris] |
| map05200 Pathways in cancer | Gene.8756 | 84 | uncharacterized protein Dvir_GJ16722 [Drosophila virilis] |
| map05200 Pathways in cancer | Gene.8756 | 10 | uncharacterized protein Dvir_GJ16722 [Drosophila virilis] |
| map05200 Pathways in cancer | Gene.2725 | 81 | PREDICTED: endoplasmin [Tribolium castaneum] |
| map05200 Pathways in cancer | Gene.2725 | 103 | PREDICTED: endoplasmin [Tribolium castaneum] |
| map05200 Pathways in cancer | Gene.2725 | 549 | PREDICTED: endoplasmin [Tribolium castaneum] |
| map05200 Pathways in cancer | Gene.2725 | 554 | PREDICTED: endoplasmin [Tribolium castaneum] |
| map05200 Pathways in cancer | Gene.2725 | 490 | PREDICTED: endoplasmin [Tribolium castaneum] |
| map05200 Pathways in cancer | Gene.7676 | 53 | PREDICTED: guanine nucleotide-binding protein G(q) subunit alpha isoform X1 [Tribolium castaneum] |
| map05200 Pathways in cancer | Gene.5803 | 20 | calcium-binding protein E63-1 [Anoplophora glabripennis] |
| map05200 Pathways in cancer | Gene.425 | 128 | glutathione S-transferase 1-1 [Anoplophora glabripennis] |
| map05200 Pathways in cancer | Gene.425 | 215 | glutathione S-transferase 1-1 [Anoplophora glabripennis] |
| map05200 Pathways in cancer | Gene.425 | 131 | glutathione S-transferase 1-1 [Anoplophora glabripennis] |
| map05200 Pathways in cancer | Gene.425 | 201 | glutathione S-transferase 1-1 [Anoplophora glabripennis] |
| map05200 Pathways in cancer | Gene.425 | 207 | glutathione S-transferase 1-1 [Anoplophora glabripennis] |
| map05200 Pathways in cancer | Gene.425 | 37 | glutathione S-transferase 1-1 [Anoplophora glabripennis] |
| map05200 Pathways in cancer | Gene.425 | 186 | glutathione S-transferase 1-1 [Anoplophora glabripennis] |
| map05200 Pathways in cancer | Gene.298 | 116 | "Csa-calmodulin 3, partial [Cupiennius salei]" |
| map05200 Pathways in cancer | Gene.298 | 95 | "Csa-calmodulin 3, partial [Cupiennius salei]" |
| map05200 Pathways in cancer | Gene.298 | 31 | "Csa-calmodulin 3, partial [Cupiennius salei]" |
| map05200 Pathways in cancer | Gene.298 | 78 | "Csa-calmodulin 3, partial [Cupiennius salei]" |
| map05200 Pathways in cancer | Gene.4842 | 1047 | PREDICTED: laminin subunit gamma-1 isoform X1 [Tribolium castaneum] |
| map04110 Cell cycle | Gene.1845 | 92 | PREDICTED: glycogen synthase kinase-3 beta isoform X10 [Tribolium castaneum] |
| map04110 Cell cycle | Gene.1845 | 87 | PREDICTED: glycogen synthase kinase-3 beta isoform X10 [Tribolium castaneum] |
| map04110 Cell cycle | Gene.8963 | 123 | 14-3-3 protein zeta isoform X2 [Anoplophora glabripennis] |
| map04110 Cell cycle | Gene.8963 | 6 | 14-3-3 protein zeta isoform X2 [Anoplophora glabripennis] |
| map04110 Cell cycle | Gene.8963 | 118 | 14-3-3 protein zeta isoform X2 [Anoplophora glabripennis] |
| map04110 Cell cycle | Gene.8963 | 83 | 14-3-3 protein zeta isoform X2 [Anoplophora glabripennis] |
| map04110 Cell cycle | Gene.8963 | 71 | 14-3-3 protein zeta isoform X2 [Anoplophora glabripennis] |
| map04110 Cell cycle | Gene.8963 | 160 | 14-3-3 protein zeta isoform X2 [Anoplophora glabripennis] |
| map04110 Cell cycle | Gene.8963 | 111 | 14-3-3 protein zeta isoform X2 [Anoplophora glabripennis] |
| map04110 Cell cycle | Gene.8963 | 14 | 14-3-3 protein zeta isoform X2 [Anoplophora glabripennis] |
| map04110 Cell cycle | Gene.8963 | 52 | 14-3-3 protein zeta isoform X2 [Anoplophora glabripennis] |
| map04110 Cell cycle | Gene.8963 | 125 | 14-3-3 protein zeta isoform X2 [Anoplophora glabripennis] |
| map04110 Cell cycle | Gene.8963 | 30 | 14-3-3 protein zeta isoform X2 [Anoplophora glabripennis] |
| map04110 Cell cycle | Gene.8938 | 10 | 14-3-3 epsilon [Tenebrio molitor] |
| map04110 Cell cycle | Gene.8938 | 12 | 14-3-3 epsilon [Tenebrio molitor] |
| map04110 Cell cycle | Gene.8938 | 78 | 14-3-3 epsilon [Tenebrio molitor] |
| map05231 Choline metabolism in cancer | Gene.7526 | 96 | ras-related protein Rac1 [Anoplophora glabripennis] |
| map04666 Fc gamma R-mediated phagocytosis | Gene.8288 | 19 | cofilin/actin-depolymerizing factor homolog [Anoplophora glabripennis] |
| map04666 Fc gamma R-mediated phagocytosis | Gene.8288 | 41 | cofilin/actin-depolymerizing factor homolog [Anoplophora glabripennis] |
| map04666 Fc gamma R-mediated phagocytosis | Gene.7526 | 96 | ras-related protein Rac1 [Anoplophora glabripennis] |
| map05416 Viral myocarditis | Gene.8756 | 84 | uncharacterized protein Dvir_GJ16722 [Drosophila virilis] |
| map05416 Viral myocarditis | Gene.8756 | 10 | uncharacterized protein Dvir_GJ16722 [Drosophila virilis] |
| map05416 Viral myocarditis | Gene.7526 | 96 | ras-related protein Rac1 [Anoplophora glabripennis] |
| map05416 Viral myocarditis | Gene.2357 | 165 | "eukaryotic translation initiation factor 4 gamma 3-like, partial [Asbolus verrucosus]" |
| map05416 Viral myocarditis | Gene.2357 | 739 | "eukaryotic translation initiation factor 4 gamma 3-like, partial [Asbolus verrucosus]" |
| map05416 Viral myocarditis | Gene.172 | 216 | "beta-actin, partial [Cotesia chilonis]" |
| map05416 Viral myocarditis | Gene.172 | 51 | "beta-actin, partial [Cotesia chilonis]" |
| map05416 Viral myocarditis | Gene.172 | 114 | "beta-actin, partial [Cotesia chilonis]" |
| map05416 Viral myocarditis | Gene.172 | 62 | "beta-actin, partial [Cotesia chilonis]" |
| map05416 Viral myocarditis | Gene.4973 | 1173 | "PREDICTED: myosin heavy chain, muscle isoform X18 [Tribolium castaneum]" |
| map05416 Viral myocarditis | Gene.4973 | 1579 | "PREDICTED: myosin heavy chain, muscle isoform X18 [Tribolium castaneum]" |
| map05416 Viral myocarditis | Gene.4973 | 1457 | "PREDICTED: myosin heavy chain, muscle isoform X18 [Tribolium castaneum]" |
| map05416 Viral myocarditis | Gene.4973 | 1332 | "PREDICTED: myosin heavy chain, muscle isoform X18 [Tribolium castaneum]" |
| map05416 Viral myocarditis | Gene.4973 | 907 | "PREDICTED: myosin heavy chain, muscle isoform X18 [Tribolium castaneum]" |
| map05416 Viral myocarditis | Gene.4973 | 1641 | "PREDICTED: myosin heavy chain, muscle isoform X18 [Tribolium castaneum]" |
| map05416 Viral myocarditis | Gene.4973 | 599 | "PREDICTED: myosin heavy chain, muscle isoform X18 [Tribolium castaneum]" |
| map05416 Viral myocarditis | Gene.4973 | 1390 | "PREDICTED: myosin heavy chain, muscle isoform X18 [Tribolium castaneum]" |
| map05416 Viral myocarditis | Gene.4973 | 84 | "PREDICTED: myosin heavy chain, muscle isoform X18 [Tribolium castaneum]" |
| map05416 Viral myocarditis | Gene.4973 | 1005 | "PREDICTED: myosin heavy chain, muscle isoform X18 [Tribolium castaneum]" |
| map05416 Viral myocarditis | Gene.4973 | 1919 | "PREDICTED: myosin heavy chain, muscle isoform X18 [Tribolium castaneum]" |
| map05416 Viral myocarditis | Gene.4973 | 895 | "PREDICTED: myosin heavy chain, muscle isoform X18 [Tribolium castaneum]" |
| map05416 Viral myocarditis | Gene.4973 | 450 | "PREDICTED: myosin heavy chain, muscle isoform X18 [Tribolium castaneum]" |
| map05416 Viral myocarditis | Gene.4973 | 551 | "PREDICTED: myosin heavy chain, muscle isoform X18 [Tribolium castaneum]" |
| map05416 Viral myocarditis | Gene.4973 | 879 | "PREDICTED: myosin heavy chain, muscle isoform X18 [Tribolium castaneum]" |
| map05416 Viral myocarditis | Gene.4973 | 1416 | "PREDICTED: myosin heavy chain, muscle isoform X18 [Tribolium castaneum]" |
| map05416 Viral myocarditis | Gene.4973 | 611 | "PREDICTED: myosin heavy chain, muscle isoform X18 [Tribolium castaneum]" |
| map05416 Viral myocarditis | Gene.4973 | 1109 | "PREDICTED: myosin heavy chain, muscle isoform X18 [Tribolium castaneum]" |
| map05416 Viral myocarditis | Gene.4973 | 721 | "PREDICTED: myosin heavy chain, muscle isoform X18 [Tribolium castaneum]" |
| map05416 Viral myocarditis | Gene.4973 | 1838 | "PREDICTED: myosin heavy chain, muscle isoform X18 [Tribolium castaneum]" |
| map05416 Viral myocarditis | Gene.4973 | 1262 | "PREDICTED: myosin heavy chain, muscle isoform X18 [Tribolium castaneum]" |
| map05416 Viral myocarditis | Gene.4973 | 1444 | "PREDICTED: myosin heavy chain, muscle isoform X18 [Tribolium castaneum]" |
| map05416 Viral myocarditis | Gene.4973 | 1277 | "PREDICTED: myosin heavy chain, muscle isoform X18 [Tribolium castaneum]" |
| map05416 Viral myocarditis | Gene.4973 | 1247 | "PREDICTED: myosin heavy chain, muscle isoform X18 [Tribolium castaneum]" |
| map05416 Viral myocarditis | Gene.4973 | 971 | "PREDICTED: myosin heavy chain, muscle isoform X18 [Tribolium castaneum]" |
| map05416 Viral myocarditis | Gene.4973 | 851 | "PREDICTED: myosin heavy chain, muscle isoform X18 [Tribolium castaneum]" |
| map05416 Viral myocarditis | Gene.4973 | 1316 | "PREDICTED: myosin heavy chain, muscle isoform X18 [Tribolium castaneum]" |
| map05416 Viral myocarditis | Gene.4973 | 1374 | "PREDICTED: myosin heavy chain, muscle isoform X18 [Tribolium castaneum]" |
| map05416 Viral myocarditis | Gene.4973 | 951 | "PREDICTED: myosin heavy chain, muscle isoform X18 [Tribolium castaneum]" |
| map05416 Viral myocarditis | Gene.4973 | 1354 | "PREDICTED: myosin heavy chain, muscle isoform X18 [Tribolium castaneum]" |
| map05416 Viral myocarditis | Gene.4973 | 429 | "PREDICTED: myosin heavy chain, muscle isoform X18 [Tribolium castaneum]" |
| map05416 Viral myocarditis | Gene.4973 | 1103 | "PREDICTED: myosin heavy chain, muscle isoform X18 [Tribolium castaneum]" |
| map05416 Viral myocarditis | Gene.4973 | 1093 | "PREDICTED: myosin heavy chain, muscle isoform X18 [Tribolium castaneum]" |
| map05416 Viral myocarditis | Gene.4973 | 1451 | "PREDICTED: myosin heavy chain, muscle isoform X18 [Tribolium castaneum]" |
| map05416 Viral myocarditis | Gene.4973 | 940 | "PREDICTED: myosin heavy chain, muscle isoform X18 [Tribolium castaneum]" |
| map05416 Viral myocarditis | Gene.4973 | 1791 | "PREDICTED: myosin heavy chain, muscle isoform X18 [Tribolium castaneum]" |
| map05416 Viral myocarditis | Gene.2646 | 99 | beta actin [Polyrhachis vicina] |
| map05416 Viral myocarditis | Gene.2646 | 88 | beta actin [Polyrhachis vicina] |
| map05416 Viral myocarditis | Gene.2646 | 64 | beta actin [Polyrhachis vicina] |
| map05416 Viral myocarditis | Gene.2646 | 101 | beta actin [Polyrhachis vicina] |
| map00604 Glycosphingolipid biosynthesis - ganglio series | Gene.3252 | 101 | chitooligosaccharidolytic beta-N-acetylglucosaminidase isoform X2 [Anoplophora glabripennis] |
| map04270 Vascular smooth muscle contraction | Gene.5709 | 26 | cAMP-dependent protein kinase catalytic subunit [Agrilus planipennis] |
| map04270 Vascular smooth muscle contraction | Gene.5709 | 257 | cAMP-dependent protein kinase catalytic subunit [Agrilus planipennis] |
| map04270 Vascular smooth muscle contraction | Gene.298 | 116 | "Csa-calmodulin 3, partial [Cupiennius salei]" |
| map04270 Vascular smooth muscle contraction | Gene.298 | 95 | "Csa-calmodulin 3, partial [Cupiennius salei]" |
| map04270 Vascular smooth muscle contraction | Gene.298 | 31 | "Csa-calmodulin 3, partial [Cupiennius salei]" |
| map04270 Vascular smooth muscle contraction | Gene.298 | 78 | "Csa-calmodulin 3, partial [Cupiennius salei]" |
| map04270 Vascular smooth muscle contraction | Gene.7676 | 53 | PREDICTED: guanine nucleotide-binding protein G(q) subunit alpha isoform X1 [Tribolium castaneum] |
| map04270 Vascular smooth muscle contraction | Gene.2317 | 49 | PREDICTED: myosin-2 essential light chain isoform X2 [Tribolium castaneum] |
| map04270 Vascular smooth muscle contraction | Gene.2317 | 94 | PREDICTED: myosin-2 essential light chain isoform X2 [Tribolium castaneum] |
| map04270 Vascular smooth muscle contraction | Gene.5803 | 20 | calcium-binding protein E63-1 [Anoplophora glabripennis] |
| map04270 Vascular smooth muscle contraction | Gene.502 | 147 | "acid phosphatase, partial [Cryptolaemus montrouzieri]" |
| map04270 Vascular smooth muscle contraction | Gene.502 | 141 | "acid phosphatase, partial [Cryptolaemus montrouzieri]" |
| map04270 Vascular smooth muscle contraction | Gene.502 | 260 | "acid phosphatase, partial [Cryptolaemus montrouzieri]" |
| map00600 Sphingolipid metabolism | Gene.3500 | 152 | sphingosine-1-phosphate lyase [Asbolus verrucosus] |
| map00600 Sphingolipid metabolism | Gene.3500 | 415 | sphingosine-1-phosphate lyase [Asbolus verrucosus] |
| map04212 Longevity regulating pathway - worm | Gene.8382 | 77 | GST [Lygus lineolaris] |
| map04212 Longevity regulating pathway - worm | Gene.8382 | 80 | GST [Lygus lineolaris] |
| map04212 Longevity regulating pathway - worm | Gene.8382 | 31 | GST [Lygus lineolaris] |
| map04212 Longevity regulating pathway - worm | Gene.8963 | 123 | 14-3-3 protein zeta isoform X2 [Anoplophora glabripennis] |
| map04212 Longevity regulating pathway - worm | Gene.8963 | 6 | 14-3-3 protein zeta isoform X2 [Anoplophora glabripennis] |
| map04212 Longevity regulating pathway - worm | Gene.8963 | 118 | 14-3-3 protein zeta isoform X2 [Anoplophora glabripennis] |
| map04212 Longevity regulating pathway - worm | Gene.8963 | 83 | 14-3-3 protein zeta isoform X2 [Anoplophora glabripennis] |
| map04212 Longevity regulating pathway - worm | Gene.8963 | 71 | 14-3-3 protein zeta isoform X2 [Anoplophora glabripennis] |
| map04212 Longevity regulating pathway - worm | Gene.8963 | 160 | 14-3-3 protein zeta isoform X2 [Anoplophora glabripennis] |
| map04212 Longevity regulating pathway - worm | Gene.8963 | 111 | 14-3-3 protein zeta isoform X2 [Anoplophora glabripennis] |
| map04212 Longevity regulating pathway - worm | Gene.8963 | 14 | 14-3-3 protein zeta isoform X2 [Anoplophora glabripennis] |
| map04212 Longevity regulating pathway - worm | Gene.8963 | 52 | 14-3-3 protein zeta isoform X2 [Anoplophora glabripennis] |
| map04212 Longevity regulating pathway - worm | Gene.8963 | 125 | 14-3-3 protein zeta isoform X2 [Anoplophora glabripennis] |
| map04212 Longevity regulating pathway - worm | Gene.8963 | 30 | 14-3-3 protein zeta isoform X2 [Anoplophora glabripennis] |
| map04212 Longevity regulating pathway - worm | Gene.5965 | 63 | "PREDICTED: superoxide dismutase [Mn] 1, mitochondrial-like [Aethina tumida]" |
| map04212 Longevity regulating pathway - worm | Gene.5965 | 68 | "PREDICTED: superoxide dismutase [Mn] 1, mitochondrial-like [Aethina tumida]" |
| map04212 Longevity regulating pathway - worm | Gene.425 | 128 | glutathione S-transferase 1-1 [Anoplophora glabripennis] |
| map04212 Longevity regulating pathway - worm | Gene.425 | 215 | glutathione S-transferase 1-1 [Anoplophora glabripennis] |
| map04212 Longevity regulating pathway - worm | Gene.425 | 131 | glutathione S-transferase 1-1 [Anoplophora glabripennis] |
| map04212 Longevity regulating pathway - worm | Gene.425 | 201 | glutathione S-transferase 1-1 [Anoplophora glabripennis] |
| map04212 Longevity regulating pathway - worm | Gene.425 | 207 | glutathione S-transferase 1-1 [Anoplophora glabripennis] |
| map04212 Longevity regulating pathway - worm | Gene.425 | 37 | glutathione S-transferase 1-1 [Anoplophora glabripennis] |
| map04212 Longevity regulating pathway - worm | Gene.425 | 186 | glutathione S-transferase 1-1 [Anoplophora glabripennis] |
| map04212 Longevity regulating pathway - worm | Gene.5435 | 35 | putative GABA-A receptor associated protein [Graphocephala atropunctata] |
| map04212 Longevity regulating pathway - worm | Gene.9040 | 57 | PREDICTED: microsomal glutathione S-transferase 1 [Tribolium castaneum] |
| map04212 Longevity regulating pathway - worm | Gene.9040 | 55 | PREDICTED: microsomal glutathione S-transferase 1 [Tribolium castaneum] |
| map04212 Longevity regulating pathway - worm | Gene.5903 | 309 | Z9 acyl-CoA desaturase B [Tribolium castaneum] |
| map04212 Longevity regulating pathway - worm | Gene.316 | 127 | heat shock 70 kDa protein cognate 5-like protein [Epicauta chinensis] |
| map04212 Longevity regulating pathway - worm | Gene.316 | 612 | heat shock 70 kDa protein cognate 5-like protein [Epicauta chinensis] |
| map04212 Longevity regulating pathway - worm | Gene.316 | 412 | heat shock 70 kDa protein cognate 5-like protein [Epicauta chinensis] |
| map04212 Longevity regulating pathway - worm | Gene.316 | 241 | heat shock 70 kDa protein cognate 5-like protein [Epicauta chinensis] |
| map04212 Longevity regulating pathway - worm | Gene.316 | 307 | heat shock 70 kDa protein cognate 5-like protein [Epicauta chinensis] |
| map04212 Longevity regulating pathway - worm | Gene.316 | 607 | heat shock 70 kDa protein cognate 5-like protein [Epicauta chinensis] |
| map04212 Longevity regulating pathway - worm | Gene.316 | 632 | heat shock 70 kDa protein cognate 5-like protein [Epicauta chinensis] |
| map04212 Longevity regulating pathway - worm | Gene.316 | 617 | heat shock 70 kDa protein cognate 5-like protein [Epicauta chinensis] |
| map04212 Longevity regulating pathway - worm | Gene.316 | 677 | heat shock 70 kDa protein cognate 5-like protein [Epicauta chinensis] |
| map04212 Longevity regulating pathway - worm | Gene.316 | 352 | heat shock 70 kDa protein cognate 5-like protein [Epicauta chinensis] |
| map04212 Longevity regulating pathway - worm | Gene.316 | 144 | heat shock 70 kDa protein cognate 5-like protein [Epicauta chinensis] |
| map04212 Longevity regulating pathway - worm | Gene.316 | 378 | heat shock 70 kDa protein cognate 5-like protein [Epicauta chinensis] |
| map04212 Longevity regulating pathway - worm | Gene.316 | 82 | heat shock 70 kDa protein cognate 5-like protein [Epicauta chinensis] |
| map04212 Longevity regulating pathway - worm | Gene.316 | 149 | heat shock 70 kDa protein cognate 5-like protein [Epicauta chinensis] |
| map04212 Longevity regulating pathway - worm | Gene.316 | 182 | heat shock 70 kDa protein cognate 5-like protein [Epicauta chinensis] |
| map04212 Longevity regulating pathway - worm | Gene.316 | 367 | heat shock 70 kDa protein cognate 5-like protein [Epicauta chinensis] |
| map04212 Longevity regulating pathway - worm | Gene.316 | 619 | heat shock 70 kDa protein cognate 5-like protein [Epicauta chinensis] |
| map04212 Longevity regulating pathway - worm | Gene.316 | 140 | heat shock 70 kDa protein cognate 5-like protein [Epicauta chinensis] |
| map04212 Longevity regulating pathway - worm | Gene.2149 | 359 | "heat shock protein, partial [Cryptolaemus montrouzieri]" |
| map04212 Longevity regulating pathway - worm | Gene.2149 | 387 | "heat shock protein, partial [Cryptolaemus montrouzieri]" |
| map04212 Longevity regulating pathway - worm | Gene.2149 | 156 | "heat shock protein, partial [Cryptolaemus montrouzieri]" |
| map04212 Longevity regulating pathway - worm | Gene.2149 | 75 | "heat shock protein, partial [Cryptolaemus montrouzieri]" |
| map04212 Longevity regulating pathway - worm | Gene.2149 | 108 | "heat shock protein, partial [Cryptolaemus montrouzieri]" |
| map04212 Longevity regulating pathway - worm | Gene.2149 | 202 | "heat shock protein, partial [Cryptolaemus montrouzieri]" |
| map04212 Longevity regulating pathway - worm | Gene.2149 | 417 | "heat shock protein, partial [Cryptolaemus montrouzieri]" |
| map04212 Longevity regulating pathway - worm | Gene.2149 | 469 | "heat shock protein, partial [Cryptolaemus montrouzieri]" |
| map04212 Longevity regulating pathway - worm | Gene.2149 | 72 | "heat shock protein, partial [Cryptolaemus montrouzieri]" |
| map04212 Longevity regulating pathway - worm | Gene.2149 | 89 | "heat shock protein, partial [Cryptolaemus montrouzieri]" |
| map04212 Longevity regulating pathway - worm | Gene.2149 | 125 | "heat shock protein, partial [Cryptolaemus montrouzieri]" |
| map04212 Longevity regulating pathway - worm | Gene.2149 | 133 | "heat shock protein, partial [Cryptolaemus montrouzieri]" |
| map04212 Longevity regulating pathway - worm | Gene.5542 | 28 | catalase [Onthophagus taurus] |
| map04212 Longevity regulating pathway - worm | Gene.5542 | 179 | catalase [Onthophagus taurus] |
| map04212 Longevity regulating pathway - worm | Gene.5542 | 325 | catalase [Onthophagus taurus] |
| map04212 Longevity regulating pathway - worm | Gene.5542 | 247 | catalase [Onthophagus taurus] |
| map04212 Longevity regulating pathway - worm | Gene.5542 | 438 | catalase [Onthophagus taurus] |
| map04212 Longevity regulating pathway - worm | Gene.5542 | 48 | catalase [Onthophagus taurus] |
| map04713 Circadian entrainment | Gene.5709 | 26 | cAMP-dependent protein kinase catalytic subunit [Agrilus planipennis] |
| map04713 Circadian entrainment | Gene.5709 | 257 | cAMP-dependent protein kinase catalytic subunit [Agrilus planipennis] |
| map04713 Circadian entrainment | Gene.298 | 116 | "Csa-calmodulin 3, partial [Cupiennius salei]" |
| map04713 Circadian entrainment | Gene.298 | 95 | "Csa-calmodulin 3, partial [Cupiennius salei]" |
| map04713 Circadian entrainment | Gene.298 | 31 | "Csa-calmodulin 3, partial [Cupiennius salei]" |
| map04713 Circadian entrainment | Gene.298 | 78 | "Csa-calmodulin 3, partial [Cupiennius salei]" |
| map04713 Circadian entrainment | Gene.7676 | 53 | PREDICTED: guanine nucleotide-binding protein G(q) subunit alpha isoform X1 [Tribolium castaneum] |
| map04713 Circadian entrainment | Gene.164 | 78 | PREDICTED: guanine nucleotide-binding protein subunit beta-1 [Tribolium castaneum] |
| map04713 Circadian entrainment | Gene.5803 | 20 | calcium-binding protein E63-1 [Anoplophora glabripennis] |
| map05142 Chagas disease (American trypanosomiasis) | Gene.8135 | 359 | PREDICTED: calreticulin [Tribolium castaneum] |
| map05142 Chagas disease (American trypanosomiasis) | Gene.8135 | 203 | PREDICTED: calreticulin [Tribolium castaneum] |
| map05142 Chagas disease (American trypanosomiasis) | Gene.8135 | 206 | PREDICTED: calreticulin [Tribolium castaneum] |
| map05142 Chagas disease (American trypanosomiasis) | Gene.8135 | 61 | PREDICTED: calreticulin [Tribolium castaneum] |
| map05142 Chagas disease (American trypanosomiasis) | Gene.8135 | 108 | PREDICTED: calreticulin [Tribolium castaneum] |
| map05142 Chagas disease (American trypanosomiasis) | Gene.8135 | 52 | PREDICTED: calreticulin [Tribolium castaneum] |
| map05142 Chagas disease (American trypanosomiasis) | Gene.8135 | 354 | PREDICTED: calreticulin [Tribolium castaneum] |
| map05142 Chagas disease (American trypanosomiasis) | Gene.8135 | 37 | PREDICTED: calreticulin [Tribolium castaneum] |
| map05142 Chagas disease (American trypanosomiasis) | Gene.8135 | 45 | PREDICTED: calreticulin [Tribolium castaneum] |
| map05142 Chagas disease (American trypanosomiasis) | Gene.8135 | 275 | PREDICTED: calreticulin [Tribolium castaneum] |
| map05142 Chagas disease (American trypanosomiasis) | Gene.8135 | 31 | PREDICTED: calreticulin [Tribolium castaneum] |
| map05142 Chagas disease (American trypanosomiasis) | Gene.7676 | 53 | PREDICTED: guanine nucleotide-binding protein G(q) subunit alpha isoform X1 [Tribolium castaneum] |
| map05142 Chagas disease (American trypanosomiasis) | Gene.6561 | 544 | "protein phosphatase 2, regulatory subunit A, alpha isoform [Tribolium castaneum]" |
| map05142 Chagas disease (American trypanosomiasis) | Gene.6561 | 268 | "protein phosphatase 2, regulatory subunit A, alpha isoform [Tribolium castaneum]" |
| map04912 GnRH signaling pathway | Gene.5709 | 26 | cAMP-dependent protein kinase catalytic subunit [Agrilus planipennis] |
| map04912 GnRH signaling pathway | Gene.5709 | 257 | cAMP-dependent protein kinase catalytic subunit [Agrilus planipennis] |
| map04912 GnRH signaling pathway | Gene.298 | 116 | "Csa-calmodulin 3, partial [Cupiennius salei]" |
| map04912 GnRH signaling pathway | Gene.298 | 95 | "Csa-calmodulin 3, partial [Cupiennius salei]" |
| map04912 GnRH signaling pathway | Gene.298 | 31 | "Csa-calmodulin 3, partial [Cupiennius salei]" |
| map04912 GnRH signaling pathway | Gene.298 | 78 | "Csa-calmodulin 3, partial [Cupiennius salei]" |
| map04912 GnRH signaling pathway | Gene.7676 | 53 | PREDICTED: guanine nucleotide-binding protein G(q) subunit alpha isoform X1 [Tribolium castaneum] |
| map04912 GnRH signaling pathway | Gene.5803 | 20 | calcium-binding protein E63-1 [Anoplophora glabripennis] |
| map00220 Arginine biosynthesis | Gene.2656 | 56 | "PREDICTED: aspartate aminotransferase, cytoplasmic [Tribolium castaneum]" |
| map00220 Arginine biosynthesis | Gene.2656 | 141 | "PREDICTED: aspartate aminotransferase, cytoplasmic [Tribolium castaneum]" |
| map00220 Arginine biosynthesis | Gene.1489 | 401 | "aspartate aminotransferase, mitochondrial [Asbolus verrucosus]" |
| map00220 Arginine biosynthesis | Gene.1489 | 87 | "aspartate aminotransferase, mitochondrial [Asbolus verrucosus]" |
| map00220 Arginine biosynthesis | Gene.1489 | 189 | "aspartate aminotransferase, mitochondrial [Asbolus verrucosus]" |
| map00220 Arginine biosynthesis | Gene.1489 | 334 | "aspartate aminotransferase, mitochondrial [Asbolus verrucosus]" |
| map00220 Arginine biosynthesis | Gene.1489 | 174 | "aspartate aminotransferase, mitochondrial [Asbolus verrucosus]" |
| map00220 Arginine biosynthesis | Gene.1489 | 393 | "aspartate aminotransferase, mitochondrial [Asbolus verrucosus]" |
| map00220 Arginine biosynthesis | Gene.1489 | 332 | "aspartate aminotransferase, mitochondrial [Asbolus verrucosus]" |
| map00220 Arginine biosynthesis | Gene.1489 | 91 | "aspartate aminotransferase, mitochondrial [Asbolus verrucosus]" |
| map00220 Arginine biosynthesis | Gene.1489 | 295 | "aspartate aminotransferase, mitochondrial [Asbolus verrucosus]" |
| map00220 Arginine biosynthesis | Gene.3721 | 212 | PREDICTED: glutamine synthetase-like [Aethina tumida] |
| map00220 Arginine biosynthesis | Gene.3721 | 39 | PREDICTED: glutamine synthetase-like [Aethina tumida] |
| map00220 Arginine biosynthesis | Gene.3721 | 208 | PREDICTED: glutamine synthetase-like [Aethina tumida] |
| map00220 Arginine biosynthesis | Gene.3721 | 227 | PREDICTED: glutamine synthetase-like [Aethina tumida] |
| map00220 Arginine biosynthesis | Gene.3721 | 195 | PREDICTED: glutamine synthetase-like [Aethina tumida] |
| map00220 Arginine biosynthesis | Gene.500 | 434 | alanine aminotransferase 1 [Anoplophora glabripennis] |
| map00220 Arginine biosynthesis | Gene.500 | 545 | alanine aminotransferase 1 [Anoplophora glabripennis] |
| map00220 Arginine biosynthesis | Gene.500 | 540 | alanine aminotransferase 1 [Anoplophora glabripennis] |
| map00220 Arginine biosynthesis | Gene.500 | 285 | alanine aminotransferase 1 [Anoplophora glabripennis] |
| map00220 Arginine biosynthesis | Gene.500 | 107 | alanine aminotransferase 1 [Anoplophora glabripennis] |
| map00220 Arginine biosynthesis | Gene.500 | 316 | alanine aminotransferase 1 [Anoplophora glabripennis] |
| map00220 Arginine biosynthesis | Gene.2236 | 516 | "Glutamate dehydrogenase, mitochondrial-like Protein [Tribolium castaneum]" |
| map00220 Arginine biosynthesis | Gene.2236 | 412 | "Glutamate dehydrogenase, mitochondrial-like Protein [Tribolium castaneum]" |
| map00220 Arginine biosynthesis | Gene.2236 | 492 | "Glutamate dehydrogenase, mitochondrial-like Protein [Tribolium castaneum]" |
| map00220 Arginine biosynthesis | Gene.2236 | 457 | "Glutamate dehydrogenase, mitochondrial-like Protein [Tribolium castaneum]" |
| map00220 Arginine biosynthesis | Gene.2236 | 87 | "Glutamate dehydrogenase, mitochondrial-like Protein [Tribolium castaneum]" |
| map00220 Arginine biosynthesis | Gene.2236 | 186 | "Glutamate dehydrogenase, mitochondrial-like Protein [Tribolium castaneum]" |
| map00220 Arginine biosynthesis | Gene.2236 | 500 | "Glutamate dehydrogenase, mitochondrial-like Protein [Tribolium castaneum]" |
| map00220 Arginine biosynthesis | Gene.2236 | 372 | "Glutamate dehydrogenase, mitochondrial-like Protein [Tribolium castaneum]" |
| map00220 Arginine biosynthesis | Gene.2236 | 477 | "Glutamate dehydrogenase, mitochondrial-like Protein [Tribolium castaneum]" |
| map00220 Arginine biosynthesis | Gene.2236 | 195 | "Glutamate dehydrogenase, mitochondrial-like Protein [Tribolium castaneum]" |
| map00220 Arginine biosynthesis | Gene.2236 | 82 | "Glutamate dehydrogenase, mitochondrial-like Protein [Tribolium castaneum]" |
| map05322 Systemic lupus erythematosus | Gene.8758 | 197 | PREDICTED: la protein homolog [Tribolium castaneum] |
| map05322 Systemic lupus erythematosus | Gene.8758 | 180 | PREDICTED: la protein homolog [Tribolium castaneum] |
| map05322 Systemic lupus erythematosus | Gene.8758 | 317 | PREDICTED: la protein homolog [Tribolium castaneum] |
| map05322 Systemic lupus erythematosus | Gene.8758 | 204 | PREDICTED: la protein homolog [Tribolium castaneum] |
| map05322 Systemic lupus erythematosus | Gene.8758 | 232 | PREDICTED: la protein homolog [Tribolium castaneum] |
| map05322 Systemic lupus erythematosus | Gene.8758 | 224 | PREDICTED: la protein homolog [Tribolium castaneum] |
| map05322 Systemic lupus erythematosus | Gene.8758 | 313 | PREDICTED: la protein homolog [Tribolium castaneum] |
| map05322 Systemic lupus erythematosus | Gene.6221 | 123 | PREDICTED: histone H3.3-like [Takifugu rubripes] |
| map05322 Systemic lupus erythematosus | Gene.6221 | 80 | PREDICTED: histone H3.3-like [Takifugu rubripes] |
| map05322 Systemic lupus erythematosus | Gene.2958 | 401 | alpha actinin [Coleomegilla maculata] |
| map05322 Systemic lupus erythematosus | Gene.2958 | 763 | alpha actinin [Coleomegilla maculata] |
| map05322 Systemic lupus erythematosus | Gene.1218 | 27 | histone H2B-like [Zootermopsis nevadensis] |
| map05322 Systemic lupus erythematosus | Gene.1218 | 123 | histone H2B-like [Zootermopsis nevadensis] |
| map05322 Systemic lupus erythematosus | Gene.1218 | 127 | histone H2B-like [Zootermopsis nevadensis] |
| map05322 Systemic lupus erythematosus | Gene.1218 | 53 | histone H2B-like [Zootermopsis nevadensis] |
| map05322 Systemic lupus erythematosus | Gene.1218 | 41 | histone H2B-like [Zootermopsis nevadensis] |
| map05322 Systemic lupus erythematosus | Gene.1218 | 115 | histone H2B-like [Zootermopsis nevadensis] |
| map05322 Systemic lupus erythematosus | Gene.1218 | 92 | histone H2B-like [Zootermopsis nevadensis] |
| map05322 Systemic lupus erythematosus | Gene.1218 | 31 | histone H2B-like [Zootermopsis nevadensis] |
| map04750 Inflammatory mediator regulation of TRP channels | Gene.5709 | 26 | cAMP-dependent protein kinase catalytic subunit [Agrilus planipennis] |
| map04750 Inflammatory mediator regulation of TRP channels | Gene.5709 | 257 | cAMP-dependent protein kinase catalytic subunit [Agrilus planipennis] |
| map04750 Inflammatory mediator regulation of TRP channels | Gene.298 | 116 | "Csa-calmodulin 3, partial [Cupiennius salei]" |
| map04750 Inflammatory mediator regulation of TRP channels | Gene.298 | 95 | "Csa-calmodulin 3, partial [Cupiennius salei]" |
| map04750 Inflammatory mediator regulation of TRP channels | Gene.298 | 31 | "Csa-calmodulin 3, partial [Cupiennius salei]" |
| map04750 Inflammatory mediator regulation of TRP channels | Gene.298 | 78 | "Csa-calmodulin 3, partial [Cupiennius salei]" |
| map04750 Inflammatory mediator regulation of TRP channels | Gene.7676 | 53 | PREDICTED: guanine nucleotide-binding protein G(q) subunit alpha isoform X1 [Tribolium castaneum] |
| map04750 Inflammatory mediator regulation of TRP channels | Gene.5803 | 20 | calcium-binding protein E63-1 [Anoplophora glabripennis] |
| map04750 Inflammatory mediator regulation of TRP channels | Gene.502 | 147 | "acid phosphatase, partial [Cryptolaemus montrouzieri]" |
| map04750 Inflammatory mediator regulation of TRP channels | Gene.502 | 141 | "acid phosphatase, partial [Cryptolaemus montrouzieri]" |
| map04750 Inflammatory mediator regulation of TRP channels | Gene.502 | 260 | "acid phosphatase, partial [Cryptolaemus montrouzieri]" |
| map01523 Antifolate resistance | Gene.5527 | 139 | dihydrofolate reductase [Asbolus verrucosus] |
| map01523 Antifolate resistance | Gene.5015 | 46 | multidrug resistance-associated protein 4 [Anoplophora glabripennis] |
| map01523 Antifolate resistance | Gene.5015 | 916 | multidrug resistance-associated protein 4 [Anoplophora glabripennis] |
| map01523 Antifolate resistance | Gene.5015 | 1141 | multidrug resistance-associated protein 4 [Anoplophora glabripennis] |
| map01523 Antifolate resistance | Gene.7326 | 464 | "serine hydroxymethyltransferase, cytosolic isoform X1 [Anoplophora glabripennis]" |
| map01523 Antifolate resistance | Gene.7326 | 475 | "serine hydroxymethyltransferase, cytosolic isoform X1 [Anoplophora glabripennis]" |
| map01523 Antifolate resistance | Gene.261 | 149 | PREDICTED: bifunctional purine biosynthesis protein PURH [Tribolium castaneum] |
| map01523 Antifolate resistance | Gene.261 | 501 | PREDICTED: bifunctional purine biosynthesis protein PURH [Tribolium castaneum] |
| map01523 Antifolate resistance | Gene.261 | 461 | PREDICTED: bifunctional purine biosynthesis protein PURH [Tribolium castaneum] |
| map01523 Antifolate resistance | Gene.261 | 89 | PREDICTED: bifunctional purine biosynthesis protein PURH [Tribolium castaneum] |
| map01523 Antifolate resistance | Gene.261 | 66 | PREDICTED: bifunctional purine biosynthesis protein PURH [Tribolium castaneum] |
| map01523 Antifolate resistance | Gene.261 | 266 | PREDICTED: bifunctional purine biosynthesis protein PURH [Tribolium castaneum] |
| map01523 Antifolate resistance | Gene.261 | 477 | PREDICTED: bifunctional purine biosynthesis protein PURH [Tribolium castaneum] |
| map01523 Antifolate resistance | Gene.524 | 528 | PREDICTED: trifunctional purine biosynthetic protein adenosine-3 [Tribolium castaneum] |
| map01523 Antifolate resistance | Gene.524 | 19 | PREDICTED: trifunctional purine biosynthetic protein adenosine-3 [Tribolium castaneum] |
| map00730 Thiamine metabolism | Gene.8621 | 139 | low molecular weight phosphotyrosine protein phosphatase 1-like [Leptinotarsa decemlineata] |
| map00730 Thiamine metabolism | Gene.6894 | 67 | PREDICTED: adenylate kinase [Tribolium castaneum] |
| map00730 Thiamine metabolism | Gene.6894 | 232 | PREDICTED: adenylate kinase [Tribolium castaneum] |
| map00730 Thiamine metabolism | Gene.6894 | 188 | PREDICTED: adenylate kinase [Tribolium castaneum] |
| map00730 Thiamine metabolism | Gene.6894 | 121 | PREDICTED: adenylate kinase [Tribolium castaneum] |
| map00730 Thiamine metabolism | Gene.8351 | 97 | "probable cysteine desulfurase, mitochondrial [Anoplophora glabripennis]" |
| map00730 Thiamine metabolism | Gene.752 | 132 | thiamin pyrophosphokinase 1 [Anoplophora glabripennis] |
| map00562 Inositol phosphate metabolism | Gene.196 | 25 | PREDICTED: triosephosphate isomerase isoform X1 [Aethina tumida] |
| map00562 Inositol phosphate metabolism | Gene.196 | 197 | PREDICTED: triosephosphate isomerase isoform X1 [Aethina tumida] |
| map00562 Inositol phosphate metabolism | Gene.196 | 189 | PREDICTED: triosephosphate isomerase isoform X1 [Aethina tumida] |
| map00562 Inositol phosphate metabolism | Gene.196 | 67 | PREDICTED: triosephosphate isomerase isoform X1 [Aethina tumida] |
| map00562 Inositol phosphate metabolism | Gene.196 | 217 | PREDICTED: triosephosphate isomerase isoform X1 [Aethina tumida] |
| map00562 Inositol phosphate metabolism | Gene.196 | 83 | PREDICTED: triosephosphate isomerase isoform X1 [Aethina tumida] |
| map00562 Inositol phosphate metabolism | Gene.196 | 70 | PREDICTED: triosephosphate isomerase isoform X1 [Aethina tumida] |
| map00562 Inositol phosphate metabolism | Gene.196 | 118 | PREDICTED: triosephosphate isomerase isoform X1 [Aethina tumida] |
| map00562 Inositol phosphate metabolism | Gene.196 | 12 | PREDICTED: triosephosphate isomerase isoform X1 [Aethina tumida] |
| map00562 Inositol phosphate metabolism | Gene.196 | 173 | PREDICTED: triosephosphate isomerase isoform X1 [Aethina tumida] |
| map00562 Inositol phosphate metabolism | Gene.3076 | 302 | PREDICTED: inositol-3-phosphate synthase [Tribolium castaneum] |
| map00562 Inositol phosphate metabolism | Gene.3875 | 350 | "PREDICTED: probable methylmalonate-semialdehyde dehydrogenase [acylating], mitochondrial [Aethina tumida]" |
| map00562 Inositol phosphate metabolism | Gene.3875 | 241 | "PREDICTED: probable methylmalonate-semialdehyde dehydrogenase [acylating], mitochondrial [Aethina tumida]" |
| map00562 Inositol phosphate metabolism | Gene.3875 | 253 | "PREDICTED: probable methylmalonate-semialdehyde dehydrogenase [acylating], mitochondrial [Aethina tumida]" |
| map00562 Inositol phosphate metabolism | Gene.1012 | 145 | inositol monophosphatase 1 isoform X2 [Leptinotarsa decemlineata] |
| map00562 Inositol phosphate metabolism | Gene.1012 | 138 | inositol monophosphatase 1 isoform X2 [Leptinotarsa decemlineata] |
| map00330 Arginine and proline metabolism | Gene.2656 | 56 | "PREDICTED: aspartate aminotransferase, cytoplasmic [Tribolium castaneum]" |
| map00330 Arginine and proline metabolism | Gene.2656 | 141 | "PREDICTED: aspartate aminotransferase, cytoplasmic [Tribolium castaneum]" |
| map00330 Arginine and proline metabolism | Gene.1489 | 401 | "aspartate aminotransferase, mitochondrial [Asbolus verrucosus]" |
| map00330 Arginine and proline metabolism | Gene.1489 | 87 | "aspartate aminotransferase, mitochondrial [Asbolus verrucosus]" |
| map00330 Arginine and proline metabolism | Gene.1489 | 189 | "aspartate aminotransferase, mitochondrial [Asbolus verrucosus]" |
| map00330 Arginine and proline metabolism | Gene.1489 | 334 | "aspartate aminotransferase, mitochondrial [Asbolus verrucosus]" |
| map00330 Arginine and proline metabolism | Gene.1489 | 174 | "aspartate aminotransferase, mitochondrial [Asbolus verrucosus]" |
| map00330 Arginine and proline metabolism | Gene.1489 | 393 | "aspartate aminotransferase, mitochondrial [Asbolus verrucosus]" |
| map00330 Arginine and proline metabolism | Gene.1489 | 332 | "aspartate aminotransferase, mitochondrial [Asbolus verrucosus]" |
| map00330 Arginine and proline metabolism | Gene.1489 | 91 | "aspartate aminotransferase, mitochondrial [Asbolus verrucosus]" |
| map00330 Arginine and proline metabolism | Gene.1489 | 295 | "aspartate aminotransferase, mitochondrial [Asbolus verrucosus]" |
| map00330 Arginine and proline metabolism | Gene.3947 | 476 | PREDICTED: delta-1-pyrroline-5-carboxylate synthase [Tribolium castaneum] |
| map00330 Arginine and proline metabolism | Gene.3947 | 207 | PREDICTED: delta-1-pyrroline-5-carboxylate synthase [Tribolium castaneum] |
| map00330 Arginine and proline metabolism | Gene.3947 | 330 | PREDICTED: delta-1-pyrroline-5-carboxylate synthase [Tribolium castaneum] |
| map00330 Arginine and proline metabolism | Gene.3947 | 607 | PREDICTED: delta-1-pyrroline-5-carboxylate synthase [Tribolium castaneum] |
| map00330 Arginine and proline metabolism | Gene.3947 | 425 | PREDICTED: delta-1-pyrroline-5-carboxylate synthase [Tribolium castaneum] |
| map00330 Arginine and proline metabolism | Gene.3947 | 531 | PREDICTED: delta-1-pyrroline-5-carboxylate synthase [Tribolium castaneum] |
| map00330 Arginine and proline metabolism | Gene.3947 | 353 | PREDICTED: delta-1-pyrroline-5-carboxylate synthase [Tribolium castaneum] |
| map00330 Arginine and proline metabolism | Gene.3947 | 432 | PREDICTED: delta-1-pyrroline-5-carboxylate synthase [Tribolium castaneum] |
| map00330 Arginine and proline metabolism | Gene.3947 | 321 | PREDICTED: delta-1-pyrroline-5-carboxylate synthase [Tribolium castaneum] |
| map00330 Arginine and proline metabolism | Gene.3947 | 318 | PREDICTED: delta-1-pyrroline-5-carboxylate synthase [Tribolium castaneum] |
| map00330 Arginine and proline metabolism | Gene.2533 | 286 | PREDICTED: putative aldehyde dehydrogenase family 7 member A1 homolog [Aethina tumida] |
| map00330 Arginine and proline metabolism | Gene.2533 | 400 | PREDICTED: putative aldehyde dehydrogenase family 7 member A1 homolog [Aethina tumida] |
| map00330 Arginine and proline metabolism | Gene.2533 | 41 | PREDICTED: putative aldehyde dehydrogenase family 7 member A1 homolog [Aethina tumida] |
| map00330 Arginine and proline metabolism | Gene.2533 | 56 | PREDICTED: putative aldehyde dehydrogenase family 7 member A1 homolog [Aethina tumida] |
| map00330 Arginine and proline metabolism | Gene.2533 | 382 | PREDICTED: putative aldehyde dehydrogenase family 7 member A1 homolog [Aethina tumida] |
| map00330 Arginine and proline metabolism | Gene.2533 | 73 | PREDICTED: putative aldehyde dehydrogenase family 7 member A1 homolog [Aethina tumida] |
| map00330 Arginine and proline metabolism | Gene.2533 | 62 | PREDICTED: putative aldehyde dehydrogenase family 7 member A1 homolog [Aethina tumida] |
| map00330 Arginine and proline metabolism | Gene.2533 | 65 | PREDICTED: putative aldehyde dehydrogenase family 7 member A1 homolog [Aethina tumida] |
| map00330 Arginine and proline metabolism | Gene.8481 | 237 | PREDICTED: arginine kinase isoform X1 [Tribolium castaneum] |
| map00330 Arginine and proline metabolism | Gene.8481 | 210 | PREDICTED: arginine kinase isoform X1 [Tribolium castaneum] |
| map00330 Arginine and proline metabolism | Gene.8481 | 310 | PREDICTED: arginine kinase isoform X1 [Tribolium castaneum] |
| map00330 Arginine and proline metabolism | Gene.8481 | 38 | PREDICTED: arginine kinase isoform X1 [Tribolium castaneum] |
| map00330 Arginine and proline metabolism | Gene.8481 | 201 | PREDICTED: arginine kinase isoform X1 [Tribolium castaneum] |
| map00330 Arginine and proline metabolism | Gene.8481 | 349 | PREDICTED: arginine kinase isoform X1 [Tribolium castaneum] |
| map00330 Arginine and proline metabolism | Gene.8481 | 45 | PREDICTED: arginine kinase isoform X1 [Tribolium castaneum] |
| map00330 Arginine and proline metabolism | Gene.8481 | 372 | PREDICTED: arginine kinase isoform X1 [Tribolium castaneum] |
| map00330 Arginine and proline metabolism | Gene.8481 | 196 | PREDICTED: arginine kinase isoform X1 [Tribolium castaneum] |
| map00330 Arginine and proline metabolism | Gene.7938 | 42 | PREDICTED: spermine synthase isoform X1 [Tribolium castaneum] |
| map00330 Arginine and proline metabolism | Gene.7938 | 302 | PREDICTED: spermine synthase isoform X1 [Tribolium castaneum] |
| map00330 Arginine and proline metabolism | Gene.7938 | 148 | PREDICTED: spermine synthase isoform X1 [Tribolium castaneum] |
| map00330 Arginine and proline metabolism | Gene.7938 | 305 | PREDICTED: spermine synthase isoform X1 [Tribolium castaneum] |
| map00330 Arginine and proline metabolism | Gene.2841 | 88 | "ornithine aminotransferase, mitochondrial-like [Leptinotarsa decemlineata]" |
| map00330 Arginine and proline metabolism | Gene.2841 | 115 | "ornithine aminotransferase, mitochondrial-like [Leptinotarsa decemlineata]" |
| map00330 Arginine and proline metabolism | Gene.8989 | 179 | "PREDICTED: aldehyde dehydrogenase, dimeric NADP-preferring-like [Aethina tumida]" |
| map00330 Arginine and proline metabolism | Gene.8989 | 258 | "PREDICTED: aldehyde dehydrogenase, dimeric NADP-preferring-like [Aethina tumida]" |
| map00330 Arginine and proline metabolism | Gene.8989 | 438 | "PREDICTED: aldehyde dehydrogenase, dimeric NADP-preferring-like [Aethina tumida]" |
| map00330 Arginine and proline metabolism | Gene.468 | 427 | "retinal dehydrogenase 1, partial [Asbolus verrucosus]" |
| map00330 Arginine and proline metabolism | Gene.468 | 27 | "retinal dehydrogenase 1, partial [Asbolus verrucosus]" |
| map00330 Arginine and proline metabolism | Gene.468 | 131 | "retinal dehydrogenase 1, partial [Asbolus verrucosus]" |
| map00330 Arginine and proline metabolism | Gene.468 | 20 | "retinal dehydrogenase 1, partial [Asbolus verrucosus]" |
| map00330 Arginine and proline metabolism | Gene.8727 | 296 | "PREDICTED: proline dehydrogenase 1, mitochondrial isoform X2 [Dendroctonus ponderosae]" |
| map00330 Arginine and proline metabolism | Gene.8727 | 343 | "PREDICTED: proline dehydrogenase 1, mitochondrial isoform X2 [Dendroctonus ponderosae]" |
| map00330 Arginine and proline metabolism | Gene.8424 | 34 | PREDICTED: NAD/NADP-dependent betaine aldehyde dehydrogenase [Tribolium castaneum] |
| map00330 Arginine and proline metabolism | Gene.8424 | 532 | PREDICTED: NAD/NADP-dependent betaine aldehyde dehydrogenase [Tribolium castaneum] |
| map00330 Arginine and proline metabolism | Gene.182 | 52 | cytosolic non-specific dipeptidase [Anoplophora glabripennis] |
| map00330 Arginine and proline metabolism | Gene.182 | 127 | cytosolic non-specific dipeptidase [Anoplophora glabripennis] |
| map00330 Arginine and proline metabolism | Gene.182 | 453 | cytosolic non-specific dipeptidase [Anoplophora glabripennis] |
| map00330 Arginine and proline metabolism | Gene.182 | 40 | cytosolic non-specific dipeptidase [Anoplophora glabripennis] |
| map00330 Arginine and proline metabolism | Gene.182 | 342 | cytosolic non-specific dipeptidase [Anoplophora glabripennis] |
| map00330 Arginine and proline metabolism | Gene.182 | 338 | cytosolic non-specific dipeptidase [Anoplophora glabripennis] |
| map00330 Arginine and proline metabolism | Gene.182 | 288 | cytosolic non-specific dipeptidase [Anoplophora glabripennis] |
| map00330 Arginine and proline metabolism | Gene.269 | 241 | PREDICTED: cytosol aminopeptidase isoform X2 [Tribolium castaneum] |
| map00330 Arginine and proline metabolism | Gene.269 | 58 | PREDICTED: cytosol aminopeptidase isoform X2 [Tribolium castaneum] |
| map00330 Arginine and proline metabolism | Gene.2749 | 84 | "PREDICTED: aldehyde dehydrogenase, mitochondrial [Tribolium castaneum]" |
| map00330 Arginine and proline metabolism | Gene.2749 | 370 | "PREDICTED: aldehyde dehydrogenase, mitochondrial [Tribolium castaneum]" |
| map00330 Arginine and proline metabolism | Gene.2749 | 446 | "PREDICTED: aldehyde dehydrogenase, mitochondrial [Tribolium castaneum]" |
| map00330 Arginine and proline metabolism | Gene.2749 | 151 | "PREDICTED: aldehyde dehydrogenase, mitochondrial [Tribolium castaneum]" |
| map00330 Arginine and proline metabolism | Gene.2749 | 140 | "PREDICTED: aldehyde dehydrogenase, mitochondrial [Tribolium castaneum]" |
| map00330 Arginine and proline metabolism | Gene.2749 | 423 | "PREDICTED: aldehyde dehydrogenase, mitochondrial [Tribolium castaneum]" |
| map00330 Arginine and proline metabolism | Gene.9057 | 232 | PREDICTED: pyrroline-5-carboxylate reductase [Tribolium castaneum] |
| map00330 Arginine and proline metabolism | Gene.9057 | 144 | PREDICTED: pyrroline-5-carboxylate reductase [Tribolium castaneum] |
| map00330 Arginine and proline metabolism | Gene.9057 | 151 | PREDICTED: pyrroline-5-carboxylate reductase [Tribolium castaneum] |
| map00330 Arginine and proline metabolism | Gene.1071 | 104 | "PREDICTED: delta-1-pyrroline-5-carboxylate dehydrogenase, mitochondrial [Tribolium castaneum]" |
| map00330 Arginine and proline metabolism | Gene.1071 | 370 | "PREDICTED: delta-1-pyrroline-5-carboxylate dehydrogenase, mitochondrial [Tribolium castaneum]" |
| map00330 Arginine and proline metabolism | Gene.1071 | 183 | "PREDICTED: delta-1-pyrroline-5-carboxylate dehydrogenase, mitochondrial [Tribolium castaneum]" |
| map00330 Arginine and proline metabolism | Gene.1071 | 79 | "PREDICTED: delta-1-pyrroline-5-carboxylate dehydrogenase, mitochondrial [Tribolium castaneum]" |
| map00330 Arginine and proline metabolism | Gene.1071 | 47 | "PREDICTED: delta-1-pyrroline-5-carboxylate dehydrogenase, mitochondrial [Tribolium castaneum]" |
| map00330 Arginine and proline metabolism | Gene.1071 | 109 | "PREDICTED: delta-1-pyrroline-5-carboxylate dehydrogenase, mitochondrial [Tribolium castaneum]" |
| map00330 Arginine and proline metabolism | Gene.1071 | 98 | "PREDICTED: delta-1-pyrroline-5-carboxylate dehydrogenase, mitochondrial [Tribolium castaneum]" |
| map00330 Arginine and proline metabolism | Gene.1071 | 76 | "PREDICTED: delta-1-pyrroline-5-carboxylate dehydrogenase, mitochondrial [Tribolium castaneum]" |
| map00330 Arginine and proline metabolism | Gene.3948 | 34 | PREDICTED: delta-1-pyrroline-5-carboxylate synthase [Nicrophorus vespilloides] |
| map00330 Arginine and proline metabolism | Gene.3948 | 31 | PREDICTED: delta-1-pyrroline-5-carboxylate synthase [Nicrophorus vespilloides] |
| map04216 Ferroptosis | Gene.1333 | 59 | soma ferritin [Asbolus verrucosus] |
| map04216 Ferroptosis | Gene.1333 | 74 | soma ferritin [Asbolus verrucosus] |
| map04216 Ferroptosis | Gene.1333 | 155 | soma ferritin [Asbolus verrucosus] |
| map04216 Ferroptosis | Gene.1333 | 55 | soma ferritin [Asbolus verrucosus] |
| map04216 Ferroptosis | Gene.229 | 159 | PREDICTED: probable phospholipid hydroperoxide glutathione peroxidase [Tribolium castaneum] |
| map04216 Ferroptosis | Gene.229 | 156 | PREDICTED: probable phospholipid hydroperoxide glutathione peroxidase [Tribolium castaneum] |
| map04216 Ferroptosis | Gene.229 | 198 | PREDICTED: probable phospholipid hydroperoxide glutathione peroxidase [Tribolium castaneum] |
| map04216 Ferroptosis | Gene.229 | 183 | PREDICTED: probable phospholipid hydroperoxide glutathione peroxidase [Tribolium castaneum] |
| map04216 Ferroptosis | Gene.229 | 19 | PREDICTED: probable phospholipid hydroperoxide glutathione peroxidase [Tribolium castaneum] |
| map04216 Ferroptosis | Gene.229 | 126 | PREDICTED: probable phospholipid hydroperoxide glutathione peroxidase [Tribolium castaneum] |
| map04216 Ferroptosis | Gene.4302 | 243 | long-chain-fatty-acid--CoA ligase 4 isoform X1 [Agrilus planipennis] |
| map04216 Ferroptosis | Gene.4302 | 416 | long-chain-fatty-acid--CoA ligase 4 isoform X1 [Agrilus planipennis] |
| map04216 Ferroptosis | Gene.4302 | 411 | long-chain-fatty-acid--CoA ligase 4 isoform X1 [Agrilus planipennis] |
| map04216 Ferroptosis | Gene.4302 | 263 | long-chain-fatty-acid--CoA ligase 4 isoform X1 [Agrilus planipennis] |
| map04216 Ferroptosis | Gene.8407 | 27 | PREDICTED: voltage-dependent anion-selective channel isoform X1 [Tribolium castaneum] |
| map04216 Ferroptosis | Gene.8407 | 11 | PREDICTED: voltage-dependent anion-selective channel isoform X1 [Tribolium castaneum] |
| map04216 Ferroptosis | Gene.8407 | 60 | PREDICTED: voltage-dependent anion-selective channel isoform X1 [Tribolium castaneum] |
| map04216 Ferroptosis | Gene.8407 | 64 | PREDICTED: voltage-dependent anion-selective channel isoform X1 [Tribolium castaneum] |
| map04216 Ferroptosis | Gene.4303 | 171 | PREDICTED: LOW QUALITY PROTEIN: long-chain-fatty-acid--CoA ligase 4 [Aethina tumida] |
| map04216 Ferroptosis | Gene.331 | 185 | PREDICTED: ferritin subunit [Aethina tumida] |
| map04216 Ferroptosis | Gene.331 | 206 | PREDICTED: ferritin subunit [Aethina tumida] |
| map04120 Ubiquitin mediated proteolysis | Gene.2517 | 133 | "effete, isoform A [Drosophila melanogaster]" |
| map04120 Ubiquitin mediated proteolysis | Gene.5801 | 337 | SUMO-activating enzyme subunit 2 [Anoplophora glabripennis] |
| map04120 Ubiquitin mediated proteolysis | Gene.5801 | 531 | SUMO-activating enzyme subunit 2 [Anoplophora glabripennis] |
| map04657 IL-17 signaling pathway | Gene.7085 | 22 | PREDICTED: serine/arginine-rich splicing factor 1A [Tribolium castaneum] |
| map04657 IL-17 signaling pathway | Gene.7085 | 33 | PREDICTED: serine/arginine-rich splicing factor 1A [Tribolium castaneum] |
| map04657 IL-17 signaling pathway | Gene.1845 | 92 | PREDICTED: glycogen synthase kinase-3 beta isoform X10 [Tribolium castaneum] |
| map04657 IL-17 signaling pathway | Gene.1845 | 87 | PREDICTED: glycogen synthase kinase-3 beta isoform X10 [Tribolium castaneum] |
| map04657 IL-17 signaling pathway | Gene.2725 | 81 | PREDICTED: endoplasmin [Tribolium castaneum] |
| map04657 IL-17 signaling pathway | Gene.2725 | 103 | PREDICTED: endoplasmin [Tribolium castaneum] |
| map04657 IL-17 signaling pathway | Gene.2725 | 549 | PREDICTED: endoplasmin [Tribolium castaneum] |
| map04657 IL-17 signaling pathway | Gene.2725 | 554 | PREDICTED: endoplasmin [Tribolium castaneum] |
| map04657 IL-17 signaling pathway | Gene.2725 | 490 | PREDICTED: endoplasmin [Tribolium castaneum] |
| map04657 IL-17 signaling pathway | Gene.7264 | 643 | "heat shock protein 90, partial [Harmonia axyridis]" |
| map04657 IL-17 signaling pathway | Gene.7264 | 559 | "heat shock protein 90, partial [Harmonia axyridis]" |
| map04657 IL-17 signaling pathway | Gene.7264 | 571 | "heat shock protein 90, partial [Harmonia axyridis]" |
| map04657 IL-17 signaling pathway | Gene.7264 | 67 | "heat shock protein 90, partial [Harmonia axyridis]" |
| map04657 IL-17 signaling pathway | Gene.7264 | 217 | "heat shock protein 90, partial [Harmonia axyridis]" |
| map04657 IL-17 signaling pathway | Gene.7264 | 475 | "heat shock protein 90, partial [Harmonia axyridis]" |
| map04657 IL-17 signaling pathway | Gene.7264 | 341 | "heat shock protein 90, partial [Harmonia axyridis]" |
| map04657 IL-17 signaling pathway | Gene.7264 | 269 | "heat shock protein 90, partial [Harmonia axyridis]" |
| map04657 IL-17 signaling pathway | Gene.7264 | 464 | "heat shock protein 90, partial [Harmonia axyridis]" |
| map04657 IL-17 signaling pathway | Gene.7264 | 212 | "heat shock protein 90, partial [Harmonia axyridis]" |
| map04657 IL-17 signaling pathway | Gene.7264 | 72 | "heat shock protein 90, partial [Harmonia axyridis]" |
| map04657 IL-17 signaling pathway | Gene.7264 | 532 | "heat shock protein 90, partial [Harmonia axyridis]" |
| map04657 IL-17 signaling pathway | Gene.7264 | 444 | "heat shock protein 90, partial [Harmonia axyridis]" |
| map04657 IL-17 signaling pathway | Gene.7264 | 105 | "heat shock protein 90, partial [Harmonia axyridis]" |
| map04657 IL-17 signaling pathway | Gene.7264 | 525 | "heat shock protein 90, partial [Harmonia axyridis]" |
| map04657 IL-17 signaling pathway | Gene.7264 | 553 | "heat shock protein 90, partial [Harmonia axyridis]" |
| map04657 IL-17 signaling pathway | Gene.7264 | 562 | "heat shock protein 90, partial [Harmonia axyridis]" |
| map04657 IL-17 signaling pathway | Gene.7264 | 259 | "heat shock protein 90, partial [Harmonia axyridis]" |
| map04657 IL-17 signaling pathway | Gene.7264 | 280 | "heat shock protein 90, partial [Harmonia axyridis]" |
| map04657 IL-17 signaling pathway | Gene.7264 | 51 | "heat shock protein 90, partial [Harmonia axyridis]" |
| map04657 IL-17 signaling pathway | Gene.7264 | 429 | "heat shock protein 90, partial [Harmonia axyridis]" |
| map04657 IL-17 signaling pathway | Gene.7264 | 601 | "heat shock protein 90, partial [Harmonia axyridis]" |
| map04657 IL-17 signaling pathway | Gene.7264 | 184 | "heat shock protein 90, partial [Harmonia axyridis]" |
| map04657 IL-17 signaling pathway | Gene.7264 | 568 | "heat shock protein 90, partial [Harmonia axyridis]" |
| map04657 IL-17 signaling pathway | Gene.7264 | 393 | "heat shock protein 90, partial [Harmonia axyridis]" |
| map04657 IL-17 signaling pathway | Gene.7264 | 278 | "heat shock protein 90, partial [Harmonia axyridis]" |
| map04657 IL-17 signaling pathway | Gene.7264 | 544 | "heat shock protein 90, partial [Harmonia axyridis]" |
| map04657 IL-17 signaling pathway | Gene.7264 | 432 | "heat shock protein 90, partial [Harmonia axyridis]" |
| map04657 IL-17 signaling pathway | Gene.7264 | 405 | "heat shock protein 90, partial [Harmonia axyridis]" |
| map04657 IL-17 signaling pathway | Gene.7264 | 195 | "heat shock protein 90, partial [Harmonia axyridis]" |
| map04657 IL-17 signaling pathway | Gene.2724 | 2 | PREDICTED: endoplasmin [Tribolium castaneum] |
| map04657 IL-17 signaling pathway | Gene.2724 | 29 | PREDICTED: endoplasmin [Tribolium castaneum] |
| map05211 Renal cell carcinoma | Gene.8873 | 99 | "PREDICTED: fumarate hydratase, mitochondrial [Tribolium castaneum]" |
| map05211 Renal cell carcinoma | Gene.8873 | 106 | "PREDICTED: fumarate hydratase, mitochondrial [Tribolium castaneum]" |
| map05211 Renal cell carcinoma | Gene.8873 | 57 | "PREDICTED: fumarate hydratase, mitochondrial [Tribolium castaneum]" |
| map05211 Renal cell carcinoma | Gene.8873 | 45 | "PREDICTED: fumarate hydratase, mitochondrial [Tribolium castaneum]" |
| map05211 Renal cell carcinoma | Gene.8873 | 122 | "PREDICTED: fumarate hydratase, mitochondrial [Tribolium castaneum]" |
| map05211 Renal cell carcinoma | Gene.8873 | 218 | "PREDICTED: fumarate hydratase, mitochondrial [Tribolium castaneum]" |
| map05211 Renal cell carcinoma | Gene.7526 | 96 | ras-related protein Rac1 [Anoplophora glabripennis] |
| map00270 Cysteine and methionine metabolism | Gene.8167 | 149 | "malate dehydrogenase, cytoplasmic [Leptinotarsa decemlineata]" |
| map00270 Cysteine and methionine metabolism | Gene.8167 | 317 | "malate dehydrogenase, cytoplasmic [Leptinotarsa decemlineata]" |
| map00270 Cysteine and methionine metabolism | Gene.8167 | 265 | "malate dehydrogenase, cytoplasmic [Leptinotarsa decemlineata]" |
| map00270 Cysteine and methionine metabolism | Gene.8167 | 118 | "malate dehydrogenase, cytoplasmic [Leptinotarsa decemlineata]" |
| map00270 Cysteine and methionine metabolism | Gene.8167 | 259 | "malate dehydrogenase, cytoplasmic [Leptinotarsa decemlineata]" |
| map00270 Cysteine and methionine metabolism | Gene.8167 | 229 | "malate dehydrogenase, cytoplasmic [Leptinotarsa decemlineata]" |
| map00270 Cysteine and methionine metabolism | Gene.8167 | 214 | "malate dehydrogenase, cytoplasmic [Leptinotarsa decemlineata]" |
| map00270 Cysteine and methionine metabolism | Gene.8167 | 110 | "malate dehydrogenase, cytoplasmic [Leptinotarsa decemlineata]" |
| map00270 Cysteine and methionine metabolism | Gene.2656 | 56 | "PREDICTED: aspartate aminotransferase, cytoplasmic [Tribolium castaneum]" |
| map00270 Cysteine and methionine metabolism | Gene.2656 | 141 | "PREDICTED: aspartate aminotransferase, cytoplasmic [Tribolium castaneum]" |
| map00270 Cysteine and methionine metabolism | Gene.8586 | 68 | PREDICTED: putative cystathionine gamma-lyase 2 [Aethina tumida] |
| map00270 Cysteine and methionine metabolism | Gene.1489 | 401 | "aspartate aminotransferase, mitochondrial [Asbolus verrucosus]" |
| map00270 Cysteine and methionine metabolism | Gene.1489 | 87 | "aspartate aminotransferase, mitochondrial [Asbolus verrucosus]" |
| map00270 Cysteine and methionine metabolism | Gene.1489 | 189 | "aspartate aminotransferase, mitochondrial [Asbolus verrucosus]" |
| map00270 Cysteine and methionine metabolism | Gene.1489 | 334 | "aspartate aminotransferase, mitochondrial [Asbolus verrucosus]" |
| map00270 Cysteine and methionine metabolism | Gene.1489 | 174 | "aspartate aminotransferase, mitochondrial [Asbolus verrucosus]" |
| map00270 Cysteine and methionine metabolism | Gene.1489 | 393 | "aspartate aminotransferase, mitochondrial [Asbolus verrucosus]" |
| map00270 Cysteine and methionine metabolism | Gene.1489 | 332 | "aspartate aminotransferase, mitochondrial [Asbolus verrucosus]" |
| map00270 Cysteine and methionine metabolism | Gene.1489 | 91 | "aspartate aminotransferase, mitochondrial [Asbolus verrucosus]" |
| map00270 Cysteine and methionine metabolism | Gene.1489 | 295 | "aspartate aminotransferase, mitochondrial [Asbolus verrucosus]" |
| map00270 Cysteine and methionine metabolism | Gene.7734 | 408 | PREDICTED: LOW QUALITY PROTEIN: adenosylhomocysteinase [Aethina tumida] |
| map00270 Cysteine and methionine metabolism | Gene.7734 | 188 | PREDICTED: LOW QUALITY PROTEIN: adenosylhomocysteinase [Aethina tumida] |
| map00270 Cysteine and methionine metabolism | Gene.7734 | 388 | PREDICTED: LOW QUALITY PROTEIN: adenosylhomocysteinase [Aethina tumida] |
| map00270 Cysteine and methionine metabolism | Gene.6692 | 59 | "D-3-phosphoglycerate dehydrogenase, partial [Asbolus verrucosus]" |
| map00270 Cysteine and methionine metabolism | Gene.6692 | 182 | "D-3-phosphoglycerate dehydrogenase, partial [Asbolus verrucosus]" |
| map00270 Cysteine and methionine metabolism | Gene.6692 | 43 | "D-3-phosphoglycerate dehydrogenase, partial [Asbolus verrucosus]" |
| map00270 Cysteine and methionine metabolism | Gene.6692 | 55 | "D-3-phosphoglycerate dehydrogenase, partial [Asbolus verrucosus]" |
| map00270 Cysteine and methionine metabolism | Gene.3511 | 158 | cystathionine gamma-lyase-like [Anoplophora glabripennis] |
| map00270 Cysteine and methionine metabolism | Gene.3511 | 57 | cystathionine gamma-lyase-like [Anoplophora glabripennis] |
| map00270 Cysteine and methionine metabolism | Gene.3511 | 82 | cystathionine gamma-lyase-like [Anoplophora glabripennis] |
| map00270 Cysteine and methionine metabolism | Gene.960 | 51 | probable phosphoserine aminotransferase [Anoplophora glabripennis] |
| map00270 Cysteine and methionine metabolism | Gene.7938 | 42 | PREDICTED: spermine synthase isoform X1 [Tribolium castaneum] |
| map00270 Cysteine and methionine metabolism | Gene.7938 | 302 | PREDICTED: spermine synthase isoform X1 [Tribolium castaneum] |
| map00270 Cysteine and methionine metabolism | Gene.7938 | 148 | PREDICTED: spermine synthase isoform X1 [Tribolium castaneum] |
| map00270 Cysteine and methionine metabolism | Gene.7938 | 305 | PREDICTED: spermine synthase isoform X1 [Tribolium castaneum] |
| map00270 Cysteine and methionine metabolism | Gene.6958 | 236 | PREDICTED: S-methyl-5'-thioadenosine phosphorylase isoform X2 [Nicrophorus vespilloides] |
| map00270 Cysteine and methionine metabolism | Gene.6969 | 8 | kynurenine--oxoglutarate transaminase 3 [Anoplophora glabripennis] |
| map00270 Cysteine and methionine metabolism | Gene.6969 | 154 | kynurenine--oxoglutarate transaminase 3 [Anoplophora glabripennis] |
| map00270 Cysteine and methionine metabolism | Gene.6674 | 238 | "PREDICTED: malate dehydrogenase, mitochondrial [Tribolium castaneum]" |
| map00270 Cysteine and methionine metabolism | Gene.6674 | 90 | "PREDICTED: malate dehydrogenase, mitochondrial [Tribolium castaneum]" |
| map00270 Cysteine and methionine metabolism | Gene.6674 | 323 | "PREDICTED: malate dehydrogenase, mitochondrial [Tribolium castaneum]" |
| map00270 Cysteine and methionine metabolism | Gene.6674 | 184 | "PREDICTED: malate dehydrogenase, mitochondrial [Tribolium castaneum]" |
| map00270 Cysteine and methionine metabolism | Gene.6674 | 163 | "PREDICTED: malate dehydrogenase, mitochondrial [Tribolium castaneum]" |
| map00270 Cysteine and methionine metabolism | Gene.6674 | 77 | "PREDICTED: malate dehydrogenase, mitochondrial [Tribolium castaneum]" |
| map00270 Cysteine and methionine metabolism | Gene.6674 | 125 | "PREDICTED: malate dehydrogenase, mitochondrial [Tribolium castaneum]" |
| map00270 Cysteine and methionine metabolism | Gene.6674 | 335 | "PREDICTED: malate dehydrogenase, mitochondrial [Tribolium castaneum]" |
| map00270 Cysteine and methionine metabolism | Gene.6674 | 86 | "PREDICTED: malate dehydrogenase, mitochondrial [Tribolium castaneum]" |
| map04361 Axon regeneration | Gene.5709 | 26 | cAMP-dependent protein kinase catalytic subunit [Agrilus planipennis] |
| map04361 Axon regeneration | Gene.5709 | 257 | cAMP-dependent protein kinase catalytic subunit [Agrilus planipennis] |
| map04361 Axon regeneration | Gene.7676 | 53 | PREDICTED: guanine nucleotide-binding protein G(q) subunit alpha isoform X1 [Tribolium castaneum] |
| map04361 Axon regeneration | Gene.7526 | 96 | ras-related protein Rac1 [Anoplophora glabripennis] |
| map05222 Small cell lung cancer | Gene.8756 | 84 | uncharacterized protein Dvir_GJ16722 [Drosophila virilis] |
| map05222 Small cell lung cancer | Gene.8756 | 10 | uncharacterized protein Dvir_GJ16722 [Drosophila virilis] |
| map05222 Small cell lung cancer | Gene.4842 | 1047 | PREDICTED: laminin subunit gamma-1 isoform X1 [Tribolium castaneum] |
| map05224 Breast cancer | Gene.1845 | 92 | PREDICTED: glycogen synthase kinase-3 beta isoform X10 [Tribolium castaneum] |
| map05224 Breast cancer | Gene.1845 | 87 | PREDICTED: glycogen synthase kinase-3 beta isoform X10 [Tribolium castaneum] |
| map00500 Starch and sucrose metabolism | Gene.3793 | 109 | "maltase 2, partial [Asbolus verrucosus]" |
| map00500 Starch and sucrose metabolism | Gene.1161 | 74 | PREDICTED: UTP--glucose-1-phosphate uridylyltransferase isoform X1 [Tribolium castaneum] |
| map00500 Starch and sucrose metabolism | Gene.1161 | 71 | PREDICTED: UTP--glucose-1-phosphate uridylyltransferase isoform X1 [Tribolium castaneum] |
| map00500 Starch and sucrose metabolism | Gene.1161 | 424 | PREDICTED: UTP--glucose-1-phosphate uridylyltransferase isoform X1 [Tribolium castaneum] |
| map00500 Starch and sucrose metabolism | Gene.1161 | 305 | PREDICTED: UTP--glucose-1-phosphate uridylyltransferase isoform X1 [Tribolium castaneum] |
| map00500 Starch and sucrose metabolism | Gene.1161 | 294 | PREDICTED: UTP--glucose-1-phosphate uridylyltransferase isoform X1 [Tribolium castaneum] |
| map00500 Starch and sucrose metabolism | Gene.1161 | 434 | PREDICTED: UTP--glucose-1-phosphate uridylyltransferase isoform X1 [Tribolium castaneum] |
| map00500 Starch and sucrose metabolism | Gene.1161 | 312 | PREDICTED: UTP--glucose-1-phosphate uridylyltransferase isoform X1 [Tribolium castaneum] |
| map00500 Starch and sucrose metabolism | Gene.1161 | 19 | PREDICTED: UTP--glucose-1-phosphate uridylyltransferase isoform X1 [Tribolium castaneum] |
| map00500 Starch and sucrose metabolism | Gene.1161 | 284 | PREDICTED: UTP--glucose-1-phosphate uridylyltransferase isoform X1 [Tribolium castaneum] |
| map00500 Starch and sucrose metabolism | Gene.8318 | 197 | PGM PMM I domain containing protein [Asbolus verrucosus] |
| map00500 Starch and sucrose metabolism | Gene.8318 | 466 | PGM PMM I domain containing protein [Asbolus verrucosus] |
| map00500 Starch and sucrose metabolism | Gene.8318 | 204 | PGM PMM I domain containing protein [Asbolus verrucosus] |
| map00500 Starch and sucrose metabolism | Gene.8318 | 486 | PGM PMM I domain containing protein [Asbolus verrucosus] |
| map00500 Starch and sucrose metabolism | Gene.8318 | 489 | PGM PMM I domain containing protein [Asbolus verrucosus] |
| map00500 Starch and sucrose metabolism | Gene.8318 | 9 | PGM PMM I domain containing protein [Asbolus verrucosus] |
| map00500 Starch and sucrose metabolism | Gene.8318 | 348 | PGM PMM I domain containing protein [Asbolus verrucosus] |
| map00500 Starch and sucrose metabolism | Gene.8318 | 421 | PGM PMM I domain containing protein [Asbolus verrucosus] |
| map00500 Starch and sucrose metabolism | Gene.8318 | 352 | PGM PMM I domain containing protein [Asbolus verrucosus] |
| map00500 Starch and sucrose metabolism | Gene.8318 | 278 | PGM PMM I domain containing protein [Asbolus verrucosus] |
| map00500 Starch and sucrose metabolism | Gene.8318 | 151 | PGM PMM I domain containing protein [Asbolus verrucosus] |
| map00500 Starch and sucrose metabolism | Gene.8318 | 459 | PGM PMM I domain containing protein [Asbolus verrucosus] |
| map00500 Starch and sucrose metabolism | Gene.8318 | 449 | PGM PMM I domain containing protein [Asbolus verrucosus] |
| map00500 Starch and sucrose metabolism | Gene.1361 | 702 | PREDICTED: glycogenin-1 isoform X3 [Aethina tumida] |
| map00500 Starch and sucrose metabolism | Gene.4419 | 291 | glycogen phosphorylase [Harmonia axyridis] |
| map00500 Starch and sucrose metabolism | Gene.4419 | 177 | glycogen phosphorylase [Harmonia axyridis] |
| map00500 Starch and sucrose metabolism | Gene.4419 | 10 | glycogen phosphorylase [Harmonia axyridis] |
| map00500 Starch and sucrose metabolism | Gene.4419 | 29 | glycogen phosphorylase [Harmonia axyridis] |
| map00500 Starch and sucrose metabolism | Gene.4419 | 249 | glycogen phosphorylase [Harmonia axyridis] |
| map00500 Starch and sucrose metabolism | Gene.4419 | 78 | glycogen phosphorylase [Harmonia axyridis] |
| map00500 Starch and sucrose metabolism | Gene.4366 | 47 | phosphoglucomutase-2 [Leptinotarsa decemlineata] |
| map00500 Starch and sucrose metabolism | Gene.1533 | 280 | PREDICTED: hexokinase type 2 isoform X2 [Tribolium castaneum] |
| map00500 Starch and sucrose metabolism | Gene.1533 | 73 | PREDICTED: hexokinase type 2 isoform X2 [Tribolium castaneum] |
| map00500 Starch and sucrose metabolism | Gene.3208 | 28 | phosphoglucose isomerase [Colias eurytheme] |
| map00500 Starch and sucrose metabolism | Gene.3208 | 462 | phosphoglucose isomerase [Colias eurytheme] |
| map00500 Starch and sucrose metabolism | Gene.3208 | 41 | phosphoglucose isomerase [Colias eurytheme] |
| map00500 Starch and sucrose metabolism | Gene.8998 | 226 | "RecName: Full=Alpha-amylase; AltName: Full=1,4-alpha-D-glucan glucanohydrolase" |
| map00500 Starch and sucrose metabolism | Gene.8998 | 322 | "RecName: Full=Alpha-amylase; AltName: Full=1,4-alpha-D-glucan glucanohydrolase" |
| map00500 Starch and sucrose metabolism | Gene.8017 | 315 | glycogen synthase [Harmonia axyridis] |
| map00500 Starch and sucrose metabolism | Gene.8017 | 298 | glycogen synthase [Harmonia axyridis] |
| map00500 Starch and sucrose metabolism | Gene.8017 | 247 | glycogen synthase [Harmonia axyridis] |
| map00500 Starch and sucrose metabolism | Gene.4417 | 92 | glycogen phosphorylase [Harmonia axyridis] |
| map00500 Starch and sucrose metabolism | Gene.4417 | 205 | glycogen phosphorylase [Harmonia axyridis] |
| map00500 Starch and sucrose metabolism | Gene.4417 | 120 | glycogen phosphorylase [Harmonia axyridis] |
| map00500 Starch and sucrose metabolism | Gene.4417 | 134 | glycogen phosphorylase [Harmonia axyridis] |
| map00500 Starch and sucrose metabolism | Gene.4417 | 44 | glycogen phosphorylase [Harmonia axyridis] |
| map00500 Starch and sucrose metabolism | Gene.4417 | 288 | glycogen phosphorylase [Harmonia axyridis] |
| map00500 Starch and sucrose metabolism | Gene.4417 | 113 | glycogen phosphorylase [Harmonia axyridis] |
| map00500 Starch and sucrose metabolism | Gene.1365 | 453 | PREDICTED: maltase 2 isoform X3 [Tribolium castaneum] |
| map00500 Starch and sucrose metabolism | Gene.665 | 572 | "PREDICTED: 1,4-alpha-glucan-branching enzyme [Aethina tumida]" |
| map00500 Starch and sucrose metabolism | Gene.665 | 166 | "PREDICTED: 1,4-alpha-glucan-branching enzyme [Aethina tumida]" |
| map00500 Starch and sucrose metabolism | Gene.665 | 631 | "PREDICTED: 1,4-alpha-glucan-branching enzyme [Aethina tumida]" |
| map00500 Starch and sucrose metabolism | Gene.665 | 71 | "PREDICTED: 1,4-alpha-glucan-branching enzyme [Aethina tumida]" |
| map00500 Starch and sucrose metabolism | Gene.665 | 570 | "PREDICTED: 1,4-alpha-glucan-branching enzyme [Aethina tumida]" |
| map00500 Starch and sucrose metabolism | Gene.665 | 105 | "PREDICTED: 1,4-alpha-glucan-branching enzyme [Aethina tumida]" |
| map00500 Starch and sucrose metabolism | Gene.665 | 606 | "PREDICTED: 1,4-alpha-glucan-branching enzyme [Aethina tumida]" |
| map00500 Starch and sucrose metabolism | Gene.665 | 143 | "PREDICTED: 1,4-alpha-glucan-branching enzyme [Aethina tumida]" |
| map04014 Ras signaling pathway | Gene.5709 | 26 | cAMP-dependent protein kinase catalytic subunit [Agrilus planipennis] |
| map04014 Ras signaling pathway | Gene.5709 | 257 | cAMP-dependent protein kinase catalytic subunit [Agrilus planipennis] |
| map04014 Ras signaling pathway | Gene.298 | 116 | "Csa-calmodulin 3, partial [Cupiennius salei]" |
| map04014 Ras signaling pathway | Gene.298 | 95 | "Csa-calmodulin 3, partial [Cupiennius salei]" |
| map04014 Ras signaling pathway | Gene.298 | 31 | "Csa-calmodulin 3, partial [Cupiennius salei]" |
| map04014 Ras signaling pathway | Gene.298 | 78 | "Csa-calmodulin 3, partial [Cupiennius salei]" |
| map04014 Ras signaling pathway | Gene.2348 | 116 | ras-related protein Rab-5B isoform X1 [Leptinotarsa decemlineata] |
| map04014 Ras signaling pathway | Gene.7526 | 96 | ras-related protein Rac1 [Anoplophora glabripennis] |
| map04014 Ras signaling pathway | Gene.164 | 78 | PREDICTED: guanine nucleotide-binding protein subunit beta-1 [Tribolium castaneum] |
| map04014 Ras signaling pathway | Gene.5803 | 20 | calcium-binding protein E63-1 [Anoplophora glabripennis] |
| map00051 Fructose and mannose metabolism | Gene.1633 | 147 | PREDICTED: fructose-bisphosphate aldolase [Tribolium castaneum] |
| map00051 Fructose and mannose metabolism | Gene.1633 | 42 | PREDICTED: fructose-bisphosphate aldolase [Tribolium castaneum] |
| map00051 Fructose and mannose metabolism | Gene.1633 | 28 | PREDICTED: fructose-bisphosphate aldolase [Tribolium castaneum] |
| map00051 Fructose and mannose metabolism | Gene.3223 | 72 | Aldose reductase-like Protein [Tribolium castaneum] |
| map00051 Fructose and mannose metabolism | Gene.3223 | 264 | Aldose reductase-like Protein [Tribolium castaneum] |
| map00051 Fructose and mannose metabolism | Gene.3223 | 202 | Aldose reductase-like Protein [Tribolium castaneum] |
| map00051 Fructose and mannose metabolism | Gene.3223 | 167 | Aldose reductase-like Protein [Tribolium castaneum] |
| map00051 Fructose and mannose metabolism | Gene.2563 | 97 | PREDICTED: aldose reductase [Tribolium castaneum] |
| map00051 Fructose and mannose metabolism | Gene.1533 | 280 | PREDICTED: hexokinase type 2 isoform X2 [Tribolium castaneum] |
| map00051 Fructose and mannose metabolism | Gene.1533 | 73 | PREDICTED: hexokinase type 2 isoform X2 [Tribolium castaneum] |
| map00051 Fructose and mannose metabolism | Gene.2124 | 330 | PREDICTED: sorbitol dehydrogenase [Tribolium castaneum] |
| map00051 Fructose and mannose metabolism | Gene.2124 | 83 | PREDICTED: sorbitol dehydrogenase [Tribolium castaneum] |
| map00051 Fructose and mannose metabolism | Gene.2124 | 210 | PREDICTED: sorbitol dehydrogenase [Tribolium castaneum] |
| map00051 Fructose and mannose metabolism | Gene.2124 | 322 | PREDICTED: sorbitol dehydrogenase [Tribolium castaneum] |
| map00051 Fructose and mannose metabolism | Gene.2124 | 316 | PREDICTED: sorbitol dehydrogenase [Tribolium castaneum] |
| map00051 Fructose and mannose metabolism | Gene.2124 | 324 | PREDICTED: sorbitol dehydrogenase [Tribolium castaneum] |
| map00051 Fructose and mannose metabolism | Gene.2124 | 344 | PREDICTED: sorbitol dehydrogenase [Tribolium castaneum] |
| map00051 Fructose and mannose metabolism | Gene.2124 | 219 | PREDICTED: sorbitol dehydrogenase [Tribolium castaneum] |
| map00051 Fructose and mannose metabolism | Gene.2124 | 351 | PREDICTED: sorbitol dehydrogenase [Tribolium castaneum] |
| map00051 Fructose and mannose metabolism | Gene.196 | 25 | PREDICTED: triosephosphate isomerase isoform X1 [Aethina tumida] |
| map00051 Fructose and mannose metabolism | Gene.196 | 197 | PREDICTED: triosephosphate isomerase isoform X1 [Aethina tumida] |
| map00051 Fructose and mannose metabolism | Gene.196 | 189 | PREDICTED: triosephosphate isomerase isoform X1 [Aethina tumida] |
| map00051 Fructose and mannose metabolism | Gene.196 | 67 | PREDICTED: triosephosphate isomerase isoform X1 [Aethina tumida] |
| map00051 Fructose and mannose metabolism | Gene.196 | 217 | PREDICTED: triosephosphate isomerase isoform X1 [Aethina tumida] |
| map00051 Fructose and mannose metabolism | Gene.196 | 83 | PREDICTED: triosephosphate isomerase isoform X1 [Aethina tumida] |
| map00051 Fructose and mannose metabolism | Gene.196 | 70 | PREDICTED: triosephosphate isomerase isoform X1 [Aethina tumida] |
| map00051 Fructose and mannose metabolism | Gene.196 | 118 | PREDICTED: triosephosphate isomerase isoform X1 [Aethina tumida] |
| map00051 Fructose and mannose metabolism | Gene.196 | 12 | PREDICTED: triosephosphate isomerase isoform X1 [Aethina tumida] |
| map00051 Fructose and mannose metabolism | Gene.196 | 173 | PREDICTED: triosephosphate isomerase isoform X1 [Aethina tumida] |
| map00051 Fructose and mannose metabolism | Gene.7644 | 70 | PREDICTED: aldose reductase [Tribolium castaneum] |
| map00051 Fructose and mannose metabolism | Gene.7644 | 151 | PREDICTED: aldose reductase [Tribolium castaneum] |
| map00051 Fructose and mannose metabolism | Gene.7644 | 61 | PREDICTED: aldose reductase [Tribolium castaneum] |
| map00051 Fructose and mannose metabolism | Gene.1399 | 285 | PREDICTED: GDP-L-fucose synthase-like [Aethina tumida] |
| map04020 Calcium signaling pathway | Gene.7676 | 53 | PREDICTED: guanine nucleotide-binding protein G(q) subunit alpha isoform X1 [Tribolium castaneum] |
| map04020 Calcium signaling pathway | Gene.4352 | 128 | PREDICTED: calcium-transporting ATPase sarcoplasmic/endoplasmic reticulum type isoform X1 [Tribolium castaneum] |
| map04020 Calcium signaling pathway | Gene.4352 | 514 | PREDICTED: calcium-transporting ATPase sarcoplasmic/endoplasmic reticulum type isoform X1 [Tribolium castaneum] |
| map04020 Calcium signaling pathway | Gene.4352 | 30 | PREDICTED: calcium-transporting ATPase sarcoplasmic/endoplasmic reticulum type isoform X1 [Tribolium castaneum] |
| map04020 Calcium signaling pathway | Gene.6805 | 205 | peptidyl-prolyl cis-trans isomerase [Leptinotarsa decemlineata] |
| map04020 Calcium signaling pathway | Gene.6805 | 130 | peptidyl-prolyl cis-trans isomerase [Leptinotarsa decemlineata] |
| map04020 Calcium signaling pathway | Gene.6805 | 85 | peptidyl-prolyl cis-trans isomerase [Leptinotarsa decemlineata] |
| map04020 Calcium signaling pathway | Gene.6805 | 194 | peptidyl-prolyl cis-trans isomerase [Leptinotarsa decemlineata] |
| map04020 Calcium signaling pathway | Gene.5803 | 20 | calcium-binding protein E63-1 [Anoplophora glabripennis] |
| map04020 Calcium signaling pathway | Gene.901 | 772 | PREDICTED: plasma membrane calcium-transporting ATPase 2 isoform X2 [Tribolium castaneum] |
| map04020 Calcium signaling pathway | Gene.901 | 166 | PREDICTED: plasma membrane calcium-transporting ATPase 2 isoform X2 [Tribolium castaneum] |
| map04020 Calcium signaling pathway | Gene.901 | 16 | PREDICTED: plasma membrane calcium-transporting ATPase 2 isoform X2 [Tribolium castaneum] |
| map04020 Calcium signaling pathway | Gene.298 | 116 | "Csa-calmodulin 3, partial [Cupiennius salei]" |
| map04020 Calcium signaling pathway | Gene.298 | 95 | "Csa-calmodulin 3, partial [Cupiennius salei]" |
| map04020 Calcium signaling pathway | Gene.298 | 31 | "Csa-calmodulin 3, partial [Cupiennius salei]" |
| map04020 Calcium signaling pathway | Gene.298 | 78 | "Csa-calmodulin 3, partial [Cupiennius salei]" |
| map04020 Calcium signaling pathway | Gene.5709 | 26 | cAMP-dependent protein kinase catalytic subunit [Agrilus planipennis] |
| map04020 Calcium signaling pathway | Gene.5709 | 257 | cAMP-dependent protein kinase catalytic subunit [Agrilus planipennis] |
| map04020 Calcium signaling pathway | Gene.8096 | 274 | "PREDICTED: ADP,ATP carrier protein 1 [Tribolium castaneum]" |
| map04020 Calcium signaling pathway | Gene.8096 | 74 | "PREDICTED: ADP,ATP carrier protein 1 [Tribolium castaneum]" |
| map04020 Calcium signaling pathway | Gene.8096 | 271 | "PREDICTED: ADP,ATP carrier protein 1 [Tribolium castaneum]" |
| map04020 Calcium signaling pathway | Gene.8096 | 21 | "PREDICTED: ADP,ATP carrier protein 1 [Tribolium castaneum]" |
| map04020 Calcium signaling pathway | Gene.8096 | 63 | "PREDICTED: ADP,ATP carrier protein 1 [Tribolium castaneum]" |
| map04020 Calcium signaling pathway | Gene.8096 | 263 | "PREDICTED: ADP,ATP carrier protein 1 [Tribolium castaneum]" |
| map04020 Calcium signaling pathway | Gene.8096 | 107 | "PREDICTED: ADP,ATP carrier protein 1 [Tribolium castaneum]" |
| map04020 Calcium signaling pathway | Gene.8096 | 158 | "PREDICTED: ADP,ATP carrier protein 1 [Tribolium castaneum]" |
| map04020 Calcium signaling pathway | Gene.8096 | 60 | "PREDICTED: ADP,ATP carrier protein 1 [Tribolium castaneum]" |
| map04020 Calcium signaling pathway | Gene.8096 | 177 | "PREDICTED: ADP,ATP carrier protein 1 [Tribolium castaneum]" |
| map04020 Calcium signaling pathway | Gene.8096 | 257 | "PREDICTED: ADP,ATP carrier protein 1 [Tribolium castaneum]" |
| map04020 Calcium signaling pathway | Gene.8096 | 54 | "PREDICTED: ADP,ATP carrier protein 1 [Tribolium castaneum]" |
| map04020 Calcium signaling pathway | Gene.8098 | 93 | "hypothetical protein, partial [Rhynchophorus ferrugineus]" |
| map04020 Calcium signaling pathway | Gene.8098 | 53 | "hypothetical protein, partial [Rhynchophorus ferrugineus]" |
| map04020 Calcium signaling pathway | Gene.8098 | 64 | "hypothetical protein, partial [Rhynchophorus ferrugineus]" |
| map04020 Calcium signaling pathway | Gene.8098 | 106 | "hypothetical protein, partial [Rhynchophorus ferrugineus]" |
| map04020 Calcium signaling pathway | Gene.8098 | 97 | "hypothetical protein, partial [Rhynchophorus ferrugineus]" |
| map04020 Calcium signaling pathway | Gene.8098 | 148 | "hypothetical protein, partial [Rhynchophorus ferrugineus]" |
| map04020 Calcium signaling pathway | Gene.8098 | 167 | "hypothetical protein, partial [Rhynchophorus ferrugineus]" |
| map04020 Calcium signaling pathway | Gene.8407 | 27 | PREDICTED: voltage-dependent anion-selective channel isoform X1 [Tribolium castaneum] |
| map04020 Calcium signaling pathway | Gene.8407 | 11 | PREDICTED: voltage-dependent anion-selective channel isoform X1 [Tribolium castaneum] |
| map04020 Calcium signaling pathway | Gene.8407 | 60 | PREDICTED: voltage-dependent anion-selective channel isoform X1 [Tribolium castaneum] |
| map04020 Calcium signaling pathway | Gene.8407 | 64 | PREDICTED: voltage-dependent anion-selective channel isoform X1 [Tribolium castaneum] |
| map04064 NF-kappa B signaling pathway | Gene.5769 | 244 | casein kinase II subunit alpha isoform X1 [Pogonomyrmex barbatus] |
| map04922 Glucagon signaling pathway | Gene.7815 | 345 | "probable pyruvate dehydrogenase E1 component subunit alpha, mitochondrial isoform X2 [Leptinotarsa decemlineata]" |
| map04922 Glucagon signaling pathway | Gene.7815 | 315 | "probable pyruvate dehydrogenase E1 component subunit alpha, mitochondrial isoform X2 [Leptinotarsa decemlineata]" |
| map04922 Glucagon signaling pathway | Gene.7815 | 317 | "probable pyruvate dehydrogenase E1 component subunit alpha, mitochondrial isoform X2 [Leptinotarsa decemlineata]" |
| map04922 Glucagon signaling pathway | Gene.7815 | 77 | "probable pyruvate dehydrogenase E1 component subunit alpha, mitochondrial isoform X2 [Leptinotarsa decemlineata]" |
| map04922 Glucagon signaling pathway | Gene.7815 | 330 | "probable pyruvate dehydrogenase E1 component subunit alpha, mitochondrial isoform X2 [Leptinotarsa decemlineata]" |
| map04922 Glucagon signaling pathway | Gene.4285 | 93 | PREDICTED: LOW QUALITY PROTEIN: pyruvate kinase-like [Aethina tumida] |
| map04922 Glucagon signaling pathway | Gene.4285 | 245 | PREDICTED: LOW QUALITY PROTEIN: pyruvate kinase-like [Aethina tumida] |
| map04922 Glucagon signaling pathway | Gene.2997 | 50 | PREDICTED: pyruvate kinase-like isoform X3 [Dendroctonus ponderosae] |
| map04922 Glucagon signaling pathway | Gene.2997 | 57 | PREDICTED: pyruvate kinase-like isoform X3 [Dendroctonus ponderosae] |
| map04922 Glucagon signaling pathway | Gene.2997 | 310 | PREDICTED: pyruvate kinase-like isoform X3 [Dendroctonus ponderosae] |
| map04922 Glucagon signaling pathway | Gene.2997 | 492 | PREDICTED: pyruvate kinase-like isoform X3 [Dendroctonus ponderosae] |
| map04922 Glucagon signaling pathway | Gene.2997 | 94 | PREDICTED: pyruvate kinase-like isoform X3 [Dendroctonus ponderosae] |
| map04922 Glucagon signaling pathway | Gene.2997 | 135 | PREDICTED: pyruvate kinase-like isoform X3 [Dendroctonus ponderosae] |
| map04922 Glucagon signaling pathway | Gene.2997 | 377 | PREDICTED: pyruvate kinase-like isoform X3 [Dendroctonus ponderosae] |
| map04922 Glucagon signaling pathway | Gene.2997 | 145 | PREDICTED: pyruvate kinase-like isoform X3 [Dendroctonus ponderosae] |
| map04922 Glucagon signaling pathway | Gene.2997 | 264 | PREDICTED: pyruvate kinase-like isoform X3 [Dendroctonus ponderosae] |
| map04922 Glucagon signaling pathway | Gene.2997 | 255 | PREDICTED: pyruvate kinase-like isoform X3 [Dendroctonus ponderosae] |
| map04922 Glucagon signaling pathway | Gene.2997 | 140 | PREDICTED: pyruvate kinase-like isoform X3 [Dendroctonus ponderosae] |
| map04922 Glucagon signaling pathway | Gene.2997 | 478 | PREDICTED: pyruvate kinase-like isoform X3 [Dendroctonus ponderosae] |
| map04922 Glucagon signaling pathway | Gene.7676 | 53 | PREDICTED: guanine nucleotide-binding protein G(q) subunit alpha isoform X1 [Tribolium castaneum] |
| map04922 Glucagon signaling pathway | Gene.4419 | 291 | glycogen phosphorylase [Harmonia axyridis] |
| map04922 Glucagon signaling pathway | Gene.4419 | 177 | glycogen phosphorylase [Harmonia axyridis] |
| map04922 Glucagon signaling pathway | Gene.4419 | 10 | glycogen phosphorylase [Harmonia axyridis] |
| map04922 Glucagon signaling pathway | Gene.4419 | 29 | glycogen phosphorylase [Harmonia axyridis] |
| map04922 Glucagon signaling pathway | Gene.4419 | 249 | glycogen phosphorylase [Harmonia axyridis] |
| map04922 Glucagon signaling pathway | Gene.4419 | 78 | glycogen phosphorylase [Harmonia axyridis] |
| map04922 Glucagon signaling pathway | Gene.3489 | 2124 | PREDICTED: acetyl-CoA carboxylase isoform X1 [Tribolium castaneum] |
| map04922 Glucagon signaling pathway | Gene.3489 | 2067 | PREDICTED: acetyl-CoA carboxylase isoform X1 [Tribolium castaneum] |
| map04922 Glucagon signaling pathway | Gene.3489 | 387 | PREDICTED: acetyl-CoA carboxylase isoform X1 [Tribolium castaneum] |
| map04922 Glucagon signaling pathway | Gene.3489 | 1848 | PREDICTED: acetyl-CoA carboxylase isoform X1 [Tribolium castaneum] |
| map04922 Glucagon signaling pathway | Gene.3489 | 2305 | PREDICTED: acetyl-CoA carboxylase isoform X1 [Tribolium castaneum] |
| map04922 Glucagon signaling pathway | Gene.3489 | 341 | PREDICTED: acetyl-CoA carboxylase isoform X1 [Tribolium castaneum] |
| map04922 Glucagon signaling pathway | Gene.3489 | 167 | PREDICTED: acetyl-CoA carboxylase isoform X1 [Tribolium castaneum] |
| map04922 Glucagon signaling pathway | Gene.3489 | 1964 | PREDICTED: acetyl-CoA carboxylase isoform X1 [Tribolium castaneum] |
| map04922 Glucagon signaling pathway | Gene.3489 | 1645 | PREDICTED: acetyl-CoA carboxylase isoform X1 [Tribolium castaneum] |
| map04922 Glucagon signaling pathway | Gene.3489 | 1389 | PREDICTED: acetyl-CoA carboxylase isoform X1 [Tribolium castaneum] |
| map04922 Glucagon signaling pathway | Gene.3489 | 2131 | PREDICTED: acetyl-CoA carboxylase isoform X1 [Tribolium castaneum] |
| map04922 Glucagon signaling pathway | Gene.3489 | 179 | PREDICTED: acetyl-CoA carboxylase isoform X1 [Tribolium castaneum] |
| map04922 Glucagon signaling pathway | Gene.3489 | 383 | PREDICTED: acetyl-CoA carboxylase isoform X1 [Tribolium castaneum] |
| map04922 Glucagon signaling pathway | Gene.3489 | 1820 | PREDICTED: acetyl-CoA carboxylase isoform X1 [Tribolium castaneum] |
| map04922 Glucagon signaling pathway | Gene.3489 | 1264 | PREDICTED: acetyl-CoA carboxylase isoform X1 [Tribolium castaneum] |
| map04922 Glucagon signaling pathway | Gene.3489 | 1412 | PREDICTED: acetyl-CoA carboxylase isoform X1 [Tribolium castaneum] |
| map04922 Glucagon signaling pathway | Gene.3489 | 2235 | PREDICTED: acetyl-CoA carboxylase isoform X1 [Tribolium castaneum] |
| map04922 Glucagon signaling pathway | Gene.3489 | 746 | PREDICTED: acetyl-CoA carboxylase isoform X1 [Tribolium castaneum] |
| map04922 Glucagon signaling pathway | Gene.3489 | 2342 | PREDICTED: acetyl-CoA carboxylase isoform X1 [Tribolium castaneum] |
| map04922 Glucagon signaling pathway | Gene.3489 | 1379 | PREDICTED: acetyl-CoA carboxylase isoform X1 [Tribolium castaneum] |
| map04922 Glucagon signaling pathway | Gene.3489 | 1369 | PREDICTED: acetyl-CoA carboxylase isoform X1 [Tribolium castaneum] |
| map04922 Glucagon signaling pathway | Gene.3489 | 2245 | PREDICTED: acetyl-CoA carboxylase isoform X1 [Tribolium castaneum] |
| map04922 Glucagon signaling pathway | Gene.5803 | 20 | calcium-binding protein E63-1 [Anoplophora glabripennis] |
| map04922 Glucagon signaling pathway | Gene.3972 | 228 | "pyruvate dehydrogenase E1 component subunit beta, mitochondrial [Leptinotarsa decemlineata]" |
| map04922 Glucagon signaling pathway | Gene.3972 | 185 | "pyruvate dehydrogenase E1 component subunit beta, mitochondrial [Leptinotarsa decemlineata]" |
| map04922 Glucagon signaling pathway | Gene.3972 | 260 | "pyruvate dehydrogenase E1 component subunit beta, mitochondrial [Leptinotarsa decemlineata]" |
| map04922 Glucagon signaling pathway | Gene.5709 | 26 | cAMP-dependent protein kinase catalytic subunit [Agrilus planipennis] |
| map04922 Glucagon signaling pathway | Gene.5709 | 257 | cAMP-dependent protein kinase catalytic subunit [Agrilus planipennis] |
| map04922 Glucagon signaling pathway | Gene.298 | 116 | "Csa-calmodulin 3, partial [Cupiennius salei]" |
| map04922 Glucagon signaling pathway | Gene.298 | 95 | "Csa-calmodulin 3, partial [Cupiennius salei]" |
| map04922 Glucagon signaling pathway | Gene.298 | 31 | "Csa-calmodulin 3, partial [Cupiennius salei]" |
| map04922 Glucagon signaling pathway | Gene.298 | 78 | "Csa-calmodulin 3, partial [Cupiennius salei]" |
| map04922 Glucagon signaling pathway | Gene.8017 | 315 | glycogen synthase [Harmonia axyridis] |
| map04922 Glucagon signaling pathway | Gene.8017 | 298 | glycogen synthase [Harmonia axyridis] |
| map04922 Glucagon signaling pathway | Gene.8017 | 247 | glycogen synthase [Harmonia axyridis] |
| map04922 Glucagon signaling pathway | Gene.5844 | 181 | phosphoglycerate mutase 1 [Asbolus verrucosus] |
| map04922 Glucagon signaling pathway | Gene.5844 | 165 | phosphoglycerate mutase 1 [Asbolus verrucosus] |
| map04922 Glucagon signaling pathway | Gene.5844 | 68 | phosphoglycerate mutase 1 [Asbolus verrucosus] |
| map04922 Glucagon signaling pathway | Gene.5844 | 122 | phosphoglycerate mutase 1 [Asbolus verrucosus] |
| map04922 Glucagon signaling pathway | Gene.5844 | 105 | phosphoglycerate mutase 1 [Asbolus verrucosus] |
| map04922 Glucagon signaling pathway | Gene.5844 | 176 | phosphoglycerate mutase 1 [Asbolus verrucosus] |
| map04922 Glucagon signaling pathway | Gene.4417 | 92 | glycogen phosphorylase [Harmonia axyridis] |
| map04922 Glucagon signaling pathway | Gene.4417 | 205 | glycogen phosphorylase [Harmonia axyridis] |
| map04922 Glucagon signaling pathway | Gene.4417 | 120 | glycogen phosphorylase [Harmonia axyridis] |
| map04922 Glucagon signaling pathway | Gene.4417 | 134 | glycogen phosphorylase [Harmonia axyridis] |
| map04922 Glucagon signaling pathway | Gene.4417 | 44 | glycogen phosphorylase [Harmonia axyridis] |
| map04922 Glucagon signaling pathway | Gene.4417 | 288 | glycogen phosphorylase [Harmonia axyridis] |
| map04922 Glucagon signaling pathway | Gene.4417 | 113 | glycogen phosphorylase [Harmonia axyridis] |
| map04922 Glucagon signaling pathway | Gene.3247 | 93 | PREDICTED: phosphoenolpyruvate carboxykinase [GTP] isoform X1 [Tribolium castaneum] |
| map05215 Prostate cancer | Gene.1845 | 92 | PREDICTED: glycogen synthase kinase-3 beta isoform X10 [Tribolium castaneum] |
| map05215 Prostate cancer | Gene.1845 | 87 | PREDICTED: glycogen synthase kinase-3 beta isoform X10 [Tribolium castaneum] |
| map05215 Prostate cancer | Gene.2725 | 81 | PREDICTED: endoplasmin [Tribolium castaneum] |
| map05215 Prostate cancer | Gene.2725 | 103 | PREDICTED: endoplasmin [Tribolium castaneum] |
| map05215 Prostate cancer | Gene.2725 | 549 | PREDICTED: endoplasmin [Tribolium castaneum] |
| map05215 Prostate cancer | Gene.2725 | 554 | PREDICTED: endoplasmin [Tribolium castaneum] |
| map05215 Prostate cancer | Gene.2725 | 490 | PREDICTED: endoplasmin [Tribolium castaneum] |
| map05215 Prostate cancer | Gene.7264 | 643 | "heat shock protein 90, partial [Harmonia axyridis]" |
| map05215 Prostate cancer | Gene.7264 | 559 | "heat shock protein 90, partial [Harmonia axyridis]" |
| map05215 Prostate cancer | Gene.7264 | 571 | "heat shock protein 90, partial [Harmonia axyridis]" |
| map05215 Prostate cancer | Gene.7264 | 67 | "heat shock protein 90, partial [Harmonia axyridis]" |
| map05215 Prostate cancer | Gene.7264 | 217 | "heat shock protein 90, partial [Harmonia axyridis]" |
| map05215 Prostate cancer | Gene.7264 | 475 | "heat shock protein 90, partial [Harmonia axyridis]" |
| map05215 Prostate cancer | Gene.7264 | 341 | "heat shock protein 90, partial [Harmonia axyridis]" |
| map05215 Prostate cancer | Gene.7264 | 269 | "heat shock protein 90, partial [Harmonia axyridis]" |
| map05215 Prostate cancer | Gene.7264 | 464 | "heat shock protein 90, partial [Harmonia axyridis]" |
| map05215 Prostate cancer | Gene.7264 | 212 | "heat shock protein 90, partial [Harmonia axyridis]" |
| map05215 Prostate cancer | Gene.7264 | 72 | "heat shock protein 90, partial [Harmonia axyridis]" |
| map05215 Prostate cancer | Gene.7264 | 532 | "heat shock protein 90, partial [Harmonia axyridis]" |
| map05215 Prostate cancer | Gene.7264 | 444 | "heat shock protein 90, partial [Harmonia axyridis]" |
| map05215 Prostate cancer | Gene.7264 | 105 | "heat shock protein 90, partial [Harmonia axyridis]" |
| map05215 Prostate cancer | Gene.7264 | 525 | "heat shock protein 90, partial [Harmonia axyridis]" |
| map05215 Prostate cancer | Gene.7264 | 553 | "heat shock protein 90, partial [Harmonia axyridis]" |
| map05215 Prostate cancer | Gene.7264 | 562 | "heat shock protein 90, partial [Harmonia axyridis]" |
| map05215 Prostate cancer | Gene.7264 | 259 | "heat shock protein 90, partial [Harmonia axyridis]" |
| map05215 Prostate cancer | Gene.7264 | 280 | "heat shock protein 90, partial [Harmonia axyridis]" |
| map05215 Prostate cancer | Gene.7264 | 51 | "heat shock protein 90, partial [Harmonia axyridis]" |
| map05215 Prostate cancer | Gene.7264 | 429 | "heat shock protein 90, partial [Harmonia axyridis]" |
| map05215 Prostate cancer | Gene.7264 | 601 | "heat shock protein 90, partial [Harmonia axyridis]" |
| map05215 Prostate cancer | Gene.7264 | 184 | "heat shock protein 90, partial [Harmonia axyridis]" |
| map05215 Prostate cancer | Gene.7264 | 568 | "heat shock protein 90, partial [Harmonia axyridis]" |
| map05215 Prostate cancer | Gene.7264 | 393 | "heat shock protein 90, partial [Harmonia axyridis]" |
| map05215 Prostate cancer | Gene.7264 | 278 | "heat shock protein 90, partial [Harmonia axyridis]" |
| map05215 Prostate cancer | Gene.7264 | 544 | "heat shock protein 90, partial [Harmonia axyridis]" |
| map05215 Prostate cancer | Gene.7264 | 432 | "heat shock protein 90, partial [Harmonia axyridis]" |
| map05215 Prostate cancer | Gene.7264 | 405 | "heat shock protein 90, partial [Harmonia axyridis]" |
| map05215 Prostate cancer | Gene.7264 | 195 | "heat shock protein 90, partial [Harmonia axyridis]" |
| map05215 Prostate cancer | Gene.2724 | 2 | PREDICTED: endoplasmin [Tribolium castaneum] |
| map05215 Prostate cancer | Gene.2724 | 29 | PREDICTED: endoplasmin [Tribolium castaneum] |
| map04962 Vasopressin-regulated water reabsorption | Gene.5709 | 26 | cAMP-dependent protein kinase catalytic subunit [Agrilus planipennis] |
| map04962 Vasopressin-regulated water reabsorption | Gene.5709 | 257 | cAMP-dependent protein kinase catalytic subunit [Agrilus planipennis] |
| map04962 Vasopressin-regulated water reabsorption | Gene.2582 | 127 | rho GDP-dissociation inhibitor 1 [Anoplophora glabripennis] |
| map04962 Vasopressin-regulated water reabsorption | Gene.2582 | 45 | rho GDP-dissociation inhibitor 1 [Anoplophora glabripennis] |
| map04962 Vasopressin-regulated water reabsorption | Gene.2348 | 116 | ras-related protein Rab-5B isoform X1 [Leptinotarsa decemlineata] |
| map04962 Vasopressin-regulated water reabsorption | Gene.5636 | 179 | ras-related protein Rab-11A [Anoplophora glabripennis] |
| map04723 Retrograde endocannabinoid signaling | Gene.5910 | 270 | "PREDICTED: NADH dehydrogenase [ubiquinone] 1 alpha subcomplex subunit 10, mitochondrial [Tribolium castaneum]" |
| map04723 Retrograde endocannabinoid signaling | Gene.164 | 78 | PREDICTED: guanine nucleotide-binding protein subunit beta-1 [Tribolium castaneum] |
| map04723 Retrograde endocannabinoid signaling | Gene.742 | 137 | "NADH dehydrogenase [ubiquinone] 1 alpha subcomplex subunit 9, mitochondrial [Anoplophora glabripennis]" |
| map04723 Retrograde endocannabinoid signaling | Gene.742 | 278 | "NADH dehydrogenase [ubiquinone] 1 alpha subcomplex subunit 9, mitochondrial [Anoplophora glabripennis]" |
| map04723 Retrograde endocannabinoid signaling | Gene.742 | 142 | "NADH dehydrogenase [ubiquinone] 1 alpha subcomplex subunit 9, mitochondrial [Anoplophora glabripennis]" |
| map04723 Retrograde endocannabinoid signaling | Gene.742 | 268 | "NADH dehydrogenase [ubiquinone] 1 alpha subcomplex subunit 9, mitochondrial [Anoplophora glabripennis]" |
| map04723 Retrograde endocannabinoid signaling | Gene.5885 | 94 | NADH dehydrogenase [ubiquinone] 1 alpha subcomplex subunit 6 [Onthophagus taurus] |
| map04723 Retrograde endocannabinoid signaling | Gene.3481 | 53 | NADH dehydrogenase [ubiquinone] 1 alpha subcomplex subunit 8 [Asbolus verrucosus] |
| map04723 Retrograde endocannabinoid signaling | Gene.5709 | 26 | cAMP-dependent protein kinase catalytic subunit [Agrilus planipennis] |
| map04723 Retrograde endocannabinoid signaling | Gene.5709 | 257 | cAMP-dependent protein kinase catalytic subunit [Agrilus planipennis] |
| map04723 Retrograde endocannabinoid signaling | Gene.2034 | 471 | "NADH dehydrogenase [ubiquinone] flavoprotein 1, mitochondrial [Tribolium castaneum]" |
| map04723 Retrograde endocannabinoid signaling | Gene.2034 | 381 | "NADH dehydrogenase [ubiquinone] flavoprotein 1, mitochondrial [Tribolium castaneum]" |
| map04723 Retrograde endocannabinoid signaling | Gene.2034 | 47 | "NADH dehydrogenase [ubiquinone] flavoprotein 1, mitochondrial [Tribolium castaneum]" |
| map04723 Retrograde endocannabinoid signaling | Gene.5767 | 89 | NADH dehydrogenase [ubiquinone] iron-sulfur protein 5 [Anoplophora glabripennis] |
| map04723 Retrograde endocannabinoid signaling | Gene.6135 | 95 | PREDICTED: NADH dehydrogenase [ubiquinone] 1 alpha subcomplex subunit 13-like [Aethina tumida] |
| map04723 Retrograde endocannabinoid signaling | Gene.8516 | 373 | PREDICTED: NADH-ubiquinone oxidoreductase 49 kDa subunit [Tribolium castaneum] |
| map04723 Retrograde endocannabinoid signaling | Gene.8516 | 310 | PREDICTED: NADH-ubiquinone oxidoreductase 49 kDa subunit [Tribolium castaneum] |
| map04723 Retrograde endocannabinoid signaling | Gene.8516 | 368 | PREDICTED: NADH-ubiquinone oxidoreductase 49 kDa subunit [Tribolium castaneum] |
| map04723 Retrograde endocannabinoid signaling | Gene.7676 | 53 | PREDICTED: guanine nucleotide-binding protein G(q) subunit alpha isoform X1 [Tribolium castaneum] |
| map04723 Retrograde endocannabinoid signaling | Gene.3795 | 166 | "NADH-ubiquinone oxidoreductase 75 kDa subunit, mitochondrial [Asbolus verrucosus]" |
| map04723 Retrograde endocannabinoid signaling | Gene.3795 | 172 | "NADH-ubiquinone oxidoreductase 75 kDa subunit, mitochondrial [Asbolus verrucosus]" |
| map04723 Retrograde endocannabinoid signaling | Gene.3795 | 691 | "NADH-ubiquinone oxidoreductase 75 kDa subunit, mitochondrial [Asbolus verrucosus]" |
| map04723 Retrograde endocannabinoid signaling | Gene.3795 | 626 | "NADH-ubiquinone oxidoreductase 75 kDa subunit, mitochondrial [Asbolus verrucosus]" |
| map04723 Retrograde endocannabinoid signaling | Gene.3795 | 307 | "NADH-ubiquinone oxidoreductase 75 kDa subunit, mitochondrial [Asbolus verrucosus]" |
| map04723 Retrograde endocannabinoid signaling | Gene.3795 | 541 | "NADH-ubiquinone oxidoreductase 75 kDa subunit, mitochondrial [Asbolus verrucosus]" |
| map04723 Retrograde endocannabinoid signaling | Gene.3795 | 622 | "NADH-ubiquinone oxidoreductase 75 kDa subunit, mitochondrial [Asbolus verrucosus]" |
| map04723 Retrograde endocannabinoid signaling | Gene.3063 | 178 | PREDICTED: uncharacterized protein LOC108560181 [Nicrophorus vespilloides] |
| map04723 Retrograde endocannabinoid signaling | Gene.6318 | 57 | NADH dehydrogenase [ubiquinone] 1 beta subcomplex subunit 3 [Leptinotarsa decemlineata] |
| map04723 Retrograde endocannabinoid signaling | Gene.6318 | 43 | NADH dehydrogenase [ubiquinone] 1 beta subcomplex subunit 3 [Leptinotarsa decemlineata] |
| map04723 Retrograde endocannabinoid signaling | Gene.6318 | 23 | NADH dehydrogenase [ubiquinone] 1 beta subcomplex subunit 3 [Leptinotarsa decemlineata] |
| map04723 Retrograde endocannabinoid signaling | Gene.2624 | 70 | PREDICTED: NADH dehydrogenase [ubiquinone] 1 alpha subcomplex subunit 5 [Tribolium castaneum] |
| map04723 Retrograde endocannabinoid signaling | Gene.6252 | 60 | NADH dehydrogenase [ubiquinone] 1 beta subcomplex subunit 4 [Leptinotarsa decemlineata] |
| map04723 Retrograde endocannabinoid signaling | Gene.6252 | 111 | NADH dehydrogenase [ubiquinone] 1 beta subcomplex subunit 4 [Leptinotarsa decemlineata] |
| map04723 Retrograde endocannabinoid signaling | Gene.6262 | 104 | "probable NADH dehydrogenase [ubiquinone] flavoprotein 2, mitochondrial [Leptinotarsa decemlineata]" |
| map04723 Retrograde endocannabinoid signaling | Gene.6262 | 230 | "probable NADH dehydrogenase [ubiquinone] flavoprotein 2, mitochondrial [Leptinotarsa decemlineata]" |
| map04723 Retrograde endocannabinoid signaling | Gene.8771 | 54 | "PREDICTED: NADH dehydrogenase [ubiquinone] iron-sulfur protein 6, mitochondrial [Tribolium castaneum]" |
| map04723 Retrograde endocannabinoid signaling | Gene.1912 | 120 | PREDICTED: NADH dehydrogenase [ubiquinone] 1 beta subcomplex subunit 9 [Aethina tumida] |
| map04723 Retrograde endocannabinoid signaling | Gene.1912 | 51 | PREDICTED: NADH dehydrogenase [ubiquinone] 1 beta subcomplex subunit 9 [Aethina tumida] |
| map05034 Alcoholism | Gene.5709 | 26 | cAMP-dependent protein kinase catalytic subunit [Agrilus planipennis] |
| map05034 Alcoholism | Gene.5709 | 257 | cAMP-dependent protein kinase catalytic subunit [Agrilus planipennis] |
| map05034 Alcoholism | Gene.298 | 116 | "Csa-calmodulin 3, partial [Cupiennius salei]" |
| map05034 Alcoholism | Gene.298 | 95 | "Csa-calmodulin 3, partial [Cupiennius salei]" |
| map05034 Alcoholism | Gene.298 | 31 | "Csa-calmodulin 3, partial [Cupiennius salei]" |
| map05034 Alcoholism | Gene.298 | 78 | "Csa-calmodulin 3, partial [Cupiennius salei]" |
| map05034 Alcoholism | Gene.164 | 78 | PREDICTED: guanine nucleotide-binding protein subunit beta-1 [Tribolium castaneum] |
| map05034 Alcoholism | Gene.6221 | 123 | PREDICTED: histone H3.3-like [Takifugu rubripes] |
| map05034 Alcoholism | Gene.6221 | 80 | PREDICTED: histone H3.3-like [Takifugu rubripes] |
| map05034 Alcoholism | Gene.5803 | 20 | calcium-binding protein E63-1 [Anoplophora glabripennis] |
| map05034 Alcoholism | Gene.502 | 147 | "acid phosphatase, partial [Cryptolaemus montrouzieri]" |
| map05034 Alcoholism | Gene.502 | 141 | "acid phosphatase, partial [Cryptolaemus montrouzieri]" |
| map05034 Alcoholism | Gene.502 | 260 | "acid phosphatase, partial [Cryptolaemus montrouzieri]" |
| map05034 Alcoholism | Gene.1218 | 27 | histone H2B-like [Zootermopsis nevadensis] |
| map05034 Alcoholism | Gene.1218 | 123 | histone H2B-like [Zootermopsis nevadensis] |
| map05034 Alcoholism | Gene.1218 | 127 | histone H2B-like [Zootermopsis nevadensis] |
| map05034 Alcoholism | Gene.1218 | 53 | histone H2B-like [Zootermopsis nevadensis] |
| map05034 Alcoholism | Gene.1218 | 41 | histone H2B-like [Zootermopsis nevadensis] |
| map05034 Alcoholism | Gene.1218 | 115 | histone H2B-like [Zootermopsis nevadensis] |
| map05034 Alcoholism | Gene.1218 | 92 | histone H2B-like [Zootermopsis nevadensis] |
| map05034 Alcoholism | Gene.1218 | 31 | histone H2B-like [Zootermopsis nevadensis] |
| map01521 EGFR tyrosine kinase inhibitor resistance | Gene.1845 | 92 | PREDICTED: glycogen synthase kinase-3 beta isoform X10 [Tribolium castaneum] |
| map01521 EGFR tyrosine kinase inhibitor resistance | Gene.1845 | 87 | PREDICTED: glycogen synthase kinase-3 beta isoform X10 [Tribolium castaneum] |
| map01521 EGFR tyrosine kinase inhibitor resistance | Gene.919 | 149 | ribosomal protein S6 [Chrysomela tremula] |
| map01521 EGFR tyrosine kinase inhibitor resistance | Gene.919 | 159 | ribosomal protein S6 [Chrysomela tremula] |
| map01521 EGFR tyrosine kinase inhibitor resistance | Gene.919 | 165 | ribosomal protein S6 [Chrysomela tremula] |
| map01521 EGFR tyrosine kinase inhibitor resistance | Gene.919 | 51 | ribosomal protein S6 [Chrysomela tremula] |
| map01521 EGFR tyrosine kinase inhibitor resistance | Gene.919 | 58 | ribosomal protein S6 [Chrysomela tremula] |
| map01521 EGFR tyrosine kinase inhibitor resistance | Gene.919 | 211 | ribosomal protein S6 [Chrysomela tremula] |
| map01521 EGFR tyrosine kinase inhibitor resistance | Gene.919 | 14 | ribosomal protein S6 [Chrysomela tremula] |
| map01521 EGFR tyrosine kinase inhibitor resistance | Gene.919 | 23 | ribosomal protein S6 [Chrysomela tremula] |
| map04611 Platelet activation | Gene.5154 | 2626 | PREDICTED: hemocytin isoform X2 [Tribolium castaneum] |
| map04611 Platelet activation | Gene.5154 | 2001 | PREDICTED: hemocytin isoform X2 [Tribolium castaneum] |
| map04611 Platelet activation | Gene.5709 | 26 | cAMP-dependent protein kinase catalytic subunit [Agrilus planipennis] |
| map04611 Platelet activation | Gene.5709 | 257 | cAMP-dependent protein kinase catalytic subunit [Agrilus planipennis] |
| map04611 Platelet activation | Gene.7676 | 53 | PREDICTED: guanine nucleotide-binding protein G(q) subunit alpha isoform X1 [Tribolium castaneum] |
| map04611 Platelet activation | Gene.4326 | 171 | hemocytin [Agrilus planipennis] |
| map04611 Platelet activation | Gene.4326 | 62 | hemocytin [Agrilus planipennis] |
| map04611 Platelet activation | Gene.502 | 147 | "acid phosphatase, partial [Cryptolaemus montrouzieri]" |
| map04611 Platelet activation | Gene.502 | 141 | "acid phosphatase, partial [Cryptolaemus montrouzieri]" |
| map04611 Platelet activation | Gene.502 | 260 | "acid phosphatase, partial [Cryptolaemus montrouzieri]" |
| map04611 Platelet activation | Gene.172 | 216 | "beta-actin, partial [Cotesia chilonis]" |
| map04611 Platelet activation | Gene.172 | 51 | "beta-actin, partial [Cotesia chilonis]" |
| map04611 Platelet activation | Gene.172 | 114 | "beta-actin, partial [Cotesia chilonis]" |
| map04611 Platelet activation | Gene.172 | 62 | "beta-actin, partial [Cotesia chilonis]" |
| map04611 Platelet activation | Gene.2646 | 99 | beta actin [Polyrhachis vicina] |
| map04611 Platelet activation | Gene.2646 | 88 | beta actin [Polyrhachis vicina] |
| map04611 Platelet activation | Gene.2646 | 64 | beta actin [Polyrhachis vicina] |
| map04611 Platelet activation | Gene.2646 | 101 | beta actin [Polyrhachis vicina] |
| map05226 Gastric cancer | Gene.1845 | 92 | PREDICTED: glycogen synthase kinase-3 beta isoform X10 [Tribolium castaneum] |
| map05226 Gastric cancer | Gene.1845 | 87 | PREDICTED: glycogen synthase kinase-3 beta isoform X10 [Tribolium castaneum] |
| map05226 Gastric cancer | Gene.1224 | 985 | PREDICTED: multidrug resistance protein 1 [Tribolium castaneum] |
| map05226 Gastric cancer | Gene.1224 | 393 | PREDICTED: multidrug resistance protein 1 [Tribolium castaneum] |
| map05226 Gastric cancer | Gene.1224 | 534 | PREDICTED: multidrug resistance protein 1 [Tribolium castaneum] |
| map05226 Gastric cancer | Gene.1224 | 268 | PREDICTED: multidrug resistance protein 1 [Tribolium castaneum] |
| map05226 Gastric cancer | Gene.1224 | 576 | PREDICTED: multidrug resistance protein 1 [Tribolium castaneum] |
| map05226 Gastric cancer | Gene.1224 | 1158 | PREDICTED: multidrug resistance protein 1 [Tribolium castaneum] |
| map05135 Yersinia infection | Gene.1845 | 92 | PREDICTED: glycogen synthase kinase-3 beta isoform X10 [Tribolium castaneum] |
| map05135 Yersinia infection | Gene.1845 | 87 | PREDICTED: glycogen synthase kinase-3 beta isoform X10 [Tribolium castaneum] |
| map05135 Yersinia infection | Gene.7526 | 96 | ras-related protein Rac1 [Anoplophora glabripennis] |
| map05135 Yersinia infection | Gene.172 | 216 | "beta-actin, partial [Cotesia chilonis]" |
| map05135 Yersinia infection | Gene.172 | 51 | "beta-actin, partial [Cotesia chilonis]" |
| map05135 Yersinia infection | Gene.172 | 114 | "beta-actin, partial [Cotesia chilonis]" |
| map05135 Yersinia infection | Gene.172 | 62 | "beta-actin, partial [Cotesia chilonis]" |
| map05135 Yersinia infection | Gene.2646 | 99 | beta actin [Polyrhachis vicina] |
| map05135 Yersinia infection | Gene.2646 | 88 | beta actin [Polyrhachis vicina] |
| map05135 Yersinia infection | Gene.2646 | 64 | beta actin [Polyrhachis vicina] |
| map05135 Yersinia infection | Gene.2646 | 101 | beta actin [Polyrhachis vicina] |
| map04728 Dopaminergic synapse | Gene.7676 | 53 | PREDICTED: guanine nucleotide-binding protein G(q) subunit alpha isoform X1 [Tribolium castaneum] |
| map04728 Dopaminergic synapse | Gene.6561 | 544 | "protein phosphatase 2, regulatory subunit A, alpha isoform [Tribolium castaneum]" |
| map04728 Dopaminergic synapse | Gene.6561 | 268 | "protein phosphatase 2, regulatory subunit A, alpha isoform [Tribolium castaneum]" |
| map04728 Dopaminergic synapse | Gene.164 | 78 | PREDICTED: guanine nucleotide-binding protein subunit beta-1 [Tribolium castaneum] |
| map04728 Dopaminergic synapse | Gene.5803 | 20 | calcium-binding protein E63-1 [Anoplophora glabripennis] |
| map04728 Dopaminergic synapse | Gene.502 | 147 | "acid phosphatase, partial [Cryptolaemus montrouzieri]" |
| map04728 Dopaminergic synapse | Gene.502 | 141 | "acid phosphatase, partial [Cryptolaemus montrouzieri]" |
| map04728 Dopaminergic synapse | Gene.502 | 260 | "acid phosphatase, partial [Cryptolaemus montrouzieri]" |
| map04728 Dopaminergic synapse | Gene.298 | 116 | "Csa-calmodulin 3, partial [Cupiennius salei]" |
| map04728 Dopaminergic synapse | Gene.298 | 95 | "Csa-calmodulin 3, partial [Cupiennius salei]" |
| map04728 Dopaminergic synapse | Gene.298 | 31 | "Csa-calmodulin 3, partial [Cupiennius salei]" |
| map04728 Dopaminergic synapse | Gene.298 | 78 | "Csa-calmodulin 3, partial [Cupiennius salei]" |
| map04728 Dopaminergic synapse | Gene.5709 | 26 | cAMP-dependent protein kinase catalytic subunit [Agrilus planipennis] |
| map04728 Dopaminergic synapse | Gene.5709 | 257 | cAMP-dependent protein kinase catalytic subunit [Agrilus planipennis] |
| map04728 Dopaminergic synapse | Gene.1845 | 92 | PREDICTED: glycogen synthase kinase-3 beta isoform X10 [Tribolium castaneum] |
| map04728 Dopaminergic synapse | Gene.1845 | 87 | PREDICTED: glycogen synthase kinase-3 beta isoform X10 [Tribolium castaneum] |
| map04370 VEGF signaling pathway | Gene.7526 | 96 | ras-related protein Rac1 [Anoplophora glabripennis] |
| map00310 Lysine degradation | Gene.2533 | 286 | PREDICTED: putative aldehyde dehydrogenase family 7 member A1 homolog [Aethina tumida] |
| map00310 Lysine degradation | Gene.2533 | 400 | PREDICTED: putative aldehyde dehydrogenase family 7 member A1 homolog [Aethina tumida] |
| map00310 Lysine degradation | Gene.2533 | 41 | PREDICTED: putative aldehyde dehydrogenase family 7 member A1 homolog [Aethina tumida] |
| map00310 Lysine degradation | Gene.2533 | 56 | PREDICTED: putative aldehyde dehydrogenase family 7 member A1 homolog [Aethina tumida] |
| map00310 Lysine degradation | Gene.2533 | 382 | PREDICTED: putative aldehyde dehydrogenase family 7 member A1 homolog [Aethina tumida] |
| map00310 Lysine degradation | Gene.2533 | 73 | PREDICTED: putative aldehyde dehydrogenase family 7 member A1 homolog [Aethina tumida] |
| map00310 Lysine degradation | Gene.2533 | 62 | PREDICTED: putative aldehyde dehydrogenase family 7 member A1 homolog [Aethina tumida] |
| map00310 Lysine degradation | Gene.2533 | 65 | PREDICTED: putative aldehyde dehydrogenase family 7 member A1 homolog [Aethina tumida] |
| map00310 Lysine degradation | Gene.8819 | 124 | "hydroxyacyl-coenzyme A dehydrogenase, mitochondrial [Anoplophora glabripennis]" |
| map00310 Lysine degradation | Gene.8819 | 272 | "hydroxyacyl-coenzyme A dehydrogenase, mitochondrial [Anoplophora glabripennis]" |
| map00310 Lysine degradation | Gene.8819 | 234 | "hydroxyacyl-coenzyme A dehydrogenase, mitochondrial [Anoplophora glabripennis]" |
| map00310 Lysine degradation | Gene.8819 | 280 | "hydroxyacyl-coenzyme A dehydrogenase, mitochondrial [Anoplophora glabripennis]" |
| map00310 Lysine degradation | Gene.8819 | 75 | "hydroxyacyl-coenzyme A dehydrogenase, mitochondrial [Anoplophora glabripennis]" |
| map00310 Lysine degradation | Gene.8819 | 64 | "hydroxyacyl-coenzyme A dehydrogenase, mitochondrial [Anoplophora glabripennis]" |
| map00310 Lysine degradation | Gene.8819 | 80 | "hydroxyacyl-coenzyme A dehydrogenase, mitochondrial [Anoplophora glabripennis]" |
| map00310 Lysine degradation | Gene.8819 | 119 | "hydroxyacyl-coenzyme A dehydrogenase, mitochondrial [Anoplophora glabripennis]" |
| map00310 Lysine degradation | Gene.8819 | 93 | "hydroxyacyl-coenzyme A dehydrogenase, mitochondrial [Anoplophora glabripennis]" |
| map00310 Lysine degradation | Gene.8819 | 293 | "hydroxyacyl-coenzyme A dehydrogenase, mitochondrial [Anoplophora glabripennis]" |
| map00310 Lysine degradation | Gene.5058 | 234 | "2-oxoglutarate dehydrogenase E1 component DHKTD1 -like protein, mitochondrial, partial [Asbolus verrucosus]" |
| map00310 Lysine degradation | Gene.4335 | 148 | "alpha-aminoadipic semialdehyde synthase, mitochondrial [Anoplophora glabripennis]" |
| map00310 Lysine degradation | Gene.2749 | 84 | "PREDICTED: aldehyde dehydrogenase, mitochondrial [Tribolium castaneum]" |
| map00310 Lysine degradation | Gene.2749 | 370 | "PREDICTED: aldehyde dehydrogenase, mitochondrial [Tribolium castaneum]" |
| map00310 Lysine degradation | Gene.2749 | 446 | "PREDICTED: aldehyde dehydrogenase, mitochondrial [Tribolium castaneum]" |
| map00310 Lysine degradation | Gene.2749 | 151 | "PREDICTED: aldehyde dehydrogenase, mitochondrial [Tribolium castaneum]" |
| map00310 Lysine degradation | Gene.2749 | 140 | "PREDICTED: aldehyde dehydrogenase, mitochondrial [Tribolium castaneum]" |
| map00310 Lysine degradation | Gene.2749 | 423 | "PREDICTED: aldehyde dehydrogenase, mitochondrial [Tribolium castaneum]" |
| map00310 Lysine degradation | Gene.922 | 243 | "acetyl-CoA acetyltransferase, mitochondrial, partial [Asbolus verrucosus]" |
| map00310 Lysine degradation | Gene.922 | 252 | "acetyl-CoA acetyltransferase, mitochondrial, partial [Asbolus verrucosus]" |
| map00310 Lysine degradation | Gene.922 | 214 | "acetyl-CoA acetyltransferase, mitochondrial, partial [Asbolus verrucosus]" |
| map00310 Lysine degradation | Gene.922 | 293 | "acetyl-CoA acetyltransferase, mitochondrial, partial [Asbolus verrucosus]" |
| map00310 Lysine degradation | Gene.922 | 249 | "acetyl-CoA acetyltransferase, mitochondrial, partial [Asbolus verrucosus]" |
| map00310 Lysine degradation | Gene.3075 | 162 | "dihydrolipoyl dehydrogenase, mitochondrial [Anoplophora glabripennis]" |
| map00310 Lysine degradation | Gene.3075 | 118 | "dihydrolipoyl dehydrogenase, mitochondrial [Anoplophora glabripennis]" |
| map00310 Lysine degradation | Gene.3075 | 259 | "dihydrolipoyl dehydrogenase, mitochondrial [Anoplophora glabripennis]" |
| map00310 Lysine degradation | Gene.3075 | 273 | "dihydrolipoyl dehydrogenase, mitochondrial [Anoplophora glabripennis]" |
| map00310 Lysine degradation | Gene.3075 | 139 | "dihydrolipoyl dehydrogenase, mitochondrial [Anoplophora glabripennis]" |
| map00310 Lysine degradation | Gene.8989 | 179 | "PREDICTED: aldehyde dehydrogenase, dimeric NADP-preferring-like [Aethina tumida]" |
| map00310 Lysine degradation | Gene.8989 | 258 | "PREDICTED: aldehyde dehydrogenase, dimeric NADP-preferring-like [Aethina tumida]" |
| map00310 Lysine degradation | Gene.8989 | 438 | "PREDICTED: aldehyde dehydrogenase, dimeric NADP-preferring-like [Aethina tumida]" |
| map00310 Lysine degradation | Gene.468 | 427 | "retinal dehydrogenase 1, partial [Asbolus verrucosus]" |
| map00310 Lysine degradation | Gene.468 | 27 | "retinal dehydrogenase 1, partial [Asbolus verrucosus]" |
| map00310 Lysine degradation | Gene.468 | 131 | "retinal dehydrogenase 1, partial [Asbolus verrucosus]" |
| map00310 Lysine degradation | Gene.468 | 20 | "retinal dehydrogenase 1, partial [Asbolus verrucosus]" |
| map00310 Lysine degradation | Gene.1305 | 104 | hypothetical protein AMK59_6936 [Oryctes borbonicus] |
| map00310 Lysine degradation | Gene.1305 | 162 | hypothetical protein AMK59_6936 [Oryctes borbonicus] |
| map00310 Lysine degradation | Gene.1305 | 214 | hypothetical protein AMK59_6936 [Oryctes borbonicus] |
| map00310 Lysine degradation | Gene.4581 | 408 | "PREDICTED: trifunctional enzyme subunit alpha, mitochondrial [Tribolium castaneum]" |
| map00310 Lysine degradation | Gene.4581 | 401 | "PREDICTED: trifunctional enzyme subunit alpha, mitochondrial [Tribolium castaneum]" |
| map00310 Lysine degradation | Gene.4581 | 280 | "PREDICTED: trifunctional enzyme subunit alpha, mitochondrial [Tribolium castaneum]" |
| map00310 Lysine degradation | Gene.4581 | 118 | "PREDICTED: trifunctional enzyme subunit alpha, mitochondrial [Tribolium castaneum]" |
| map00310 Lysine degradation | Gene.4581 | 385 | "PREDICTED: trifunctional enzyme subunit alpha, mitochondrial [Tribolium castaneum]" |
| map00310 Lysine degradation | Gene.4581 | 199 | "PREDICTED: trifunctional enzyme subunit alpha, mitochondrial [Tribolium castaneum]" |
| map00310 Lysine degradation | Gene.4581 | 249 | "PREDICTED: trifunctional enzyme subunit alpha, mitochondrial [Tribolium castaneum]" |
| map00310 Lysine degradation | Gene.4581 | 126 | "PREDICTED: trifunctional enzyme subunit alpha, mitochondrial [Tribolium castaneum]" |
| map00310 Lysine degradation | Gene.1417 | 124 | "PREDICTED: dihydrolipoyllysine-residue succinyltransferase component of 2-oxoglutarate dehydrogenase complex, mitochondrial [Tribolium castaneum]" |
| map00310 Lysine degradation | Gene.2913 | 82 | "PREDICTED: glutaryl-CoA dehydrogenase, mitochondrial [Nicrophorus vespilloides]" |
| map00310 Lysine degradation | Gene.8424 | 34 | PREDICTED: NAD/NADP-dependent betaine aldehyde dehydrogenase [Tribolium castaneum] |
| map00310 Lysine degradation | Gene.8424 | 532 | PREDICTED: NAD/NADP-dependent betaine aldehyde dehydrogenase [Tribolium castaneum] |
| map00650 Butanoate metabolism | Gene.922 | 243 | "acetyl-CoA acetyltransferase, mitochondrial, partial [Asbolus verrucosus]" |
| map00650 Butanoate metabolism | Gene.922 | 252 | "acetyl-CoA acetyltransferase, mitochondrial, partial [Asbolus verrucosus]" |
| map00650 Butanoate metabolism | Gene.922 | 214 | "acetyl-CoA acetyltransferase, mitochondrial, partial [Asbolus verrucosus]" |
| map00650 Butanoate metabolism | Gene.922 | 293 | "acetyl-CoA acetyltransferase, mitochondrial, partial [Asbolus verrucosus]" |
| map00650 Butanoate metabolism | Gene.922 | 249 | "acetyl-CoA acetyltransferase, mitochondrial, partial [Asbolus verrucosus]" |
| map00650 Butanoate metabolism | Gene.4113 | 171 | "succinyl-CoA:3-ketoacid coenzyme A transferase 1, mitochondrial, partial [Asbolus verrucosus]" |
| map00650 Butanoate metabolism | Gene.1305 | 104 | hypothetical protein AMK59_6936 [Oryctes borbonicus] |
| map00650 Butanoate metabolism | Gene.1305 | 162 | hypothetical protein AMK59_6936 [Oryctes borbonicus] |
| map00650 Butanoate metabolism | Gene.1305 | 214 | hypothetical protein AMK59_6936 [Oryctes borbonicus] |
| map00650 Butanoate metabolism | Gene.8819 | 124 | "hydroxyacyl-coenzyme A dehydrogenase, mitochondrial [Anoplophora glabripennis]" |
| map00650 Butanoate metabolism | Gene.8819 | 272 | "hydroxyacyl-coenzyme A dehydrogenase, mitochondrial [Anoplophora glabripennis]" |
| map00650 Butanoate metabolism | Gene.8819 | 234 | "hydroxyacyl-coenzyme A dehydrogenase, mitochondrial [Anoplophora glabripennis]" |
| map00650 Butanoate metabolism | Gene.8819 | 280 | "hydroxyacyl-coenzyme A dehydrogenase, mitochondrial [Anoplophora glabripennis]" |
| map00650 Butanoate metabolism | Gene.8819 | 75 | "hydroxyacyl-coenzyme A dehydrogenase, mitochondrial [Anoplophora glabripennis]" |
| map00650 Butanoate metabolism | Gene.8819 | 64 | "hydroxyacyl-coenzyme A dehydrogenase, mitochondrial [Anoplophora glabripennis]" |
| map00650 Butanoate metabolism | Gene.8819 | 80 | "hydroxyacyl-coenzyme A dehydrogenase, mitochondrial [Anoplophora glabripennis]" |
| map00650 Butanoate metabolism | Gene.8819 | 119 | "hydroxyacyl-coenzyme A dehydrogenase, mitochondrial [Anoplophora glabripennis]" |
| map00650 Butanoate metabolism | Gene.8819 | 93 | "hydroxyacyl-coenzyme A dehydrogenase, mitochondrial [Anoplophora glabripennis]" |
| map00650 Butanoate metabolism | Gene.8819 | 293 | "hydroxyacyl-coenzyme A dehydrogenase, mitochondrial [Anoplophora glabripennis]" |
| map00650 Butanoate metabolism | Gene.4581 | 408 | "PREDICTED: trifunctional enzyme subunit alpha, mitochondrial [Tribolium castaneum]" |
| map00650 Butanoate metabolism | Gene.4581 | 401 | "PREDICTED: trifunctional enzyme subunit alpha, mitochondrial [Tribolium castaneum]" |
| map00650 Butanoate metabolism | Gene.4581 | 280 | "PREDICTED: trifunctional enzyme subunit alpha, mitochondrial [Tribolium castaneum]" |
| map00650 Butanoate metabolism | Gene.4581 | 118 | "PREDICTED: trifunctional enzyme subunit alpha, mitochondrial [Tribolium castaneum]" |
| map00650 Butanoate metabolism | Gene.4581 | 385 | "PREDICTED: trifunctional enzyme subunit alpha, mitochondrial [Tribolium castaneum]" |
| map00650 Butanoate metabolism | Gene.4581 | 199 | "PREDICTED: trifunctional enzyme subunit alpha, mitochondrial [Tribolium castaneum]" |
| map00650 Butanoate metabolism | Gene.4581 | 249 | "PREDICTED: trifunctional enzyme subunit alpha, mitochondrial [Tribolium castaneum]" |
| map00650 Butanoate metabolism | Gene.4581 | 126 | "PREDICTED: trifunctional enzyme subunit alpha, mitochondrial [Tribolium castaneum]" |
| map00650 Butanoate metabolism | Gene.970 | 56 | "short-chain specific acyl-CoA dehydrogenase, mitochondrial-like [Leptinotarsa decemlineata]" |
| map00650 Butanoate metabolism | Gene.970 | 193 | "short-chain specific acyl-CoA dehydrogenase, mitochondrial-like [Leptinotarsa decemlineata]" |
| map00650 Butanoate metabolism | Gene.7388 | 327 | "short-chain specific acyl-CoA dehydrogenase, mitochondrial [Anoplophora glabripennis]" |
| map00650 Butanoate metabolism | Gene.7388 | 294 | "short-chain specific acyl-CoA dehydrogenase, mitochondrial [Anoplophora glabripennis]" |
| map00650 Butanoate metabolism | Gene.7388 | 212 | "short-chain specific acyl-CoA dehydrogenase, mitochondrial [Anoplophora glabripennis]" |
| map00650 Butanoate metabolism | Gene.7388 | 330 | "short-chain specific acyl-CoA dehydrogenase, mitochondrial [Anoplophora glabripennis]" |
| map00650 Butanoate metabolism | Gene.7388 | 298 | "short-chain specific acyl-CoA dehydrogenase, mitochondrial [Anoplophora glabripennis]" |
| map00650 Butanoate metabolism | Gene.7388 | 335 | "short-chain specific acyl-CoA dehydrogenase, mitochondrial [Anoplophora glabripennis]" |
| map00650 Butanoate metabolism | Gene.7388 | 51 | "short-chain specific acyl-CoA dehydrogenase, mitochondrial [Anoplophora glabripennis]" |
| map00650 Butanoate metabolism | Gene.7388 | 218 | "short-chain specific acyl-CoA dehydrogenase, mitochondrial [Anoplophora glabripennis]" |
| map05032 Morphine addiction | Gene.5709 | 26 | cAMP-dependent protein kinase catalytic subunit [Agrilus planipennis] |
| map05032 Morphine addiction | Gene.5709 | 257 | cAMP-dependent protein kinase catalytic subunit [Agrilus planipennis] |
| map05032 Morphine addiction | Gene.164 | 78 | PREDICTED: guanine nucleotide-binding protein subunit beta-1 [Tribolium castaneum] |
| map04917 Prolactin signaling pathway | Gene.1845 | 92 | PREDICTED: glycogen synthase kinase-3 beta isoform X10 [Tribolium castaneum] |
| map04917 Prolactin signaling pathway | Gene.1845 | 87 | PREDICTED: glycogen synthase kinase-3 beta isoform X10 [Tribolium castaneum] |
| map05016 Huntington disease | Gene.1542 | 237 | "PREDICTED: ATP synthase subunit alpha, mitochondrial [Tribolium castaneum]" |
| map05016 Huntington disease | Gene.1542 | 130 | "PREDICTED: ATP synthase subunit alpha, mitochondrial [Tribolium castaneum]" |
| map05016 Huntington disease | Gene.1542 | 63 | "PREDICTED: ATP synthase subunit alpha, mitochondrial [Tribolium castaneum]" |
| map05016 Huntington disease | Gene.1542 | 165 | "PREDICTED: ATP synthase subunit alpha, mitochondrial [Tribolium castaneum]" |
| map05016 Huntington disease | Gene.1542 | 71 | "PREDICTED: ATP synthase subunit alpha, mitochondrial [Tribolium castaneum]" |
| map05016 Huntington disease | Gene.1542 | 314 | "PREDICTED: ATP synthase subunit alpha, mitochondrial [Tribolium castaneum]" |
| map05016 Huntington disease | Gene.1542 | 537 | "PREDICTED: ATP synthase subunit alpha, mitochondrial [Tribolium castaneum]" |
| map05016 Huntington disease | Gene.1542 | 514 | "PREDICTED: ATP synthase subunit alpha, mitochondrial [Tribolium castaneum]" |
| map05016 Huntington disease | Gene.1542 | 526 | "PREDICTED: ATP synthase subunit alpha, mitochondrial [Tribolium castaneum]" |
| map05016 Huntington disease | Gene.1542 | 422 | "PREDICTED: ATP synthase subunit alpha, mitochondrial [Tribolium castaneum]" |
| map05016 Huntington disease | Gene.1542 | 159 | "PREDICTED: ATP synthase subunit alpha, mitochondrial [Tribolium castaneum]" |
| map05016 Huntington disease | Gene.1542 | 228 | "PREDICTED: ATP synthase subunit alpha, mitochondrial [Tribolium castaneum]" |
| map05016 Huntington disease | Gene.1542 | 303 | "PREDICTED: ATP synthase subunit alpha, mitochondrial [Tribolium castaneum]" |
| map05016 Huntington disease | Gene.1542 | 518 | "PREDICTED: ATP synthase subunit alpha, mitochondrial [Tribolium castaneum]" |
| map05016 Huntington disease | Gene.1542 | 425 | "PREDICTED: ATP synthase subunit alpha, mitochondrial [Tribolium castaneum]" |
| map05016 Huntington disease | Gene.1542 | 501 | "PREDICTED: ATP synthase subunit alpha, mitochondrial [Tribolium castaneum]" |
| map05016 Huntington disease | Gene.1542 | 529 | "PREDICTED: ATP synthase subunit alpha, mitochondrial [Tribolium castaneum]" |
| map05016 Huntington disease | Gene.1542 | 432 | "PREDICTED: ATP synthase subunit alpha, mitochondrial [Tribolium castaneum]" |
| map05016 Huntington disease | Gene.1542 | 507 | "PREDICTED: ATP synthase subunit alpha, mitochondrial [Tribolium castaneum]" |
| map05016 Huntington disease | Gene.1542 | 496 | "PREDICTED: ATP synthase subunit alpha, mitochondrial [Tribolium castaneum]" |
| map05016 Huntington disease | Gene.5910 | 270 | "PREDICTED: NADH dehydrogenase [ubiquinone] 1 alpha subcomplex subunit 10, mitochondrial [Tribolium castaneum]" |
| map05016 Huntington disease | Gene.742 | 137 | "NADH dehydrogenase [ubiquinone] 1 alpha subcomplex subunit 9, mitochondrial [Anoplophora glabripennis]" |
| map05016 Huntington disease | Gene.742 | 278 | "NADH dehydrogenase [ubiquinone] 1 alpha subcomplex subunit 9, mitochondrial [Anoplophora glabripennis]" |
| map05016 Huntington disease | Gene.742 | 142 | "NADH dehydrogenase [ubiquinone] 1 alpha subcomplex subunit 9, mitochondrial [Anoplophora glabripennis]" |
| map05016 Huntington disease | Gene.742 | 268 | "NADH dehydrogenase [ubiquinone] 1 alpha subcomplex subunit 9, mitochondrial [Anoplophora glabripennis]" |
| map05016 Huntington disease | Gene.5885 | 94 | NADH dehydrogenase [ubiquinone] 1 alpha subcomplex subunit 6 [Onthophagus taurus] |
| map05016 Huntington disease | Gene.7655 | 158 | "ATP synthase subunit b, mitochondrial [Leptinotarsa decemlineata]" |
| map05016 Huntington disease | Gene.7655 | 119 | "ATP synthase subunit b, mitochondrial [Leptinotarsa decemlineata]" |
| map05016 Huntington disease | Gene.7655 | 170 | "ATP synthase subunit b, mitochondrial [Leptinotarsa decemlineata]" |
| map05016 Huntington disease | Gene.7655 | 233 | "ATP synthase subunit b, mitochondrial [Leptinotarsa decemlineata]" |
| map05016 Huntington disease | Gene.7655 | 154 | "ATP synthase subunit b, mitochondrial [Leptinotarsa decemlineata]" |
| map05016 Huntington disease | Gene.7655 | 53 | "ATP synthase subunit b, mitochondrial [Leptinotarsa decemlineata]" |
| map05016 Huntington disease | Gene.7655 | 194 | "ATP synthase subunit b, mitochondrial [Leptinotarsa decemlineata]" |
| map05016 Huntington disease | Gene.143 | 69 | cytochrome c oxidase subunit IV [Tribolium castaneum] |
| map05016 Huntington disease | Gene.143 | 180 | cytochrome c oxidase subunit IV [Tribolium castaneum] |
| map05016 Huntington disease | Gene.143 | 94 | cytochrome c oxidase subunit IV [Tribolium castaneum] |
| map05016 Huntington disease | Gene.143 | 184 | cytochrome c oxidase subunit IV [Tribolium castaneum] |
| map05016 Huntington disease | Gene.2034 | 471 | "NADH dehydrogenase [ubiquinone] flavoprotein 1, mitochondrial [Tribolium castaneum]" |
| map05016 Huntington disease | Gene.2034 | 381 | "NADH dehydrogenase [ubiquinone] flavoprotein 1, mitochondrial [Tribolium castaneum]" |
| map05016 Huntington disease | Gene.2034 | 47 | "NADH dehydrogenase [ubiquinone] flavoprotein 1, mitochondrial [Tribolium castaneum]" |
| map05016 Huntington disease | Gene.8096 | 274 | "PREDICTED: ADP,ATP carrier protein 1 [Tribolium castaneum]" |
| map05016 Huntington disease | Gene.8096 | 74 | "PREDICTED: ADP,ATP carrier protein 1 [Tribolium castaneum]" |
| map05016 Huntington disease | Gene.8096 | 271 | "PREDICTED: ADP,ATP carrier protein 1 [Tribolium castaneum]" |
| map05016 Huntington disease | Gene.8096 | 21 | "PREDICTED: ADP,ATP carrier protein 1 [Tribolium castaneum]" |
| map05016 Huntington disease | Gene.8096 | 63 | "PREDICTED: ADP,ATP carrier protein 1 [Tribolium castaneum]" |
| map05016 Huntington disease | Gene.8096 | 263 | "PREDICTED: ADP,ATP carrier protein 1 [Tribolium castaneum]" |
| map05016 Huntington disease | Gene.8096 | 107 | "PREDICTED: ADP,ATP carrier protein 1 [Tribolium castaneum]" |
| map05016 Huntington disease | Gene.8096 | 158 | "PREDICTED: ADP,ATP carrier protein 1 [Tribolium castaneum]" |
| map05016 Huntington disease | Gene.8096 | 60 | "PREDICTED: ADP,ATP carrier protein 1 [Tribolium castaneum]" |
| map05016 Huntington disease | Gene.8096 | 177 | "PREDICTED: ADP,ATP carrier protein 1 [Tribolium castaneum]" |
| map05016 Huntington disease | Gene.8096 | 257 | "PREDICTED: ADP,ATP carrier protein 1 [Tribolium castaneum]" |
| map05016 Huntington disease | Gene.8096 | 54 | "PREDICTED: ADP,ATP carrier protein 1 [Tribolium castaneum]" |
| map05016 Huntington disease | Gene.8016 | 180 | "UCR TM, Rieske, and/or Ubiq-Cytc-red N domain containing protein [Asbolus verrucosus]" |
| map05016 Huntington disease | Gene.8016 | 171 | "UCR TM, Rieske, and/or Ubiq-Cytc-red N domain containing protein [Asbolus verrucosus]" |
| map05016 Huntington disease | Gene.8016 | 110 | "UCR TM, Rieske, and/or Ubiq-Cytc-red N domain containing protein [Asbolus verrucosus]" |
| map05016 Huntington disease | Gene.8016 | 176 | "UCR TM, Rieske, and/or Ubiq-Cytc-red N domain containing protein [Asbolus verrucosus]" |
| map05016 Huntington disease | Gene.6135 | 95 | PREDICTED: NADH dehydrogenase [ubiquinone] 1 alpha subcomplex subunit 13-like [Aethina tumida] |
| map05016 Huntington disease | Gene.7840 | 189 | "PREDICTED: cytochrome c1, heme protein, mitochondrial [Tribolium castaneum]" |
| map05016 Huntington disease | Gene.7840 | 44 | "PREDICTED: cytochrome c1, heme protein, mitochondrial [Tribolium castaneum]" |
| map05016 Huntington disease | Gene.2830 | 84 | PREDICTED: LOW QUALITY PROTEIN: clathrin heavy chain [Aethina tumida] |
| map05016 Huntington disease | Gene.2830 | 103 | PREDICTED: LOW QUALITY PROTEIN: clathrin heavy chain [Aethina tumida] |
| map05016 Huntington disease | Gene.2830 | 902 | PREDICTED: LOW QUALITY PROTEIN: clathrin heavy chain [Aethina tumida] |
| map05016 Huntington disease | Gene.2830 | 1619 | PREDICTED: LOW QUALITY PROTEIN: clathrin heavy chain [Aethina tumida] |
| map05016 Huntington disease | Gene.8756 | 84 | uncharacterized protein Dvir_GJ16722 [Drosophila virilis] |
| map05016 Huntington disease | Gene.8756 | 10 | uncharacterized protein Dvir_GJ16722 [Drosophila virilis] |
| map05016 Huntington disease | Gene.8261 | 92 | PREDICTED: cytochrome c oxidase subunit 6C-like [Dendroctonus ponderosae] |
| map05016 Huntington disease | Gene.8261 | 88 | PREDICTED: cytochrome c oxidase subunit 6C-like [Dendroctonus ponderosae] |
| map05016 Huntington disease | Gene.8261 | 40 | PREDICTED: cytochrome c oxidase subunit 6C-like [Dendroctonus ponderosae] |
| map05016 Huntington disease | Gene.5746 | 137 | "PREDICTED: ATP synthase subunit delta, mitochondrial [Tribolium castaneum]" |
| map05016 Huntington disease | Gene.7676 | 53 | PREDICTED: guanine nucleotide-binding protein G(q) subunit alpha isoform X1 [Tribolium castaneum] |
| map05016 Huntington disease | Gene.5965 | 63 | "PREDICTED: superoxide dismutase [Mn] 1, mitochondrial-like [Aethina tumida]" |
| map05016 Huntington disease | Gene.5965 | 68 | "PREDICTED: superoxide dismutase [Mn] 1, mitochondrial-like [Aethina tumida]" |
| map05016 Huntington disease | Gene.8081 | 119 | "ATP synthase subunit gamma, mitochondrial [Leptinotarsa decemlineata]" |
| map05016 Huntington disease | Gene.8081 | 125 | "ATP synthase subunit gamma, mitochondrial [Leptinotarsa decemlineata]" |
| map05016 Huntington disease | Gene.8081 | 111 | "ATP synthase subunit gamma, mitochondrial [Leptinotarsa decemlineata]" |
| map05016 Huntington disease | Gene.8081 | 78 | "ATP synthase subunit gamma, mitochondrial [Leptinotarsa decemlineata]" |
| map05016 Huntington disease | Gene.8081 | 54 | "ATP synthase subunit gamma, mitochondrial [Leptinotarsa decemlineata]" |
| map05016 Huntington disease | Gene.6805 | 205 | peptidyl-prolyl cis-trans isomerase [Leptinotarsa decemlineata] |
| map05016 Huntington disease | Gene.6805 | 130 | peptidyl-prolyl cis-trans isomerase [Leptinotarsa decemlineata] |
| map05016 Huntington disease | Gene.6805 | 85 | peptidyl-prolyl cis-trans isomerase [Leptinotarsa decemlineata] |
| map05016 Huntington disease | Gene.6805 | 194 | peptidyl-prolyl cis-trans isomerase [Leptinotarsa decemlineata] |
| map05016 Huntington disease | Gene.6318 | 57 | NADH dehydrogenase [ubiquinone] 1 beta subcomplex subunit 3 [Leptinotarsa decemlineata] |
| map05016 Huntington disease | Gene.6318 | 43 | NADH dehydrogenase [ubiquinone] 1 beta subcomplex subunit 3 [Leptinotarsa decemlineata] |
| map05016 Huntington disease | Gene.6318 | 23 | NADH dehydrogenase [ubiquinone] 1 beta subcomplex subunit 3 [Leptinotarsa decemlineata] |
| map05016 Huntington disease | Gene.8337 | 50 | "PREDICTED: cytochrome c oxidase subunit 5B, mitochondrial [Tribolium castaneum]" |
| map05016 Huntington disease | Gene.8337 | 44 | "PREDICTED: cytochrome c oxidase subunit 5B, mitochondrial [Tribolium castaneum]" |
| map05016 Huntington disease | Gene.6425 | 116 | PREDICTED: clathrin light chain isoform X2 [Tribolium castaneum] |
| map05016 Huntington disease | Gene.6252 | 60 | NADH dehydrogenase [ubiquinone] 1 beta subcomplex subunit 4 [Leptinotarsa decemlineata] |
| map05016 Huntington disease | Gene.6252 | 111 | NADH dehydrogenase [ubiquinone] 1 beta subcomplex subunit 4 [Leptinotarsa decemlineata] |
| map05016 Huntington disease | Gene.2624 | 70 | PREDICTED: NADH dehydrogenase [ubiquinone] 1 alpha subcomplex subunit 5 [Tribolium castaneum] |
| map05016 Huntington disease | Gene.6262 | 104 | "probable NADH dehydrogenase [ubiquinone] flavoprotein 2, mitochondrial [Leptinotarsa decemlineata]" |
| map05016 Huntington disease | Gene.6262 | 230 | "probable NADH dehydrogenase [ubiquinone] flavoprotein 2, mitochondrial [Leptinotarsa decemlineata]" |
| map05016 Huntington disease | Gene.1073 | 88 | "PREDICTED: succinate dehydrogenase [ubiquinone] flavoprotein subunit, mitochondrial isoform X1 [Tribolium castaneum]" |
| map05016 Huntington disease | Gene.1073 | 529 | "PREDICTED: succinate dehydrogenase [ubiquinone] flavoprotein subunit, mitochondrial isoform X1 [Tribolium castaneum]" |
| map05016 Huntington disease | Gene.1073 | 546 | "PREDICTED: succinate dehydrogenase [ubiquinone] flavoprotein subunit, mitochondrial isoform X1 [Tribolium castaneum]" |
| map05016 Huntington disease | Gene.1073 | 175 | "PREDICTED: succinate dehydrogenase [ubiquinone] flavoprotein subunit, mitochondrial isoform X1 [Tribolium castaneum]" |
| map05016 Huntington disease | Gene.1073 | 632 | "PREDICTED: succinate dehydrogenase [ubiquinone] flavoprotein subunit, mitochondrial isoform X1 [Tribolium castaneum]" |
| map05016 Huntington disease | Gene.1073 | 543 | "PREDICTED: succinate dehydrogenase [ubiquinone] flavoprotein subunit, mitochondrial isoform X1 [Tribolium castaneum]" |
| map05016 Huntington disease | Gene.1073 | 331 | "PREDICTED: succinate dehydrogenase [ubiquinone] flavoprotein subunit, mitochondrial isoform X1 [Tribolium castaneum]" |
| map05016 Huntington disease | Gene.1912 | 120 | PREDICTED: NADH dehydrogenase [ubiquinone] 1 beta subcomplex subunit 9 [Aethina tumida] |
| map05016 Huntington disease | Gene.1912 | 51 | PREDICTED: NADH dehydrogenase [ubiquinone] 1 beta subcomplex subunit 9 [Aethina tumida] |
| map05016 Huntington disease | Gene.580 | 231 | "PREDICTED: cytochrome b-c1 complex subunit 2, mitochondrial [Tribolium castaneum]" |
| map05016 Huntington disease | Gene.580 | 248 | "PREDICTED: cytochrome b-c1 complex subunit 2, mitochondrial [Tribolium castaneum]" |
| map05016 Huntington disease | Gene.6169 | 53 | cytochrome b-c1 complex subunit 7-like [Anoplophora glabripennis] |
| map05016 Huntington disease | Gene.6169 | 77 | cytochrome b-c1 complex subunit 7-like [Anoplophora glabripennis] |
| map05016 Huntington disease | Gene.3481 | 53 | NADH dehydrogenase [ubiquinone] 1 alpha subcomplex subunit 8 [Asbolus verrucosus] |
| map05016 Huntington disease | Gene.2017 | 103 | "ATP synthase subunit O, mitochondrial [Asbolus verrucosus]" |
| map05016 Huntington disease | Gene.2017 | 182 | "ATP synthase subunit O, mitochondrial [Asbolus verrucosus]" |
| map05016 Huntington disease | Gene.2017 | 91 | "ATP synthase subunit O, mitochondrial [Asbolus verrucosus]" |
| map05016 Huntington disease | Gene.2017 | 121 | "ATP synthase subunit O, mitochondrial [Asbolus verrucosus]" |
| map05016 Huntington disease | Gene.2017 | 184 | "ATP synthase subunit O, mitochondrial [Asbolus verrucosus]" |
| map05016 Huntington disease | Gene.2522 | 46 | "cytochrome c oxidase subunit 5A, mitochondrial [Anoplophora glabripennis]" |
| map05016 Huntington disease | Gene.2522 | 101 | "cytochrome c oxidase subunit 5A, mitochondrial [Anoplophora glabripennis]" |
| map05016 Huntington disease | Gene.2522 | 51 | "cytochrome c oxidase subunit 5A, mitochondrial [Anoplophora glabripennis]" |
| map05016 Huntington disease | Gene.5767 | 89 | NADH dehydrogenase [ubiquinone] iron-sulfur protein 5 [Anoplophora glabripennis] |
| map05016 Huntington disease | Gene.4276 | 74 | "superoxide dismutase, partial [Harmonia axyridis]" |
| map05016 Huntington disease | Gene.4276 | 152 | "superoxide dismutase, partial [Harmonia axyridis]" |
| map05016 Huntington disease | Gene.4276 | 4 | "superoxide dismutase, partial [Harmonia axyridis]" |
| map05016 Huntington disease | Gene.4276 | 134 | "superoxide dismutase, partial [Harmonia axyridis]" |
| map05016 Huntington disease | Gene.4276 | 106 | "superoxide dismutase, partial [Harmonia axyridis]" |
| map05016 Huntington disease | Gene.8655 | 74 | "PREDICTED: transcription factor A, mitochondrial [Tribolium castaneum]" |
| map05016 Huntington disease | Gene.8655 | 203 | "PREDICTED: transcription factor A, mitochondrial [Tribolium castaneum]" |
| map05016 Huntington disease | Gene.8098 | 93 | "hypothetical protein, partial [Rhynchophorus ferrugineus]" |
| map05016 Huntington disease | Gene.8098 | 53 | "hypothetical protein, partial [Rhynchophorus ferrugineus]" |
| map05016 Huntington disease | Gene.8098 | 64 | "hypothetical protein, partial [Rhynchophorus ferrugineus]" |
| map05016 Huntington disease | Gene.8098 | 106 | "hypothetical protein, partial [Rhynchophorus ferrugineus]" |
| map05016 Huntington disease | Gene.8098 | 97 | "hypothetical protein, partial [Rhynchophorus ferrugineus]" |
| map05016 Huntington disease | Gene.8098 | 148 | "hypothetical protein, partial [Rhynchophorus ferrugineus]" |
| map05016 Huntington disease | Gene.8098 | 167 | "hypothetical protein, partial [Rhynchophorus ferrugineus]" |
| map05016 Huntington disease | Gene.8516 | 373 | PREDICTED: NADH-ubiquinone oxidoreductase 49 kDa subunit [Tribolium castaneum] |
| map05016 Huntington disease | Gene.8516 | 310 | PREDICTED: NADH-ubiquinone oxidoreductase 49 kDa subunit [Tribolium castaneum] |
| map05016 Huntington disease | Gene.8516 | 368 | PREDICTED: NADH-ubiquinone oxidoreductase 49 kDa subunit [Tribolium castaneum] |
| map05016 Huntington disease | Gene.8673 | 48 | "succinate dehydrogenase [ubiquinone] iron-sulfur subunit, mitochondrial, partial [Asbolus verrucosus]" |
| map05016 Huntington disease | Gene.3795 | 166 | "NADH-ubiquinone oxidoreductase 75 kDa subunit, mitochondrial [Asbolus verrucosus]" |
| map05016 Huntington disease | Gene.3795 | 172 | "NADH-ubiquinone oxidoreductase 75 kDa subunit, mitochondrial [Asbolus verrucosus]" |
| map05016 Huntington disease | Gene.3795 | 691 | "NADH-ubiquinone oxidoreductase 75 kDa subunit, mitochondrial [Asbolus verrucosus]" |
| map05016 Huntington disease | Gene.3795 | 626 | "NADH-ubiquinone oxidoreductase 75 kDa subunit, mitochondrial [Asbolus verrucosus]" |
| map05016 Huntington disease | Gene.3795 | 307 | "NADH-ubiquinone oxidoreductase 75 kDa subunit, mitochondrial [Asbolus verrucosus]" |
| map05016 Huntington disease | Gene.3795 | 541 | "NADH-ubiquinone oxidoreductase 75 kDa subunit, mitochondrial [Asbolus verrucosus]" |
| map05016 Huntington disease | Gene.3795 | 622 | "NADH-ubiquinone oxidoreductase 75 kDa subunit, mitochondrial [Asbolus verrucosus]" |
| map05016 Huntington disease | Gene.3063 | 178 | PREDICTED: uncharacterized protein LOC108560181 [Nicrophorus vespilloides] |
| map05016 Huntington disease | Gene.7134 | 95 | "PREDICTED: ATP synthase subunit d, mitochondrial [Tribolium castaneum]" |
| map05016 Huntington disease | Gene.7134 | 105 | "PREDICTED: ATP synthase subunit d, mitochondrial [Tribolium castaneum]" |
| map05016 Huntington disease | Gene.1846 | 205 | "ATP synthase subunit beta, mitochondrial [Tribolium castaneum]" |
| map05016 Huntington disease | Gene.1846 | 128 | "ATP synthase subunit beta, mitochondrial [Tribolium castaneum]" |
| map05016 Huntington disease | Gene.1846 | 497 | "ATP synthase subunit beta, mitochondrial [Tribolium castaneum]" |
| map05016 Huntington disease | Gene.1846 | 263 | "ATP synthase subunit beta, mitochondrial [Tribolium castaneum]" |
| map05016 Huntington disease | Gene.1846 | 268 | "ATP synthase subunit beta, mitochondrial [Tribolium castaneum]" |
| map05016 Huntington disease | Gene.1846 | 202 | "ATP synthase subunit beta, mitochondrial [Tribolium castaneum]" |
| map05016 Huntington disease | Gene.1846 | 430 | "ATP synthase subunit beta, mitochondrial [Tribolium castaneum]" |
| map05016 Huntington disease | Gene.6759 | 54 | hypothetical protein AMK59_2413 [Oryctes borbonicus] |
| map05016 Huntington disease | Gene.8771 | 54 | "PREDICTED: NADH dehydrogenase [ubiquinone] iron-sulfur protein 6, mitochondrial [Tribolium castaneum]" |
| map05016 Huntington disease | Gene.8407 | 27 | PREDICTED: voltage-dependent anion-selective channel isoform X1 [Tribolium castaneum] |
| map05016 Huntington disease | Gene.8407 | 11 | PREDICTED: voltage-dependent anion-selective channel isoform X1 [Tribolium castaneum] |
| map05016 Huntington disease | Gene.8407 | 60 | PREDICTED: voltage-dependent anion-selective channel isoform X1 [Tribolium castaneum] |
| map05016 Huntington disease | Gene.8407 | 64 | PREDICTED: voltage-dependent anion-selective channel isoform X1 [Tribolium castaneum] |
| map05164 Influenza A | Gene.8756 | 84 | uncharacterized protein Dvir_GJ16722 [Drosophila virilis] |
| map05164 Influenza A | Gene.8756 | 10 | uncharacterized protein Dvir_GJ16722 [Drosophila virilis] |
| map05164 Influenza A | Gene.1530 | 354 | PREDICTED: farnesyl pyrophosphate synthase-like [Atta cephalotes] |
| map05164 Influenza A | Gene.1530 | 260 | PREDICTED: farnesyl pyrophosphate synthase-like [Atta cephalotes] |
| map05164 Influenza A | Gene.1530 | 323 | PREDICTED: farnesyl pyrophosphate synthase-like [Atta cephalotes] |
| map05164 Influenza A | Gene.1530 | 187 | PREDICTED: farnesyl pyrophosphate synthase-like [Atta cephalotes] |
| map05164 Influenza A | Gene.5296 | 96 | eukaryotic translation initiation factor 2 subunit 1 [Anoplophora glabripennis] |
| map05164 Influenza A | Gene.5296 | 79 | eukaryotic translation initiation factor 2 subunit 1 [Anoplophora glabripennis] |
| map05164 Influenza A | Gene.6558 | 63 | PREDICTED: polyadenylate-binding protein 2 isoform X2 [Tribolium castaneum] |
| map05164 Influenza A | Gene.6558 | 133 | PREDICTED: polyadenylate-binding protein 2 isoform X2 [Tribolium castaneum] |
| map05164 Influenza A | Gene.5636 | 179 | ras-related protein Rab-11A [Anoplophora glabripennis] |
| map05164 Influenza A | Gene.8096 | 274 | "PREDICTED: ADP,ATP carrier protein 1 [Tribolium castaneum]" |
| map05164 Influenza A | Gene.8096 | 74 | "PREDICTED: ADP,ATP carrier protein 1 [Tribolium castaneum]" |
| map05164 Influenza A | Gene.8096 | 271 | "PREDICTED: ADP,ATP carrier protein 1 [Tribolium castaneum]" |
| map05164 Influenza A | Gene.8096 | 21 | "PREDICTED: ADP,ATP carrier protein 1 [Tribolium castaneum]" |
| map05164 Influenza A | Gene.8096 | 63 | "PREDICTED: ADP,ATP carrier protein 1 [Tribolium castaneum]" |
| map05164 Influenza A | Gene.8096 | 263 | "PREDICTED: ADP,ATP carrier protein 1 [Tribolium castaneum]" |
| map05164 Influenza A | Gene.8096 | 107 | "PREDICTED: ADP,ATP carrier protein 1 [Tribolium castaneum]" |
| map05164 Influenza A | Gene.8096 | 158 | "PREDICTED: ADP,ATP carrier protein 1 [Tribolium castaneum]" |
| map05164 Influenza A | Gene.8096 | 60 | "PREDICTED: ADP,ATP carrier protein 1 [Tribolium castaneum]" |
| map05164 Influenza A | Gene.8096 | 177 | "PREDICTED: ADP,ATP carrier protein 1 [Tribolium castaneum]" |
| map05164 Influenza A | Gene.8096 | 257 | "PREDICTED: ADP,ATP carrier protein 1 [Tribolium castaneum]" |
| map05164 Influenza A | Gene.8096 | 54 | "PREDICTED: ADP,ATP carrier protein 1 [Tribolium castaneum]" |
| map05164 Influenza A | Gene.172 | 216 | "beta-actin, partial [Cotesia chilonis]" |
| map05164 Influenza A | Gene.172 | 51 | "beta-actin, partial [Cotesia chilonis]" |
| map05164 Influenza A | Gene.172 | 114 | "beta-actin, partial [Cotesia chilonis]" |
| map05164 Influenza A | Gene.172 | 62 | "beta-actin, partial [Cotesia chilonis]" |
| map05164 Influenza A | Gene.8098 | 93 | "hypothetical protein, partial [Rhynchophorus ferrugineus]" |
| map05164 Influenza A | Gene.8098 | 53 | "hypothetical protein, partial [Rhynchophorus ferrugineus]" |
| map05164 Influenza A | Gene.8098 | 64 | "hypothetical protein, partial [Rhynchophorus ferrugineus]" |
| map05164 Influenza A | Gene.8098 | 106 | "hypothetical protein, partial [Rhynchophorus ferrugineus]" |
| map05164 Influenza A | Gene.8098 | 97 | "hypothetical protein, partial [Rhynchophorus ferrugineus]" |
| map05164 Influenza A | Gene.8098 | 148 | "hypothetical protein, partial [Rhynchophorus ferrugineus]" |
| map05164 Influenza A | Gene.8098 | 167 | "hypothetical protein, partial [Rhynchophorus ferrugineus]" |
| map05164 Influenza A | Gene.2646 | 99 | beta actin [Polyrhachis vicina] |
| map05164 Influenza A | Gene.2646 | 88 | beta actin [Polyrhachis vicina] |
| map05164 Influenza A | Gene.2646 | 64 | beta actin [Polyrhachis vicina] |
| map05164 Influenza A | Gene.2646 | 101 | beta actin [Polyrhachis vicina] |
| map04961 Endocrine and other factor-regulated calcium reabsorption | Gene.5709 | 26 | cAMP-dependent protein kinase catalytic subunit [Agrilus planipennis] |
| map04961 Endocrine and other factor-regulated calcium reabsorption | Gene.5709 | 257 | cAMP-dependent protein kinase catalytic subunit [Agrilus planipennis] |
| map04961 Endocrine and other factor-regulated calcium reabsorption | Gene.7676 | 53 | PREDICTED: guanine nucleotide-binding protein G(q) subunit alpha isoform X1 [Tribolium castaneum] |
| map04961 Endocrine and other factor-regulated calcium reabsorption | Gene.2599 | 765 | PREDICTED: sodium/potassium-transporting ATPase subunit alpha isoform X3 [Tribolium castaneum] |
| map04961 Endocrine and other factor-regulated calcium reabsorption | Gene.6425 | 116 | PREDICTED: clathrin light chain isoform X2 [Tribolium castaneum] |
| map04961 Endocrine and other factor-regulated calcium reabsorption | Gene.5636 | 179 | ras-related protein Rab-11A [Anoplophora glabripennis] |
| map04961 Endocrine and other factor-regulated calcium reabsorption | Gene.2830 | 84 | PREDICTED: LOW QUALITY PROTEIN: clathrin heavy chain [Aethina tumida] |
| map04961 Endocrine and other factor-regulated calcium reabsorption | Gene.2830 | 103 | PREDICTED: LOW QUALITY PROTEIN: clathrin heavy chain [Aethina tumida] |
| map04961 Endocrine and other factor-regulated calcium reabsorption | Gene.2830 | 902 | PREDICTED: LOW QUALITY PROTEIN: clathrin heavy chain [Aethina tumida] |
| map04961 Endocrine and other factor-regulated calcium reabsorption | Gene.2830 | 1619 | PREDICTED: LOW QUALITY PROTEIN: clathrin heavy chain [Aethina tumida] |
| map04961 Endocrine and other factor-regulated calcium reabsorption | Gene.901 | 772 | PREDICTED: plasma membrane calcium-transporting ATPase 2 isoform X2 [Tribolium castaneum] |
| map04961 Endocrine and other factor-regulated calcium reabsorption | Gene.901 | 166 | PREDICTED: plasma membrane calcium-transporting ATPase 2 isoform X2 [Tribolium castaneum] |
| map04961 Endocrine and other factor-regulated calcium reabsorption | Gene.901 | 16 | PREDICTED: plasma membrane calcium-transporting ATPase 2 isoform X2 [Tribolium castaneum] |
| map04740 Olfactory transduction | Gene.5709 | 26 | cAMP-dependent protein kinase catalytic subunit [Agrilus planipennis] |
| map04740 Olfactory transduction | Gene.5709 | 257 | cAMP-dependent protein kinase catalytic subunit [Agrilus planipennis] |
| map04740 Olfactory transduction | Gene.298 | 116 | "Csa-calmodulin 3, partial [Cupiennius salei]" |
| map04740 Olfactory transduction | Gene.298 | 95 | "Csa-calmodulin 3, partial [Cupiennius salei]" |
| map04740 Olfactory transduction | Gene.298 | 31 | "Csa-calmodulin 3, partial [Cupiennius salei]" |
| map04740 Olfactory transduction | Gene.298 | 78 | "Csa-calmodulin 3, partial [Cupiennius salei]" |
| map04740 Olfactory transduction | Gene.164 | 78 | PREDICTED: guanine nucleotide-binding protein subunit beta-1 [Tribolium castaneum] |
| map04740 Olfactory transduction | Gene.5803 | 20 | calcium-binding protein E63-1 [Anoplophora glabripennis] |
| map04933 AGE-RAGE signaling pathway in diabetic complications | Gene.7526 | 96 | ras-related protein Rac1 [Anoplophora glabripennis] |
| map04210 Apoptosis | Gene.8756 | 84 | uncharacterized protein Dvir_GJ16722 [Drosophila virilis] |
| map04210 Apoptosis | Gene.8756 | 10 | uncharacterized protein Dvir_GJ16722 [Drosophila virilis] |
| map04210 Apoptosis | Gene.5296 | 96 | eukaryotic translation initiation factor 2 subunit 1 [Anoplophora glabripennis] |
| map04210 Apoptosis | Gene.5296 | 79 | eukaryotic translation initiation factor 2 subunit 1 [Anoplophora glabripennis] |
| map04210 Apoptosis | Gene.1613 | 401 | Dper\GL12416-PA-like protein [Anopheles sinensis] |
| map04210 Apoptosis | Gene.1613 | 163 | Dper\GL12416-PA-like protein [Anopheles sinensis] |
| map04210 Apoptosis | Gene.1613 | 60 | Dper\GL12416-PA-like protein [Anopheles sinensis] |
| map04210 Apoptosis | Gene.1613 | 370 | Dper\GL12416-PA-like protein [Anopheles sinensis] |
| map04210 Apoptosis | Gene.1613 | 326 | Dper\GL12416-PA-like protein [Anopheles sinensis] |
| map04210 Apoptosis | Gene.1613 | 394 | Dper\GL12416-PA-like protein [Anopheles sinensis] |
| map04210 Apoptosis | Gene.1613 | 96 | Dper\GL12416-PA-like protein [Anopheles sinensis] |
| map04210 Apoptosis | Gene.1613 | 336 | Dper\GL12416-PA-like protein [Anopheles sinensis] |
| map04210 Apoptosis | Gene.1579 | 310 | putative gut cathepsin D-like aspartic protease [Callosobruchus maculatus] |
| map04210 Apoptosis | Gene.1579 | 118 | putative gut cathepsin D-like aspartic protease [Callosobruchus maculatus] |
| map04210 Apoptosis | Gene.77 | 200 | cathepsin L [Anoplophora glabripennis] |
| map04210 Apoptosis | Gene.77 | 57 | cathepsin L [Anoplophora glabripennis] |
| map04210 Apoptosis | Gene.77 | 134 | cathepsin L [Anoplophora glabripennis] |
| map04210 Apoptosis | Gene.77 | 36 | cathepsin L [Anoplophora glabripennis] |
| map04210 Apoptosis | Gene.77 | 216 | cathepsin L [Anoplophora glabripennis] |
| map04210 Apoptosis | Gene.77 | 211 | cathepsin L [Anoplophora glabripennis] |
| map04210 Apoptosis | Gene.4867 | 1455 | PREDICTED: spectrin alpha chain isoform X4 [Tribolium castaneum] |
| map04210 Apoptosis | Gene.4867 | 127 | PREDICTED: spectrin alpha chain isoform X4 [Tribolium castaneum] |
| map04210 Apoptosis | Gene.4867 | 2295 | PREDICTED: spectrin alpha chain isoform X4 [Tribolium castaneum] |
| map04210 Apoptosis | Gene.4867 | 370 | PREDICTED: spectrin alpha chain isoform X4 [Tribolium castaneum] |
| map04210 Apoptosis | Gene.4867 | 266 | PREDICTED: spectrin alpha chain isoform X4 [Tribolium castaneum] |
| map04210 Apoptosis | Gene.4867 | 287 | PREDICTED: spectrin alpha chain isoform X4 [Tribolium castaneum] |
| map04210 Apoptosis | Gene.4867 | 991 | PREDICTED: spectrin alpha chain isoform X4 [Tribolium castaneum] |
| map04210 Apoptosis | Gene.4867 | 734 | PREDICTED: spectrin alpha chain isoform X4 [Tribolium castaneum] |
| map04210 Apoptosis | Gene.4867 | 453 | PREDICTED: spectrin alpha chain isoform X4 [Tribolium castaneum] |
| map04210 Apoptosis | Gene.4867 | 2218 | PREDICTED: spectrin alpha chain isoform X4 [Tribolium castaneum] |
| map04210 Apoptosis | Gene.4867 | 851 | PREDICTED: spectrin alpha chain isoform X4 [Tribolium castaneum] |
| map04210 Apoptosis | Gene.4867 | 912 | PREDICTED: spectrin alpha chain isoform X4 [Tribolium castaneum] |
| map04210 Apoptosis | Gene.4867 | 1018 | PREDICTED: spectrin alpha chain isoform X4 [Tribolium castaneum] |
| map04210 Apoptosis | Gene.4867 | 2070 | PREDICTED: spectrin alpha chain isoform X4 [Tribolium castaneum] |
| map04210 Apoptosis | Gene.4867 | 1412 | PREDICTED: spectrin alpha chain isoform X4 [Tribolium castaneum] |
| map04210 Apoptosis | Gene.4867 | 1051 | PREDICTED: spectrin alpha chain isoform X4 [Tribolium castaneum] |
| map04210 Apoptosis | Gene.4867 | 1138 | PREDICTED: spectrin alpha chain isoform X4 [Tribolium castaneum] |
| map04210 Apoptosis | Gene.4867 | 1656 | PREDICTED: spectrin alpha chain isoform X4 [Tribolium castaneum] |
| map04210 Apoptosis | Gene.4867 | 1165 | PREDICTED: spectrin alpha chain isoform X4 [Tribolium castaneum] |
| map04210 Apoptosis | Gene.4867 | 1042 | PREDICTED: spectrin alpha chain isoform X4 [Tribolium castaneum] |
| map04210 Apoptosis | Gene.4867 | 356 | PREDICTED: spectrin alpha chain isoform X4 [Tribolium castaneum] |
| map04210 Apoptosis | Gene.4867 | 779 | PREDICTED: spectrin alpha chain isoform X4 [Tribolium castaneum] |
| map04210 Apoptosis | Gene.4867 | 247 | PREDICTED: spectrin alpha chain isoform X4 [Tribolium castaneum] |
| map04210 Apoptosis | Gene.4867 | 1922 | PREDICTED: spectrin alpha chain isoform X4 [Tribolium castaneum] |
| map04210 Apoptosis | Gene.1295 | 274 | PREDICTED: lamin Dm0 isoform X1 [Aethina tumida] |
| map04210 Apoptosis | Gene.1295 | 486 | PREDICTED: lamin Dm0 isoform X1 [Aethina tumida] |
| map04210 Apoptosis | Gene.1295 | 455 | PREDICTED: lamin Dm0 isoform X1 [Aethina tumida] |
| map04210 Apoptosis | Gene.1295 | 458 | PREDICTED: lamin Dm0 isoform X1 [Aethina tumida] |
| map04210 Apoptosis | Gene.1295 | 111 | PREDICTED: lamin Dm0 isoform X1 [Aethina tumida] |
| map04210 Apoptosis | Gene.1295 | 167 | PREDICTED: lamin Dm0 isoform X1 [Aethina tumida] |
| map04210 Apoptosis | Gene.1295 | 205 | PREDICTED: lamin Dm0 isoform X1 [Aethina tumida] |
| map04210 Apoptosis | Gene.1295 | 448 | PREDICTED: lamin Dm0 isoform X1 [Aethina tumida] |
| map04210 Apoptosis | Gene.1295 | 244 | PREDICTED: lamin Dm0 isoform X1 [Aethina tumida] |
| map04210 Apoptosis | Gene.1295 | 101 | PREDICTED: lamin Dm0 isoform X1 [Aethina tumida] |
| map04210 Apoptosis | Gene.1295 | 144 | PREDICTED: lamin Dm0 isoform X1 [Aethina tumida] |
| map04210 Apoptosis | Gene.1295 | 329 | PREDICTED: lamin Dm0 isoform X1 [Aethina tumida] |
| map04210 Apoptosis | Gene.4379 | 57 | cathepsin L precursor [Tribolium castaneum] |
| map04210 Apoptosis | Gene.2884 | 100 | PREDICTED: cathepsin L1 [Tribolium castaneum] |
| map04210 Apoptosis | Gene.272 | 395 | PREDICTED: aspartic proteinase A2 [Tribolium castaneum] |
| map04210 Apoptosis | Gene.272 | 344 | PREDICTED: aspartic proteinase A2 [Tribolium castaneum] |
| map04210 Apoptosis | Gene.272 | 239 | PREDICTED: aspartic proteinase A2 [Tribolium castaneum] |
| map04210 Apoptosis | Gene.6077 | 8 | "Spectrin, SH3 1, and/or DUF3584 domain containing protein, partial [Asbolus verrucosus]" |
| map04210 Apoptosis | Gene.6077 | 11 | "Spectrin, SH3 1, and/or DUF3584 domain containing protein, partial [Asbolus verrucosus]" |
| map04210 Apoptosis | Gene.6077 | 77 | "Spectrin, SH3 1, and/or DUF3584 domain containing protein, partial [Asbolus verrucosus]" |
| map04210 Apoptosis | Gene.6077 | 85 | "Spectrin, SH3 1, and/or DUF3584 domain containing protein, partial [Asbolus verrucosus]" |
| map04210 Apoptosis | Gene.6077 | 34 | "Spectrin, SH3 1, and/or DUF3584 domain containing protein, partial [Asbolus verrucosus]" |
| map04210 Apoptosis | Gene.4622 | 449 | putative cysteine proteinase CG12163 [Leptinotarsa decemlineata] |
| map04210 Apoptosis | Gene.6754 | 217 | "apoptosis-inducing factor 1, mitochondrial [Leptinotarsa decemlineata]" |
| map04210 Apoptosis | Gene.6754 | 543 | "apoptosis-inducing factor 1, mitochondrial [Leptinotarsa decemlineata]" |
| map04210 Apoptosis | Gene.172 | 216 | "beta-actin, partial [Cotesia chilonis]" |
| map04210 Apoptosis | Gene.172 | 51 | "beta-actin, partial [Cotesia chilonis]" |
| map04210 Apoptosis | Gene.172 | 114 | "beta-actin, partial [Cotesia chilonis]" |
| map04210 Apoptosis | Gene.172 | 62 | "beta-actin, partial [Cotesia chilonis]" |
| map04210 Apoptosis | Gene.1904 | 209 | PREDICTED: cathepsin B [Tribolium castaneum] |
| map04210 Apoptosis | Gene.2646 | 99 | beta actin [Polyrhachis vicina] |
| map04210 Apoptosis | Gene.2646 | 88 | beta actin [Polyrhachis vicina] |
| map04210 Apoptosis | Gene.2646 | 64 | beta actin [Polyrhachis vicina] |
| map04210 Apoptosis | Gene.2646 | 101 | beta actin [Polyrhachis vicina] |
| map04659 Th17 cell differentiation | Gene.7264 | 643 | "heat shock protein 90, partial [Harmonia axyridis]" |
| map04659 Th17 cell differentiation | Gene.7264 | 559 | "heat shock protein 90, partial [Harmonia axyridis]" |
| map04659 Th17 cell differentiation | Gene.7264 | 571 | "heat shock protein 90, partial [Harmonia axyridis]" |
| map04659 Th17 cell differentiation | Gene.7264 | 67 | "heat shock protein 90, partial [Harmonia axyridis]" |
| map04659 Th17 cell differentiation | Gene.7264 | 217 | "heat shock protein 90, partial [Harmonia axyridis]" |
| map04659 Th17 cell differentiation | Gene.7264 | 475 | "heat shock protein 90, partial [Harmonia axyridis]" |
| map04659 Th17 cell differentiation | Gene.7264 | 341 | "heat shock protein 90, partial [Harmonia axyridis]" |
| map04659 Th17 cell differentiation | Gene.7264 | 269 | "heat shock protein 90, partial [Harmonia axyridis]" |
| map04659 Th17 cell differentiation | Gene.7264 | 464 | "heat shock protein 90, partial [Harmonia axyridis]" |
| map04659 Th17 cell differentiation | Gene.7264 | 212 | "heat shock protein 90, partial [Harmonia axyridis]" |
| map04659 Th17 cell differentiation | Gene.7264 | 72 | "heat shock protein 90, partial [Harmonia axyridis]" |
| map04659 Th17 cell differentiation | Gene.7264 | 532 | "heat shock protein 90, partial [Harmonia axyridis]" |
| map04659 Th17 cell differentiation | Gene.7264 | 444 | "heat shock protein 90, partial [Harmonia axyridis]" |
| map04659 Th17 cell differentiation | Gene.7264 | 105 | "heat shock protein 90, partial [Harmonia axyridis]" |
| map04659 Th17 cell differentiation | Gene.7264 | 525 | "heat shock protein 90, partial [Harmonia axyridis]" |
| map04659 Th17 cell differentiation | Gene.7264 | 553 | "heat shock protein 90, partial [Harmonia axyridis]" |
| map04659 Th17 cell differentiation | Gene.7264 | 562 | "heat shock protein 90, partial [Harmonia axyridis]" |
| map04659 Th17 cell differentiation | Gene.7264 | 259 | "heat shock protein 90, partial [Harmonia axyridis]" |
| map04659 Th17 cell differentiation | Gene.7264 | 280 | "heat shock protein 90, partial [Harmonia axyridis]" |
| map04659 Th17 cell differentiation | Gene.7264 | 51 | "heat shock protein 90, partial [Harmonia axyridis]" |
| map04659 Th17 cell differentiation | Gene.7264 | 429 | "heat shock protein 90, partial [Harmonia axyridis]" |
| map04659 Th17 cell differentiation | Gene.7264 | 601 | "heat shock protein 90, partial [Harmonia axyridis]" |
| map04659 Th17 cell differentiation | Gene.7264 | 184 | "heat shock protein 90, partial [Harmonia axyridis]" |
| map04659 Th17 cell differentiation | Gene.7264 | 568 | "heat shock protein 90, partial [Harmonia axyridis]" |
| map04659 Th17 cell differentiation | Gene.7264 | 393 | "heat shock protein 90, partial [Harmonia axyridis]" |
| map04659 Th17 cell differentiation | Gene.7264 | 278 | "heat shock protein 90, partial [Harmonia axyridis]" |
| map04659 Th17 cell differentiation | Gene.7264 | 544 | "heat shock protein 90, partial [Harmonia axyridis]" |
| map04659 Th17 cell differentiation | Gene.7264 | 432 | "heat shock protein 90, partial [Harmonia axyridis]" |
| map04659 Th17 cell differentiation | Gene.7264 | 405 | "heat shock protein 90, partial [Harmonia axyridis]" |
| map04659 Th17 cell differentiation | Gene.7264 | 195 | "heat shock protein 90, partial [Harmonia axyridis]" |
| map04911 Insulin secretion | Gene.5709 | 26 | cAMP-dependent protein kinase catalytic subunit [Agrilus planipennis] |
| map04911 Insulin secretion | Gene.5709 | 257 | cAMP-dependent protein kinase catalytic subunit [Agrilus planipennis] |
| map04911 Insulin secretion | Gene.7676 | 53 | PREDICTED: guanine nucleotide-binding protein G(q) subunit alpha isoform X1 [Tribolium castaneum] |
| map04911 Insulin secretion | Gene.2599 | 765 | PREDICTED: sodium/potassium-transporting ATPase subunit alpha isoform X3 [Tribolium castaneum] |
| map04614 Renin-angiotensin system | Gene.68 | 468 | PREDICTED: prolyl endopeptidase isoform X1 [Tribolium castaneum] |
| map04614 Renin-angiotensin system | Gene.68 | 376 | PREDICTED: prolyl endopeptidase isoform X1 [Tribolium castaneum] |
| map04614 Renin-angiotensin system | Gene.4018 | 408 | glutamyl aminopeptidase-like isoform X2 [Leptinotarsa decemlineata] |
| map04614 Renin-angiotensin system | Gene.993 | 249 | puromycin-sensitive aminopeptidase-like protein isoform X1 [Anoplophora glabripennis] |
| map04612 Antigen processing and presentation | Gene.1917 | 93 | PREDICTED: calnexin [Tribolium castaneum] |
| map04612 Antigen processing and presentation | Gene.1917 | 502 | PREDICTED: calnexin [Tribolium castaneum] |
| map04612 Antigen processing and presentation | Gene.7276 | 170 | protein disulfide-isomerase A3 [Asbolus verrucosus] |
| map04612 Antigen processing and presentation | Gene.7276 | 134 | protein disulfide-isomerase A3 [Asbolus verrucosus] |
| map04612 Antigen processing and presentation | Gene.7276 | 165 | protein disulfide-isomerase A3 [Asbolus verrucosus] |
| map04612 Antigen processing and presentation | Gene.7276 | 210 | protein disulfide-isomerase A3 [Asbolus verrucosus] |
| map04612 Antigen processing and presentation | Gene.7276 | 78 | protein disulfide-isomerase A3 [Asbolus verrucosus] |
| map04612 Antigen processing and presentation | Gene.7276 | 268 | protein disulfide-isomerase A3 [Asbolus verrucosus] |
| map04612 Antigen processing and presentation | Gene.7276 | 449 | protein disulfide-isomerase A3 [Asbolus verrucosus] |
| map04612 Antigen processing and presentation | Gene.7276 | 62 | protein disulfide-isomerase A3 [Asbolus verrucosus] |
| map04612 Antigen processing and presentation | Gene.7276 | 247 | protein disulfide-isomerase A3 [Asbolus verrucosus] |
| map04612 Antigen processing and presentation | Gene.8135 | 359 | PREDICTED: calreticulin [Tribolium castaneum] |
| map04612 Antigen processing and presentation | Gene.8135 | 203 | PREDICTED: calreticulin [Tribolium castaneum] |
| map04612 Antigen processing and presentation | Gene.8135 | 206 | PREDICTED: calreticulin [Tribolium castaneum] |
| map04612 Antigen processing and presentation | Gene.8135 | 61 | PREDICTED: calreticulin [Tribolium castaneum] |
| map04612 Antigen processing and presentation | Gene.8135 | 108 | PREDICTED: calreticulin [Tribolium castaneum] |
| map04612 Antigen processing and presentation | Gene.8135 | 52 | PREDICTED: calreticulin [Tribolium castaneum] |
| map04612 Antigen processing and presentation | Gene.8135 | 354 | PREDICTED: calreticulin [Tribolium castaneum] |
| map04612 Antigen processing and presentation | Gene.8135 | 37 | PREDICTED: calreticulin [Tribolium castaneum] |
| map04612 Antigen processing and presentation | Gene.8135 | 45 | PREDICTED: calreticulin [Tribolium castaneum] |
| map04612 Antigen processing and presentation | Gene.8135 | 275 | PREDICTED: calreticulin [Tribolium castaneum] |
| map04612 Antigen processing and presentation | Gene.8135 | 31 | PREDICTED: calreticulin [Tribolium castaneum] |
| map04612 Antigen processing and presentation | Gene.77 | 200 | cathepsin L [Anoplophora glabripennis] |
| map04612 Antigen processing and presentation | Gene.77 | 57 | cathepsin L [Anoplophora glabripennis] |
| map04612 Antigen processing and presentation | Gene.77 | 134 | cathepsin L [Anoplophora glabripennis] |
| map04612 Antigen processing and presentation | Gene.77 | 36 | cathepsin L [Anoplophora glabripennis] |
| map04612 Antigen processing and presentation | Gene.77 | 216 | cathepsin L [Anoplophora glabripennis] |
| map04612 Antigen processing and presentation | Gene.77 | 211 | cathepsin L [Anoplophora glabripennis] |
| map04612 Antigen processing and presentation | Gene.2884 | 100 | PREDICTED: cathepsin L1 [Tribolium castaneum] |
| map04612 Antigen processing and presentation | Gene.4379 | 57 | cathepsin L precursor [Tribolium castaneum] |
| map04612 Antigen processing and presentation | Gene.7264 | 643 | "heat shock protein 90, partial [Harmonia axyridis]" |
| map04612 Antigen processing and presentation | Gene.7264 | 559 | "heat shock protein 90, partial [Harmonia axyridis]" |
| map04612 Antigen processing and presentation | Gene.7264 | 571 | "heat shock protein 90, partial [Harmonia axyridis]" |
| map04612 Antigen processing and presentation | Gene.7264 | 67 | "heat shock protein 90, partial [Harmonia axyridis]" |
| map04612 Antigen processing and presentation | Gene.7264 | 217 | "heat shock protein 90, partial [Harmonia axyridis]" |
| map04612 Antigen processing and presentation | Gene.7264 | 475 | "heat shock protein 90, partial [Harmonia axyridis]" |
| map04612 Antigen processing and presentation | Gene.7264 | 341 | "heat shock protein 90, partial [Harmonia axyridis]" |
| map04612 Antigen processing and presentation | Gene.7264 | 269 | "heat shock protein 90, partial [Harmonia axyridis]" |
| map04612 Antigen processing and presentation | Gene.7264 | 464 | "heat shock protein 90, partial [Harmonia axyridis]" |
| map04612 Antigen processing and presentation | Gene.7264 | 212 | "heat shock protein 90, partial [Harmonia axyridis]" |
| map04612 Antigen processing and presentation | Gene.7264 | 72 | "heat shock protein 90, partial [Harmonia axyridis]" |
| map04612 Antigen processing and presentation | Gene.7264 | 532 | "heat shock protein 90, partial [Harmonia axyridis]" |
| map04612 Antigen processing and presentation | Gene.7264 | 444 | "heat shock protein 90, partial [Harmonia axyridis]" |
| map04612 Antigen processing and presentation | Gene.7264 | 105 | "heat shock protein 90, partial [Harmonia axyridis]" |
| map04612 Antigen processing and presentation | Gene.7264 | 525 | "heat shock protein 90, partial [Harmonia axyridis]" |
| map04612 Antigen processing and presentation | Gene.7264 | 553 | "heat shock protein 90, partial [Harmonia axyridis]" |
| map04612 Antigen processing and presentation | Gene.7264 | 562 | "heat shock protein 90, partial [Harmonia axyridis]" |
| map04612 Antigen processing and presentation | Gene.7264 | 259 | "heat shock protein 90, partial [Harmonia axyridis]" |
| map04612 Antigen processing and presentation | Gene.7264 | 280 | "heat shock protein 90, partial [Harmonia axyridis]" |
| map04612 Antigen processing and presentation | Gene.7264 | 51 | "heat shock protein 90, partial [Harmonia axyridis]" |
| map04612 Antigen processing and presentation | Gene.7264 | 429 | "heat shock protein 90, partial [Harmonia axyridis]" |
| map04612 Antigen processing and presentation | Gene.7264 | 601 | "heat shock protein 90, partial [Harmonia axyridis]" |
| map04612 Antigen processing and presentation | Gene.7264 | 184 | "heat shock protein 90, partial [Harmonia axyridis]" |
| map04612 Antigen processing and presentation | Gene.7264 | 568 | "heat shock protein 90, partial [Harmonia axyridis]" |
| map04612 Antigen processing and presentation | Gene.7264 | 393 | "heat shock protein 90, partial [Harmonia axyridis]" |
| map04612 Antigen processing and presentation | Gene.7264 | 278 | "heat shock protein 90, partial [Harmonia axyridis]" |
| map04612 Antigen processing and presentation | Gene.7264 | 544 | "heat shock protein 90, partial [Harmonia axyridis]" |
| map04612 Antigen processing and presentation | Gene.7264 | 432 | "heat shock protein 90, partial [Harmonia axyridis]" |
| map04612 Antigen processing and presentation | Gene.7264 | 405 | "heat shock protein 90, partial [Harmonia axyridis]" |
| map04612 Antigen processing and presentation | Gene.7264 | 195 | "heat shock protein 90, partial [Harmonia axyridis]" |
| map04612 Antigen processing and presentation | Gene.8074 | 142 | PREDICTED: gamma-interferon-inducible lysosomal thiol reductase [Tribolium castaneum] |
| map04612 Antigen processing and presentation | Gene.8074 | 148 | PREDICTED: gamma-interferon-inducible lysosomal thiol reductase [Tribolium castaneum] |
| map04612 Antigen processing and presentation | Gene.8074 | 242 | PREDICTED: gamma-interferon-inducible lysosomal thiol reductase [Tribolium castaneum] |
| map04612 Antigen processing and presentation | Gene.5416 | 11 | PREDICTED: proteasome activator complex subunit 3 isoform X2 [Tribolium castaneum] |
| map04612 Antigen processing and presentation | Gene.5416 | 183 | PREDICTED: proteasome activator complex subunit 3 isoform X2 [Tribolium castaneum] |
| map04612 Antigen processing and presentation | Gene.1895 | 365 | heat shock protein 70 [Harmonia axyridis] |
| map04612 Antigen processing and presentation | Gene.1895 | 271 | heat shock protein 70 [Harmonia axyridis] |
| map04612 Antigen processing and presentation | Gene.1895 | 26 | heat shock protein 70 [Harmonia axyridis] |
| map04612 Antigen processing and presentation | Gene.1895 | 475 | heat shock protein 70 [Harmonia axyridis] |
| map04612 Antigen processing and presentation | Gene.1895 | 438 | heat shock protein 70 [Harmonia axyridis] |
| map04612 Antigen processing and presentation | Gene.1895 | 42 | heat shock protein 70 [Harmonia axyridis] |
| map04612 Antigen processing and presentation | Gene.1895 | 22 | heat shock protein 70 [Harmonia axyridis] |
| map04612 Antigen processing and presentation | Gene.1895 | 473 | heat shock protein 70 [Harmonia axyridis] |
| map04612 Antigen processing and presentation | Gene.1895 | 233 | heat shock protein 70 [Harmonia axyridis] |
| map04612 Antigen processing and presentation | Gene.1895 | 426 | heat shock protein 70 [Harmonia axyridis] |
| map04612 Antigen processing and presentation | Gene.1895 | 40 | heat shock protein 70 [Harmonia axyridis] |
| map04612 Antigen processing and presentation | Gene.1895 | 509 | heat shock protein 70 [Harmonia axyridis] |
| map04612 Antigen processing and presentation | Gene.1895 | 160 | heat shock protein 70 [Harmonia axyridis] |
| map04612 Antigen processing and presentation | Gene.1895 | 421 | heat shock protein 70 [Harmonia axyridis] |
| map04612 Antigen processing and presentation | Gene.1895 | 414 | heat shock protein 70 [Harmonia axyridis] |
| map04612 Antigen processing and presentation | Gene.1895 | 242 | heat shock protein 70 [Harmonia axyridis] |
| map04612 Antigen processing and presentation | Gene.1904 | 209 | PREDICTED: cathepsin B [Tribolium castaneum] |
| map04612 Antigen processing and presentation | Gene.2309 | 138 | Gamma-interferon-inducible lysosomal thiol reductase-like Protein [Tribolium castaneum] |
| map04971 Gastric acid secretion | Gene.5709 | 26 | cAMP-dependent protein kinase catalytic subunit [Agrilus planipennis] |
| map04971 Gastric acid secretion | Gene.5709 | 257 | cAMP-dependent protein kinase catalytic subunit [Agrilus planipennis] |
| map04971 Gastric acid secretion | Gene.298 | 116 | "Csa-calmodulin 3, partial [Cupiennius salei]" |
| map04971 Gastric acid secretion | Gene.298 | 95 | "Csa-calmodulin 3, partial [Cupiennius salei]" |
| map04971 Gastric acid secretion | Gene.298 | 31 | "Csa-calmodulin 3, partial [Cupiennius salei]" |
| map04971 Gastric acid secretion | Gene.298 | 78 | "Csa-calmodulin 3, partial [Cupiennius salei]" |
| map04971 Gastric acid secretion | Gene.7676 | 53 | PREDICTED: guanine nucleotide-binding protein G(q) subunit alpha isoform X1 [Tribolium castaneum] |
| map04971 Gastric acid secretion | Gene.5803 | 20 | calcium-binding protein E63-1 [Anoplophora glabripennis] |
| map04971 Gastric acid secretion | Gene.2599 | 765 | PREDICTED: sodium/potassium-transporting ATPase subunit alpha isoform X3 [Tribolium castaneum] |
| map04927 Cortisol synthesis and secretion | Gene.5709 | 26 | cAMP-dependent protein kinase catalytic subunit [Agrilus planipennis] |
| map04927 Cortisol synthesis and secretion | Gene.5709 | 257 | cAMP-dependent protein kinase catalytic subunit [Agrilus planipennis] |
| map04927 Cortisol synthesis and secretion | Gene.7676 | 53 | PREDICTED: guanine nucleotide-binding protein G(q) subunit alpha isoform X1 [Tribolium castaneum] |
| map04927 Cortisol synthesis and secretion | Gene.3807 | 269 | scavenger receptor protein [Tribolium castaneum] |
| map04927 Cortisol synthesis and secretion | Gene.3807 | 180 | scavenger receptor protein [Tribolium castaneum] |
| map04927 Cortisol synthesis and secretion | Gene.3807 | 344 | scavenger receptor protein [Tribolium castaneum] |
| map04927 Cortisol synthesis and secretion | Gene.3807 | 544 | scavenger receptor protein [Tribolium castaneum] |
| map04927 Cortisol synthesis and secretion | Gene.3807 | 355 | scavenger receptor protein [Tribolium castaneum] |
| map04914 Progesterone-mediated oocyte maturation | Gene.5709 | 26 | cAMP-dependent protein kinase catalytic subunit [Agrilus planipennis] |
| map04914 Progesterone-mediated oocyte maturation | Gene.5709 | 257 | cAMP-dependent protein kinase catalytic subunit [Agrilus planipennis] |
| map04914 Progesterone-mediated oocyte maturation | Gene.7264 | 643 | "heat shock protein 90, partial [Harmonia axyridis]" |
| map04914 Progesterone-mediated oocyte maturation | Gene.7264 | 559 | "heat shock protein 90, partial [Harmonia axyridis]" |
| map04914 Progesterone-mediated oocyte maturation | Gene.7264 | 571 | "heat shock protein 90, partial [Harmonia axyridis]" |
| map04914 Progesterone-mediated oocyte maturation | Gene.7264 | 67 | "heat shock protein 90, partial [Harmonia axyridis]" |
| map04914 Progesterone-mediated oocyte maturation | Gene.7264 | 217 | "heat shock protein 90, partial [Harmonia axyridis]" |
| map04914 Progesterone-mediated oocyte maturation | Gene.7264 | 475 | "heat shock protein 90, partial [Harmonia axyridis]" |
| map04914 Progesterone-mediated oocyte maturation | Gene.7264 | 341 | "heat shock protein 90, partial [Harmonia axyridis]" |
| map04914 Progesterone-mediated oocyte maturation | Gene.7264 | 269 | "heat shock protein 90, partial [Harmonia axyridis]" |
| map04914 Progesterone-mediated oocyte maturation | Gene.7264 | 464 | "heat shock protein 90, partial [Harmonia axyridis]" |
| map04914 Progesterone-mediated oocyte maturation | Gene.7264 | 212 | "heat shock protein 90, partial [Harmonia axyridis]" |
| map04914 Progesterone-mediated oocyte maturation | Gene.7264 | 72 | "heat shock protein 90, partial [Harmonia axyridis]" |
| map04914 Progesterone-mediated oocyte maturation | Gene.7264 | 532 | "heat shock protein 90, partial [Harmonia axyridis]" |
| map04914 Progesterone-mediated oocyte maturation | Gene.7264 | 444 | "heat shock protein 90, partial [Harmonia axyridis]" |
| map04914 Progesterone-mediated oocyte maturation | Gene.7264 | 105 | "heat shock protein 90, partial [Harmonia axyridis]" |
| map04914 Progesterone-mediated oocyte maturation | Gene.7264 | 525 | "heat shock protein 90, partial [Harmonia axyridis]" |
| map04914 Progesterone-mediated oocyte maturation | Gene.7264 | 553 | "heat shock protein 90, partial [Harmonia axyridis]" |
| map04914 Progesterone-mediated oocyte maturation | Gene.7264 | 562 | "heat shock protein 90, partial [Harmonia axyridis]" |
| map04914 Progesterone-mediated oocyte maturation | Gene.7264 | 259 | "heat shock protein 90, partial [Harmonia axyridis]" |
| map04914 Progesterone-mediated oocyte maturation | Gene.7264 | 280 | "heat shock protein 90, partial [Harmonia axyridis]" |
| map04914 Progesterone-mediated oocyte maturation | Gene.7264 | 51 | "heat shock protein 90, partial [Harmonia axyridis]" |
| map04914 Progesterone-mediated oocyte maturation | Gene.7264 | 429 | "heat shock protein 90, partial [Harmonia axyridis]" |
| map04914 Progesterone-mediated oocyte maturation | Gene.7264 | 601 | "heat shock protein 90, partial [Harmonia axyridis]" |
| map04914 Progesterone-mediated oocyte maturation | Gene.7264 | 184 | "heat shock protein 90, partial [Harmonia axyridis]" |
| map04914 Progesterone-mediated oocyte maturation | Gene.7264 | 568 | "heat shock protein 90, partial [Harmonia axyridis]" |
| map04914 Progesterone-mediated oocyte maturation | Gene.7264 | 393 | "heat shock protein 90, partial [Harmonia axyridis]" |
| map04914 Progesterone-mediated oocyte maturation | Gene.7264 | 278 | "heat shock protein 90, partial [Harmonia axyridis]" |
| map04914 Progesterone-mediated oocyte maturation | Gene.7264 | 544 | "heat shock protein 90, partial [Harmonia axyridis]" |
| map04914 Progesterone-mediated oocyte maturation | Gene.7264 | 432 | "heat shock protein 90, partial [Harmonia axyridis]" |
| map04914 Progesterone-mediated oocyte maturation | Gene.7264 | 405 | "heat shock protein 90, partial [Harmonia axyridis]" |
| map04914 Progesterone-mediated oocyte maturation | Gene.7264 | 195 | "heat shock protein 90, partial [Harmonia axyridis]" |
| map04510 Focal adhesion | Gene.7526 | 96 | ras-related protein Rac1 [Anoplophora glabripennis] |
| map04510 Focal adhesion | Gene.2958 | 401 | alpha actinin [Coleomegilla maculata] |
| map04510 Focal adhesion | Gene.2958 | 763 | alpha actinin [Coleomegilla maculata] |
| map04510 Focal adhesion | Gene.502 | 147 | "acid phosphatase, partial [Cryptolaemus montrouzieri]" |
| map04510 Focal adhesion | Gene.502 | 141 | "acid phosphatase, partial [Cryptolaemus montrouzieri]" |
| map04510 Focal adhesion | Gene.502 | 260 | "acid phosphatase, partial [Cryptolaemus montrouzieri]" |
| map04510 Focal adhesion | Gene.2368 | 2143 | PREDICTED: filamin-A isoform X6 [Tribolium castaneum] |
| map04510 Focal adhesion | Gene.2368 | 1801 | PREDICTED: filamin-A isoform X6 [Tribolium castaneum] |
| map04510 Focal adhesion | Gene.2368 | 585 | PREDICTED: filamin-A isoform X6 [Tribolium castaneum] |
| map04510 Focal adhesion | Gene.2368 | 1534 | PREDICTED: filamin-A isoform X6 [Tribolium castaneum] |
| map04510 Focal adhesion | Gene.2368 | 2141 | PREDICTED: filamin-A isoform X6 [Tribolium castaneum] |
| map04510 Focal adhesion | Gene.2368 | 49 | PREDICTED: filamin-A isoform X6 [Tribolium castaneum] |
| map04510 Focal adhesion | Gene.2368 | 867 | PREDICTED: filamin-A isoform X6 [Tribolium castaneum] |
| map04510 Focal adhesion | Gene.2368 | 2100 | PREDICTED: filamin-A isoform X6 [Tribolium castaneum] |
| map04510 Focal adhesion | Gene.5154 | 2626 | PREDICTED: hemocytin isoform X2 [Tribolium castaneum] |
| map04510 Focal adhesion | Gene.5154 | 2001 | PREDICTED: hemocytin isoform X2 [Tribolium castaneum] |
| map04510 Focal adhesion | Gene.1845 | 92 | PREDICTED: glycogen synthase kinase-3 beta isoform X10 [Tribolium castaneum] |
| map04510 Focal adhesion | Gene.1845 | 87 | PREDICTED: glycogen synthase kinase-3 beta isoform X10 [Tribolium castaneum] |
| map04510 Focal adhesion | Gene.172 | 216 | "beta-actin, partial [Cotesia chilonis]" |
| map04510 Focal adhesion | Gene.172 | 51 | "beta-actin, partial [Cotesia chilonis]" |
| map04510 Focal adhesion | Gene.172 | 114 | "beta-actin, partial [Cotesia chilonis]" |
| map04510 Focal adhesion | Gene.172 | 62 | "beta-actin, partial [Cotesia chilonis]" |
| map04510 Focal adhesion | Gene.4326 | 171 | hemocytin [Agrilus planipennis] |
| map04510 Focal adhesion | Gene.4326 | 62 | hemocytin [Agrilus planipennis] |
| map04510 Focal adhesion | Gene.4842 | 1047 | PREDICTED: laminin subunit gamma-1 isoform X1 [Tribolium castaneum] |
| map04510 Focal adhesion | Gene.2646 | 99 | beta actin [Polyrhachis vicina] |
| map04510 Focal adhesion | Gene.2646 | 88 | beta actin [Polyrhachis vicina] |
| map04510 Focal adhesion | Gene.2646 | 64 | beta actin [Polyrhachis vicina] |
| map04510 Focal adhesion | Gene.2646 | 101 | beta actin [Polyrhachis vicina] |
| map04066 HIF-1 signaling pathway | Gene.7815 | 345 | "probable pyruvate dehydrogenase E1 component subunit alpha, mitochondrial isoform X2 [Leptinotarsa decemlineata]" |
| map04066 HIF-1 signaling pathway | Gene.7815 | 315 | "probable pyruvate dehydrogenase E1 component subunit alpha, mitochondrial isoform X2 [Leptinotarsa decemlineata]" |
| map04066 HIF-1 signaling pathway | Gene.7815 | 317 | "probable pyruvate dehydrogenase E1 component subunit alpha, mitochondrial isoform X2 [Leptinotarsa decemlineata]" |
| map04066 HIF-1 signaling pathway | Gene.7815 | 77 | "probable pyruvate dehydrogenase E1 component subunit alpha, mitochondrial isoform X2 [Leptinotarsa decemlineata]" |
| map04066 HIF-1 signaling pathway | Gene.7815 | 330 | "probable pyruvate dehydrogenase E1 component subunit alpha, mitochondrial isoform X2 [Leptinotarsa decemlineata]" |
| map04066 HIF-1 signaling pathway | Gene.1633 | 147 | PREDICTED: fructose-bisphosphate aldolase [Tribolium castaneum] |
| map04066 HIF-1 signaling pathway | Gene.1633 | 42 | PREDICTED: fructose-bisphosphate aldolase [Tribolium castaneum] |
| map04066 HIF-1 signaling pathway | Gene.1633 | 28 | PREDICTED: fructose-bisphosphate aldolase [Tribolium castaneum] |
| map04066 HIF-1 signaling pathway | Gene.1533 | 280 | PREDICTED: hexokinase type 2 isoform X2 [Tribolium castaneum] |
| map04066 HIF-1 signaling pathway | Gene.1533 | 73 | PREDICTED: hexokinase type 2 isoform X2 [Tribolium castaneum] |
| map04066 HIF-1 signaling pathway | Gene.572 | 84 | PREDICTED: phosphoglycerate kinase [Tribolium castaneum] |
| map04066 HIF-1 signaling pathway | Gene.572 | 89 | PREDICTED: phosphoglycerate kinase [Tribolium castaneum] |
| map04066 HIF-1 signaling pathway | Gene.572 | 16 | PREDICTED: phosphoglycerate kinase [Tribolium castaneum] |
| map04066 HIF-1 signaling pathway | Gene.572 | 351 | PREDICTED: phosphoglycerate kinase [Tribolium castaneum] |
| map04066 HIF-1 signaling pathway | Gene.572 | 10 | PREDICTED: phosphoglycerate kinase [Tribolium castaneum] |
| map04066 HIF-1 signaling pathway | Gene.572 | 323 | PREDICTED: phosphoglycerate kinase [Tribolium castaneum] |
| map04066 HIF-1 signaling pathway | Gene.572 | 129 | PREDICTED: phosphoglycerate kinase [Tribolium castaneum] |
| map04066 HIF-1 signaling pathway | Gene.572 | 5 | PREDICTED: phosphoglycerate kinase [Tribolium castaneum] |
| map04066 HIF-1 signaling pathway | Gene.3972 | 228 | "pyruvate dehydrogenase E1 component subunit beta, mitochondrial [Leptinotarsa decemlineata]" |
| map04066 HIF-1 signaling pathway | Gene.3972 | 185 | "pyruvate dehydrogenase E1 component subunit beta, mitochondrial [Leptinotarsa decemlineata]" |
| map04066 HIF-1 signaling pathway | Gene.3972 | 260 | "pyruvate dehydrogenase E1 component subunit beta, mitochondrial [Leptinotarsa decemlineata]" |
| map04066 HIF-1 signaling pathway | Gene.1891 | 103 | hypothetical protein AMK59_3112 [Oryctes borbonicus] |
| map04066 HIF-1 signaling pathway | Gene.1891 | 88 | hypothetical protein AMK59_3112 [Oryctes borbonicus] |
| map04066 HIF-1 signaling pathway | Gene.1891 | 71 | hypothetical protein AMK59_3112 [Oryctes borbonicus] |
| map04066 HIF-1 signaling pathway | Gene.1891 | 96 | hypothetical protein AMK59_3112 [Oryctes borbonicus] |
| map04066 HIF-1 signaling pathway | Gene.919 | 149 | ribosomal protein S6 [Chrysomela tremula] |
| map04066 HIF-1 signaling pathway | Gene.919 | 159 | ribosomal protein S6 [Chrysomela tremula] |
| map04066 HIF-1 signaling pathway | Gene.919 | 165 | ribosomal protein S6 [Chrysomela tremula] |
| map04066 HIF-1 signaling pathway | Gene.919 | 51 | ribosomal protein S6 [Chrysomela tremula] |
| map04066 HIF-1 signaling pathway | Gene.919 | 58 | ribosomal protein S6 [Chrysomela tremula] |
| map04066 HIF-1 signaling pathway | Gene.919 | 211 | ribosomal protein S6 [Chrysomela tremula] |
| map04066 HIF-1 signaling pathway | Gene.919 | 14 | ribosomal protein S6 [Chrysomela tremula] |
| map04066 HIF-1 signaling pathway | Gene.919 | 23 | ribosomal protein S6 [Chrysomela tremula] |
| map04066 HIF-1 signaling pathway | Gene.2375 | 84 | "glyceraldehyde-3-phosphate, partial [Harmonia axyridis]" |
| map04066 HIF-1 signaling pathway | Gene.2375 | 252 | "glyceraldehyde-3-phosphate, partial [Harmonia axyridis]" |
| map04066 HIF-1 signaling pathway | Gene.2375 | 90 | "glyceraldehyde-3-phosphate, partial [Harmonia axyridis]" |
| map04066 HIF-1 signaling pathway | Gene.2375 | 228 | "glyceraldehyde-3-phosphate, partial [Harmonia axyridis]" |
| map04066 HIF-1 signaling pathway | Gene.2375 | 330 | "glyceraldehyde-3-phosphate, partial [Harmonia axyridis]" |
| map04066 HIF-1 signaling pathway | Gene.2375 | 58 | "glyceraldehyde-3-phosphate, partial [Harmonia axyridis]" |
| map04066 HIF-1 signaling pathway | Gene.2375 | 163 | "glyceraldehyde-3-phosphate, partial [Harmonia axyridis]" |
| map04066 HIF-1 signaling pathway | Gene.2375 | 66 | "glyceraldehyde-3-phosphate, partial [Harmonia axyridis]" |
| map04066 HIF-1 signaling pathway | Gene.2375 | 73 | "glyceraldehyde-3-phosphate, partial [Harmonia axyridis]" |
| map04066 HIF-1 signaling pathway | Gene.2375 | 260 | "glyceraldehyde-3-phosphate, partial [Harmonia axyridis]" |
| map04066 HIF-1 signaling pathway | Gene.2375 | 264 | "glyceraldehyde-3-phosphate, partial [Harmonia axyridis]" |
| map04066 HIF-1 signaling pathway | Gene.2375 | 223 | "glyceraldehyde-3-phosphate, partial [Harmonia axyridis]" |
| map04066 HIF-1 signaling pathway | Gene.2375 | 195 | "glyceraldehyde-3-phosphate, partial [Harmonia axyridis]" |
| map04066 HIF-1 signaling pathway | Gene.1892 | 205 | PREDICTED: enolase [Musca domestica] |
| map04066 HIF-1 signaling pathway | Gene.1892 | 74 | PREDICTED: enolase [Musca domestica] |
| map04066 HIF-1 signaling pathway | Gene.1892 | 221 | PREDICTED: enolase [Musca domestica] |
| map04066 HIF-1 signaling pathway | Gene.1892 | 225 | PREDICTED: enolase [Musca domestica] |
| map04066 HIF-1 signaling pathway | Gene.1892 | 66 | PREDICTED: enolase [Musca domestica] |
| map04066 HIF-1 signaling pathway | Gene.1892 | 80 | PREDICTED: enolase [Musca domestica] |
| map04066 HIF-1 signaling pathway | Gene.1892 | 133 | PREDICTED: enolase [Musca domestica] |
| map01524 Platinum drug resistance | Gene.8382 | 77 | GST [Lygus lineolaris] |
| map01524 Platinum drug resistance | Gene.8382 | 80 | GST [Lygus lineolaris] |
| map01524 Platinum drug resistance | Gene.8382 | 31 | GST [Lygus lineolaris] |
| map01524 Platinum drug resistance | Gene.8756 | 84 | uncharacterized protein Dvir_GJ16722 [Drosophila virilis] |
| map01524 Platinum drug resistance | Gene.8756 | 10 | uncharacterized protein Dvir_GJ16722 [Drosophila virilis] |
| map01524 Platinum drug resistance | Gene.9040 | 57 | PREDICTED: microsomal glutathione S-transferase 1 [Tribolium castaneum] |
| map01524 Platinum drug resistance | Gene.9040 | 55 | PREDICTED: microsomal glutathione S-transferase 1 [Tribolium castaneum] |
| map01524 Platinum drug resistance | Gene.425 | 128 | glutathione S-transferase 1-1 [Anoplophora glabripennis] |
| map01524 Platinum drug resistance | Gene.425 | 215 | glutathione S-transferase 1-1 [Anoplophora glabripennis] |
| map01524 Platinum drug resistance | Gene.425 | 131 | glutathione S-transferase 1-1 [Anoplophora glabripennis] |
| map01524 Platinum drug resistance | Gene.425 | 201 | glutathione S-transferase 1-1 [Anoplophora glabripennis] |
| map01524 Platinum drug resistance | Gene.425 | 207 | glutathione S-transferase 1-1 [Anoplophora glabripennis] |
| map01524 Platinum drug resistance | Gene.425 | 37 | glutathione S-transferase 1-1 [Anoplophora glabripennis] |
| map01524 Platinum drug resistance | Gene.425 | 186 | glutathione S-transferase 1-1 [Anoplophora glabripennis] |
| map05120 Epithelial cell signaling in Helicobacter pylori infection | Gene.2318 | 109 | PREDICTED: LOW QUALITY PROTEIN: V-type proton ATPase 116 kDa subunit a-like [Aethina tumida] |
| map05120 Epithelial cell signaling in Helicobacter pylori infection | Gene.2318 | 50 | PREDICTED: LOW QUALITY PROTEIN: V-type proton ATPase 116 kDa subunit a-like [Aethina tumida] |
| map05120 Epithelial cell signaling in Helicobacter pylori infection | Gene.2318 | 522 | PREDICTED: LOW QUALITY PROTEIN: V-type proton ATPase 116 kDa subunit a-like [Aethina tumida] |
| map05120 Epithelial cell signaling in Helicobacter pylori infection | Gene.2318 | 76 | PREDICTED: LOW QUALITY PROTEIN: V-type proton ATPase 116 kDa subunit a-like [Aethina tumida] |
| map05120 Epithelial cell signaling in Helicobacter pylori infection | Gene.2318 | 266 | PREDICTED: LOW QUALITY PROTEIN: V-type proton ATPase 116 kDa subunit a-like [Aethina tumida] |
| map05120 Epithelial cell signaling in Helicobacter pylori infection | Gene.6879 | 42 | V-type proton ATPase subunit E [Leptinotarsa decemlineata] |
| map05120 Epithelial cell signaling in Helicobacter pylori infection | Gene.6879 | 59 | V-type proton ATPase subunit E [Leptinotarsa decemlineata] |
| map05120 Epithelial cell signaling in Helicobacter pylori infection | Gene.6879 | 156 | V-type proton ATPase subunit E [Leptinotarsa decemlineata] |
| map05120 Epithelial cell signaling in Helicobacter pylori infection | Gene.6879 | 10 | V-type proton ATPase subunit E [Leptinotarsa decemlineata] |
| map05120 Epithelial cell signaling in Helicobacter pylori infection | Gene.6879 | 68 | V-type proton ATPase subunit E [Leptinotarsa decemlineata] |
| map05120 Epithelial cell signaling in Helicobacter pylori infection | Gene.7526 | 96 | ras-related protein Rac1 [Anoplophora glabripennis] |
| map05120 Epithelial cell signaling in Helicobacter pylori infection | Gene.4190 | 409 | V-type proton ATPase subunit H [Asbolus verrucosus] |
| map05120 Epithelial cell signaling in Helicobacter pylori infection | Gene.246 | 39 | PREDICTED: V-type proton ATPase subunit d [Tribolium castaneum] |
| map05120 Epithelial cell signaling in Helicobacter pylori infection | Gene.5868 | 50 | PREDICTED: V-type proton ATPase subunit D [Polistes canadensis] |
| map05120 Epithelial cell signaling in Helicobacter pylori infection | Gene.5815 | 276 | PREDICTED: uncharacterized protein LOC663029 [Tribolium castaneum] |
| map05120 Epithelial cell signaling in Helicobacter pylori infection | Gene.5815 | 279 | PREDICTED: uncharacterized protein LOC663029 [Tribolium castaneum] |
| map05120 Epithelial cell signaling in Helicobacter pylori infection | Gene.5815 | 410 | PREDICTED: uncharacterized protein LOC663029 [Tribolium castaneum] |
| map05120 Epithelial cell signaling in Helicobacter pylori infection | Gene.5862 | 8 | "V-type proton ATPase subunit F, partial [Asbolus verrucosus]" |
| map05120 Epithelial cell signaling in Helicobacter pylori infection | Gene.5862 | 106 | "V-type proton ATPase subunit F, partial [Asbolus verrucosus]" |
| map05120 Epithelial cell signaling in Helicobacter pylori infection | Gene.7366 | 21 | PREDICTED: V-type proton ATPase subunit G [Nicrophorus vespilloides] |
| map05120 Epithelial cell signaling in Helicobacter pylori infection | Gene.7366 | 61 | PREDICTED: V-type proton ATPase subunit G [Nicrophorus vespilloides] |
| map05120 Epithelial cell signaling in Helicobacter pylori infection | Gene.7366 | 37 | PREDICTED: V-type proton ATPase subunit G [Nicrophorus vespilloides] |
| map05120 Epithelial cell signaling in Helicobacter pylori infection | Gene.1524 | 445 | V-type proton ATPase subunit B [Galleria mellonella] |
| map05120 Epithelial cell signaling in Helicobacter pylori infection | Gene.6297 | 155 | PREDICTED: V-type proton ATPase subunit C isoform X3 [Tribolium castaneum] |
| map05120 Epithelial cell signaling in Helicobacter pylori infection | Gene.6297 | 139 | PREDICTED: V-type proton ATPase subunit C isoform X3 [Tribolium castaneum] |
| map05120 Epithelial cell signaling in Helicobacter pylori infection | Gene.6297 | 267 | PREDICTED: V-type proton ATPase subunit C isoform X3 [Tribolium castaneum] |
| map05120 Epithelial cell signaling in Helicobacter pylori infection | Gene.6297 | 262 | PREDICTED: V-type proton ATPase subunit C isoform X3 [Tribolium castaneum] |
| map05120 Epithelial cell signaling in Helicobacter pylori infection | Gene.664 | 50 | PREDICTED: V-type proton ATPase 116 kDa subunit a isoform 1 isoform X3 [Tribolium castaneum] |
| map05120 Epithelial cell signaling in Helicobacter pylori infection | Gene.3482 | 588 | V-type proton ATPase catalytic subunit A [Leptinotarsa decemlineata] |
| map05120 Epithelial cell signaling in Helicobacter pylori infection | Gene.3482 | 533 | V-type proton ATPase catalytic subunit A [Leptinotarsa decemlineata] |
| map05120 Epithelial cell signaling in Helicobacter pylori infection | Gene.3482 | 130 | V-type proton ATPase catalytic subunit A [Leptinotarsa decemlineata] |
| map05120 Epithelial cell signaling in Helicobacter pylori infection | Gene.3482 | 593 | V-type proton ATPase catalytic subunit A [Leptinotarsa decemlineata] |
| map05120 Epithelial cell signaling in Helicobacter pylori infection | Gene.3482 | 584 | V-type proton ATPase catalytic subunit A [Leptinotarsa decemlineata] |
| map05120 Epithelial cell signaling in Helicobacter pylori infection | Gene.3482 | 513 | V-type proton ATPase catalytic subunit A [Leptinotarsa decemlineata] |
| map00130 Ubiquinone and other terpenoid-quinone biosynthesis | Gene.3583 | 412 | 4-coumarate--CoA ligase-like [Leptinotarsa decemlineata] |
| map00130 Ubiquinone and other terpenoid-quinone biosynthesis | Gene.3583 | 440 | 4-coumarate--CoA ligase-like [Leptinotarsa decemlineata] |
| map00130 Ubiquinone and other terpenoid-quinone biosynthesis | Gene.3583 | 583 | 4-coumarate--CoA ligase-like [Leptinotarsa decemlineata] |
| map00130 Ubiquinone and other terpenoid-quinone biosynthesis | Gene.3583 | 326 | 4-coumarate--CoA ligase-like [Leptinotarsa decemlineata] |
| map00130 Ubiquinone and other terpenoid-quinone biosynthesis | Gene.3583 | 381 | 4-coumarate--CoA ligase-like [Leptinotarsa decemlineata] |
| map00130 Ubiquinone and other terpenoid-quinone biosynthesis | Gene.3583 | 550 | 4-coumarate--CoA ligase-like [Leptinotarsa decemlineata] |
| map04664 Fc epsilon RI signaling pathway | Gene.7526 | 96 | ras-related protein Rac1 [Anoplophora glabripennis] |
| map04966 Collecting duct acid secretion | Gene.2318 | 109 | PREDICTED: LOW QUALITY PROTEIN: V-type proton ATPase 116 kDa subunit a-like [Aethina tumida] |
| map04966 Collecting duct acid secretion | Gene.2318 | 50 | PREDICTED: LOW QUALITY PROTEIN: V-type proton ATPase 116 kDa subunit a-like [Aethina tumida] |
| map04966 Collecting duct acid secretion | Gene.2318 | 522 | PREDICTED: LOW QUALITY PROTEIN: V-type proton ATPase 116 kDa subunit a-like [Aethina tumida] |
| map04966 Collecting duct acid secretion | Gene.2318 | 76 | PREDICTED: LOW QUALITY PROTEIN: V-type proton ATPase 116 kDa subunit a-like [Aethina tumida] |
| map04966 Collecting duct acid secretion | Gene.2318 | 266 | PREDICTED: LOW QUALITY PROTEIN: V-type proton ATPase 116 kDa subunit a-like [Aethina tumida] |
| map04966 Collecting duct acid secretion | Gene.6879 | 42 | V-type proton ATPase subunit E [Leptinotarsa decemlineata] |
| map04966 Collecting duct acid secretion | Gene.6879 | 59 | V-type proton ATPase subunit E [Leptinotarsa decemlineata] |
| map04966 Collecting duct acid secretion | Gene.6879 | 156 | V-type proton ATPase subunit E [Leptinotarsa decemlineata] |
| map04966 Collecting duct acid secretion | Gene.6879 | 10 | V-type proton ATPase subunit E [Leptinotarsa decemlineata] |
| map04966 Collecting duct acid secretion | Gene.6879 | 68 | V-type proton ATPase subunit E [Leptinotarsa decemlineata] |
| map04966 Collecting duct acid secretion | Gene.246 | 39 | PREDICTED: V-type proton ATPase subunit d [Tribolium castaneum] |
| map04966 Collecting duct acid secretion | Gene.5868 | 50 | PREDICTED: V-type proton ATPase subunit D [Polistes canadensis] |
| map04966 Collecting duct acid secretion | Gene.5862 | 8 | "V-type proton ATPase subunit F, partial [Asbolus verrucosus]" |
| map04966 Collecting duct acid secretion | Gene.5862 | 106 | "V-type proton ATPase subunit F, partial [Asbolus verrucosus]" |
| map04966 Collecting duct acid secretion | Gene.7366 | 21 | PREDICTED: V-type proton ATPase subunit G [Nicrophorus vespilloides] |
| map04966 Collecting duct acid secretion | Gene.7366 | 61 | PREDICTED: V-type proton ATPase subunit G [Nicrophorus vespilloides] |
| map04966 Collecting duct acid secretion | Gene.7366 | 37 | PREDICTED: V-type proton ATPase subunit G [Nicrophorus vespilloides] |
| map04966 Collecting duct acid secretion | Gene.1524 | 445 | V-type proton ATPase subunit B [Galleria mellonella] |
| map04966 Collecting duct acid secretion | Gene.6297 | 155 | PREDICTED: V-type proton ATPase subunit C isoform X3 [Tribolium castaneum] |
| map04966 Collecting duct acid secretion | Gene.6297 | 139 | PREDICTED: V-type proton ATPase subunit C isoform X3 [Tribolium castaneum] |
| map04966 Collecting duct acid secretion | Gene.6297 | 267 | PREDICTED: V-type proton ATPase subunit C isoform X3 [Tribolium castaneum] |
| map04966 Collecting duct acid secretion | Gene.6297 | 262 | PREDICTED: V-type proton ATPase subunit C isoform X3 [Tribolium castaneum] |
| map04966 Collecting duct acid secretion | Gene.664 | 50 | PREDICTED: V-type proton ATPase 116 kDa subunit a isoform 1 isoform X3 [Tribolium castaneum] |
| map04966 Collecting duct acid secretion | Gene.3482 | 588 | V-type proton ATPase catalytic subunit A [Leptinotarsa decemlineata] |
| map04966 Collecting duct acid secretion | Gene.3482 | 533 | V-type proton ATPase catalytic subunit A [Leptinotarsa decemlineata] |
| map04966 Collecting duct acid secretion | Gene.3482 | 130 | V-type proton ATPase catalytic subunit A [Leptinotarsa decemlineata] |
| map04966 Collecting duct acid secretion | Gene.3482 | 593 | V-type proton ATPase catalytic subunit A [Leptinotarsa decemlineata] |
| map04966 Collecting duct acid secretion | Gene.3482 | 584 | V-type proton ATPase catalytic subunit A [Leptinotarsa decemlineata] |
| map04966 Collecting duct acid secretion | Gene.3482 | 513 | V-type proton ATPase catalytic subunit A [Leptinotarsa decemlineata] |
| map00040 Pentose and glucuronate interconversions | Gene.2064 | 292 | "PREDICTED: trans-1,2-dihydrobenzene-1,2-diol dehydrogenase [Tribolium castaneum]" |
| map00040 Pentose and glucuronate interconversions | Gene.3731 | 221 | PREDICTED: UDP-glucuronosyltransferase 2B2 [Tribolium castaneum] |
| map00040 Pentose and glucuronate interconversions | Gene.3734 | 395 | PREDICTED: UDP-glucuronosyltransferase 2B7 isoform X1 [Tribolium castaneum] |
| map00040 Pentose and glucuronate interconversions | Gene.3734 | 305 | PREDICTED: UDP-glucuronosyltransferase 2B7 isoform X1 [Tribolium castaneum] |
| map00040 Pentose and glucuronate interconversions | Gene.3734 | 400 | PREDICTED: UDP-glucuronosyltransferase 2B7 isoform X1 [Tribolium castaneum] |
| map00040 Pentose and glucuronate interconversions | Gene.3734 | 378 | PREDICTED: UDP-glucuronosyltransferase 2B7 isoform X1 [Tribolium castaneum] |
| map00040 Pentose and glucuronate interconversions | Gene.3734 | 406 | PREDICTED: UDP-glucuronosyltransferase 2B7 isoform X1 [Tribolium castaneum] |
| map00040 Pentose and glucuronate interconversions | Gene.3223 | 72 | Aldose reductase-like Protein [Tribolium castaneum] |
| map00040 Pentose and glucuronate interconversions | Gene.3223 | 264 | Aldose reductase-like Protein [Tribolium castaneum] |
| map00040 Pentose and glucuronate interconversions | Gene.3223 | 202 | Aldose reductase-like Protein [Tribolium castaneum] |
| map00040 Pentose and glucuronate interconversions | Gene.3223 | 167 | Aldose reductase-like Protein [Tribolium castaneum] |
| map00040 Pentose and glucuronate interconversions | Gene.2176 | 48 | UDP-glucuronosyltransferase 1-9-like isoform X2 [Leptinotarsa decemlineata] |
| map00040 Pentose and glucuronate interconversions | Gene.8787 | 296 | UDPGT domain containing protein [Asbolus verrucosus] |
| map00040 Pentose and glucuronate interconversions | Gene.8787 | 208 | UDPGT domain containing protein [Asbolus verrucosus] |
| map00040 Pentose and glucuronate interconversions | Gene.8787 | 442 | UDPGT domain containing protein [Asbolus verrucosus] |
| map00040 Pentose and glucuronate interconversions | Gene.1161 | 74 | PREDICTED: UTP--glucose-1-phosphate uridylyltransferase isoform X1 [Tribolium castaneum] |
| map00040 Pentose and glucuronate interconversions | Gene.1161 | 71 | PREDICTED: UTP--glucose-1-phosphate uridylyltransferase isoform X1 [Tribolium castaneum] |
| map00040 Pentose and glucuronate interconversions | Gene.1161 | 424 | PREDICTED: UTP--glucose-1-phosphate uridylyltransferase isoform X1 [Tribolium castaneum] |
| map00040 Pentose and glucuronate interconversions | Gene.1161 | 305 | PREDICTED: UTP--glucose-1-phosphate uridylyltransferase isoform X1 [Tribolium castaneum] |
| map00040 Pentose and glucuronate interconversions | Gene.1161 | 294 | PREDICTED: UTP--glucose-1-phosphate uridylyltransferase isoform X1 [Tribolium castaneum] |
| map00040 Pentose and glucuronate interconversions | Gene.1161 | 434 | PREDICTED: UTP--glucose-1-phosphate uridylyltransferase isoform X1 [Tribolium castaneum] |
| map00040 Pentose and glucuronate interconversions | Gene.1161 | 312 | PREDICTED: UTP--glucose-1-phosphate uridylyltransferase isoform X1 [Tribolium castaneum] |
| map00040 Pentose and glucuronate interconversions | Gene.1161 | 19 | PREDICTED: UTP--glucose-1-phosphate uridylyltransferase isoform X1 [Tribolium castaneum] |
| map00040 Pentose and glucuronate interconversions | Gene.1161 | 284 | PREDICTED: UTP--glucose-1-phosphate uridylyltransferase isoform X1 [Tribolium castaneum] |
| map00040 Pentose and glucuronate interconversions | Gene.1779 | 101 | PREDICTED: lambda-crystallin [Tribolium castaneum] |
| map00040 Pentose and glucuronate interconversions | Gene.1779 | 263 | PREDICTED: lambda-crystallin [Tribolium castaneum] |
| map00040 Pentose and glucuronate interconversions | Gene.2052 | 422 | "UDP-glucuronosyltransferase 2C1-like, partial [Asbolus verrucosus]" |
| map00040 Pentose and glucuronate interconversions | Gene.4396 | 69 | PREDICTED: 2-hydroxyacylsphingosine 1-beta-galactosyltransferase-like [Tribolium castaneum] |
| map00040 Pentose and glucuronate interconversions | Gene.4396 | 317 | PREDICTED: 2-hydroxyacylsphingosine 1-beta-galactosyltransferase-like [Tribolium castaneum] |
| map00040 Pentose and glucuronate interconversions | Gene.4396 | 49 | PREDICTED: 2-hydroxyacylsphingosine 1-beta-galactosyltransferase-like [Tribolium castaneum] |
| map00040 Pentose and glucuronate interconversions | Gene.4396 | 259 | PREDICTED: 2-hydroxyacylsphingosine 1-beta-galactosyltransferase-like [Tribolium castaneum] |
| map00040 Pentose and glucuronate interconversions | Gene.2563 | 97 | PREDICTED: aldose reductase [Tribolium castaneum] |
| map00040 Pentose and glucuronate interconversions | Gene.2124 | 330 | PREDICTED: sorbitol dehydrogenase [Tribolium castaneum] |
| map00040 Pentose and glucuronate interconversions | Gene.2124 | 83 | PREDICTED: sorbitol dehydrogenase [Tribolium castaneum] |
| map00040 Pentose and glucuronate interconversions | Gene.2124 | 210 | PREDICTED: sorbitol dehydrogenase [Tribolium castaneum] |
| map00040 Pentose and glucuronate interconversions | Gene.2124 | 322 | PREDICTED: sorbitol dehydrogenase [Tribolium castaneum] |
| map00040 Pentose and glucuronate interconversions | Gene.2124 | 316 | PREDICTED: sorbitol dehydrogenase [Tribolium castaneum] |
| map00040 Pentose and glucuronate interconversions | Gene.2124 | 324 | PREDICTED: sorbitol dehydrogenase [Tribolium castaneum] |
| map00040 Pentose and glucuronate interconversions | Gene.2124 | 344 | PREDICTED: sorbitol dehydrogenase [Tribolium castaneum] |
| map00040 Pentose and glucuronate interconversions | Gene.2124 | 219 | PREDICTED: sorbitol dehydrogenase [Tribolium castaneum] |
| map00040 Pentose and glucuronate interconversions | Gene.2124 | 351 | PREDICTED: sorbitol dehydrogenase [Tribolium castaneum] |
| map00040 Pentose and glucuronate interconversions | Gene.2140 | 401 | PREDICTED: UDP-glucuronosyltransferase 2B10-like [Tribolium castaneum] |
| map00040 Pentose and glucuronate interconversions | Gene.2140 | 80 | PREDICTED: UDP-glucuronosyltransferase 2B10-like [Tribolium castaneum] |
| map00040 Pentose and glucuronate interconversions | Gene.2140 | 425 | PREDICTED: UDP-glucuronosyltransferase 2B10-like [Tribolium castaneum] |
| map00040 Pentose and glucuronate interconversions | Gene.2140 | 410 | PREDICTED: UDP-glucuronosyltransferase 2B10-like [Tribolium castaneum] |
| map00040 Pentose and glucuronate interconversions | Gene.2569 | 315 | "1,5-anhydro-D-fructose reductase-like Protein [Tribolium castaneum]" |
| map00040 Pentose and glucuronate interconversions | Gene.2569 | 27 | "1,5-anhydro-D-fructose reductase-like Protein [Tribolium castaneum]" |
| map00040 Pentose and glucuronate interconversions | Gene.2569 | 4 | "1,5-anhydro-D-fructose reductase-like Protein [Tribolium castaneum]" |
| map00040 Pentose and glucuronate interconversions | Gene.2569 | 60 | "1,5-anhydro-D-fructose reductase-like Protein [Tribolium castaneum]" |
| map00040 Pentose and glucuronate interconversions | Gene.2569 | 56 | "1,5-anhydro-D-fructose reductase-like Protein [Tribolium castaneum]" |
| map00040 Pentose and glucuronate interconversions | Gene.2569 | 70 | "1,5-anhydro-D-fructose reductase-like Protein [Tribolium castaneum]" |
| map00040 Pentose and glucuronate interconversions | Gene.2569 | 169 | "1,5-anhydro-D-fructose reductase-like Protein [Tribolium castaneum]" |
| map00040 Pentose and glucuronate interconversions | Gene.2569 | 9 | "1,5-anhydro-D-fructose reductase-like Protein [Tribolium castaneum]" |
| map00040 Pentose and glucuronate interconversions | Gene.1050 | 121 | PREDICTED: 2-hydroxyacylsphingosine 1-beta-galactosyltransferase-like [Tribolium castaneum] |
| map00040 Pentose and glucuronate interconversions | Gene.7644 | 70 | PREDICTED: aldose reductase [Tribolium castaneum] |
| map00040 Pentose and glucuronate interconversions | Gene.7644 | 151 | PREDICTED: aldose reductase [Tribolium castaneum] |
| map00040 Pentose and glucuronate interconversions | Gene.7644 | 61 | PREDICTED: aldose reductase [Tribolium castaneum] |
| map00040 Pentose and glucuronate interconversions | Gene.2175 | 192 | PREDICTED: 2-hydroxyacylsphingosine 1-beta-galactosyltransferase-like [Tribolium castaneum] |
| map00040 Pentose and glucuronate interconversions | Gene.2178 | 47 | UDPGT and/or Glyco tran 28 C domain containing protein [Asbolus verrucosus] |
| map05202 Transcriptional misregulation in cancer | Gene.453 | 382 | DEAD-box ATP-dependent RNA helicase 20-like [Onthophagus taurus] |
| map05202 Transcriptional misregulation in cancer | Gene.8418 | 74 | PREDICTED: 15-hydroxyprostaglandin dehydrogenase [NAD(+)]-like [Aethina tumida] |
| map05202 Transcriptional misregulation in cancer | Gene.2167 | 59 | PREDICTED: 15-hydroxyprostaglandin dehydrogenase [NAD(+)] [Tribolium castaneum] |
| map05202 Transcriptional misregulation in cancer | Gene.2167 | 233 | PREDICTED: 15-hydroxyprostaglandin dehydrogenase [NAD(+)] [Tribolium castaneum] |
| map05202 Transcriptional misregulation in cancer | Gene.2167 | 53 | PREDICTED: 15-hydroxyprostaglandin dehydrogenase [NAD(+)] [Tribolium castaneum] |
| map05202 Transcriptional misregulation in cancer | Gene.2167 | 64 | PREDICTED: 15-hydroxyprostaglandin dehydrogenase [NAD(+)] [Tribolium castaneum] |
| map05202 Transcriptional misregulation in cancer | Gene.2167 | 45 | PREDICTED: 15-hydroxyprostaglandin dehydrogenase [NAD(+)] [Tribolium castaneum] |
[truncated: 648,245 more chars]
